# Meta-analytic prevalence of comorbid mental disorders in individuals at clinical high risk of psychosis: the case for transdiagnostic assessment

**DOI:** 10.1038/s41380-023-02029-8

**Published:** 2023-06-09

**Authors:** Marco Solmi, Livia Soardo, Simi Kaur, Matilda Azis, Anna Cabras, Marco Censori, Luigi Fausti, Filippo Besana, Gonzalo Salazar de Pablo, Paolo Fusar-Poli

**Affiliations:** 1 Department of Psychiatry, University of Ottawa, Ottawa, ON Canada; 2 On Track, First Episode Psychosis Program, Department of Mental Health, The Ottawa Hospital, Ottawa, ON Canada; 3 Ottawa Hospital Research Institute (OHRI) Clinical Epidemiology Program University of Ottawa Ottawa Ontario, Ottawa, ON Canada; 4 School of Epidemiology and Public Health, Faculty of Medicine, University of Ottawa, Ottawa, ON Canada; 5 Early Psychosis: Interventions and Clinical-detection (EPIC) Lab, Institute of Psychiatry, Psychology & Neuroscience, Department of Psychosis Studies, King’s College London, London, United Kingdom; 6 Department of Child and Adolescent Psychiatry, Charité Universitätsmedizin, Berlin, Germany; 7 Department of Brain and Behavioral Sciences, University of Pavia, Pavia, Italy; 8 Sapienza University of Rome, Department of Neurology and Psychiatry, Roma, Italy; 9 Department of Neuroscience (DNS), University of Padova, Padua, Italy; 10 Dipartimento di Salute Mentale, Azienda ULSS 3 Serenissima, Venezia, Italy; 11 Department of Child and Adolescent Psychiatry, Institute of Psychiatry, Psychology & Neuroscience, King’s College London UK, London, UK; 12 Child and Adolescent Mental Health Services, South London and Maudsley NHS Foundation Trust, London, UK; 13 Institute of Psychiatry and Mental Health. Department of Child and Adolescent Psychiatry, Hospital General Universitario Gregorio Marañón School of Medicine, Universidad Complutense, Instituto de Investigación Sanitaria Gregorio Marañón (IiSGM), CIBERSAM, Madrid, Spain

**Keywords:** Schizophrenia, Depression, Addiction

## Abstract

Comorbid mental disorders in subjects at clinical high risk for psychosis (CHR-P) may impact preventive care. We conducted a PRISMA/MOOSE-compliant systematic meta-analysis, searching PubMed/PsycInfo up to June 21st, 2021 for observational studies/randomized controlled trials reporting on comorbid DSM/ICD-mental disorders in CHR-P subjects (protocol). The primary and secondary outcomes were baseline and follow-up prevalence of comorbid mental disorders. We also explored the association of comorbid mental disorders compared with CHR-P versus psychotic/non-psychotic control groups, their impact on baseline functioning and transition to psychosis. We conducted random-effects meta-analyses, meta-regression, and assessed heterogeneity/publication bias/quality (Newcastle Ottawa Scale, NOS). We included 312 studies (largest meta-analyzed sample = 7834, any anxiety disorder, mean age = 19.98 (3.40), females = 43.88%, overall NOS > 6 in 77.6% of studies). The prevalence was 0.78 (95% CI = 0.73–0.82, *k* = 29) for any comorbid non-psychotic mental disorder, 0.60 (95% CI = 0.36–0.84, *k* = 3) for anxiety/mood disorders, 0.44 (95% CI = 0.39–0.49, *k* = 48) for any mood disorders, 0.38 (95% CI = 0.33–0.42, *k* = 50) for any depressive disorder/episode, 0.34 (95% CI = 0.30–0.38, *k* = 69) for any anxiety disorder, 0.30 (95% CI 0.25–0.35, *k* = 35) for major depressive disorders, 0.29 (95% CI, 0.08–0.51, *k* = 3) for any trauma-related disorder, 0.23 (95% CI = 0.17–0.28, *k* = 24) for any personality disorder, and <0.23 in other mental disorders (I^2^ > 50% in 71.01% estimates). The prevalence of any comorbid mental disorder decreased over time (0.51, 95% CI = 0.25–0.77 over 96 months), except any substance use which increased (0.19, 95% CI = 0.00–0.39, *k* = 2, >96 months). Compared with controls, the CHR-P status was associated with a higher prevalence of anxiety, schizotypal personality, panic, and alcohol use disorders (OR from 2.90 to 1.54 versus without psychosis), a higher prevalence of anxiety/mood disorders (OR = 9.30 to 2.02) and lower prevalence of any substance use disorder (OR = 0.41, versus psychosis). Higher baseline prevalence of alcohol use disorder/schizotypal personality disorder was negatively associated with baseline functioning (beta from −0.40 to −0.15), while dysthymic disorder/generalized anxiety disorder with higher functioning (beta 0.59 to 1.49). Higher baseline prevalence of any mood disorder/generalized anxiety disorder/agoraphobia (beta from −2.39 to −0.27) was negatively associated with transition to psychosis. In conclusion, over three-quarters of CHR-P subjects have comorbid mental disorders, which modulate baseline functionig and transition to psychosis. Transdiagnostic mental health assessment should be warranted in subjects at CHR-P.

## Introduction

The clinical high risk for psychosis (CHR-P) is defined by the concomitant presence of attenuated psychotic positive symptoms that do not last long enough or that are not severe enough to meet full blown psychotic disorder DSM/ICD diagnosis [1–6]. A recent meta-analysis estimated that the epidemiological prevalence of CHR-P status is around 1.7% in the general population, and 19.2% in clinical samples [7]. Several psychometric tools have been developed to assess young people with potential CHR-P features; the two most frequently used are the the Comprehensive Assessment of At Risk Mental States (CAARMS) [8], and the Structured Interview for Psychosis-risk Syndromes (SIPS) [9]. Such psychometric tools have demonstrated excellent prognostic accuracy (AUC = 0.85) for predicting psychosis when used in clinical samples (largely driven by an outstanding capacity to rule out psychosis risk) [10], albeit at a group-level only. On the other hand, when used in non clinical samples, these tools have poor clinical utility [10].

Subjects with CHR-P have complex and heterogeneous clinical presentations with frequent non-psychotic comorbid mental disorders [11, 12] beyond their attenuated psychotic symptoms, such as negative, and affective symptoms, mood, anxiety, obsessive compulsive and personality disorders. However the prevalence of these disorders is highly variables in CHR-P samples. The clinical impact of comorbid disorders on the level of functioning in CHR-P individuals is not fully understood [13]. The clinical evolution over time of the CHR-P status is similarly heterogeneous. Overall, CHR-P individuals have a risk of transitioning to a first episode of psychosis of 20% by two years and 35% by 10 years, which only plateaus after year 4 [5, 14]. There is also contrasting evidence that baseline comorbid mental disorders impact risk of transition to psychosis in CHR-P individuals, with some studies showing that depression increases the risk [15, 16], or that anxiety decreases it [16], and other studies showing no significant association [17]. Beyond transition to psychosis, functioning tends to improve, but only less than half of the baseline CHR-P individuals fully remit after over three years [18, 19]. Most of those who will not develop psychosis at follow-up will present persistent comorbid mental disorders [13].

After an earlier meta-analysis that investigated the prevalence of comorbid mental disorder in people with CHR-P [11] and their clincial impact on baseline functioning and transition to psychosis, many more original studies have been published. Furthermore, that previous meta-analysis did not investigate the prevalence of comorbid mental disordes at follow-up. Given the contrasting findings on these areas, an updated evidence based appraisal is required. Quantifying the magnitude and variablity of the prevalence of comorbid mental disorders in subjects with CHR-P, and their clinical impact, is an essential step towards more accurate clinical assessments, prognostication and tailored preventive interventions in this patient population.

## Methods

Detailed methods are reported in Supplementary Methods section.

### Search strategy and selection criteria

We conducted a PRISMA 2020-compliant [20] and MOOSE-compliant [21] (Supplementary Tables 1 and 2) systematic search of PubMed and PsycInfo, up to June 26^th^, 2021 (key in Supplementary Methods), plus manual search (a-priori protocol). Title/abstract, and full text of those eligible after title/abstract assessment were screened independently by two authors (GS, LS, SK, MA, AC, MC, LF, FB) (a third author resolved any conflict – MS, PFP). The reason for exclusion of articles after full text assessment is available in Supplementary Appendix 2 and 3.

Inclusion criteria were: i) observational studies (cross-sectional or longitudinal) and randomized controlled trials (RCTs) (given previous evidence indicating that the risk of transitioning to psychosis do not significantly differ between cohort studies and the control arm of RCTs in this patient population, therefore suggesting no substantial sampling biases in RCT designs [14]), ii) that reported on subjects meeting CHR-P criteria as per established psychometric instruments (Supplementary Methods), iii) and reported on the prevalence of any mental disorders established according to DSM-any version [22], ICD-any version [23], or validated scales employing cut-offs that map onto DSM or ICD diagnostic categories, iv) and that were published in English language. The type of comorbid mental disorder was classified as reported by authors of eligible studies, and each disorder, spectrum, or combination of disorders/spectra was considered as a separate outcome (Supplementary Table 1, Supplementary Methods).

We excluded i) reviews, ii) studies not assessing the CHR-P state with established psychometric instruments, iii) not assessing mental disorders with DSM/ICD/validated criteria/scales, iv) in language other-than-English. We included the largest among overlapping cohorts/outcomes (i.e. if more than one study from the same center reported on the same population and outcome, we only retained the larger sample).

### Outcomes and data extraction

The primary outcome was the baseline prevalence of comorbid mental disorders in CHR-P individuals. Secondary outcomes were the prevalence of comorbid mental disorders at follow-up, the association of baseline comorbid mental disorders with CHR-P status, when compared to non-psychotic and psychotic control groups, in studies reporting these data. The non-psychotic control group included those undergoing a CHR-P assessment but eventually not meeting CHR-P criteria, or any other population where mental disorders were reported even without undergoing CHR-P status assessment. The psychotic control group included those diagnosed with a DSM/ICD-any version psychotic disorder, regardless if they were or were not previously meeting CHR-P criteria. Secondary outcomes also included the metaregression association of baseline comorbid mental disorders with baseline functioning, and with transition to psychosis at follow-up in studies reporting these outcomes.

The same authors that performend the screening, also extracted the data. From each included study we extracted the following variables: author, year, country, study design, sample size, age, sex, CHR-P criteria, mental disorders diagnostic criteria, prevalence of each mental disorder at any time point, baseline functioning, duration of follow-up, risk of transition to psychosis at follow-up.

### Quality assessment

Studies’ quality was assessed with the Newcastle-Ottawa scale (NOS, Supplementary Methods) [24].

### Statistical analyses

The primary effect size measure was the baseline prevalence of comorbid mental disorders. The secondary outcomes were investigated with the meta-analytic OR of the prevalence of comorbid mental disorders in CHR-P individuals compared to control groups. Transition to psychosis was measured at 6–24, 24–48, 48–96, and more than 96 months of follow-up. Other secondary outcomes were investigated with meta-regression analyses (when at least ten studies provided data on both the moderator and the outcome) testing whether the baseline prevalence of comorbid mental disorders was associated with baseline functioning as well as transition to psychosis at follow-up. For these analyses we reported the average follow-up time across the pooled studies, reported by each individual study.

We conducted a random-effects [25] meta-analysis (Comprehensive Meta-Analysis software v.3) when at least two studies reported on the same outcome at the same time point. Heterogeneity was measured with Q and I^2^ statistics, while publication bias with meta-regression with sample size for prevalence meta-analysis, while with Egger’s test for comparative meta-analysis, as well as with trim-and-fill procedure to calculate corrected OR if Egger’s *p* value <0.1.

## Results

### Search results, sample characteristics and quality of included studies

**Fig. 1:**
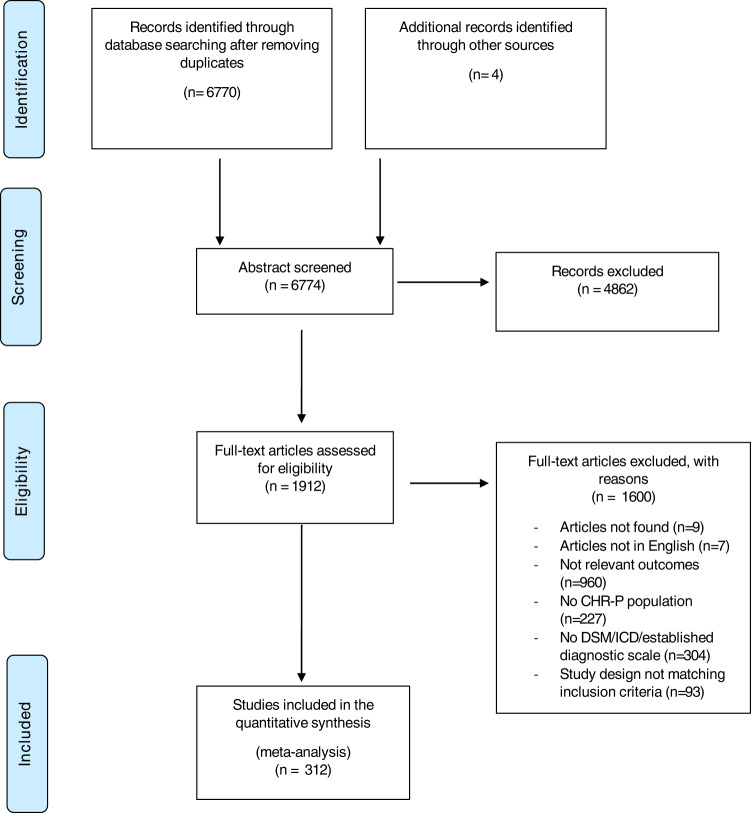
PRISMA figure, study selection flow.

Out of 6774 records initially screened, we assessed the full text of 1912 studies, of which 1600 were excluded after full-text assessment (Fig. 1). Reasons for exclusion of these studies are available in the Supplementary Appendix 2 and 3. We ultimately included 312 publications in this meta-analysis, reporting on a maximum of 7834 subjects (for the outcome any anxiety disorder), with a mean age of 19.98 (SD3.40); 43.88% were females. The detailed characteristics of the included publications, with all references, are available in Supplementary Table 3. Overall, 97 studies were cross-sectional, 200 were cohort studies, and 15 were RCTs. Overall, 32 publications came from multiple countries, 64 from the USA, 38 from Australia, 26 from the UK, 25 from Germany, 22 from Italy, 22 from Switzerland, 13 from the Netherlands, 13 from South Korea, 10 from Singapore, 9 from Japan, six from Finland, five from Canada, Denmark, and Poland, three from Austria, two from China, Norway, and Spain, and one from Brazil, Chile, France, Greece, Hong Kong, Israel, Russia, Turkey (Supplementary Fig. 1). The follow-up of CHR-P individuals ranged form 2.3 to 196 months. The median NOS score was 7, with 242 (77.6%) studies scoring 7 or more.

### Baseline prevalence of comorbid mental disorders in CHR-P individuals

The results of the primary analysis investigating the baseline prevalence of 70 different (combinations of) comorbid mental disorders in CHR-P are reported in Table 1. Pooling data from 29 studies, the results showed that over three-quarters (0.78, 95% CI 0.73–0.82) of subjects had any comorbid mental disorder. The higher prevalence emerged for anxiety/mood disorders (0.60, 95% CI 0.36–0.84), progressively decreasing through any mood disorders (0.44, 95% CI 0.39–0.49), any depressive disorder/episode (0.38, 95% CI 0.33–0.42), any anxiety disorder (0.34, 95% CI 0.30–0.38), major depressive disorder (0.30, 95% CI 0.25–0.35), any trauma-related disorder (0.29, 95% CI, 0.08–0.51), any personality disorder (0.23, 95% CI 0.17–0.28), to other disorders present in less than 23% of CHR-P individuals. Heterogeneity was high (i.e. I^2^ > 50%) in 48 meta-analytic estimates (68.57%).

**Table 1:** Meta-analytical prevalence of baseline comorbid mental disorders in CHR-P individuals.

Outcome	Prevalence	95 % CI		Q	I^2^	Tau^2^	*N* of studies	Subjects	Meta-regression with sample size			
									Beta	95% CI		*P* value
Any non-psychotic mental disorder	0.78	0.73	0.82	58.23	51.92	0.01	29	4032	n.s.			
Anxiety/mood disorder	0.60	0.36	0.84	20.46	90.23	0.04	3	413	n.s.			
Any mood disorder	0.44	0.39	0.49	315.54	85.11	0.02	48	4917	n.s.			
Any depressive disorder/episode	0.38	0.33	0.42	383.71	87.23	0.02	50	6518	<0.001	0.000	0.001	0.018
Any anxiety disorder	0.34	0.30	0.38	786.10	91.35	0.03	69	7834	n.s.			
Major depressive disorder	0.30	0.25	0.35	319.47	89.36	0.02	35	4111	n.s.			
Any trauma disorder	0.29	0.08	0.51	53.15	96.24	0.03	3	480	n.s.			
Any personality disorder	0.23	0.17	0.28	453.61	95.93	0.02	24	2523	n.s.			
Sexual trauma	0.22	−0.02	0.47	14.57	93.14	0.03	2	292	d.n.a.			
Any pervasive developmental disorder	0.15	0.07	0.23	0.09	0.00	0.00	2	87	d.n.a.			
Neurotic, stress-related and somatoform disorders	0.14	0.06	0.23	0.15	0.00	0.00	2	76	d.n.a.			
Social anxiety disorder (or social phobia)	0.14	0.11	0.16	135.31	78.57	0.00	30	4134	n.s.			
ADHD	0.13	0.09	0.17	161.84	85.79	0.01	24	2326	n.s.			
Any behavioral disorder	0.13	0.04	0.22	18.52	78.40	0.01	5	436	n.s.			
Cannabis use disorder	0.13	0.10	0.16	207.99	88.46	0.01	25	3119	n.s.			
Pervasive developmental disorder NOS	0.12	0.01	0.24	13.28	84.94	0.01	3	198	n.s.			
Schizotypal personality disorder	0.12	0.09	0.14	327.29	89.61	0.00	35	3819	n.s.			
Phobias NOS	0.12	0.07	0.16	0.62	0.00	0.00	2	185	d.n.a.			
Any substance use disorder	0.11	0.09	0.13	304.62	85.56	0.00	45	4897	n.s.			
Avoidant personality disorder	0.11	0.07	0.14	44.73	79.88	0.00	10	1336	n.s.			
Borderline personality disorder	0.10	0.07	0.14	158.24	89.26	0.00	18	2005	n.s.			
Conduct disorder	0.10	0.05	0.14	53.04	81.15	0.00	11	919	n.s.			
ODD	0.09	0.04	0.13	65.95	87.87	0.00	9	1546	n.s.			
Panic disorder	0.08	0.06	0.10	165.42	83.68	0.00	28	3757	<0.001	0.000	0.000	0.022
GAD	0.08	0.06	0.10	79.46	69.80	0.00	25	3660	n.s.			
Any neurodevelopmental disorder	0.08	0.03	0.12	67.01	91.05	0.00	7	1040	n.s.			
Anxiety disorder NOS	0.08	0.05	0.10	83.88	83.33	0.00	15	2217	n.s.			
Alcohol use disorder	0.08	0.05	0.10	184.94	88.10	0.00	23	2780	n.s.			
Depressive disorder NOS	0.07	0.04	0.10	88.26	85.27	0.00	14	1801	n.s.			
OCD	0.07	0.06	0.08	125.75	63.13	0.00	47	5717	n.s.			
Substance use disorder NOS	0.07	0.03	0.10	82.22	92.70	0.00	7	1707	n.s.			
Specific phobia	0.06	0.05	0.08	66.47	74.42	0.00	18	3104	n.s.			
Paranoid personality disorder	0.06	0.03	0.09	19.11	63.38	0.00	8	973	n.s.			
Other disorders NOS	0.06	0.04	0.08	124.90	84.79	0.00	20	3252	n.s.			
Obsessive personality disorder	0.06	0.03	0.09	14.84	66.32	0.00	6	859	<0.001	0.000	0.000	0.000
Relational disorder	0.05	−0.03	0.13	2.85	64.97	0.00	2	246	d.n.a.			
Dysthymia	0.05	0.04	0.06	42.34	40.96	0.00	26	3635	n.s.			
PTSD	0.05	0.04	0.07	131.48	75.66	0.00	33	4395	n.s.			
Learning disorder	0.05	0.03	0.07	5.07	60.53	0.00	3	1149	<0.001	0.000	0.000	0.032
Mood disorder NOS	0.05	0.01	0.08	27.24	85.31	0.00	5	786	n.s.			
Adjustment disorder	0.05	0.03	0.06	86.94	74.69	0.00	23	2492	n.s.			
Other bipolar disorder (e.g. BD-NOS, BD-II)	0.04	0.03	0.05	117.70	74.51	0.00	31	4073	n.s.			
Schizoid personality disorder	0.04	0.02	0.06	24.96	55.94	0.00	12	1195	n.s.			
Agoraphobia	0.04	0.02	0.05	74.66	78.57	0.00	17	2939	n.s.			
Stimulants use disorder	0.03	0.01	0.06	0.14	0.00	0.00	2	199	d.n.a.			
Personality disorder NOS	0.03	0.02	0.05	0.76	0.00	0.00	3	564	n.s.			
Hypochondriasis	0.03	0.00	0.07	14.44	79.23	0.00	4	405	n.s.			
Autism spectrum disorder	0.03	0.01	0.05	20.48	56.06	0.00	10	1475	n.s.			
Dependent personality disorder	0.03	0.01	0.05	9.81	59.24	0.00	5	801	−0.000	−0.000	−0.000	0.020
Dissociative disorders	0.03	0.02	0.04	4.51	0.00	0.00	8	791	n.s.			
Antisocial personality disorder	0.03	0.00	0.05	14.02	71.46	0.00	5	791	n.s.			
Bipolar disorder type I	0.03	0.02	0.04	21.20	29.25	0.00	16	2317	n.s.			
Any eating disorder	0.03	0.02	0.03	49.82	41.79	0.00	30	3843	n.s.			
Somatoform disorders	0.02	0.01	0.03	50.09	58.08	0.00	22	3309	n.s.			
Histrionic personality disorder	0.02	−0.00	0.02	5.63	46.74	0.00	4	772	n.s.			
Body dysmorphic disorder	0.02	0.01	0.03	2.31	0.00	0.00	7	706	n.s.			
Impulsive control disorder	0.02	−0.00	0.04	0.00	0.00	0.00	2	189	d.n.a.			
Narcissistic personality disorder	0.01	0.00	0.03	2.63	24.08	0.00	3	643	n.s.			
Binge-eating disorder	0.01	−0.00	0.03	3.12	35.96	0.00	3	430	n.s.			
Cocaine use disorder	0.01	−0.00	0.03	14.90	79.86	0.00	4	1486	n.s.			
Amphetamines use disorder	0.01	−0.00	0.03	12.87	84.46	0.00	3	1457	−0.000	−0.000	−0.000	0.029
Polysubstance use disorder	0.01	−0.00	0.03	12.83	68.82	0.00	5	1361	−0.000	−0.000	−0.000	0.0199
Eating disorder NOS	0.01	0.01	0.02	1.11	0.00	0.00	4	911	n.s.			
Opioid use disorder	0.01	−0.00	0.03	0.57	0.00	0.00	3	223	n.s.			
Depressive personality disorder	0.01	−0.01	0.02	0.55	0.00	0.00	2	158	d.n.a.			
Trichotillomania	0.01	−0.01	0.02	0.73	0.00	0.00	2	187	d.n.a.			
Cyclothymia	0.00	0.00	0.01	0.59	0.00	0.00	2	804	d.n.a.			
Bulimia	0.00	−0.00	0.01	7.80	23.04	0.00	7	1397	−0.000	−0.000	0.000	0.038
Hallucinogen use disorder	0.00	−0.00	0.01	3.96	24.19	0.00	4	1209	n.s.			
Anorexia nervosa	0.00	0.00	0.00	4.88	0.00	0.00	6	1410	n.s.			

*CHR-P* clinical high risk for psychosis, *K* number of studies, *N* sample size, *n.s* not significant, *d.n.a.* does not apply.

### Follow-up prevalence of comorbid mental disorders in CHR-P individuals

**Fig. 2:**
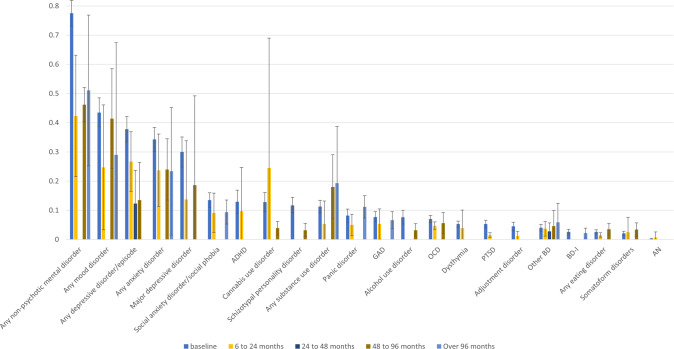
Meta-analytic prevalence of comorbid mental disorders at baseline and follow-up in CHR-P individuals. ADHD attention-deficit/hyperactivity disorder, AN anorexia nervosa, BD-I bipolar disorder type I, GAD generalized anxiety disorder, OCD obsessive-compulsive disorder, Other BD BD-NOS, BD-II, PTSD post-traumatic stress disorder. 95% confidence intervals are displayed.

Results of prevalence of (combinations of) comorbid mental disorders at 6–24 months, 24–48 months, 48–96 months, over 96 months follow-up are summarized in Fig. 2 and are fully reported in Supplementary Tables 4–7, including the sample size and the number of studies included in each analysis. Among disorders for which the baseline prevalence exceeded 23%, any mental disorder estimates decreased from 0.78 (baseline), to 0.42 (6–24 months), 0.46 (48–96 months), 0.51 (more than 96 months), any mood disorders from 0.44 (baseline) to 0.25 (6–24 months), 0.41 (48–96 months), 29 (more than 96 months), and any anxiety disorders from 0.34 (baseline), to 0.24 (6–24 months), 0.24 (48–96 months), 0.23 (more than 96 months). For any depressive disorder/episode, the prevalence among CHR-P subjects went from 0.38 to 0.27 (6–24 months), 0.12 (24–48 months), and 0.14 (48–96 months), and for major depressive disorder from 0.30 at baseline, to 0.14 (6–24 months), and 0.19 (48–96 months). All the other disorders maintained a lower prevalence at different follow-up points, apart from any substance use disorder, which increased from 0.11 (baseline) to 0.19 (more than 96 months) follow-up (longest follow-up based on two studies only).

### Association between baseline comorbid disorders and CHR-P status

Compared with non-psychotic controls, CHR-P subjects display higher prevalence of anxiety disorders not otherwise specified (NOS) (OR = 2.90, 95% CI 1.43–5.87), panic disorder (OR = 2.56, 95% CI 1.06–6.17), any anxiety disorder (OR = 1.75, 95% CI 1.36–2.25), schizotypal personality disorder (OR = 1.54, 95% CI 1.12–2.11), and alcohol use disorder (OR = 1.54, 95% CI 1.04–2.27). No significant associations emerged with the other mental disorders (Supplementary Table 8).

Compared with psychotic controls, CHR-P individuals displayed higher prevalence of social anxiety disorder (OR = 9.28, 95% CI 1.18–73.50), any mood disorder (OR = 4.62, 95% CI 2.23–9.58), any depressive disorder (OR = 2.18, 95% CI 1.75–2.73), any anxiety disorder (OR = 2.02, 95% CI 1.62–2.51), but with lower prevalence of any substance use disorder (OR = 0.41, 95% CI 0.20–0.82). No significant associations emerged with the other 10 mental disorders (Supplementary Table 9).

### Meta-regressions of baseline prevalence of mental disorders and baseline functioning and transition to psychosis at follow-up

As reported in detail in the Table 2, baseline comorbid alcohol use disorder (beta = −0.40, SE = 0.20, *p* = 0.048, *k* = 12), and schizotypal personality disorder (beta = −0.15, SE = 0.06, *p* = 0.010, *k* = 24) were negatively associated with baseline functioning, whilst baseline comorbid dysthymic disorder or generalized anxiety disorder were positively associated with baseline functioning (beta = 1.50, SE = 0.49, *p* = 0.004, *k* = 13; beta = 0.59, SE = 0.30, *p* = 0.050, *k* = 16, respectively). Other disorders did not show statistically significant meta-regression results.

**Table 2:** Meta-regression between the baseline prevalence of comorbid mental disorders and baseline functioning in CHR-P individuals.

Outcome	Coefficient	SE	*P* value	*N* of studies
**Alcohol use disorder**	**−0.40**	**0.20**	**0.05**	**12**
**Schizotypal personality disorder**	**−0.15**	**0.06**	**0.01**	**24**
**Dysthymia**	**1.50**	**0.49**	**0.00**	**13**
**GAD**	**0.59**	**0.30**	**0.05**	**16**
Any eating disorder	−0.23	0.23	0.31	15
Somatoform disorder	−1.16	0.73	0.11	13
PTSD	−0.14	0.18	0.45	21
OCD	−0.12	0.42	0.77	25
Social anxiety disorder (or social phobia)	−0.12	0.16	0.45	15
Any depressive episode/disorder	−0.11	0.08	0.13	31
Cannabis use disorder	−0.11	0.15	0.45	15
Any mood disorder	−0.09	0.08	0.26	26
Any non-psychotic mental disorder	−0.08	0.10	0.45	15
Borderline personality disorder	−0.08	0.09	0.36	12
ADHD	−0.08	0.17	0.66	13
Panic disorder	−0.07	0.16	0.64	14
Major depressive disorder	−0.07	0.07	0.34	19
Any anxiety disorder	−0.01	0.09	0.83	47
Other bipolar disorder (e.g. BD-NOS, BD-II)	0.20	0.07	0.15	18
Other disorders NOS	0.37	0.22	0.10	11
Any personality disorder	0.07	0.12	0.58	12
Any substance use disorder	0.09	0.13	0.52	27

*ADHD* attention-deficit/hyperactivity disorder, *BD* bipolar disorder, *CHR-P* clinical high risk for psychosis, *GAD* generalized anxiety disorder, *NOS* not otherwise specified, *OCD* obsessive-copulsive disorder, *PTSD* post-traumatic stress disorder, *SE* standard error.

The baseline prevalence of any mood disorder (beta = −0.27, SE = 0.05, *p* = 0.007, mean follow-up = 30.72 months), generalized anxiety disorder (beta = −1.01, SE = 0.47, *p* = 0.031, mean follow-up = 36.54 months), agoraphobia (beta = −2.38, SE = 0.95, *p* = 0.012, mean follow-up = 27.69 months) were negatively associated with the risk transition to psychosis at follow-up, while other disorders showed no statistically significant effects (Supplementary Fig. 2, Table 3).

**Table 3:** Meta-regression between baseline prevalence of comorbid mental disorders in CHR-P individuals and transition to psychosis at follow-up.

Outcome	Coefficient	SE	*P* value	*N* of studies	Follow-up, mean^a^	Follow-up, SD
**Any mood disorder**	**−0.27**	**0.05**	**0.01**	**31**	**30.72**	**22.43**
**GAD**	**−1.01**	**0.47**	**0.03**	**15**	**36.54**	**36.24**
**Agoraphobia**	**−2.38**	**0.95**	**0.01**	**10**	**27.69**	**33.56**
Dysthymia	0.73	0.92	0.43	15	39.21	36.71
Somatoform disorder	0.62	1.24	0.62	17	38.83	35.93
Adjustment disorder	0.42	0.82	0.61	13	29.01	16.13
Any personality disorder	0.19	0.18	0.29	12	18.96	9.427
Scizotypal personality disorder	0.15	0.32	0.64	17	29.94	24.07
PTSD	0.14	0.32	0.65	15	29.79	25.72
Any depressive disorder/episode	0.11	0.13	0.40	25	33.81	28.04
Other disorders NOS	0.10	0.72	0.89	13	24.86	17.41
Cannabis use disorder	0.05	0.32	0.88	11	28.86	14.81
Alcohol use disorder	0.03	0.31	0.91	11	28.31	18.15
Any non-psychotic mental disorder	0.03	0.18	0.88	15	31.52	21.82
Any substance use disorder	0.02	0.16	0.89	26	28.52	22.10
Panic disorders	−0.08	0.46	0.86	17	35.77	34.04
Major depressive disorder	−0.12	0.14	0.40	20	32.03	32.48
Any anxiety disorder	−0.12	0.08	0.14	42	24.02	17.24
Social anxiety disorder (or social phobia)	−0.13	0.46	0.78	15	27.17	16.22
Borderline personality disorder	−0.17	0.21	0.40	12	41.95	38.88
OCD	−0.42	0.36	0.24	25	28.56	25.93
Any eating disorder	−0.67	0.69	0.33	20	28.71	27.60
Specific phobia	−0.99	0.82	0.23	11	33.24	33.70
Other bipolar disorder (e.g. BD-NOS, BD-II)	−1.02	0.96	0.29	20	34.94	28.48

*ADHD* attention-deficit/hyperactivity disorder, *BD* bipolar disorder, *CHR-P* clinical high risk for psychosis, *GAD* generalized anxiety disorder, *NOS* not otherwise specified, *OCD* obsessive-copulsive disorder, *PTSD* post-traumatic stress disorder, *SD* standard deviation, *SE* standard error.

^a^Months.

### Publication biases

Regarding the baseline prevalence of mental disorders, sample size did not moderate statistically significantly most of the outcomes, apart from few for which the magnitude of the coefficient was negligible (Table 1). Similarly, regarding OR meta-analysis, publication bias did not emerge for any of the comparisons versus non-psychotic controls, yet it emerged for any depressive disorder, and any substance use disorder, for which tri-and-fill analyses confirmed significant findings (Supplementary Tables 8 and 9).

## Discussion

To our best knowledge this the first meta-analysis computing both the baseline and follow-up prevalence of comorbid mental disorders in CHR-P individuls, and their clinical impact.

Such meta-analytic summaries (diseases’ prevalence, and associations with outcomes) are routinely used in medicine and psychiatry to quantify the burden to patients and their families as well as clinicians [26], and to inform preventive approaches. Having included 312 studies and up to 765 individuals in the largest study from North America, South America, Europe, Asia, Australia, the results of this work can be considered representative of global clinical setting where preventive services have been implemented [27]. The core finding of this meta-analysis is that over three-quarters of CHR-P subjects present with baseline comorbid mental disorders beyond their CHR-P features. This finding aligns with ancient phenomenological accounts (e.g. Conrad’s mood dysregulation [28]) and more recent epidemiological evidence indicating that psychosis onset can originate from various non-psychotic precurors (i.e. etherotypic phenomenon [29]) and therefore it is essentially transdiagnostic in nature. Although transdiagnostic psychiatry is currently affected by substantial methodological and conceptual biases [30], the corrent results make the case for establishing a baseline transdiagnostic assessment in young people accessing preventive CHR-P services that can capture not only the emergence of attenuated positive psychotic symptoms but also comorbid psychopathological dimensions. From a clinical perspective, the present results might also indicate that psychometric CHR-P tools that are too stringent (i.e. requiring to make a differential diagnosis between a “pure” CHR-P presentation and other comorbid mental disorders that may “better explain” the clinical presentation, e.g. the SIPS or the DSM-5 Attenuated Psychosis Syndrome but not the CAARMS [4]) may exclude many young people, who may be at risk of psychosis, from the much needed preventive care, ultimately further worsening the currenly poor ability to detect them [31, 32]. Indeed, to our best knowledge a new version of CHR-P instruments (i.e. the “PSYCHS”) that allow a broader transdiagnostic inclusion of psychosis-risk is under validation as part of an ongoing international cohort study (AMP initiative). Furthermore, a baseline transdiagnostic assessment in CHR-P individuals allow focusing the recommended preventive treatment (which is Cognitive Behavioral Therapy [33]) on the presenting complaint, therefore improving treatment adherence and service engagement in this vulnerable patient population. Notably, CHR-P individuals tend to seek help largely because of their functonal impairment and comorbid mental disroders such as anxiety and depression, as opposed to the attenuated psychotic symptoms per se [34]. At the same time, this finding indicate that future effective preventive treatments in this patient population are required to target non-psychotic comorbid mental disorders beyond attenuated positive psychotic symptoms.

We also found that the prevalence of comorbid mental disorders tended to decrease over time, yet remaining high, with the exception of any substance use disorders, which increased over follow-up. While the results regarding prevalence of comorbid mental disorders at follow-up are based on a lower number of studies than baseline, calling for cautious interpretation, these findings align with the notion that most comorbid disorders observed at follow-up in CHR-P individuals are actually carried over from baseline. While CHR-P individuals do present transdiagnostic features at presentation, there is no evidence that the CHR-P state is predicting the onset of new/incident non-psychotic mental disorders [35]. For example, to predict the emergence of new/incident biplar disorders, complementary assessment instruments (e.g. the Semistructured Interview of Bipolar At Risk States [36]) would be needed. Although these tools have shown promising psychometric validity, further confirmatory longitudinal studies are ongoing [36–39]. Althoguh we obserbed an increased prevalence of any substance use disorder, a previous longitudinal cohort study found that the CHR-P status has no prognostic validity in forecasting the onset of these disorders [35]. The observed increased prevalence may be partially explained by the fact that cannabis use disorders (which is part of any substance use disorders) is an independent risk factor for developing psychosis [40, 41]. Hence, the increased prevalence of substance use disorders in CHR-P individual may acutally reflect clinical worsening (or a self-medication attempt [42]). Overall, our findings on increased rates of substance use disorders after CHR-P status should be interpreted with caution as they are based on five studies at best across multiple follow-up time points. A related clinical implication of our findings is that future preventive services targeting mental disorders other than psychosis should ideally focus on younger subjects than the CHR-P ones (who had a mean age of 20 years), as in over 75% of them an onset of some non-psychotic mental disorder had already occurred. The largest meta-analysis on age at onset of mental disorders published to date, showed that the proportion of individuals with an onset of any mental disorders before the ages of 14, 18, 25 were 34.6%, 48.4%, 62.5%, respectively, and that the peak age was 14.5 years [43].

We furter found that CHR-P individuals had higher baseline prevalence of any anxiety, panic, anxiety disorders not otherwise specified, schizotypal personality disorder, and alchol use disorders compared to non-psychotic controls. This is the first meta-analytic findings showing that that CHR-P samples accumulate non-psychotic comorbidities, in particular common mental disorders, compared to other help-seeking individuals eventually not meeting the CHR-P criteria. The higher prevalence of schizotypal personality disorders confirms the methodological robustness of our analyses, because this disorder represents a core intake criterion of the CHR-P sample. Similarly, the higher prevalence of alcohol use disorder may reflect the fact that such substance-related comorbidity is explicitely allowed by some CHR-P instruments (e.g. the CAARMS). However, these findings taken together confirm the substantial risk enrichment which is acrrued during the recuritment phase of individuals assessed by CHR-P services, which has been elaborated in full in previous publications [44, 45]. Interestingly, social anxiety disorder, any mood disorder, any depressive disorder, any anxiety disorder were higher in CHR-P individuals compared to psychotic controls. It is possible to interpret this finding in the context of the clnical staging model [46] and speculate that the CHR-P state might represent and earlier stage than the onset of psychosis, which is more predominaltly characterized by mood dysregulation and affective manifestations rather than by frank psychotic features. Finally, the lower prevalence of any substance use disorder compared to psychotic controls aligns with the converging evidence supporting the high prevalence of comorbid substance use disorders in those with schizophrenia spectrum disorders [47].

Notably, we also demonstratred that baseline, alcohol use and schizotypal personalty disorders were associated with lower baseline functioning impairment. This finding is clinically valuable as it indicates that if CHR-P individuals are “probaly at risk” of developing a severe mental disorders, over three-quarters of them are “certainly ill” [48] at presentation and that their highly prevalent comorbid disorders substantially impact their level of functioning. Therefore, this patient population requires support and care at presentation independently from their longitudinal risk of developing psychosis. A recent study has demonstrated, against corrent criticisms, that CHR-P services are best placed than any other mental health service to prioritise the needs of these vulnerable groups, well beyond their attenauted psychotic features [27]. Interestingly, dysthimic and generalized anxiety disorders emerged as positive factors associated with a better baseline functioning. This might be explained by a milder impact on functioning of GAD and dysthimic disorder compared with other comorbid disorders. Finally, we replicated our earlier findings [11] that, despite highly prevalent, comorbid non-psychotic mental disordes do not increase the likelihood of developing psychosis. On the contrary, mood, agoraphobia, and generalized anxiety disorders were associated with lower risk of transition. Previous studies have indicated that anxiety disorders may represent a protective factors. From a broader perspective, this finding tempers the claims that to prevent psychosis it is suficient to target mood and anxiety disorders in the general population [49]; although highly comorbid with psychotic-like features, they may actually be associated with a reduced risk of psychosis onset, at least in CHR-P sampels.

This study has some limitations. First, most findings are characterized by high heterogeneity, which is expected in this patient population [50, 51]. Second, comorbid mental disorders have been grouped differently in the original included studies. We have avoided mixing apples and oranges, and double counting, and have sticked to authors’ disorders categorization. Third, the follow-up results were clustered in large time intervals that many not accurately reflect the granular course of the disorders. Fourth, several of the analyses at follow-up were carachterized by few studies, making findings exploratory rather than conclusive. Fifth, several publications were availabe from the same research team, increasing the chances of overlapping studies. We have minimized this bias by inclucing only one study from each center for each outcome/time-point. Sixth, we could not conduct meta-regressions for all baseline comorbid disorders, since for some of them less than 10 studies were available.

## Conclusions

About three quarters of CHR-P subjects have comorbid mental disorders, which modulate baseline functioning and probability of developing psychosis. A comprehensive transdiagnostic assessment in CHR-P individual is essential to inform preventive care.

### Supplementary information

Supplementary material part 1

Supplementary material part 2

Supplementary material part 3
