# Supplementary material for: Meta-analytic prevalence of comorbid mental disorders in individuals at clinical high risk of psychosis: the case for transdiagnostic assessment
Source: Mol Psychiatry. 2023 Jun 9;28(6):2291–300. doi: 10.1038/s41380-023-02029-8 (PMC10611568; doi:10.1038/s41380-023-02029-8)
Supplement: Supplementary file 1 — Supplementary material part 1 [file 41380_2023_2029_MOESM1_ESM.docx]

**APPENDIX 1 - Supplementary methods and results**

**Meta-analytic prevalence and impact of psychiatric comorbidities in individuals at clinical high risk of psychosis: the case for transdiagnostic assessment**

Marco Solmi^1-6^, Livia Soardo^7^, Simi Kaur^5^, Matilza Azis^5^, Anna Cabras^8^, Marco Censori^9-10^, Luigi Fausti^7^, Filippo Besana^7^, Gonzalo Salazar de Pablo^5-11-13^, Paolo Fusar-Poli^5,7^

1 Department of Psychiatry, University of Ottawa, Ontario, Canada.

2 On Track, First Episode Psychosis Program, Department of Mental Health, The Ottawa Hospital, Ontario, Canada.

3 Ottawa Hospital Research Institute (OHRI) Clinical Epidemiology Program University of Ottawa Ottawa Ontario

4 School of Epidemiology and Public Health, Faculty of Medicine, University of Ottawa, Ottawa, Canada

5 Early Psychosis: Interventions and Clinical-detection (EPIC) Lab, Institute of Psychiatry, Psychology & Neuroscience, Department of Psychosis Studies, King's College London, London, United Kingdom.

6 Department of Child and Adolescent Psychiatry, Charité Universitätsmedizin, Berlin, Germany

7 Department of Brain and Behavioral Sciences, University of Pavia, Pavia, Italy

8 Sapienza University of Rome, Department of Neurology and Psychiatry

9 Department of Neuroscience (DNS), University of Padova, Padua, Italy

10 Dipartimento di Salute Mentale, Azienda ULSS 3 Serenissima, Venezia, Italy

11 Department of Child and Adolescent Psychiatry, Institute of Psychiatry, Psychology & Neuroscience, King’s College London UK

12 Child and Adolescent Mental Health Services, South London and Maudsley NHS Foundation Trust, London, UK

13 Institute of Psychiatry and Mental Health. Department of Child and Adolescent Psychiatry, Hospital General Universitario Gregorio Marañón School of Medicine, Universidad Complutense, Instituto de Investigación Sanitaria Gregorio Marañón (IiSGM), CIBERSAM, Madrid, Spain

**Corresponding author**

Paolo Fusar-Poli

paolo.fusar-poli@kcl.ac.uk

**Protocol available at:**

**[https://osf.io/f2t6w/?view_only=fe08c02e1e9e45b09d101ad5c3c84fee](https://osf.io/f2t6w/?view_only=fe08c02e1e9e45b09d101ad5c3c84fee%0c)**

**eFigure 1.** Heat map of country of origin of studies reporting on mental disorders comorbidities in CHR-P subjects

**
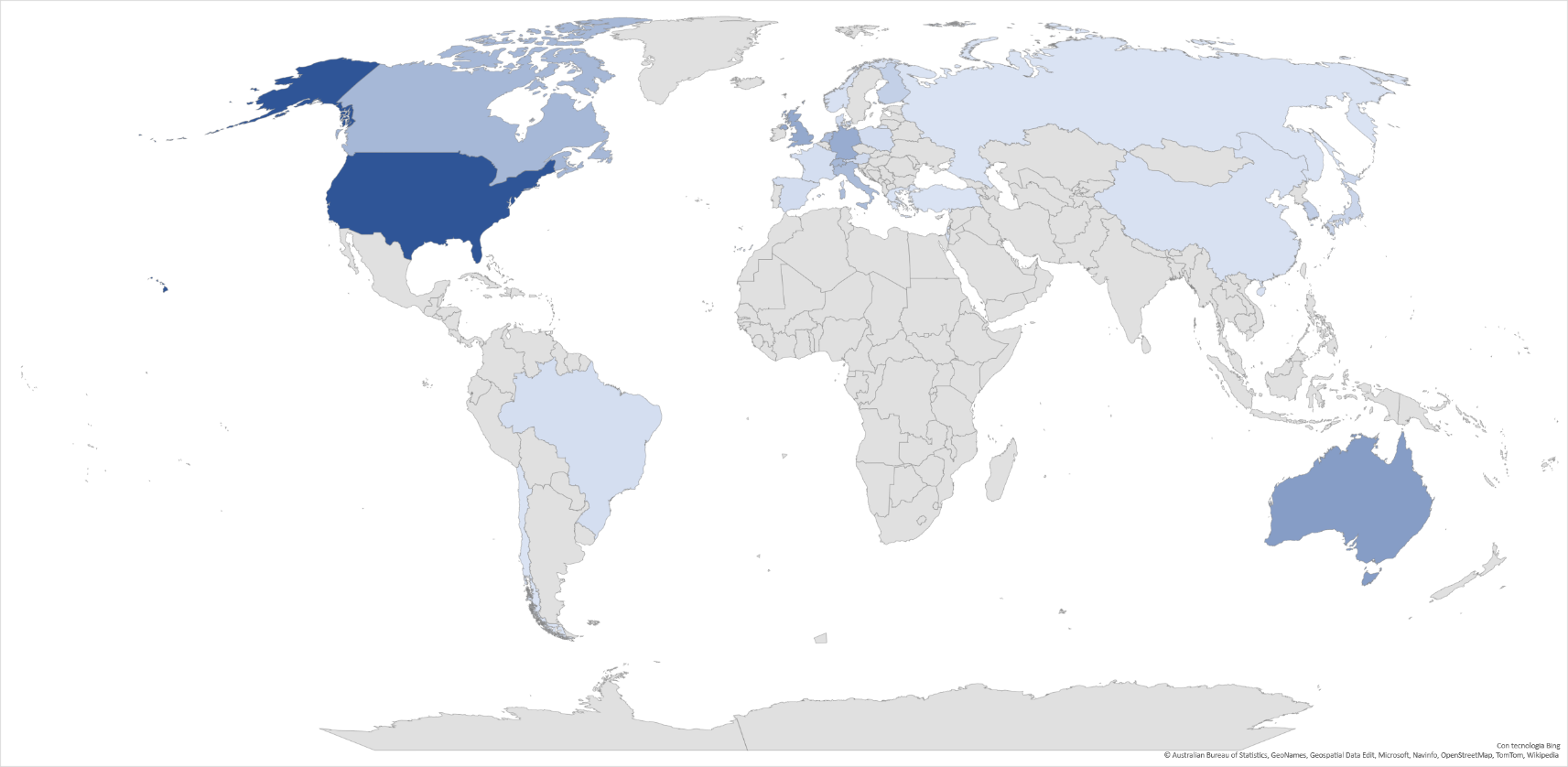
**

**Legend.** *Darker colour indicating more studies. Full details on country where studies were set are reported in eTable 3.*

**eFigure 2. Meta-regression with significant findings between generalized anxiety disorder (A), any mood disorder (B), and agoraphobia (C) and transition to psychosis in CHR-P subjects.**


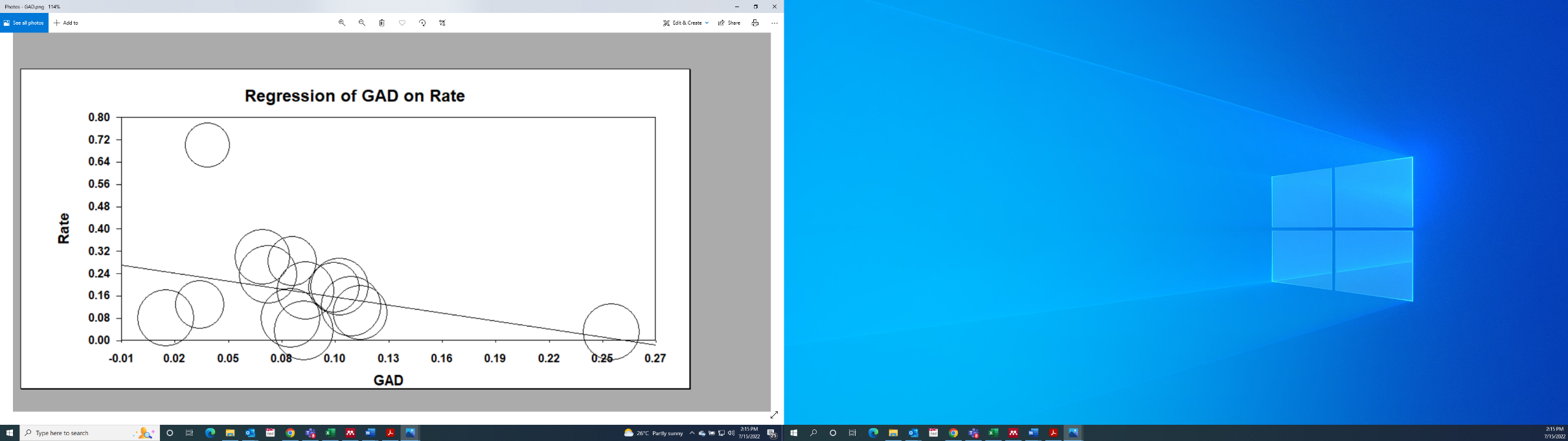
(A)


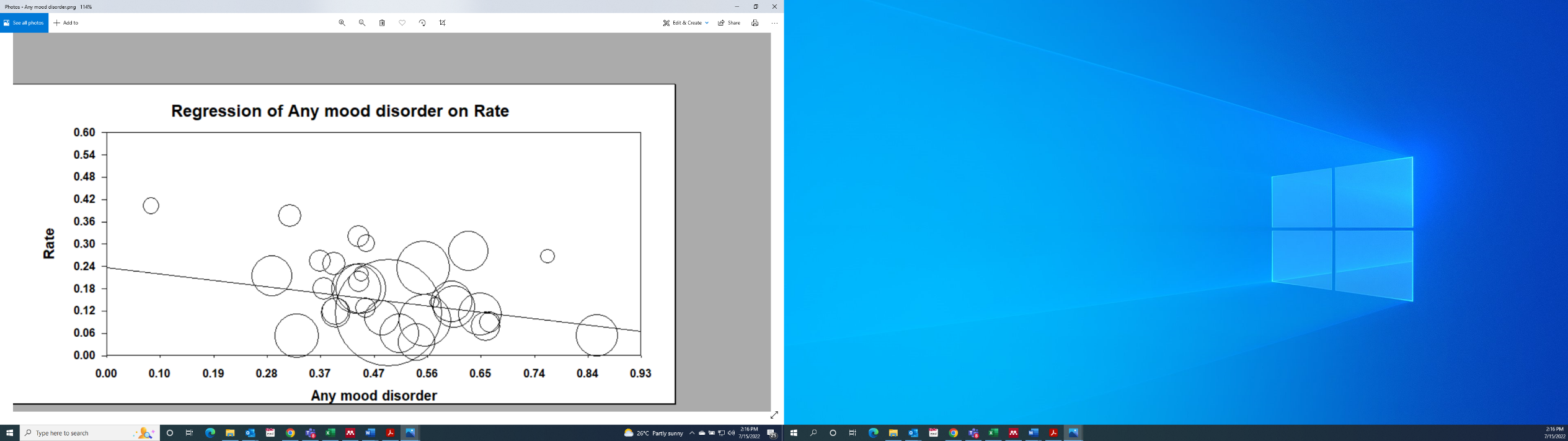


(B)


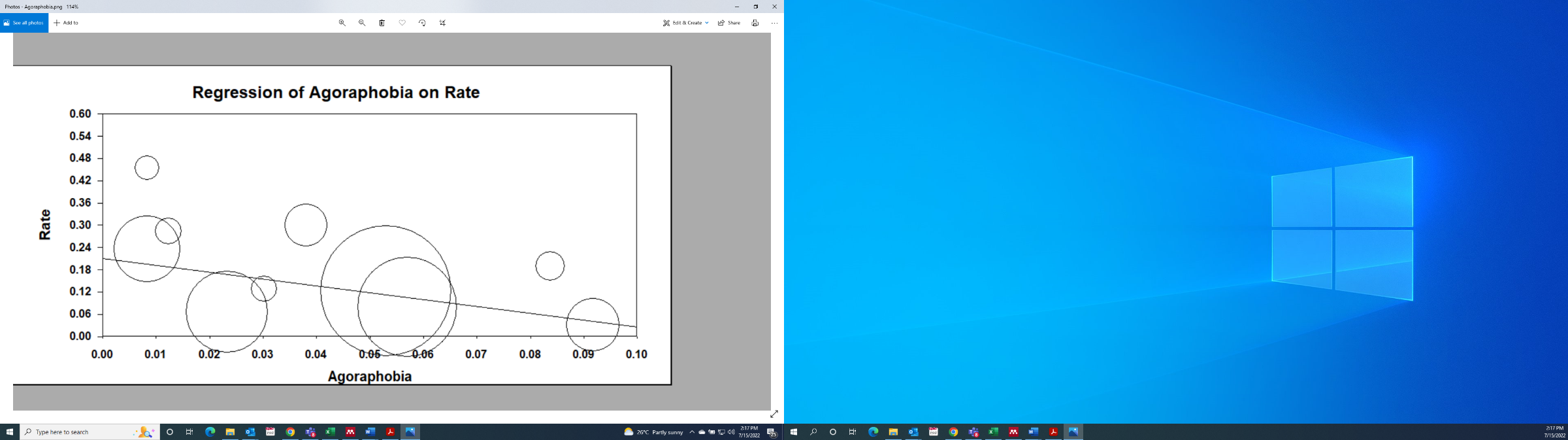


(C)

**eMethods**

*Search strategy and selection criteria*

We conducted a PRISMA 2020-compliant^1^ and MOOSE-compliant^2^ (eTables 1-2) systematic search of PubMed and PsycInfo, up to June 26^th^, 2021, using the search key “*((psychosis risk) or (risk of psychosis) or caarms or sips or sops or uhr or prodrom* or (ultra-high and risk) or (clinical high risk) or aps* or blip* or bips or attenuated or grd or (basic symptoms) or (at risk mental state)) and (psychosis or schizophrenia)”*and following an a-priori [protocol](https://osf.io/f2t6w/?view_only=fe08c02e1e9e45b09d101ad5c3c84fee)*.* The search was supplemented with manual search of the articles identified, and previous reviews. Two authors independently screened the title and abstract of all the retrieved publications, as well as the full text of those that were eligible at title abstract level (LS, SK, MA, AC, MC, LF, FB). Any disagreement was resolved after consensus, and if needed a third author made the final decision (MS, PFP). The reason for exclusion of articles after full text assessment was noted (Appendix 2-3).

We applied the following inclusion criteria: i) observational (cross-sectional or longitudinal) and randomized controlled trials (RCTs) (given previous evidence indicating that the risk of transitioning to psychosis do not significantly differ between cohort studies and the control arm of RCTs in this patient population, therefore suggesging no substantial sampling biases in RCT designs^3^), ii) that reported on subjects meeting CHR-P criteria as per established psychometric instruments (i.e. the CAARMS^4^, SIPS^5^, Early Recognition Inventory based on IRAOS -ERIraos-^6^, Basel Screening Instrument für Psychosen -BSIP-^7^, Bonn Scale for the Assessment of Basic Symptoms -BSABS-^8^, Schizophrenia Proneness Instrument, Adult -SPI-A-^9^, Child and Youth version -SPI-CY-^10^, iii) and reported on the prevalence of any mental disorders established according to DSM-any version^11^, ICD-any version^12^, or validated scales employing cut-offs that map onto DSM or ICD diagnostic categories, iv) and that were published in English language. The type of comorbid mental disorder was classified as reported by authors of eligible studies, and each disorder, spectrum, or combination of disorders/spectra was considered as a separate outcome. (eTable 1, eMethods).

We excluded i) reviews, ii) studies not assessing the CHR-P state with established psychometric instruments, iii) studies not assessing mental disorders with DSM/ICD/validated scales, iv) studies in language other-than-English. Since many different publications frequently reported on the same cohort, and on the same outcomes, in the case of overlapping samples for the same primary/secondary outcomes we have selected the largest cohort.

*Outcomes and data extraction*

The primary outcome was the baseline prevalence of comorbid mental disorders in CHR-P individuals. Secondary outcomes were the prevalence of comorbid mental disorders at follow-up, the association of baseline comorbid mental disorders with CHR-P status, when compared to non-psychotic and psychotic control groups, in studies reporting these data. The non-psychotic control group included those undergoing a CHR-P assessment but eventually not meeting CHR-P criteria, or any other population where mental disorders were reported even without undergoing CHR-P status assessment. The psychotic control group included those diagnosed with a DSM/ICD-any version psychotic disorder, regardless they were or were not previously meeting CHR-P criteria. Secondary outcomes included the metaregression association of baseline comorbid mental disorders with baseline functioning, and with transition to psychosis at follow-up in studies reporting these outcomes.

The same authors that performend the screening extracted the data, with the supervision of a third author who resolved any conflict. From each included study we extracted the following variables: author, year, country, study design, sample size, age, sex, CHR-P criteria, mental disorders diagnostic criteria, prevalence of each mental disorder at any time point, baseline functioning, duration of follow-up, risk of transition to psychosis at follow-up.

*Comorbid disorders definitions*

The type of comorbid mental disorder was classified as reported by authors of eligible studies, and each disorder, spectrum, or combination of disorders/spectra was considered as a separate outcome. (eTable 1). For instance, some authors reported on prevalence of any depressive/anxiety disorder, other on major depressive disorder, others on any depressive episode/disorder. We deliberately did not lump different definitions and adhered to categories as in included articles, and we did not split diagnostic groups encompassing more than one diagnosis as we did not have access to individual-patient data for this project.

*Quality assessment*

The quality of the included studies was assessed with the Newcastle-Ottawa scale (NOS)^13^. We measured the quality of RCTs with NOS as well, as we were here interested in the quality of the baseline and follow-up assessment, and not on the efficacy or safety of a given intervention.

*Statistical analyses*

The primary effect size measure was the baseline prevalence of comorbid mental disorders, defined as as the percentage of individuals with a diagnosis of comorbid mental disorders at baseline in the total CHR-P sample. The secondary outcomes were investigated with the meta-analytic OR of the prevalence of comorbid mental disorders in CHR-P individuals compared to non-psychotic and psychotic control groups. Transition to psychosis was measured at 6-24, 24-48, 48-96, and more than 96 months of follow-up. Other secondary outcomes were investigated with meta-regression analyses testing whether the baseline prevalence of comorbid mental disorders was associated with baseline functioning as well as transition to psychosis at follow-up. For these analyses we reported the average follow-up time across the pooled studies, reported by each individual study. In the meta-regression on functioning, we anticipated that almost studies reported a score on a 0-100 scale, and we converted to the same scale (i.e. 0-100) scores from tools measuring functioning on a different range.

We conducted a random-effects^14^ meta-analysis given the expected heterogeneity, when at least two studies reported on the same outcome at the same time point. Q statistic was used to assess heterogeneity among study point estimates, while the proportion of total variability in prevalence was evaluated with the I^2^ index. Publications bias was assessed with the Egger’s test^15^ for OR meta-analysis in CHR-P vs control groups, and with meta-regression between the main outcome (baseline prevalence of comorbid mental disorders) as dependent variable and sample size as independent variable for prevalence meta-analysis^16^. For OR meta-analyses, in case of publication bias (i.e. Egger’s test p value <0.1), we also recalculated the corrected OR after trim-and-fill procedure. Meta-regressions were performed when at least 10 studies were available. Comprehensive Meta-Analysis software v.3 was used for the analyses. P value was set to <0.05 two sided.

**eTable 1.** PRISMA 2020 checklist.

| **Section and Topic** | **Item #** | **Checklist item** | **Page** |
| --- | --- | --- | --- |
| **TITLE** | | |  |
| Title | 1 | Identify the report as a systematic review. | 3 |
| **ABSTRACT** | | |  |
| Abstract | 2 | See the PRISMA 2020 for Abstracts checklist. | 3 |
| **INTRODUCTION** | | |  |
| Rationale | 3 | Describe the rationale for the review in the context of existing knowledge. | 4 |
| Objectives | 4 | Provide an explicit statement of the objective(s) or question(s) the review addresses. | 4 |
| **METHODS** | | |  |
| Eligibility criteria | 5 | Specify the inclusion and exclusion criteria for the review and how studies were grouped for the syntheses. | 5 |
| Information sources | 6 | Specify all databases, registers, websites, organisations, reference lists and other sources searched or consulted to identify studies. Specify the date when each source was last searched or consulted. | 5 |
| Search strategy | 7 | Present the full search strategies for all databases, registers and websites, including any filters and limits used. | 5 |
| Selection process | 8 | Specify the methods used to decide whether a study met the inclusion criteria of the review, including how many reviewers screened each record and each report retrieved, whether they worked independently, and if applicable, details of automation tools used in the process. | 5-eMethods |
| Data collection process | 9 | Specify the methods used to collect data from reports, including how many reviewers collected data from each report, whether they worked independently, any processes for obtaining or confirming data from study investigators, and if applicable, details of automation tools used in the process. | 6-eMethods |
| Data items | 10a | List and define all outcomes for which data were sought. Specify whether all results that were compatible with each outcome domain in each study were sought (e.g. for all measures, time points, analyses), and if not, the methods used to decide which results to collect. | 5-6 |
|  | 10b | List and define all other variables for which data were sought (e.g. participant and intervention characteristics, funding sources). Describe any assumptions made about any missing or unclear information. | 6-eMethods |
| Study risk of bias assessment | 11 | Specify the methods used to assess risk of bias in the included studies, including details of the tool(s) used, how many reviewers assessed each study and whether they worked independently, and if applicable, details of automation tools used in the process. | 6-eMethods |
| Effect measures | 12 | Specify for each outcome the effect measure(s) (e.g. risk ratio, mean difference) used in the synthesis or presentation of results. | 6-eMethods |
| Synthesis methods | 13a | Describe the processes used to decide which studies were eligible for each synthesis (e.g. tabulating the study intervention characteristics and comparing against the planned groups for each synthesis (item #5)). | 5-eMethods |
|  | 13b | Describe any methods required to prepare the data for presentation or synthesis, such as handling of missing summary statistics, or data conversions. | 5-eMethods |
|  | 13c | Describe any methods used to tabulate or visually display results of individual studies and syntheses. | eMethods |
|  | 13d | Describe any methods used to synthesize results and provide a rationale for the choice(s). If meta-analysis was performed, describe the model(s), method(s) to identify the presence and extent of statistical heterogeneity, and software package(s) used. | 6-eMethods |
|  | 13e | Describe any methods used to explore possible causes of heterogeneity among study results (e.g. subgroup analysis, meta-regression). | 6-eMethods |
|  | 13f | Describe any sensitivity analyses conducted to assess robustness of the synthesized results. | 6-eMethods |
| Reporting bias assessment | 14 | Describe any methods used to assess risk of bias due to missing results in a synthesis (arising from reporting biases). | 6-eMethods |
| Certainty assessment | 15 | Describe any methods used to assess certainty (or confidence) in the body of evidence for an outcome. | eMethods |
| **RESULTS** | | |  |
| Study selection | 16a | Describe the results of the search and selection process, from the number of records identified in the search to the number of studies included in the review, ideally using a flow diagram. | Figure1 |
|  | 16b | Cite studies that might appear to meet the inclusion criteria, but which were excluded, and explain why they were excluded. | Appendix 2-3 |
| Study characteristics | 17 | Cite each included study and present its characteristics. | eTable 3 |
| Risk of bias in studies | 18 | Present assessments of risk of bias for each included study. | eTable1 |
| Results of individual studies | 19 | For all outcomes, present, for each study: (a) summary statistics for each group (where appropriate) and (b) an effect estimate and its precision (e.g. confidence/credible interval), ideally using structured tables or plots. | Table1, Table2, Table 3, eTable4, eTable5, eTable6, eTable7, eTable 8, eTable9 |
| Results of syntheses | 20a | For each synthesis, briefly summarise the characteristics and risk of bias among contributing studies. | 8 |
|  | 20b | Present results of all statistical syntheses conducted. If meta-analysis was done, present for each the summary estimate and its precision (e.g. confidence/credible interval) and measures of statistical heterogeneity. If comparing groups, describe the direction of the effect. | 7-8 |
|  | 20c | Present results of all investigations of possible causes of heterogeneity among study results. | 5-eMethods |
|  | 20d | Present results of all sensitivity analyses conducted to assess the robustness of the synthesized results. | eTables |
| Reporting biases | 21 | Present assessments of risk of bias due to missing results (arising from reporting biases) for each synthesis assessed. | eTables |
| Certainty of evidence | 22 | Present assessments of certainty (or confidence) in the body of evidence for each outcome assessed. | Table1, Table2, Table 3, eTable4, eTable5, eTable6, eTable7, eTable 8, eTable9 |
| **DISCUSSION** | | |  |
| Discussion | 23a | Provide a general interpretation of the results in the context of other evidence. | 9 |
|  | 23b | Discuss any limitations of the evidence included in the review. | eDiscussion |
|  | 23c | Discuss any limitations of the review processes used. | eDiscussion |
|  | 23d | Discuss implications of the results for practice, policy, and future research. | 9-10-11 |
| **OTHER INFORMATION** | | |  |
| Registration and protocol | 24a | Provide registration information for the review, including register name and registration number, or state that the review was not registered. | 3 |
|  | 24b | Indicate where the review protocol can be accessed, or state that a protocol was not prepared. | 3 |
|  | 24c | Describe and explain any amendments to information provided at registration or in the protocol. | eMethods |
| Support | 25 | Describe sources of financial or non-financial support for the review, and the role of the funders or sponsors in the review. | 11-12 |
| Competing interests | 26 | Declare any competing interests of review authors. | 11-12 |
| Availability of data, code and other materials | 27 | Report which of the following are publicly available and where they can be found: template data collection forms; data extracted from included studies; data used for all analyses; analytic code; any other materials used in the review. | 11 |

*From:*  Page MJ, McKenzie JE, Bossuyt PM, Boutron I, Hoffmann TC, Mulrow CD, et al. The PRISMA 2020 statement: an updated guideline for reporting systematic reviews. BMJ 2021;372:n71. doi: 10.1136/bmj.n71

**eTable 2.** MOOSE checklist

| **Item No** | **Recommendation** | **Reported on Page No** |
| --- | --- | --- |
| Reporting of background should include | | |
| 1 | Problem definition | 4 |
| 2 | Hypothesis statement | 4 |
| 3 | Description of study outcome(s) | 5-6 |
| 4 | Type of exposure or intervention used | 5-eMethods |
| 5 | Type of study designs used | 5 |
| 6 | Study population | 5 |
| Reporting of search strategy should include | | |
| 7 | Qualifications of searchers (eg, librarians and investigators) | eMethods |
| 8 | Search strategy, including time period included in the synthesis and key words | 5- eMethods |
| 9 | Effort to include all available studies, including contact with authors | eMethods |
| 10 | Databases and registries searched | 5- eMethods |
| 11 | Search software used, name and version, including special features used (eg, explosion) | 5- eMethods |
| 12 | Use of hand searching (eg, reference lists of obtained articles) | 5- eMethods |
| 13 | List of citations located and those excluded, including justification | Appendix2-3 |
| 14 | Method of addressing articles published in languages other than English | 5- eMethods |
| 15 | Method of handling abstracts and unpublished studies | eMethods |
| 16 | Description of any contact with authors | eMethods |
| Reporting of methods should include | | |
| 17 | Description of relevance or appropriateness of studies assembled for assessing the hypothesis to be tested |  |
| 18 | Rationale for the selection and coding of data (eg, sound clinical principles or convenience) |  |
| 19 | Documentation of how data were classified and coded (eg, multiple raters, blinding and interrater reliability) | 5- eMethods |
| 20 | Assessment of confounding (eg, comparability of cases and controls in studies where appropriate) |  |
| 21 | Assessment of study quality, including blinding of quality assessors, stratification or regression on possible predictors of study results | 6, eMethods |
| 22 | Assessment of heterogeneity | 6-eMethods |
| 23 | Description of statistical methods (eg, complete description of fixed or random effects models, justification of whether the chosen models account for predictors of study results, dose-response models, or cumulative meta-analysis) in sufficient detail to be replicated | 6- eMethods |
| 24 | Provision of appropriate tables and graphics | Table1, Table2, Table 3, eTable4, eTable5, eTable6, eTable7, eTable 8, eTable9, Figure2,eFigure1 |
| Reporting of results should include | | |
| 25 | Graphic summarizing individual study estimates and overall estimate | Table1, Table2, Table 3, eTable4, eTable5, eTable6, eTable7, eTable 8, eTable9 |
| 26 | Table giving descriptive information for each study included | eTable3 |
| 27 | Results of sensitivity testing (eg, subgroup analysis) | eTables |
| 28 | Indication of statistical uncertainty of findings | Tables and eTables |

| **Item No** | **Recommendation** | **Reported on Page No** |
| --- | --- | --- |
| Reporting of discussion should include | | |
| 29 | Quantitative assessment of bias (eg, publication bias) | Tables and eTables |
| 30 | Justification for exclusion (eg, exclusion of non-English language citations) | Appendix 2-3 |
| 31 | Assessment of quality of included studies | eTable3 |
| Reporting of conclusions should include | | |
| 32 | Consideration of alternative explanations for observed results | 11 |
| 33 | Generalization of the conclusions (ie, appropriate for the data presented and within the domain of the literature review) | 11 |
| 34 | Guidelines for future research | 11 |
| 35 | Disclosure of funding source | 11-12 |

*From*: Stroup DF, Berlin JA, Morton SC, et al, for the Meta-analysis Of Observational Studies in Epidemiology (MOOSE) Group. Meta-analysis of Observational Studies in Epidemiology. A Proposal for Reporting. *JAMA*. 2000;283(15):2008-2012. doi: 10.1001/jama.283.15.2008.

**eTable 3.** Characteristics of the included Studies

| **First author, year** | **Country** | **Design** | **CHR-P sample size** | **CHR-P subgroups (%)** | **Age: mean, SD (range)** | **Sex: % female** | **CHR-P, functioning assessment tools** | **Disorders** | **Control group** | **Diagnostic criteria** | **Total duration**  **(Months)** | **NOS** |
| --- | --- | --- | --- | --- | --- | --- | --- | --- | --- | --- | --- | --- |
| Addington 2007^19^ | Multisite | Longitudinal cohort | 370 | 94.3 APS, 3.9 BLIPS, 25.2 GRD | 18.2, 2.7 | 38.1 | SIPS | Any mood disorder, Any anxiety disorder, Alcohol use disorder, Substance use disorder NOS, | Non-psychotic disorders | DSM | NA | 8 |
| Addington 2011a^20^ | Canada | Randomised clinical trial | 51 | 100 APS | 20.9, 4.2 (14-30) | 29.4 | SIPS | Any mood disorder, Any depressive disorder, Any anxiety disorder, Cannabis use disorder, Alcohol use disorder | None | DSM | 18 | 7 |
| Addington 2011b^21^ | USA, Canada | Longitudinal cohort | 111 | 100 APS | 18.0, 4.9 (12-36) | 44.1 | SIPS | Any depressive disorder, Borderline personality disorder, Paranoid personality disorder, Avoidant personality disorder, Obsessive compulsive personality disorder, Any anxiety disorder, Any substance use disorder | None | DSM | 12 | 7 |
| Addington 2012^22^ | USA, Canada | Cross-sectional | 360 | 11.4 APS, 2.3 BLIPS, 14.0 GRD | 19.0, 4.2 (12-34) | 41.4 | CAARMS | Borderline personality disorder, Avoidant personality disorder | None | DSM | NA | 7 |
| Addington 2013^23^ | USA, Canada | Cross-sectional | 360 | NA | 19.0, 4.2 | 44.7 | SIPS | Any neurodevelopmental disorder | None | DSM | NA | 7 |
| Addington 2017^24^ | USA, Canada | Longitudinal cohort | 744 | NA | 18.5, 4.2 (12-35) | 42.7 | SIPS | Any non-psychotic mental disorder, Any mood disorder, Any depressive disorder, Major depressive disorder, Dysthymia, Bipolar disorder-I, Other bipolar disorders, Cyclothymia, Autism spectrum disorder, Aspergers disorder, Learning disorder, ADHD, Developmental disorder, Oppositional deficient disorder, Any anxiety disorder, Panic disorder, Generalised anxiety disorder, Specific phobia, Social anxiety disorder, Agoraphobia, Anxiety disorder NOS, Obsessive compulsive disorder, Cannabis use disorder. Alcohol use disorder, Cocaine use disorder, Amphetamine use disorder, Hallucinogen use disorder, Polysubstance use disorder, Substance use disorder NOS, Post traumatic stress disorder, Any eating disorder, Bulimia, Anorexia nervosa, Eating disorder NOS, Somatoform disorder | None | DSM | 24 | 7 |
| Addington 2019a^25^ | Canada | Longitudinal cohort | 108 | NA | 16.8, 3.3 (12-25) | 56.5 | SIPS | Any depressive disorder, Borderline personality disorder, Autism spectrum disorder, Attention deficit hyperactivity disorder, Panic disorder, Generalised anxiety disorder, Specific phobia, Social anxiety disorder, Agoraphobia, Anxiety disorder NOS, Obsessive compulsive disorder, Cannabis use disorder, Alcohol use disorder, Post-traumatic stress disorder | None | DSM | NA | 7 |
| Addington 2019b^26^ | Multisite | Longitudinal cohort | 278 | NA | 18.8, 4.4 | 44.6 | SIPS | Any behavioural disorder, Any substance use disorder | Non clinical controls | DSM | 24 | 7 |
| Allott 2014^27^ | Austria | Longitudinal cohort | 37 | 91.9 APS, 37.8 BLIPS, 5.4 GRD | 16.2, 1.7 (13-22) | 67.6 | CAARMS | Major depressive disorder, Borderline personality disorder, Any anxiety disorder, Any substance use disorder | None | DSM | 12 | 7 |
| Amminger 2015^28^ | Austria | Randomised clinical trial | 81 | 90.1 APS, 43.2 BLIPS, 7.4 GRD | 16.4, 2.1 (13-25) | 67.0 | CAARMS | Any non-psychotic mental disorder, Any mood disorder, Major depressive disorder, Other bipolar disorder, Any anxiety disorder, Social anxiety disorder, Agoraphobia, Anxiety disorder NOS, Obsessive compulsive disorder, Any substance use disorder, Cannabis use disorder-NOS, Alcohol use disorder-NOS, Any eating disorder, Bulimia, Eating disorder NOS | None | DSM | 80* | 7 |
| An 2010^29^ | South Korea | Longitudinal cohort | 24 | 95.8 APS, 20.8 BLIPS, 12.5 GRD | 20.0, 3.9 (15-35) | 41.7 | SIPS | Trichotillomania | Psychotic disorders | DSM | 5 | 6 |
| Andreou 2014^30^ | Germany | Cross-sectional | 18 | 83.3 APS/BLIP, 5.6 GRD | 24.2, 4.2 | 38.9 | SIPS | Any non-psychotic mental disorder, | Psychotic disorders | DSM | NA | 6 |
| Armando 2015^31^ | Italy | Longitudinal cohort | 35 | 82.9 APS, 14.3 BLIPS, 11.4 GRD | 13.8, 2.1 (9-17) | 48.6 | SIPS | Any behavioural disorder | None | DSM | 12 | 7 |
| Aston 2012^32^ | Switzerland | Longitudinal cohort | 61 | NA | 26.6, 8.5 | 41.0 | BSIPS | Any mood disorder, Any anxiety disorder | None | DSM | NA | 7 |
| Auther 2012^33^ | USA | Longitudinal cohort | 101 | 100 APS | 16.1, 2.2 (12-22) | 34.7 | SIPS | Cannabis use disorder | None | DSM | 36 | 8 |
| Auther 2015^34^ | USA, Canada | Longitudinal cohort | 341 | 96.8 APS, 2.9 BLIPS, 0.3 GRD | 18.3 | NA | SIPS | Cannabis use disorder, Alcohol use disorder, Cocaine use disorder | None | DSM | 24 | 7 |
| Aziz 2019^35^ | USA | Cross-sectional | 214 | 91.0 APS, 0 BLIPS, 16.0 GRD | 22.2, 3.8 (12-31) | 47.0 | SIPS | Any non-psychotic mental disorder, Any mood disorder, Bipolar disorder-I, Schizotypal personality disorder, Any anxiety disorder | None | DSM | NA | 7 |
| Ballon 2008^36^ | USA | Longitudinal cohort | 52 | NA | (12-30) | 44.1 | SIPS | Any depressive disorder, Schizotypal personality disorder, Any anxiety disorder | None | DSM | 24 | 6 |
| Bechdolf 2010^37^ | Australia | Longitudinal cohort | 92 | 80.4 APS, 4.3 BLIPS, 29.3 GRD | 18.0, 2.0 (15-24) | 65.2 | CAARMS | Any depressive disorder, Dysthymia, Any anxiety disorder, Obsessive compulsive disorder, Post traumatic stress disorder, Adjustment disorder | None | DSM | 20.5* | 8 |
| Bechdolf 2011^38^ | Germany | Longitudinal cohort | 156 | 69.2 APS, 6.4 BLIPS, 0 GRD | 23.9, 4.8 | 32.1 | SIPS | Any non-psychotic mental disorder, Major depressive disorder, Dysthymia, Depressive disorder NOS, Any personality disorder, Borderline Personality disorder, Schizotypal personality disorder, Schizoid personality disorder, Paranoid personality disorder, Antisocial personality disorder, Histrionic Personality disorder, Narcissitic personality disorder, Avoidant personality disorder, Dependent personality disorder, Obsessive-compulsive personality disorder, Personality disorder NOS, Panic disorder, Social anxiety disorder, Agoraphobia, Phobias-NOS, Obsessive compulsive disorder, Body dysmorphic disorder, Cannabis use disorder, Alcohol use disorder, Substance use disorder NOS, Post-traumatic stress disorder, Adjustment disorder, Bulimia, Binge-eating disorder, Somatoform disorder, Hypochondriasis | None | DSM | 12 | 6 |
| Bechdolf 2012^39^ | Australia | Longitudinal cohort | 22 | NA | 20.8, 4.3 | 54.6 | CAARMS | Other bipolar disorders | None | DSM | 12 | 6 |
| Beck 2019^40^ | Switzerland | Longitudinal cohort | 74 | NA | NA | NA | SIPS BPRS-E; SPI-A; BSIP | Any non-psychotic mental disorder, Any mood disorder, Any anxiety disorder, Any substance use disorder | None | DSM | 192 | 8 |
| Bentley 2016^41^ | USA | Cross-sectional | 36 | 77.8 APS, 8.3 BLIPS, 13.9 GRD | 15.2, 2.4 (12-22) | 72.0 | SIPS | Any mood disorder, Attention déficit hyperactivity disorder, Any behavioural disorder, Any anxiety disorder, Post-traumatic stress disorder | Non-psychotic disorders | DSM | NA | 6 |
| Berger 2017^42^ | Austria | Randomised clinical trial | 69 | NA | 16.4, 1.8 (13-25) | 68.1 | SIPS | Major depressive disorder, Other bipolar disorders, Any anxiety disorder, Any substance use disorder | None | DSM | 84 | 8 |
| Bernard 2017^43^ | USA | Longitudinal cohort | 26 | NA | 18.7, 1.7 (12-21) | 30.8 | SIPS | Any depressive disorder, Other bipolar disorders | None | DSM | 12 | 8 |
| Bodatsch 2011^44^ | Germany | Longitudinal cohort | 62 | NA | 24.8, 6.0 (18-40) | 33.9 | ERIraos | Any mood disorder, Any anxiety disorder, Any substance use disorder, Somatoform disorders | None | DSM | 32* | 7 |
| Boldrini 2020^45^ | Italy | Longitudinal cohort | 57 | NA | 15.8, 1.9 (12-25) | 54.4 | SIPS | Any non-psychotic mental disorder, Any mood disorder, Any personality disorder, Any anxiety disorder | None | DSM | 14 | 7 |
| Bowie 2012^46^ | USA | Longitudinal cohort | 53 | NA | 16.2, 1.9 (12-22) | 24.5 | SIPS | Any mood disorder, Any anxiety disorder, Any substance use disorder | None | DSM | 6 | 6 |
| Brandizzi 2015^47^ | UK | Longitudinal cohort | 154 | 70.8 APS, 7.1 BLIPS, 0 GRD | 23.4, 4.6 (15-35) | 39.6 | CAARMS | Other disorder NOS, Anxiety/Mood disorders | None | DSM | 132 | 8 |
| Brewer 2005^48^ | Australia | Longitudinal cohort | 98 | 60.2 APS, 28.6 BLIPS, 40.8 GRD | 19.8, 3.8 (14-29) | 47.9 | CAARMS | Any non psychotic mental disorder, Major depressive disorder, Dysthymia, Panic disorders, Generalised anxiety disorder, Social anxiety disorder, Obsessive compulsive disorder, Post traumatic stress disorder, Adjustment disorder | None | DSM | 12 | 5 |
| Bright, 2018^49^ | UK | Cross-sectional | 109 | NA | 20.7, 4.3 (14-35) | 42.2 | CAARMS | Any non-psychotic mental disorder, Any depressive disorder, Panic disorders, Social anxiety disorder | None | DSM | NA | 7 |
| Broome 2005^50^ | UK | Longitudinal cohort | 58 | 84.5 APS, 20.7 BLIPS 13.8 GRD | 24.1, 4.2 (14-35) | 34.5 | CAARMS | Any depressive disorder, Any personality disorder, Any anxiety disorder, Obsessive compulsive disorder, Any substance use disorder | None | DSM | 30 | 6 |
| Brucato 2017^51^ | USA | Longitudinal cohort | 200 | 100 APS, 0 BLIPS, 3.6 GRD | 20.8, 3.9 (13-30) | 27.0 | SIPS | Any non-psychotic mental disorder, Major depressive disorder, Dysthymia, Depressive disorder NOS, Bipolar disorder-I, Mood disorder NOS, Panic disorder, Generalised anxiety disorder, Specific phobia, Social anxiety disorder, Agoraphobia, Anxiety disorder NOS, Obsessive compulsive disorder, Body dysmorphic disorder, Post traumatic stress disorder, Any eating disorder, Bulimia, Anorexia Nervosa, Binge eating disorder, Somatoform disorder | None | DSM | 13* | 8 |
| Brucato 2019^52^ | USA | Longitudinal cohort | 200 | 100 APS, 0 BLIPS, 4.2 GRD | 20.1, 3.9 (13-30) | 28.0 | SIPS | Any depressive disorder, Other bipolar disorders, Schizotypal personality disorder, Any anxiety disorder, Any trauma disorder, Sexual trauma | None | DSM | 24 | 7 |
| Brucato 2021^53^ | USA | Longitudinal cohort | 109 | NA | 20.0, 3.7 | 33.7 | SIPS | Any mood disorder, Any substance use disorder | None | DSM | 8* | 7 |
| Buchy 2014^54^ | USA | Longitudinal cohort | 170 | 98.2 APS, 3.5 GRD | 19.8, 4.5 | 43.5 | SIPS | Cannabis use disorder, Alcohol use disorder | None | DSM | 48 | 7 |
| Buchy 2015^55^ | Multisite | Longitudinal cohort | 735 | NA | 18.5, 4.2 | 42.4 | SIPS | Any substance use disorder | None | DSM | 24 | 7 |
| Carol 2015^56^ | USA | Cross-sectional | 37 | NA | 18.8, 1.7 | 35.1 | SIPS | Any mood disorder, Attention déficit hyperactivity disorder, Any anxiety disorder, Post traumatic stress disorder | None | DSM | NA | 6 |
| Carol 2016^57^ | USA | Cross-sectional | 28 | NA | 18.6, 2.0 | 46.4 | SIPS | Any mood disorder, Attention déficit hyperactivity disorder, Any anxiety disorder, Post traumatic stress disorder | None | DSM | NA | 7 |
| Carol 2017^58^ | USA | Cross-sectional | 46 | NA | 19.0, 1.6 | 50.0 | SIPS | Social anxiety disorders | None | DSM | NA | 7 |
| Carr 2000^59^ | Australia | Longitudinal cohort | 60 | 48.3 APS, 36.7 BLIPS, 15.0 GRD | 17.6 | 38.3 | SIPS | Major depressive disorder, Dysthymia, Depressive disorder NOS, Bipolar disorder I, Cyclothymia, Schizotypal personality disorder, Panic disorder, Social anxiety disorder, Obsessive compulsive disorder, Disassociative disorder | Psychotic disorders | DSM | NA | 7 |
| Carríon 2013^60^ | USA | Longitudinal cohort | 92 | NA | 16.0, 2.2 (12-22) | 37.0 | SIPS | Schizotypal personality disorder | None | DSM | 36* | 8 |
| Carríon 2015^61^ | USA | Longitudinal cohort | 45 | 100 APS | 17.5, 1.7 | 20.0 | SIPS | Any mood disorder, Any anxiety disorder, Any substance use disorder | None | DSM | 21* | 7 |
| Carrión 2016^62^ | USA | Longitudinal cohort | 76 | 100 APS | 16.0, 2.2 (12-22) | 31.6 | SIPS | Schizotypal personality disorder | None | DSM | 60 | 7 |
| Carríon 2017^63^ | USA | Longitudinal cohort | 138 | NA | 16.0, 2.1 (12-22) | 30.4 | SIPS | Any mood disorder, Any anxiety disorder, Any substance use disorder | Psychotic disorders | DSM | 60 | 7 |
| Carrion 2018^64^ | USA | Longitudinal cohort | 205 | NA | 16.5, 3.3 (12-25) | 41.5 | SIPS | Any mood disorder, Any anxiety disorder, Any substance use disorder | Psychotic disorders, Non-psychotic disorders | DSM | 23 | 7 |
| Carrion 2021^65^ | USA, Canada | Longitudinal cohort | 349 | NA | 18.5, 4.2 (12-35) | 49.8 | SIPS | Any mood disorder, Any anxiety disorder | None | DSM | 24 | 7 |
| Chung 2010^66^ | South Korea | Cross-sectional | 38 | 97.4 APS, 2.6 BLIPS | 24.2, 6.4 (18-35) | 18.4 | CAARMS | Major depressive disorder, Dysthymia, Depressive disorder NOS, Any anxiety disorder, Social anxiety disorder, Obsessive compulsive disorder, Post traumatic stress disorder, Adjustment disorder | None | DSM | NA | 7 |
| Clamor 2019^67^ | Germany | Cross-sectional | 22 | 95.5, 0 BLIPS, 22.7 GRD | 33.0, 14.1 (18-65) | 72.7 | SIPS | Any anxiety disorder, Any substance use disorder, Post-traumatic stress disorder, Any eating disorder | None | DSM | NA | 6 |
| Cocchi 2008^68^ | Italy | Longitudinal cohort | 71 | NA | 22.1, 3.5 | 33.8 | ERIraos BPRS | Any substance use disorder | Psychotic disorders | DSM, ICD | 60 | 8 |
| Cocchi 2014^69^ | Italy | Cross-sectional | 106 | NA | 22.1, 3.7 (17-30) | 31.1 | ERIraos | Any substance use disorder | Psychotic disorders | ICD | NA | 8 |
| Comparelli 2016^70^ | Italy | Cross-sectional | 45 | 89.0 APS, 2.0 BLIPS, 20.0 GRD | 21.0, 0.4 (15-25) | 51.1 | SIPS | Any mood disorder, Any personality disorder, Any anxiety disorder, Adjustment disorder, Any eating disorder, Any other non-psychotic mental disorder | Non-psychotic disorders | DSM | NA | 6 |
| Conrad 2014^71^ | Australia | Longitudinal cohort | 191 | 69.1 APS, 16.2 BLIPS, 26.2 GRD | 17.6, 3.0 (12-25) | 42.9 | CAARMS | Any depressive disorder, Any personality disorder, Any anxiety disorder, Any substance use disorder | None | ICD | NA | 7 |
| Conrad 2017^72^ | Australia | Longitudinal cohort | 191 | 69.1 APS, 16.2 BLIPS | 17.5, 3.0 (12-25) | 42.9 | CAARMS | Any non-psychotic mental disorder, Any depressive disorder, Any personality disorders, Any anxiety disorders, Any substance use disorder | Psychotic disorders, Non-psychotic disorders | ICD | 87* | 7 |
| Corcoran 2008^73^ | USA | Cross-sectional | 32 | 100 APS, 0 BLIPS, 25.0 GRD | 18.8, 3.6 (12-25) | 18.8 | SIPS | Cannabis use disorder | None | DSM | NA | 7 |
| Corcoran 2011^74^ | USA | Longitudinal cohort | 191 | 98.2 APS, 1.8 BLIPS, 28.6 GRD | 19.6, 3.6 (12-25) | 23.0 | SIPS | Major depressive disorder | None | DSM | 36 | 7 |
| Cornblatt 2007^75^ | USA | Longitudinal cohort | 48 | NA | 15.9, 2.3 (12-22) | 39.6 | SIPS | Cannabis use disorder, Alcohol use disorder | None | DSM | 31* | 7 |
| Cornblatt 2012^76^ | Multisite | Longitudinal cohort | 100 | NA | 18.1, 3.6 | 36.0 | SIPS | Bipolar disorder-I | None | DSM | 30 | 6 |
| Cornblatt 2015^77^ | USA | Longitudinal cohort | 92 | 100 APS | 16.0, 2.2 (12-22) | 30.8 | SIPS | Any mood disorder, Any anxiety disorder, Any substance use disorder | None | DSM | 72 | 8 |
| Cotter 2017^78^ | Australia | Longitudinal cohort | 246 | 76.8 APS, 12.2 BLIPS, 0 GRD | 18.6, 3.3 (15-30) | 53.3 | CAARMS | Any mood disorder. Any anxiety disorder, Any substance use disorder | None | DSM | 89.2* | 8 |
| Cowan 2019^79^ | USA | Cross-sectional | 73 | NA | 18.7, 1.8 | 40.0 | SIPS | Any mood disorder, Attention deficit hyperactivity disorder, Any anxiety disorder, Obsessive compulsive disorder, Post traumatic stress disorder, Any eating disorder | None | DSM | NA | 6 |
| Damme 2020^80^ | USA | Cross-sectional | 26 | NA | 19.2, 1.9 (12-21) | 100 | SIPS | Major depressive disorder, Dysthymia, Bipolar disorder-I, Panic Disorders, Generalised anxiety disorder, Specific phobia, Agoraphobia, Cannabis use disorder, Alcohol use disorder, Adjustment disorder, Somatoform disorders | None | DSM | NA | 7 |
| Dandash 2014^81^ | Singapore | Cross-sectional | 74 | 78.4 APS, 28.4 BLIPS, 0 GRD | 21.4, 3.6 | 33.0 | CAARMS | Any depressive disorder, Any anxiety disorder | None | DSM | NA | 6 |
| De Bock 2020^82^ | Switzerland | Longitudinal cohort | 54 | NA | 25.5, 7.8 | 31.5 | CAARMS | Somatoform disorders | Psychotic disorders | DSM | 72 | 7 |
| De Vos 2019^83^ | Australia/ Netherlands | Longitudinal cohort | 81 | 82.7 APS, 0 BLIPS, 7.4 GRD | 18.0 | 60.5 | CAARMS | Major depressive disorder, Dysthymia, Borderline personality disorder, Schizotypal personality disorder, Any anxiety disorder, Any substance use disorder, Any eating disorder, | None | DSM | 12 | 7 |
| De Wit 2014^84^ | Netherlands | Longitudinal cohort | 44 | NA | 15.1, 2.2 (12-18) | 16.4 | SIPS | Any non psychotic mental disorder, Any depressive disorder, Other bipolar disorders, Borderline personality disorder, Schizotypal personality disorder, Any Pervasive developmental disorder, Aspergers disorders, Pervasive developmental disorder NOS, Attention déficit hyperactivity disorder | None | DSM | 72 | 6 |
| Dean 2014^85^ | USA | Cross-sectional | 26 | NA | 18.5, 2.0 | 38.5 | SIPS | Schizotypal personality disorder | None | DSM | NA | 7 |
| Dean 2016^86^ | USA | Longitudinal cohort | 38 | NA | 18.9, 1.4 (16-21) | 42.1 | SIPS | Any mood disorder, Any anxiety disorder | None | DSM | 12 | 6 |
| Dolz 2019^87^ | Spain | Longitudinal cohort | 91 | 79.1 APS, 4.4 BLIPS, 48.4 GRD | 15.1, 1.7 (10-17) | 59.3 | SIPS | Any depressive disorder, Conduct disorder, Any anxiety disorder | None | DSM | NA | 8 |
| Dragt 2010^88^ | Netherlands | Cross-sectional | 68 | 94.1 APS, 16.2 BLIPS, 13.1 GRD | 19.0, 3.9 (12-29) | 30.9 | SIPS, EriRAOS; BSABS-P | Cannabis use disorder | None | DSM | NA | 7 |
| Dragt 2012^89^ | Multisite | Longitudinal cohort | 243 | NA | 22.6, 5.2 (9-30) | 42.4 | SIPS, BSABS-P | Cannabis use disorder, Alcohol use disorder, | None | DSM | 18 | 7 |
| Eastvold 2007^90^ | USA | Longitudinal cohort | 40 | NA | 20.8, 3.5 (16-30) | 49.0 | SIPS | Any mood disorder, Other bipolar disorders, Any anxiety disorder, | None | DSM | 12 | 7 |
| Epstein 2014^91^ | USA | Cross-sectional | 21 | 78.0 APS, 19.0 BLIPS, 3.0 GRD | 16.1, 3.3 (10-23) | 14.3 | SIPS | Any mood disorder, Attention deficit hyperactivity disorder, Any anxiety disorder, Cannabis use disorder | Psychotic disorders, Non-psychotic disorders | DSM | NA | 8 |
| Falkenberg 2015^92^ | UK | Longitudinal cohort | 221 | 71.0 APS, 9.2 BLIPS, 0 GRD | 22.6, 4.7 (14-35) | 47.1 | CAARMS | Social anxiety disorders | None | DSM | 54* | 7 |
| Fluckiger 2016^93^ | Switzerland | Longitudinal cohort | 103 | NA | 23.4, 6.0 (11-39) | 43.7 | SIPS | Any non psychotic mntal disorder, Any depressive disorder, Any personality disorders, Somatform disorders | None | ICD | 101 | 7 |
| Fontenelle 2011^94^ | Australia | Longitudinal cohort | 312 | NA | 19.8, 3.3 (15-25) | 55.8 | CAARMS | Obsessive compulsive disorder | None | DSM | 89 | 8 |
| Fontenelle 2012^95^ | Australia | Longitudinal cohort | 58 | NA | 19.1, 3.3 (15-30) | 53.4 | CAARMS | Bipolar disorder-I, Somatoform disorder, Any anxiety disorder, Any substance use disorder, Any eating disorder | None | DSM | 88 | 8 |
| Foss Feig 2019^96^ | USA | Longitudinal cohort | 764 | NA | 18.5 (12-35) | 43.3 | SIPS | Autism spectrum disorder | None | DSM | 30 | 8 |
| Francesconi 2017^97^ | Italy | Longitudinal cohort | 67 | NA | 24.5, 3.4 (17-35) | 42.2 | CAARMS | Any mood disorder, Borderline personality disorder | Non-psychotic disorders | DSM | 36 | 7 |
| Francey 2005^98^ | Australia | Longitudinal cohort | 70 | 70.0 APS, 30.0 BLIPS, 27.1 GRD | 20.2 | 47.1 | CAARMS | Any non psychotic mental disorder, Major depressive disorder, Dysthymia, Adjustment disorder | None | DSM | 12 | 7 |
| Fusar-Poli 2013^99^ | UK | Longitudinal cohort | 290 | 89.0 APS, 17.9 BLIPS, 13.8 GRD | 22.9, 4.6 (14-35) | 43.9 | CAARMS | Any non-psychotic mental disorders, Any depressive disorder, Any personality disorders, Any anxiety disorders, Obsessive compulsive disorder, Any substance use disorder | None | DSM | 24 | 7 |
| Fusar-Poli 2015^100^ | UK | Longitudinal cohort | 258 | 70.2 APS, 8.5 BLIPS, 0 GRD | 22.9, 4.5 | 43.4 | CAARMS | Any mood disorder | None | DSM | 72* | 8 |
| Fusar-Poli 2017^101^ | UK | Longitudinal cohort | 80 | 37.5 APS, 100 BLIPS, 6.3 GRD | 25.1, 5.5 | 41.3 | CAARMS | Cannabis use disorder | None | ICD | 60 | 7 |
| Fusar-Poli 2020^102^ | UK | Longitudinal cohort | 598 | 80.4 APS, 18.1 BLIPS, 1.5 GRD | 22.6, 4.9 (13-36) | 44.7 | CAARMS | Any mood disorders, Any personality disorders, Any anxiety disorders, Obsessive compulsive disorder, Post traumatic stress disorder, Any eating disorder, Somatoform disorders | None | DSM | NA | 8 |
| Fux 2013^10^ | Germany | Cross-sectional | 23 | 79.0 APS | 16.8, 1.6 (8-18) | 43.5 | SIPS, SPI-CY | Any mood disorder, Schizotypal personality disorder, Neurodevelopmental disorder, Substance use disorder | Non-psychotic disorders | ICD | NA | 6 |
| Garner 2005^103^ | Australia | Longitudinal cohort | 94 | 44.5 APS, 13.0 BLIPS, 15.0 GRD | 19.1, 3.8 (14-28) | 38.2 | CAARMS | Bipolar disorder-I | None | DSM | 12 | 8 |
| Gaspar 2019^104^ | Chile | Longitudinal cohort | 27 | 92.6 APS, 0 BLIPS, 7.4 GRD | 17.6, 2.9 (12-28) | 29.7 | SIPS | Mood disorder, Any anxiety disorder, Any personality disorder | None | DSM | 24 | 7 |
| Gerstenberg 2016^105^ | Switzerland | Longitudinal cohort | 175 | 53.7 APS | 20.6, 5.8 (13-35) | 38.3 | SIPS | Major depressive disorder, Dysthymia, Other bipolar disorders, Panic disorder, Generalised anxiety disorder, Social anxiety disorder, Agoraphobia, Obsessive compulsive disorder, Post traumatic stress disorder, Bulimia, Anorexia nervosa | None | DSM | NA | 8 |
| Gill 2014^106^ | USA | Longitudinal cohort | 71 | NA | 19.3, 3.6 (14-30) | 26.0 | SIPS | Autism spectrum disorder, Attention deficit hyperactivity disorder | None | DSM | 24 | 7 |
| Girgis 2021a^107^ | USA | Longitudinal cohort | 14 | NA | 22.4, 2.8 | 35.7 | SIPS | Any anxiety disorder, Cannabis use disorder, Any eating disorder, | None | DSM | 24 | 6 |
| Girgis 2021b^108^ | USA | Cross-sectional | 50 | NA | 20.9, 3.9 | 30.0 | SIPS | Major depressive disorder, Any anxiety disorder, Obsessive compulsive disorder | None | DSM | NA | 7 |
| Gisselgard 2018^109^ | Norway | Cross-sectional | 41 | NA | 16.7, 2.4 (13-65) | 61.0 | SIPS | Any depressive disorder, Dysthymia, Other bipolar disorders, Any anxiety disorder, Obsessive compulsive disorder, Polysubstance use disorder, Adjustment disorder | None | DSM | NA | 4 |
| Glenthøj, 2017^110^ | Germany | Cross-sectional | 84 | 98.8 APS, 2.4 BLIPS, 0 GRD | 24.5, 4.7 (18-40) | 58.3 | CAARMS | Any mood disorder, Any personality disorder, Any anxiety disorder, Anu substance use disorder, Adjustment disorder, Any eating disorder, Somatoform disorder | None | DSM | NA | 7 |
| Glenthøj, 2020a^111^ | Denmark | Randomised clinical trial | 146 | 98.7 APS, 2.0 BLIPS, 21.9 GRD | 23.9, 4.2 (18-40) | 58.2 | CAARMS | Any mood disorder, Any personality disorders, Any anxiety disorders, Any substance use disorder, Adjustment disorder, Any eating disorder, Somatoform disorders | None | DSM | 12 | 6 |
| Glenthøj, 2020b^112^ | Denmark | Randomised clinical trial | 146 | 98.7 APS, 2.0 BLIPS, 21.9 GRD | 23.9, 4.2 (18-40) | 58.2 | CAARMS | Any mood disorder, Any personality disorder, Any anxiety disorder, Any substance use disorder, Adjustment disorder, Any eating disorder, Somatoform disorder | None | DSM | 12 | 6 |
| Graber 2019^113^ | USA | Longitudinal cohort | 43 | 70.0 APS, 30.0 BLIPS, 7.0 GRD | 13.0, 3.0 (5-18) | 48.8 | SIPS | Any non-psychotic mental disorder | Psychotic disorders | DSM | NA | 7 |
| Granö 2014^114^ | Finland | Longitudinal cohort | 34 | NA | (12-21) | NA | SIPS | Any depressive disorder, Any anxiety disorder | None | DSM | 11 | 7 |
| Grent-'t-Jong 2020^115^ | UK | Longitudinal cohort | 119 | 73.1 APS, 0 BLIPS, 1.7 GRD | 22.0, 4.4 | 73.1 | CAARMS | Any depressive disorder, Any anxiety disorder, Any substance use disorder, Any eating disorder | None | DSM | 36 | 7 |
| Grent‘t-Jong 2021^116^ | UK | Longitudinal cohort | 116 | NA | 22.0, 4.5 | 70.7 | CAARMS | Any mood disorder | Psychotic disorders, Non-psychotic disorders | DSM | 36 | 8 |
| Grivel 2018^117^ | USA | Longitudinal cohort | 200 | 100 APS | 20.1, 3.9 (13-30) | 28.0 | SIPS | Post traumatic stress disorder | None | DSM | 24 | 7 |
| Gupta 2021^118^ | USA | Longitudinal cohort | 244 | NA | 20.3, 3.7 (12-31) | 51.2 | SIPS | Any depressive disorder, Any anxiety disorder, Cannabis use disorder, Alcohol use disorder | None | DSM | NA | 7 |
| Haining 2020^119^ | UK | Longitudinal cohort | 108 | NA | 21.9 APS, 4.3 (16-35) | 75.9 | CAARMS | Alcohol use disorder | Non-psychotic disorders | DSM | NA | 8 |
| Haroun 2006^120^ | USA | Longitudinal cohort | 50 | NA | 18.7, 4.4 (12-30) | 42.0 | SIPS | Any mood disorder, Any substance use disorder, Any non psychotic mental disorder, Major depressive disorder, Other bipolar disorders, Attention deficit hyperactivity disorder, Conduct disorder, Any anxiety disorders | None | DSM | NA | 8 |
| Hartmann 2016^121^ | Australia | Longitudinal cohort | 397 | 79.3 APS, 14.1 BLIPS, 29.0 GRD | 18.8, 3.4 (15-30) | 53.1 | CAARMS | Any non psychotic mental disorder, Any depressive disorder, Any anxiety disorder, Any substance use disorder | None | DSM | 87* | 7 |
| Hengartner 2017^122^ | Switzerland | Longitudinal cohort | 188 | 53.2 APS, 3.2 BLIPS | 20.5, 5.8 (13-35) | 39.8 | SIPS, SPI-A/CY | Any depressive disorder, Schizotypal personality disorder, Any anxiety disorder, Any substance use disorder | None | DSM | 36 | 7 |
| Hirt 2019b^123^ | Germany | Longitudinal cohort | 33 | 66.7 APS, 24.2 BLIPS, 15.2 GRD | 22.6, 4.1 | 24.2 | BSIP | Any mood disorder, Any anxiety disorder, Any substance use disorder | None | DSM | 6 | 6 |
| Hirt 2019a^124^ | Germany | Longitudinal cohort | 29 | 69.0 APS, 24.1 BLIPS, 6.9 GRD | 22.5, 4.2 | 20.7 | BSIP | Any mood disorder, Any anxiety disorder, Any substance use disorder | None | ICD | 3 | 8 |
| Hubl 2018^125^ | Switzerland | Cross-sectional | 29 | 69.0 APS, 3.0 BLIPS | 19.3, 4.8 | 59.0 | SIPS | Alcohol use disorder, | Non-psychotic disorders | DSM | NA | 6 |
| Hui 2013^126^ | UK | Longitudinal cohort | 60 | 100 APS, 11.7 GRD | 20.2, 2.9 | 48.3 |  | Major depressive disorder, Other bipolar disorders, Panic disorders, Generalised anxiety disorder, Social anxiety disorder, obsessive compulsive disorder, Post traumatic stress disorder | None | DSM | 12 | 7 |
| Ilonen 2010^127^ | Finland | Cross-sectional | 22 | NA | 15.7, 1.8 (13-21) | 90.9 | SIPS | Any mood disorder, Any anxiety disorder | None | DSM, ICD | NA | 6 |
| Ising 2016^128^ | Netherlands | Longitudinal cohort | 185 | 80.5 APS, 19.5 GRD | 22.7, 5.5 | 51.4 | CAARMS | Major depressive disorder, Any personality disorder, Autism spectrum disorder, Attention deficit hyperactivity disorder, Oppositional deficient disorder, Any anxiety disorder, Any substance use disorder, Post traumatic stress disorder, Any eating disorder | None | DSM | 18 | 7 |
| Janssen 2021^129^ | Netherlands | Randomised clinical trial | 61 | NA | 15.8, 3.2 (12-25) | 55.7 | CAARMS | Any depressive disorder, Any personality disorder, Autism spectrum disorder, Attention deficit hyperactivity disorder, Any anxiety disorder, obsessive compulsive disorder | Non-psychotic disorders | DSM | 6 | 6 |
| Jeffries 2018^130^ | USA, Canada | Longitudinal cohort | 72 | NA | 19.4, 4.2 (12-35) | 34.7 | SIPS | Any depressive disorder, Any anxiety disorder | None | DSM | 24 | 8 |
| Jhung 2016^131^ | South Korea | Cross-sectional | 24 | 100 APS, 25.0 BLIPS, 4.2 GRD | 19.9, 3.6 | 45.8 | SIPS | Major depressive disorder, Other bipolar disorder, Schizotypal personality disorder, Panic disorders, Specific phobia, Obsessive compulsive disorder, Any substance use disorder, Any eating disorder | None | DSM | NA | 6 |
| Jung 2011^132^ | South Korea | Cross-sectional | 29 | 96.6 APS, 3.5 BLIPS, 0 GRD | 22.2, 4.3 | 48.3 | CAARMS | Major depressive disorder, Dysthymia, Anxiety disorder NOS | None | DSM | NA | 6 |
| Kang 2018^133^ | South Korea | Cross-sectional | 65 | 98.5 APS, 12.3 BLIPS, 16.9 GRD | 20.1, 3.4 (15-34) | 40.0 | SIPS | Any non-psychotic mental disorder, Any depressive disorder, Schizotypal personality disorder, Any anxiety disorder, Somatoform disorders, Disassociative disorder | None | DSM | NA | 6 |
| Karlsgodt 2009^134^ | USA | Longitudinal cohort | 36 | 78.0 APS, 19.0 BLIPS, 3.0 GRD | 17.0, 3.5 | 25.0 | SIPS | Schizotypal personality disorder | None | DSM | 16 | 7 |
| Karlsgodt 2014^135^ | USA | Longitudinal cohort | 20 | 75.0 APS, 20.0 BLIPS, 5.0 GRD | 16.8, 2.1 | 15.0 | SIPS | Any pervasive developmental disorder, attention deficit hyperactivity disorder | None | DSM | 24 | 7 |
| Kennedy 2021^136^ | USA | Longitudinal cohort | 156 | NA | 19.8, 4.2 | 37.0 | SIPS | Obsessive compulsive disorder | None | DSM | 24 | 8 |
| Kim 2011^137^ | South Korea | Longitudinal cohort | 27 | 92.6 APS, 14.8 BLIPS, 7.4 GRD | 19.9, 3.9 | 48.2 | SIPS | Any depressive disorder, Schizotypal personality disorder, Social anxiety disorder, Any eating disorder | None | DSM | 5 | 8 |
| Kim 2012^138^ | South Korea | Longitudinal cohort | 78 | 91.0 APS, 1.3 BLIPS, 16.7 GRD | 21.3, 4.2 | 34.3 | CAARMS | Any non-psychotic mental disorder, Any anxiety disorders | None | DSM | 60 | 7 |
| Kim 2013^139^ | South Korea | Longitudinal cohort | 60 | 86.7 APS, 18.3 BLIPS, 6.7 GRD | 19.7, 3.3 (15-35) | 41.7 | SIPS | Borderline personality disorder, Schizoid personality disorder, Avoidant personality disorder, Impulse control disorder, Cannabis use disorder | None | DSM | 36 | 7 |
| Kindler 2016^140^ | Switzerland | Cross-sectional | 11 | 54.5 APS | 13.8, 2.5 (8-18) | 100 | SIPS | Attention deficit hyperactivity disorder, Specific phobia | Psychotic disorders, Non-psychotic disorders | DSM | NA | 7 |
| Kindler 2018^141^ | Switzerland | Cross-sectional | 29 | 69.0 APS, 3.4 BLIPS | 19.3, 4.8 (18-65) | 59.0 | SIPS | Attention deficit hyperactivity disorder, Alcohol use disorder | None | DSM | NA | 7 |
| Klauser 2015^142^ | Singapore | Longitudinal cohort | 69 | 81.0 APS, 7.0 BLIPS, 30.0 GRD | 21.5, 3.5 (14-29) | 32.0 | CAARMS | Any mood disorder | None | DSM | 28 | 6 |
| Kline 2018^143^ | Multisite | Cross-sectional | 744 | NA | 18.5 (12-35) | 43.0 | SIPS | Other bipolar disorders | None | DSM | NA | 6 |
| Klosterkotter, 2001^144^ | Germany | Longitudinal cohort | 110 | NA | 28.8, 9.8 | 46.6 | BSABS | Major depressive disorder, Dysthymia, Borderline personality disorder, Schizotypal personality disorder, Schizoid personality disorder, Histrionic personality disorder, Narcissitic personality disorder, Dependent personality disorder, Obsessive compulsive personality disorder, Panic disorder, Generalised anxiety disorder, Obsessive compulsive disorder, Somatoform disorder, Hypochondriasis | Non-psychotic disorders | DSM | 115* | 8 |
| Kollias 2018^145^ | Greece | Longitudinal cohort | 26 | 76.9 APS, 11.5 BLIPS | 25.3, 4.3 (17-42) | 46.2 | CAARMS | Any personality disorders, Panic disorder, Obsessive compulsive disorder, Any substance use disorder | Psychotic disorders | DSM | 36 | 8 |
| Koren 2019^146^ | Israel | Longitudinal cohort | 22 | 100 APS | 15.9, 1.4 | 40.9 | SIPS | Attention deficit hyperactivity disorder, Obsessive compulsive disorder | None | DSM | NA | 7 |
| Korkelia 2013^147^ | Finland, Netherlands | Longitudinal cohort | 244 | 84.0 APS, 0.8 BLIPS, 16.4 GRD | 22.6, 5.1 (16-36) | 44.0 | SIPS | Any depressive disorder | None | DSM | 48 | 8 |
| Kotlicka-Antczak 2014^148^ | Poland | Cross-sectional | 66 | NA | 18.6, 3.3 (15-29) | 53.0 | CAARMS | Any depressive disorder, Schizotypal personality disorder | None | ICD | NA | 8 |
| Kotlicka-Antczak 2017^149^ | Poland | Longitudinal cohort | 81 | 76.5 APS, 4.9 BLIPS, 0 GRD | 18.7, 3.5 (15-32) | 51.9 | CAARMS | Other bipolar disorders | None | DSM | 24 | 7 |
| Kotlicka-Antczak 2018^150^ | Poland | Longitudinal cohort | 99 | 76.8 APS, 4.0 BLIPS, 37.4 GRD | 19.0, 3.6 (15-32) | 54.6 | CAARMS | Other bipolar disorders, Any substance use disorder, Cannabis use disorder, Alcohol use disorder, Opioid use disorder, Polysubstance use disorder, Stimulants use disorder, | None | DSM | 36 | 7 |
| Kotlicka-Antczak 2019^151^ | Poland | Longitudinal cohort | 105 | 77.1 APS, 3.8 BLIPS, 37.1 GRD | 18.8, 3.5 (15-32) | 53.3 | CAARMS | Any depressive disorder, Bipolar disorder-I, Any personality disorder, Borderline Personality disorder, Schizotypal personality disorder, Conduct disorder, Any anxiety disorder, Body dysmorphic disorder, Somatoform disorders | None | DSM | 35.4* | 7 |
| Koutsouleris 2010^152^ | Germany | Longitudinal cohort | 25 | NA | 23.2, 4.7 | 28.0 | CAARMS | Major depressive disorder | None | DSM | 44.4* | 6 |
| Koutsouleris 2012a^153^ | Germany | Longitudinal cohort | 48 | NA | 24.7, 5.8 | 33.3 | SIPS | Any non-psychotic mental disorder, Major depressive disorder, Other bipolar disorders, Adjustment disorder, Conversion disorder | None | DSM | 48 | 6 |
| Koutsouleris 2012b^154^ | Germany | Longitudinal cohort | 40 | NA | 24.5, 5.9 | 32.5 | CAARMS | Major depressive disorder | None | DSM | 44 | 7 |
| Koutsouleris 2021^155^ | Multisite | Longitudinal cohort | 167 | NA | 23.9, 5.4 | 49.7 | SIPS | Major depressive disorder | None | DSM | 23 | 7 |
| Kraan 2018^156^ | Multisite | Longitudinal cohort | 259 | 85.7 APS, 5.8 BLIPS, 15.6 GRD | 22.7, 4.5 (15-35) | 46.3 | CAARMS | Any depressive disorder, Panic disorders, Social anxiety disorder, Obsessive compulsive disorder, Post traumatic stress disorder | None | DSM | 24 | 7 |
| Kristensen 2019^157^ | Denmark | Cross-sectional | 116 | 98.2 APS, 2.6 BLIPS, 23.7 GRD | 23.8, 4.2 (18-40) | 52.6 | CAARMS | Anxiety disorder NOS | None | DSM | NA | 7 |
| Kwak 2019^158^ | South Korea | Longitudinal cohort | 74 | NA | 20.6, 3.8 | 27.0 | SIPS | Any depressive disorder, Major depressive disorder, Dysthymia, Depressive disorder NOS, Panic disorder, Generalised anxiety disorder, Specific phobia, Social anxiety disorder, Anxiety disorder NOS, Obsessive compulsive disorder, Alcohol use disorder, Any eating disorder, Bulimia, Binge eating disorder, Delusional disorder | None | DSM | 72 | 7 |
| Lam 2006^159^ | Hong Kong | Longitudinal cohort | 62 | 82.3 APS, 19.4 BLIPS, 19.4 GRD | 16.2, 3.7 (9-25) | 41.9 | CAARMS | Any depressive disorder, Other bipolar disorders, Generalised anxiety disorder, Post traumatic stress disorder, Anorexia nervosa | None | DSM | 6 | 7 |
| Lam 2015^160^ | Singapore | Longitudinal cohort | 173 | NA | 21.3, 3.5 | 67.6 | CAARMS | Any mood disorder, Any anxiety disorder, Other disorders NOS | None | DSM | 12 | 7 |
| Leanza 2020^161^ | Switzerland | Longitudinal cohort | 200 | 67.5 APS, 7.5 BLIPS, 24.0 GRD | 25.1, 6.9 | 31.0 | BSIP | Any mood disorder, Any anxiety disorder | None | DSM | 60 | 8 |
| Lee 2013^162^ | Singapore | Longitudinal cohort | 173 | 82.2 APS, 3.5 BLIPS, 28.3 GRD | 21.3, 3.5 (14-29) | 32.4 | CAARMS | Major depressive disorder, Dysthymia, Other bipolar disorders, Panic disorder, Generalised anxiety disorder, Social anxiety disorder, Obsessive compulsive disorder, Any substance use disorder, Alcohol use disorder, Post traumatic disorder, Adjustment disorder | Non-psychotic disorders | DSM | 24 | 7 |
| Lee 2017^163^ | South Korea | Cross-sectional | 63 | 90.5 APS, 15.9 BLIPS, 12.7 GRD | 19.7, 3.5 (15-35) | 39.7 | SIPS | Any mood disorder, Social anxiety disorders, Any eating disorder, Other disorders NOS | None | DSM | NA | 7 |
| Leicht 2016^164^ | Germany | Cross-sectional | 24 | NA | 21.5, 3.6 | 45.8 | SPI | Any depressive disorder, Dysthymia, Social anxiety disorder, Agoraphobia, Obsessive compulsive disorder, Cannabis use disorder, Alcohol use disorder, Opioid use disorder | None | ICD | NA | 7 |
| Lencz 2004^165^ | USA | Cross-sectional | 62 | NA | 16.4, 2.3 | 29.0 | SIPS | Any depressive disorder, Any personality disorder, Schizoid personality disorder, Attention deficit hyperactivity disorder, Oppositional defiant disorder, Social anxiety disorder, Obsessive compulsive disorder | Psychotic disorders | DSM | NA | 7 |
| Li 2018^166^ | China | Longitudinal cohort | 100 | 98.0 APS, 3.0 BLIPS, 9.0 GRD | 21.0, 5.4 (15-37) | 57.0 | SIPS | Schizotypal personality disorder, Any non psychotic mental disorder, Any mood disorder, Any anxiety disorder, Obsessive compulsive disorder, Adjustment disorder | None | DSM | 24 | 6 |
| Lim 2015^167^ | Singapore | Longitudinal cohort | 163 | 68.1 APS, 2.5 BLIPS, 14.7 GRD | 21.3, 3.5 (14-29) | 31.9 | CAARMS | Any anxiety disorders, Specific disorder, Agoraphobia, Trichotillomania, Anorexia nervosa, Somatoform disorder | None | DSM | 12 | 7 |
| Lim 2018^168^ | South Korea | Longitudinal cohort | 129 | 92.2 APS, 0.8 BLIPS, 19.4 GRD | 20.3, 3.8 | 33.3 | SIPS | Any mood disorder, Any personality disorders, Schizotypal personality disorder. Schizoid personality disorder, Paranoid personality disorder, Antisocial personality disorder, Histrionic personality disorder, Avoidant personality disorder, Obsessive compulsive personality disorder, Depressive personality disorder, Impulse control disorder, Any anxiety disorder, Adjustment disorder, Somatoform disorder | None | DSM | 24 | 8 |
| Lin 2015^169^ | Australia | Longitudinal cohort | 226 | 77.9 APS, 10.2 BLIPS, 0 GRD | 18.6, 3.3 (15-30) | 55.3 | CAARMS | Any non-psychotic mental disorder, Any mood disorder, Any depressive disorder, Bipolar disorder-I, Other bipolar disorders, Panic disorder, Generalised anxiety disorder, Social anxiety disorder, Phobias-NOS, Obsessive compulsive disorder, Cannabis use disorder-NOS, Alcohol use disorder NOS, Post traumatic stress disorder, Any eating disorder, Somatoform disorders | None | DSM | 82.8* | 7 |
| Lincoln 2014^170^ | USA | Randomised clinical trial | 22 | NA | 22.1, 4.5 (15-35) | NA | SIPS | Social anxiety disorder, Post traumatic stress disorder, Any eating disorder | None | DSM | NA | 4 |
| Lincoln 2018^171^ | Germany | Cross-sectional | 25 | 92.0 APS, 0 BLIPS, 8.0 GRD | 34.7, 14.1 (18-65) | 72.0 | SIPS | Any mood disorder, Any anxiety disorder, Any substance use disorder, Post traumatic stress disorder, Bulimia | Non-psychotic disorders | DSM | NA | 6 |
| Lindgren 2014^172^ | Finland | Longitudinal cohort | 54 | NA | 16.7, 0.9 (15-18) | 81.5 | SIPS | Any mood disorder, Any Neurodevelopmental disorder, Any anxiety disorder, Any substance use disorder, Any eating disorder | Non-psychotic disorders | DSM | 67* | 7 |
| Lindgren 2017^173^ | Finland | Longitudinal cohort | 54 | NA | 16.7, 0.9 (15-18 | 81.5 | SIPS | Any mood disorder, Any neurodevelopmental disorder, Any anxiety disorder, Any substance use disorder, Any eating disorder | Non-psychotic disorders | DSM | 67* | 7 |
| Lo Cascio 2016^174^ | Italy, USA | Cross-sectional | 22 | NA | 14.7, 1.4 | 63.6 | SIPS | Major depressive disorder, Depressive disorder NOS, Other bipolar disorders, Autism spectrum disorder, Attention deficit hyperactivity disorder, Conduct disorder, Oppositional deficient disorder, Any anxiety disorder | Psychotic disorders, Non-psychotic disorders | DSM | NA | 7 |
| Lo Cascio 2017^175^ | Italy | Cross-sectional | 39 | 100 APS | 15.3, 1.8 (12-21) | 48.7 | SIPS | Other bipolar disorders, Any personality disorder, Any neurodevelopmental disorder, Adjustment disorder | Non-psychotic disorders | DSM | NA | 6 |
| Loewy 2019^176^ | USA | Cross-sectional | 103 | 98.0 APS, 1.0 BLIPS, 8.7 GRD | 18.0, 2.2 (12-30) | 52.0 | SIPS | Any trauma disorder, Post-traumatic stress disorder | None | DSM | NA | 7 |
| Machielsen 2010^177^ | Netherlands | Cross-sectional | 169 | NA | 22.3, 4.4 (12-44) | 18.9 | SIPS, PANSS | Cannabis use disorder | Psychotic disorders | DSM | NA | 7 |
| Madsen 2018^178^ | Denmark | Cross-sectional | 42 | 92.9 APS, 7.1 BLIPS, 0 GRD | 23.8, 4.7 (18-40) | 57.0 | CAARMS | Any non-psychotic mental disorder, Major depressive disorder, Dysthymia, Other bipolar disorders, Borderline personality disorder, Schizotypal personality disorder, Any anxiety disorder, Panic disorder, Generalised anxiety disorder, Social anxiety disorder, Anxiety disorder NOS, Obsessive compulsive disorder, Body dysmorphic disorder, Any substance use disorder, Cannabis use disorder, Alcohol use disorder, Substance use disorder NOS, Post-traumatic stress disorder, Any Eating disorder, Hypochondriasis, Disassociative disorders | None | DSM | NA | 7 |
| Manninen 2014^179^ | Finland | Longitudinal cohort | 7 | 100 APS, 0 BLIPS, 0 GRD | (15-18) | 28.6 | SIPS | Any mood disorder, Conduct disorder, Any substance use disorder | Non-psychotic disorders | ICD | 60 | 6 |
| Marshall 2012^180^ | Canada | Randomised clinical trial | 48 | 100 APS | 21.1, 4.2 (14-30) | 68.8 | SIPS | Any mood disorder, Any anxiety disorder, Cannabis use disorder, Alcohol use disorder | None | DSM | 18 | 6 |
| Mason 2004^181^ | Australia | Longitudinal cohort | 74 | 58.1 APS, 31.1 BLIPS, 25.7 GRD | 17.3, 2.8 (13-28) | 47.3 | CAARMS | Any depressive disorder, Other bipolar disorders | None | DSM | 26* | 8 |
| Matsumoto 2019^182^ | Japan | Longitudinal cohort | 309 | 95.1 APS, 11.0 BLIPS, 20.4 GRD | 21.4, 5.5 (14-40) | 61.5 | SIPS | Any non psychotic mental disorder, Any depressive disorder, Other bipolar disorders, Any Neurodevelopmental disorder, Any anxiety disorder, Adjustment disorder, Any eating disorder, Somatoform disorders, Disassociative disorder | None | DSM | 60 | 8 |
| Mazzoni 2009^183^ | USA | Cross-sectional | 9 | NA | 14.7 (13-17) | 22.2 | SIPS | Conduct disorder, Oppositional defiant disorder, Separation anxiety disorder | None | DSM | NA | 6 |
| McAusland 2017^184^ | Multisite | Longitudinal cohort | 765 | NA | 18.5, 4.2 (4-24) | 43.0 | SIPS | Any anxiety disorder, Panic disorder, Generalised anxiety disorder, Specific phobia. Social anxiety disorder, Agoraphobia, Obsessive compulsive disorder, Post traumatic stress disorder | None | DSM | 24 | 8 |
| McDonald 2019^185^ | UK | Cross-sectional | 101 | NA | 22.0, 4.0 (16-35) | 76.2 | CAARMS | Autism spectrum disorder, Attention deficit hyperactivity disorder, Post traumatic stress disorder | None | DSM | NA | 6 |
| McFarlane 2012^186^ | USA | Longitudinal cohort | 250 | NA | 16.7, 3.3 (12-25) | 40.0 | SIPS | Any mood disorder, Any depressive disorder, Other bipolar disorders, Any anxiety disorder, Generalised anxiety disorder, Obsessive compulsive disorder, Any substance use disorder, Post traumatic stress disorder, Other disorders NOS | Non-psychotic disorders | DSM | 24 | 7 |
| McFarlane 2015^187^ | USA | Longitudinal cohort | 87 | NA | 16.3, 3.2 (12-25) | 30.0 | SIPS | Major depressive disorder, Bipolar disorder-I | None | DSM | 24 | 7 |
| Meneghelli 2010^188^ | Italy | Longitudinal cohort | 81 | NA | 22.3, 3.6 (17-30) | 29.6 | ERIraos, BPRS | Any substance use disorder | Psychotic disorders | DSM, ICD | 12 | 7 |
| Menghini-Muller 2019^189^ | Multisite | Longitudinal cohort | 336 | NA | 22.4, 4.9 (18-35) | 47.3 | CAARMS | Any depressive disorder, Dysthymia, Any anxiety disorder, Panic disorder, Generalised anxiety disorder, Specific phobia, Social anxiety disorder, Agoraphobia, Obsessive compulsive disorder, Cannabis use disorder, Cocaine use disorder, Amphetamine use disorder, Hallucinogen use disorder, Post traumatic stress disorder | None | DSM | NA | 7 |
| Metzler 2014^190^ | Switzerland | Longitudinal cohort | 177 | 52.5 APS, 4.0 BLIPS, 8.5 GRD | 21.1, 5.2 | 40.1 | SIPS | Any trauma disorder, Any eating disorder | Non-psychotic disorders | DSM | 12 | 8 |
| Meyer 2005^191^ | USA | Cross-sectional | 24 | 96.0 APS, 4.2 BLIPS, 4.2 GRD | 15.8, 2.2 (12-19) | 29.2 | SIPS | Specific phobia, Obsessive compulsive disorder, Post traumatic stress disorder | None | DSM | NA | 7 |
| Michel 2014^192^ | Germany | Longitudinal cohort | 97 | NA | 24.8, 5.5 (16-40) | 35.1 | SIPS, SPI-A | Depressive disorder NOS, Bipolar disorder-I, Other bipolar disorders, Panic disorder, Specific phobia, Social anxiety disorder, Agoraphobia, Anxiety disorder NOS, Obsessive compulsive disorder, Body dysmorphic disorder, Anorexia nervosa, Eating disorder NOS, Somatoform disorders, Hypochondriasis | None | DSM | 24 | 7 |
| Michel 2018^193^ | Germany | Longitudinal cohort | 194 | 80.9 APS, 10.8 BLIPS, 0 GRD | 24.9, 5.9 (14-40) | 37.0 | SIPS | Any non-psychotic mental disorder, Any mood disorder, Schizotypal personality disorder, Any anxiety disorder, Any substance use disorder, Cannabis use disorder NOS, Alcohol use disorder-NOS, Substance use disorder NOS, Any eating disorder, Somatoform disorders | Non-psychotic disorders | DSM | 96 | 8 |
| Millman 2017^194^ | USA | Cross-sectional | 45 | 82.2 APS, 11.1 BLIPS, 15.6 GRD | 15.2, 2.3 (12-22) | 67.0 | SIPS | Any non-psychotic mental disorder, Any mood disorder, Schizotypal personality disorder, Attention deficit hyperactivity disorder, Any behavioural Disorder, Any anxiety disorder, Post-traumatic stress disorder | Non-psychotic disorders | DSM | NA | 7 |
| Millman 2018^195^ | USA | Cross-sectional | 45 | 82.2 APS, 11.1 BLIPS, 15.5 GRD | 15.3, 2.3 (12-25) | 67.0 | SIPS | Any non psychotic mental disorder, Any mood disorder, Schizotypal personality disorder, Attention deficit hyperactivity disorder, Any behavioural disorder, Any anxiety disorder, Post traumatic stress disorder | Non-psychotic disorders | DSM | NA | 8 |
| Millman 2019^196^ | USA | Cross-sectional | 43 | NA | 15.9, 2.6 (12-25) | 69.8 | SIPS | Any non-psychotic mental disorder, Any mood disorder, Attention deficit hyperactivity disorder, Any anxiety disorder, Any substance use disorder, Post traumatic stress disorder | Non-psychotic disorders | DSM | NA | 7 |
| Millman 2020^197^ | USA | Longitudinal cohort | 22 | NA | 17.3, 3.3 (12-25) | 45.5 | SIPS | Any mood disorder, Attention deficit hyperactivity disorder, Any anxiety disorder, Any substance use disorder, Post traumatic stress disorder | None | DSM | 12 | 7 |
| Mittal 2010^198^ | USA | Longitudinal cohort | 90 | NA | 15.6, 3.0 (11.29) | 32.2 | SIPS | Any depressive disorder, Attention deficit hyperactivity disorder, Conduct disorder, Any anxiety disorder | None | DSM | 24 | 7 |
| Modinos 2014^199^ | UK | Longitudinal cohort | 52 | NA | 25.3, 4.3 | 38.0 | CAARMS | Any mood disorder, Any anxiety disorder | Non-psychotic disorders | DSM | 66 | 8 |
| Modinos 2021^200^ | UK | Longitudinal cohort | 67 | NA | 22.9, 3.9 | 40.0 | CAARMS | Cannabis use disorder | None | DSM | 15 | 8 |
| Morcillo 2015^201^ | UK | Longitudinal cohort | 60 | 100 APS, 11.7 GRD | 20.3, 2.4 (16-30) | 48.3 | CAARMS | Any non-psychotic mental disorder, Major depressive disorder, Other bipolar disorders, Panic disorder, Generalised anxiety disorder, Social anxiety disorder, Obsessive compulsive disorder, Post traumatic stress disorder | None | DSM | 24 | 7 |
| Morrison 2012^202^ | UK | Randomised clinical trial | 288 | NA | 20.7, 4.3 (14-35) | 37.5 | CAARMS | Any non psychotic mental disorder, Major depressive disorder, Dysthymia, Panic disorders, Generalised anxiety disorder, Specific phobia, Social anxiety disorders, Agoraphobia, Obsessive compulsive disorder, Post traumatic stress disorder | None | DSM | 24 | 4 |
| Mukkala 2014^203^ | Finland | Cross-sectional | 21 | NA | 22.8, 1.1 | 54.8 | SIPS | Any substance use disorder | Non-psychotic disorders, non clinical controls | DSM | NA | 8 |
| Nägele 2021^204^ | Germany | Longitudinal cohort | 30 | NA | 21.3, 3.5 | NA | SIPS | Any mood disorder, Any personality disorder, Any anxiety disorder, Any substance use disorder | None | DSM | 15 | 7 |
| Natsubori 2014^205^ | Japan | Longitudinal cohort | 24 | 87.5 APS, 8.3 BLIPS, 8.3 GRD | 21.7, 3.8 (15-29) | 50.0 | SIPS | Major depressive disorder, Anxiety disorder NOS, Adjustment disorder | None | DSM | 12 | 7 |
| Nelson 2012^206^ | Australia | Longitudinal cohort | 49 | 89.9 APS, 2.0 BLIPS, 22.4 GRD | 19.2, 2.9 (15-22) | 55.1 | CAARMS | Schizotypal personality disorder, Other disorders NOS | None | DSM | 18 | 7 |
| Nelson 2020^207^ | Australia | Cross-sectional | 50 | 98.0 APS, 4.0 BLIPS, 22.0 GRD | 18.8, 4.9 (15-25) | 56.0 | CAARMS | Schizotypal personality disorder, Schizoid personality disorder, Paranoid personality disorder | Psychotic disorders | DSM | NA | 7 |
| Niendam 2009^208^ | USA | Longitudinal cohort | 64 | 79.7 APS, 18.8 BLIPS, 1.6 GRD | 16.5, 2.5 (12-22) | 39.1 | SIPS | Obsessive compulsive disorder | None | DSM | 11 | 6 |
| O’Brien 2007^209^ | USA | Randomised clinical trial | 29 | NA | 15.9 (12-22) | 41.4 | SIPS | Any mood disorder, Dysthymia, Bipolar disorder-I, Oppositional deficient disorder, Any anxiety disorder, Panic disorder, Generalised anxiety disorder, Phobias-NOS, Obsessive compulsive disorder, Any substance use disorder, Adjustment disorder, Any eating disorder | None | DSM | 24 | 7 |
| Ohmuro 2015^210^ | Japan | Cross-sectional | 50 | 96.0 APS, 4.0 BLIPS, 16.0 GRD | 20.1, 4.3 (14-35) | 64.0 | CAARMS | Any non psychotic mental disorder, Any mood disorder, Major depressive disorder, Depressive disorder NOS, Other bipolar disorders, Mood disorder NOS, Pervasive developmental disorder NOS, Any anxiety disorder, Adjustment disorder, Any eating disorder, Somatoform disorders, Disassociative disorders | None | DSM | NA | 6 |
| Ohmuro 2017^211^ | Japan | Cross-sectional | 104 | 97.1 APS, 3.8 BLIPS, 16.3 GRD | 19.9, 4.2 (14-35) | 63.0 | CAARMS | Any non psychotic mental disorder, Any mood disorder, Major depressive disorder, Dysthymia, Depressive disorder NOS, Other bipolar disorder, Mood disorder NOS, Austism spectrum disorder, Pervasive developmental disorder NOS, Any anxiety disorder, Panic disorder, Generalised anxiety disorder, Social anxiety disorder, Anxiety disorder NOS, Obsessive compulsive disorder, Post traumatic stress disorder, Adjustment disorder, Any eating disorder, Somatoform disorders, Disassociative disorders | None | DSM | NA | 7 |
| Oppetit 2018^212^ | France | Longitudinal cohort | 73 | 89.0 APS | 21.1, 3.7 (15-30) | 32.9 | CAARMS | Any depressive disorder, Any personality disorder, Any anxiety disorder, Obsessive compulsive disorder, Any substance use disorder, Any eating disorder | None | DSM | 24 | 8 |
| Pantelis 2003^213^ | Australia | Longitudinal cohort | 75 | NA | 20.9, 3.6 (14-30) | 42.7 | CAARMS | Major depressive disorder, Dythymia, Any anxiety disorder, Panic disorders, Generalised anxiety disorder, Social anxiety disorder, Obsessive compulsive disorder, Adjustment disorder, Any eating disorder | None | DSM | 24 | 8 |
| Park 2021^214^ | South Korea | Cross-sectional | 24 | 95.8 APS, 12.5 BLIPS, 20.8 GRD | 21.9, 3.8 (15-35) | 33.3 | SIPS | Any depressive disorder, Panic disorder, Social anxiety disorder, Obsessive compulsive disorder, Somatform disorder, Dissociative disorder | None | DSM | NA | 6 |
| Pawelczyk 2021^215^ | Poland | Longitudinal cohort | 73 | 71.2 APS, 6.8 BLIPS, 21.9 GRD | 19.1, 4.1 | 57.5 | CAARMS | Schizoid personality disorder | None | DSM | 60 | 7 |
| Pelizza 2018^216^ | Italy | Longitudinal cohort | 25 | 88.0 APS, 8.0 BLIPS, 28.0 GRD | 14.8, 1.3 (13-18) | 52.0 | COGDIS, SPI-CY | Any depressive disorder, Schizotypal personality disorder | None | DSM | 12 | 7 |
| Pelizza 2019a^217^ | Italy | Longitudinal cohort | 79 | 91.1 APS, 3.8 BLIPS, 5.1 GRD | 18.6, 4.4 (13-35) | 54.4 | CAARMS | Any depressive disorder, Schizotypal personality disorder, Any anxiety disorder | None | DSM | NA | 7 |
| Pelizza 2019b^218^ | Italy | Longitudinal cohort | 79 | 91.1 APS, 3.8 BLIPS, 5.1 GRD | 18.6, 4.4 (13-35) | 54.4 | CAARMS | Any depressive disorder, Schizotypal personality disorder, Any anxiety disorder | None | DSM | NA | 7 |
| Pelizza 2020^219^ | Italy | Longitudinal cohort | 273 | NA | 21.1, 5.9 (13-35) | 42.9 | CAARMS | Any depressive disorder, Any anxiety disorder | None | DSM | 24 | 7 |
| Pelizza 2021^220^ | Italy | Longitudinal cohort | 78 | 91.0 APS, 3.8 BLIPS, 5.6 GRD | 18.6, 4.4 | 55.1 | CAARMS | Any substance use disorder | Psychotic disorders, Non-psychotic disorders | DSM | 12 | 7 |
| Perkins 2015^221^ | USA, Canada | Longitudinal cohort | 72 | 100 APS | 19.4, 4.2 (12-35) | 24 | SIPS | Any depressive disorder, Any anxiety disorder | None | DSM | 24 | 8 |
| Peters 2008^222^ | Netherlands | Longitudinal cohort | 10 | 90.0 APS, 30.0 BLIPS, 20.0 GRD | 21.6, 2.8 | 0 | BSABPS, PANSS | Any depressive disorder, Other bipolar disorders, Schizoid personality disorder | None | DSM | 18 | 8 |
| Phillips 2002a^223^ | Australia | Longitudinal cohort | 60 | 65.0 APS, 23.3 BLIPS, 41.7 GRD | 20.0, 3.3 (14-29) | 42.0 | CAARMS | Any non psychotic mental disorder, Major depressive disorder, Dysthymia, Panic disorders, Generalised anxiety disorder, Obsessive compulaive disorder, Adjustment disorder | None | DSM | 12 | 8 |
| Phillips 2002b^224^ | Australia | Longitudinal cohort | 100 | 67.0 APS, 27.0 BLIPS, 37.0 GRD | 19.3, 3.5 (14-28) | 51.0 | CAARMS | Cannabis use disorder, Hallucinogen use disorder, Opioid use disorder, Polysubstance use disorder | None | DSM | 12 | 8 |
| Phillips 2009^225^ | Australia | Randomised clinical trial | 193 | 83.9 APS, 7.8 BLIPS, 7.8 GRD | 17.9, 2.9 (14-30) | 60.6 | CAARMS, BPRS, SANS | Major depressive disorder, Dysthymia, Panic disorder, Generalised anxiety disorder, Specific phobia, Specific phobia, Social anxiety disorder, Any eating disorder | None | DSM | 24 | 8 |
| Piskulic 2012^226^ | USA, Canada | Longitudinal cohort | 138 | 100 APS | 18.6, 4.9 | 36.2 | SIPS | Any mood disorder, Any anxiety disorder, Any substance use disorder | None | DSM | 12 | 7 |
| Poletti 2019^227^ | Italy | Longitudinal cohort | 51 | 70.6 APS, 3.9 BLIPS, 3.0 GRD | 15.4, 1.6 (13-18) | 58.8 | COGDIS, SPI-CY | Obsessive compulsive disorder | None | DSM | 24 | 7 |
| Poletti 2021^228^ | Italy | Longitudinal cohort | 44 | 80.9 APS, 4.5 BLIPS, 4.5 GRD | 15.5, 2.0 | 59.1 | CAARMS | Major depressive disorder, Conduct disorder | Psychotic disorders, Non-psychotic disorders | DSM | NA | 7 |
| Pollak 2021^229^ | Multisite | Longitudinal cohort | 254 | 16.1 APS, 6.3 BLIPS | 22.7, 5.0 (14-45) | 46.5 | CAARMS | Major depressive disorder, Bipolar disorder-I, Other bipolar disorders, Adjustment disorder, Somatoform disorders | None | DSM | 24 | 8 |
| Pozza 2020a^230^ | Italy | Cross-sectional | 30 | NA | NA | NA | ERIraos | Any depressive disorder, Borderline personality disorder. Schizotypal personality disorder, Generalised anxiety disorder, Social anxiety disorder, Anxiety disorder NOS, Obsessive compulsive disorder, Adjustment disorder | Psychotic disorders | DSM | NA | 7 |
| Pozza 2020b^231^ | Italy | Randomised clinical trial | 58 | 100 APS, 5.2 BLIPS, 13.8 GRD | 25.7, 6.1 (16-35) | 32.8 | CAARMS | Any depressive disorder, Any personality disorder, Borderline personality disorder, Any anxiety disorder, Panic disorder, Generalised Anxiety disorder, Specific phobia, Social anxiety disorder, Agoraphobia, Obsessive compulsive disorder, Alcohol use disorder, Any eating disorder | None | DSM | 14 | 7 |
| Pozza 2021^232^ | Italy | Longitudinal cohort | 58 | 100 APS, 5.2 BLIPS, 13.8 GRD | 25.7, 6.0 (16-35) | 32.8 | CAARMS | Any non psychotic mental disorder, Any depressive disorder, Any personality disorder, Borderline personality disorder, Paranoid personality disorder, Avoidant personality disorder, Dependent personality disorder, Obsessive compulsive personality disorder, Any anxiety disorder, Obsessive compulsive disorder, Cannabis use disorder, Alcohol use disorder, Any eating disorder | None | DSM | 14 | 7 |
| Preda 2002^233^ | USA | Cross-sectional | 47 | NA | 17.0, 5.0 (12-35) | 32.0 | SIPS | Any mood disorder, Any depressive disorder, Other bipolar disorder, Autism spectrum disorder, Attention deficit hyperactivity disorder, Conduct disorder, Adjustment disorder | None | DSM | NA | 6 |
| Preti 2009^234^ | Italy | Longitudinal cohort | 81 | NA | 22.3, 3.7 | 29.6 | ERIraos; BPRS | Any substance use disorder | Psychotic disorders | ICD | 12 | 7 |
| Pruessner 2011^235^ | Canada | Cross-sectional | 30 | 83.3 APS, 3.3 BLIPS, 13.3 GRD | 20.3, 3.2 | 46.7 | CAARMS | Any depressive disorder, Any anxiety disorder, Obsessive compulsive disorder, Post traumatic stress disorder | None | DSM | NA | 7 |
| Raballo 2016^236^ | Italy | Longitudinal cohort | 29 | 96.6 APS, 3.4 BLIPS | 20.3, 2.8 (14-25) | 25.8 | SIPS, SPI-A-CY | Any mood disorder, Any personality disorder, Any anxiety disorder | Non-psychotic disorders | DSM | NA | 6 |
| Ramyead 2016^237^ | Switzerland | Longitudinal cohort | 53 | NA | 26.1, 7.5 | 37.7 | BSIP | Any substance use disorder | None | DSM | 36 | 8 |
| Randers 2020^238^ | Denmark | Cross-sectional | 39 | 92.3 APS, 2.6 BLIPS, 53.8 GRD | 23.4, 4.4 | 56.4 | CAARMS | Schizotypal personality disorder | None | DSM | NA | 7 |
| Ranlund 2014^239^ | UK | Cross-sectional | 33 | 84.8 APS, 18.2 BLIPS, 12.1 GRD | 23.8, 4.0 | 39.4 | CAARMS | Any depressive disorder, Any personality disorder, Any anxiety disorder, Any substance use disorder | Non clinical controls | DSM | NA | 6 |
| Ratheesh 2013^240^ | Australia | Longitudinal cohort | 62 | NA | 20.7, 3.5 (15-30) | 58.1 | CAARMS | Other bipolar disorders | None | DSM | 96 | 7 |
| Rausch 2013^6^ | Germany | Cross-sectional | 63 | NA | 24.6, 6.6 | 20.7 | ERIraos | Any non- psychotic mental disorder, Major depressive disorder, Any personality disorder, Cannabis use disorder | Psychotic disorders | DSM | NA | 6 |
| Reininghaus 2016^241^ | UK | Cross-sectional | 46 | NA | 23.6, 4.7 (18-35) | 54.1 | CAARMS | Any mood disorder, Any anxiety disorder | None | DSM | NA | 6 |
| Reininghaus 2019^242^ | UK | Cross-sectional | 46 | NA | 23.6, 4.7 (18-35) | 54.4 | CAARMS | Any mood disorder, Any anxiety disorder | None | DSM | NA | 6 |
| Rekhi 2018^243^ | Singapore | Cross-sectional | 167 | NA | 21.4, 3.5 (14-29) | 31.7 | CAARMS | Any depressive disorder, Depressive disorder NOS | None | DSM | NA | 7 |
| Rekhi 2019^244^ | Singapore | Longitudinal cohort | 173 | 96.5 APS | 21.3, 3.5 (14-29) | 32.4 | CAARMS |  | None | DSM | 12 | 7 |
| Ribolsi 2017^245^ | Italy | Cross-sectional | 94 | 100 APS | 14.6, 2.0 (9-18) | 47.9 | SIPS | Any non-psychotic mental disorder, Any depressive disorder, Schizotypal personality disorder, Any anxiety disorder, obsessive compulsive disorder | None | DSM | NA | 7 |
| Riecher-Rossler 2009^246^ | Switzerland | Longitudinal cohort | 64 | NA | 26.5, 8.6 | 40.6 | BSIP, BSABS-P | Any substance use disorder | None | DSM | 84 | 7 |
| Rietdijk 2013^247^ | Netherlands | Randomised clinical trial | 201 | 95.5 APS, 2.0 BLIPS, 16.9 GRD | 22.7, 5.5 (14-35) | 50.7 | CAARMS | Any depressive disorder, Social anxiety disorder | None | DSM | NA | 8 |
| Rietschel 2017^248^ | Germany | Cross-sectional | 239 | NA | 24.6, 5.4 (18-40) | 33.5 | SIPS | Any non-psychotic mental disorder, Any mood disorder, Any anxiety disorder | None | DSM | NA | 7 |
| Rosen 2006^249^ | USA | Cross-sectional | 29 | 96.6 APS, 3.5 BLIPS | 18.4, 4.8 (12-17) | 52.0 | SIPS | Any mood disorder, Major depressive disorder, Dysthymia, Depressive disorder NOS, Borderline personality disorder, Schizotypal personality disorder, Schizoid personality disorder, Paranoid personality disorder, Antisocial personality disorder, Avoidant personality disorder, Obsessive compulsive personality disorder, Depressive personality disorder, Any anxiety disorder, Panic disorder, Generalised anxiety disorder, Social anxiety disorder, Agoraphobia, Obsessive compulsive disorder, Any substance use disorder, Cannabis use disorder, Alcohol use disorder, Cocaine use disorder, Hallucinogen use disorder, Substance use disorder NOS, Post traumatic stress disorder | None | DSM | NA | 7 |
| Ruhrmann 2010^250^ | Multisite | Longitudinal cohort | 245 | NA | 23.0, 5.2 (15-35) | 44.1 | SIPS | Schizotypal personality disorder, Alcohol use disorder, Substance use disorder NOS | None | DSM | 18 | 8 |
| Russo 2014a^251^ | UK | Longitudinal cohort | 60 | 100 APS, 0 BLIPS, 11.7 GRD | 19.9, 2.4 (16-35) | 48.3 | CAARMS | Any depressive disorder, Other bipolar disorders, Panic disorders, Generalised anxiety disorder, Social anxiety disorder, Obsessive compulsive disorder, Post traumatic stress disorder | None | DSM | 24 | 7 |
| Russo 2014b^252^ | UK | Cross-sectional | 60 | 100 APS, 0 BLIPS, 11.7 GRD | 19.9, 2.4 (16-35) | 48.3 | CAARMS | Any non psychotic mental disorder, Major depressive disorder, Other bipolar disorders, Panic disorder, Generalised anxiety disorder, Social anxiety disorder, Obsessive compulsive disorder, Post traumatic stress disorder | None | DSM | NA | 7 |
| Russo 2018^253^ | UK | Longitudinal cohort | 60 | 100 APS | 19.9, 2.4 (16-31) | 48.3 | CAARMS | Any non-psychotic mental disorder, Any depressive disorder, Bipolar disorder-I, Panic disorder, Generalised anxiety disorder, Social anxiety disorder, Obsessive compulsive disorder, Post traumatic stress disorder | None | DSM, ICD | 24 | 7 |
| Rutligliano 2016^254^ | UK | Longitudinal cohort | 74 | 98.6 APS, 17.6 BLIPS, 12.2 GRD | 23.2, 4.9 (14-35) | 50.0 | CAARMS | Any non-psychotic mental disorder, Any mood disorder, Major depressive disorder, Dysthymia, Bipolar disorder-I, Other bipolar disorders, Mood disorder NOS, Any personality disorders, Borderline personality disorder, Paranoid personality disorder, Oppositional defiant disorder, Any anxiety disorder, Panic disorder, Generalised anxiety disorder, Specific phobia, Social anxiety disorder, Agoraphobia, Anxiety disorder NOS, Obsessive compulsive disorder, Body dysmorphic disorder, Post-traumatic stress disorder, Any eating disorder, Somatoform disorder | None | DSM | 120 | 7 |
| Ryan 2017^255^ | Australia | Cross-sectional | 180 | 84.4 APS, 38.9 BLIPS, 24.4 GRD | 18.2, 3.1 (15-24) | 37.2 | CAARMS | Borderline personality disorder | None | DSM | 12 | 7 |
| Sabb 2010^256^ | USA | Longitudinal cohort | 40 | NA | 17.4, 3.6 | 30.0 | SIPS | Major depressive disorder, Depressive disorder NOS, Other bipolar disorders, Pervasive developmental disorder NOS, Attention deficit hyperactivity disorder, Social anxiety disorders, Anxiety disorder NOS, Obsessive compulsive disorder, Alcohol use disorder, Adjustment disorder, Eating disorder NOS | None | DSM | 24 | 7 |
| Salinger 2018^257^ | USA, Canada | Cross-sectional | 58 | NA | 15.3, 1.3 (12-25) | 39.7 | SIPS | Any mood disorder, Learning disorder, Any anxiety disorder, Any substance use disorder | Non-psychotic disorders | DSM | NA | 7 |
| Salokangas 2012^258^ | Multisite | Longitudinal cohort | 245 | 84.1 APS, 11.4 BLIPS, 16.3 GRD | 22.4, 5.2 (15-35) | 44.1 | SIPS | Any depressive disorder, Other bipolar disorder, Any anxiety disorder, Any eating disorder, Somatoform disorder | None | DSM | 18 | 6 |
| Salokangas 2013^259^ | Multisite | Longitudinal cohort | 245 | 84.1 APS, 11.4 BLIPS, 16.3 GRD | 22.4, 5.2 (15-35) | 44.1 | SIPS | Cannabis use disorder | None | DSM | 18 | 7 |
| Salokangas 2016^260^ | Multisite | Longitudinal cohort | 245 | NA | 22.4, 5.2 (15-35) | 44.1 | SIPS | Any non-psychotic mental disorder, Any depressive disorder, Other bipolar disorders, Any anxiety disorders, Obsessive compulsive disorder, Any eating disorder, Somatoform disorder | None | DSM | 18 | 6 |
| Salokangas 2019^261^ | Germany | Longitudinal cohort | 238 | NA | 22.4 (15-35) | 45.0 | SIPS | Any depressive disorder | None | DSM | 18 | 6 |
| Schlosser 2012^262^ | USA | Longitudinal cohort | 84 | 77.5 APS, 20.2 BLIPS, 2.4 GRD | 16.9, 3.5 | 38.0 | SIPS | Any mood disorder, Any anxiety disorder | None | DSM | 24 | 7 |
| Schmidt 2014^263^ | Switzerland | Cross-sectional | 21 | 61.9 APS, 4.8 BLIPS | 19.4, 4.6 (12-28) | 76.7 | SIPS | Any depressive disorder, Somatoform disorders | Psychotic disorders | DSM | NA | 7 |
| Schmidt 2017^264^ | Switzerland | Cross-sectional | 73 | 4.1 BLIPS | 18.4, 4.6 (10-35) | 47.9 | SIPS | Major depressive disorder, Any substance use disorder | None | DSM | NA | 7 |
| Schultze-Lutter 2007a^265^ | Germany | Cross-sectional | 102 | NA | 23.9, 5.3 | 33.3 | SIPS, BSAS, PANSS; SPI-A | Any non-psychotic mental disorder, | None | DSM | NA | 7 |
| Schultze-Lutter 2007b^266^ | Germany | Cross-sectional | 146 | 80.0 APS, 0.7 BLIPS, 0 GRD | 24.4, 5.2 (16-40) | 30.8 | SIPS SPI-A, PANSS | Any depressive disorder, Major depressive disorder, Dysthymia | Psychotic disorders, Non-psychotic disorders | DSM | NA | 8 |
| Schultze-Lutter 2012^267^ | Germany | Longitudinal cohort | 100 | 84.0 APS, 13.0 BLIPS, 0 GRD | 24.0, 6.0 (16-38) | 24.0 | SIPS | Borderline personality disorder, Schizotypal personality disorder, Schizoid personality disorder, Paranoid personality disorder, Antisocial personality disorder, Avoidant personality disorder, Dependent personality disorder | None | DSM | 46 | 8 |
| Schultze‑Lutter 2017^268^ | Switzerland | Cross-sectional | 133 | 25.0 APS, 2.0 BLIPS, 0 GRD | 17.5 (8-40) | 42.9 | SIPS | Any mood disorder, Schizotypal personality disorder, Any anxiety disorder | None | DSM | NA | 8 |
| Schulze 2013^269^ | Switzerland | Longitudinal cohort | 47 | NA | 26.2, 8.7 | 42.6 | BSIPS | Any depressive disorder, Dysthymia, Adjustment disorder | Non-psychotic disorders | ICD | 65 | 8 |
| Shi 2017^270^ | China | Longitudinal cohort | 32 | 100 APS, 21.9 GRD | 18.8 (17-21) | 59.4 | SIPS | Major depressive disorder, Dysthymia, Panic disorder, Generalised anxiety disorder, Social anxiety disorder, Agoraphobia, obsessive compulsive disorder, Body dysmorphic disorder, Somatoform disorder | None | DSM, ICD | 6 | 7 |
| Simeonova 2015^271^ | USA | Longitudinal cohort | 53 | 98.1 APS, 0 BLIPS, 1.9 GRD | 14.2, 1.2 (12-18) | 24.0 | SIPS | Any personality disorders | None | DSM | 12 | 7 |
| Simon 2006^272^ | Switzerland | Cross-sectional | 54 | NA | 22.1, 5.8 (14-40) | 38.9 | SIPS | Any mood disorder, Any anxiety disorder, Adjustment disorder, Disassociative disorder | Psychotic disorders | DSM | NA | 7 |
| Simon 2007^273^ | Switzerland | Cross-sectional | 93 | 64.5 APS, 7.5 BLIPS, 2.2 GRD | 20.8, 5.0 (14-40) | 40.9 | SIPS | Any mood disorder, Any anxiety disorder, Adjustment disorder, Disassociative disorder | Non-psychotic disorders | DSM | NA | 7 |
| Simon 2010^274^ | Switzerland | Longitudinal cohort | 72 | 67.0 APS, 3.0 BLIPS, 2.0 GRD | 20.3, 4.9 (20+) | 40.2 | SIPS | Any mood disorder, Schizotypal personality disorder, Any anxiety disorder, Disassociative disorders | None | DSM | 12 | 6 |
| Simon 2012^275^ | Switzerland | Longitudinal cohort | 99 | NA | 20.7, 5.0 (14-40) | 38.4 | SIPS; SPI-A | Schizotypal personality disorder, Adjustment disorder, Any eating disorder, Disassociative disorder, Anxiety/mood disorder | Psychotic disorders, Non-psychotic disorders | DSM | 22 | 7 |
| Smith 2006^276^ | USA | Cross-sectional | 8 | NA | 16.3, 2.6 | 0 | SIPS | Any depressive disorder, Attention deficit hyperactivity disorder, Any anxiety disorder, | None | DSM | NA | 7 |
| Song 2013^277^ | South Korea | Longitudinal cohort | 50 | 94.0 APS, 22.0 BLIPS, 26.0 GRD | 20.0, 3.5 | 40.0 | SIPS | Any mood disorder, Other bipolar disorders, Any personality disorder, Panic disorder, Social anxiety disorders, Obsessive compulsive disorder, Any substance use disorder, Post traumatic stress disorder, Acute stress disorder, Any eating disorder | None | DSM | 24 | 7 |
| Spada 2016^278^ | Italy | Longitudinal cohort | 31 | 54.8 APS, 3.2 BLIPS, 12.0 GRD | 15.9, 1.5 (12-18) | 45.2 | CAARMS | Any non-psychotic mental disorder, Any mood disorder, Major depressive disorder, Depressive disorder NOS, Other bipolar disorders, Mood disorder NOS, Any personality disorder, Borderline personality disorder, Personality disorder NOS, Conduct disorder, Any anxiety disorder, Panic disorder, Generalised anxiety disorder, Agoraphobia, Anxiety disorder NOS, Obsessive compulsive disorder, Any substance use disorder, Any eating disorder, Anorexia nervosa | None | DSM | 7* | 8 |
| Spark 2021^279^ | Australia | Cross-sectional | 48 | NA | NA | NA | CAARMS | Any mood disorder, Schizotypal personality disorder, Paranoid personality disorder, Any substance use disorder | None | DSM | NA | 7 |
| Stain 2016^280^ | Australia | Randomised clinical trial | 57 | 81.0 APS, 7.0 BLIPS, 0 GRD | 16.5, 3.0 (14-30) | 59.6 | SIPS | Any mood disorder, Any Neurodevelopmental disorder | None | DSM | 12 | 6 |
| Stain 2018^281^ | Australia | Cross-sectional | 57 | 81.0 APS, 7.0 BLIPS, 33.3 GRD | 16.5, 2.9 (12-25) | 57.9 | CAARMS | Any depressive disorder, Any behavioural disorder, Any anxiety disorder, Any substance use disorder | None | DSM | NA | 5 |
| Sterk 2011^282^ | Netherlands | Longitudinal cohort | 29 | NA | 19.1, 3.4 | 10.3 | SIPS | Obsessive-compulsive disorder | Psychotic disorders, Non-psychotic disorders | DSM | 24 | 7 |
| Takahashi 2009^283^ | Australia | Longitudinal cohort | 97 | NA | 19.8, 3.4 (14-30) | 39.2 | CAARMS, SANS, BPRS | Substance induced mood disorder, Any anxiety disorder, Any eating disorder | None | DSM | 12 | 7 |
| Takahashi 2017^284^ | Japan | Longitudinal cohort | 33 | 100 APS | 17.7, 3.0 (15-30) | 45.5 | CAARMS | Any non-psychotic mental disorder | None | DSM | 26* | 7 |
| Takahashi 2018^285^ | Japan | Longitudinal cohort | 38 | 100 APS | 18.4, 3.9 (15-30) | 36.8 | CAARMS | Any depressive disorder, Schizotypal personality disorder, Any pervasive developmental disorder, Any anxiety disorder, Adjustment disorder, Dissociative disorder, | None | DSM | 30 | 6 |
| Takahashi 2019^286^ | Japan | Longitudinal cohort | 38 | NA | 18.4, 3.9 | 36.8 | CAARMS | Any depressive disorder, Schizotypal personality disorder, Any pervasive developmental disorder, Attention deficit hyperactivity disorder, Any anxiety disorder | None | DSM | 126 | 8 |
| Takahashi 2021^287^ | Japan | Longitudinal cohort | 57 | 100 APS, 1.8 BLIPS, 11.4 GRD | 18.6, 4.3 | 40.4 | CAARMS | Any depressive disorder, Schizotypal personality disorder, Any Pervasive developmental disorder, Any anxiety disorder, Adjustment disorder | None | DSM | 38 | 6 |
| Talib 2021^288^ | Brazil | Longitudinal cohort | 85 | NA | 24.9, 4.1 (18-30) | 63.5 | CAARMS | Any depressive disorder, Any anxiety disorder | None | DSM | 30* | 8 |
| Tamagni 2013^289^ | Switzerland | Longitudinal cohort | 53 | 86.3 APS or BLIPS or both | 26.8, 9.2 | 45.3 | BSIP, BRPS | Neurotic stress-related-somatoform disorder | None | ICD | 84 | 7 |
| Tateno 2021^290^ | Japan | Longitudinal cohort | 39 | 66.7 APS, 2.6 BLIPS, 2.6 GRD | 18.5, 4.4 (13-31) | 43.6 | CAARMS | Major depressive disorder, Depressive disorder NOS, Bipolar disorder-I, Schizoid personality disorder, Avoidant personality disorder, Autism spectrum disorder, Attention deficit hyperactivity disorder, Dissociatative disorder | None | DSM | 24 | 7 |
| Tay 2015^291^ | Singapore | Cross-sectional | 155 | 80.6 APS, 2.9 BLIPS, 28.0 GRD | 21.0, 3.5 (15-31) | 30.3 | CAARMS | Any nonpsychotic mental disorder, Any depressive disorder, Bipolar disorder-I, Adjustment disorder, Somatoform disorder | None | DSM | NA | 7 |
| Thompson 2007^292^ | Australia | Longitudinal cohort | 23 | 87.0 APS, 13.0 BLIPS, 30.4 GRD | 18.9, 3.3 (14-30) | 39.1 | CAARMS | Bipolar disorder-I, Bulimia | None | DSM | 24 | 7 |
| Thompson 2010^293^ | Australia | Cross-sectional | 92 | 80.4 APS, 5.4 BLIPS, 30.4 GRD | 18.0, 3.0 (15-24) | 65.2 | CAARMS | Any non psychotic mental disorder, Any depressive disorder, Any anxiety disorder, Post traumatic stress disorder, Adjustment disorder, Sexual trauma | None | DSM | NA | 8 |
| Thompson 2011^294^ | Australia | Longitudinal cohort | 104 | 66.3 APS, 27.9 BLIPS, 37.5 GRD | 19.4, 3.5 (14-30) | 51.0 | CAARMS | Any substance use disorder | None | DSM | 28 | 8 |
| Thompson 2012^295^ | Australia | Longitudinal cohort | 96 | 86.5 APS, 7.3 BLIPS, 24.0 GRD | 18.4, 2.7 (15-24) | 54.2 | CAARMS | Borderline personality disorder, Conduct disorder | None | DSM | 24 | 8 |
| Thompson 2015^296^ | USA | Cross-sectional | 35 | 80.0 APS, 5.7 BLIPS, 11.4 GRD | 15.0, 2.2 (12-21) | 71.4 | SIPS | Any mood disorder, Schizotypal personality disorder, Attention deficit hyperactivity disorder, Any behavioural disorder, Any anxiety disorder, Post traumatic stress disorder | Non-psychotic disorders | DSM | NA | 7 |
| Thompson 2020^297^ | USA | Longitudinal cohort | 22 | NA | 20.9, 2.6 | 45.5 | SIPS | Any depressive disorder, Other bipolar disorders, Attention deficit hyperactivity disorder, Any anxiety disorder, Any substance use disorder, Post traumatic stress disorder | Non-psychotic disorders | DSM | NA | 7 |
| Tomyshev 2017^298^ | Russia | Cross-sectional | 27 | NA | 20.7, 2.6 (17-28) | 0 | SIPS | Any depressive disorder, Schizotypal personality disorder | None | ICD | NA | 4 |
| Tor 2020^299^ | Spain | Cross-sectional | 81 | 81.5 APS, 1.2 BLIPS, 16.1 GRD | 15.1, 1.8 (10-17) | 59.3 | SIPS | Any depressive disorder, Any anxiety disorder | None | DSM | NA | 8 |
| Tseng 2018^300^ | Canada | Cross-sectional | 24 | NA | 23.6, 4.7 | 45.8 | SIPS | Major depressive disorder, Cannabis use disorder, Alcohol use disorder | Psychotic disorders | DSM | NA | 7 |
| Üçok 2021^301^ | Turkey | Cross-sectional | 107 | NA | 20.5, 4.6 | 27.1 | CAARMS | Any depressive disorder | None | CDSS (with cut off) | NA | 6 |
| Vaemes 2019^302^ | Norway | Cross-sectional | 31 | 90.3 APS, 0 BLIPS, 3.2 GRD | 19.0, 3.3 (15-30) | 42.0 | SIPS | Any non-psychotic mental disorder, Any mood disorder, Schizotypal personality disorder, Any anxiety disorder | Non-psychotic disorders | DSM | NA | 7 |
| Van Der Gaag 2012^303^ | Netherlands | Randomised clinical trial | 201 | NA | 22.8, 5.6 (14-35) | 51.1 | CAARMS | Any depressive disorder, Any personality disorder, Austism spectrum disorder, Attention deficit hyperactivity disorder, Oppositional defiant disorder, Any substance abuse disorder, post-traumatic stress disorder, Any eating disorder | None | DSM | 18 | 5 |
| Van der Steen 2017^304^ | Germany | Cross-sectional | 22 | NA | 25.2, 5.0 (19-38) | 22.7 | SIPS | Major depressive disorder, Dysthymia, Panic disorders, Social anxiety disorder, Obsessive compulsive disorder, Alcohol use disorder, Post traumatic stress disorder | None | DSM | NA | 5 |
| Van Rijn 2011^305^ | Netherlands | Cross-sectional | 36 | 88.9 APS, 0 BLIPS, 2.8 GRD | 15.2, 2.1 (12-18) | 30.6 | SIPS, BSABS-P | Dysthymia, Aspergers disorder, Attention deficit hyperactivity disorder, Generalised anxiety disorder, Obsessive compulsive disorder, Cannabis use disorder | None | DSM | NA | 6 |
| Velakoulis 2006^306^ | Australia | Longitudinal cohort | 135 | NA | 20.1, 3.6 (14-30) | 42.2 | CAARMS | Any non psychotic mental disorder, Major depressive disorder, Other bipolar disorders, Generalised anxiety disorder, Social anxiety disorder, Obsessive compulsive disorder, Adjustment disorder, Any eating disorder | None | DSM | NA | 6 |
| Velthorst 2009^307^ | Netherlands | Longitudinal cohort | 73 | 94.5 APS, 13.7 BLIPS, 6.8 GRD | 19.1, 3.9 (12-35) | 35.7 | SIPS; BSABS-P | Any non-psychotic mental disorder, Any mood disorder, Any neurodevelopmental disorder, Any anxiety disorder, Any substance use disorder, Disassociative disorder | None | DSM | 36 | 7 |
| Velthorst 2012^308^ | Netherlands | Cross-sectional | 201 | 90.0 APS, 2.0 BLIPS, 16.9 GRD | 22.8, 5.6 (14-35) | 51.1 | CAARMS | Any mood disorder, Other bipolar disorders, Any anxiety disorder, Obsessive-compulsive disorder | None | DSM | NA | 7 |
| Velthorst 2013^309^ | Australia | Cross-sectional | 127 | 89.8 APS, 3.9 BLIPS, 25.2 GRD | 18.2, 2.7 (14-30) | 58.3 | CAARMS | Post traumatic stress disorder, Adjustment disorder | None | DSM | NA | 7 |
| von Hohenberg, 2014^310^ | USA | Longitudinal cohort | 28 | 82.1 APS, 0 BLIPS, 7.1 GRD | 20.6, 3.9 (14-35) | 36.0 | SIPS; SPI-A | Any mood disorder, Schizotypal personality disorder, Avoidant personality disorder, Autism spectrum disorder, Learning disorder, Attention deficit hyperactivity disorder, Oppositional deficient disorder, Any anxiety disorder, Any eating disorder | None | DSM | 12 | 7 |
| Walker 2010^311^ | USA | Longitudinal cohort | 56 | 91.1 APS | 14.2, 1.7 (12-18) | 32.2 | SIPS | Major depressive disorder | None | DSM | 60 | 8 |
| Walterfang 2008^312^ | Australia | Longitudinal cohort | 75 | NA | 20.9 (14-30) | 42.6 | BPRS, SANS | Major depressive disorder, Dysthymia, Any anxiety disorder, Panic disorder, Generalised anxiety disorder, Social anxiety disorder, Obsessive compulsive disorder, Any eating disorder | None | DSM | 18 | 7 |
| Wang 2018^313^ | Singapore | Longitudinal cohort | 88 | NA | 21.4, 3.6 | 33.0 | CAARMS | Any depressive disorder, Any anxiety disorder | None | DSM | 84 | 7 |
| Webb 2015a^314^ | Multisite | Longitudinal cohort | 271 | NA  NA | 18.7  4.6 | 43.9 | SIPS | Anxiety/mood disorder, Any mood disorder, Any anxiety disorder, other bipolar disorders | Non-psychotic disorders | DSM | 48 | 7 |
| Welsh 2014^315^ | UK | Longitudinal cohort | 30 | NA | 15.8, 1.4 | 53.0 | CAARMS | Any depressive disorder, Any pervasive developmental disorder, Aspergers disorder, Any behavioural disorder, Any anxiety disorder, Generalised anxiety disorder, Social anxiety disorder, Alcohol use disorder, Post traumatic stress disorder, Eating disorder NOS | None | ICD | 6 | 5 |
| Wood 2007^316^ | Australia | Longitudinal cohort | 17 | 64.7 APS, 23.5 BLIPS, 29.4 GRD | 19.4, 3.5 (14-29) | 41.2 | BPRS | Major depressive disorder, Dysthymia, Generalised anxiety disorder, Obsessive compulsive disorder | None | DSM | 12 | 8 |
| Woodberry 2018^317^ | Multisite | Cross-sectional | 745 | NA | 18.9, 4.3 | 43.0 | SIPS | Any depressive disorder, Schizotypal personality disorder, attention deficit hyperactivity disorder | None | DSM | NA | 6 |
| Woods 2009^318^ | Multisite | Longitudinal cohort | 377 | 98.5 APS, 3.3 BLIPS, 12.5 GRD | 18.2 | 37.9 | SIPS | Any mood disorder, Major depressive disorder, Dysthymia, Other bipolar disorders, Mood disorder NOS, Any personality disorder, Borderline personality disorder. Schizotypal personality disorder, Schizoid personality disorder, Paranoid personality disorder, Antisocial personality disorder, Narcissitic personality disorder, Avoidant personality disorder, Dependent personality disorder, Obsessive compulsive personality disorder, Personality disorder NOS, Learning disorder, Attention deficit hyperactivity disorder, Conduct disorder, Oppositional defiant disorder, Any anxiety disorder, Panic disorder, Generalised anxiety disorder, Social anxiety disorder. Agoraphobia, Anxiety disorder NOS, Obsessive compulsive disorder, Any substance use disorder, Cannabis use disorder. Alcohol use disorder, Cocaine use disorder, Amphetamine use disorder, Polysubstance use disorder, Post traumatic stress disorder, Adjustment disorder, Any eating disorder | Non-psychotic disorders, non clinical controls | DSM | 33 | 8 |
| Woods 2013^319^ | Multisite | Cross-sectional | 391 | NA | 16.3 (10-45) | 39.4 | SIPS | Any depressive disorder, Any anxiety disorder | None | DSM | NA | 7 |
| Yang 2019^320^ | USA | Cross-sectional | 148 | NA | 18.6, 4.2 (12-35) | 34.2 | SIPS | Any non-psychotic mental disorder, Any depressive disorder, Bipolar disorder-I, Any personality disorder, Any neurodevelopmental disorder, Attention déficit hyperactivity disorder, Any anxiety disorder, Any substance use disorder, Post traumatic stress disorder | None | DSM | NA | 6 |
| Yee 2018^321^ | Singapore | Longitudinal cohort | 105 | NA | 21.8, 3.6 (14-29) | 32.0 | CAARMS | Bipolar disorder-I | None | DSM | 24 | 7 |
| Yucel 2003^322^ | Australia | Longitudinal cohort | 63 | 68.3 APS, 23.8 BLIPS, 38.1 GRD | 19.4, 3.8 (14-30) | 0 | CAARMS | Any non psychotic mental disorder, Major depressive disorder, Dysthymia, Panic disorders, Social anxiety disorder, Obsessive compulsive disorder, Adjustment disorder | None | DSM | 12 | 6 |
| Yung 2003^323^ | Australia | Longitudinal cohort | 49 | 71.4 APS, 24.5 BLIPS, 26.7 GRD | 19.1, 3.8 (14-28) | 49.0 | CAARMS | Any mood disorder, Any anxiety disorder | None | DSM | 12 | 8 |
| Yung 2006^324^ | Australia | Longitudinal cohort | 119 | 93.3 APS, 0 BLIPS, 10.9 GRD | 18.1 (15-24) | 51.0 | CAARMS | Any anxiety disorder, Any substance use disorder | None | DSM | 6 | 7 |
| Yung 2007^325^ | Australia | Longitudinal cohort | 149 | NA | (15-24) | NA | CAARMS | Any mood disorder | None | DSM | 6 | 7 |
| Ziermans 2009^326^ | Netherlands | Longitudinal cohort | 54 | 80.0 APS, 3.3 BLIPS, 1.7 GRD | 15.8, 2.1 | 38.9 | SIPS, BSABS-P | Pervasive developmental disorder NOS | None | DSM | 18 | 8 |
| Zikidi 2020^327^ | UK | Cross-sectional | 114 | 73.7 APS, 1.7 GRD | 21.7, 4.5 | 74.5 | CAARMS, SPI-A | Any mood disorder, Any anxiety disorder, Obsessive compulsive disorder, Alcohol use disorder, Substance use disorder NOS, Post traumatic stress disorder, Any eating disorder | Non-psychotic disorders | DSM | NA | 7 |
| Zink 2014^328^ | Germany | Longitudinal cohort | 233 | NA | 24.6, 5.4 (18-40) | 33.9 | SIPS | Obsessive compulsive disorder | None | DSM | NA | 7 |

***Legend.*** *APS: Attenuated Psychosis Symptoms; BLIPS: Brief Limited Intermittent Psychotic Symptoms; BPRS: Brief Psychiatric Rating Scale;*

*BSABS-P: Bonn Scale for the Assessment of Basic Symptoms; BSIP: Basel Screening Instrument for Psychosis; CAARMS: Comprehensive Assessment of At-Risk Mental States; CHR-P: Clinical High Risk of Psychosis; DSM: Diagnostic and Statistical Manual of Mental Disorders; ERIraos: Early Recognition Inventory; GRD: Genetic risk and deterioration syndrome; HoNOS: Health of the Nation Outcome Scales; ICD: International Classification of Diseases; NOS: Newcastle-Ottawa Scale; PANSS: Positive and Negative Syndrome Scale; SANS: Scale for the Assessment of Negative Symptoms; SIPS: Structured Interview for Prodromal Syndromes; SPI: Schizophrenia Proneness Instrument; SPI-A: Schizophrenia Proneness Instrument-Adult; SPI-CY: The Schizophrenia Proneness Instrument, Child and Youth Version*

**eTable 4.** Meta-analytical proportion of comorbid mental disorders in CHR-P individuals at follow-up (from 6 to 24 months)

|  |  |  | | **Test for Heterogeneity** | | |  |  |
| --- | --- | --- | --- | --- | --- | --- | --- | --- |
| **Outcome** | **Prevalence** | **95 % CI** | | **Q** | **I^2^** | **Tau^2^** | **Sample size** | **N of studies** |
| Any non-psychotic mental disorder | 0.423 | 0.215 | 0.631 | 43.057 | 93.032 | 0.040 | 493 | 4 |
| Any depressive episode/disorder | 0.267 | 0.165 | 0.369 | 32.746 | 87.785 | 0.011 | 1196 | 5 |
| Any mood disorder | 0.247 | 0.033 | 0.461 | 120.284 | 97.506 | 0.046 | 1043 | 4 |
| Cannabis use disorder | 0.245 | -0.201 | 0.690 | 28.762 | 96.523 | 0.100 | 811 | 2 |
| Any anxiety disorder | 0.237 | 0.113 | 0.361 | 127.403 | 95.291 | 0.025 | 1359 | 7 |
| Major depressive disorder | 0.137 | -0.064 | 0.338 | 118.837 | 98.317 | 0.031 | 869 | 3 |
| ADHD | 0.097 | -0.053 | 0.247 | 37.009 | 97.298 | 0.110 | 794 | 2 |
| Social anxiety disorder (or social phobia) | 0.091 | 0.024 | 0.159 | 5.468 | 81.713 | 0.002 | 819 | 2 |
| GAD | 0.053 | 0.001 | 0.105 | 14.321 | 86.035 | 0.002 | 881 | 3 |
| Any substance use disorder | 0.053 | -0.025 | 0.132 | 6.858 | 85.419 | 0.003 | 389 | 2 |
| Panic disorder | 0.050 | 0.013 | 0.086 | 3.228 | 69.019 | 0.000 | 819 | 2 |
| OCD | 0.047 | 0.035 | 0.060 | 0.561 | 0.000 | 0.000 | 1164 | 4 |
| Dysthymia | 0.039 | -0.022 | 0.101 | 3.887 | 74.276 | 0.002 | 819 | 2 |
| Other bipolar disorder | 0.037 | 0.012 | 0.062 | 11.631 | 65.608 | 0.000 | 1123 | 5 |
| Somatoform disorder | 0.025 | -0.026 | 0.076 | 12.605 | 92.067 | 0.001 | 989 | 2 |
| PTSD | 0.015 | 0.007 | 0.023 | 0.658 | 0.000 | 0.000 | 904 | 3 |
| Any eating disorder | 0.015 | 0.008 | 0.023 | 0.076 | 0.000 | 0.000 | 1064 | 3 |
| Adjustment disorder | 0.012 | -0.004 | 0.028 | 0.014 | 0.000 | 0.000 | 175 | 2 |
| Anorexia nervosa | 0.007 | -0.011 | 0.026 | 1.433 | 30.211 | 0.000 | 806 | 2 |

Legend. *CHR-P, clinical high risk for psychosis; CI, confidence interval*

**eTable 5.** Meta-analytical proportion of comorbid mental disorders in CHR-P individuals at follow-up (from over 24 months to 48 months)

|  |  |  |  | **Test for Heterogeneity** | | |  |  |
| --- | --- | --- | --- | --- | --- | --- | --- | --- |
| **Outcome** | **Prevalence** | **95 % CI** | | **Q** | **I^2^** | **Tau^2^** | **Sample size** | **N of studies** |
| Any depressive episode/disorder | 0.123 | 0.008 | 0.237 | 4.108 | 75.658 | 0.005 | 159 | 2 |
| Other bipolar disorder (e.g. BD-NOS, BD-II) | 0.028 | -0.002 | 0.057 | 0.004 | 0.000 | 0.000 | 122 | 2 |

Legend. *BD, bipolar disorder; CHR-P, clinical high risk for psychosis; CI, confidence interval; NOS, not otherwise specified*

**eTable 6.** Meta-analytical proportion of comorbid mental disorders in CHR-P individuals at follow-up (from over 48 to 96 months)

|  |  |  |  | **Test for Heterogeneity** | | |  |  |
| --- | --- | --- | --- | --- | --- | --- | --- | --- |
| **Outcome** | **Prevalence** | **95 % CI** | | **Q** | **I^2^** | **Tau^2^** | **Sample size** | **N of studies** |
| Any non-psychotic mental disorder | 0.462 | 0.403 | 0.521 | 3.002 | 0.067 | 0.000 | 510 | 4 |
| Any mood disorder | 0.414 | 0.243 | 0.585 | 21.343 | 85.944 | 0.022 | 528 | 4 |
| Any anxiety disorder | 0.240 | 0.135 | 0.345 | 35.220 | 88.643 | 0.013 | 756 | 5 |
| Major depressive disorder | 0.186 | -0.119 | 0.492 | 19.709 | 94.926 | 0.046 | 137 | 2 |
| Any substance use disorder | 0.180 | 0.071 | 0.290 | 47.467 | 91.573 | 0.012 | 719 | 5 |
| Any depressive episode/disorder | 0.135 | 0.007 | 0.264 | 6.633 | 84.923 | 0.007 | 235 | 2 |
| OCD | 0.056 | 0.021 | 0.092 | 4.182 | 52.174 | 0.001 | 437 | 3 |
| Other bipolar disorder | 0.046 | -0.008 | 0.099 | 1.548 | 35.408 | 0.001 | 125 | 2 |
| Cannabis use disorder | 0.039 | 0.015 | 0.062 | 0.646 | 0.000 | 0.000 | 275 | 2 |
| Any eating disorder | 0.035 | 0.015 | 0.055 | 0.427 | 0.000 | 0.000 | 333 | 3 |
| Somatoform disorder | 0.034 | 0.011 | 0.057 | 0.001 | 0.000 | 0.000 | 252 | 2 |
| Schizotypal personality disorder | 0.032 | 0.010 | 0.055 | 0.062 | 0.000 | 0.000 | 238 | 2 |
| Alcohol use disorder | 0.032 | 0.011 | 0.054 | 0.047 | 0.000 | 0.000 | 275 | 2 |

*Legend. CHR-P, clinical high risk for psychosis; CI, confidence interval; NOS, not otherwise specified; OCD, obsessive-compulsive disorder*

**eTable 7.** Meta-analytical proportion of comorbid mental disorders in CHR-P individuals at follow-up (over 96 months)

|  |  |  |  | **Test for Heterogeneity** | | |  |  |
| --- | --- | --- | --- | --- | --- | --- | --- | --- |
| **Outcome** | **Prevalence** | **95 % CI** | | **Q** | **I^2^** | **Tau^2^** | **Sample size** | **N of studies** |
| Any non-psychotic mental disorder | 0.511 | 0.252 | 0.769 | 23.410 | 91.457 | 0.047 | 374 | 3 |
| Any mood disorder | 0.290 | -0.094 | 0.674 | 44.687 | 97.762 | 0.075 | 300 | 2 |
| Any anxiety disorder | 0.234 | 0.015 | 0.452 | 15.662 | 93.615 | 0.023 | 300 | 2 |
| Any substance use disorder | 0.193 | 0.000 | 0.387 | 15.067 | 93.363 | 0.018 | 300 | 2 |
| Panic disorder | 0.112 | 0.074 | 0.150 | 0.318 | 0.000 | 0.000 | 300 | 2 |
| Social anxiety disorder (or social phobia) | 0.094 | 0.053 | 0.135 | 1.311 | 23.739 | 0.000 | 300 | 2 |
| GAD | 0.066 | 0.037 | 0.095 | 0.264 | 0.000 | 0.000 | 300 | 2 |
| Other bipolar disorder (e.g. BD-NOS, BD-II) | 0.059 | -0.006 | 0.124 | 14.497 | 86.204 | 0.002 | 362 | 3 |
| BD-I | 0.022 | 0.005 | 0.039 | 0.548 | 0.000 | 0.000 | 300 | 2 |

Legend. *BD, bipolar disorder; CHR-P, clinical high risk for psychosis; CI, confidence interval; NOS, not otherwise specified*

**eTable 8.** Meta-analytic prevalence of baseline comorbid disorders in CHR-P individuals compared to non-psychotic controls

| **Outcome** | **OR** | **95% CI** | | **P value** | **Q** | **I^2^** | **Tau^2^** | **Egger’s test** | **Corrected OR** | **N of studies** | **N of CHR** | **N of Non psychotic controls** |
| --- | --- | --- | --- | --- | --- | --- | --- | --- | --- | --- | --- | --- |
| **Anxiety disorder NOS** | **2.898** | **1.430** | **5.872** | **0.003** | **0.003** | **0.000** | **0.000** | **d.n.a.** | **d.n.a.** | **2** | **286** | **299** |
| **Panic disorder** | **2.556** | **1.059** | **6.174** | **0.037** | **6.784** | **55.778** | **0.418** | **0.964** | **d.n.a.** | **4** | **1145** | **843** |
| **Any anxiety disorder** | **1.750** | **1.363** | **2.247** | **0.000** | **44.370** | **54.924** | **0.156** | **0.367** | **d.n.a.** | **21** | **2321** | **2329** |
| **Schizotypal personality disorder** | **1.540** | **1.121** | **2.115** | **0.008** | **0.936** | **0.000** | **0.000** | **0.511** | **d.n.a.** | **4** | **241** | **59** |
| **Alcohol use disorder** | **1.538** | **1.044** | **2.266** | **0.030** | **5.903** | **15.296** | **0.038** | **0.824** | **d.n.a.** | **6** | **1286** | **892** |
| Major depressive disorder | 6.466 | 0.910 | 45.923 | 0.062 | 74.464 | 95.971 | 3.751 | 0.547 | d.n.a. | 4 | 1059 | 818 |
| Any non-psychotic mental disorder | 2.961 | 0.252 | 34.747 | 0.388 | 1.331 | 24.864 | 0.788 | d.n.a. | d.n.a. | 2 | 76 | 72 |
| Dysthymia | 2.411 | 0.577 | 10.082 | 0.228 | 13.003 | 84.619 | 1.339 | 0.382 | d.n.a. | 3 | 1037 | 791 |
| OCD | 1.887 | 0.778 | 4.573 | 0.160 | 21.291 | 67.123 | 0.903 | 0.935 | d.n.a. | 8 | 1599 | 1117 |
| Other bipolar disorder (e.g. BD-NOS, BD-II) | 1.851 | 0.927 | 3.696 | 0.081 | 4.771 | 0.000 | 0.000 | 0.000 | 1.371 | 6 | 666 | 922 |
| PTSD | 1.708 | 0.875 | 3.333 | 0.117 | 11.827 | 40.811 | 0.346 | 0.474 | d.n.a. | 8 | 1491 | 1040 |
| Substance use disorder NOS | 1.608 | 0.711 | 3.634 | 0.254 | 0.064 | 0.000 | 0.000 | d.n.a. | d.n.a. | 2 | 868 | 286 |
| Social anxiety disorder (or social phobia) | 1.585 | 0.474 | 5.304 | 0.454 | 12.518 | 84.023 | 0.937 | 0.475 | d.n.a. | 3 | 658 | 744 |
| Any personality disorder | 1.496 | 0.762 | 2.936 | 0.242 | 21.100 | 81.043 | 0.393 | 0.200 | d.n.a. | 5 | 1074 | 1417 |
| Any mood disorder | 1.465 | 0.989 | 2.176 | 0.057 | 56.434 | 71.648 | 0.397 | 0.051 | No change | 17 | 1869 | 1125 |
| ODD | 1.295 | 0.893 | 1.878 | 0.172 | 0.197 | 0.000 | 0.000 | d.n.a. | d.n.a. | 2 | 776 | 274 |
| GAD | 1.279 | 0.543 | 3.008 | 0.573 | 12.068 | 66.854 | 0.569 | 0.335 | d.n.a. | 5 | 1395 | 930 |
| Any substance use disorder | 1.271 | 0.743 | 2.176 | 0.381 | 49.449 | 73.710 | 0.597 | 0.982 | d.n.a. | 14 | 1858 | 2250 |
| Dependent personality disorder | 1.170 | 0.168 | 8.158 | 0.874 | 1.613 | 37.997 | 0.845 | d.n.a. | d.n.a. | 2 | 864 | 297 |
| Any depressive disorder/episode | 1.151 | 0.668 | 1.985 | 0.612 | 55.299 | 81.916 | 0.607 | 0.054 | No change | 11 | 1217 | 1628 |
| Any eating disorder | 0.962 | 0.253 | 3.662 | 0.954 | 10.185 | 50.909 | 1.386 | 0.452 | d.n.a. | 6 | 1248 | 541 |
| Borderline personality disorder | 0.930 | 0.539 | 1.605 | 0.794 | 3.596 | 16.579 | 0.059 | 0.841 | d.n.a. | 4 | 1039 | 420 |
| Any neurodevelopmental disorder | 0.774 | 0.351 | 1.706 | 0.525 | 0.001 | 0.000 | 0.000 | d.n.a. | d.n.a. | 2 | 93 | 305 |
| Autism spectrum disorder | 0.761 | 0.284 | 2.039 | 0.596 | 1.138 | 12.125 | 0.120 | d.n.a. | d.n.a. | 2 | 13 | 19 |
| ADHD | 0.692 | 0.384 | 1.245 | 0.219 | 17.381 | 59.725 | 0.344 | 0.394 | d.n.a. | 8 | 1062 | 538 |
| Conduct disorder | 0.651 | 0.269 | 1.577 | 0.342 | 1.561 | 35.943 | 0.210 | d.n.a. | d.n.a. | 2 | 761 | 287 |
| Other disorders NOS | 0.584 | 0.268 | 1.274 | 0.177 | 8.562 | 41.60 | 0.386 | 0.130 | d.n.a. | 6 | 446 | 637 |
| Adjustment disorder | 0.555 | 0.207 | 1.486 | 0.241 | 16.192 | 69.121 | 0.955 | 0.566 | d.n.a. | 6 | 1157 | 1092 |
| Narcissistic personality disorder | 0.494 | 0.064 | 3.803 | 0.498 | 2.976 | 66.393 | 1.517 | d.n.a. | d.n.a. | 2 | 864 | 297 |
| Schizoid personality disorder | 0.428 | 0.051 | 3.561 | 0.432 | 2.258 | 55.705 | 1.576 | d.n.a. | d.n.a. | 2 | 864 | 297 |
| Histrionic personality disorder | 0.308 | 0.007 | 13.884 | 0.461 | 3.913 | 74.445 | 5.659 | d.n.a. | d.n.a. | 2 | 487 | 99 |
| Cannabis use disorder | 0.274 | 0.006 | 12.970 | 0.511 | 18.421 | 89.143 | 10.117 | 0.569 | d.n.a. | 3 | 883 | 330 |

***Legend.*** *ADHD, attention-deficit/hyperactivity disorder; BD, bipolar disorder; CI, confidence interval; OCD, obsessive-compulsive disorder; OR, odds ratio; k, number of studies; *, corrected OR after trim-and-fill analyses.*

**eTable 9.** Meta-analytic prevalence of baseline comorbid disorders in CHR-P individuals compared to psychotic controls

| **Outcome** | **Point estimate (OR)** | **95% CI** | | **P value** | **Q** | **I^2^** | **Tau^2^** | **Egger’s test** | **Corrected OR*** | **k** | **N of CHR** | **N of Non psychotic controls** |
| --- | --- | --- | --- | --- | --- | --- | --- | --- | --- | --- | --- | --- |
| **Social anxiety disorder (or social phobia)** | **9.297** | **1.176** | **73.501** | **0.035** | **0.075** | **0.000** | **0.000** | **d.n.a.** | **d.n.a.** | **2** | **122** | **76** |
| **Any mood disorder** | **4.622** | **2.231** | **9.575** | **0.000** | **21.086** | **66.803** | **0.688** | **0.316** | **d.n.a.** | **8** | **667** | **258** |
| **Any depressive disorder** | **2.183** | **1.747** | **2.728** | **0.000** | **1.447** | **0.000** | **0.000** | **0.049** | **2.095** | **5** | **641** | **909** |
| **Any anxiety disorder** | **2.019** | **1.621** | **2.514** | **0.000** | **6.673** | **0.000** | **0.000** | **0.406** | **d.n.a.** | **10** | **969** | **940** |
| **Any substance use disorder** | **0.408** | **0.203** | **0.818** | **0.012** | **32.919** | **75.624** | **0.765** | **0.000** | **0.290** | **9** | **1002** | **1072** |
| Dissociative disorder | 2.427 | 0.327 | 18.019 | 0.386 | 0.929 | 0.000 | 0.000 | d.n.a. | d.n.a. | 2 | 159 | 104 |
| OCD | 1.971 | 0.637 | 6.098 | 0.239 | 1.708 | 0.000 | 0.000 | 0.082 | 1.589 | 4 | 147 | 337 |
| Any personality disorder | 1.341 | 0.986 | 1.823 | 0.062 | 0.025 | 0.000 | 0.000 | d.n.a. | d.n.a. | 2 | 444 | 704 |
| Panic disorder | 0.983 | 0.176 | 5.490 | 0.984 | 0.605 | 0.000 | 0.000 | d.n.a. | d.n.a. | 2 | 86 | 73 |
| Any non-psychotic mental disorder | 0.960 | 0.458 | 2.011 | 0.914 | 2.432 | 17.752 | 0.078 | 0.524 | d.n.a. | 3 | 124 | 67 |
| Schizoid personality disorder | 0.818 | 0.259 | 2.579 | 0.731 | 0.351 | 0.000 | 0.000 | d.n.a. | d.n.a. | 2 | 112 | 59 |
| ODD | 0.786 | 0.307 | 2.010 | 0.615 | 0.029 | 0.000 | 0.000 | d.n.a. | d.n.a. | 2 | 84 | 40 |
| ADHD | 0.646 | 0.299 | 1.398 | 0.268 | 1.290 | 0.000 | 0.000 | 0.941 | d.n.a. | 4 | 134 | 107 |
| Schizotypal personality disorder | 0.596 | 0.303 | 1.174 | 0.135 | 1.388 | 0.000 | 0.000 | 0.306 | d.n.a. | 3 | 211 | 107 |
| Cannabis use disorder | 0.547 | 0.154 | 1.938 | 0.350 | 5.854 | 65.834 | 0.816 | 0.814 | d.n.a. | 3 | 114 | 233 |

***Legend.*** *CI, confidence interval; OCD, obsessive-compulsive disorder; OR, odds ratio; k, number of studies; *, corrected OR after trim-and-fill analyses*

**Supplementary references**

1. Page MJ, McKenzie JE, Bossuyt PM, et al. The PRISMA 2020 statement: An updated guideline for reporting systematic reviews. *BMJ*. 2021;372. doi:10.1136/bmj.n71

2. Stroup DF, Berlin JA, Morton SC, Olkin I, Williamson GD RD. MOOSE Guidelines for Meta-Analyses and Systematic Reviews of Observational Studies. *Jama*. Published online 2000.

3. Salazar de Pablo G, Davies C, de Diego H, et al. Transition to psychosis in randomized clinical trials of individuals at clinical high risk of psychosis compared to observational cohorts: a systematic review and meta-analysis. *Eur Psychiatry*. 2021;64(1):e51. doi:10.1192/j.eurpsy.2021.2222

4. Yung AR, Yung AR, Pan Yuen H, et al. Mapping the Onset of Psychosis: The Comprehensive Assessment of At-Risk Mental States. *Aust New Zeal J Psychiatry*. 2005;39(11-12):964-971. doi:10.1080/j.1440-1614.2005.01714.x

5. Miller TJ, McGlashan TH, Rosen JL, et al. Prodromal Assessment With the Structured Interview for Prodromal Syndromes and the Scale of Prodromal Symptoms: Predictive Validity, Interrater Reliability, and Training to Reliability. *Schizophr Bull*. 2003;29(4):703-715. doi:10.1093/oxfordjournals.schbul.a007040

6. Rausch F, Eifler S, Esser A, et al. The Early Recognition Inventory ERIraos detects at risk mental states of psychosis with high sensitivity. *Compr Psychiatry*. 2013;54(7):1068-1076. doi:https://doi.org/10.1016/j.comppsych.2013.04.016

7. Riecher-Rössler A, Aston J, Ventura J, et al. Das Basel Screening Instrument für Psychosen (BSIP): Entwicklung, aufbau, reliabilität und validität. *Fortschritte der Neurol Psychiatr*. 2008;76(4):207-216. doi:10.1055/S-2008-1038155/ID/43

8. Vollmer-Larsen A, Handest P, Parnas J. Reliability of Measuring Anomalous Experience: The Bonn Scale for the Assessment of Basic Symptoms. *Psychopathology*. 2007;40(5):345-348. doi:10.1159/000106311

9. Schultze-Lutter F, Addington J, Ruhrmann S, Klosterkötter J. Schozophrenia Proneness Instrument, Adult version (SPI-A). Published online 2007.

10. Fux L, Walger P, Schimmelmann BG, Schultze-Lutter F. The Schizophrenia Proneness Instrument, Child and Youth version (SPI-CY): Practicability and discriminative validity. *Schizophr Res*. 2013;146(1):69-78. doi:https://doi.org/10.1016/j.schres.2013.02.014

11. American Psychiatric Association. *Diagnostic and Statistical Manual of Mental Disorders : DSM-5.*; 2013.

12. WHO. *International Classification of Diseases - 11 (ICD-11). Version: 2020.*; 2019. https://icd.who.int/browse11/l-m/en

13. Wells G, Shea B, O’Connell D, et al. The Newcastle-Ottawa Scale (NOS) for assessing the quality if nonrandomized studies in meta-analyses. *(Available from URL http//www.ohri.ca/programs/clinical_epidemiology/oxford.asp)*. Published online 2012. doi:10.2307/632432

14. DerSimonian R, Laird N. Meta-analysis in clinical trials. *Control Clin Trials*. 1986;7(3):177-188. doi:10.1016/0197-2456(86)90046-2

15. Egger M, Smith GD, Schneider M, Minder C. Bias in meta-analysis detected by a simple , graphical test measures of funnel plot asymmetry. *Br Med J - Clin Res*. 1997;315(7109):629-634. doi:10.1136/bmj.315.7109.629

16. Provenzani U, Salazar de Pablo G, Arribas M, Pillmann F, Fusar-Poli P. Clinical outcomes in brief psychotic episodes: a systematic review and meta-analysis. *Epidemiol Psychiatr Sci*. 2021;30:e71-e71. doi:10.1017/S2045796021000548

17. Fusar-Poli P, Rutigliano G, Stahl D, et al. Deconstructing Pretest Risk Enrichment to Optimize Prediction of Psychosis in Individuals at Clinical High Risk. *JAMA psychiatry*. 2016;73(12):1260-1267. doi:10.1001/jamapsychiatry.2016.2707

18. Fusar-Poli P, Cappucciati M, Borgwardt S, et al. Heterogeneity of Psychosis Risk Within Individuals at Clinical High Risk: A Meta-analytical Stratification. *JAMA Psychiatry*. 2016;73(2):113-120. doi:10.1001/jamapsychiatry.2015.2324

19. Addington J, Cadenhead KS, Cannon TD, et al. North American Prodrome Longitudinal Study: A Collaborative Multisite Approach to Prodromal Schizophrenia Research. *Schizophr Bull*. 2007;33(3):665-672. doi:10.1093/schbul/sbl075

20. Addington J, Epstein I, Liu L, French P, Boydell KM, Zipursky RB. A randomized controlled trial of cognitive behavioral therapy for individuals at clinical high risk of psychosis. *Schizophr Res*. 2011;125(1):54-61. doi:https://doi.org/10.1016/j.schres.2010.10.015

21. Addington J, Cornblatt BA, Cadenhead KS, et al. At clinical high risk for psychosis: Outcome for nonconverters. *Am J Psychiatry*. 2011;168(8):800-805. doi:10.1176/appi.ajp.2011.10081191

22. Addington J, Cadenhead KS, Cornblatt BA, et al. North American Prodrome Longitudinal Study (NAPLS 2): Overview and recruitment. *Schizophr Res*. 2012;142(1-3):77-82. doi:10.1016/j.schres.2012.09.012

23. Addington J, Stowkowy J, Cadenhead KS, et al. Early traumatic experiences in those at clinical high risk for psychosis. *Early Interv Psychiatry*. 2013;7(3):300-305. doi:10.1111/eip.12020

24. Addington J, Piskulic D, Liu L, et al. Comorbid diagnoses for youth at clinical high risk of psychosis. *Schizophr Res*. 2017;190:90-95. doi:10.1016/j.schres.2017.03.043

25. Addington J, Liu L, Goldstein BI, et al. Clinical staging for youth at-risk for serious mental illness. *Early Interv Psychiatry*. 2019;13(6):1416-1423. doi:10.1111/eip.12786

26. Addington J, Stowkowy J, Liu L, et al. Clinical and functional characteristics of youth at clinical high-risk for psychosis who do not transition to psychosis. *Psychol Med*. 2019;49(10):1670-1677. doi:10.1017/S0033291718002258

27. Allott KA, Schäfer MR, Thompson A, et al. Emotion recognition as a predictor of transition to a psychotic disorder in ultra-high risk participants. *Schizophr Res*. 2014;153(1-3):25-31. doi:10.1016/j.schres.2014.01.037

28. Amminger GP, Schäfer MR, Schlögelhofer M, Klier CM, McGorry PD. Longer-term outcome in the prevention of psychotic disorders by the Vienna omega-3 study. *Nat Commun*. 2015;6(1):7934. doi:10.1038/ncomms8934

29. An SK, Kang JI, Park JY, Kim KR, Lee SY, Lee E. Attribution bias in ultra-high risk for psychosis and first-episode schizophrenia. *Schizophr Res*. 2010;118(1-3):54-61. doi:10.1016/j.schres.2010.01.025

30. Andreou C, Faber PL, Leicht G, et al. Resting-state connectivity in the prodromal phase of schizophrenia: Insights from EEG microstates. *Schizophr Res*. 2014;152(2-3):513-520. doi:10.1016/j.schres.2013.12.008

31. Armando M, Pontillo M, De Crescenzo F, et al. Twelve-month psychosis-predictive value of the ultra-high risk criteria in children and adolescents. *Schizophr Res*. 2015;169(1-3):186-192. doi:10.1016/j.schres.2015.10.033

32. Aston J, Bull N, Gschwandtner U, et al. First self-perceived signs and symptoms in emerging psychosis compared with depression. *Early Interv Psychiatry*. 2012;6(4):455-459. doi:10.1111/j.1751-7893.2012.00354.x

33. Auther AM, McLaughlin D, Carrión RE, Nagachandran P, Correll CU, Cornblatt BA. Prospective study of cannabis use in adolescents at clinical high risk for psychosis: Impact on conversion to psychosis and functional outcome. *Psychol Med*. 2012;42(12):2485-2497. doi:10.1017/S0033291712000803

34. Auther AM, Cadenhead KS, Carrión RE, et al. Alcohol confounds relationship between cannabis misuse and psychosis conversion in a high-risk sample. *Acta Psychiatr Scand*. 2015;132(1):60-68. doi:10.1111/acps.12382

35. Azis M, Strauss GP, Walker E, Revelle W, Zinbarg R, Mittal V. Factor Analysis of Negative Symptom Items in the Structured Interview for Prodromal Syndromes. *Schizophr Bull*. 2019;45(5):1042-1050. doi:10.1093/schbul/sby177

36. Ballon JS, Dean KA, Cadenhead KS. Obstetrical complications in people at risk for developing schizophrenia. *Schizophr Res*. 2008;98(1-3):307-311. doi:10.1016/j.schres.2007.05.011

37. Bechdolf A, Thompson A, Nelson B, et al. Experience of trauma and conversion to psychosis in an ultra-high-risk (prodromal) group. *Acta Psychiatr Scand*. 2010;121(5):377-384. doi:10.1111/j.1600-0447.2010.01542.x

38. Bechdolf A, Müller H, Stützer H, et al. Rationale and baseline characteristics of PREVENT: a second-generation intervention trial in subjects at-risk (prodromal) of developing first-episode psychosis evaluating cognitive behavior therapy, aripiprazole, and placebo for the prevention of psychosi. *Schizophr Bull*. 2011;37 Suppl 2(Suppl 2):S111-S121. doi:10.1093/schbul/sbr083

39. Bechdolf A, Wood SJ, Nelson B, et al. Amygdala and insula volumes prior to illness onset in bipolar disorder: A magnetic resonance imaging study. *Psychiatry Res - Neuroimaging*. 2012;201(1):34-39. doi:10.1016/j.pscychresns.2011.06.010

40. Beck K, Studerus E, Andreou C, et al. Clinical and functional ultra-long-term outcome of patients with a clinical high risk (CHR) for psychosis. *Eur Psychiatry*. 2019;62:30-37. doi:10.1016/j.eurpsy.2019.08.005

41. Bentley E, Millman ZB, Thompson E, et al. High-risk diagnosis, social stress, and parent-child relationships: A moderation model. *Schizophr Res*. 2016;174(1-3):65-70. doi:10.1016/j.schres.2016.04.014

42. Berger ME, Smesny S, Kim SW, et al. Omega-6 to omega-3 polyunsaturated fatty acid ratio and subsequent mood disorders in young people with at-risk mental states: A 7-year longitudinal study. *Transl Psychiatry*. 2017;7(8):1-6. doi:10.1038/tp.2017.190

43. Bernard JA, Orr JM, Mittal VA. Cerebello-thalamo-cortical networks predict positive symptom progression in individuals at ultra-high risk for psychosis. *NeuroImage Clin*. 2017;14:622-628. doi:10.1016/j.nicl.2017.03.001

44. Bodatsch M, Ruhrmann S, Wagner M, et al. Prediction of psychosis by mismatch negativity. *Biol Psychiatry*. 2011;69(10):959-966. doi:10.1016/j.biopsych.2010.09.057

45. Boldrini T, Pontillo M, Tanzilli A, et al. An attachment perspective on the risk for psychosis: Clinical correlates and the predictive value of attachment patterns and mentalization. *Schizophr Res*. 2020;222:209-217. doi:10.1016/j.schres.2020.05.052

46. Bowie CR, McLaughlin D, Carrión RE, Auther AM, Cornblatt BA. Cognitive changes following antidepressant or antipsychotic treatment in adolescents at clinical risk for psychosis. *Schizophr Res*. 2012;137(1-3):110-117. doi:10.1016/j.schres.2012.02.008

47. Brandizzi M, Valmaggia L, Byrne M, et al. Predictors of functional outcome in individuals at high clinical risk for psychosis at six years follow-up. *J Psychiatr Res*. 2015;65:115-123. doi:10.1016/j.jpsychires.2015.03.005

48. Brewer WJ, Francey SM, Wood SJ, et al. Memory impairments identified in people at ultra-high risk for psychosis who later develop first-episode psychosis. *Am J Psychiatry*. 2005;162(1):71-78. doi:10.1176/appi.ajp.162.1.71

49. Bright M, Parker S, French P, et al. Metacognitive beliefs as psychological predictors of social functioning: An investigation with young people at risk of psychosis. *Psychiatry Res*. 2018;262:520-526. doi:10.1016/j.psychres.2017.09.037

50. Broome MR, Woolley JB, Johns LC, et al. Outreach and support in south London (OASIS): Implementation of a clinical service for prodromal psychosis and the at risk mental state. *Eur Psychiatry*. 2005;20(5-6):372-378. doi:10.1016/j.eurpsy.2005.03.001

51. Brucato G, Masucci MD, Arndt LY, et al. Baseline demographics, clinical features and predictors of conversion among 200 individuals in a longitudinal prospective psychosis-risk cohort. *Psychol Med*. 2017;47(11):1923-1935. doi:10.1017/S0033291717000319

52. Brucato G, Appelbaum PS, Masucci MD, et al. Prevalence and phenomenology of violent ideation and behavior among 200 young people at clinical high-risk for psychosis: an emerging model of violence and psychotic illness. *Neuropsychopharmacology*. 2019;44(5):907-914. doi:10.1038/s41386-018-0304-5

53. Brucato G, First MB, Dishy GA, et al. Recency and intensification of positive symptoms enhance prediction of conversion to syndromal psychosis in clinical high-risk patients. *Psychol Med*. 2021;51(1):112-120. doi:10.1017/S0033291719003040

54. Buchy L, Perkins D, Woods SW, Liu L, Addington J. Impact of substance use on conversion to psychosis in youth at clinical high risk of psychosis. *Schizophr Res*. 2014;156(2-3):277-280. doi:10.1016/j.schres.2014.04.021

55. Buchy L, Cadenhead KS, Cannon TD, et al. Substance use in individuals at clinical high risk of psychosis. *Psychol Med*. 2015;45(11):2275-2284. doi:10.1017/S0033291715000227

56. Carol EE, Mittal VA. Resting cortisol level, self-concept, and putative familial environment in adolescents at ultra high-risk for psychotic disorders. *Psychoneuroendocrinology*. 2015;57:26-36. doi:10.1016/j.psyneuen.2015.03.018

57. Carol EE, Spencer RL, Mittal VA. Sex differences in morning cortisol in youth at ultra-high-risk for psychosis. *Psychoneuroendocrinology*. 2016;72:87-93. doi:10.1016/j.psyneuen.2016.06.013

58. Carol EE, Spencer RL, Mittal VA. The relationship between cannabis use and cortisol levels in youth at ultra high-risk for psychosis. *Psychoneuroendocrinology*. 2017;83:58-64. doi:10.1016/j.psyneuen.2017.04.017

59. Carr V, Halpin S, Lau N, O’brien S, Beckmann J, Lewin T. A Risk Factor Screening and Assessment Protocol for Schizophrenia and Related Psychosis. *Aust N Z J Psychiatry*. 2000;34(2_suppl):S170-S180. doi:10.1080/000486700240

60. Carrión RE, McLaughlin D, Goldberg TE, et al. Prediction of functional outcome in individuals at clinical high risk for psychosis. *JAMA Psychiatry*. 2013;70(11):1133-1142. doi:10.1001/jamapsychiatry.2013.1909

61. Carrión RE, McLaughlin D, Auther AM, Olsen R, Correll CU, Cornblatt BA. The impact of psychosis on the course of cognition: A prospective, nested case-control study in individuals at clinical high-risk for psychosis. *Psychol Med*. 2015;45(15):3341-3354. doi:10.1017/S0033291715001233

62. Carrión RE, Demmin D, Auther AM, et al. Duration of attenuated positive and negative symptoms in individuals at clinical high risk: Associations with risk of conversion to psychosis and functional outcome. *J Psychiatr Res*. 2016;81:95-101. doi:10.1016/j.jpsychires.2016.06.021

63. Carrión RE, Correll CU, Auther AM, Cornblatt BA. A severity-based clinical staging model for the psychosis prodrome: Longitudinal findings from the New York recognition and prevention program. *Schizophr Bull*. 2017;43(1):64-74. doi:10.1093/schbul/sbw155

64. Carrión RE, Walder DJ, Auther AM, et al. From the psychosis prodrome to the first-episode of psychosis: No evidence of a cognitive decline. *J Psychiatr Res*. 2018;96:231-238. doi:10.1016/j.jpsychires.2017.10.014

65. Carrión RE, Auther AM, McLaughlin D, et al. Social decline in the psychosis prodrome: Predictor potential and heterogeneity of outcome. *Schizophr Res*. 2021;227:44-51. doi:10.1016/j.schres.2020.09.006

66. Chung YC, Jung HY, Kim SW, et al. What factors are related to delayed treatment in individuals at high risk for psychosis? *Early Interv Psychiatry*. 2010;4(2):124-131. doi:10.1111/j.1751-7893.2010.00170.x

67. Clamor A, Sundag J, Lincoln TM. Specificity of resting-state heart rate variability in psychosis: A comparison with clinical high risk, anxiety, and healthy controls. *Schizophr Res*. 2019;206:89-95. doi:10.1016/j.schres.2018.12.009

68. Cocchi A, Meneghelli A, Preti A. Programma 2000: Celebrating 10 years of activity of an Italian pilot programme on early intervention in psychosis. *Aust N Z J Psychiatry*. 2008;42(12):1003-1012. doi:10.1080/00048670802512032

69. Cocchi A, Lora A, Meneghelli A, et al. Sex differences in first-episode psychosis and in people at ultra-high risk. *Psychiatry Res*. 2014;215(2):314-322. doi:10.1016/j.psychres.2013.11.023

70. Comparelli A, Corigliano V, De Carolis A, et al. Anomalous self-experiences and their relationship with symptoms, neuro-cognition, and functioning in at-risk adolescents and young adults. *Compr Psychiatry*. 2016;65:44-49. doi:10.1016/j.comppsych.2015.09.011

71. Conrad AM, Lewin TJ, Sly KA, et al. Ten-year audit of clients presenting to a specialised service for young people experiencing or at increased risk for psychosis. *BMC Psychiatry*. 2014;14(1):318. doi:10.1186/s12888-014-0318-4

72. Conrad AM, Lewin TJ, Sly KA, et al. Utility of risk-status for predicting psychosis and related outcomes: evaluation of a 10-year cohort of presenters to a specialised early psychosis community mental health service. *Psychiatry Res*. 2017;247:336-344. doi:10.1016/j.psychres.2016.12.005

73. Corcoran CM, Kimhy D, Stanford A, et al. Temporal association of cannabis use with symptoms in individuals at clinical high risk for psychosis. *Schizophr Res*. 2008;106(2-3):286-293. doi:10.1016/j.schres.2008.08.008

74. Corcoran CM, Kimhy D, Parrilla-Escobar MA, et al. The relationship of social function to depressive and negative symptoms in individuals at clinical high risk for psychosis. *Psychol Med*. 2011;41(2):251-261. doi:10.1017/S0033291710000802

75. Cornblatt BA, Lencz T, Smith CW, et al. Can antidepressants be used to treat the schizophrenia prodrome? Results of a prospective, naturalistic treatment study of adolescents. *J Clin Psychiatry*. 2007;68(4):546-557. doi:10.4088/JCP.v68n0410

76. Cornblatt BA, Carrión RE, Addington J, et al. Risk factors for psychosis: Impaired social and role functioning. *Schizophr Bull*. 2012;38(6):1247-1257. doi:10.1093/schbul/sbr136

77. Cornblatt BA, Carrión RE, Auther A, et al. Psychosis prevention: A modified clinical high risk perspective from the recognition and prevention (RAP) Program. *Am J Psychiatry*. 2015;172(10):986-994. doi:10.1176/appi.ajp.2015.13121686

78. Cotter J, Lin A, Drake RJ, et al. Long-term employment among people at ultra-high risk for psychosis. *Schizophr Res*. 2017;184:26-31. doi:10.1016/j.schres.2016.11.033

79. Cowan HR, McAdams DP, Mittal VA. Core beliefs in healthy youth and youth at ultra high-risk for psychosis: Dimensionality and links to depression, anxiety, and attenuated psychotic symptoms. *Dev Psychopathol*. 2018;31(1):1-14. doi:10.1017/S0954579417001912

80. Damme KSF, Ristanovic I, Vargas T, Mittal VA. Timing of menarche and abnormal hippocampal connectivity in youth at clinical-high risk for psychosis. *Psychoneuroendocrinology*. 2020;117:104672. doi:10.1016/j.psyneuen.2020.104672

81. Dandash O, Fornito A, Lee J, et al. Altered striatal functional connectivity in subjects with an at-risk mental state for psychosis. *Schizophr Bull*. 2014;40(4):904-913. doi:10.1093/schbul/sbt093

82. de Bock R, Mackintosh AJ, Maier F, Borgwardt S, Riecher-Rössler A, Andreou C. EEG microstates as biomarker for psychosis in ultra-high-risk patients. *Transl Psychiatry*. 2020;10(1):300. doi:10.1038/s41398-020-00963-7

83. de Vos C, Thompson A, Amminger P, et al. The relationship between childhood trauma and clinical characteristics in ultra-high risk for psychosis youth. *Psychosis*. 2019;11(1):28-41. doi:10.1080/17522439.2019.1582686

84. De Wit S, Schothorst PF, Oranje B, Ziermans TB, Durston S, Kahn RS. Adolescents at ultra-high risk for psychosis: Long-term outcome of individuals who recover from their at-risk state. *Eur Neuropsychopharmacol*. 2014;24(6):865-873. doi:10.1016/j.euroneuro.2014.02.008

85. Dean DJ, Bernard JA, Orr JM, et al. Cerebellar morphology and procedural learning impairment in neuroleptic-naive youth at ultrahigh risk of psychosis. *Clin Psychol Sci*. 2014;2(2):152-164. doi:10.1177/2167702613500039

86. Dean DJ, Orr JM, Bernard JA, et al. Hippocampal Shape Abnormalities Predict Symptom Progression in Neuroleptic-Free Youth at Ultrahigh Risk for Psychosis. *Schizophr Bull*. 2016;42(1):161-169. doi:10.1093/schbul/sbv086

87. Dolz M, Tor J, De la Serna E, et al. Characterization of children and adolescents with psychosis risk syndrome: The Children and Adolescents Psychosis Risk Syndrome (CAPRIS) study. *Early Interv Psychiatry*. 2019;13(5):1062-1072. doi:10.1111/eip.12728

88. Dragt S, Nieman DH, Becker HE, et al. Age of onset of cannabis use is associated with age of onset of high-risk symptoms for psychosis. *Can J Psychiatry*. 2010;55(3):165-171. doi:10.1177/070674371005500308

89. Dragt S, Nieman DH, Schultze-Lutter F, et al. Cannabis use and age at onset of symptoms in subjects at clinical high risk for psychosis. *Acta Psychiatr Scand*. 2012;125(1):45-53. doi:10.1111/j.1600-0447.2011.01763.x

90. Eastvold AD, Heaton RK, Cadenhead KS. Neurocognitive deficits in the (putative) prodrome and first episode of psychosis. *Schizophr Res*. 2007;93(1-3):266-277. doi:10.1016/j.schres.2007.03.013

91. Epstein KA, Cullen KR, Mueller BA, Robinson P, Lee S, Kumra S. White matter abnormalities and cognitive impairment in early-onset schizophrenia-spectrum disorders. *J Am Acad Child Adolesc Psychiatry*. 2014;53(3):362-72.e1-2. doi:10.1016/j.jaac.2013.12.007

92. Falkenberg I, Valmaggia L, Byrnes M, et al. Why are help-seeking subjects at ultra-high risk for psychosis help-seeking? *Psychiatry Res*. 2015;228(3):808-815. doi:https://doi.org/10.1016/j.psychres.2015.05.018

93. Flückiger R, Ruhrmann S, Debbané M, et al. Psychosis-predictive value of self-reported schizotypy in a clinical high-risk sample. *J Abnorm Psychol*. 2016;125(7):923-932. doi:10.1037/abn0000192

94. Fontenelle LF, Lin A, Pantelis C, Wood SJ, Nelson B, Yung AR. A longitudinal study of obsessive-compulsive disorder in individuals at ultra-high risk for psychosis. *J Psychiatr Res*. 2011;45(9):1140-1145. doi:10.1016/j.jpsychires.2011.03.005

95. Fontenelle LF, Lin A, Pantelis C, Wood SJ, Nelson B, Yung AR. Markers of vulnerability to obsessive-compulsive disorder in an ultra-high risk sample of patients who developed psychosis. *Early Interv Psychiatry*. 2012;6(2):201-206. doi:10.1111/j.1751-7893.2012.00357.x

96. Foss-Feig JH, Velthorst E, Smith L, et al. Clinical Profiles and Conversion Rates Among Young Individuals With Autism Spectrum Disorder Who Present to Clinical High Risk for Psychosis Services. *J Am Acad Child Adolesc Psychiatry*. 2019;58(6):582-588. doi:10.1016/j.jaac.2018.09.446

97. Francesconi M, Minichino A, Carrión RE, et al. Psychosis prediction in secondary mental health services. A broad, comprehensive approach to the “at risk mental state” syndrome. *Eur Psychiatry*. 2017;40:96-104. doi:10.1016/j.eurpsy.2016.09.002

98. Francey SM, Jackson HJ, Phillips LJ, Wood SJ, Yung AR, McGorry PD. Sustained attention in young people at high risk of psychosis does not predict transition to psychosis. In: *Schizophrenia Research*. Vol 79. Elsevier; 2005:127-136. doi:10.1016/j.schres.2005.06.023

99. Fusar-Poli P, Byrne M, Badger S, Valmaggia LR, McGuire PK. Outreach and support in South London (OASIS), 2001-2011: Ten years of early diagnosis and treatment for young individuals at high clinical risk for psychosis. *Eur Psychiatry*. 2013;28(5):315-326. doi:10.1016/j.eurpsy.2012.08.002

100. Fusar-Poli P, Frascarelli M, Valmaggia L, et al. Antidepressant, antipsychotic and psychological interventions in subjects at high clinical risk for psychosis: OASIS 6-year naturalistic study. *Psychol Med*. 2015;45(6):1327-1339. doi:10.1017/S003329171400244X

101. Fusar-Poli P, Cappucciati M, De Micheli A, et al. Diagnostic and prognostic significance of brief limited intermittent psychotic symptoms (BLIPS) in individuals at ultra high risk. *Schizophr Bull*. 2017;43(1):48-56. doi:10.1093/schbul/sbw151

102. Fusar-Poli P, Spencer T, De Micheli A, Curzi V, Nandha S, McGuire P. Outreach and support in South-London (OASIS) 2001-2020: Twenty years of early detection, prognosis and preventive care for young people at risk of psychosis. *Eur Neuropsychopharmacol*. 2020;39:111-122. doi:10.1016/j.euroneuro.2020.08.002

103. Garner B, Pariante CM, Wood SJ, et al. Pituitary volume predicts future transition to psychosis in individuals at ultra-high risk of developing psychosis. *Biol Psychiatry*. 2005;58(5):417-423. doi:10.1016/j.biopsych.2005.04.018

104. Gaspar PA, Castillo RI, Maturana A, et al. Early psychosis detection program in Chile: A first step for the South American challenge in psychosis research. *Early Interv Psychiatry*. 2019;13(2):328-334. doi:10.1111/eip.12766

105. Gerstenberg M, Theodoridou A, Traber-Walker N, et al. Adolescents and adults at clinical high-risk for psychosis: Age-related differences in attenuated positive symptoms syndrome prevalence and entanglement with basic symptoms. *Psychol Med*. 2016;46(5):1069-1078. doi:10.1017/S0033291715002627

106. Gill KE, Evans E, Kayser J, et al. Smell identification in individuals at clinical high risk for schizophrenia. *Psychiatry Res*. 2014;220(1-2):201-204. doi:10.1016/j.psychres.2014.07.018

107. Girgis RR, Slifstein M, Brucato G, et al. Imaging synaptic dopamine availability in individuals at clinical high-risk for psychosis: a [11C]-(+)-PHNO PET with methylphenidate challenge study. *Mol Psychiatry*. 2021;26(6):2504-2513. doi:10.1038/s41380-020-00934-w

108. Girgis RR, Basavaraju R, France J, et al. An exploratory magnetic resonance imaging study of suicidal ideation in individuals at clinical high-risk for psychosis. *Psychiatry Res - Neuroimaging*. 2021;312:111287. doi:10.1016/j.pscychresns.2021.111287

109. Gisselgård J, Lebedev A V., Kurz KD, Joa I, Johannessen JO, Brønnick K. Structural and functional alterations in the brain during working memory in medication-naïve patients at clinical high-risk for psychosis. van Amelsvoort T, ed. *PLoS One*. 2018;13(5):e0196289. doi:10.1371/journal.pone.0196289

110. Glenthøj LB, Jepsen JRM, Hjorthøj C, et al. Negative symptoms mediate the relationship between neurocognition and function in individuals at ultrahigh risk for psychosis. *Acta Psychiatr Scand*. 2017;135(3):250-258. doi:10.1111/acps.12682

111. Glenthøj LB, Mariegaard LS, Fagerlund B, et al. Cognitive remediation plus standard treatment versus standard treatment alone for individuals at ultra-high risk of developing psychosis: Results of the FOCUS randomised clinical trial. *Schizophr Res*. 2020;224:151-158. doi:https://doi.org/10.1016/j.schres.2020.08.016

112. Glenthøj LB, Kristensen TD, Wenneberg C, Hjorthøj C, Nordentoft M. Experiential negative symptoms are more predictive of real-life functional outcome than expressive negative symptoms in clinical high-risk states. *Schizophr Res*. 2020;218:151-156. doi:https://doi.org/10.1016/j.schres.2020.01.012

113. Graber K, Bosquet Enlow M, Duffy FH, et al. P300 amplitude attenuation in high risk and early onset psychosis youth. *Schizophr Res*. 2019;210:228-238. doi:10.1016/j.schres.2018.12.029

114. Granö N, Karjalainen M, Edlund V, et al. Changes in depression, anxiety and hopelessness symptoms during family- and community-oriented intervention for help-seeking adolescents and adolescents at risk of psychosis. *Nord J Psychiatry*. 2014;68(2):93-99. doi:10.3109/08039488.2013.768294

115. Grent-’t-Jong T, Gajwani R, Gross J, et al. Association of magnetoencephalographically measured high-frequency oscillations in visual cortex with circuit dysfunctions in local and large-scale networks during emerging psychosis. *JAMA Psychiatry*. 2020;77(8):852-862. doi:10.1001/jamapsychiatry.2020.0284

116. Grent-‘t-Jong T, Gajwani R, Gross J, et al. 40-Hz Auditory Steady-State Responses Characterize Circuit Dysfunctions and Predict Clinical Outcomes in Clinical High-Risk for Psychosis Participants: A Magnetoencephalography Study. *Biol Psychiatry*. 2021;90(6):419-429. doi:10.1016/j.biopsych.2021.03.018

117. Grivel MM, Leong W, Masucci MD, et al. Impact of lifetime traumatic experiences on suicidality and likelihood of conversion in a cohort of individuals at clinical high-risk for psychosis. *Schizophr Res*. 2018;195:549-553. doi:10.1016/j.schres.2017.09.006

118. Gupta T, Cowan HR, Strauss GP, Walker EF, Mittal VA. Deconstructing Negative Symptoms in Individuals at Clinical High-Risk for Psychosis: Evidence for Volitional and Diminished Emotionality Subgroups That Predict Clinical Presentation and Functional Outcome. *Schizophr Bull*. 2021;47(1):54-63. doi:10.1093/schbul/sbaa084

119. Haining K, Matrunola C, Mitchell L, et al. Neuropsychological deficits in participants at clinical high risk for psychosis recruited from the community: Relationships to functioning and clinical symptoms. *Psychol Med*. 2020;50(1):77-85. doi:10.1017/S0033291718003975

120. Haroun N, Dunn L, Haroun A, Cadenhead KS. Risk and protection in prodromal schizophrenia: Ethical implications for clinical practice and future research. *Schizophr Bull*. 2006;32(1):166-178. doi:10.1093/schbul/sbj007

121. Hartmann JA, Yuen HP, McGorry PD, et al. Declining transition rates to psychotic disorder in “ultra-high risk” clients: Investigation of a dilution effect. *Schizophr Res*. 2016;170(1):130-136. doi:10.1016/j.schres.2015.11.026

122. Hengartner MP, Heekeren K, Dvorsky D, Rössler W, Theodoridou A, Walitza S. Checking the predictive accuracy of basic symptoms against ultra high-risk criteria and testing of a multivariable prediction model: Evidence from a prospective three-year observational study of persons at clinical high-risk for psychosis. *Eur Psychiatry*. 2017;45:27-35. doi:10.1016/j.eurpsy.2017.05.026

123. Hirt V, Schubring D, Schalinski I, Rockstroh B. Mismatch negativity and cognitive performance in the course of schizophrenia. *Int J Psychophysiol*. 2019;145:30-39. doi:10.1016/j.ijpsycho.2019.01.006

124. Hirt V, Schalinski I, Rockstroh B. Decoding the impact of adverse childhood experiences on the progression of schizophrenia. *Ment Heal Prev*. 2019;13:82-91. doi:10.1016/j.mhp.2019.01.002

125. Hubl D, Schultze-Lutter F, Hauf M, et al. Striatal cerebral blood flow, executive functioning, and fronto-striatal functional connectivity in clinical high risk for psychosis. *Schizophr Res*. 2018;201:231-236. doi:10.1016/j.schres.2018.06.018

126. Hui C, Morcillo C, Russo DA, et al. Psychiatric morbidity, functioning and quality of life in young people at clinical high risk for psychosis. *Schizophr Res*. 2013;148(1-3):175-180. doi:10.1016/j.schres.2013.05.026

127. Ilonen T, Heinimaa M, Korkeila J, Svirskis T, Salokangas RKR. Differentiating adolescents at clinical high risk for psychosis from psychotic and non-psychotic patients with the Rorschach. *Psychiatry Res*. 2010;179(2):151-156. doi:10.1016/j.psychres.2009.04.011

128. Ising HK, Ruhrmann S, Burger NAFM, et al. Development of a stage-dependent prognostic model to predict psychosis in ultra-high-risk patients seeking treatment for co-morbid psychiatric disorders. *Psychol Med*. 2016;46(9):1839-1851. doi:10.1017/S0033291716000325

129. Janssen H, Maat A, Slot MIE, Scheepers F. Efficacy of psychological interventions in young individuals at ultra-high risk for psychosis: A naturalistic study. *Early Interv Psychiatry*. 2021;15(4):1019-1027. doi:10.1111/eip.13048

130. Jeffries CD, Perkins DO, Fournier M, et al. Networks of blood proteins in the neuroimmunology of schizophrenia. *Transl Psychiatry*. 2018;8(1):112. doi:10.1038/s41398-018-0158-y

131. Jhung K, Park JY, Song YY, Kang JI, Lee E, An SK. Experiential pleasure deficits in the prodrome: A study of emotional experiences in individuals at ultra-high risk for psychosis and recent-onset schizophrenia. *Compr Psychiatry*. 2016;68:209-216. doi:10.1016/j.comppsych.2016.04.021

132. Jung WH, Kim JS, Jang JH, et al. Cortical thickness reduction in individuals at ultra-high-risk for psychosis. *Schizophr Bull*. 2011;37(4):839-849. doi:10.1093/schbul/sbp151

133. Kang MJ, Bang M, Lee SY, Lee E, Yoo SW, An SK. Coping styles in individuals at ultra-high risk for psychosis: Associations with cognitive appraisals. *Psychiatry Res*. 2018;264:162-168. doi:10.1016/j.psychres.2018.03.079

134. Karlsgodt KH, Niendam TA, Bearden CE, Cannon TD. White Matter Integrity and Prediction of Social and Role Functioning in Subjects at Ultra-High Risk for Psychosis. *Biol Psychiatry*. 2009;66(6):562-569. doi:10.1016/j.biopsych.2009.03.013

135. Karlsgodt KH, van Erp TGM, Bearden CE, Cannon TD. Altered relationships between age and functional brain activation in adolescents at clinical high risk for psychosis. *Psychiatry Res - Neuroimaging*. 2014;221(1):21-29. doi:10.1016/j.pscychresns.2013.08.004

136. Kennedy L, Brucato G, Lundgren B, et al. Thematic content of obsessive and compulsive symptoms and conversion to psychosis in a clinical high-risk cohort. *Early Interv Psychiatry*. 2021;15(5):1423-1428. doi:10.1111/eip.13057

137. Kim KR, Park JY, Song DH, Koo HK, An SK. Neurocognitive performance in subjects at ultrahigh risk for schizophrenia: A comparison with first-episode schizophrenia. *Compr Psychiatry*. 2011;52(1):33-40. doi:10.1016/j.comppsych.2010.04.010

138. Kim E, Jang JH, Park HY, et al. Pharmacotherapy and clinical characteristics of ultra-high-risk for psychosis according to conversion status: A naturalistic observational study. *Early Interv Psychiatry*. 2012;6(1):30-37. doi:10.1111/j.1751-7893.2011.00295.x

139. Kim KR, Song YY, Park JY, et al. The relationship between psychosocial functioning and resilience and negative symptoms in individuals at ultra-high risk for psychosis. *Aust N Z J Psychiatry*. 2013;47(8):762-771. doi:10.1177/0004867413488218

140. Kindler J, Schultze-Lutter F, Michel C, et al. Abnormal involuntary movements are linked to psychosis-risk in children and adolescents: Results of a population-based study. *Schizophr Res*. 2016;174(1-3):58-64. doi:10.1016/j.schres.2016.04.032

141. Kindler J, Schultze-Lutter F, Hauf M, et al. Increased Striatal and Reduced Prefrontal Cerebral Blood Flow in Clinical High Risk for Psychosis. *Schizophr Bull*. 2018;44(1):182-192. doi:10.1093/schbul/sbx070

142. Klauser P, Zhou J, Lim JKW, et al. Lack of Evidence for Regional Brain Volume or Cortical Thickness Abnormalities in Youths at Clinical High Risk for Psychosis: Findings from the Longitudinal Youth at Risk Study. *Schizophr Bull*. 2015;41(6):1285-1293. doi:10.1093/schbul/sbv012

143. Kline ER, Seidman LJ, Cornblatt BA, et al. Depression and clinical high-risk states: Baseline presentation of depressed vs. non-depressed participants in the NAPLS-2 cohort. *Schizophr Res*. 2018;192:357-363. doi:10.1016/j.schres.2017.05.032

144. Klosterkötter J, Hellmich M, Steinmeyer EM, Schultze-Lutter F. Diagnosing schizophrenia in the initial prodromal phase. *Arch Gen Psychiatry*. 2001;58(2):158-164. doi:10.1001/archpsyc.58.2.158

145. Kollias C, Xenaki LA, Dimitrakopoulos S, et al. Early psychosis intervention outpatient service of the 1st Psychiatric University Clinic in Athens: 3 Years of experience. *Early Interv Psychiatry*. 2018;12(3):491-496. doi:10.1111/eip.12407

146. Koren D, Scheyer R, Stern Y, et al. Metacognition strengthens the association between neurocognition and attenuated psychosis syndrome: Preliminary evidence from a pilot study among treatment-seeking versus healthy adolescents. *Schizophr Res*. 2019;210:207-214. doi:10.1016/j.schres.2018.12.036

147. Korkeila J, Salokangas RKR, Heinimaaa M, et al. Physical illnesses, developmental risk factors and psychiatric diagnoses among subjects at risk of psychosis. *Eur Psychiatry*. 2013;28(3):135-140. doi:10.1016/j.eurpsy.2011.06.005

148. Kotlicka-Antczak M, Pawełczyk A, Rabe-Jabłońska J, Śmigielski J, Pawełczyk T. Obstetrical complications and Apgar score in subjects at risk of psychosis. *J Psychiatr Res*. 2014;48(1):79-85. doi:10.1016/j.jpsychires.2013.10.004

149. Kotlicka-Antczak M, Pawełczyk A, Karbownik MS, et al. Deficits in the identification of pleasant odors predict the transition of an at-risk mental state to psychosis. *Schizophr Res*. 2017;181:49-54. doi:10.1016/j.schres.2016.10.019

150. Kotlicka-Antczak M, Pawełczyk T, Podgórski M, Żurner N, Karbownik MS, Pawełczyk A. Polish individuals with an at-risk mental state: demographic and clinical characteristics. *Early Interv Psychiatry*. 2018;12(3):391-399. doi:10.1111/eip.12333

151. Kotlicka-Antczak M, Karbownik MS, Stawiski K, et al. Short clinically-based prediction model to forecast transition to psychosis in individuals at clinical high risk state. *Eur Psychiatry*. 2019;58:72-79. doi:10.1016/j.eurpsy.2019.02.007

152. Koutsouleris N, Gaser C, Bottlender R, et al. Use of neuroanatomical pattern regression to predict the structural brain dynamics of vulnerability and transition to psychosis. *Schizophr Res*. 2010;123(2-3):175-187. doi:10.1016/j.schres.2010.08.032

153. Koutsouleris N, Davatzikos C, Bottlender R, et al. Early recognition and disease prediction in the at-risk mental states for psychosis using neurocognitive pattern classification. *Schizophr Bull*. 2012;38(6):1200-1215. doi:10.1093/schbul/sbr037

154. Koutsouleris N, Gaser C, Patschurek-Kliche K, et al. Multivariate patterns of brain-cognition associations relating to vulnerability and clinical outcome in the at-risk mental states for psychosis. *Hum Brain Mapp*. 2012;33(9):2104-2124. doi:10.1002/hbm.21342

155. Koutsouleris N, Dwyer DB, Degenhardt F, et al. Multimodal Machine Learning Workflows for Prediction of Psychosis in Patients with Clinical High-Risk Syndromes and Recent-Onset Depression. *JAMA Psychiatry*. 2021;78(2):195-209. doi:10.1001/jamapsychiatry.2020.3604

156. Kraan TC, Velthorst E, Themmen M, et al. Child Maltreatment and Clinical Outcome in Individuals at Ultra-High Risk for Psychosis in the EU-GEI High Risk Study. *Schizophr Bull*. 2018;44(3):584-592. doi:10.1093/schbul/sbw162

157. Kristensen TD, Mandl RCW, Raghava JM, et al. Widespread higher fractional anisotropy associates to better cognitive functions in individuals at ultra-high risk for psychosis. *Hum Brain Mapp*. 2019;40(18):5185-5201. doi:10.1002/hbm.24765

158. Kwak Y Bin, Kim M, Cho KIK, Lee J, Lee TY, Kwon JS. Reduced cortical thickness in subjects at clinical high risk for psychosis and clinical attributes. *Aust N Z J Psychiatry*. 2019;53(3):219-227. doi:10.1177/0004867418807299

159. Lam MML, Hung S-F, Chen EYH. Transition to Psychosis: 6-Month follow-up of a Chinese High-Risk Group in Hong Kong. *Aust New Zeal J Psychiatry*. 2006;40(5):414-420. doi:10.1080/j.1440-1614.2006.01817.x

160. Lam M, Abdul Rashid NA, Lee SA, et al. Baseline social amotivation predicts 1-year functioning in UHR subjects: A validation and prospective investigation. *Eur Neuropsychopharmacol*. 2015;25(12):2187-2196. doi:10.1016/j.euroneuro.2015.10.007

161. Leanza L, Studerus E, Mackintosh AJ, et al. Predictors of study drop-out and service disengagement in patients at clinical high risk for psychosis. *Soc Psychiatry Psychiatr Epidemiol*. 2020;55(5):539-548. doi:10.1007/s00127-019-01796-6

162. Lee J, Rekhi G, Mitter N, et al. The Longitudinal Youth at Risk Study (LYRIKS) - An Asian UHR perspective. *Schizophr Res*. 2013;151(1-3):279-283. doi:10.1016/j.schres.2013.09.025

163. Lee SJ, Kim KR, Lee SY, An SK. Impaired social and role function in ultra-high risk for psychosis and first-episode schizophrenia: Its relations with negative symptoms. *Psychiatry Investig*. 2017;14(5):539-545. doi:10.4306/pi.2017.14.5.539

164. Leicht G, Vauth S, Polomac N, et al. EEG-Informed fMRI Reveals a Disturbed Gamma-Band-Specific Network in Subjects at High Risk for Psychosis. *Schizophr Bull*. 2016;42(1):239-249. doi:10.1093/schbul/sbv092

165. Lencz T, Smith CW, Auther A, Correll CU, Cornblatt B. Nonspecific and attenuated negative symptoms in patients at clinical high-risk for schizophrenia. *Schizophr Res*. 2004;68(1):37-48. doi:10.1016/S0920-9964(03)00214-7

166. Li H, Zhang TH, Xu LH, et al. A comparison of conversion rates, clinical profiles and predictors of outcomes in two independent samples of individuals at clinical high risk for psychosis in China. *Schizophr Res*. 2018;197:509-515. doi:10.1016/j.schres.2017.11.029

167. Lim J, Rekhi G, Rapisarda A, et al. Impact of psychiatric comorbidity in individuals at Ultra High Risk of psychosis — Findings from the Longitudinal Youth at Risk Study (LYRIKS). *Schizophr Res*. 2015;164(1):8-14. doi:https://doi.org/10.1016/j.schres.2015.03.007

168. Lim KO, Lee TY, Kim M, et al. Early referral and comorbidity as possible causes of the declining transition rate in subjects at clinical high risk for psychosis. *Early Interv Psychiatry*. 2018;12(4):596-604. doi:10.1111/eip.12363

169. Lin A, Wood SJ, Nelson B, Beavan A, McGorry P, Yung AR. Outcomes of nontransitioned cases in a sample at ultra-high risk for psychosis. *Am J Psychiatry*. 2015;172(3):249-258. doi:10.1176/appi.ajp.2014.13030418

170. Lincoln SH, Hooker CIL. Neural structure and social dysfunction in individuals at clinical high risk for psychosis. *Psychiatry Res - Neuroimaging*. 2014;224(3):152-158. doi:10.1016/j.pscychresns.2014.08.008

171. Lincoln TM, Sundag J, Schlier B, Karow A. The Relevance of Emotion Regulation in Explaining Why Social Exclusion Triggers Paranoia in Individuals at Clinical High Risk of Psychosis. *Schizophr Bull*. 2018;44(4):757-767. doi:10.1093/schbul/sbx135

172. Lindgren M, Manninen M, Kalska H, et al. Predicting psychosis in a general adolescent psychiatric sample. *Schizophr Res*. 2014;158(1-3):1-6. doi:10.1016/j.schres.2014.06.028

173. Lindgren M, Manninen M, Kalska H, et al. Suicidality, self-harm and psychotic-like symptoms in a general adolescent psychiatric sample. *Early Interv Psychiatry*. 2017;11(2):113-122. doi:10.1111/eip.12218

174. Lo Cascio N, Saba R, Hauser M, et al. Attenuated psychotic and basic symptom characteristics in adolescents with ultra-high risk criteria for psychosis, other non-psychotic psychiatric disorders and early-onset psychosis. *Eur Child Adolesc Psychiatry*. 2016;25(10):1091-1102. doi:10.1007/s00787-016-0832-7

175. Lo Cascio N, Curto M, Pasqualetti P, et al. Impairment in Social Functioning differentiates youth meeting Ultra-High Risk for psychosis criteria from other mental health help-seekers: A validation of the Italian version of the Global Functioning: Social and Global Functioning: Role scales. *Psychiatry Res*. 2017;253:296-302. doi:10.1016/j.psychres.2017.04.008

176. Loewy RL, Corey S, Amirfathi F, et al. Childhood trauma and clinical high risk for psychosis. *Schizophr Res*. 2019;205:10-14. doi:10.1016/j.schres.2018.05.003

177. MacHielsen M, Van Der Sluis S, De Haan L. Cannabis use in patients with a first psychotic episode and subjects at ultra high risk of psychosis: Impact on psychotic- and pre-psychotic symptoms. *Aust N Z J Psychiatry*. 2010;44(8):721-728. doi:10.3109/00048671003689710

178. Madsen HK, Nordholm D, Krakauer K, Randers L, Nordentoft M. Psychopathology and social functioning of 42 subjects from a Danish ultra high-risk cohort. *Early Interv Psychiatry*. 2018;12(6):1181-1187. doi:10.1111/eip.12438

179. Manninen M, Lindgren M, Therman S, et al. Clinical high-risk state does not predict later psychosis in a delinquent adolescent population. *Early Interv Psychiatry*. 2014;8(1):87-90. doi:10.1111/eip.12045

180. Marshall C, Addington J, Epstein I, Liu L, Deighton S, Zipursky RB. Treating young individuals at clinical high risk for psychosis. *Early Interv Psychiatry*. 2012;6(1):60-68. doi:10.1111/j.1751-7893.2011.00299.x

181. Mason O, Startup M, Halpin S, Schall U, Conrad A, Carr V. Risk factors for transition to first episode psychosis among individuals with “at-risk mental states.” *Schizophr Res*. 2004;71(2-3):227-237. doi:10.1016/j.schres.2004.04.006

182. Matsumoto K, Katsura M, Tsujino N, et al. Federated multi-site longitudinal study of at-risk mental state for psychosis in Japan. *Schizophr Res*. 2019;204:343-352. doi:10.1016/j.schres.2018.09.001

183. Mazzoni P, Kimhy D, Khan S, et al. Childhood onset diagnoses in a case series of teens at clinical high risk for psychosis. *J Child Adolesc Psychopharmacol*. 2009;19(6):771-776. doi:10.1089/cap.2008.0105

184. McAusland L, Buchy L, Cadenhead KS, et al. Anxiety in youth at clinical high risk for psychosis. *Early Interv Psychiatry*. 2017;11(6):480-487. doi:10.1111/eip.12274

185. McDonald M, Christoforidou E, Van Rijsbergen N, et al. Using online screening in the general population to detect participants at clinical high-risk for psychosis. *Schizophr Bull*. 2019;45(3):600-609. doi:10.1093/schbul/sby069

186. McFarlane WR, Cook WL, Downing D, et al. Early Detection, Intervention, and Prevention of Psychosis Program: Rationale, Design, and Sample Description. *Adolesc Psychiatrye*. 2012;2(2):112-124. doi:10.2174/2210676611202020112

187. McFarlane WR, Levin B, Travis L, et al. Clinical and functional outcomes after 2 years in the early detection and intervention for the prevention of psychosis multisite effectiveness trial. *Schizophr Bull*. 2015;41(1):30-43. doi:10.1093/schbul/sbu108

188. Meneghelli A, Cocchi A, Preti A. “Programma2000”: A multi-modal pilot programme on early intervention in psychosis underway in Italy since 1999. *Early Interv Psychiatry*. 2010;4(1):97-103. doi:10.1111/j.1751-7893.2009.00158.x

189. Menghini-Müller S, Studerus E, Ittig S, et al. Gender differences of patients at-risk for psychosis regarding symptomatology, drug use, comorbidity and functioning – Results from the EU-GEI study. *Eur Psychiatry*. 2019;59:52-59. doi:10.1016/j.eurpsy.2019.04.007

190. Metzler S, Dvorsky D, Wyss C, et al. Neurocognitive profiles in help-seeking individuals: Comparison of risk for psychosis and bipolar disorder criteria. *Psychol Med*. 2014;44(16):3543-3555. doi:10.1017/S0033291714001007

191. Meyer SE, Bearden CE, Lux SR, et al. The psychosis prodrome in adolescent patients viewed through the lens of DSM-IV. *J Child Adolesc Psychopharmacol*. 2005;15(3):434-451. doi:10.1089/cap.2005.15.434

192. Michel C, Ruhrmann S, Schimmelmann BG, Klosterkötter J, Schultze-Lutter F. A stratified model for psychosis prediction in clinical practice. *Schizophr Bull*. 2014;40(6):1533-1542. doi:10.1093/schbul/sbu025

193. Michel C, Ruhrmann S, Schimmelmann BG, Klosterkötter J, Schultze-Lutter F. Course of clinical high-risk states for psychosis beyond conversion. *Eur Arch Psychiatry Clin Neurosci*. 2018;268(1):39-48. doi:10.1007/s00406-016-0764-8

194. Millman ZB, Weintraub MJ, Bentley E, et al. Differential relations of locus of control to perceived social stress among help-seeking adolescents at low vs. high clinical risk of psychosis. *Schizophr Res*. 2017;184:39-44. doi:10.1016/j.schres.2016.12.006

195. Millman ZB, Pitts SC, Thompson E, et al. Perceived social stress and symptom severity among help-seeking adolescents with versus without clinical high-risk for psychosis. *Schizophr Res*. 2018;192:364-370. doi:10.1016/j.schres.2017.06.002

196. Millman ZB, Rakhshan Rouhakhtar PJ, DeVylder JE, et al. Evidence for differential predictive performance of the prime screen between black and white help-seeking youths. *Psychiatr Serv*. 2019;70(10):907-914. doi:10.1176/appi.ps.201800536

197. Millman ZB, Gallagher K, Demro C, et al. Evidence of reward system dysfunction in youth at clinical high-risk for psychosis from two event-related fMRI paradigms. *Schizophr Res*. 2020;226:111-119. doi:10.1016/j.schres.2019.03.017

198. Mittal VA, Walker EF, Bearden CE, et al. Markers of Basal Ganglia Dysfunction and Conversion to Psychosis: Neurocognitive Deficits and Dyskinesias in the Prodromal Period. *Biol Psychiatry*. 2010;68(1):93-99. doi:10.1016/j.biopsych.2010.01.021

199. Modinos G, Allen P, Frascarelli M, et al. Are we really mapping psychosis risk? Neuroanatomical signature of affective disorders in subjects at ultra high risk. *Psychol Med*. 2014;44(16):3491-3501. doi:10.1017/S0033291714000865

200. Modinos G, Richter A, Egerton A, et al. Interactions between hippocampal activity and striatal dopamine in people at clinical high risk for psychosis: relationship to adverse outcomes. *Neuropsychopharmacology*. 2021;46(8):1468-1474. doi:10.1038/s41386-021-01019-0

201. Morcillo C, Stochl J, Russo DA, et al. First-rank symptoms and premorbid adjustment in young individuals at increased risk of developing psychosis. *Psychopathology*. 2015;48(2):120-126. doi:10.1159/000369859

202. Morrison AP, French P, Stewart SLK, et al. Early detection and intervention evaluation for people at risk of psychosis: Multisite randomised controlled trial. *BMJ*. 2012;344(7852). doi:10.1136/bmj.e2233

203. Mukkala S, Ilonen T, Koskela J, et al. Response initiation in young adults at risk for psychosis in the Northern Finland 1986 Birth Cohort. *Cogn Neuropsychiatry*. 2014;19(3):226-240. doi:10.1080/13546805.2013.840569

204. Nägele FL, Pasternak O, Bitzan L V., et al. Cellular and extracellular white matter alterations indicate conversion to psychosis among individuals at clinical high-risk for psychosis. *World J Biol Psychiatry*. 2020;22(3):1-14. doi:10.1080/15622975.2020.1775890

205. Natsubori T, Inoue H, Abe O, et al. Reduced frontal glutamate + glutamine and N-acetylaspartate levels in patients with chronic schizophrenia but not in those at clinical high risk for psychosis or with first-episode schizophrenia. *Schizophr Bull*. 2014;40(5):1128-1139. doi:10.1093/schbul/sbt124

206. Nelson B, Thompson A, Yung AR. Basic self-disturbance predicts psychosis onset in the ultra high risk for psychosis “prodromal” population. *Schizophr Bull*. 2012;38(6):1277-1287. doi:10.1093/schbul/sbs007

207. Nelson B, Lavoie S, Gawęda, et al. The neurophenomenology of early psychosis: An integrative empirical study. *Conscious Cogn*. 2020;77:102845. doi:10.1016/j.concog.2019.102845

208. Niendam TA, Berzak J, Cannon TD, Bearden CE. Obsessive compulsive symptoms in the psychosis prodrome: Correlates of clinical and functional outcome. *Schizophr Res*. 2009;108(1-3):170-175. doi:10.1016/j.schres.2008.11.023

209. O’Brien MP, Zinberg JL, Bearden CE, et al. Psychoeducational multi-family group treatment with adolescents at high risk for developing psychosis. *Early Interv Psychiatry*. 2007;1(4):325-332. doi:10.1111/j.1751-7893.2007.00046.x

210. Ohmuro N, Matsumoto K, Katsura M, et al. The association between cognitive deficits and depressive symptoms in at-risk mental state: A comparison with first-episode psychosis. *Schizophr Res*. 2015;162(1-3):67-73. doi:10.1016/j.schres.2015.01.008

211. Ohmuro N, Matsumoto K, Ishii Y, et al. The associations between quality of life and clinical symptoms in individuals with an at-risk mental state and first-episode psychosis. *Psychiatry Res*. 2017;254:54-59. doi:10.1016/j.psychres.2017.04.031

212. Oppetit A, Bourgin J, Martinez G, et al. The C’JAAD: a French team for early intervention in psychosis in Paris. *Early Interv Psychiatry*. 2018;12(2):243-249. doi:10.1111/eip.12376

213. Pantelis C, Velakoulis D, McGorry PD, et al. Neuroanatomical abnormalities before and after onset of psychosis: A cross-sectional and longitudinal MRI comparison. *Lancet*. 2003;361(9354):281-288. doi:10.1016/S0140-6736(03)12323-9

214. Park HY, Seo E, Park KM, Koo SJ, Lee E, An SK. Shame and guilt in youth at ultra-high risk for psychosis. *Compr Psychiatry*. 2021;108:152241. doi:10.1016/j.comppsych.2021.152241

215. Pawełczyk A, Łojek E, Żurner N, Kotlicka-Antczak M, Pawełczyk T. Higher order language impairments can predict the transition of ultrahigh risk state to psychosis—An empirical study. *Early Interv Psychiatry*. 2021;15(2):314-327. doi:10.1111/eip.12943

216. Pelizza L, Azzali S, Garlassi S, et al. Adolescents at ultra-high risk of psychosis in Italian neuropsychiatry services: prevalence, psychopathology and transition rate. *Eur Child Adolesc Psychiatry*. 2018;27(6):725-737. doi:10.1007/s00787-017-1070-3

217. Pelizza L, Azzali S, Paterlini F, et al. The “Reggio Emilia At-Risk Mental States” program: A diffused, “liquid” model of early intervention in psychosis implemented in an Italian Department of Mental Health. *Early Interv Psychiatry*. 2019;13(6):1513-1524. doi:10.1111/eip.12851

218. Pelizza L, Azzali S, Paterlini F, et al. Characterization of young people with first episode psychosis or at ultra-high risk: The Reggio Emilia At-Risk Mental States (ReARMS) program. *Riv Psichiatr*. 2019;54(6):254-263. doi:10.1708/3281.32544

219. Pelizza L, Poletti M, Azzali S, et al. Suicide risk in young people at Ultra-High Risk (UHR) of psychosis: Findings from a 2-year longitudinal study. *Schizophr Res*. 2020;220:98-105. doi:10.1016/j.schres.2020.03.051

220. Pelizza L, Azzali S, Paterlini F, et al. Anhedonia in the Psychosis Risk Syndrome: State and Trait Characteristics. *Psychiatr Danub*. 2021;33(1):36-47. doi:10.24869/psyd.2021.36

221. Perkins DO, Jeffries CD, Addington J, et al. Towards a Psychosis Risk Blood Diagnostic for Persons Experiencing High-Risk Symptoms: Preliminary Results from the NAPLS Project. *Schizophr Bull*. 2015;41(2):419-428. doi:10.1093/schbul/sbu099

222. Peters BD, De Haan L, Dekker N, et al. White matter fibertracking in first-episode schizophrenia, schizoaffective patients and subjects at ultra-high risk of psychosis. *Neuropsychobiology*. 2008;58(1):19-28. doi:10.1159/000154476

223. Phillips LJ, Velakoulis D, Pantelis C, et al. Non-reduction in hippocampal volume is associated with higher risk of psychosis. *Schizophr Res*. 2002;58(2-3):145-158. doi:10.1016/S0920-9964(01)00392-9

224. Phillips LJ, Curry C, Yung AR, Yuen HP, Adlard S, McGorry PD. Cannabis use is not associated with the development of psychosis in an “ultra” high-risk group. *Aust N Z J Psychiatry*. 2002;36(6):800-806. doi:10.1046/j.1440-1614.2002.01089.x

225. Phillips LJ, Nelson B, Yuen HP, et al. Randomized controlled trial of interventions for young people at ultra-high risk of psychosis: Study design and baseline characteristics. *Aust N Z J Psychiatry*. 2009;43(9):818-829. doi:10.1080/00048670903107625

226. Piskulic D, Addington J, Cadenhead KS, et al. Negative symptoms in individuals at clinical high risk of psychosis. *Psychiatry Res*. 2012;196(2-3):220-224. doi:10.1016/j.psychres.2012.02.018

227. Poletti M, Pelizza L, Azzali S, et al. Clinical high risk for psychosis in childhood and adolescence: findings from the 2-year follow-up of the ReARMS project. *Eur Child Adolesc Psychiatry*. 2019;28(7):957-971. doi:10.1007/s00787-018-1262-5

228. Poletti M, Pelizza L, Azzali S, et al. Overcoming the gap between child and adult mental health services: The Reggio Emilia experience in an early intervention in psychosis program. *Early Interv Psychiatry*. 2021;15(6):1749-1758. doi:10.1111/eip.13097

229. Pollak TA, Kempton MJ, Iyegbe C, et al. Clinical, cognitive and neuroanatomical associations of serum NMDAR autoantibodies in people at clinical high risk for psychosis. *Mol Psychiatry*. 2021;26(6):2590-2604. doi:10.1038/s41380-020-00899-w

230. Pozza A, Meneghelli A, Meliante M, Amato L, Dèttore D. Anxiety sensitivity dimensions in young individuals with at-risk-mental states. *Res Psychother Psychopathol Process Outcome*. 2020;23(1):38-47. doi:10.4081/ripppo.2020.431

231. Pozza A, Dèttore D. Modular cognitive-behavioral therapy for affective symptoms in young individuals at ultra-high risk of first episode of psychosis: Randomized controlled trial. *J Clin Psychol*. 2020;76(3):392-405. doi:10.1002/jclp.22901

232. Pozza A, Domenichetti S, Dèttore D. Cognitive behavioural therapy for help-seeking adolescents and young adults with at-risk-mental state: Effects on subclinical positive symptoms. *Early Interv Psychiatry*. 2021;15(3):513-524. doi:https://doi.org/10.1111/eip.12974

233. Preda A, Miller TJ, Rosen JL, Somjee L, McGlashan TH, Woods SW. Treatment histories of patients with a syndrome putatively prodromal to schizophrenia. *Psychiatr Serv*. 2002;53(3):342-344. doi:10.1176/appi.ps.53.3.342

234. Preti A, Meneghelli A, Pisano A, Cocchi A. Risk of suicide and suicidal ideation in psychosis: Results from an Italian multi-modal pilot program on early intervention in psychosis. *Schizophr Res*. 2009;113(2-3):145-150. doi:10.1016/j.schres.2009.06.007

235. Pruessner M, Iyer SN, Faridi K, Joober R, Malla AK. Stress and protective factors in individuals at ultra-high risk for psychosis, first episode psychosis and healthy controls. *Schizophr Res*. 2011;129(1):29-35. doi:10.1016/j.schres.2011.03.022

236. Raballo A, Pappagallo E, Dell Erba A, et al. Self-disorders and clinical high risk for psychosis: An empirical study in help-seeking youth attending community mental health facilities. *Schizophr Bull*. 2016;42(4):926-932. doi:10.1093/schbul/sbv223

237. Ramyead A, Studerus E, Kometer M, et al. Prediction of psychosis using neural oscillations and machine learning in neuroleptic-naïve at-risk patients. *World J Biol Psychiatry*. 2016;17(4):285-295. doi:10.3109/15622975.2015.1083614

238. Randers L, Fagerlund B, Jepsen JRM, et al. Interview and questionnaire assessment of cognitive impairment in subjects at ultra-high risk for psychosis: Associations with cognitive test performance, psychosocial functioning, and positive symptoms. *Psychiatry Res*. 2020;294:113498. doi:10.1016/j.psychres.2020.113498

239. Ranlund S, Nottage J, Shaikh M, et al. Resting EEG in psychosis and at-risk populations - A possible endophenotype? *Schizophr Res*. 2014;153(1-3):96-102. doi:10.1016/j.schres.2013.12.017

240. Ratheesh A, Lin A, Nelson B, et al. Neurocognitive functioning in the prodrome of mania - An exploratory study. *J Affect Disord*. 2013;147(1-3):441-445. doi:10.1016/j.jad.2012.09.017

241. Reininghaus U, Kempton MJ, Valmaggia L, et al. Stress sensitivity, aberrant salience, and threat anticipation in early psychosis: An experience sampling study. *Schizophr Bull*. 2016;42(3):712-722. doi:10.1093/schbul/sbv190

242. Reininghaus U, Oorschot M, Moritz S, et al. Liberal acceptance bias, momentary aberrant salience, and psychosis: An experimental experience sampling study. *Schizophr Bull*. 2019;45(4):871-882. doi:10.1093/schbul/sby116

243. Rekhi G, Ng WY, Lee J. Clinical utility of the Calgary Depression Scale for Schizophrenia in individuals at ultra-high risk of psychosis. *Schizophr Res*. 2018;193:423-427. doi:10.1016/j.schres.2017.06.056

244. Rekhi G, Rapisarda A, Lee J. Impact of distress related to attenuated psychotic symptoms in individuals at ultra high risk of psychosis: Findings from the Longitudinal Youth at Risk Study. *Early Interv Psychiatry*. 2019;13(1):73-78. doi:10.1111/eip.12451

245. Ribolsi M, Lin A, Wardenaar KJ, et al. Clinical presentation of Attenuated Psychosis Syndrome in children and adolescents: Is there an age effect? *Psychiatry Res*. 2017;252:169-174. doi:10.1016/j.psychres.2017.02.050

246. Riecher-Rössler A, Pflueger MO, Aston J, et al. Efficacy of Using Cognitive Status in Predicting Psychosis: A 7-Year Follow-Up. *Biol Psychiatry*. 2009;66(11):1023-1030. doi:10.1016/j.biopsych.2009.07.020

247. Rietdijk J, Ising HK, Dragt S, et al. Depression and social anxiety in help-seeking patients with an ultra-high risk for developing psychosis. *Psychiatry Res*. 2013;209(3):309-313. doi:10.1016/j.psychres.2013.01.012

248. Rietschel L, Lambert M, Karow A, et al. Clinical high risk for psychosis: gender differences in symptoms and social functioning. *Early Interv Psychiatry*. 2017;11(4):306-313. doi:10.1111/eip.12240

249. Rosen JL, Miller TJ, D’Andrea JT, McGlashan TH, Woods SW. Comorbid diagnoses in patients meeting criteria for the schizophrenia prodrome. *Schizophr Res*. 2006;85(1-3):124-131. doi:10.1016/j.schres.2006.03.034

250. Ruhrmann S, Schultze-Lutter F, Salokangas RKR, et al. Prediction of psychosis in adolescents and young adults at high risk: Results from the prospective European prediction of psychosis study. *Arch Gen Psychiatry*. 2010;67(3):241-251. doi:10.1001/archgenpsychiatry.2009.206

251. Russo DA, Stochl J, Painter M, et al. Trauma history characteristics associated with mental states at clinical high risk for psychosis. *Psychiatry Res*. 2014;220(1-2):237-244. doi:10.1016/j.psychres.2014.08.028

252. Russo DA, Stochl J, Painter M, Jones PB, Perez J. Substance use in people at clinical high-risk for psychosis. *BMC Psychiatry*. 2014;14(1):361. doi:10.1186/s12888-014-0361-1

253. Russo DA, Stochl J, Hodgekins J, et al. Attachment styles and clinical correlates in people at ultra high risk for psychosis. *Br J Psychol*. 2018;109(1):45-62. doi:10.1111/bjop.12249

254. Rutigliano G, Valmaggia L, Landi P, et al. Persistence or recurrence of non-psychotic comorbid mental disorders associated with 6-year poor functional outcomes in patients at ultra high risk for psychosis. *J Affect Disord*. 2016;203:101-110. doi:https://doi.org/10.1016/j.jad.2016.05.053

255. Ryan J, Graham A, Nelson B, Yung A. Borderline personality pathology in young people at ultra high risk of developing a psychotic disorder. *Early Interv Psychiatry*. 2017;11(3):208-214. doi:10.1111/eip.12236

256. Sabb FW, van Erp TGM, Hardt ME, et al. Language network dysfunction as a predictor of outcome in youth at clinical high risk for psychosis. *Schizophr Res*. 2010;116(2-3):173-183. doi:10.1016/j.schres.2009.09.042

257. Salinger JM, O’Brien MP, Miklowitz DJ, Marvin SE, Cannon TD. Family communication with teens at clinical high-risk for psychosis or bipolar disorder. *J Fam Psychol*. 2018;32(4):507-516. doi:10.1037/fam0000393

258. Salokangas RKR, Ruhrmann S, von Reventlow HG, et al. Axis I diagnoses and transition to psychosis in clinical high-risk patients EPOS project: Prospective follow-up of 245 clinical high-risk outpatients in four countries. *Schizophr Res*. 2012;138(2):192-197. doi:https://doi.org/10.1016/j.schres.2012.03.008

259. Salokangas RKR, Dingemans P, Heinimaa M, et al. Prediction of psychosis in clinical high-risk patients by the Schizotypal Personality Questionnaire. Results of the EPOS project. *Eur Psychiatry*. 2013;28(8):469-475. doi:10.1016/j.eurpsy.2013.01.001

260. Salokangas RKR, Schultze-Lutter F, Hietala J, et al. Depression predicts persistence of paranoia in clinical high-risk patients to psychosis: results of the EPOS project. *Soc Psychiatry Psychiatr Epidemiol*. 2016;51(2):247-257. doi:10.1007/s00127-015-1160-9

261. Salokangas RKR, Patterson P, Hietala J, et al. Childhood adversity predicts persistence of suicidal thoughts differently in females and males at clinical high-risk patients of psychosis. Results of the EPOS project. *Early Interv Psychiatry*. 2019;13(4):935-942. doi:10.1111/eip.12714

262. Schlosser DA, Jacobson S, Chen Q, et al. Recovery from an at-risk state: Clinical and functional outcomes of putatively prodromal youth who do not develop psychosis. *Schizophr Bull*. 2012;38(6):1225-1233. doi:10.1093/schbul/sbr098

263. Schmidt SJ, Grunert VM, Schimmelmann BG, Schultze-Lutter F, Michel C. Differences in coping, self-efficacy, and external control beliefs between patients at-risk for psychosis and patients with first-episode psychosis. *Psychiatry Res*. 2014;219(1):95-102. doi:10.1016/j.psychres.2014.04.045

264. Schmidt SJ, Schultze-Lutter F, Bendall S, et al. Mediators linking childhood adversities and trauma to suicidality in individuals at risk for psychosis. *Front Psychiatry*. 2017;8(NOV):242. doi:10.3389/fpsyt.2017.00242

265. Schultze-Lutter F, Ruhrmann S, Picker H, et al. Relationship between subjective and objective cognitive function in the early and late prodrome. *Br J Psychiatry*. 2007;191(SUPPL. 51):s43-51. doi:10.1192/bjp.191.51.s43

266. Schultze-Lutter F, Ruhrmann S, Picker H, Von Reventlow HG, Brockhaus-Dumke A, Klosterkötter J. Basic symptoms in early psychotic and depressive disorders. *Br J Psychiatry*. 2007;191(SUPPL. 51):s31-7. doi:10.1192/bjp.191.51.s31

267. Schultze-Lutter F, Klosterkötter J, Michel C, Winkler K, Ruhrmann S. Personality disorders and accentuations in at-risk persons with and without conversion to first-episode psychosis. *Early Interv Psychiatry*. 2012;6(4):389-398. doi:10.1111/j.1751-7893.2011.00324.x

268. Schultze-Lutter F, Hubl D, Schimmelmann BG, Michel C. Age effect on prevalence of ultra-high risk for psychosis symptoms: replication in a clinical sample of an early detection of psychosis service. *Eur Child Adolesc Psychiatry*. 2017;26(11):1401-1405. doi:10.1007/s00787-017-0994-y

269. Schulze C, Zimmermann R, Gschwandtner U, et al. Can cognitive deficits facilitate differential diagnosis between at-risk mental state for psychosis and depressive disorders? *Early Interv Psychiatry*. 2013;7(4):381-390. doi:10.1111/eip.12004

270. Shi J, Wang L, Yao Y, et al. Comorbid mental disorders and 6-month symptomatic and functioning outcomes in chinese university students at clinical high risk for psychosis. *Front Psychiatry*. 2017;8(OCT):209. doi:10.3389/fpsyt.2017.00209

271. Simeonova DI, Lee FJ, Walker EF. Longitudinal investigation of the relationship between family history of psychosis and affective disorders and Child Behavior Checklist ratings in clinical high-risk adolescents. *Schizophr Res*. 2015;166(1-3):24-30. doi:10.1016/j.schres.2015.04.027

272. Simon AE, Dvorsky DN, Boesch J, et al. Defining subjects at risk for psychosis: A comparison of two approaches. *Schizophr Res*. 2006;81(1):83-90. doi:10.1016/j.schres.2005.10.006

273. Simon AE, Cattapan-Ludewig K, Zmilacher S, et al. Cognitive functioning in the schizophrenia prodrome. *Schizophr Bull*. 2007;33(3):761-771. doi:10.1093/schbul/sbm018

274. Simon AE, Umbricht D. High remission rates from an initial ultra-high risk state for psychosis. *Schizophr Res*. 2010;116(2-3):168-172. doi:10.1016/j.schres.2009.10.001

275. Simon AE, Grädel M, Cattapan-Ludewig K, et al. Cognitive functioning in at-risk mental states for psychosis and 2-year clinical outcome. *Schizophr Res*. 2012;142(1-3):108-115. doi:10.1016/j.schres.2012.09.004

276. Smith CW, Park S, Cornblatt B. Spatial working memory deficits in adolescents at clinical high risk for schizophrenia. *Schizophr Res*. 2006;81(2-3):211-215. doi:10.1016/j.schres.2005.09.019

277. Song YY, Kang JI, Kim SJ, Lee MK, Lee E, An SK. Temperament and character in individuals at ultra-high risk for psychosis and with first-episode schizophrenia: Associations with psychopathology, psychosocial functioning, and aspects of psychological health. *Compr Psychiatry*. 2013;54(8):1161-1168. doi:10.1016/j.comppsych.2013.05.015

278. Spada G, Molteni S, Pistone C, et al. Identifying children and adolescents at ultra high risk of psychosis in Italian neuropsychiatry services: a feasibility study. *Eur Child Adolesc Psychiatry*. 2016;25(1):91-106. doi:10.1007/s00787-015-0710-8

279. Spark J, Gawęda Ł, Allott K, et al. Distinguishing schizophrenia spectrum from non-spectrum disorders among young patients with first episode psychosis and at high clinical risk: The role of basic self-disturbance and neurocognition. *Schizophr Res*. 2021;228:19-28. doi:10.1016/j.schres.2020.11.061

280. Stain HJ, Bucci S, Baker AL, et al. A randomised controlled trial of cognitive behaviour therapy versus non-directive reflective listening for young people at ultra high risk of developing psychosis: The detection and evaluation of psychological therapy (DEPTh) trial. *Schizophr Res*. 2016;176(2-3):212-219. doi:10.1016/j.schres.2016.08.008

281. Stain HJ, Halpin SA, Baker AL, et al. Impact of rurality and substance use on young people at ultra high risk for psychosis. *Early Interv Psychiatry*. 2018;12(6):1173-1180. doi:10.1111/eip.12437

282. Sterk B, Lankreijer K, Linszen DH, De Haan L. Obsessive-compulsive symptoms in first episode psychosis and in subjects at ultra high risk for developing psychosis; Onset and relationship to psychotic symptoms. *Aust N Z J Psychiatry*. 2011;45(5):400-406. doi:10.3109/00048674.2010.533363

283. Takahashi T, Wood SJ, Yung AR, et al. Insular cortex gray matter changes in individuals at ultra-high-risk of developing psychosis. *Schizophr Res*. 2009;111(1-3):94-102. doi:10.1016/j.schres.2009.03.024

284. Takahashi T, Higuchi Y, Komori Y, et al. Quality of life in individuals with attenuated psychotic symptoms: Possible role of anxiety, depressive symptoms, and socio-cognitive impairments. *Psychiatry Res*. 2017;257:431-437. doi:10.1016/j.psychres.2017.08.024

285. Takahashi T, Higuchi Y, Komori Y, et al. Pituitary volume and socio-cognitive functions in individuals at risk of psychosis and patients with schizophrenia. *Front Psychiatry*. 2018;9(NOV):574. doi:10.3389/fpsyt.2018.00574

286. Takahashi T, Nakamura M, Nishikawa Y, et al. Potential role of orbitofrontal surface morphology on social and cognitive functions in high-risk subjects for psychosis and schizophrenia patients. *Psychiatry Res - Neuroimaging*. 2019;283:92-95. doi:10.1016/j.pscychresns.2018.12.002

287. Takahashi T, Sasabayashi D, Takayanagi Y, et al. Heschl’s Gyrus Duplication Pattern in Individuals at Risk of Developing Psychosis and Patients With Schizophrenia. *Front Behav Neurosci*. 2021;15:647069. doi:10.3389/fnbeh.2021.647069

288. Talib LL, Costa AC, Joaquim HPG, et al. Increased PLA2 activity in individuals at ultra-high risk for psychosis. *Eur Arch Psychiatry Clin Neurosci*. 2021;271(8):1593-1599. doi:10.1007/s00406-021-01246-y

289. Tamagni C, Studerus E, Gschwandtner U, Aston J, Borgwardt S, Riecher-Rössler A. Are neurological soft signs pre-existing markers in individuals with an at-risk mental state for psychosis? *Psychiatry Res*. 2013;210(2):427-431. doi:10.1016/j.psychres.2013.06.016

290. Tateno T, Higuchi Y, Nakajima S, et al. Features of Duration Mismatch Negativity around the Onset of Overt Psychotic Disorders: A Longitudinal Study. *Cereb Cortex*. 2021;31(5):2416-2424. doi:10.1093/cercor/bhaa364

291. Tay SA, Yuen S, Lim LK, et al. Support for Wellness Achievement Programme (SWAP): Clinical and demographic characteristics of young people with at-risk mental state in Singapore. *Early Interv Psychiatry*. 2015;9(6):516-522. doi:10.1111/eip.12176

292. Thompson KN, Phillips LJ, Komesaroff P, et al. Stress and HPA-axis functioning in young people at ultra high risk for psychosis. *J Psychiatr Res*. 2007;41(7):561-569. doi:10.1016/j.jpsychires.2006.05.010

293. Thompson A, Nelson B, McNab C, et al. Psychotic symptoms with sexual content in the “ultra high risk” for psychosis population: Frequency and association with sexual trauma. *Psychiatry Res*. 2010;177(1-2):84-91. doi:10.1016/j.psychres.2010.02.011

294. Thompson A, Nelson B, Yung A. Predictive validity of clinical variables in the “at risk” for psychosis population: International comparison with results from the North American Prodrome Longitudinal Study. *Schizophr Res*. 2011;126(1-3):51-57. doi:10.1016/j.schres.2010.09.024

295. Thompson A, Nelson B, Bechdolf A, et al. Borderline Personality Features And Development Of Psychosis In An “ultra High Risk” (Uhr) Population: A Case Control Study. *Early Interv Psychiatry*. 2012;6(3):247-255. doi:10.1111/j.1751-7893.2012.00365.x

296. Johnson L. Maintenance of certification: To the editor [5]. *Ann Intern Med*. 2015;162(1):79. doi:10.7326/L15-5040-5

297. Thompson EC, Andorko ND, Rakhshan Rouhakhtar P, et al. Psychosis-Spectrum Screening and Assessment within a College Counseling Center: A Pilot Study Exploring Feasibility and Clinical Need. *J College Stud Psychother*. Published online September 2020:1-22. doi:10.1080/87568225.2020.1797604

298. Tomyshev AS, Lebedeva IS, Akhadov TA, et al. MRI Study for the Features of Brain Conduction Pathways in Patients with an Ultra-High Risk of Endogenous Psychoses. *Bull Exp Biol Med*. 2017;162(4):425-429. doi:10.1007/s10517-017-3631-3

299. Tor J, Dolz M, Sintes-Estevez A, et al. Neuropsychological profile of children and adolescents with psychosis risk syndrome: the CAPRIS study. *Eur Child Adolesc Psychiatry*. 2020;29(9):1311-1324. doi:10.1007/s00787-019-01459-6

300. Tseng HH, Watts JJ, Kiang M, et al. Nigral Stress-Induced Dopamine Release in Clinical High Risk and Antipsychotic-Naïve Schizophrenia. *Schizophr Bull*. 2018;44(3):542-551. doi:10.1093/schbul/sbx042

301. Üçok A, Direk N, Kaya H, et al. Relationship of negative symptom severity with cognitive symptoms and functioning in subjects at ultra-high risk for psychosis. *Early Interv Psychiatry*. 2021;15(4):966-974. doi:10.1111/eip.13042

302. Værnes TG, Røssberg JI, Møller P. Anomalous self-experiences are strongly associated with negative symptoms in a clinical high-risk for psychosis sample. *Compr Psychiatry*. 2019;93:65-72. doi:10.1016/j.comppsych.2019.07.003

303. Van Der Gaag M, Nieman DH, Rietdijk J, et al. Cognitive behavioral therapy for subjects at ultrahigh risk for developing psychosis: A randomized controlled clinical trial. *Schizophr Bull*. 2012;38(6):1180-1188. doi:10.1093/schbul/sbs105

304. van der Steen Y, Gimpel-Drees J, Lataster T, et al. Clinical high risk for psychosis: the association between momentary stress, affective and psychotic symptoms. *Acta Psychiatr Scand*. 2017;136(1):63-73. doi:10.1111/acps.12714

305. Van Rijn S, Aleman A, De Sonneville L, et al. Misattribution of facial expressions of emotion in adolescents at increased risk of psychosis: The role of inhibitory control. *Psychol Med*. 2011;41(3):499-508. doi:10.1017/S0033291710000929

306. Velakoulis D, Wood SJ, Wong MTH, et al. Hippocampal and amygdala volumes according to psychosis stage and diagnosis: A magnetic resonance imaging study of chronic schizophrenia, first-episode psychosis, and ultra-high-risk individuals. *Arch Gen Psychiatry*. 2006;63(2):139-149. doi:10.1001/archpsyc.63.2.139

307. Velthorst E, Nieman DH, Becker HE, et al. Baseline differences in clinical symptomatology between ultra high risk subjects with and without a transition to psychosis. *Schizophr Res*. 2009;109(1-3):60-65. doi:10.1016/j.schres.2009.02.002

308. Velthorst E, Nieman DH, Veling W, et al. Ethnicity and baseline symptomatology in patients with an at Risk Mental State for psychosis. *Psychol Med*. 2012;42(2):247-256. doi:10.1017/S0033291711001486

309. Velthorst E, Nelson B, O’Connor K, et al. History of trauma and the association with baseline symptoms in an Ultra-High Risk for psychosis cohort. *Psychiatry Res*. 2013;210(1):75-81. doi:10.1016/j.psychres.2013.06.007

310. von Hohenberg CC, Pasternak O, Kubicki M, et al. White Matter Microstructure in Individuals at Clinical High Risk of Psychosis: A Whole-Brain Diffusion Tensor Imaging Study. *Schizophr Bull*. 2014;40(4):895-903. doi:10.1093/schbul/sbt079

311. Walker EF, Brennan PA, Esterberg M, Brasfield J, Pearce B, Compton MT. Longitudinal changes in cortisol secretion and conversion to psychosis in at-risk youth. *J Abnorm Psychol*. 2010;119(2):401-408. doi:10.1037/a0018399

312. Walterfang M, McGuire PK, Yung AR, et al. White matter volume changes in people who develop psychosis. *Br J Psychiatry*. 2008;193(3):210-215. doi:10.1192/bjp.bp.107.043463

313. Wang C, Lee J, Ho NF, et al. Large-scale network topology reveals heterogeneity in individuals with at risk mental state for psychosis: Findings from the longitudinal youth-at-risk study. *Cereb Cortex*. 2018;28(12):4234-4243. doi:10.1093/cercor/bhx278

314. Webb JR, Addington J, Perkins DO, et al. Specificity of Incident Diagnostic Outcomes in Patients at Clinical High Risk for Psychosis. *Schizophr Bull*. 2015;41(5):1066-1075. doi:10.1093/schbul/sbv091

315. Welsh P, Tiffin PA. The “At-Risk Mental State” for psychosis in adolescents: Clinical presentation, transition and remission. *Child Psychiatry Hum Dev*. 2014;45(1):90-98. doi:10.1007/s10578-013-0380-z

316. Wood SJ, Brewer WJ, Koutsouradis P, et al. Cognitive decline following psychosis onset: Data from the PACE clinic. *Br J Psychiatry*. 2007;191(SUPPL. 51):s52-7. doi:10.1192/bjp.191.51.s52

317. Woodberry KA, Seidman LJ, Bryant C, et al. Treatment Precedes Positive Symptoms in North American Adolescent and Young Adult Clinical High Risk Cohort. *J Clin Child Adolesc Psychol*. 2018;47(1):69-78. doi:10.1080/15374416.2016.1212361

318. Woods SW, Addington J, Cadenhead KS, et al. Validity of the prodromal risk syndrome for first psychosis: Findings from the north american prodrome longitudinal study. *Schizophr Bull*. 2009;35(5):894-908. doi:10.1093/schbul/sbp027

319. Woods SW, Addington J, Bearden CE, et al. Psychotropic medication use in youth at high risk for psychosis: Comparison of baseline data from two research cohorts 1998-2005 and 2008-2011. *Schizophr Res*. 2013;148(1-3):99-104. doi:10.1016/j.schres.2013.05.019

320. Yang LH, Woodberry KA, Link BG, et al. Impact of “psychosis risk” identification: Examining predictors of how youth view themselves. *Schizophr Res*. 2019;208:300-307. doi:10.1016/j.schres.2019.01.037

321. Yee JY, Lee TS, Lee J. Levels of serum brain-derived neurotropic factor in individuals at ultra-high risk for psychosis-findings from the longitudinal youth at risk study (LYRIKS). *Int J Neuropsychopharmacol*. 2018;21(8):734-739. doi:10.1093/ijnp/pyy036

322. Yücel M, Wood SJ, Phillips LJ, et al. Morphology of the anterior cingulate cortex in young men at ultra-high risk of developing a psychotic illness. *Br J Psychiatry*. 2003;182(JUNE):518-524. doi:10.1192/bjp.182.6.518

323. Yung AR, Phillips LJ, Yuen HP, et al. Psychosis prediction: 12-Month follow up of a high-risk (“prodromal”) group. *Schizophr Res*. 2003;60(1):21-32. doi:10.1016/S0920-9964(02)00167-6

324. Yung AR, Stanford C, Cosgrave E, et al. Testing the Ultra High Risk (prodromal) criteria for the prediction of psychosis in a clinical sample of young people. *Schizophr Res*. 2006;84(1):57-66. doi:10.1016/j.schres.2006.03.014

325. Yung AR, Buckby JA, Cosgrave EM, et al. Association between psychotic experiences and depression in a clinical sample over 6 months. *Schizophr Res*. 2007;91(1-3):246-253. doi:10.1016/j.schres.2006.11.026

326. Ziermans TB, Durston S, Sprong M, et al. No evidence for structural brain changes in young adolescents at ultra high risk for psychosis. *Schizophr Res*. 2009;112(1-3):1-6. doi:10.1016/j.schres.2009.04.013

327. Zikidi K, Gajwani R, Gross J, et al. Grey-matter abnormalities in clinical high-risk participants for psychosis. *Schizophr Res*. 2020;226:120-128. doi:10.1016/j.schres.2019.08.034

328. Zink M, Schirmbeck F, Rausch F, et al. Obsessive-compulsive symptoms in at-risk mental states for psychosis: associations with clinical impairment and cognitive function. *Acta Psychiatr Scand*. 2014;130(3):214-226. doi:10.1111/acps.12258
