# Supplementary material for: Meta-analytic prevalence of comorbid mental disorders in individuals at clinical high risk of psychosis: the case for transdiagnostic assessment
Source: Mol Psychiatry. 2023 Jun 9;28(6):2291–300. doi: 10.1038/s41380-023-02029-8 (PMC10611568; doi:10.1038/s41380-023-02029-8)
Supplement: Supplementary file 2 — Supplementary material part 2 [file 41380_2023_2029_MOESM2_ESM.docx]

**APPENDIX 2 – Studies excluded after full-text assessment, part 1**

**Meta-analytic prevalence of comorbid mental disorders in individuals at clinical high risk of psychosis: the case for transdiagnostic assessment**

Marco Solmi1-6, Livia Soardo7, Simi Kaur5, Matilza Azis5, Anna Cabras8, Marco Censori9-10, Luigi Fausti7, Filippo Besana7, Gonzalo Salazar de Pablo5-11-13, Paolo Fusar-Poli5,7

1 Department of Psychiatry, University of Ottawa, Ontario, Canada.

2 On Track, First Episode Psychosis Program, Department of Mental Health, The Ottawa Hospital, Ontario, Canada.

3 Ottawa Hospital Research Institute (OHRI) Clinical Epidemiology Program University of Ottawa Ottawa Ontario

4 School of Epidemiology and Public Health, Faculty of Medicine, University of Ottawa, Ottawa, Canada

5 Early Psychosis: Interventions and Clinical-detection (EPIC) Lab, Institute of Psychiatry, Psychology & Neuroscience, Department of Psychosis Studies, King's College London, London, United Kingdom.

6 Department of Child and Adolescent Psychiatry, Charité Universitätsmedizin, Berlin, Germany

7 Department of Brain and Behavioral Sciences, University of Pavia, Pavia, Italy

8 Sapienza University of Rome, Department of Neurology and Psychiatry

9 Department of Neuroscience (DNS), University of Padova, Padua, Italy

10 Dipartimento di Salute Mentale, Azienda ULSS 3 Serenissima, Venezia, Italy

11 Department of Child and Adolescent Psychiatry, Institute of Psychiatry, Psychology & Neuroscience, King’s College London UK

12 Child and Adolescent Mental Health Services, South London and Maudsley NHS Foundation Trust, London, UK

13 Institute of Psychiatry and Mental Health. Department of Child and Adolescent Psychiatry, Hospital General Universitario Gregorio Marañón School of Medicine, Universidad Complutense, Instituto de Investigación Sanitaria Gregorio Marañón (IiSGM), CIBERSAM, Madrid, Spain

Corresponding author

Paolo Fusar-Poli

paolo.fusar-poli@kcl.ac.uk

| **Excluded studies after full-text assessment** | |
| --- | --- |
| Author, year | Reason for exclusion |
| 1. Aase 2018 ^1^ | No relevant outcome |
| 1. Adamson 2018 ^2^ | No relevant outcome |
| 1. Addington 2005 ^3^ | Study Design |
| 1. Addington 2008 ^4^ | No relevant outcome |
| 1. Addington 2008 ^5^ | No relevant outcome |
| 1. Addington 2008 ^6^ | No dsm/icd/cut off scale criteria |
| 1. Addington 2009 ^7^ | No dsm/icd/cut off scale criteria |
| 1. Addington 2012 ^8^ | No CHR-P population |
| 1. Addington 2012 ^9^ | No relevant outcome |
| 1. Addington 2013 ^10^ | Study Design |
| 1. Addington 2014 ^11^ | No relevant outcome |
| 1. Addington 2015 ^12^ | No relevant outcome |
| 1. Addington 2019 ^13^ | Study Design |
| 1. Addington 2020 ^14^ | No relevant outcome |
| 1. Addington 2021 ^15^ | No relevant outcome |
| 1. Agurto 2020 ^16^ | No relevant outcome |
| 1. Albers 1998 ^17^ | No CHR-P population |
| 1. Alderman 2015 ^18^ | No relevant outcome |
| 1. Aleksandrowicz 2020 ^19^ | No relevant outcome |
| 1. Allen 2010 ^20^ | No relevant outcome |
| 1. Allen 2011 ^21^ | No relevant outcome |
| 1. Allen 2012 ^22^ | No relevant outcome |
| 1. Allen 2012 ^23^ | No relevant outcome |
| 1. Allen 2015 ^24^ | No relevant outcome |
| 1. Allen 2015 ^25^ | No relevant outcome |
| 1. Allen 2018 ^26^ | No dsm/icd/cut off scale criteria |
| 1. Allot 2019 ^27^ | No dsm/icd/cut off scale criteria |
| 1. Allswede 2020 ^28^ | No relevant outcome |
| 1. Alqarni 2020 ^29^ | No relevant outcome |
| 1. Alqarni 2020 ^30^ | No relevant outcome |
| 1. Alvarez-Jimenez 2018 ^31^ | No dsm/icd/cut off scale criteria |
| 1. Amminger 2006 ^32^ | No relevant outcome |
| 1. Amminger 2007 ^33^ | No relevant outcome |
| 1. Amminger 2010 ^34^ | No relevant outcome |
| 1. Amminger 2012 ^35^ | No relevant outcome |
| 1. Amminger 2012 ^36^ | Study Design |
| 1. Amminger 2012 ^37^ | No relevant outcome |
| 1. Amminger 2013 ^38^ | Study Design |
| 1. Amminger 2013 ^39^ | No relevant outcome |
| 1. Amminger 2015 ^40^ | No relevant outcome |
| 1. Amminger 2020 ^41^ | No dsm/icd/cut off scale criteria |
| 1. Amos 2013 ^42^ | Study Design |
| 1. Andersen 2016 ^43^ | No CHR-P population |
| 1. Andrade 2016 ^44^ | Study Design |
| 1. Andreou 2015 ^45^ | no CHR-P population |
| 1. Anglin 2014 ^46^ | No CHR-P population |
| 1. Anglin 2016 ^47^ | No relevant outcome |
| 1. Anglin 2018 ^48^ | No dsm/icd/cut off scale criteria |
| 1. Anonymus 2011^49^ | Study Design |
| 1. Anticevic 2014 ^50^ | No relevant outcome |
| 1. Anticevic 2015 ^51^ | No relevant outcome |
| 1. Appiah-Kusi 2017 ^52^ | No dsm/icd/cut off scale criteria |
| 1. Appiah-Kusi 2020 ^53^ | No dsm/icd/cut off scale criteria |
| 1. Appiah-Kusi 2020 ^54^ | No dsm/icd/cut off scale criteria |
| 1. Armando 2012 ^55^ | No dsm/icd/cut off scale criteria |
| 1. Armando 2017 ^56^ | No relevant outcome |
| 1. Aston 2010 ^57^ | No relevant outcome |
| 1. Atkinson 2012 ^58^ | No relevant outcome |
| 1. Atkinson 2017 ^59^ | No dsm/icd/cut off scale criteria |
| 1. Austin 2012 ^60^ | No CHR-P population |
| 1. Avery 2015 ^61^ | Article not found |
| 1. Aydin 2008 ^62^ | No relevant outcome |
| 1. Aylott 2019 ^63^ | No CHR-P population |
| 1. Azar 2018 ^64^ | No relevant outcome |
| 1. Azzali 2018^65^ | No relevant outcome |
| 1. Ӓrmӓnen 2018 ^66^ | No relevant outcome |
| 1. Baer 2019 ^67^ | No relevant outcome |
| 1. Baker 2011 ^68^ | Study Design |
| 1. Bakker 2016 ^69^ | No CHR-P population |
| 1. Baldelli 2019^70^ | No relevant outcome |
| 1. Ballon 2007 ^71^ | Article not found |
| 1. Bang 2015 ^72^ | No relevant outcome |
| 1. Bang 2018 ^73^ | No relevant outcome |
| 1. Bang 2019 ^74^ | No relevant outcome |
| 1. Bang 2019 ^75^ | No relevant outcome |
| 1. Bang 2019 ^76^ | No relevant outcome |
| 1. Barbato 2013 ^77^ | No relevant outcome |
| 1. Barbato 2013 ^78^ | No relevant outcome |
| 1. Barbato 2014 ^79^ | No relevant outcome |
| 1. Barbato 2014 ^80^ | No relevant outcome |
| 1. Barkus 2010 ^81^ | No relevant outcome |
| 1. Barkus 2011 ^82^ | No dsm/icd/cut off scale criteria |
| 1. Barkus 2016 ^83^ | No CHR-P population |
| 1. Barnett 2005 ^84^ | Study Design |
| 1. Baron 1992 ^85^ | No dsm/icd/cut off scale criteria |
| 1. Barrantes-Vidal 2013 ^86^ | No CHR-P population |
| 1. Bartholomeusz 2014 ^87^ | No dsm/icd/cut off scale criteria |
| 1. Bartok 2005 ^88^ | No dsm/icd/cut off scale criteria |
| 1. Bearden 2011 ^89^ | No relevant outcome |
| 1. Bechdolf 2007 ^90^ | No relevant outcome |
| 1. Bechdolf 2005 ^91^ | No dsm/icd/cut off scale criteria |
| 1. Bechdolf 2005 ^92^ | No dsm/icd/cut off scale criteria |
| 1. Bechdolf 2007 ^93^ | No dsm/icd/cut off scale criteria |
| 1. Bechdolf 2012 ^94^ | No relevant outcome |
| 1. Becker 2010 ^95^ | No dsm/icd/cut off scale criteria |
| 1. Becker 2010 ^96^ | No relevant outcome |
| 1. Bedwell 2005 ^97^ | No relevant outcome |
| 1. Benavides 2018 ^98^ | No CHR-P population |
| 1. Ben-David 2014 ^99^ | No relevant outcome |
| 1. Ben-David 2019 ^100^ | No relevant outcome |
| 1. Ben-David 2019 ^101^ | No relevant outcome |
| 1. Bendfeldt 2015^102^ | No relevant outcome |
| 1. Benetti 2009 ^103^ | No relevant outcome |
| 1. Benetti 2013 ^104^ | No relevant outcome |
| 1. Benetti 2015 ^105^ | No relevant outcome |
| 1. Berger 2012 ^106^ | No relevant outcome |
| 1. Berger 2016 ^107^ | No relevant outcome |
| 1. Berger 2017 ^108^ | No relevant outcome |
| 1. Berger 2020 ^109^ | No relevant outcome |
| 1. Berman 2011 ^110^ | No relevant outcome |
| 1. Berna 2016 ^111^ | No CHR-P population |
| 1. Bernard 2014 ^112^ | No relevant outcome |
| 1. Bernard 2018 ^113^ | No relevant outcome |
| 1. Bernasconi 2015 ^114^ | No relevant outcome |
| 1. Berrocal 2021 ^115^ | No dsm/icd/cut off scale criteria |
| 1. Bertisch 2008 ^116^ | Study Design |
| 1. Bhattacharyya 2018 ^117^ | No relevant outcome |
| 1. Bhojraj 2009 ^118^ | No relevant outcome |
| 1. Birchwood 1989 ^119^ | No relevant outcome |
| 1. Bjornestad 2021 ^120^ | No CHR-P population |
| 1. Blanchard 2010 ^121^ | No relevant outcome |
| 1. Blasco 2020 ^122^ | No CHR-P population |
| 1. Blessing 2017 ^123^ | No dsm/icd/cut off scale criteria |
| 1. Bloemen 2010 ^124^ | No dsm/icd/cut off scale criteria |
| 1. Bloemen 2013 ^125^ | No relevant outcome |
| 1. Blomstrom 2014 ^126^ | No relevant outcome |
| 1. Bohner 2012 ^127^ | No CHR-P population |
| 1. Boldrini 2020 ^128^ | No relevant outcome |
| 1. Bolt 2019 ^129^ | No relevant outcome |
| 1. Borgwardt 2006 ^130^ | No relevant outcome |
| 1. Borgwardt 2007 ^131^ | No relevant outcome |
| 1. Borgwardt 2007 ^132^ | No dsm/icd/cut off scale criteria |
| 1. Borgwardt 2008 ^133^ | No CHR-P population |
| 1. Borgwardt 2008 ^134^ | No relevant outcome |
| 1. Borgwardt 2013 ^135^ | Study Design |
| 1. Bossong 2019 ^136^ | No relevant outcome |
| 1. Bourgin 2020 ^137^ | No dsm/icd/cut off scale criteria |
| 1. Bousman 2013 ^138^ | No dsm/icd/cut off scale criteria |
| 1. Braham 2014 ^139^ | No relevant outcome |
| 1. Bramon 2008 ^140^ | No relevant outcome |
| 1. Brandizzi 2014 ^141^ | No relevant outcome |
| 1. Brett 2015 ^142^ | No CHR-P population |
| 1. Brewer 2003 ^143^ | No relevant outcome |
| 1. Brewer 2012 ^144^ | No relevant outcome |
| 1. Brockhaus-Dumke 2005 ^145^ | No relevant outcome |
| 1. Brockhaus-Dumke 2008 ^146^ | No relevant outcome |
| 1. Brodey 2018 ^147^ | No relevant outcome |
| 1. Brodey 2018 ^148^ | No relevant outcome |
| 1. Brodey 2019 ^149^ | No relevant outcome |
| 1. Broome 2007 ^150^ | No relevant outcome |
| 1. Broome 2009 ^151^ | Study Design |
| 1. Broome 2010 ^152^ | No relevant outcome |
| 1. Broome 2010^153^ | No relevant outcome |
| 1. Broome 2012 ^154^ | No relevant outcome |
| 1. Brucato 2018 ^155^ | No dsm/icd/cut off scale criteria |
| 1. Brummitt 2013 ^156^ | No dsm/icd/cut off scale criteria |
| 1. [Brüne](https://pubmed.ncbi.nlm.nih.gov/?term=Br%C3%BCne+M&cauthor_id=21147235) 2011 ^157^ | No dsm/icd/cut off scale criteria |
| 1. Brüne 2019 ^158^ | No relevant outcome |
| 1. Bucci 2010 ^159^ | No dsm/icd/cut off scale criteria |
| 1. Buchy 2015 ^160^ | No dsm/icd/cut off scale criteria |
| 1. Buchy 2015 ^161^ | No relevant outcome |
| 1. Buchy 2015 ^162^ | No relevant outcome |
| 1. Buchy 2016^163^ | No relevant outcome |
| 1. Buehlmann 2010 ^164^ | No dsm/icd/cut off scale criteria |
| 1. Bukenaite 2017 ^165^ | No relevant outcome |
| 1. Burley 2009 ^166^ | No relevant outcome |
| 1. Burton 2019 ^167^ | No relevant outcome |
| 1. Büschlen 2011 ^168^ | No relevant outcome |
| 1. Byars 2014 ^169^ | No relevant outcome |
| 1. Bykowsky 2019 ^170^ | Study Design |
| 1. Byrne 2010 ^171^ | No dsm/icd/cut off scale criteria |
| 1. Byrne 2014 ^172^ | No relevant outcome |
| 1. Byrne 2019 ^173^ | No relevant outcome |
| 1. Byun 2012 ^174^ | No relevant outcome |
| 1. Cadenhead 2005 ^175^ | No relevant outcome |
| 1. Cadenhead 2010 ^176^ | No dsm/icd/cut off scale criteria |
| 1. Cadenhead 2011 ^177^ | No relevant outcome |
| 1. Cadenhead 2019 ^178^ | No relevant outcome |
| 1. Caldani 2017 ^179^ | No relevant outcome |
| 1. Caldani 2017 ^180^ | No relevant outcome |
| 1. Calkins 2021 ^181^ | No relevant outcome |
| 1. Callaway 2014 ^182^ | No relevant outcome |
| 1. Campanella 2014 ^183^ | No relevant outcome |
| 1. Campion 2019 ^184^ | Study Design |
| 1. Can 2019 ^185^ | No relevant outcome |
| 1. Cannon 2002 ^186^ | No CHR-P population |
| 1. Cannon 2008 ^187^ | No CHR-P population |
| 1. Cannon 2015 ^188^ | No relevant outcome |
| 1. Cannon 2016 ^189^ | No relevant outcome |
| 1. Cao 2018 ^190^ | No relevant outcome |
| 1. Cao 2019 ^191^ | No relevant outcome |
| 1. Caravaggio 2017 ^192^ | No relevant outcome |
| 1. Carberry 1982 ^193^ | No relevant outcome |
| 1. Carey 2021 ^194^ | No CHR-P population |
| 1. Carletti 2012 ^195^ | Study Design |
| 1. Carney 2017 ^196^ | No relevant outcome |
| 1. Carney 2017 ^197^ | Study Design |
| 1. Carney 2017 ^198^ | Study Design |
| 1. Carney 2018 ^199^ | No dsm/icd/cut off scale criteria |
| 1. Carol 2014 ^200^ | Study Design |
| 1. Carol 2018 ^201^ | Study Design |
| 1. Carpenter 2018 ^202^ | Study Design |
| 1. Carrion 2011 ^203^ | Study Design |
| 1. Carrión 2015 ^204^ | No relevant outcome |
| 1. Carrion 2016 ^205^ | No relevant outcome |
| 1. Carrión 2019 ^206^ | No relevant outcome |
| 1. Carroll 2004 ^207^ | No dsm/icd/cut off scale criteria |
| 1. Castle 2012 ^208^ | Study Design |
| 1. Castro 2015 ^209^ | Study Design |
| 1. Catalan 2020 ^210^ | No relevant outcome |
| 1. Chan 2017 ^211^ | No relevant outcome |
| 1. Chan 2018 ^212^ | Study Design |
| 1. Chan 2019 ^213^ | No relevant outcome |
| 1. Chan 2021 ^214^ | No relevant outcome |
| 1. Chang 2018 ^215^ | No relevant outcome |
| 1. Chang 2019 ^216^ | No dsm/icd/cut off scale criteria |
| 1. Chapman 1980 ^217^ | No relevant outcome |
| 1. Chapman 1980 ^218^ | No CHR-P population |
| 1. Chapman 1994 ^219^ | No CHR-P population |
| 1. Chapman 2020 ^220^ | No CHR-P population |
| 1. Chaumette 2016 ^221^ | No relevant outcome |
| 1. Chaumette 2019 ^222^ | Study Design |
| 1. Chaumette 2020 ^223^ | No relevant outcome |
| 1. Chen 2014 ^224^ | No relevant outcome |
| 1. Chen 2016 ^225^ | No dsm/icd/cut off scale criteria |
| 1. Chen 2016 ^226^ | No relevant outcome |
| 1. Chen 2019 ^227^ | No relevant outcome |
| 1. Chen 2021 ^228^ | No relevant outcome |
| 1. Cho 2016 ^229^ | No relevant outcome |
| 1. Choi 2008 ^230^ | No relevant outcome |
| 1. Choi 2012 ^231^ | No dsm/icd/cut off scale criteria |
| 1. Choi 2017 ^232^ | No relevant outcome |
| 1. Choi 2017 ^233^ | No relevant outcome |
| 1. Chon 2015 ^234^ | No relevant outcome |
| 1. Chu 2019 ^235^ | No relevant outcome |
| 1. Chudleigh 2011 ^236^ | No relevant outcome |
| 1. Chung 2008 ^237^ | No dsm/icd/cut off scale criteria |
| 1. Chung 2013 ^238^ | No dsm/icd/cut off scale criteria |
| 1. Chung 2018 ^239^ | No dsm/icd/cut off scale criteria |
| 1. Chung 2019 ^240^ | No relevant outcome |
| 1. Ciarleglio 2019 ^241^ | No relevant outcome |
| 1. Cicero 2014 ^242^ | No relevant outcome |
| 1. Clamor 2014 ^243^ | No relevant outcome |
| 1. Clark 1989 ^244^ | No CHR-P population |
| 1. Clark 2016 ^245^ | No CHR-P population |
| 1. Clark 2018 ^246^ | No dsm/icd/cut off scale criteria |
| 1. Clay 2020 ^247^ | No relevant outcome |
| 1. Clayson 2019 ^248^ | No relevant outcome |
| 1. Cocchi 2013 ^249^ | No relevant outcome |
| 1. Cocchi 2015 ^250^ | No relevant outcome |
| 1. Codjoe 2013 ^251^ | No relevant outcome |
| 1. Cohen 2019 ^252^ | No relevant outcome |
| 1. Colibazzi 2016 ^253^ | No CHR-P population |
| 1. Collin 2020 ^254^ | No relevant outcome |
| 1. Collin 2020 ^255^ | No relevant outcome |
| 1. Collip 2013 ^256^ | No relevant outcome |
| 1. Comparelli 2014 ^257^ | No CHR-P population |
| 1. Comparelli 2011 ^258^ | No dsm/icd/cut off scale criteria |
| 1. Comparelli 2013 ^259^ | No relevant outcome |
| 1. Comparelli 2013 ^260^ | No dsm/icd/cut off scale criteria |
| 1. Comparelli 2013 ^261^ | No relevant outcome |
| 1. Cooper 2014 ^262^ | No CHR-P population |
| 1. Cooper 2018 ^263^ | Study Design |
| 1. Cooper 2018 ^264^ | No CHR-P population |
| 1. Copolov 2000 ^265^ | No relevant outcome |
| 1. Corcoran 2012 ^266^ | No relevant outcome |
| 1. Corcoran 2015 ^267^ | No dsm/icd/cut off scale criteria |
| 1. Cordes 2017 ^268^ | No relevant outcome |
| 1. Corigliano 2014 ^269^ | No relevant outcome |
| 1. Cornblatt 2003 ^270^ | No dsm/icd/cut off scale criteria |
| 1. Cornblatt 2007 ^271^ | No relevant outcome |
| 1. Correll 2005 ^272^ | No relevant outcome |
| 1. Correll 2007 ^273^ | No CHR-P population |
| 1. Correll 2008 ^274^ | No CHR-P population |
| 1. Corsi-Zuelli 2020 ^275^ | No CHR-P population |
| 1. Costello 2012 ^276^ | No CHR-P population |
| 1. Cotter 2017 ^277^ | Article not found |
| 1. Cotter 2019 ^278^ | No dsm/icd/cut off scale criteria |
| 1. Counotte 2017 ^279^ | No dsm/icd/cut off scale criteria |
| 1. Counotte 2018 ^280^ | No relevant outcome |
| 1. Counotte 2019 ^281^ | No dsm/icd/cut off scale criteria |
| 1. Couture 2008 ^282^ | No relevant outcome |
| 1. Cowan 2021 ^283^ | No relevant outcome |
| 1. Cropley 2016 ^284^ | No relevant outcome |
| 1. Crossley 2009 ^285^ | No dsm/icd/cut off scale criteria |
| 1. Crump 2018 ^286^ | No relevant outcome |
| 1. Cui 2020 ^287^ | No relevant outcome |
| 1. Cullen 2010 ^288^ | No relevant outcome |
| 1. Cullen 2013 ^289^ | No CHR-P population |
| 1. Cullen 2014 ^290^ | No CHR-P population |
| 1. Cullen 2020 ^291^ | No relevant outcome |
| 1. Da Silva 2018 ^292^ | No relevant outcome |
| 1. Da Silva 2018 ^293^ | No relevant outcome |
| 1. Da Silva 2019 ^294^ | No relevant outcome |
| 1. Dal Mas 2019 ^295^ | No relevant outcome |
| 1. Damme 2019 ^296^ | No relevant outcome |
| 1. Damme 2019 ^297^ | No relevant outcome |
| 1. Damme 2019 ^298^ | No relevant outcome |
| 1. Damme 2020 ^299^ | Study Design |
| 1. Daneault 2019 ^300^ | No relevant outcome |
| 1. D'Angelo 2017 ^301^ | No CHR-P population |
| 1. D'Angelo 2019 ^302^ | No relevant outcome |
| 1. Dannevang 2018 ^303^ | No relevant outcome |
| 1. Darrell-Berry 2017 ^304^ | No dsm/icd/cut off scale criteria |
| 1. Das 2018 ^305^ | No dsm/icd/cut off scale criteria |
| 1. Das-Munshi 2012 ^306^ | No relevant outcome |
| 1. Davidsen 2009 ^307^ | No CHR-P population |
| Davidsen 2009^308^ | No CHR-P population |
| 1. Davidsen 2012 ^309^ | No relevant outcome |
| 1. Davidson 2018 ^310^ | No dsm/icd/cut off scale criteria |
| 1. Davies 2019 ^311^ | No relevant outcome |
| 1. Davies 2019 ^312^ | No dsm/icd/cut off scale criteria |
| 1. Day 2014 ^313^ | No relevant outcome |
| Dazzan 2012^314^ | No dsm/icd/cut off scale criteria |
| 1. De Koning 2014 ^315^ | No relevant outcome |
| 1. de la Fuente-Sandoval 2011 ^316^ | No relevant outcome |
| 1. de la Fuente-Sandoval 2013 ^317^ | No relevant outcome |
| 1. de la Fuente-Sandoval 2016 ^318^ | No relevant outcome |
| 1. de la Serna 2010 ^319^ | No relevant outcome |
| 1. de la Serna 2011 ^320^ | No CHR-P population |
| 1. de Wit 2017 ^321^ | No CHR-P population |
| 1. Dean 2013 ^322^ | No relevant outcome |
| 1. Dean 2013 ^323^ | No relevant outcome |
| 1. Dean 2015 ^324^ | Study Design |
| 1. Dean 2016 ^325^ | No relevant outcome |
| 1. Dean 2017 ^326^ | No relevant outcome |
| 1. Dean 2018 ^327^ | No relevant outcome |
| 1. Dean 2018 ^328^ | No relevant outcome |
| 1. Debbané 2010 ^329^ | No relevant outcome |
| Debbané 2012^330^ | No CHR-P population |
| 1. Deighton 2015 ^331^ | No CHR-P population |
| 1. Deighton 2016 ^332^ | No relevant outcome |
| 1. Del Re 2014 ^333^ | No relevant outcome |
| 1. del Re 2015 ^334^ | No relevant outcome |
| 1. Delaney 2019 ^335^ | No relevant outcome |
| 1. Delevoye-Turrell 2012 ^336^ | No relevant outcome |
| 1. Demars 2020 ^337^ | No relevant outcome |
| 1. Demjaha 2012 ^338^ | No relevant outcome |
| 1. Demjaha 2017 ^339^ | No dsm/icd/cut off scale criteria |
| 1. Demro 2017 ^340^ | No relevant outcome |
| 1. Devoe 2021 ^341^ | No dsm/icd/cut off scale criteria |
| 1. DeVylder 2013 ^342^ | No relevant outcome |
| 1. DeVylder 2012 ^343^ | No CHR-P population |
| 1. DeVylder 2013 ^344^ | No dsm/icd/cut off scale criteria |
| 1. DeVylder 2013 ^345^ | No dsm/icd/cut off scale criteria |
| 1. DeVylder 2014 ^346^ | No dsm/icd/cut off scale criteria |
| 1. DeVylder 2014a ^347^ | No relevant outcome |
| 1. DeVylder 2014b ^348^ | Study Design |
| 1. Di Biase 2017 ^349^ | No relevant outcome |
| 1. Dickson 2014 ^350^ | No relevant outcome |
| 1. Dickson 2014 ^351^ | No CHR-P population |
| 1. Dishy 2020 ^352^ | No CHR-P population |
| 1. Diwadkar 2011 ^353^ | No relevant outcome |
| 1. Diwadkar 2011 ^354^ | No CHR-P population |
| 1. Diwadkar 2012 ^355^ | No CHR-P population |
| 1. Dodell-Feder 2014 ^356^ | No CHR-P population |
| 1. Domingues 2011 ^357^ | No CHR-P population |
| 1. Dominguez-Martinez 2014 ^358^ | No CHR-P population |
| 1. Dominguez-Martinez 2015 ^359^ | No dsm/icd/cut off scale criteria |
| 1. Dominguez-Martinez 2017 ^360^ | No relevant outcome |
| 1. Donkers 2011 ^361^ | No CHR-P population |
| 1. Dragt 2011 ^362^ | No CHR-P population |
| 1. Du 2018 ^363^ | No relevant outcome |
| 1. Du 2018 ^364^ | No relevant outcome |
| 1. Duffy 2015 ^365^ | No relevant outcome |
| 1. Dukart 2017 ^366^ | No relevant outcome |
| 1. Eack 2010 ^367^ | No relevant outcome |
| 1. Egerton 2013 ^368^ | No relevant outcome |
| 1. Egerton 2014 ^369^ | No dsm/icd/cut off scale criteria |
| 1. Egerton 2016 ^370^ | No relevant outcome |
| 1. Egerton 2017 ^371^ | No relevant outcome |
| 1. Eggins 2018 ^372^ | No relevant outcome |
| 1. Egloff 2018 ^373^ | No dsm/icd/cut off scale criteria |
| 1. Egloff 2018 ^374^ | No relevant outcome |
| 1. Egloff 2019 ^375^ | No relevant outcome |
| 1. Eisenacher 2015 ^376^ | No relevant outcome |
| 1. Eisenacher 2016 ^377^ | No relevant outcome |
| 1. Eisenacher 2018 ^378^ | No dsm/icd/cut off scale criteria |
| 1. Ereshefsky 2020 ^379^ | No dsm/icd/cut off scale criteria |
| 1. Ermakova 2018 ^380^ | Study Design |
| 1. Eslami 2011 ^381^ | No dsm/icd/cut off scale criteria |
| 1. Esterberg 2012 ^382^ | No relevant outcome |
| 1. Esterberg 2013 ^383^ | No CHR-P population |
| 1. Esterberg 2013 ^384^ | No CHR-P population |
| 1. Falkenberg 2015 ^385^ | No CHR-P population |
| 1. Falkenberg 2017 ^386^ | No relevant outcome |
| 1. Falukozi 2012 ^387^ | No dsm/icd/cut off scale criteria |
| 1. Farrell 2002 ^388^ | No relevant outcome |
| 1. Feng 2019 ^389^ | No CHR-P population |
| 1. Fernandez 2018 ^390^ | No relevant outcome |
| 1. Fluckiger 2019 ^391^ | No relevant outcome |
| 1. Flynn 2012 ^392^ | No relevant outcome |
| 1. Föcking 2016 ^393^ | No dsm/icd/cut off scale criteria |
| 1. Fonseca-Pedrero 2014 ^394^ | No relevant outcome |
| 1. Fonseca-Pedrero 2011 ^395^ | No CHR-P population |
| 1. Fornito 2008 ^396^ | No CHR-P population |
| 1. Freeman 2007 ^397^ | No dsm/icd/cut off scale criteria |
| 1. French 2007 ^398^ | No dsm/icd/cut off scale criteria |
| 1. French 2012 ^399^ | No dsm/icd/cut off scale criteria |
| 1. Fresàn 2007 ^400^ | No dsm/icd/cut off scale criteria |
| 1. Fresàn 2015 ^401^ | Article not found |
| 1. Fridgen 2013 ^402^ | No relevant outcome |
| 1. Friedman-Yakoobian 2020 ^403^ | No relevant outcome |
| 1. Friedman-Yakoobian 2019 ^404^ | No relevant outcome |
| 1. Friesen 2016 ^405^ | No relevant outcome |
| 1. Frommann 2008 ^406^ | No CHR-P population |
| 1. Frommann 2011 ^407^ | No relevant outcome |
| 1. Frumin 2002 ^408^ | No CHR-P population |
| 1. Fryer 2013 ^409^ | No CHR-P population |
| 1. Fryer 2016 ^410^ | No relevant outcome |
| 1. Fryer 2019 ^411^ | No relevant outcome |
| 1. Fryer 2020 ^412^ | No relevant outcome |
| 1. Fulford 2014 ^413^ | No relevant outcome |
| 1. Fulford 2013 ^414^ | No relevant outcome |
| 1. Fusar Poli 2016 ^415^ | No dsm/icd/cut off scale criteria |
| 1. Fusar-Poli 2012 ^416^ | No relevant outcome |
| 1. Fusar-Poli 2009 ^417^ | No dsm/icd/cut off scale criteria |
| Fusar-Poli 2009^418^ | No relevant outcome |
| 1. Fusar-Poli 2010 ^419^ | No relevant outcome |
| 1. Fusar-Poli 2010 ^420^ | No relevant outcome |
| 1. Fusar-Poli 2011 ^421^ | No relevant outcome |
| 1. Fusar-Poli 2011 ^422^ | No relevant outcome |
| 1. Fusar-Poli 2011 ^423^ | No relevant outcome |
| 1. Fusar-Poli 2011 ^424^ | No relevant outcome |
| 1. Fusar-Poli 2011 ^425^ | No dsm/icd/cut off scale criteria |
| 1. Fusar-Poli 2011 ^426^ | No relevant outcome |
| Fusar-Poli 2011^427^ | No relevant outcome |
| 1. Fusar-Poli 2012 ^428^ | No relevant outcome |
| 1. Fusar-Poli 2014 ^429^ | Study Design |
| 1. Fusar-Poli 2017 ^430^ | Study Design |
| 1. Fusar-Poli 2018 ^431^ | No relevant outcome |
| 1. Fusar-Poli 2018 ^432^ | No relevant outcome |
| 1. Fusar-Poli 2018 ^433^ | No relevant outcome |
| 1. Fusar-Poli 2019 ^434^ | No relevant outcome |
| 1. Fusar-Poli 2019 ^435^ | Study Design |
| 1. Fusar-Poli 2020 ^436^ | No CHR-P population |
| 1. Gaag 2019 ^437^ | No relevant outcome |
| 1. Gajwani 2013 ^438^ | No relevant outcome |
| 1. Galletly 2011 ^439^ | No dsm/icd/cut off scale criteria |
| 1. Garyfallos 2011 ^440^ | No CHR-P population |
| 1. Gattere 2018 ^441^ | Study Design |
| 1. Gaudiano 2013 ^442^ | No relevant outcome |
| 1. Gawęda 2018 ^443^ | No CHR-P population |
| 1. Gee 2012 ^444^ | No relevant outcome |
| 1. Gee 2016 ^445^ | No relevant outcome |
| 1. Georgopoulos 2019 ^446^ | No relevant outcome |
| 1. Geraets 2018 ^447^ | No relevant outcome |
| 1. Gerritsen 2019 ^448^ | No dsm/icd/cut off scale criteria |
| 1. Gerson 2011 ^449^ | No relevant outcome |
| 1. Gerstenberg 2015 ^450^ | No CHR-P population |
| 1. Gibson 2010 ^451^ | Article not found |
| 1. Gibson 2014 ^452^ | No CHR-P population |
| 1. Gifford 2021 ^453^ | No CHR-P population |
| 1. Gill 2015 ^454^ | No relevant outcome |
| 1. Gill 2016 ^455^ | No relevant outcome |
| 1. Gleeson 2005 ^456^ | No relevant outcome |
| 1. Glenthøj 2019 ^457^ | No CHR-P population |
| 1. Glenthøj 2019 ^458^ | No relevant outcome |
| 1. Goghari 2014 ^459^ | No dsm/icd/cut off scale criteria |
| 1. Goines 2019^460^ | No relevant outcome |
| 1. Goines 2019 ^461^ | No dsm/icd/cut off scale criteria |
| 1. Goldenberg 2012 ^462^ | No relevant outcome |
| 1. Goldsmith 2019 ^463^ | No relevant outcome |
| Goldstein 2010^464^ | No dsm/icd/cut off scale criteria |
| 1. Goldstein 2011 ^465^ | No CHR-P population |
| 1. Golembo-Smith 2014 ^466^ | No CHR-P population |
| 1. Gonçalves·2012 ^467^ | No relevant outcome |
| 1. Gonzalez-Heydrich 2015 ^468^ | No relevant outcome |
| 1. Gonzalez-Heydrich 2016 ^469^ | No relevant outcome |
| 1. Gonzalez-Rodriguez 2014 ^470^ | No relevant outcome |
| 1. Gooding 2005 ^471^ | No dsm/icd/cut off scale criteria |
| 1. Gothelf 2014 ^472^ | No CHR-P population |
| 1. Gothelf 2011 ^473^ | No CHR-P population |
| 1. Gottlieb 2004 ^474^ | No CHR-P population |
| 1. Goulding 2016 ^475^ | No relevant outcome |
| 1. Gourzis 2002 ^476^ | No relevant outcome |
| 1. Gouzoulis-Mayfrank 2007 ^477^ | No CHR-P population |
| 1. Graf von 2014 ^478^ | No CHR-P population |
| 1. Gran[ö](https://pubmed.ncbi.nlm.nih.gov/?term=Gran%C3%B6+N&cauthor_id=32878386) 2013 ^479^ | No relevant outcome |
| 1. Gran[ö](https://pubmed.ncbi.nlm.nih.gov/?term=Gran%C3%B6+N&cauthor_id=32878386) 2014 ^480^ | No dsm/icd/cut off scale criteria |
| 1. Granö 2009 ^481^ | No dsm/icd/cut off scale criteria |
| 1. [Granö](https://pubmed.ncbi.nlm.nih.gov/?term=Gran%C3%B6+N&cauthor_id=20465513) 2011 ^482^ | No dsm/icd/cut off scale criteria |
| 1. Granö 2011 ^483^ | No dsm/icd/cut off scale criteria |
| 1. [Granö](https://pubmed.ncbi.nlm.nih.gov/?term=Gran%C3%B6+N&cauthor_id=21545689) 2011 ^484^ | No dsm/icd/cut off scale criteria |
| 1. Gran[ö](https://pubmed.ncbi.nlm.nih.gov/?term=Gran%C3%B6+N&cauthor_id=23485127) 2013 ^485^ | No CHR-P population |
| 1. Gran[ö](https://pubmed.ncbi.nlm.nih.gov/?term=Gran%C3%B6+N&cauthor_id=23126455) 2013 ^486^ | No relevant outcome |
| 1. Gran[ö](https://pubmed.ncbi.nlm.nih.gov/?term=Gran%C3%B6+N&cauthor_id=32878386) 2014 ^487^ | No dsm/icd/cut off scale criteria |
| 1. Granö^488^ 2016 | No dsm/icd/cut off scale criteria |
| 1. Green 2011^489^ | No CHR-P population |
| 1. Green 2012 ^490^ | No relevant outcome |
| 1. Greenhalgh 2017 ^491^ | No relevant outcome |
| 1. Greenland-White 2017 ^492^ | No relevant outcome |
| 1. Grent-'t-Jong 2018 ^493^ | No relevant outcome |
| 1. Grossman 2020 ^494^ | No dsm/icd/cut off scale criteria |
| 1. Gruber 2018 ^495^ | No dsm/icd/cut off scale criteria |
| 1. Gschwandtner 2003 ^496^ | No dsm/icd/cut off scale criteria |
| 1. Gschwandtner 2006 ^497^ | No relevant outcome |
| 1. Gschwandtner 2009 ^498^ | No CHR-P population |
| 1. Gudlowski 2009 ^499^ | No relevant outcome |
| 1. Guma 2017 ^500^ | No relevant outcome |
| 1. Gunnell 2002 ^501^ | No relevant outcome |
| 1. Guo 2020 ^502^ | No CHR-P population |
| 1. Gupta 2014 ^503^ | No relevant outcome |
| 1. Gupta 2016 ^504^ | No relevant outcome |
| 1. Gupta 2018 ^505^ | No relevant outcome |
| 1. Gupta 2019^506^ | No relevant outcome |
| 1. Gupta 2020 ^507^ | No dsm/icd/cut off scale criteria |
| 1. Gur 2014 ^508^ | Article not found |
| 1. Gur 2015 ^509^ | No CHR-P population |
| 1. Gureje 1994 ^510^ | No relevant outcome |
| 1. Haarsma 2020 ^511^ | No CHR-P population |
| 1. Haas 2020 ^512^ | No dsm/icd/cut off scale criteria |
| 1. Hafizi 2017 ^513^ | No relevant outcome |
| 1. Hafizi 2018 ^514^ | No dsm/icd/cut off scale criteria |
| 1. Hafizi 2018 ^515^ | No relevant outcome |
| 1. Hagenmuller 2014 ^516^ | No dsm/icd/cut off scale criteria |
| 1. Hagenmuller 2019^517^ | No dsm/icd/cut off scale criteria |
| 1. Haidl 2018 ^518^ | No relevant outcome |
| Haining 2021^519^ | No relevant outcome |
| 1. Haller 2009 ^520^ | No relevant outcome |
| 1. Hamaie 2016 ^521^ | No relevant outcome |
| 1. Hambrecht 2002 ^522^ | No dsm/icd/cut off scale criteria |
| 1. Hamilton 2019 ^523^ | No CHR-P population |
| 1. Hamilton 2019 ^524^ | No relevant outcome |
| 1. Hampton 2018 ^525^ | No relevant outcome |
| 1. Han 2012 ^526^ | No relevant outcome |
| 1. Hannan 2010 ^527^ | No relevant outcome |
| 1. Harley 2010 ^528^ | No relevant outcome |
| 1. Harrisberger 2016 ^529^ | No CHR-P population |
| 1. Harrisberger 2018 ^530^ | No relevant outcome |
| 1. Hartmann 2017 ^531^ | No dsm/icd/cut off scale criteria |
| 1. Hartmann 2019 ^532^ | No dsm/icd/cut off scale criteria |
| 1. Hartmann 2020 ^533^ | No dsm/icd/cut off scale criteria |
| 1. Hasan 2012 ^534^ | No dsm/icd/cut off scale criteria |
| 1. Hauser 2009 ^535^ | No relevant outcome |
| 1. Hauser 2011 ^536^ | No relevant outcome |
| 1. Haut 2015 ^537^ | No relevant outcome |
| 1. Hawkins 2004 ^538^ | No relevant outcome |
| 1. Hawkins 2008 ^539^ | No dsm/icd/cut off scale criteria |
| 1. He 2018 ^540^ | No relevant outcome |
| 1. He 2019 ^541^ | No relevant outcome |
| 1. He 2019 ^542^ | No relevant outcome |
| 1. Healey 2013 ^543^ | Study Design |
| 1. Healey 2018 ^544^ | No relevant outcome |
| 1. Hechtman 2019 ^545^ | No dsm/icd/cut off scale criteria |
| 1. Heinimaa 2003 ^546^ | Study Design |
| 1. Heinze 2015 ^547^ | No dsm/icd/cut off scale criteria |
| 1. Heinze 2018 ^548^ | No relevant outcome |
| 1. Heitz 2019 ^549^ | No dsm/icd/cut off scale criteria |
| 1. Heitz 2019 ^550^ | No relevant outcome |
| 1. Hengartner 2017 ^551^ | No dsm/icd/cut off scale criteria |
| 1. Hermans 2020 ^552^ | No dsm/icd/cut off scale criteria |
| 1. Hickey 2020 ^553^ | No relevant outcome |
| 1. Hickie 2013 ^554^ | Study Design |
| Higuchi 2013^555^ | No CHR-P population |
| 1. Higuchi 2014 ^556^ | No relevant outcome |
| 1. Hlastala 2005 ^557^ | No relevant outcome |
| 1. Ho 2007 ^558^ | No CHR-P population |
| 1. Hodgekins 2015 ^559^ | No relevant outcome |
| 1. Hoffman 2007 ^560^ | No relevant outcome |
| 1. Hollis 2008 ^561^ | No dsm/icd/cut off scale criteria |
| 1. Holtzman 2016 ^562^ | No CHR-P population |
| 1. Holzer 2005 ^563^ | No relevant outcome |
| 1. Holzer 2014 ^564^ | Study Design |
| 1. Hong 2015 ^565^ | No relevant outcome |
| 1. Hooker 2014 ^566^ | No relevant outcome |
| 1. Hopkinson 1965 ^567^ | Study Design |
| 1. Hoptman 2008 ^568^ | No CHR-P population |
| 1. Hou 2016 ^569^ | No CHR-P population |
| 1. Howes 2009 ^570^ | No relevant outcome |
| 1. Howes 2011 ^571^ | No dsm/icd/cut off scale criteria |
| 1. Howes 2020 ^572^ | No relevant outcome |
| 1. Hsieh 2012 ^573^ | No relevant outcome |
| 1. Hua 2019 ^574^ | No relevant outcome |
| 1. Huang 2007 ^575^ | No relevant outcome |
| 1. Huang 2019 ^576^ | No dsm/icd/cut off scale criteria |
| 1. Huber 2014 ^577^ | No dsm/icd/cut off scale criteria |
| 1. Huber 2018 ^578^ | No relevant outcome |
| 1. Hur 2012 ^579^ | No dsm/icd/cut off scale criteria |
| 1. Hur 2013 ^580^ | No dsm/icd/cut off scale criteria |
| 1. Hur 2015 ^581^ | No relevant outcome |
| 1. Hurlemann 2005 ^582^ | No relevant outcome |
| 1. Hurlemann 2008 ^583^ | No dsm/icd/cut off scale criteria |
| 1. Hurlemann 2008 ^584^ | No relevant outcome |
| 1. Hurtig 2011 ^585^ | No dsm/icd/cut off scale criteria |
| 1. Hutton 2011 ^586^ | No CHR-P population |
| 1. Hutton 2012 ^587^ | No dsm/icd/cut off scale criteria |
| 1. Hӓfner 2004 ^588^ | No relevant outcome |
| 1. Iorfino 2019 ^589^ | Study Design |
| 1. Ising 2012 ^590^ | No CHR-P population |
| 1. Ising 2015 ^591^ | No CHR-P population |
| 1. Ising 2016 ^592^ | No relevant outcome |
| 1. Ising 2017 ^593^ | No relevant outcome |
| 1. Ittig 2015 ^594^ | No relevant outcome |
| 1. Ittig 2017 ^595^ | No relevant outcome |
| 1. Iwashiro 2012 ^596^ | No dsm/icd/cut off scale criteria |
| 1. Iwashiro 2016 ^597^ | No relevant outcome |
| 1. Iyer 2018 ^598^ | No relevant outcome |
| 1. Izon 2021 ^599^ | No CHR-P population |
| 1. Jabben 2007 ^600^ | No dsm/icd/cut off scale criteria |
| Jablensky 1997^601^ | No CHR-P population |
| 1. Jacobson 2010 ^602^ | No CHR-P population |
| 1. Jagannath 2017 ^603^ | No relevant outcome |
| 1. Jagannath 2018 ^604^ | No dsm/icd/cut off scale criteria |
| 1. Jagannath 2020 ^605^ | No relevant outcome |
| 1. Jahchan 2011 ^606^ | No dsm/icd/cut off scale criteria |
| 1. Jahshan 2010 ^607^ | No relevant outcome |
| 1. Jahshan 2012 ^608^ | No relevant outcome |
| 1. Jalbrzikowski 2012 ^609^ | No relevant outcome |
| 1. Jalbrzikowski 2013 ^610^ | No relevant outcome |
| 1. Jalbrzikowski 2014 ^611^ | No relevant outcome |
| 1. Jalbrzikowski 2014 ^612^ | No relevant outcome |
| 1. Jang 2011 ^613^ | No relevant outcome |
| 1. Jarrett 2012 ^614^ | No dsm/icd/cut off scale criteria |
| 1. Jarrett 2016 ^615^ | No dsm/icd/cut off scale criteria |
| 1. Jarrett 2016 ^616^ | No relevant outcome |
| 1. Jeffries 2016 ^617^ | No dsm/icd/cut off scale criteria |
| 1. Jessen 2006 ^618^ | No relevant outcome |
| 1. Jhung 2013 ^619^ | No relevant outcome |
| 1. Jia 2015 ^620^ | No dsm/icd/cut off scale criteria |
| 1. Jimeno 2020 ^621^ | No relevant outcome |
| 1. Jin 2020 ^622^ | No relevant outcome |
| 1. Johns 2010 ^623^ | No relevant outcome |
| 1. Jongeneel 2018 ^624^ | No dsm/icd/cut off scale criteria |
| 1. Juckel 2012 ^625^ | No relevant outcome |
| 1. Jukuri 2013 ^626^ | No relevant outcome |
| 1. Jung 2008 ^627^ | No CHR-P population |
| 1. Jung 2012 ^628^ | No CHR-P population |
| 1. Jutla 2020 ^629^ | No dsm/icd/cut off scale criteria |
| 1. Kafadar 2020 ^630^ | Study Design |
| 1. Kafali 2019 ^631^ | No relevant outcome |
| 1. Kalin 2020 ^632^ | No CHR-P population |
| 1. Kalin 2020 ^633^ | Study Design |
| 1. Kamath 2012 ^634^ | Study Design |
| 1. Kamath 2014 ^635^ | No relevant outcome |
| 1. Kang 2012 ^636^ | No relevant outcome |
| 1. Kang 2014 ^637^ | No CHR-P population |
| 1. Karanikas 2016 ^638^ | No dsm/icd/cut off scale criteria |
| 1. Karanikas 2017 ^639^ | No relevant outcome |
| 1. Karcher 2019 ^640^ | No relevant outcome |
| 1. Karcher 2019 ^641^ | No relevant outcome |
| 1. Karlsgodt 2019 ^642^ | No relevant outcome |
| 1. Katagiri 2015 ^643^ | Study Design |
| 1. Katagiri 2018 ^644^ | No relevant outcome |
| 1. Katagiri 2019 ^645^ | No relevant outcome |
| 1. Kates 2011 ^646^ | No relevant outcome |
| 1. Katsura 2014 ^647^ | No CHR-P population |
| 1. Kayser 2013 ^648^ | No dsm/icd/cut off scale criteria |
| 1. Kayser 2014 ^649^ | No relevant outcome |
| 1. Kebir 2018 ^650^ | No relevant outcome |
| 1. Keefe 2020 ^651^ | No relevant outcome |
| 1. Keefe 2006 ^652^ | No relevant outcome |
| 1. Kegeles 2020 ^653^ | No relevant outcome |
| 1. Kelleher 2012 ^654^ | No relevant outcome |
| 1. Kelleher 2013 ^655^ | No CHR-P population |
| 1. Kelleher 2014 ^656^ | No relevant outcome |
| 1. Kéri 2007 ^657^ | No CHR-P population |
| Kéri 2008^658^ | No relevant outcome |
| 1. Keri 2009 ^659^ | No relevant outcome |
| 1. Kerns 2000 ^660^ | No relevant outcome |
| 1. Keshavan 2009 ^661^ | No CHR-P population |
| 1. Keshavan 2009 ^662^ | No relevant outcome |
| 1. Kim 2014 ^663^ | No relevant outcome |
| 1. Kim 2010 ^664^ | No relevant outcome |
| 1. Kim 2011 ^665^ | No dsm/icd/cut off scale criteria |
| 1. Kim 2011 ^666^ | No dsm/icd/cut off scale criteria |
| 1. Kim 2012 ^667^ | No dsm/icd/cut off scale criteria |
| 1. Kim 2015 ^668^ | No CHR-P population |
| 1. Kim 2016 ^669^ | No relevant outcome |
| 1. Kim 2017 ^670^ | No dsm/icd/cut off scale criteria |
| 1. Kim 2018 ^671^ | No dsm/icd/cut off scale criteria |
| 1. Kim 2018 ^672^ | No relevant outcome |
| 1. Kimhy 2014 ^673^ | No relevant outcome |
| 1. Kimhy 2007 ^674^ | No relevant outcome |
| 1. Kimhy 2016 ^675^ | No relevant outcome |
| 1. Kindler 2019 ^676^ | No relevant outcome |
| 1. Kirkbride 2015 ^677^ | No relevant outcome |
| 1. Kiss 2012 ^678^ | No relevant outcome |
| 1. Klaassen 2011 ^679^ | Study Design |
| 1. Kleineidam 2019 ^680^ | No dsm/icd/cut off scale criteria |
| 1. Kline 2012^681^ | No dsm/icd/cut off scale criteria |
| 1. Kline 2013 ^682^ | No relevant outcome |
| 1. Kline 2014 ^683^ | No relevant outcome |
| 1. Kline 2016 ^684^ | No relevant outcome |
| 1. Kline 2016 ^685^ | No relevant outcome |
| 1. Klippel 2017 ^686^ | No relevant outcome |
| 1. Klosterkotter 1996 ^687^ | No relevant outcome |
| 1. Klosterkotter 2000 ^688^ | No relevant outcome |
| 1. Kobayashi 2008 ^689^ | Article not in English |
| 1. Kobayashi 2009 ^690^ | No relevant outcome |
| 1. Koethe 2006 ^691^ | No relevant outcome |
| 1. Koethe 2009 ^692^ | No relevant outcome |
| 1. Koethe 2009 ^693^ | No dsm/icd/cut off scale criteria |
| 1. Koh 2011 ^694^ | No relevant outcome |
| 1. Kohler 2007 ^695^ | No relevant outcome |
| 1. Kohler 2014 ^696^ | No CHR-P population |
| 1. Koike 2011 ^697^ | No dsm/icd/cut off scale criteria |
| 1. Koike 2013 ^698^ | No relevant outcome |
| 1. Koike 2017 ^699^ | No dsm/icd/cut off scale criteria |
| 1. Koivukangas 2010 ^700^ | No relevant outcome |
| 1. Kollias 2020 ^701^ | No CHR-P population |
| 1. Kommescher 2016 ^702^ | No relevant outcome |
| 1. Kommescher 2017 ^703^ | No relevant outcome |
| 1. Kong 2019 ^704^ | No relevant outcome |
| 1. Konings 2012 ^705^ | No dsm/icd/cut off scale criteria |
| 1. Konishi 2018 ^706^ | No CHR-P population |
| 1. Koponen 2008 ^707^ | No relevant outcome |
| 1. Koponen 2008 ^708^ | No relevant outcome |
| 1. Korkeila 2005 ^709^ | No CHR-P population |
| 1. Korkeila 2007 ^710^ | No relevant outcome |
| 1. Korver 2010 ^711^ | No relevant outcome |
| 1. Koshiyama 2017 ^712^ | No dsm/icd/cut off scale criteria |
| 1. Koshiyama 2018 ^713^ | No relevant outcome |
| 1. Koshiyama 2018 ^714^ | No relevant outcome |
| Koshiyama 2018^715^ | No relevant outcome |
| 1. Köther 2018 ^716^ | No relevant outcome |
| 1. Kotlicka-Antczak 2015 ^717^ | No CHR-P population |
| 1. Kotlicka-Antczak 2018 ^718^ | No relevant outcome |
| 1. Kotlicka-Antczak 2019 ^719^ | No relevant outcome |
| 1. Koutsouleris 2009 ^720^ | No relevant outcome |
| 1. Koutsouleris 2009 ^721^ | No relevant outcome |
| 1. Koutsouleris 2010 ^722^ | No relevant outcome |
| 1. Koutsouleris 2012 ^723^ | No relevant outcome |
| 1. Koutsouleris 2015 ^724^ | No relevant outcome |
| 1. Koutsouleris 2019 ^725^ | No relevant outcome |
| 1. Kraan 2015 ^726^ | Study Design |
| 1. Kraan 2017 ^727^ | No relevant outcome |
| 1. Krakauer 2017 ^728^ | No relevant outcome |
| 1. Krakauer 2018 ^729^ | No relevant outcome |
| 1. Kristensen 2007 ^730^ | No relevant outcome |
| 1. Krkovic 2017 ^731^ | No relevant outcome |
| 1. Kuharic 2020 ^732^ | No relevant outcome |
| 1. Kunwar 2012 ^733^ | Study Design |
| 1. Kwapil 1996 ^734^ | No relevant outcome |
| 1. Kwapil 2000 ^735^ | No CHR-P population |
| 1. Labad 2018 ^736^ | No relevant outcome |
| 1. Lagopoulos 2013 ^737^ | No dsm/icd/cut off scale criteria |
| 1. Laloyaux 2016 ^738^ | No dsm/icd/cut off scale criteria |
| 1. Langbein 2018 ^739^ | No CHR-P population |
| 1. Lappin 2007 ^740^ | No dsm/icd/cut off scale criteria |
| 1. Larsen 2019 ^741^ | No relevant outcome |
| 1. Larson 2012 ^742^ | No relevant outcome |
| 1. Laskaris 2019 ^743^ | Study Design |
| 1. Lataster 2009 ^744^ | No relevant outcome |
| 1. Lavoie 2012 ^745^ | No CHR-P population |
| 1. Lavoie 2014 ^746^ | No dsm/icd/cut off scale criteria |
| 1. Lavoie 2016 ^747^ | No dsm/icd/cut off scale criteria |
| 1. Lavoie 2017 ^748^ | No relevant outcome |
| 1. Lavoie 2018 ^749^ | No dsm/icd/cut off scale criteria |
| 1. Leanza 2018 ^750^ | No relevant outcome |
| 1. Lederman 2017 ^751^ | No relevant outcome |
| 1. Lee 2008 ^752^ | No dsm/icd/cut off scale criteria |
| 1. Lee 2010 ^753^ | No dsm/icd/cut off scale criteria |
| 1. Lee 2011 ^754^ | No dsm/icd/cut off scale criteria |
| 1. Lee 2012 ^755^ | No dsm/icd/cut off scale criteria |
| 1. Lee 2013 ^756^ | No dsm/icd/cut off scale criteria |
| 1. Lee 2014 ^757^ | No relevant outcome |
| 1. Lee 2014 ^758^ | No dsm/icd/cut off scale criteria |
| 1. Lee 2015 ^759^ | No dsm/icd/cut off scale criteria |
| 1. Lee 2018 ^760^ | No relevant outcome |
| 1. Lee 2020 ^761^ | No relevant outcome |
| 1. Lehembre-Shiah 2017 ^762^ | No relevant outcome |
| 1. Lemmers-Jansen 2019 ^763^ | No dsm/icd/cut off scale criteria |
| 1. Lemmers-Jansen 2019 ^764^ | No dsm/icd/cut off scale criteria |
| 1. Lemmers-Jansen 2020 ^765^ | No relevant outcome |
| 1. Lemos 2006 ^766^ | No relevant outcome |
| 1. Lemos-Giraldez 2009 ^767^ | Article not in English |
| 1. Lencz 2003 ^768^ | No relevant outcome |
| 1. Lencz 2006 ^769^ | No relevant outcome |
| 1. Lennertz 2012 ^770^ | No relevant outcome |
| 1. Lepock 2019 ^771^ | No relevant outcome |
| 1. Lewis 2020 ^772^ | No relevant outcome |
| 1. Lho 2021 ^773^ | No CHR-P population |
| 1. Li 2021 ^774^ | No relevant outcome |
| 1. Li 2018^775^ | No relevant outcome |
| 1. Li 2019 ^776^ | No relevant outcome |
| 1. Lian 2018 ^777^ | No relevant outcome |
| 1. Liemburg 2016 ^778^ | No relevant outcome |
| 1. Lin 2011 ^779^ | No relevant outcome |
| 1. Lin 2013 ^780^ | No dsm/icd/cut off scale criteria |
| 1. Lin 2013 ^781^ | No dsm/icd/cut off scale criteria |
| 1. Lin 2015 ^782^ | No relevant outcome |
| 1. Lin 2018 ^783^ | No relevant outcome |
| 1. Lindgren 2010 ^784^ | No dsm/icd/cut off scale criteria |
| 1. Lindgren 2017 ^785^ | No relevant outcome |
| 1. Lindgren 2019 ^786^ | No relevant outcome |
| 1. Lindgren 2021 ^787^ | No CHR-P population |
| 1. Liu 2010 ^788^ | No relevant outcome |
| 1. Liu 2011 ^789^ | No CHR-P population |
| 1. Liu 2013 ^790^ | No relevant outcome |
| 1. Liu 2013 ^791^ | No relevant outcome |
| 1. Liu 2015 ^792^ | No dsm/icd/cut off scale criteria |
| 1. Liu 2019 ^793^ | No relevant outcome |
| 1. Loch 2017 ^794^ | No relevant outcome |
| 1. Loch 2019 ^795^ | No CHR-P population |
| 1. Loewy 2005 ^796^ | No relevant outcome |
| 1. Loewy 2007 ^797^ | No dsm/icd/cut off scale criteria |
| 1. Loewy 2011 ^798^ | No relevant outcome |
| 1. Loewy 2012 ^799^ | No CHR-P population |
| 1. Loewy 2016 ^800^ | No dsm/icd/cut off scale criteria |
| 1. Long 2018 ^801^ | No relevant outcome |
| 1. LoPilato 2019 ^802^ | No relevant outcome |
| 1. LoPilato 2020 ^803^ | No relevant outcome |
| 1. Lord 2011 ^804^ | No relevant outcome |
| 1. Lorenzo 2018 ^805^ | No relevant outcome |
| 1. Loughland 2004 ^806^ | No relevant outcome |
| 1. Louza 2008 ^807^ | No CHR-P population |
| 1. Lu 2017 ^808^ | No relevant outcome |
| 1. Lucas-Molina 2020 ^809^ | No dsm/icd/cut off scale criteria |
| 1. Lucy Poe 2014 ^810^ | No CHR-P population |
| 1. Lunsford-Avery 2013 ^811^ | No relevant outcome |
| 1. Lunsford-Avery 2015 ^812^ | No dsm/icd/cut off scale criteria |
| 1. Lunsford-Avery 2017 ^813^ | No relevant outcome |
| 1. Lunsford-Avery 2017 ^814^ | No dsm/icd/cut off scale criteria |
| 1. Luo 2019 ^815^ | No relevant outcome |
| 1. Lyngberg 2015 ^816^ | No dsm/icd/cut off scale criteria |

1. Aase I, Kompus K, Gisselgård J, Joa I, Johannessen JO, Brønnick K. Language Lateralization and Auditory Attention Impairment in Young Adults at Ultra-High Risk for Psychosis: A Dichotic Listening Study. *Frontiers in Psychology*. 2018;9. doi:10.3389/fpsyg.2018.00608

2. Adamson V, Barrass E, McConville S, et al. Implementing the access and waiting time standard for early intervention in psychosis in the United Kingdom: An evaluation of referrals and post-assessment outcomes over the first year of operation. *Early Intervention in Psychiatry*. 2018;12(5):979-986. doi:10.1111/eip.12548

3. Addington J, Addington D. Clinical trials during the prodromal stage of schizophrenia. *Am J Psychiatry*. 2005;162(7):1387. doi:10.1176/appi.ajp.162.7.1387

4. Addington J, Penn D, Woods SW, Addington D, Perkins DO. *Facial Affect Recognition in Individuals at Clinical High Risk for Psychosis*.

5. Addington J, Penn D, Woods SW, Addington D, Perkins DO. Social functioning in individuals at clinical high risk for psychosis. *Schizophrenia Research*. 2008;99(1-3):119-124. doi:10.1016/j.schres.2007.10.001

6. Addington J, Epstein I, Reynolds A, et al. Early detection of psychosis: Finding those at clinical high risk. *Early Intervention in Psychiatry*. 2008;2(3):147-153. doi:10.1111/j.1751-7893.2008.00078.x

7. Addington J, Tran L. Using the brief core schema scales with individuals at clinical high risk of psychosis. *Behavioural and Cognitive Psychotherapy*. 2009;37(2):227-231. doi:10.1017/S1352465809005116

8. Addington D, Berzins S, Yeo M. Psychosis Literacy in a Canadian Health Region: Results from a General Population Sample. *The Canadian Journal of Psychiatry*. 2012;57(6):381-388. doi:10.1177/070674371205700608

9. Addington J, Piskulic D, Perkins D, Woods SW, Liu L, Penn DL. Affect recognition in people at clinical high risk of psychosis. *Schizophrenia Research*. 2012;140(1-3):87-92. doi:10.1016/j.schres.2012.06.012

10. Addington J, McGregor L, Marulanda D, Raedler T. Recruitment strategies for the detection of individuals at clinical high risk of developing psychosis. *Epidemiology and Psychiatric Sciences*. 2013;22(2):181-185. doi:10.1017/S2045796012000583

11. Addington J, Shah H, Liu L, Addington D. Reliability and validity of the Calgary Depression Scale for Schizophrenia (CDSS) in youth at clinical high risk for psychosis. *Schizophrenia Research*. 2014;153(1-3):64-67. doi:10.1016/j.schres.2013.12.014

12. Addington J, Liu L, Buchy L, et al. North American Prodrome Longitudinal Study (NAPLS 2). *Journal of Nervous & Mental Disease*. 2015;203(5):328-335. doi:10.1097/NMD.0000000000000290

13. Addington J, Shakeel MK, Braun A, Bonneville D, Stowkowy J. Metacognition in youth at-risk for psychosis. *Schizophrenia Research*. 2019;210:303-305. doi:10.1016/j.schres.2019.07.005

14. Addington J, Liu L, Farris MS, et al. Clinical staging for youth at-risk for serious mental illness: A longitudinal perspective. *Early Intervention in Psychiatry*. 2021;15(5):1188-1196. doi:10.1111/eip.13062

15. Addington J, Farris MS, Liu L, et al. Depression: An actionable outcome for those at clinical high-risk. *Schizophrenia Research*. 2021;227:38-43. doi:10.1016/j.schres.2020.10.001

16. Agurto C, Pietrowicz M, Norel R, et al. Analyzing acoustic and prosodic fluctuations in free speech to predict psychosis onset in high-risk youths. In: *42nd Annual International Conference of the IEEE Engineering in Medicine & Biology Society (EMBC)*. IEEE; 2020:5575-5579. doi:10.1109/EMBC44109.2020.9176841

17. Albers M, Schultze-Lutter F, Steinmeyer EM, Klosterkötter J. Can self-experienced neuropsychological deficits indicate propensity to schizophrenic psychosis? Results of an 8-year prospective follow-up study. *International Clinical Psychopharmacology*. 1998;13:S75-S80. doi:10.1097/00004850-199801001-00013

18. Alderman T, Addington J, Bearden C, et al. Negative symptoms and impaired social functioning predict later psychosis in Latino youth at clinical high risk in the North American prodromal longitudinal studies consortium. *Early Intervention in Psychiatry*. 2015;9(6):467-475. doi:10.1111/eip.12128

19. Aleksandrowicz A, Hagenmuller F, Haker H, et al. Frontal brain activity in individuals at risk for schizophrenic psychosis and bipolar disorder during the emotional Stroop task – an fNIRS study. *NeuroImage: Clinical*. 2020;26. doi:10.1016/j.nicl.2020.102232

20. Allen P, Stephan KE, Mechelli A, et al. Cingulate activity and fronto-temporal connectivity in people with prodromal signs of psychosis. *Neuroimage*. 2010;49(1):947-955. doi:10.1016/j.neuroimage.2009.08.038

21. Allen P, Seal ML, Valli I, et al. Altered Prefrontal and Hippocampal Function During Verbal Encoding and Recognition in People With Prodromal Symptoms of Psychosis. *Schizophrenia Bulletin*. 2011;37(4):746-756. doi:10.1093/schbul/sbp113

22. Allen P, Chaddock CA, Howes OD, et al. Abnormal Relationship Between Medial Temporal Lobe and Subcortical Dopamine Function in People With an Ultra High Risk for Psychosis. *Schizophrenia Bulletin*. 2012;38(5):1040-1049. doi:10.1093/schbul/sbr017

23. Allen P, Luigjes J, Howes OD, et al. Transition to Psychosis Associated With Prefrontal and Subcortical Dysfunction in Ultra High-Risk Individuals. *Schizophrenia Bulletin*. 2012;38(6):1268-1276. doi:10.1093/schbul/sbr194

24. Allen P, Chaddock CA, Egerton A, et al. Resting Hyperperfusion of the Hippocampus, Midbrain, and Basal Ganglia in People at High Risk for Psychosis. *American Journal of Psychiatry*. 2016;173(4):392-399. doi:10.1176/appi.ajp.2015.15040485

25. Allen P, Chaddock CA, Egerton A, et al. Functional Outcome in People at High Risk for Psychosis Predicted by Thalamic Glutamate Levels and Prefronto-Striatal Activation. *Schizophrenia Bulletin*. 2015;41(2):429-439. doi:10.1093/schbul/sbu115

26. Allen P, Azis M, Modinos G, et al. Increased Resting Hippocampal and Basal Ganglia Perfusion in People at Ultra High Risk for Psychosis: Replication in a Second Cohort. *Schizophrenia Bulletin*. 2018;44(6):1323-1331. doi:10.1093/schbul/sbx169

27. Allott K, Wood SJ, Yuen HP, et al. Longitudinal Cognitive Performance in Individuals at Ultrahigh Risk for Psychosis: A 10-year Follow-up. *Schizophrenia Bulletin*. 2019;45(5):1101-1111. doi:10.1093/schbul/sby143

28. Allswede DM, Addington J, Bearden CE, et al. Characterizing Covariant Trajectories of Individuals at Clinical High Risk for Psychosis Across Symptomatic and Functional Domains. *American Journal of Psychiatry*. 2020;177(2):164-171. doi:10.1176/appi.ajp.2019.18111290

29. Alqarni A, Mitchell TW, McGorry PD, et al. Supplementation with the omega-3 long chain polyunsaturated fatty acids: Changes in the concentrations of omega-3 index, fatty acids and molecular phospholipids of people at ultra high risk of developing psychosis. *Schizophrenia Research*. 2020;226:52-60. doi:10.1016/j.schres.2019.08.033

30. Alqarni A, Mitchell TW, McGorry PD, et al. Comparison of erythrocyte omega-3 index, fatty acids and molecular phospholipid species in people at ultra-high risk of developing psychosis and healthy people. *Schizophrenia Research*. 2020;226:44-51. doi:10.1016/j.schres.2019.06.020

31. Alvarez-Jimenez M, Gleeson JF, Bendall S, et al. Enhancing social functioning in young people at Ultra High Risk (UHR) for psychosis: A pilot study of a novel strengths and mindfulness-based online social therapy. *Schizophrenia Research*. 2018;202:369-377. doi:10.1016/j.schres.2018.07.022

32. Amminger GP, Leicester S, Yung AR, et al. Early-onset of symptoms predicts conversion to non-affective psychosis in ultra-high risk individuals. *Schizophrenia Research*. 2006;84(1):67-76. doi:10.1016/j.schres.2006.02.018

33. Amminger GP, McGorry PD, Berger GE, et al. Antibodies to Infectious Agents in Individuals at Ultra-High Risk for Psychosis. *Biological Psychiatry*. 2007;61(10):1215-1217. doi:10.1016/j.biopsych.2006.09.034

34. Amminger GP, Schäfer MR, Papageorgiou K, et al. Long-Chain ω-3 Fatty Acids for Indicated Prevention of Psychotic Disorders. *Archives of General Psychiatry*. 2010;67(2):146. doi:10.1001/archgenpsychiatry.2009.192

35. Amminger GP, Schafer MR, Papageorgiou K, et al. Emotion Recognition in Individuals at Clinical High-Risk for Schizophrenia. *Schizophrenia Bulletin*. 2012;38(5):1030-1039. doi:10.1093/schbul/sbr015

36. Amminger GP, Schäfer MR, Klier CM, et al. Decreased nervonic acid levels in erythrocyte membranes predict psychosis in help-seeking ultra-high-risk individuals. *Molecular Psychiatry*. 2012;17(12):1150-1152. doi:10.1038/mp.2011.167

37. Amminger GP, Schäfer MR, Klier CM, et al. Facial and vocal affect perception in people at ultra-high risk of psychosis, first-episode schizophrenia and healthy controls. *Early Intervention in Psychiatry*. 2012;6(4):450-454. doi:10.1111/j.1751-7893.2012.00362.x

38. Amminger GP, Allott K, Schlögelhofer M, et al. Affect recognition and functioning in putatively prodromal individuals. *Schizophrenia Research*. 2013;147(2-3):404-405. doi:10.1016/j.schres.2013.04.008

39. Amminger GP, Chanen AM, Ohmann S, et al. Omega-3 Fatty Acid Supplementation in Adolescents with Borderline Personality Disorder and Ultra-High Risk Criteria for Psychosis: A Post Hoc Subgroup Analysis of a Double—Blind, Randomized Controlled Trial. *The Canadian Journal of Psychiatry*. 2013;58(7):402-408. doi:10.1177/070674371305800705

40. Amminger GP, Mechelli A, Rice S, et al. Predictors of treatment response in young people at ultra-high risk for psychosis who received long-chain omega-3 fatty acids. *Translational Psychiatry*. 2015;5(1):e495-e495. doi:10.1038/tp.2014.134

41. Amminger GP, Nelson B, Markulev C, et al. The NEURAPRO Biomarker Analysis: Long-Chain Omega-3 Fatty Acids Improve 6-Month and 12-Month Outcomes in Youths at Ultra-High Risk for Psychosis. *Biological Psychiatry*. 2020;87(3):243-252. doi:10.1016/j.biopsych.2019.08.030

42. Amos AJ. Biased Reporting of Results in Patients at Ultra-High Risk of Psychosis. *The Journal of Clinical Psychiatry*. 2013;74(11). doi:10.4088/JCP.12lr08602

43. Andersen EH, Campbell AM, Schipul SE, et al. Electrophysiological Correlates of Aberrant Motivated Attention and Salience Processing in Unaffected Relatives of Schizophrenia Patients. *Clinical EEG and Neuroscience*. 2016;47(1):11-23. doi:10.1177/1550059415598063

44. Andrade C. Cannabis and Neuropsychiatry, 2: The Longitudinal Risk of Psychosis as an Adverse Outcome. *The Journal of Clinical Psychiatry*. 2016;77(06):e739-e742. doi:10.4088/JCP.16f10918

45. Andreou C, Leicht G, Nolte G, et al. Resting-state theta-band connectivity and verbal memory in schizophrenia and in the high-risk state. *Schizophrenia Research*. 2015;161(2-3):299-307. doi:10.1016/j.schres.2014.12.018

46. Anglin DM, Lighty Q, Greenspoon M, Ellman LM. Racial discrimination is associated with distressing subthreshold positive psychotic symptoms among US urban ethnic minority young adults. *Social Psychiatry and Psychiatric Epidemiology*. 2014;49(10). doi:10.1007/s00127-014-0870-8

47. Anglin DM, Greenspoon M, Lighty Q, Ellman LM. Race-based rejection sensitivity partially accounts for the relationship between racial discrimination and distressing attenuated positive psychotic symptoms. *Early Intervention in Psychiatry*. 2016;10(5):411-418. doi:10.1111/eip.12184

48. Anglin DM, Lui F, Espinosa A, Tikhonov A, Ellman L. Ethnic identity, racial discrimination and attenuated psychotic symptoms in an urban population of emerging adults. *Early Intervention in Psychiatry*. 2018;12(3):380-390. doi:10.1111/eip.12314

49. Study strengthens evidence that early marijuana use increases risk of psychosis. *Harv Ment Health Lett*. 2011;27(11).

50. Anticevic A, Tang Y, Cho YT, et al. Amygdala Connectivity Differs Among Chronic, Early Course, and Individuals at Risk for Developing Schizophrenia. *Schizophrenia Bulletin*. 2014;40(5):1105-1116. doi:10.1093/schbul/sbt165

51. Anticevic A, Haut K, Murray JD, et al. Association of Thalamic Dysconnectivity and Conversion to Psychosis in Youth and Young Adults at Elevated Clinical Risk. *JAMA Psychiatry*. 2015;72(9):882. doi:10.1001/jamapsychiatry.2015.0566

52. Appiah-Kusi E, Fisher HL, Petros N, et al. Do cognitive schema mediate the association between childhood trauma and being at ultra-high risk for psychosis? *Journal of Psychiatric Research*. 2017;88:89-96. doi:10.1016/j.jpsychires.2017.01.003

53. Appiah-Kusi E, Petros N, Wilson R, et al. Effects of short-term cannabidiol treatment on response to social stress in subjects at clinical high risk of developing psychosis. *Psychopharmacology (Berl)*. 2020;237(4):1121-1130. doi:10.1007/s00213-019-05442-6

54. Appiah-Kusi E, Wilson R, Colizzi M, et al. Childhood trauma and being at-risk for psychosis are associated with higher peripheral endocannabinoids. *Psychological Medicine*. 2020;50(11):1862-1871. doi:10.1017/S0033291719001946

55. Armando M, Girardi P, Vicari S, et al. Adolescents at ultra-high risk for psychosis with and without 22q11 deletion syndrome: A comparison of prodromal psychotic symptoms and general functioning. *Schizophrenia Research*. 2012;139(1-3):151-156. doi:10.1016/j.schres.2012.04.020

56. Armando M, Schneider M, Pontillo M, et al. No age effect in the prevalence and clinical significance of ultra-high risk symptoms and criteria for psychosis in 22q11 deletion syndrome: Confirmation of the genetically driven risk for psychosis? *PLOS ONE*. 2017;12(4):e0174797. doi:10.1371/journal.pone.0174797

57. Aston J, Rechsteiner E, Bull N, Borgwardt S, Gschwandtner U, Riecher-Rössler A. Hyperprolactinaemia in early psychosis—not only due to antipsychotics. *Progress in Neuro-Psychopharmacology and Biological Psychiatry*. 2010;34(7):1342-1344. doi:10.1016/j.pnpbp.2010.02.019

58. Atkinson RJ, Michie PT, Schall U. Duration Mismatch Negativity and P3a in First-Episode Psychosis and Individuals at Ultra-High Risk of Psychosis. *Biological Psychiatry*. 2012;71(2):98-104. doi:10.1016/j.biopsych.2011.08.023

59. Atkinson RJ, Fulham WR, Michie PT, et al. Electrophysiological, cognitive and clinical profiles of at-risk mental state: The longitudinal Minds in Transition (MinT) study. *PLOS ONE*. 2017;12(2):e0171657. doi:10.1371/journal.pone.0171657

60. Austin JC, Hippman C, Honer WG. Descriptive and numeric estimation of risk for psychotic disorders among affected individuals and relatives: Implications for clinical practice. *Psychiatry Research*. 2012;196(1):52-56. doi:10.1016/j.psychres.2012.02.005

61. Avery JRL. *Sleep Dysfunction in Adolescents at High Risk for Psychosis*. 2013.

62. Aydin K, Ucok A, Guler J. Altered Metabolic Integrity of Corpus Callosum Among Individuals at Ultra High Risk of Schizophrenia and First-Episode Patients. *Biological Psychiatry*. 2008;64(9):750-757. doi:10.1016/j.biopsych.2008.04.007

63. Aylott A, Zwicker A, MacKenzie LE, et al. Like father like daughter: sex-specific parent-of-origin effects in the transmission of liability for psychotic symptoms to offspring. *Journal of Developmental Origins of Health and Disease*. 2019;10(1):100-107. doi:10.1017/S2040174418000612

64. Azar M, Pruessner M, Baer LH, Iyer S, Malla AK, Lepage M. A study on negative and depressive symptom prevalence in individuals at ultra-high risk for psychosis. *Early Intervention in Psychiatry*. 2018;12(5):900-906. doi:10.1111/eip.12386

65. Azzali S, Pelizza L, Paterlini F, et al. Reliability of the italian version of the 16-item Prodromal Questionnaire (iPQ-16) for psychosis risk screening in a young help-seeking community sample. *Journal of Psychopathology*. 2018;24:16-23.

66. Ärmänen A, Lahti M, Therman S, Suvisaari J, Lindgren M. Psychological, social and role functioning as predictors of psychosis in an adolescent psychiatric sample. *Early Intervention in Psychiatry*. 2018;12(6):1064-1071. doi:10.1111/eip.12414

67. Baer LH, Shah JL, Lepage M. Anxiety in youth at clinical high risk for psychosis: A case study and conceptual model. *Schizophrenia Research*. 2019;208:441-446. doi:10.1016/j.schres.2019.01.006

68. Baker K, Chaddock CA, Baldeweg T, Skuse D. Neuroanatomy in adolescents and young adults with 22q11 Deletion Syndrome: Comparison to an IQ-matched group. *Neuroimage*. 2011;55(2):491-499. doi:10.1016/j.neuroimage.2010.12.041

69. Bakker G, Caan MWA, Vingerhoets WAM, et al. Cortical Morphology Differences in Subjects at Increased Vulnerability for Developing a Psychotic Disorder: A Comparison between Subjects with Ultra-High Risk and 22q11.2 Deletion Syndrome. *PLOS ONE*. 2016;11(11):e0159928. doi:10.1371/journal.pone.0159928

70. Pelletier-Baldelli A. Do youth at risk for psychosis like being liked?: A Functional Magnetic Resonance Imaging investigation of social reward processing. Published online 2019.

71. Ballon JS, Kaur T, Marks II, Cadenhead KS. Social functioning in young people at risk for schizophrenia. *Psychiatry Research*. 2007;151(1-2):29-35. doi:10.1016/j.psychres.2006.10.012

72. Bang M, Kim KR, Song YY, Baek S, Lee E, An SK. Neurocognitive impairments in individuals at ultra-high risk for psychosis: Who will really convert? *Australian & New Zealand Journal of Psychiatry*. 2015;49(5):462-470. doi:10.1177/0004867414561527

73. Bang M, Park HJ, Pae C, et al. Aberrant cerebro-cerebellar functional connectivity and minimal self-disturbance in individuals at ultra-high risk for psychosis and with first-episode schizophrenia. *Schizophrenia Research*. 2018;202:138-140. doi:10.1016/j.schres.2018.06.031

74. Bang M, Kang JI, Kim SJ, et al. Reduced DNA Methylation of the Oxytocin Receptor Gene Is Associated With Anhedonia-Asociality in Women With Recent-Onset Schizophrenia and Ultra-high Risk for Psychosis. *Schizophrenia Bulletin*. 2019;45(6):1279-1290. doi:10.1093/schbul/sbz016

75. Bang M, Park JY, Kim KR, et al. Psychotic conversion of individuals at ultra‐high risk for psychosis: The potential roles of schizotypy and basic symptoms. *Early Intervention in Psychiatry*. 2019;13(3):546-554. doi:10.1111/eip.12518

76. Bang M, Park JY, Kim KR, et al. Suicidal ideation in individuals at ultra‐high risk for psychosis and its association with suspiciousness independent of depression. *Early Intervention in Psychiatry*. 2019;13(3):539-545. doi:10.1111/eip.12517

77. Barbato M, Colijn MA, Keefe RSE, et al. The course of cognitive functioning over six months in individuals at clinical high risk for psychosis. *Psychiatry Research*. 2013;206(2-3):195-199. doi:10.1016/j.psychres.2012.10.013

78. Barbato M, Liu L, Penn DL, et al. Social cognition as a mediator between neurocognition and functional outcome in individuals at clinical high risk for psychosis. *Schizophrenia Research*. 2013;150(2-3):542-546. doi:10.1016/j.schres.2013.08.015

79. Barbato M, Penn DL, Perkins DO, Woods SW, Liu L, Addington J. Metacognitive Functioning in Individuals at Clinical High Risk for Psychosis. *Behavioural and Cognitive Psychotherapy*. 2014;42(5):526-534. doi:10.1017/S1352465813000167

80. Barbato M, Addington J. Binocular depth perception in individuals at clinical high risk for psychosis: No evidence of dysfunction. *Neuropsychology*. 2014;28(3):366-372. doi:10.1037/neu0000033

81. Barkus E, Stirling J, French P, Morrison A, Bentall R, Lewis S. Distress and Metacognition in Psychosis Prone Individuals. *Journal of Nervous & Mental Disease*. 2010;198(2):99-104. doi:10.1097/NMD.0b013e3181cc418a

82. Barkus E, Smallman R, Royle N, Barkus C, Lewis S, Rushe T. Auditory false perceptions are mediated by psychosis risk factors. *Cognitive Neuropsychiatry*. 2011;16(4):289-302. doi:10.1080/13546805.2010.530472

83. Barkus E. High-potency cannabis increases the risk of psychosis. *Evidence Based Mental Health*. 2016;19(2):54-54. doi:10.1136/eb-2015-102105

84. BARNETT JH, SAHAKIAN BJ, WERNERS U, et al. Visuospatial learning and executive function are independently impaired in first-episode psychosis. *Psychological Medicine*. 2005;35(7):1031-1041. doi:10.1017/S0033291704004301

85. Baron M, Gruen RS, Romo-Gruen JM. Positive and Negative Symptoms. *British Journal of Psychiatry*. 1992;161(5):610-614. doi:10.1192/bjp.161.5.610

86. Barrantes-Vidal N, Gross GM, Sheinbaum T, Mitjavila M, Ballespí S, Kwapil TR. Positive and negative schizotypy are associated with prodromal and schizophrenia-spectrum symptoms. *Schizophrenia Research*. 2013;145(1-3):50-55. doi:10.1016/j.schres.2013.01.007

87. Bartholomeusz CF, Whittle SL, Pilioussis E, et al. Relationship between amygdala volume and emotion recognition in adolescents at ultra-high risk for psychosis. *Psychiatry Research: Neuroimaging*. 2014;224(3). doi:10.1016/j.pscychresns.2014.10.005

88. Bartók E, Berecz R, Glaub T, Degrell I. Cognitive functions in prepsychotic patients. *Progress in Neuro-Psychopharmacology and Biological Psychiatry*. 2005;29(4):621-625. doi:10.1016/j.pnpbp.2005.01.008

89. Bearden CE, Wu KN, Caplan R, Cannon TD. Thought Disorder and Communication Deviance as Predictors of Outcome in Youth at Clinical High Risk for Psychosis. *Journal of the American Academy of Child & Adolescent Psychiatry*. 2011;50(7):669-680. doi:10.1016/j.jaac.2011.03.021

90. Bechdolf A, Veith V, Pukrop R, Klosterkötter J. Health Related Quality of Life in Subjects at Risk for a First Episode Of Psychosis. In: *Quality of Life Impairment in Schizophrenia, Mood and Anxiety Disorders*. Springer Netherlands. doi:10.1007/978-1-4020-5779-3_9

91. Bechdolf A, Ruhrmann S, Wagner M, et al. Interventions in the initial prodromal states of psychosis in Germany: concept and recruitment. *British Journal of Psychiatry*. 2005;187(S48). doi:10.1192/bjp.187.48.s45

92. Bechdolf A, Pukrop R, Köhn D, et al. Subjective quality of life in subjects at risk for a first episode of psychosis: A comparison with first episode schizophrenia patients and healthy controls. *Schizophrenia Research*. 2005;79(1):137-143. doi:10.1016/j.schres.2005.06.008

93. Bechdolf A, Wagner M, Veith V, et al. Randomized controlled multicentre trial of cognitive behaviour therapy in the early initial prodromal state: effects on social adjustment post treatment. *Early Interv Psychiatry*. 2007;1(1):71-78. doi:10.1111/j.1751-7893.2007.00013.x

94. Bechdolf A, Wagner M, Ruhrmann S, et al. Preventing progression to first-episode psychosis in early initial prodromal states. *British Journal of Psychiatry*. 2012;200(1):22-29. doi:10.1192/bjp.bp.109.066357

95. Becker HE, Nieman DH, Dingemans PM, van de Fliert JR, de Haan L, Linszen DH. Verbal fluency as a possible predictor for psychosis. *European Psychiatry*. 2010;25(2):105-110. doi:10.1016/j.eurpsy.2009.08.003

96. Becker HE, Nieman DH, Wiltink S, et al. Neurocognitive functioning before and after the first psychotic episode: does psychosis result in cognitive deterioration? *Psychological Medicine*. 2010;40(10):1599-1606. doi:10.1017/S0033291710000048

97. Bedwell JS, Donnelly RS. Schizotypal personality disorder or prodromal symptoms of schizophrenia? *Schizophrenia Research*. 2005;80(2-3):263-269. doi:10.1016/j.schres.2005.07.023

98. Benavides C, Brucato G, Kimhy D. Self-esteem and Symptoms in Individuals at Clinical High Risk for Psychosis. *Journal of Nervous & Mental Disease*. 2018;206(6):433-438. doi:10.1097/NMD.0000000000000824

99. Ben-David S, Birnbaum ML, Eilenberg ME, et al. The Subjective Experience of Youths at Clinically High Risk of Psychosis: A Qualitative Study. *Psychiatric Services*. 2014;65(12). doi:10.1176/appi.ps.201300527

100. Ben-David S, Cole AR, Brucato G, Girgis R, Munson MR. A conceptual model of mental health service utilization among young adults at clinical high-risk for developing psychosis. *Psychiatric Rehabilitation Journal*. 2019;42(1):17-25. doi:10.1037/prj0000336

101. Ben‐David S, Cole A, Brucato G, Girgis RR, Munson MR. Mental health service use decision‐making among young adults at clinical high risk for developing psychosis. *Early Intervention in Psychiatry*. 2019;13(5):1050-1055. doi:10.1111/eip.12725

102. Bendfeldt K, Smieskova R, Koutsouleris N, et al. Classifying individuals at high-risk for psychosis based on functional brain activity during working memory processing. *NeuroImage: Clinical*. 2015;9:555-563. doi:10.1016/j.nicl.2015.09.015

103. Benetti S, Mechelli A, Picchioni M, Broome M, Williams S, McGuire P. Functional integration between the posterior hippocampus and prefrontal cortex is impaired in both first episode schizophrenia and the at risk mental state. *Brain*. 2009;132(9):2426-2436. doi:10.1093/brain/awp098

104. Benetti S, Pettersson-Yeo W, Hutton C, et al. Elucidating neuroanatomical alterations in the at risk mental state and first episode psychosis: A combined voxel-based morphometry and voxel-based cortical thickness study. *Schizophrenia Research*. 2013;150(2-3):505-511. doi:10.1016/j.schres.2013.08.030

105. Benetti S, Pettersson-Yeo W, Allen P, et al. Auditory Verbal Hallucinations and Brain Dysconnectivity in the Perisylvian Language Network: A Multimodal Investigation. *Schizophrenia Bulletin*. 2015;41(1):192-200. doi:10.1093/schbul/sbt172

106. Berger G, Wood SJ, Dell’olio M, et al. Neuroprotective effects of low dose lithiumin individuals at ultra-high risk for pshychosis. A longitudinal MRI/MRS study. *Schizophrenia Research*. 2008;102(1-3):39-40. doi:10.1016/s0920-9964(08)70125-7

107. Berger GE, Smesny S, Schäfer MR, et al. Niacin Skin Sensitivity Is Increased in Adolescents at Ultra-High Risk for Psychosis. *PLOS ONE*. 2016;11(2):e0148429. doi:10.1371/journal.pone.0148429

108. Berger GE, Bartholomeusz CF, Wood SJ, et al. Ventricular volumes across stages of schizophrenia and other psychoses. *Australian & New Zealand Journal of Psychiatry*. 2017;51(10):1041-1051. doi:10.1177/0004867417715914

109. Berger M, Lavoie S, McGorry PD, et al. Relationship between allostatic load and clinical outcomes in youth at ultra-high risk for psychosis in the NEURAPRO study. *Schizophrenia Research*. 2020;226:38-43. doi:10.1016/j.schres.2018.10.002

110. Berman BA. *Pseudochromesthesia and Psychosis Proneness.* 2011.

111. Berna F, Göritz AS, Schröder J, et al. Self-disorders in individuals with attenuated psychotic symptoms: Contribution of a dysfunction of autobiographical memory. *Psychiatry Research*. 2016;239:333-341. doi:10.1016/j.psychres.2016.03.029

112. Bernard JA, Dean DJ, Kent JS, et al. Cerebellar networks in individuals at ultra high‐risk of psychosis: Impact on postural sway and symptom severity. *Human Brain Mapping*. 2014;35(8):4064-4078. doi:10.1002/hbm.22458

113. Bernard JA, Orr JM, Dean DJ, Mittal VA. The cerebellum and learning of non-motor associations in individuals at clinical-high risk for psychosis. *NeuroImage: Clinical*. 2018;19:137-146. doi:10.1016/j.nicl.2018.03.023

114. Bernasconi R, Smieskova R, Schmidt A, et al. Hippocampal volume correlates with attenuated negative psychotic symptoms irrespective of antidepressant medication. *NeuroImage: Clinical*. 2015;8:230-237. doi:10.1016/j.nicl.2015.04.016

115. Berrocal AC. *Preventing Psychosis in High Risk Adolescents: Early Detection and Intervention Reduces Symptoms AndImproves Functioning at First Hope, a California Treatment Program*. 2020.

116. Bertisch HC, Fava J, Kattan A, Delisi LE. Preliminary neuropsychological findings in individuals at high genetic risk for schizophrenia. *Early Intervention in Psychiatry*. 2008;2(1):45-49. doi:10.1111/j.1751-7893.2007.00058.x

117. Bhattacharyya S, Wilson R, Appiah-Kusi E, et al. Effect of Cannabidiol on Medial Temporal, Midbrain, and Striatal Dysfunction in People at Clinical High Risk of Psychosis. *JAMA Psychiatry*. 2018;75(11):1107. doi:10.1001/jamapsychiatry.2018.2309

118. Bhojraj TS, Francis AN, Rajarethinam R, et al. Verbal fluency deficits and altered lateralization of language brain areas in individuals genetically predisposed to schizophrenia. *Schizophrenia Research*. 2009;115(2-3):202-208. doi:10.1016/j.schres.2009.09.033

119. Birchwood M, Smith J, Macmillan F, et al. Predicting relapse in schizophrenia: the development and implementation of an early signs monitoring system using patients and families as observers, a preliminary investigation. *Psychological Medicine*. 1989;19(3):649-656. doi:10.1017/S0033291700024247

120. Bjornestad J, Tjora T, Langeveld JH, et al. Exploring specific predictors of psychosis onset over a 2-year period: A decision-tree model. *Early Intervention in Psychiatry*. Published online 2021. doi:10.1111/eip.13175

121. Blanchard MM, Jacobson S, Clarke MC, et al. Language, motor and speed of processing deficits in adolescents with subclinical psychotic symptoms. *Schizophrenia Research*. 2010;123(1):71-76. doi:10.1016/j.schres.2010.05.028

122. Blasco D, Stortz SW, Grivel MMR, et al. Naturalistic conceptions of genetic optimism and precision psychiatry among those at clinical high-risk for psychosis. *Early Intervention in Psychiatry*. 2021;15(3):742-745. doi:10.1111/eip.12983

123. Blessing A, Studer A, Gross A, Gruss LF, Schneider R, Dammann G. Disclosure of Diagnosis in Early Recognition of Psychosis. *Journal of Nervous & Mental Disease*. 2017;205(10):757-761. doi:10.1097/NMD.0000000000000729

124. Bloemen OJN, de Koning MB, Schmitz N, et al. White-matter markers for psychosis in a prospective ultra-high-risk cohort. *Psychological Medicine*. 2010;40(8):1297-1304. doi:10.1017/S0033291709991711

125. Bloemen OJN, de Koning MB, Gleich T, et al. Striatal dopamine D2/3 receptor binding following dopamine depletion in subjects at Ultra High Risk for psychosis. *European Neuropsychopharmacology*. 2013;23(2):126-132. doi:10.1016/j.euroneuro.2012.04.015

126. Blomstrom A, Karlsson H, Svensson A, et al. Hospital Admission With Infection During Childhood and Risk for Psychotic Illness--A Population-based Cohort Study. *Schizophrenia Bulletin*. 2014;40(6):1518-1525. doi:10.1093/schbul/sbt195

127. Bohner G, Milakara D, Witthaus H, et al. MTR abnormalities in subjects at ultra-high risk for schizophrenia and first-episode schizophrenic patients compared to healthy controls. *Schizophrenia Research*. 2012;137(1-3):85-90. doi:10.1016/j.schres.2012.01.020

128. Boldrini T, lo Buglio G, Giovanardi G, Lingiardi V, Salcuni S. Defense mechanisms in adolescents at high risk of developing psychosis: An empirical investigation. *Research in Psychotherapy: Psychopathology, Process and Outcome*. 2020;23(1). doi:10.4081/ripppo.2020.456

129. Bolt LK, Amminger GP, Farhall J, et al. Neurocognition as a predictor of transition to psychotic disorder and functional outcomes in ultra-high risk participants: Findings from the NEURAPRO randomized clinical trial. *Schizophrenia Research*. 2019;206:67-74. doi:10.1016/j.schres.2018.12.013

130. Borgwardt SJ, Radue EW, Götz K, et al. Radiological findings in individuals at high risk of psychosis. *Journal of Neurology, Neurosurgery and Psychiatry*. 2006;77(2):229-233. doi:10.1136/jnnp.2005.069690

131. Borgwardt SJ, McGuire PK, Aston J, et al. Structural brain abnormalities in individuals with an at-risk mental state who later develop psychosis. *British Journal of Psychiatry*. 2007;191(S51). doi:10.1192/bjp.191.51.s69

132. Borgwardt SJ, Riecher-Rössler A, Dazzan P, et al. Regional Gray Matter Volume Abnormalities in the At Risk Mental State. *Biological Psychiatry*. 2007;61(10):1148-1156. doi:10.1016/j.biopsych.2006.08.009

133. Borgwardt SJ, McGuire PK, Aston J, et al. Reductions in frontal, temporal and parietal volume associated with the onset of psychosis. *Schizophrenia Research*. 2008;106(2-3):108-114. doi:10.1016/j.schres.2008.08.007

134. Borgwardt SJ, McGuire P, Fusar-Poli P, Radue EW, Riecher-Rössler A. Anterior cingulate pathology in the prodromal stage of schizophrenia. *Neuroimage*. 2008;39(2). doi:10.1016/j.neuroimage.2007.08.047

135. Borgwardt S, Koutsouleris N, Aston J, et al. Distinguishing Prodromal From First-Episode Psychosis Using Neuroanatomical Single-Subject Pattern Recognition. *Schizophrenia Bulletin*. 2013;39(5):1105-1114. doi:10.1093/schbul/sbs095

136. Bossong MG, Antoniades M, Azis M, et al. Association of Hippocampal Glutamate Levels With Adverse Outcomes in Individuals at Clinical High Risk for Psychosis. *JAMA Psychiatry*. 2019;76(2):199. doi:10.1001/jamapsychiatry.2018.3252

137. Bourgin J, Duchesnay E, Magaud E, Gaillard R, Kazes M, Krebs MO. Predicting the individual risk of psychosis conversion in at-risk mental state (ARMS): a multivariate model reveals the influence of nonpsychotic prodromal symptoms. *European Child & Adolescent Psychiatry*. 2020;29(11):1525-1535. doi:10.1007/s00787-019-01461-y

138. Bousman CA, Yung AR, Pantelis C, et al. Effects of NRG1 and DAOA genetic variation on transition to psychosis in individuals at ultra-high risk for psychosis. *Translational Psychiatry*. 2013;3. doi:10.1038/tp.2013.23

139. Braham A, Bannour AS, ben Romdhane A, et al. Validation of the Arabic version of the Comprehensive Assessment of At Risk Mental States (CAARMS) in Tunisian adolescents and young adults. *Early Intervention in Psychiatry*. 2014;8(2):147-154. doi:10.1111/eip.12031

140. Bramon E, Shaikh M, Broome M, et al. Abnormal P300 in people with high risk of developing psychosis. *Neuroimage*. 2008;41(2):553-560. doi:10.1016/j.neuroimage.2007.12.038

141. Brandizzi M, Schultze-Lutter F, Masillo A, et al. Self-reported attenuated psychotic-like experiences in help-seeking adolescents and their association with age, functioning and psychopathology. *Schizophrenia Research*. 2014;160(1-3):110-117. doi:10.1016/j.schres.2014.10.005

142. Brett CMC, Peters ER, McGuire PK. Which psychotic experiences are associated with a need for clinical care? *European Psychiatry*. 2015;30(5):648-654. doi:10.1016/j.eurpsy.2014.12.005

143. Brewer WJ, Wood SJ, McGorry PD, et al. Impairment of Olfactory Identification Ability in Individuals at Ultra-High Risk for Psychosis Who Later Develop Schizophrenia. *American Journal of Psychiatry*. 2003;160(10):1790-1794. doi:10.1176/appi.ajp.160.10.1790

144. Brewer WJ, Lin A, Moberg PJ, et al. Phenylthiocarbamide (PTC) perception in ultra-high risk for psychosis participants who develop schizophrenia: Testing the evidence for an endophenotypic marker. *Psychiatry Research*. 2012;199(1):8-11. doi:10.1016/j.psychres.2012.03.010

145. Brockhaus-Dumke A, Tendolkar I, Pukrop R, Schultze-Lutter F, Klosterkötter J, Ruhrmann S. Impaired mismatch negativity generation in prodromal subjects and patients with schizophrenia. *Schizophrenia Research*. 2005;73(2-3):297-310. doi:10.1016/j.schres.2004.05.016

146. Brockhaus-Dumke A, Schultze-Lutter F, Mueller R, et al. Sensory Gating in Schizophrenia: P50 and N100 Gating in Antipsychotic-Free Subjects at Risk, First-Episode, and Chronic Patients. *Biological Psychiatry*. 2008;64(5):376-384. doi:10.1016/j.biopsych.2008.02.006

147. Brodey BB, Addington J, First MB, et al. The Early Psychosis Screener (EPS): Item development and qualitative validation. *Schizophrenia Research*. 2018;197:504-508. doi:10.1016/j.schres.2017.11.027

148. Brodey BB, Girgis RR, Favorov OV, et al. The Early Psychosis Screener (EPS): Quantitative validation against the SIPS using machine learning. *Schizophrenia Research*. 2018;197:516-521. doi:10.1016/j.schres.2017.11.030

149. Brodey BB, Girgis RR, Favorov OV, et al. The Early Psychosis Screener for Internet (EPSI)-SR: Predicting 12 month psychotic conversion using machine learning. *Schizophrenia Research*. 2019;208:390-396. doi:10.1016/j.schres.2019.01.015

150. Broome MR, Johns LC, Valli I, et al. Delusion formation and reasoning biases in those at clinical high risk for psychosis. *British Journal of Psychiatry*. 2007;191(S51). doi:10.1192/bjp.191.51.s38

151. Broome MR, Matthiasson P, Fusar-Poli P, et al. Neural correlates of executive function and working memory in the “at-risk mental state.” *British Journal of Psychiatry*. 2009;194(1):25-33. doi:10.1192/bjp.bp.107.046789

152. Broome MR, Matthiasson P, Fusar-Poli P, et al. Neural correlates of movement generation in the “at-risk mental state.” *Acta Psychiatrica Scandinavica*. 2010;122(4):295-301. doi:10.1111/j.1600-0447.2009.01524.x

153. Broome MR, Fusar-Poli P, Matthiasson P, et al. Neural correlates of visuospatial working memory in the ‘at-risk mental state.’ *Psychological Medicine*. 2010;40(12):1987-1999. doi:10.1017/S0033291710000280

154. Broome MR, Day F, Valli I, et al. Delusional ideation, manic symptomatology and working memory in a cohort at clinical high-risk for psychosis: A longitudinal study. *European Psychiatry*. 2012;27(4):258-263. doi:10.1016/j.eurpsy.2010.07.008

155. Brucato G, Appelbaum PS, Lieberman JA, et al. A Longitudinal Study of Violent Behavior in a Psychosis-Risk Cohort. *Neuropsychopharmacology*. 2018;43(2):264-271. doi:10.1038/npp.2017.151

156. Brummitt K, Addington J. Treatment possibilities for individuals at clinical high risk of psychosis. *Early Intervention in Psychiatry*. 2013;7(2):155-161. doi:10.1111/j.1751-7893.2012.00370.x

157. Brüne M, Özgürdal S, Ansorge N, et al. An fMRI study of “theory of mind” in at-risk states of psychosis: Comparison with manifest schizophrenia and healthy controls. *Neuroimage*. 2011;55(1):329-337. doi:10.1016/j.neuroimage.2010.12.018

158. Brüne M, Drommelschmidt K, Krüger‐Özgürdal S, Juckel G. Relationship between metacognitive beliefs and psychosocial performance in at‐risk states of psychosis and patients with first psychotic episodes. *Early Intervention in Psychiatry*. 2019;13(3):604-612. doi:10.1111/eip.12536

159. Bucci S, Baker A, Halpin SA, et al. Intervention for cannabis use in young people at ultra high risk for psychosis and in early psychosis. *Mental Health and Substance Use*. 2010;3(1):66-73. doi:10.1080/17523280903523983

160. Buchy L, Seidman LJ, Cadenhead KS, et al. Evaluating the relationship between cannabis use and IQ in youth and young adults at clinical high risk of psychosis. *Psychiatry Research*. 2015;230(3):878-884. doi:10.1016/j.psychres.2015.11.033

161. Buchy L, Cannon TD, Anticevic A, et al. Evaluating the impact of cannabis use on thalamic connectivity in youth at clinical high risk of psychosis. *BMC Psychiatry*. 2015;15(1):276. doi:10.1186/s12888-015-0656-x

162. Buchy L, Stowkowy J, MacMaster FP, Nyman K, Addington J. Meta-cognition is associated with cortical thickness in youth at clinical high risk of psychosis. *Psychiatry Research: Neuroimaging*. 2015;233(3):418-423. doi:10.1016/j.pscychresns.2015.07.010

163. Buchy L, Mathalon DH, Cannon TD, et al. Relation between cannabis use and subcortical volumes in people at clinical high risk of psychosis. *Psychiatry Research: Neuroimaging*. 2016;254:3-9. doi:10.1016/j.pscychresns.2016.06.001

164. Buehlmann E, Berger GE, Aston J, et al. Hippocampus abnormalities in at risk mental states for psychosis? A cross-sectional high resolution region of interest magnetic resonance imaging study. *Journal of Psychiatric Research*. 2010;44(7):447-453. doi:10.1016/j.jpsychires.2009.10.008

165. Bukenaite A, Stochl J, Mossaheb N, et al. Usefulness of the CAPE-P15 for detecting people at ultra-high risk for psychosis: Psychometric properties and cut-off values. *Schizophrenia Research*. 2017;189:69-74. doi:10.1016/j.schres.2017.02.017

166. Burley K, Upthegrove R, Birchwood M, Patterson P, Skeate A. “Schizophrenia postdrome”: A study of low-level psychotic experience after remission of first-episode schizophrenia. *Early Intervention in Psychiatry*. 2009;3(4):296-299. doi:10.1111/j.1751-7893.2009.00141.x

167. Burton CZ, Tso IF, Carrión RE, et al. Baseline psychopathology and relationship to longitudinal functional outcome in attenuated and early first episode psychosis. *Schizophrenia Research*. 2019;212:157-162. doi:10.1016/j.schres.2019.07.048

168. Büschlen J, Berger GE, Borgwardt SJ, et al. Pituitary volume increase during emerging psychosis. *Schizophrenia Research*. 2011;125(1):41-48. doi:10.1016/j.schres.2010.09.022

169. Byars SG, Stearns SC, Boomsma JJ. Opposite risk patterns for autism and schizophrenia are associated with normal variation in birth size: phenotypic support for hypothesized diametric gene-dosage effects. *Proceedings of the Royal Society B: Biological Sciences*. 2014;281(1794):20140604. doi:10.1098/rspb.2014.0604

170. Bykowsky O, Harrisberger F, Schmidt A, et al. Association of antidepressants with brain morphology in early stages of psychosis: an imaging genomics approach. *Scientific Reports*. 2019;9(1):8516. doi:10.1038/s41598-019-44903-y

171. Byrne R, Morrison AP. Young people at risk of psychosis: a user-led exploration of interpersonal relationships and communication of psychological difficulties. *Early Intervention in Psychiatry*. 2010;4(2):162-168. doi:10.1111/j.1751-7893.2010.00171.x

172. Byrne RE, Morrison AP. Young people at risk of psychosis: Their subjective experiences of monitoring and cognitive behaviour therapy in the early detection and intervention evaluation 2 trial. *Psychology and Psychotherapy: Theory, Research and Practice*. 2014;87(3):357-371. doi:10.1111/papt.12013

173. Byrne M, Codjoe L, Morgan C, et al. The relationship between ethnicity and service access, treatment uptake and the incidence of psychosis among people at ultra high risk for psychosis. *Psychiatry Research*. 2019;272:618-627. doi:10.1016/j.psychres.2018.12.111

174. Byun MS, Kim JS, Jung WH, et al. Regional cortical thinning in subjects with high genetic loading for schizophrenia. *Schizophrenia Research*. 2012;141(2-3):197-203. doi:10.1016/j.schres.2012.08.028

175. Cadenhead KS, Light GA, Shafer KM, Braff DL. P50 Suppression in Individuals at Risk for Schizophrenia: The Convergence of Clinical, Familial, and Vulnerability Marker Risk Assessment. *Biological Psychiatry*. 2005;57(12):1504-1509. doi:10.1016/j.biopsych.2005.03.003

176. Cadenhead KS, Addington J, Cannon T, et al. Treatment history in the psychosis prodrome: characteristics of the North American Prodrome Longitudinal Study Cohort. *Early Intervention in Psychiatry*. 2010;4(3):220-226. doi:10.1111/j.1751-7893.2010.00183.x

177. Cadenhead KS. Startle reactivity and prepulse inhibition in prodromal and early psychosis: Effects of age, antipsychotics, tobacco and cannabis in a vulnerable population. *Psychiatry Research*. 2011;188(2):208-216. doi:10.1016/j.psychres.2011.04.011

178. Cadenhead KS, Minichino A, Kelsven S, et al. Metabolic abnormalities and low dietary Omega 3 are associated with symptom severity and worse functioning prior to the onset of psychosis: Findings from the North American Prodrome Longitudinal Studies Consortium. *Schizophrenia Research*. 2019;204:96-103. doi:10.1016/j.schres.2018.09.022

179. Caldani S, Amado I, Bendjemaa N, et al. Oculomotricity and Neurological Soft Signs: Can we refine the endophenotype? A study in subjects belonging to the spectrum of schizophrenia. *Psychiatry Research*. 2017;256:490-497. doi:10.1016/j.psychres.2017.06.013

180. Caldani S, Bucci MP, Lamy JC, et al. Saccadic eye movements as markers of schizophrenia spectrum: Exploration in at-risk mental states. *Schizophrenia Research*. 2017;181:30-37. doi:10.1016/j.schres.2016.09.003

181. Calkins ME, Woods SW, Bearden CE, et al. Concordance and factor structure of subthreshold positive symptoms in youth at clinical high risk for psychosis. *Schizophrenia Research*. 2021;227:72-77. doi:10.1016/j.schres.2020.08.014

182. Callaway DA, Perkins DO, Woods SW, Liu L, Addington J. Movement abnormalities predict transitioning to psychosis in individuals at clinical high risk for psychosis. *Schizophrenia Research*. 2014;159(2-3). doi:10.1016/j.schres.2014.09.031

183. Campanella S, Colin C. Event-related potentials and biomarkers of psychiatric diseases: the necessity to adopt and develop multi-site guidelines. *Frontiers in Behavioral Neuroscience*. 2014;8. doi:10.3389/fnbeh.2014.00428

184. Campion J, Taylor MJ, McDaid D, Park A, Shiers D. Applying economic models to estimate local economic benefits of improved coverage of early intervention for psychosis. *Early Intervention in Psychiatry*. 2019;13(6):1424-1430. doi:10.1111/eip.12787

185. Can G, Bora E, Ildız A, et al. Neurocognition in young offspring of individuals with bipolar disorder: The role of co-existing familial and clinical high-risk for bipolar disorder. *Psychiatry Research*. 2019;281:112565. doi:10.1016/j.psychres.2019.112565

186. Cannon TD, Huttunen MO, Dahlström M, Larmo I, Räsänen P, Juriloo A. Antipsychotic Drug Treatment in the Prodromal Phase of Schizophrenia. *American Journal of Psychiatry*. 2002;159(7):1230-1232. doi:10.1176/appi.ajp.159.7.1230

187. Cannon TD, Cadenhead K, Cornblatt B, et al. Prediction of psychosis in youth at high clinical risk: A multisite longitudinal study in North America. *Archives of General Psychiatry*. 2008;65(1):28-37. doi:10.1001/archgenpsychiatry.2007.3

188. Cannon TD, Chung Y, He G, et al. Progressive Reduction in Cortical Thickness as Psychosis Develops: A Multisite Longitudinal Neuroimaging Study of Youth at Elevated Clinical Risk. *Biological Psychiatry*. 2015;77(2):147-157. doi:10.1016/j.biopsych.2014.05.023

189. Cannon TD, Yu C, Addington J, et al. An Individualized Risk Calculator for Research in Prodromal Psychosis. *American Journal of Psychiatry*. 2016;173(10):980-988. doi:10.1176/appi.ajp.2016.15070890

190. Cao H, Chén OY, Chung Y, et al. Cerebello-thalamo-cortical hyperconnectivity as a state-independent functional neural signature for psychosis prediction and characterization. *Nature Communications*. 2018;9(1):3836. doi:10.1038/s41467-018-06350-7

191. Cao H, McEwen SC, Chung Y, et al. Altered Brain Activation During Memory Retrieval Precedes and Predicts Conversion to Psychosis in Individuals at Clinical High Risk. *Schizophrenia Bulletin*. 2019;45(4):924-933. doi:10.1093/schbul/sby122

192. Caravaggio F, Brucato G, Kegeles LS, et al. Exploring the Relationship Between Body Mass Index and Positive Symptom Severity in Persons at Clinical High Risk for Psychosis. *Journal of Nervous & Mental Disease*. 2017;205(11):893-895. doi:10.1097/NMD.0000000000000736

193. Carberry AT. Interpersonal decentering, social interaction, and treatment status in children at risk for psychosis. *Dissertation Abstracts International*. 1982;43(5-B):1608.

194. Carey CJ. *Using Online Recruitment to Examine the Role of Social Cognition in Psychosocial Functioning in Individuals At-Risk for Psychosis.* 2020.

195. Carletti F, Woolley JB, Bhattacharyya S, et al. Alterations in White Matter Evident Before the Onset of Psychosis. *Schizophrenia Bulletin*. 2012;38(6):1170-1179. doi:10.1093/schbul/sbs053

196. Carney R, Bradshaw T, Firth J, Cotter J, Yung AR. Lifestyle factors may be linked to symptoms of metabolic syndrome in people at risk for psychosis. *Schizophrenia Research*. 2017;183:47-48. doi:10.1016/j.schres.2016.11.013

197. Carney R, Cotter J, Bradshaw T, Yung AR. Examining the physical health and lifestyle of young people at ultra-high risk for psychosis: A qualitative study involving service users, parents and clinicians. *Psychiatry Research*. 2017;255:87-93. doi:10.1016/j.psychres.2017.05.023

198. Carney R, Yung AR, Amminger GP, et al. Substance use in youth at risk for psychosis. *Schizophrenia Research*. 2017;181:23-29. doi:10.1016/j.schres.2016.08.026

199. Carney R, Bradshaw T, Yung AR. Monitoring of physical health in services for young people at ultra-high risk of psychosis. *Early Intervention in Psychiatry*. 2018;12(2):153-159. doi:10.1111/eip.12288

200. Carol EE, Mittal VA. Self-reported cannabis use is inconsistent with the results from drug-screening in youth at ultra high-risk for psychosis in Colorado. *Schizophrenia Research*. 2014;157(1-3). doi:10.1016/j.schres.2014.05.032

201. Carol EE. *Longitudinal Evaluation of Stress Systems in Youth at Risk for Developing Psychosis.* PhD Dissertation Thesis. Faculty of the Graduate School of the University of Colorado; 2018.

202. Carpenter WT. Clinical High Risk Controversies and Challenge for the Experts. *Schizophrenia Bulletin*. 2018;44(2):223-225. doi:10.1093/schbul/sbx182

203. Carrión RE, Goldberg TE, McLaughlin D, Auther AM, Correll CU, Cornblatt BA. Impact of Neurocognition on Social and Role Functioning in Individuals at Clinical High Risk for Psychosis. *American Journal of Psychiatry*. 2011;168(8):806-813. doi:10.1176/appi.ajp.2011.10081209

204. Carrión RE, Cornblatt BA, McLaughlin D, et al. Contributions of early cortical processing and reading ability to functional status in individuals at clinical high risk for psychosis. *Schizophrenia Research*. 2015;164(1-3):1-7. doi:10.1016/j.schres.2015.01.030

205. Carrión RE, Cornblatt BA, Burton CZ, et al. Personalized Prediction of Psychosis: External Validation of the NAPLS-2 Psychosis Risk Calculator With the EDIPPP Project. *American Journal of Psychiatry*. 2016;173(10):989-996. doi:10.1176/appi.ajp.2016.15121565

206. Carrión RE, Auther AM, McLaughlin D, et al. The Global Functioning: Social and Role Scales—Further Validation in a Large Sample of Adolescents and Young Adults at Clinical High Risk for Psychosis. *Schizophrenia Bulletin*. 2019;45(4):763-772. doi:10.1093/schbul/sby126

207. Carroll CA, O’Donnell BF, Shekhar A, Hetrick WP. The effects of olanzapine on sensory gating in healthy participants. *Schizophrenia Research*. 2004;66(2-3):187-189. doi:10.1016/S0920-9964(03)00149-X

208. Castle DJ. Is it appropriate to treat people at high‐risk of psychosis before first onset? *Medical Journal of Australia*. 2012;196(9):557-557. doi:10.5694/mja12.10669

209. Castro J, Zanini M, Gonçalves B da SB, et al. Circadian rest–activity rhythm in individuals at risk for psychosis and bipolar disorder. *Schizophrenia Research*. 2015;168(1-2):50-55. doi:10.1016/j.schres.2015.07.024

210. Catalan A, Tognin S, Kempton MJ, et al. Relationship between jumping to conclusions and clinical outcomes in people at clinical high-risk for psychosis. *Psychological Medicine*. Published online October 6, 2020:1-9. doi:10.1017/S0033291720003396

211. Chan RCK. Adopting a cognitive neuroscience approach to study clinically ultra-high-risk individuals. *PsyCh Journal*. 2017;6(1):100-101. doi:10.1002/pchj.163

212. Chan RCK, Cui H ru, Chu M yi, et al. Neurological soft signs precede the onset of schizophrenia: a study of individuals with schizotypy, ultra-high-risk individuals, and first-onset schizophrenia. *European Archives of Psychiatry and Clinical Neuroscience*. 2018;268(1):49-56. doi:10.1007/s00406-017-0828-4

213. Chan CT, Abdin E, Subramaniam M, Tay SA, Lim LK, Verma S. Two-Year Clinical and Functional Outcomes of an Asian Cohort at Ultra-High Risk of Psychosis. *Frontiers in Psychiatry*. 2019;9. doi:10.3389/fpsyt.2018.00758

214. Chan KN, Chang WC, Ng CM, et al. Sex differences in symptom severity, cognition and psychosocial functioning among individuals with at-risk mental state for psychosis. *Early Intervention in Psychiatry*. Published online 2021. doi:10.1111/eip.13131

215. Chang WC, Lee HC, Chan SI, et al. Negative symptom dimensions differentially impact on functioning in individuals at-risk for psychosis. *Schizophrenia Research*. 2018;202:310-315. doi:10.1016/j.schres.2018.06.041

216. Chang Q, Liu M, Tian Q, et al. EEG-Based Brain Functional Connectivity in First-Episode Schizophrenia Patients, Ultra-High-Risk Individuals, and Healthy Controls During P50 Suppression. *Frontiers in Human Neuroscience*. 2019;13. doi:10.3389/fnhum.2019.00379

217. Chapman LJ, Chapman JP. Scales for Rating Psychotic and Psychotic-like Experiences as Continua. *Schizophrenia Bulletin*. 1980;6(3). doi:10.1093/schbul/6.3.476

218. Chapman LJ, Edell WS, Chapman JP. Physical Anhedonia, Perceptual Aberration, and Psychosis Proneness. *Schizophrenia Bulletin*. 1980;6(4):639-653. doi:10.1093/schbul/6.4.639

219. Chapman JP, Chapman LJ, Kwapil TR. Does the Eysenck psychoticism scale predict psychosis? A ten year longitudinal study. *Personality and Individual Differences*. 1994;17(3):369-375. doi:10.1016/0191-8869(94)90284-4

220. Chapman HC, Visser KF, Mittal VA, Gibb BE, Coles ME, Strauss GP. Emotion regulation across the psychosis continuum. *Development and Psychopathology*. 2020;32(1):219-227. doi:10.1017/S0954579418001682

221. Chaumette B, Kebir O, Mam-Lam-Fook C, et al. Salivary cortisol in early psychosis: New findings and meta-analysis. *Psychoneuroendocrinology*. 2016;63:262-270. doi:10.1016/j.psyneuen.2015.10.007

222. Chaumette B, Kebir O, Pouch J, et al. Longitudinal Analyses of Blood Transcriptome During Conversion to Psychosis. *Schizophrenia Bulletin*. 2019;45(1):247-255. doi:10.1093/schbul/sby009

223. Chaumette B, Sengupta SM, Lepage M, et al. A polymorphism in the glutamate metabotropic receptor 7 is associated with cognitive deficits in the early phases of psychosis. *Schizophrenia Research*. Published online 2020. doi:10.1016/j.schres.2020.06.019

224. Chen F, Wang L, Heeramun-Aubeeluck A, et al. Identification and characterization of college students with Attenuated Psychosis Syndrome in China. *Psychiatry Research*. 2014;216(3). doi:10.1016/j.psychres.2014.01.051

225. Chen FZ, Wang Y, Sun XR, et al. Emotional Experiences Predict the Conversion of Individuals with Attenuated Psychosis Syndrome to Psychosis: A 6-Month Follow up Study. *Frontiers in Psychology*. 2016;7. doi:10.3389/fpsyg.2016.00818

226. Chen F, Wang L, Wang J, Heeramun-Aubeeluck A, Yuan J, Zhao X. Applicability of the Chinese version of the 16-item Prodromal Questionnaire (CPQ-16) for identifying attenuated psychosis syndrome in a college population. *Early Intervention in Psychiatry*. 2016;10(4):308-315. doi:10.1111/eip.12173

227. Chen X, Li X, Yan T, et al. Network functional connectivity analysis in individuals at ultrahigh risk for psychosis and patients with schizophrenia. *Psychiatry Research: Neuroimaging*. 2019;290:51-57. doi:10.1016/j.pscychresns.2019.06.004

228. Chén OY, Cao H, Phan H, et al. Identifying neural signatures mediating behavioral symptoms and psychosis onset: High-dimensional whole brain functional mediation analysis. *Neuroimage*. 2021;226. doi:10.1016/j.neuroimage.2020.117508

229. Cho KIK, Shenton ME, Kubicki M, et al. Altered Thalamo-Cortical White Matter Connectivity: Probabilistic Tractography Study in Clinical-High Risk for Psychosis and First-Episode Psychosis. *Schizophrenia Bulletin*. 2016;42(3):723-731. doi:10.1093/schbul/sbv169

230. Choi JS, Kang DH, Park JY, et al. Cavum septum pellucidum in subjects at ultra-high risk for psychosis: Compared with first-degree relatives of patients with schizophrenia and healthy volunteers. *Progress in Neuro-Psychopharmacology and Biological Psychiatry*. 2008;32(5):1326-1330. doi:10.1016/j.pnpbp.2008.04.011

231. Choi JS, Park JY, Jung MH, et al. Phase-Specific Brain Change of Spatial Working Memory Processing in Genetic and Ultra-High Risk Groups of Schizophrenia. *Schizophrenia Bulletin*. 2012;38(6):1189-1199. doi:10.1093/schbul/sbr038

232. Choi SH, Kyeong S, Cho KIK, et al. Brain network characteristics separating individuals at clinical high risk for psychosis into normality or psychosis. *Schizophrenia Research*. 2017;190:107-114. doi:10.1016/j.schres.2017.03.028

233. Choi J, Corcoran CM, Fiszdon JM, et al. Pupillometer-based neurofeedback cognitive training to improve processing speed and social functioning in individuals at clinical high risk for psychosis. *Psychiatric Rehabilitation Journal*. 2017;40(1):33-42. doi:10.1037/prj0000217

234. Chon MW, Lee TY, Kim SN, et al. Factors contributing to the duration of untreated prodromal positive symptoms in individuals at ultra-high risk for psychosis. *Schizophrenia Research*. 2015;162(1-3):64-66. doi:10.1016/j.schres.2015.01.013

235. Chu AOK, Chang WC, Chan SKW, Lee EHM, Hui CLM, Chen EYH. Comparison of cognitive functions between first-episode schizophrenia patients, their unaffected siblings and individuals at clinical high-risk for psychosis. *Psychological Medicine*. 2019;49(11):1929-1936. doi:10.1017/S0033291718002726

236. Chudleigh C, Naismith SL, Blaszczynski A, Hermens DF, Hodge MAR, Hickie IB. How does social functioning in the early stages of psychosis relate to depression and social anxiety? *Early Intervention in Psychiatry*. 2011;5(3):224-232. doi:10.1111/j.1751-7893.2011.00280.x

237. Yu Sun Chung, Kang DH, Na Young Shin, So Young Yoo, Jun Soo Kwon. Deficit of theory of mind in individuals at ultra-high-risk for schizophrenia. *Schizophrenia Research*. 2008;99(1-3):111-118. doi:10.1016/j.schres.2007.11.012

238. Chung YC, Kang NI, Im YJ, et al. Validation of the Korean version of the Eppendorf Schizophrenia Inventory as a screening measure to detect adolescents at ultra-high risk for psychosis. *Early Intervention in Psychiatry*. 2013;7(1):71-79. doi:10.1111/j.1751-7893.2012.00363.x

239. Chung Y, Addington J, Bearden CE, et al. Use of Machine Learning to Determine Deviance in Neuroanatomical Maturity Associated With Future Psychosis in Youths at Clinically High Risk. *JAMA Psychiatry*. 2018;75(9):960. doi:10.1001/jamapsychiatry.2018.1543

240. Chung Y, Allswede D, Addington J, et al. Cortical abnormalities in youth at clinical high-risk for psychosis: Findings from the NAPLS2 cohort. *NeuroImage: Clinical*. 2019;23:101862. doi:10.1016/j.nicl.2019.101862

241. Ciarleglio AJ, Brucato G, Masucci MD, et al. A predictive model for conversion to psychosis in clinical high-risk patients. *Psychological Medicine*. 2019;49(07):1128-1137. doi:10.1017/S003329171800171X

242. Cicero DC, Martin EA, Becker TM, Docherty AR, Kerns JG. Correspondence between psychometric and clinical high risk for psychosis in an undergraduate population. *Psychol Assess*. 2014;26(3):901-915. doi:10.1037/a0036432

243. Clamor A, Hartmann MM, Köther U, Otte C, Moritz S, Lincoln TM. Altered autonomic arousal in psychosis: An analysis of vulnerability and specificity. *Schizophrenia Research*. 2014;154(1-3):73-78. doi:10.1016/j.schres.2014.02.006

244. Clark C, Klonoff H, Tyhurst JS, Li D, Martin W, Pate BD. Regional Cerebral Glucose Metabolism in Three Sets of Identical Twins with Psychotic Symptoms. *The Canadian Journal of Psychiatry*. 1989;34(4):263-270. doi:10.1177/070674378903400401

245. Clark SR, Baune BT, Schubert KO, et al. Prediction of transition from ultra-high risk to first-episode psychosis using a probabilistic model combining history, clinical assessment and fatty-acid biomarkers. *Translational Psychiatry*. 2016;6(9):e897-e897. doi:10.1038/tp.2016.170

246. Clark S v., Mittal VA, Bernard JA, Ahmadi A, King TZ, Turner JA. Stronger default mode network connectivity is associated with poorer clinical insight in youth at ultra high-risk for psychotic disorders. *Schizophrenia Research*. 2018;193:244-250. doi:10.1016/j.schres.2017.06.043

247. Clay KB, Raugh IM, Bartolomeo LA, Strauss GP. Defeatist performance beliefs in individuals at clinical high-risk for psychosis and outpatients with chronic schizophrenia. *Early Intervention in Psychiatry*. 2021;15(4):865-873. doi:10.1111/eip.13024

248. Clayson PE, Kern RS, Nuechterlein KH, et al. Social vs. non-social measures of learning potential for predicting community functioning across phase of illness in schizophrenia. *Schizophrenia Research*. 2019;204:104-110. doi:10.1016/j.schres.2018.07.046

249. Cocchi A, Meneghelli A, Erlicher A, Pisano A, Cascio MT, Preti A. Patterns of referral in first-episode schizophrenia and ultra high-risk individuals: results from an early intervention program in Italy. *Social Psychiatry and Psychiatric Epidemiology*. 2013;48(12):1905-1916. doi:10.1007/s00127-013-0736-5

250. Cocchi A, Balbi A, Corlito G, et al. Early intervention in psychosis: a feasibility study financed by the Italian Center on Control of Maladies. *Early Intervention in Psychiatry*. 2015;9(2):163-171. doi:10.1111/eip.12135

251. Codjoe L, Byrne M, Lister M, McGuire P, Valmaggia L. Exploring Perceptions of “Wellness” in Black Ethnic Minority Individuals at Risk of Developing Psychosis. *Behavioural and Cognitive Psychotherapy*. 2013;41(2):144-161. doi:10.1017/S1352465812000707

252. Cohen CI, Mani A, Ghezelaiagh B. A Longitudinal Study of Illness Awareness in Older Adults With Schizophrenia. *The American Journal of Geriatric Psychiatry*. 2019;27(2):200-209. doi:10.1016/j.jagp.2018.10.007

253. Colibazzi T, Horga G, Wang Z, et al. Neural Dysfunction in Cognitive Control Circuits in Persons at Clinical High-Risk for Psychosis. *Neuropsychopharmacology*. 2016;41(5):1241-1250. doi:10.1038/npp.2015.273

254. Collin G, Nieto-Castanon A, Shenton ME, et al. Brain functional connectivity data enhance prediction of clinical outcome in youth at risk for psychosis. *NeuroImage: Clinical*. 2020;26. doi:10.1016/j.nicl.2019.102108

255. Collin G, Seidman LJ, Keshavan MS, et al. Functional connectome organization predicts conversion to psychosis in clinical high-risk youth from the SHARP program. *Molecular Psychiatry*. 2020;25(10):2431-2440. doi:10.1038/s41380-018-0288-x

256. Collip D, Habets P, Marcelis M, et al. Hippocampal volume as marker of daily life stress sensitivity in psychosis. *Psychological Medicine*. 2013;43(7):1377-1387. doi:10.1017/S003329171200219X

257. Comparelli A, de Carolis A, Emili E, et al. Basic symptoms and psychotic symptoms: Their relationships in the at risk mental states, first episode and multi-episode schizophrenia. *Comprehensive Psychiatry*. 2014;55(4):785-791. doi:10.1016/j.comppsych.2014.01.006

258. Comparelli A, Savoja V, Kotzalidis GD, et al. Factor–structure of the Italian version of the Scale Of Prodromal Symptoms (SOPS): a comparison with the English version. *Epidemiology and Psychiatric Sciences*. 2011;20(1):45-54. doi:10.1017/S2045796011000114

259. Comparelli A, Corigliano V, de Carolis A, et al. Emotion recognition impairment is present early and is stable throughout the course of schizophrenia. *Schizophrenia Research*. 2013;143(1):65-69. doi:10.1016/j.schres.2012.11.005

260. Comparelli A, Pucci D, Savoja V, et al. Mental disorders diagnosed in childhood and at-risk mental state in a help-seeking population. *Early Intervention in Psychiatry*. 2013;7(2):187-192. doi:10.1111/j.1751-7893.2012.00376.x

261. Comparelli A, Savoja V, de Carolis A, et al. Relationships Between Psychopathological Variables and Insight in Psychosis Risk Syndrome and First-Episode and Multiepisode Schizophrenia. *Journal of Nervous & Mental Disease*. 2013;201(3):229-233. doi:10.1097/NMD.0b013e3182834315

262. Cooper D, Barker V, Radua J, Fusar-Poli P, Lawrie SM. Multimodal voxel-based meta-analysis of structural and functional magnetic resonance imaging studies in those at elevated genetic risk of developing schizophrenia. *Psychiatry Research: Neuroimaging*. 2014;221(1). doi:10.1016/j.pscychresns.2013.07.008

263. Cooper S, Kring AM, Ellman LM. Attenuated positive psychotic symptoms and the experience of anhedonia. *Early Intervention in Psychiatry*. 2018;12(6):1188-1192. doi:10.1111/eip.12439

264. Cooper J, Jarrett M, Forrester A, et al. Substance use and at-risk mental state for psychosis in 2102 prisoners: the case for early detection and early intervention in prison. *Early Intervention in Psychiatry*. 2018;12(3):400-409. doi:10.1111/eip.12343

265. Copolov D. Neurobiological findings in early phase schizophrenia. *Brain Research Reviews*. 2000;31(2-3):157-165. doi:10.1016/S0165-0173(99)00033-8

266. Corcoran CM, Smith C, McLaughlin D, Auther A, Malaspina D, Cornblatt B. HPA axis function and symptoms in adolescents at clinical high risk for schizophrenia. *Schizophrenia Research*. 2012;135(1-3):170-174. doi:10.1016/j.schres.2011.11.035

267. Corcoran CM, Keilp JG, Kayser J, et al. Emotion recognition deficits as predictors of transition in individuals at clinical high risk for schizophrenia: a neurodevelopmental perspective. *Psychological Medicine*. 2015;45(14):2959-2973. doi:10.1017/S0033291715000902

268. Cordes J, Bechdolf A, Engelke C, et al. Prevalence of metabolic syndrome in female and male patients at risk of psychosis. *Schizophrenia Research*. 2017;181:38-42. doi:10.1016/j.schres.2016.09.012

269. Corigliano V, de Carolis A, Trovini G, et al. Neurocognition in schizophrenia: From prodrome to multi-episode illness. *Psychiatry Research*. 2014;220(1-2):129-134. doi:10.1016/j.psychres.2014.07.067

270. Cornblatt BA, Lencz T, Smith CW, Correu CU, Auther AM, Nakayama E. *The Schizophrenia Prodrome Revisited: A Neurodevelopmental Perspective*. https://academic.oup.com/schizophreniabulletin/article/29/4/633/1887776

271. Cornblatt BA, Auther AM, Niendam T, et al. Preliminary findings for two new measures of social and role functioning in the prodromal phase of schizophrenia. *Schizophrenia Bulletin*. 2007;33(3):688-702. doi:10.1093/schbul/sbm029

272. Correll CU, Lencz T, Smith CW, et al. Prospective Study of Adolescents with Subsyndromal Psychosis: Characteristics and Outcome. *Journal of Child and Adolescent Psychopharmacology*. 2005;15(3):418-433. doi:10.1089/cap.2005.15.418

273. Correll CU, Penzner JB, Frederickson AM, et al. Differentiation in the preonset phases of schizophrenia and mood disorders: Evidence in support of a bipolar mania prodrome. *Schizophrenia Bulletin*. 2007;33(3):703-714. doi:10.1093/schbul/sbm028

274. Correll CU, Smith CW, Auther AM, et al. Predictors of Remission, Schizophrenia, and Bipolar Disorder in Adolescents with Brief Psychotic Disorder or Psychotic Disorder Not Otherwise Specified Considered At Very High Risk for Schizophrenia. *Journal of Child and Adolescent Psychopharmacology*. 2008;18(5):475-490. doi:10.1089/cap.2007.110

275. Corsi-Zuelli F, Loureiro CM, Shuhama R, et al. Cytokine profile in first-episode psychosis, unaffected siblings and community-based controls: the effects of familial liability and childhood maltreatment. *Psychological Medicine*. 2020;50(7):1139-1147. doi:10.1017/S0033291719001016

276. Costello A. Mental health diagnoses during the year prior to schizophrenia, U.S. Armed Forces, 2001-2010. *MSMR*. 2012;19(3):10-13.

277. Cotter J, Bartholomeusz C, Papas A, et al. Examining the association between social cognition and functioning in individuals at ultra-high risk for psychosis. *Australian & New Zealand Journal of Psychiatry*. 2017;51(1):83-92. doi:10.1177/0004867415622691

278. Cotter J, Bucci S, Drake RJ, Yung AR, Carney R, Edge D. Exploring functional impairment in young people at ultra-high risk for psychosis: A qualitative study. *Early Intervention in Psychiatry*. 2019;13(4):789-797. doi:10.1111/eip.12560

279. Counotte J, Pot-Kolder R, van Roon AM, Hoskam O, van der Gaag M, Veling W. High psychosis liability is associated with altered autonomic balance during exposure to Virtual Reality social stressors. *Schizophrenia Research*. 2017;184:14-20. doi:10.1016/j.schres.2016.11.025

280. Counotte J, Drexhage HA, Wijkhuijs JM, et al. Th17/T regulator cell balance and NK cell numbers in relation to psychosis liability and social stress reactivity. *Brain, Behavior, and Immunity*. 2018;69:408-417. doi:10.1016/j.bbi.2017.12.015

281. Counotte J, Bergink V, Pot-Kolder R, Drexhage HA, Hoek HW, Veling W. Inflammatory cytokines and growth factors were not associated with psychosis liability or childhood trauma. *PLOS ONE*. 2019;14(7):e0219139. doi:10.1371/journal.pone.0219139

282. Couture SM, Penn DL, Addington J, Woods SW, Perkins DO. Assessment of social judgments and complex mental states in the early phases of psychosis. *Schizophrenia Research*. 2008;100(1-3):237-241. doi:10.1016/j.schres.2007.12.484

283. Cowan HR, Mittal VA. Three types of psychotic-like experiences in youth at clinical high risk for psychosis. *European Archives of Psychiatry and Clinical Neuroscience*. 2021;271(4):733-744. doi:10.1007/s00406-020-01143-w

284. Cropley VL, Lin A, Nelson B, et al. Baseline grey matter volume of non-transitioned “ultra high risk” for psychosis individuals with and without attenuated psychotic symptoms at long-term follow-up. *Schizophrenia Research*. 2016;173(3):152-158. doi:10.1016/j.schres.2015.05.014

285. Crossley NA, Mechelli A, Fusar-Poli P, et al. Superior temporal lobe dysfunction and frontotemporal dysconnectivity in subjects at risk of psychosis and in first-episode psychosis. *Human Brain Mapping*. 2009;30(12):4129-4137. doi:10.1002/hbm.20834

286. Crump FM, Arndt L, Grivel M, et al. Attenuated first-rank symptoms and conversion to psychosis in a clinical high-risk cohort. *Early Intervention in Psychiatry*. 2018;12(6):1213-1216. doi:10.1111/eip.12529

287. Cui H, Giuliano AJ, Zhang T, et al. Cognitive dysfunction in a psychotropic medication-naïve, clinical high-risk sample from the ShangHai-At-Risk-for-Psychosis (SHARP) study: Associations with clinical outcomes. *Schizophrenia Research*. 2020;226:138-146. doi:10.1016/j.schres.2020.06.018

288. Cullen AE, Dickson H, West SA, et al. Neurocognitive performance in children aged 9–12years who present putative antecedents of schizophrenia. *Schizophrenia Research*. 2010;121(1-3):15-23. doi:10.1016/j.schres.2010.05.034

289. Cullen AE, de Brito SA, Gregory SL, et al. Temporal Lobe Volume Abnormalities Precede the Prodrome: A Study of Children Presenting Antecedents of Schizophrenia. *Schizophrenia Bulletin*. 2013;39(6):1318-1327. doi:10.1093/schbul/sbs128

290. Cullen AE, Zunszain PA, Dickson H, et al. Cortisol awakening response and diurnal cortisol among children at elevated risk for schizophrenia: Relationship to psychosocial stress and cognition. *Psychoneuroendocrinology*. 2014;46. doi:10.1016/j.psyneuen.2014.03.010

291. Cullen AE, Addington J, Bearden CE, et al. Stressor-Cortisol Concordance Among Individuals at Clinical High-Risk for Psychosis: Novel Findings from the NAPLS Cohort. *Psychoneuroendocrinology*. 2020;115:104649. doi:10.1016/j.psyneuen.2020.104649

292. da Silva T, Wu A, Laksono I, et al. Mitochondrial function in individuals at clinical high risk for psychosis. *Scientific Reports*. 2018;8(1):6216. doi:10.1038/s41598-018-24355-6

293. da Silva T, Hafizi S, Andreazza AC, et al. Glutathione, the Major Redox Regulator, in the Prefrontal Cortex of Individuals at Clinical High Risk for Psychosis. *International Journal of Neuropsychopharmacology*. 2018;21(4):311-318. doi:10.1093/ijnp/pyx094

294. da Silva T, Hafizi S, Rusjan PM, et al. GABA levels and TSPO expression in people at clinical high risk for psychosis and healthy volunteers: a PET-MRS study. *Journal of Psychiatry and Neuroscience*. 2019;44(2):111-119. doi:10.1503/jpn.170201

295. Dal Mas C, Nani J v., Noto C, et al. Ndel1 oligopeptidase activity as a potential biomarker of early stages of schizophrenia. *Schizophrenia Research*. 2019;208:202-208. doi:10.1016/j.schres.2019.02.021

296. Damme KSF, Gupta T, Nusslock R, Bernard JA, Orr JM, Mittal VA. Cortical Morphometry in the Psychosis Risk Period: A Comprehensive Perspective of Surface Features. *Biological Psychiatry: Cognitive Neuroscience and Neuroimaging*. 2019;4(5):434-443. doi:10.1016/j.bpsc.2018.01.003

297. Damme KSF, Pelletier‐Baldelli A, Cowan HR, Orr JM, Mittal VA. Distinct and opposite profiles of connectivity during self‐reference task and rest in youth at clinical high risk for psychosis. *Human Brain Mapping*. 2019;40(11):3254-3264. doi:10.1002/hbm.24595

298. Damme KSF, Gallagher N, Vargas T, Osborne KJ, Gupta T, Mittal VA. Motor sequence learning and pattern recognition in youth at clinical high-risk for psychosis. *Schizophrenia Research*. 2019;208:454-456. doi:10.1016/j.schres.2019.03.023

299. Damme KSF, Osborne KJ, Gold JM, Mittal VA. Detecting motor slowing in clinical high risk for psychosis in a computerized finger tapping model. *European Archives of Psychiatry and Clinical Neuroscience*. 2020;270(3):393-397. doi:10.1007/s00406-019-01059-0

300. Daneault J ‐G., Maraj A, Lepage M, et al. Medication adherence in first episode psychosis: the role of pre‐onset subthreshold symptoms. *Acta Psychiatrica Scandinavica*. 2019;139(4):336-347. doi:10.1111/acps.13011

301. D’Angelo EJ, Lincoln SH, Morelli N, Graber K, Tembulkar S, Gonzalez-Heydrich J. Suicidal behaviors and their relationship with psychotic-like symptoms in children and adolescents at clinical high risk for psychosis. *Comprehensive Psychiatry*. 2017;78:31-37. doi:10.1016/j.comppsych.2017.07.008

302. D’Angelo EJ, Morelli N, Lincoln SH, et al. Social impairment and social language deficits in children and adolescents with and at risk for psychosis. *Schizophrenia Research*. 2019;204:304-310. doi:10.1016/j.schres.2018.07.028

303. Dannevang AL, Randers L, Gondan M, Krakauer K, Nordholm D, Nordentoft M. Premorbid adjustment in individuals at ultra-high risk for developing psychosis: a case-control study. *Early Intervention in Psychiatry*. 2018;12(5):839-847. doi:10.1111/eip.12375

304. Darrell-Berry H, Bucci S, Palmier-Claus J, Emsley R, Drake R, Berry K. Predictors and mediators of trait anger across the psychosis continuum: The role of attachment style, paranoia and social cognition. *Psychiatry Research*. 2017;249:132-138. doi:10.1016/j.psychres.2017.01.007

305. Das T, Borgwardt S, Hauke DJ, et al. Disorganized Gyrification Network Properties During the Transition to Psychosis. *JAMA Psychiatry*. 2018;75(6):613. doi:10.1001/jamapsychiatry.2018.0391

306. Das-Munshi J, Bécares L, Boydell JE, et al. Ethnic density as a buffer for psychotic experiences: findings from a national survey (EMPIRIC). *British Journal of Psychiatry*. 2012;201(4):282-290. doi:10.1192/bjp.bp.111.102376

307. Davidsen KA. Anomalous Self-Experience in Adolescents at Risk of Psychosis. *Psychopathology*. 2009;42(6). doi:10.1159/000236907

308. Davidsen KA. Anomalous Self-Experience in Adolescents at Risk of Psychosis. Clinical and conceptual elucidation. *Psychopathology*. 2009;42(6):361-369. doi:10.1159/000236907

309. Davidsen KA, Rosenbaum B. Fear of annihilation in subjects at risk of psychosis: A pilot study. *Psychosis*. 2012;4(2):149-160. doi:10.1080/17522439.2011.588339

310. Davidson CA, Piskulic D, Addington J, et al. Age-related trajectories of social cognition in youth at clinical high risk for psychosis: An exploratory study. *Schizophrenia Research*. 2018;201:130-136. doi:10.1016/j.schres.2018.05.001

311. Davies C, Rutigliano G, de Micheli A, et al. Neurochemical effects of oxytocin in people at clinical high risk for psychosis. *European Neuropsychopharmacology*. 2019;29(5):601-615. doi:10.1016/j.euroneuro.2019.03.008

312. Davies C, Paloyelis Y, Rutigliano G, et al. Oxytocin modulates hippocampal perfusion in people at clinical high risk for psychosis. *Neuropsychopharmacology*. 2019;44(7):1300-1309. doi:10.1038/s41386-018-0311-6

313. Day FL, Valmaggia LR, Mondelli V, et al. Blunted Cortisol Awakening Response in People at Ultra High Risk of Developing Psychosis. *Schizophrenia Research*. 2014;158(1-3):25-31. doi:10.1016/j.schres.2014.06.041

314. Dazzan P, Soulsby B, Mechelli A, et al. Volumetric Abnormalities Predating the Onset of Schizophrenia and Affective Psychoses: An MRI Study in Subjects at Ultrahigh Risk of Psychosis. *Schizophrenia Bulletin*. 2012;38(5):1083-1091. doi:10.1093/schbul/sbr035

315. de Koning MB, Bloemen OJ, van Duin E da, et al. Pre-pulse inhibition and striatal dopamine in subjects at an ultra-high risk for psychosis. *Journal of Psychopharmacology*. 2014;28(6):553-560. doi:10.1177/0269881113519507

316. de la Fuente-Sandoval C, León-Ortiz P, Favila R, et al. Higher Levels of Glutamate in the Associative-Striatum of Subjects with Prodromal Symptoms of Schizophrenia and Patients with First-Episode Psychosis. *Neuropsychopharmacology*. 2011;36(9):1781-1791. doi:10.1038/npp.2011.65

317. de la Fuente-Sandoval C, León-Ortiz P, Azcárraga M, Favila R, Stephano S, Graff-Guerrero A. Striatal glutamate and the conversion to psychosis: a prospective 1H-MRS imaging study. *International Journal of Neuropsychopharmacology*. 2013;16(2):471-475. doi:10.1017/S1461145712000314

318. de la Fuente-Sandoval C, Reyes-Madrigal F, Mao X, et al. Cortico-Striatal GABAergic and Glutamatergic Dysregulations in Subjects at Ultra-High Risk for Psychosis Investigated with Proton Magnetic Resonance Spectroscopy. *International Journal of Neuropsychopharmacology*. 2016;19(3):pyv105. doi:10.1093/ijnp/pyv105

319. de la Serna E, Baeza I, Toro J, et al. Relationship between clinical and neuropsychological characteristics in child and adolescent first degree relatives of subjects with schizophrenia. *Schizophrenia Research*. 2010;116(2-3):159-167. doi:10.1016/j.schres.2009.09.001

320. de la Serna E, Baeza I, Andrés S, et al. Comparison between young siblings and offspring of subjects with schizophrenia: Clinical and neuropsychological characteristics. *Schizophrenia Research*. 2011;131(1-3):35-42. doi:10.1016/j.schres.2011.06.015

321. de Wit S, Ziermans TB, Nieuwenhuis M, et al. Individual prediction of long-term outcome in adolescents at ultra-high risk for psychosis: Applying machine learning techniques to brain imaging data. *Human Brain Mapping*. 2017;38(2):704-714. doi:10.1002/hbm.23410

322. Dean DJ, Teulings HL, Caligiuri M, Mittal VA. Handwriting analysis indicates spontaneous dyskinesias in neuroleptic naïve adolescents at high risk for psychosis. *J Vis Exp*. 2013;(81). doi:10.3791/50852

323. Dean DJ, Mittal VA. Tinnitus: A potential confound when assessing perceptual abnormalities in ultra-high risk youth. *Schizophrenia Research*. 2013;147(2-3):410-411. doi:10.1016/j.schres.2013.04.033

324. Dean DJ, Kent JS, Bernard JA, et al. Increased postural sway predicts negative symptom progression in youth at ultrahigh risk for psychosis. *Schizophrenia Research*. 2015;162(1-3):86-89. doi:10.1016/j.schres.2014.12.039

325. Dean DJ, Orr JM, Newberry RE, Mittal VA. Motor behavior reflects reduced hemispheric asymmetry in the psychosis risk period. *Schizophrenia Research*. 2016;170(1):137-142. doi:10.1016/j.schres.2015.10.017

326. Dean DJ, Bryan AD, Newberry R, Gupta T, Carol E, Mittal VA. A Supervised Exercise Intervention for Youth at Risk for Psychosis. *The Journal of Clinical Psychiatry*. 2017;78(9):e1167-e1173. doi:10.4088/JCP.16m11365

327. Dean DJ, Walther S, Bernard JA, Mittal VA. Motor Clusters Reveal Differences in Risk for Psychosis, Cognitive Functioning, and Thalamocortical Connectivity: Evidence for Vulnerability Subtypes. *Clinical Psychological Science*. 2018;6(5):721-734. doi:10.1177/2167702618773759

328. Dean DJ, Samson AT, Newberry R, Mittal VA. Motion energy analysis reveals altered body movement in youth at risk for psychosis. *Schizophrenia Research*. 2018;200:35-41. doi:10.1016/j.schres.2017.05.035

329. Debbané M, Linden M, Glaser B, Eliez S. Monitoring of self-generated speech in adolescents with 22q11.2 deletion syndrome. *British Journal of Clinical Psychology*. 2010;49(3):373-386. doi:10.1348/014466509X468223

330. Debbané M, Lazouret M, Lagioia A, Schneider M, van de Ville D, Eliez S. Resting-state networks in adolescents with 22q11.2 deletion syndrome: Associations with prodromal symptoms and executive functions. *Schizophrenia Research*. 2012;139(1-3):33-39. doi:10.1016/j.schres.2012.05.021

331. Deighton S, Addington J. Exercise practices in individuals at clinical high risk of developing psychosis. *Early Intervention in Psychiatry*. 2015;9(4):284-291. doi:10.1111/eip.12107

332. Deighton S, Buchy L, Cadenhead KS, et al. Traumatic brain injury in individuals at clinical high risk for psychosis. *Schizophrenia Research*. 2016;174(1-3):77-81. doi:10.1016/j.schres.2016.04.041

333. del Re EC, Bergen SE, Mesholam-Gately RI, et al. Analysis of schizophrenia-related genes and electrophysiological measures reveals ZNF804A association with amplitude of P300b elicited by novel sounds. *Translational Psychiatry*. 2014;4. doi:10.1038/tp.2013.117

334. del Re EC, Spencer KM, Oribe N, et al. Clinical high risk and first episode schizophrenia: Auditory event-related potentials. *Psychiatry Research: Neuroimaging*. 2015;231(2):126-133. doi:10.1016/j.pscychresns.2014.11.012

335. Delaney S, Fallon B, Alaedini A, et al. Inflammatory biomarkers in psychosis and clinical high risk populations. *Schizophrenia Research*. 2019;206:440-443. doi:10.1016/j.schres.2018.10.017

336. Delevoye-Turrell Y, Wilquin H, Giersch A. A ticking clock for the production of sequential actions: Where does the problem lie in schizophrenia? *Schizophrenia Research*. 2012;135(1-3):51-54. doi:10.1016/j.schres.2011.12.020

337. Demars F, Kebir O, Marzo A, et al. Dysregulation of peripheral expression of the YWHA genes during conversion to psychosis. *Scientific Reports*. 2020;10(1):9863. doi:10.1038/s41598-020-66901-1

338. Demjaha A, Valmaggia L, Stahl D, Byrne M, McGuire P. Disorganization/Cognitive and Negative Symptom Dimensions in the At-Risk Mental State Predict Subsequent Transition to Psychosis. *Schizophrenia Bulletin*. 2012;38(2):351-359. doi:10.1093/schbul/sbq088

339. Demjaha A, Weinstein S, Stahl D, et al. Formal thought disorder in people at ultra-high risk of psychosis. *BJPsych Open*. 2017;3(4):165-170. doi:10.1192/bjpo.bp.116.004408

340. Demro C, Rowland L, Wijtenburg SA, et al. Glutamatergic metabolites among adolescents at risk for psychosis. *Psychiatry Research*. 2017;257:179-185. doi:10.1016/j.psychres.2017.07.040

341. Devoe DJ, Lu L, Cannon TD, et al. Persistent negative symptoms in youth at clinical high risk for psychosis: A longitudinal study. *Schizophrenia Research*. 2021;227:28-37. doi:10.1016/j.schres.2020.04.004

342. DeVylder JE, Lukens EP. Family history of schizophrenia as a risk factor for axis I psychiatric conditions. *Journal of Psychiatric Research*. 2013;47(2):181-187. doi:10.1016/j.jpsychires.2012.09.023

343. DeVylder JE, Oh AJ, Ben-David S, Azimov N, Harkavy-Friedman JM, Corcoran CM. Obsessive compulsive symptoms in individuals at clinical risk for psychosis: Association with depressive symptoms and suicidal ideation. *Schizophrenia Research*. 2012;140(1-3):110-113. doi:10.1016/j.schres.2012.07.009

344. DeVylder JE, Ben-David S, Kimhy D, Corcoran CM. Attributional style among youth at clinical risk for psychosis. *Early Intervention in Psychiatry*. 2013;7(1):84-88. doi:10.1111/j.1751-7893.2012.00347.x

345. DeVylder JE, Ben-David S, Schobel SA, Kimhy D, Malaspina D, Corcoran CM. Temporal association of stress sensitivity and symptoms in individuals at clinical high risk for psychosis. *Psychological Medicine*. 2013;43(2):259-268. doi:10.1017/S0033291712001262

346. DeVylder JE, Yang LH, Harkavy-Friedman JM, Azimov N, Walder DJ, Corcoran CM. Assessing depression in youth at clinical high risk for psychosis: A comparison of three measures. *Psychiatry Research*. 2014;215(2):323-328. doi:10.1016/j.psychres.2013.12.002

347. DeVylder J. Maximizing Benefits and Minimizing Risks in the Primary Prevention of Schizophrenia. *Social Work (Stellenbosch)*. 2014;59(4). doi:10.1093/sw/swu027

348. DeVylder JE, Muchomba FM, Gill KE, et al. Symptom trajectories and psychosis onset in a clinical high-risk cohort: The relevance of subthreshold thought disorder. *Schizophrenia Research*. 2014;159(2-3). doi:10.1016/j.schres.2014.08.008

349. di Biase MA, Zalesky A, O’keefe G, et al. PET imaging of putative microglial activation in individuals at ultra-high risk for psychosis, recently diagnosed and chronically ill with schizophrenia. *Translational Psychiatry*. 2017;7(8):e1225-e1225. doi:10.1038/tp.2017.193

350. Dickson Hannah, Calkins Monica E., Kohler Christian G., Hodgins Sheilagh, Laurens Kristin R. Misperceptions of Facial Emotions Among Youth Aged 9–14 Years Who Present Multiple Antecedents of Schizophrenia. *Schizophrenia Bulletin*. 2014;40(2):460-468.

351. Dickson H, Cullen AE, Reichenberg A, et al. Cognitive impairment among children at-risk for schizophrenia. *Journal of Psychiatric Research*. 2014;50:92-99. doi:10.1016/j.jpsychires.2013.12.003

352. Dishy G, Kennedy L, Lundgren B, et al. Temporal relationship between onset of positive and negative symptoms in the course of attenuated psychotic illness. *Psychiatry Research*. 2020;293:113439. doi:10.1016/j.psychres.2020.113439

353. Diwadkar VA, Segel J, Pruitt P, et al. Hypo-activation in the executive core of the sustained attention network in adolescent offspring of schizophrenia patients mediated by premorbid functional deficits. *Psychiatry Research: Neuroimaging*. 2011;192(2):91-99. doi:10.1016/j.pscychresns.2010.12.005

354. Diwadkar VA, Pruitt P, Goradia D, et al. Fronto-parietal hypo-activation during working memory independent of structural abnormalities: Conjoint fMRI and sMRI analyses in adolescent offspring of schizophrenia patients. *Neuroimage*. 2011;58(1):234-241. doi:10.1016/j.neuroimage.2011.06.033

355. Diwadkar VA, Pruitt P, Zhang A, et al. The neural correlates of performance in adolescents at risk for schizophrenia: Inefficiently increased cortico-striatal responses measured with fMRI. *Journal of Psychiatric Research*. 2012;46(1):12-21. doi:10.1016/j.jpsychires.2011.09.016

356. Dodell-Feder D, DeLisi LE, Hooker CI. Neural disruption to theory of mind predicts daily social functioning in individuals at familial high-risk for schizophrenia. *Social Cognitive and Affective Neuroscience*. 2014;9(12). doi:10.1093/scan/nst186

357. Domingues I, Alderman T, Cadenhead KS. Strategies for effective recruitment of individuals at risk for developing psychosis. *Early Intervention in Psychiatry*. 2011;5(3):233-241. doi:10.1111/j.1751-7893.2011.00278.x

358. Domínguez-Martínez T, Medina-Pradas C, Kwapil TR, Barrantes-Vidal N. Relatives׳ illness attributions mediate the association of expressed emotion with early psychosis symptoms and functioning. *Psychiatry Research*. 2014;218(1-2):48-53. doi:10.1016/j.psychres.2014.04.012

359. Domínguez-Martínez T, Kwapil TR, Barrantes-Vidal N. Subjective quality of life in At-Risk Mental State for psychosis patients: relationship with symptom severity and functional impairment. *Early Intervention in Psychiatry*. 2015;9(4):292-299. doi:10.1111/eip.12111

360. Domínguez-Martínez T, Medina-Pradas C, Kwapil TR, Barrantes-Vidal N. Relatives’ expressed emotion, distress and attributions in clinical high-risk and recent onset of psychosis. *Psychiatry Research*. 2017;247:323-329. doi:10.1016/j.psychres.2016.11.048

361. Donkers FCL, Schwikert SR, Evans AM, Cleary KM, Perkins DO, Belger A. Impaired Neural Synchrony in the Theta Frequency Range in Adolescents at Familial Risk for Schizophrenia. *Frontiers in Psychiatry*. 2011;2. doi:10.3389/fpsyt.2011.00051

362. Dragt S, Nieman DH, Veltman D, et al. Environmental factors and social adjustment as predictors of a first psychosis in subjects at ultra high risk. *Schizophrenia Research*. 2011;125(1):69-76. doi:10.1016/j.schres.2010.09.007

363. Du Y, Fryer SL, Lin D, et al. Identifying functional network changing patterns in individuals at clinical high-risk for psychosis and patients with early illness schizophrenia: A group ICA study. *NeuroImage: Clinical*. 2018;17:335-346. doi:10.1016/j.nicl.2017.10.018

364. Du Y, Fryer SL, Fu Z, et al. Dynamic functional connectivity impairments in early schizophrenia and clinical high-risk for psychosis. *Neuroimage*. 2018;180:632-645. doi:10.1016/j.neuroimage.2017.10.022

365. Duffy FH, D’Angelo E, Rotenberg A, Gonzalez-Heydrich J. Neurophysiological differences between patients clinically at high risk for schizophrenia and neurotypical controls – first steps in development of a biomarker. *BMC Medicine*. 2015;13(1):276. doi:10.1186/s12916-015-0516-z

366. Dukart J, Smieskova R, Harrisberger F, et al. Age-related brain structural alterations as an intermediate phenotype of psychosis. *Journal of Psychiatry & Neuroscience*. 2017;42(5):307-319. doi:10.1503/jpn.160179

367. Eack SM, Mermon DE, Montrose DM, et al. Social cognition deficits among individuals at familial high risk for schizophrenia. *Schizophrenia Bulletin*. 2010;36(6):1081-1088. doi:10.1093/schbul/sbp026

368. Egerton A, Chaddock CA, Winton-Brown TT, et al. Presynaptic Striatal Dopamine Dysfunction in People at Ultra-high Risk for Psychosis: Findings in a Second Cohort. *Biological Psychiatry*. 2013;74(2):106-112. doi:10.1016/j.biopsych.2012.11.017

369. Egerton A, Stone JM, Chaddock CA, et al. Relationship Between Brain Glutamate Levels and Clinical Outcome in Individuals at Ultra High Risk of Psychosis. *Neuropsychopharmacology*. 2014;39(12):2891-2899. doi:10.1038/npp.2014.143

370. Egerton A, Valmaggia LR, Howes OD, et al. Adversity in childhood linked to elevated striatal dopamine function in adulthood. *Schizophrenia Research*. 2016;176(2-3):171-176. doi:10.1016/j.schres.2016.06.005

371. Egerton A, Howes OD, Houle S, et al. Elevated Striatal Dopamine Function in Immigrants and Their Children: A Risk Mechanism for Psychosis. *Schizophrenia Bulletin*. Published online January 5, 2017:sbw181. doi:10.1093/schbul/sbw181

372. Eggins PS, Hatton SN, Hermens DF, Hickie IB, Lagopoulos J. Subcortical volumetric differences between clinical stages of young people with affective and psychotic disorders. *Psychiatry Research: Neuroimaging*. 2018;271:8-16. doi:10.1016/j.pscychresns.2017.11.015

373. Egloff L, Lenz C, Studerus E, et al. No associations between medial temporal lobe volumes and verbal learning/memory in emerging psychosis. *European Journal of Neuroscience*. 2019;50(6):3060-3071. doi:10.1111/ejn.14427

374. Egloff L, Studerus E, Zimmermann R, et al. Evaluating verbal learning and memory in patients with an at-risk mental state or first episode psychosis using structural equation modelling. *PLOS ONE*. 2018;13(5):e0196936. doi:10.1371/journal.pone.0196936

375. Egloff L, Lenz C, Studerus E, et al. Sexually dimorphic subcortical brain volumes in emerging psychosis. *Schizophrenia Research*. 2018;199:257-265. doi:10.1016/j.schres.2018.03.034

376. Eisenacher S, Rausch F, Ainser F, et al. Investigation of metamemory functioning in the at-risk mental state for psychosis. *Psychological Medicine*. 2015;45(15):3329-3340. doi:10.1017/S0033291715001373

377. Eisenacher S, Rausch F, Mier D, et al. Bias against disconfirmatory evidence in the ‘at-risk mental state’ and during psychosis. *Psychiatry Research*. 2016;238:242-250. doi:10.1016/j.psychres.2016.02.028

378. Eisenacher S, Rausch F, Ainser F, et al. Early cognitive basic symptoms are accompanied by neurocognitive impairment in patients with an ‘at-risk mental state’ for psychosis. *Early Intervention in Psychiatry*. 2018;12(4):586-595. doi:10.1111/eip.12350

379. Ereshefsky SH. *Assessing Social and Role Functioning in Help-Seeking Youth at Clinical High-Risk for Psychosis with Comorbid Attention-Deficit/Hyperactivity Disorder (ADHD) and History of Stimulant Exposure: A Six-Month Longitudinal Follow-up Study.* 2019.

380. Ermakova AO, Knolle F, Justicia A, et al. Abnormal reward prediction-error signalling in antipsychotic naive individuals with first-episode psychosis or clinical risk for psychosis. *Neuropsychopharmacology*. 2018;43(8):1691-1699. doi:10.1038/s41386-018-0056-2

381. Eslami A, Jahshan C, Cadenhead KS. Disorganized Symptoms and Executive Functioning Predict Impaired Social Functioning in Subjects at Risk for Psychosis. *The Journal of Neuropsychiatry and Clinical Neurosciences*. 2011;23(4):457-460. doi:10.1176/jnp.23.4.jnp457

382. Esterberg M, Compton M. Family history of psychosis negatively impacts age at onset, negative symptoms, and duration of untreated illness and psychosis in first-episode psychosis patients. *Psychiatry Research*. 2012;197(1-2):23-28. doi:10.1016/j.psychres.2012.03.001

383. Esterberg ML, Ousley OY, Cubells JF, Walker EF. Prodromal and autistic symptoms in schizotypal personality disorder and 22q11.2 deletion syndrome. *Journal of Abnormal Psychology*. 2013;122(1):238-249. doi:10.1037/a0028373

384. Esterberg ML, Ousley OY, Cubells JF, Walker EF. Prodromal and autistic symptoms in schizotypal personality disorder and 22q11.2 deletion syndrome. *Journal of Abnormal Psychology*. 2013;122(1):238-249. doi:10.1037/a0028373

385. Falkenberg I, Chaddock C, Murray RM, et al. Failure to deactivate medial prefrontal cortex in people at high risk for psychosis. *European Psychiatry*. 2015;30(5):633-640. doi:10.1016/j.eurpsy.2015.03.003

386. Falkenberg I, Valli I, Raffin M, et al. Pattern of activation during delayed matching to sample task predicts functional outcome in people at ultra high risk for psychosis. *Schizophrenia Research*. 2017;181:86-93. doi:10.1016/j.schres.2016.09.023

387. Falukozi E, Addington J. Impact of Trauma on Attenuated Psychotic Symptoms. *Psychosis*. 2012;4(3):203-212. doi:10.1080/17522439.2011.62686712

388. Farrell M, Boys A, Bebbington P, et al. Psychosis and drug dependence: results from a national survey of prisoners. *British Journal of Psychiatry*. 2002;181(5):393-398. doi:10.1192/bjp.181.5.393

389. Feng X, Provenzano F, Appelbaum PS, et al. Amygdalar volume and violent ideation in a sample at clinical high-risk for psychosis. *Psychiatry Research: Neuroimaging*. 2019;287:60-62. doi:10.1016/j.pscychresns.2019.04.003

390. Fernandez VG, Asarnow R, Narr KL, et al. Temporal lobe thickness and verbal memory in first-degree relatives of individuals with schizophrenia. *Schizophrenia Research*. 2018;199:221-225. doi:10.1016/j.schres.2018.02.038

391. Flückiger R, Michel C, Grant P, et al. The interrelationship between schizotypy, clinical high risk for psychosis and related symptoms: Cognitive disturbances matter. *Schizophrenia Research*. 2019;210:188-196. doi:10.1016/j.schres.2018.12.039

392. Flynn D, Smith D, Quirke L, Monks S, Kennedy HG. Ultra high risk of psychosis on committal to a young offender prison: an unrecognised opportunity for early intervention. *BMC Psychiatry*. 2012;12(1):100. doi:10.1186/1471-244X-12-100

393. Föcking M, Dicker P, Lopez LM, et al. Differential expression of the inflammation marker IL12p40 in the at-risk mental state for psychosis: a predictor of transition to psychotic disorder? *BMC Psychiatry*. 2016;16(1):326. doi:10.1186/s12888-016-1039-7

394. Fonseca-Pedrero E, Paino M, Ortuño-Sierra J, Lemos-Giráldez S, Muñiz J. The assessment of positive dimension of the psychosis phenotype in college students. *Comprehensive Psychiatry*. 2014;55(3):699-707. doi:10.1016/j.comppsych.2013.09.013

395. Fonseca-Pedrero E, Lemos-Giráldez S, Paino M, Sierra-Baigrie S, Santarén Rosell M, Muñiz J. Internal structure and reliability of the Oviedo Schizotypy Assessment Questionnaire (ESQUIZO-Q). *International Journal of Clinical and Health Psychology*. 2011;11:385-402.

396. Fornito A, Yung AR, Wood SJ, et al. Anatomic Abnormalities of the Anterior Cingulate Cortex Before Psychosis Onset: An MRI Study of Ultra-High-Risk Individuals. *Biological Psychiatry*. 2008;64(9):758-765. doi:10.1016/j.biopsych.2008.05.032

397. Freeman D, Pugh K, Green C, Valmaggia L, Dunn G, Garety P. A measure of state persecutory ideation for experimental studies. *Journal of Nervous and Mental Disease*. 2007;195(9):781-784. doi:10.1097/NMD.0b013e318145a0a9

398. French P, Shryane N, Bentall RP, Lewis SW, Morrison AP. Effects of cognitive therapy on the longitudinal development of psychotic experiences in people at high risk of developing psychosis. *British Journal of Psychiatry*. 2007;191(S51). doi:10.1192/bjp.191.51.s82

399. French P, Owens J, Parker S, Dunn G. Identification of young people in the early stages of psychosis: Validation of a checklist for use in primary care. *Psychiatry Research*. 2012;200(2-3):911-916. doi:10.1016/j.psychres.2012.07.040

400. Fresan A, Apiquián R, Ulloa RE, Nicolini H. Reliability study of the translation into Spanish of the PRIME Screen Questionnaire for Prodromic Symtoms. *Actas espanolas de psiquiatria*. 35(6):368-371.

401. Fresán A, León-Ortiz P, Robles-García R, et al. Personality features in ultra-high risk for psychosis: A comparative study with schizophrenia and control subjects using the Temperament and Character Inventory-Revised (TCI-R). *Journal of Psychiatric Research*. 2015;61:168-173. doi:10.1016/j.jpsychires.2014.12.013

402. Fridgen GJ, Aston J, Gschwandtner U, et al. Help-seeking and pathways to care in the early stages of psychosis. *Social Psychiatry and Psychiatric Epidemiology*. 2013;48(7):1033-1043. doi:10.1007/s00127-012-0628-0

403. Friedman-Yakoobian MS, Parrish EM, Eack SM, Keshavan MS. Neurocognitive and social cognitive training for youth at clinical high risk (CHR) for psychosis: A randomized controlled feasibility trial. *Schizophrenia Research*. Published online 2020. doi:10.1016/j.schres.2020.09.005

404. Friedman-Yakoobian M, Parrish EM, Thomas A, et al. An integrated neurocognitive and social-cognitive treatment for youth at clinical high risk for psychosis: Cognition for Learning and for Understanding Everyday Social Situations (CLUES). *Schizophrenia Research*. 2019;208:55-59. doi:10.1016/j.schres.2019.01.029

405. FRIESEN P, LAWRENCE RE, BRUCATO G, GIRGIS RR, DIXON L. Hopes and Expectations Regarding Genetic Testing for Schizophrenia Among Young Adults at Clinical High-Risk for Psychosis. *Journal of Psychiatric Practice*. 2016;22(6):442-449. doi:10.1097/PRA.0000000000000188

406. Frommann I, Brinkmeyer J, Ruhrmann S, et al. Auditory P300 in individuals clinically at risk for psychosis. *International Journal of Psychophysiology*. 2008;70(3):192-205. doi:10.1016/j.ijpsycho.2008.07.003

407. Woodberry KA, Seidman LJ, Giuliano AJ, Verdi MB, Cook WL, McFarlane WR. Neuropsychological profiles in individuals at clinical high risk for psychosis: Relationship to psychosis and intelligence. *Schizophrenia Research*. 2010;123(2-3):188-198. doi:10.1016/j.schres.2010.06.021

408. Frumin M, Golland P, Kikinis R, et al. Shape Differences in the Corpus Callosum in First-Episode Schizophrenia and First-Episode Psychotic Affective Disorder. *American Journal of Psychiatry*. 2002;159(5):866-868. doi:10.1176/appi.ajp.159.5.866

409. Fryer SL, Woods SW, Kiehl KA, et al. Deficient Suppression of Default Mode Regions during Working Memory in Individuals with Early Psychosis and at Clinical High-Risk for Psychosis. *Frontiers in Psychiatry*. 2013;4. doi:10.3389/fpsyt.2013.00092

410. Fryer SL, Roach BJ, Wiley K, Loewy RL, Ford JM, Mathalon DH. Reduced Amplitude of Low-Frequency Brain Oscillations in the Psychosis Risk Syndrome and Early Illness Schizophrenia. *Neuropsychopharmacology*. 2016;41(9):2388-2398. doi:10.1038/npp.2016.51

411. Fryer SL, Roach BJ, Ford JM, et al. Should I Stay or Should I Go? FMRI Study of Response Inhibition in Early Illness Schizophrenia and Risk for Psychosis. *Schizophrenia Bulletin*. 2019;45(1):158-168. doi:10.1093/schbul/sbx198

412. Fryer SL, Roach BJ, Hamilton HK, et al. Deficits in auditory predictive coding in individuals with the psychosis risk syndrome: Prediction of conversion to psychosis. *Journal of Abnormal Psychology*. 2020;129(6):599-611. doi:10.1037/abn0000513

413. Fulford D, Pearson R, Stuart BK, et al. Symptom assessment in early psychosis: The use of well-established rating scales in clinical high-risk and recent-onset populations. *Psychiatry Research*. 2014;220(3):1077-1083. doi:10.1016/j.psychres.2014.07.047

414. Fulford D, Niendam TA, Floyd EG, et al. Symptom dimensions and functional impairment in early psychosis: More to the story than just negative symptoms. *Schizophrenia Research*. 2013;147(1):125-131. doi:10.1016/j.schres.2013.03.024

415. Fusar-Poli P, Rutigliano G, Stahl D, et al. Deconstructing Pretest Risk Enrichment to Optimize Prediction of Psychosis in Individuals at Clinical High Risk. *JAMA Psychiatry*. 2016;73(12):1260. doi:10.1001/jamapsychiatry.2016.2707

416. Fusar-Poli P, Hobson R, Raduelli M, Balottin U. Reliability and Validity of the Comprehensive Assessment of the at Risk Mental State, Italian Version (CAARMS-I). *Current Pharmaceutical Design*. 2012;18(4). doi:10.2174/138161212799316118

417. Fusar-Poli P, Meneghelli A, Valmaggia L, et al. Duration of untreated prodromal symptoms and 12-month functional outcome of individuals at risk of psychosis. *British Journal of Psychiatry*. 2009;194(2):181-182. doi:10.1192/bjp.bp.107.047951

418. Fusar-Poli P, Howes OD, McGuire P. Pseudohallucinations Versus True Hallucinations in Prodromal Psychosis. *The Journal of Clinical Psychiatry*. 2009;70(7):1056-1057. doi:10.4088/JCP.08l04848

419. Fusar-Poli P, Broome MR, Matthiasson P, et al. Spatial working memory in individuals at high risk for psychosis: Longitudinal fMRI study. *Schizophrenia Research*. 2010;123(1):45-52. doi:10.1016/j.schres.2010.06.008

420. Fusar-Poli P, Byrne M, Valmaggia L, et al. Social dysfunction predicts two years clinical outcome in people at ultra high risk for psychosis. *Journal of Psychiatric Research*. 2010;44(5):294-301. doi:10.1016/j.jpsychires.2009.08.016

421. Fusar-Poli P, Broome MR, Woolley JB, et al. Altered brain function directly related to structural abnormalities in people at ultra high risk of psychosis: Longitudinal VBM-fMRI study. *Journal of Psychiatric Research*. 2011;45(2):190-198. doi:10.1016/j.jpsychires.2010.05.012

422. Fusar-Poli P, Crossley N, Woolley J, et al. Gray matter alterations related to P300 abnormalities in subjects at high risk for psychosis: Longitudinal MRI-EEG study. *Neuroimage*. 2011;55(1):320-328. doi:10.1016/j.neuroimage.2010.11.075

423. Fusar-Poli P, Crossley N, Woolley J, et al. White matter alterations related to P300 abnormalities in individuals at high risk for psychosis: an MRI–EEG study. *Journal of Psychiatry and Neuroscience*. 2011;36(4):239-248. doi:10.1503/jpn.100083

424. Fusar-Poli P. Thalamic Glutamate Levels as a Predictor of Cortical Response During Executive Functioning in Subjects at High Risk for Psychosis. *Archives of General Psychiatry*. 2011;68(9):881. doi:10.1001/archgenpsychiatry.2011.46

425. Fusar-Poli P, Broome MR, Matthiasson P, et al. Prefrontal Function at Presentation Directly Related to Clinical Outcome in People at Ultrahigh Risk of Psychosis. *Schizophrenia Bulletin*. 2011;37(1):189-198. doi:10.1093/schbul/sbp074

426. Fusar-Poli P, Howes OD, Allen P, et al. Abnormal Frontostriatal Interactions in People With Prodromal Signs of Psychosis. *Archives of General Psychiatry*. 2010;67(7):683. doi:10.1001/archgenpsychiatry.2010.77

427. Fusar-Poli P, Howes OD, Allen P, et al. Abnormal prefrontal activation directly related to pre-synaptic striatal dopamine dysfunction in people at clinical high risk for psychosis. *Molecular Psychiatry*. 2011;16(1):67-75. doi:10.1038/mp.2009.108

428. Fusar-Poli P. Prodromal Psychosis: Diagnosis and Treatment. *Current Pharmaceutical Design*. 2012;18(4). doi:10.2174/138161212799316154

429. Fusar-Poli P, Nelson B, Valmaggia L, Yung AR, McGuire PK. Comorbid Depressive and Anxiety Disorders in 509 Individuals With an At-Risk Mental State: Impact on Psychopathology and Transition to Psychosis. *Schizophrenia Bulletin*. 2014;40(1):120-131. doi:10.1093/schbul/sbs136

430. Fusar-Poli P, Rutigliano G, Stahl D, et al. Long-term validity of the At Risk Mental State (ARMS) for predicting psychotic and non-psychotic mental disorders. *European Psychiatry*. 2017;42:49-54. doi:10.1016/j.eurpsy.2016.11.010

431. Fusar-Poli P, de Micheli A, Cappucciati M, et al. Diagnostic and Prognostic Significance of DSM-5 Attenuated Psychosis Syndrome in Services for Individuals at Ultra High Risk for Psychosis. *Schizophrenia Bulletin*. 2018;44(2):264-275. doi:10.1093/schbul/sbx055

432. Fusar-Poli P, de Micheli A, Rocchetti M, et al. Semistructured Interview for Bipolar At Risk States (SIBARS). *Psychiatry Research*. 2018;264:302-309. doi:10.1016/j.psychres.2018.03.074

433. Fusar-Poli P, Palombini E, Davies C, et al. Why transition risk to psychosis is not declining at the OASIS ultra high risk service: The hidden role of stable pretest risk enrichment. *Schizophrenia Research*. 2018;192:385-390. doi:10.1016/j.schres.2017.06.015

434. Fusar-Poli P. Specialized services for individuals at clinical high risk for psychosis target simultaneously adolescents and young adults. *International Journal of Technology Assessment in Health Care*. 2019;35(5):408-409. doi:10.1017/S0266462319000692

435. Fusar-Poli P, Werbeloff N, Rutigliano G, et al. Transdiagnostic Risk Calculator for the Automatic Detection of Individuals at Risk and the Prediction of Psychosis: Second Replication in an Independent National Health Service Trust. *Schizophrenia Bulletin*. 2019;45(3):562-570. doi:10.1093/schbul/sby070

436. Fusar-Poli P, de Micheli A, Chalambrides M, Singh A, Augusto C, McGuire P. Unmet needs for treatment in 102 individuals with brief and limited intermittent psychotic symptoms (BLIPS): implications for current clinical recommendations. *Epidemiology and Psychiatric Sciences*. 2020;29:e67. doi:10.1017/S2045796019000635

437. Gaag M, Eurelings‐Bontekoe L, Ising H, Berg D. Ultrahigh risk for developing psychosis and psychotic personality organization. *Early Intervention in Psychiatry*. 2019;13(3):673-676. doi:10.1111/eip.12687

438. Gajwani R, Patterson P, Birchwood M. Attachment: Developmental pathways to affective dysregulation in young people at ultra-high risk of developing psychosis. *British Journal of Clinical Psychology*. 2013;52(4):424-437. doi:10.1111/bjc.12027

439. Galletly C, van Hooff M, McFarlane A. Psychotic symptoms in young adults exposed to childhood trauma—A 20year follow-up study. *Schizophrenia Research*. 2011;127(1-3):76-82. doi:10.1016/j.schres.2010.12.010

440. Garyfallos G, Lavrentiadis G, Giouzepas J. Psychosis risk syndrome: pharmacological interventions. *Psychiatrike = Psychiatriki*. 22(4).

441. Gattere G, Stojanovic-Pérez A, Monseny R, et al. Gene-environment interaction between the brain-derived neurotrophic factor Val66Met polymorphism, psychosocial stress and dietary intake in early psychosis. *Early Intervention in Psychiatry*. 2018;12(5):811-820. doi:10.1111/eip.12371

442. Gaudiano BA, Zimmerman M. Prevalence of Attenuated Psychotic Symptoms and Their Relationship With *DSM-IV* Diagnoses in a General Psychiatric Outpatient Clinic. *The Journal of Clinical Psychiatry*. 2013;74(02):149-155. doi:10.4088/JCP.12m07788

443. Gawęda Ł, Li E, Lavoie S, Whitford TJ, Moritz S, Nelson B. Impaired action self-monitoring and cognitive confidence among ultra-high risk for psychosis and first-episode psychosis patients. *European Psychiatry*. 2018;47:67-75. doi:10.1016/j.eurpsy.2017.09.003

444. Gee DG, Karlsgodt KH, van Erp TGM, et al. Altered age-related trajectories of amygdala-prefrontal circuitry in adolescents at clinical high risk for psychosis: A preliminary study. *Schizophrenia Research*. 2012;134(1):1-9. doi:10.1016/j.schres.2011.10.005

445. Gee D. *Amygdala-Prefrontal Function and Clinical Course among Adolescents and Young Adults at Clinical High Risk for Psychosis*.; 2016. https://escholarship.org/uc/item/41n0x4rm

446. Georgopoulos G, Stowkowy J, Liu L, et al. The role of a family history of psychosis for youth at clinical high risk of psychosis. *Early Intervention in Psychiatry*. 2019;13(2):251-256. doi:10.1111/eip.12471

447. Geraets CNW, van Beilen M, Pot-Kolder R, Counotte J, van der Gaag M, Veling W. Social environments and interpersonal distance regulation in psychosis: A virtual reality study. *Schizophrenia Research*. 2018;192:96-101. doi:10.1016/j.schres.2017.04.034

448. Gerritsen C, Bagby RM, Sanches M, et al. Stress precedes negative symptom exacerbations in clinical high risk and early psychosis: A time-lagged experience sampling study. *Schizophrenia Research*. 2019;210:52-58. doi:10.1016/j.schres.2019.06.015

449. Gerson R, Wong C, Davidson L, Malaspina D, McGlashan T, Corcoran C. Self-reported coping strategies in families of patients in early stages of psychotic disorder: an exploratory study. *Early Intervention in Psychiatry*. 2011;5(1):76-80. doi:10.1111/j.1751-7893.2010.00251.x

450. Gerstenberg M, Hauser M, Al-Jadiri A, et al. Frequency and correlates of DSM-5 attenuated psychosis syndrome in a sample of adolescent inpatients with nonpsychotic psychiatric disorders. *The Journal of Clinical Psychiatry*. 2015;76(11):e1449-e1458. doi:10.4088/JCP.14m09435

451. Gibson CM, Penn DL, Prinstein MJ, Perkins DO, Belger A. Social skill and social cognition in adolescents at genetic risk for psychosis. *Schizophrenia Research*. 2010;122(1-3):179-184. doi:10.1016/j.schres.2010.04.018

452. Gibson LE, Anglin DM, Klugman JT, et al. Stress sensitivity mediates the relationship between traumatic life events and attenuated positive psychotic symptoms differentially by gender in a college population sample. *Journal of Psychiatric Research*. 2014;53:111-118. doi:10.1016/j.jpsychires.2014.02.020

453. Gifford G, Crossley N, Morgan S, et al. Integrated metastate functional connectivity networks predict change in symptom severity in clinical high risk for psychosis. *Human Brain Mapping*. 2021;42(2):439-451. doi:10.1002/hbm.25235

454. Gill KE, Poe L, Azimov N, et al. Reasons for cannabis use among youths at ultra high risk for psychosis. *Early Intervention in Psychiatry*. 2015;9(3):207-210. doi:10.1111/eip.12112

455. Gill KE, Cressman V, Poe SL, et al. Social inference in individuals at clinical high risk for psychosis. *Early Intervention in Psychiatry*. 2016;10(1):77-80. doi:10.1111/eip.12182

456. Gleeson JF, Rawlings D, Jackson HJ, McGorry PD. Early warning signs of relapse following a first episode of psychosis. *Schizophrenia Research*. 2005;80(1):107-111. doi:10.1016/j.schres.2005.07.019

457. Glenthøj LB, Fagerlund B, Bak N, et al. Examining speed of processing of facial emotion recognition in individuals at ultra-high risk for psychosis: Associations with symptoms and cognition. *Schizophrenia Research*. 2018;195:562-563. doi:10.1016/j.schres.2017.10.032

458. Glenthøj LB, Albert N, Fagerlund B, et al. Emotion recognition latency, but not accuracy, relates to real life functioning in individuals at ultra-high risk for psychosis. *Schizophrenia Research*. 2019;210:197-202. doi:10.1016/j.schres.2018.12.038

459. Goghari VM, Brett C, Tabraham P, et al. Spatial working memory ability in individuals at ultra high risk for psychosis. *Journal of Psychiatric Research*. 2014;50. doi:10.1016/j.jpsychires.2013.12.010

460. Goines K. *Sleep Problems and Positive Prodromal Symptoms.* PhD Dissertation Thesis. Laney Graduate School; 2018.

461. Goines KB, LoPilato AM, Addington J, et al. Sleep problems and attenuated psychotic symptoms in youth at clinical high-risk for psychosis. *Psychiatry Research*. 2019;282:112492. doi:10.1016/j.psychres.2019.112492

462. Goldenberg PC, Calkins ME, Richard J, et al. Computerized neurocognitive profile in young people with 22q11.2 deletion syndrome compared to youths with schizophrenia and At‐Risk for psychosis. *American Journal of Medical Genetics Part B: Neuropsychiatric Genetics*. 2012;159B(1):87-93. doi:10.1002/ajmg.b.32005

463. Goldsmith DR, Haroon E, Miller AH, et al. Association of baseline inflammatory markers and the development of negative symptoms in individuals at clinical high risk for psychosis. *Brain, Behavior, and Immunity*. 2019;76:268-274. doi:10.1016/j.bbi.2018.11.315

464. Goldstein JM, Buka SL, Seidman LJ, Tsuang MT. Specificity of Familial Transmission of Schizophrenia Psychosis Spectrum and Affective Psychoses in the New England Family Study’s High-Risk Design. *Archives of General Psychiatry*. 2010;67(5):458. doi:10.1001/archgenpsychiatry.2010.38

465. Goldstein JM, Cherkerzian S, Seidman LJ, et al. Sex-specific rates of transmission of psychosis in the New England high-risk family study. *Schizophrenia Research*. 2011;128(1-3):150-155. doi:10.1016/j.schres.2011.01.019

466. Golembo-Smith S, Bachman P, Senturk D, Cannon TD, Bearden CE. Youth-caregiver Agreement on Clinical High-risk Symptoms of Psychosis. *Journal of Abnormal Child Psychology*. 2014;42(4):649-658. doi:10.1007/s10802-013-9809-x

467. Gonçalves PD, Martins PA, Gordon P, Louzã M. Prodromal Questionnaire: translation, adaptation to Portuguese and preliminary results in ultra-high risk individuals and first episode psychosis. *Jornal Brasileiro de Psiquiatria*. 2012;61(2):96-101. doi:10.1590/S0047-20852012000200007

468. Gonzalez-Heydrich J, Bosquet Enlow M, D’Angelo E, et al. Early auditory processing evoked potentials (N100) show a continuum of blunting from clinical high risk to psychosis in a pediatric sample. *Schizophrenia Research*. 2015;169(1-3):340-345. doi:10.1016/j.schres.2015.10.037

469. Gonzalez-Heydrich J, Bosquet Enlow M, D’Angelo E, et al. N100 Repetition Suppression Indexes Neuroplastic Defects in Clinical High Risk and Psychotic Youth. *Neural Plasticity*. 2016;2016:1-11. doi:10.1155/2016/4209831

470. González-Rodríguez A, Studerus E, Spitz A, et al. Gender differences in the psychopathology of emerging psychosis. *Isr J Psychiatry Relat Sci*. 2014;51(2).

471. Gooding DC, Shea HB, Matts CW. Saccadic performance in questionnaire-identified schizotypes over time. *Psychiatry Research*. 2005;133(2-3):173-186. doi:10.1016/j.psychres.2003.12.029

472. Gothelf D, Law AJ, Frisch A, et al. Biological Effects of COMT Haplotypes and Psychosis Risk in 22q11.2 Deletion Syndrome. *Biological Psychiatry*. 2014;75(5). doi:10.1016/j.biopsych.2013.07.021

473. Gothelf D, Hoeft F, Ueno T, et al. Developmental changes in multivariate neuroanatomical patterns that predict risk for psychosis in 22q11.2 deletion syndrome. *Journal of Psychiatric Research*. 2011;45(3):322-331. doi:10.1016/j.jpsychires.2010.07.008

474. Gottlieb JD. *Aspects of Cognitive Vulnerability as Predictive of General and Specific Themes of Delusional Ideation in Individuals at Risk for Psychosis*. 2004.

475. Goulding SM. *Social Cognitive Performance and the Psychosis-Spectrum Prodrome*. 2015.

476. Gourzis P, Katrivanou A, Beratis S. *Symptomatology of the Initial Prodromal Phase in Schizophrenia*. https://academic.oup.com/schizophreniabulletin/article/28/3/415/1839390

477. Gouzoulis-Mayfrank E, Balke M, Hajsamou S, et al. Orienting of attention in unmedicated patients with schizophrenia, prodromal subjects and healthy relatives. *Schizophrenia Research*. 2007;97(1-3):35-42. doi:10.1016/j.schres.2007.06.028

478. von Reventlow HG, Krüger-Özgürdal S, Ruhrmann S, et al. Pathways to care in subjects at high risk for psychotic disorders — A European perspective. *Schizophrenia Research*. 2014;152(2-3). doi:10.1016/j.schres.2013.11.031

479. Granö N, Karjalainen M, Edlund V, et al. Depression symptoms in help-seeking adolescents: A comparison between adolescents at-risk for psychosis and other help-seekers. *Journal of Mental Health*. 2013;22(4):317-324. doi:10.3109/09638237.2012.734654

480. Granö N, Karjalainen M, Edlund V, et al. Health-related quality of life among adolescents: a comparison between subjects at risk for psychosis and other help seekers. *Early Intervention in Psychiatry*. 2014;8(2). doi:10.1111/eip.12033

481. Granö N, Karjalainen M, Anto J, Itkonen A, Edlund V, Roine M. Intervention to improve level of overall functioning and mental condition of adolescents at high risk of developing first-episode psychosis in Finland. *Early Intervention in Psychiatry*. 2009;3(2):94-98. doi:10.1111/j.1751-7893.2009.00114.x

482. Granö N, Karjalainen M, Suominen K, Roine M. Poor functioning ability is associated with high risk of developing psychosis in adolescents. *Nordic Journal of Psychiatry*. 2011;65(1):16-21. doi:10.3109/08039488.2010.483743

483. Granö N, Karjalainen M, Anto J, Itkonen A, Edlund V, Roine M. Associations between number of different type of care meetings with social network and improvement in mental well-being in adolescents at risk of first-episode psychosis. *Early Intervention in Psychiatry*. 2011;5(3):212-218. doi:10.1111/j.1751-7893.2011.00269.x

484. Granö N, Karjalainen M, Itkonen A, et al. Differential results between self-report and interview-based ratings of risk symptoms of psychosis. *Early Intervention in Psychiatry*. 2011;5(4):309-314. doi:10.1111/j.1751-7893.2011.00266.x

485. Granö N, Karjalainen M, Edlund V, et al. Changes in health-related quality of life and functioning ability in help-seeking adolescents and adolescents at heightened risk of developing psychosis during family- and community-oriented intervention model. *International Journal of Psychiatry in Clinical Practice*. 2013;17(4):253-258. doi:10.3109/13651501.2013.784791

486. Granö N, Karjalainen M, Edlund V, et al. Adolescents at risk of psychosis have higher level of hopelessness than adolescents not at risk of psychosis. *Nordic Journal of Psychiatry*. 2013;67(4):258-264. doi:10.3109/08039488.2012.735253

487. Granö N, Karjalainen M, Edlund V, et al. Anxiety symptoms in adolescents at risk for psychosis: a comparison among help seekers. *Child and Adolescent Mental Health*. 2014;19(2):97-101. doi:10.1111/camh.12012

488. Granö N, Karjalainen M, Ranta K, Lindgren M, Roine M, Therman S. Community-oriented family-based intervention superior to standard treatment in improving depression, hopelessness and functioning among adolescents with any psychosis-risk symptoms. *Psychiatry Research*. 2016;237:9-16. doi:10.1016/j.psychres.2016.01.037

489. Green CEL, McGuire PK, Ashworth M, Valmaggia LR. Outreach and Support in South London (OASIS). Outcomes of non-attenders to a service for people at high risk of psychosis: the case for a more assertive approach to assessment. *Psychological Medicine*. 2011;41(2):243-250. doi:10.1017/S0033291710000723

490. Green MF, Bearden CE, Cannon TD, et al. Social Cognition in Schizophrenia, Part 1: Performance Across Phase of Illness. *Schizophrenia Bulletin*. 2012;38(4):854-864. doi:10.1093/schbul/sbq171

491. Greenhalgh KT, Shanley DC. Recognising an at Risk Mental State for Psychosis: Australian Lay People and Clinicians’ Ability to Identify a Problem and Recommend Help Across Vignette Types. *Australian Psychologist*. 2017;52(6):524-532. doi:10.1111/ap.12238

492. Greenland-White SE, Ragland JD, Niendam TA, Ferrer E, Carter CS. Episodic memory functions in first episode psychosis and clinical high risk individuals. *Schizophrenia Research*. 2017;188:151-157. doi:10.1016/j.schres.2017.01.035

493. Grent-’t-Jong T, Gross J, Goense J, et al. Resting-state gamma-band power alterations in schizophrenia reveal E/I-balance abnormalities across illness-stages. *Elife*. 2018;7. doi:10.7554/eLife.37799

494. Grossman MJ, Woolridge S, Lichtenstein S, et al. Patterns and perceptions of face-to-face and digital communication in the clinical high risk and early stages of psychosis. *Psychiatry Research*. 2020;284:112667. doi:10.1016/j.psychres.2019.112667

495. Gruber J, Strauss GP, Dombrecht L, Mittal VA. Neuroleptic-free youth at ultrahigh risk for psychosis evidence diminished emotion reactivity that is predicted by depression and anxiety. *Schizophrenia Research*. 2018;193:428-434. doi:10.1016/j.schres.2017.08.013

496. Gschwandtner U, Aston J, Borgwardt S, et al. Neuropsychological and neurophysiological findings in individuals suspected to be at risk for schizophrenia: preliminary results from the Basel early detection of psychosis study - Früherkennung von Psychosen (FEPSY). *Acta Psychiatrica Scandinavica*. 2003;108(2):152-155. doi:10.1034/j.1600-0447.2003.00157.x

497. Gschwandtner U, Pflüger M, Aston J, et al. Fine motor function and neuropsychological deficits in individuals at risk for schizophrenia. *European Archives of Psychiatry and Clinical Neuroscience*. 2006;256(4):201-206. doi:10.1007/s00406-005-0626-2

498. Gschwandtner U, Pflueger MO, Semenin V, Gaggiotti M, Riecher-Rössler A, Fuhr P. EEG: A helpful tool in the prediction of psychosis. *European Archives of Psychiatry and Clinical Neuroscience*. 2009;259(5):257-262. doi:10.1007/s00406-008-0854-3

499. Gudlowski Y, Özgürdal S, Witthaus H, et al. Serotonergic dysfunction in the prodromal, first-episode and chronic course of schizophrenia as assessed by the loudness dependence of auditory evoked activity. *Schizophrenia Research*. 2009;109(1-3):141-147. doi:10.1016/j.schres.2009.02.008

500. Guma E, Devenyi GA, Malla A, Shah J, Chakravarty MM, Pruessner M. Neuroanatomical and Symptomatic Sex Differences in Individuals at Clinical High Risk for Psychosis. *Frontiers in Psychiatry*. 2017;8. doi:10.3389/fpsyt.2017.00291

501. Gunnell D, Harrison G, Rasmussen F, Fouskakis D, Tynelius P. Associations between premorbid intellectual performance, early-life exposures and early-onset schizophrenia. *British Journal of Psychiatry*. 2002;181(4):298-305. doi:10.1192/bjp.181.4.298

502. Guo JY, Niendam TA, Auther AM, et al. Predicting psychosis risk using a specific measure of cognitive control: a 12-month longitudinal study. *Psychological Medicine*. 2020;50(13):2230-2239. doi:10.1017/S0033291719002332

503. Gupta T, Mittal VA. Nicotine usage is associated with elevated processing speed, spatial working memory, and visual learning performance in youth at ultrahigh-risk for psychosis. *Psychiatry Research*. 2014;220(1-2):687-690. doi:10.1016/j.psychres.2014.07.085

504. Gupta T, Silverstein SM, Bernard JA, et al. Disruptions in neural connectivity associated with reduced susceptibility to a depth inversion illusion in youth at ultra high risk for psychosis. *NeuroImage: Clinical*. 2016;12:681-690. doi:10.1016/j.nicl.2016.09.022

505. Gupta T, Hespos SJ, Horton WS, Mittal VA. Automated analysis of written narratives reveals abnormalities in referential cohesion in youth at ultra high risk for psychosis. *Schizophrenia Research*. 2018;192:82-88. doi:10.1016/j.schres.2017.04.025

506. Gupta T, Haase CM, Strauss GP, Cohen AS, Mittal VA. Alterations in facial expressivity in youth at clinical high-risk for psychosis. *Journal of Abnormal Psychology*. 2019;128(4):341-351. doi:10.1037/abn0000413

507. Gupta T, Haase CM, Strauss GP, Cohen AS, Ricard JR, Mittal VA. Alterations in facial expressions of emotion: Determining the promise of ultrathin slicing approaches and comparing human and automated coding methods in psychosis risk. *Emotion*. Published online June 25, 2020. doi:10.1037/emo0000819

508. Gur RC, Calkins ME, Satterthwaite TD, et al. Neurocognitive Growth Charting in Psychosis Spectrum Youths. *JAMA Psychiatry*. 2014;71(4):366. doi:10.1001/jamapsychiatry.2013.4190

509. Gur RE, March M, Calkins ME, et al. Negative symptoms in youths with psychosis spectrum features: Complementary scales in relation to neurocognitive performance and function. *Schizophrenia Research*. 2015;166(1-3):322-327. doi:10.1016/j.schres.2015.05.037

510. Gureje O, Bamidele R, Aderibigbe YA. Heritability of Schizophrenia. *British Journal of Psychiatry*. 1994;164(4):481-486. doi:10.1192/bjp.164.4.481

511. Haarsma J, Knolle F, Griffin JD, et al. Influence of prior beliefs on perception in early psychosis: Effects of illness stage and hierarchical level of belief. *Journal of Abnormal Psychology*. 2020;129(6):581-598. doi:10.1037/abn0000494

512. Haas SS, Doucet GE, Garg S, et al. Linking language features to clinical symptoms and multimodal imaging in individuals at clinical high risk for psychosis. *European Psychiatry*. 2020;63(1). doi:10.1192/j.eurpsy.2020.73

513. Hafizi S, da Silva T, Gerritsen C, et al. Imaging Microglial Activation in Individuals at Clinical High Risk for Psychosis: an In Vivo PET Study with [18F]FEPPA. *Neuropsychopharmacology*. 2017;42(13):2474-2481. doi:10.1038/npp.2017.111

514. Hafizi S, Guma E, Koppel A, et al. TSPO expression and brain structure in the psychosis spectrum. *Brain, Behavior, and Immunity*. 2018;74:79-85. doi:10.1016/j.bbi.2018.06.009

515. Hafizi S, da Silva T, Meyer JH, et al. Interaction between TSPO—a neuroimmune marker—and redox status in clinical high risk for psychosis: a PET–MRS study. *Neuropsychopharmacology*. 2018;43(8):1700-1705. doi:10.1038/s41386-018-0061-5

516. Hagenmuller F, Heekeren K, Theodoridou A, et al. Early somatosensory processing in individuals at risk for developing psychoses. *Frontiers in Behavioral Neuroscience*. 2014;8. doi:10.3389/fnbeh.2014.00308

517. Hagenmuller F, Heekeren K, Roser P, et al. Early Somatosensory Processing Over Time in Individuals at Risk to Develop Psychosis. *Frontiers in Psychiatry*. 2019;10. doi:10.3389/fpsyt.2019.00047

518. Haidl T, Rosen M, Schultze-Lutter F, et al. Expressed emotion as a predictor of the first psychotic episode — Results of the European prediction of psychosis study. *Schizophrenia Research*. 2018;199:346-352. doi:10.1016/j.schres.2018.03.019

519. Haining K, Karagiorgou O, Gajwani R, et al. Prevalence and predictors of suicidality and non‐suicidal self‐harm among individuals at clinical high‐risk for psychosis: Results from a community‐recruited sample. *Early Intervention in Psychiatry*. 2021;15(5):1256-1265. doi:10.1111/eip.13075

520. Haller S, Borgwardt SJ, Schindler C, Aston J, Radue EW, Riecher-Rössler A. Can Cortical Thickness Asymmetry Analysis Contribute to Detection of At-Risk Mental State and First-Episode Psychosis?: A Pilot Study. *Radiology*. 2009;250(1). doi:10.1148/radiol.2501072153

521. Hamaie Y, Ohmuro N, Katsura M, et al. Criticism and Depression among the Caregivers of At-Risk Mental State and First-Episode Psychosis Patients. *PLOS ONE*. 2016;11(2):e0149875. doi:10.1371/journal.pone.0149875

522. Hambrecht M, Lammertink M, Klosterkötter J, Matuschek E, Pukrop R. Subjective and objective neuropsychological abnormalities in a psychosis prodrome clinic. *British Journal of Psychiatry*. 2002;181(S43):s30-s37. doi:10.1192/bjp.181.43.s30

523. Hamilton HK, Woods SW, Roach BJ, et al. Auditory and Visual Oddball Stimulus Processing Deficits in Schizophrenia and the Psychosis Risk Syndrome: Forecasting Psychosis Risk With P300. *Schizophrenia Bulletin*. 2019;45(5):1068-1080. doi:10.1093/schbul/sby167

524. Hamilton HK, Roach BJ, Bachman PM, et al. Association Between P300 Responses to Auditory Oddball Stimuli and Clinical Outcomes in the Psychosis Risk Syndrome. *JAMA Psychiatry*. 2019;76(11):1187. doi:10.1001/jamapsychiatry.2019.2135

525. Hampton JN, Trotman HD, Addington J, et al. The relation of atypical antipsychotic use and stress with weight in individuals at clinical high risk for psychosis. *Stress and Health*. 2018;34(5):591-600. doi:10.1002/smi.2819

526. Han HJ, Jung WH, Jang JH, et al. Reduced volume in the anterior internal capsule but its maintained correlation with the frontal gray matter in subjects at ultra-high risk for psychosis. *Psychiatry Research: Neuroimaging*. 2012;204(2-3):82-90. doi:10.1016/j.pscychresns.2012.09.012

527. Hannan KL, Wood SJ, Yung AR, et al. Caudate nucleus volume in individuals at ultra-high risk of psychosis: A cross-sectional magnetic resonance imaging study. *Psychiatry Research: Neuroimaging*. 2010;182(3):223-230. doi:10.1016/j.pscychresns.2010.02.006

528. Harley M, Kelleher I, Clarke M, et al. Cannabis use and childhood trauma interact additively to increase the risk of psychotic symptoms in adolescence. *Psychological Medicine*. 2010;40(10):1627-1634. doi:10.1017/S0033291709991966

529. Harrisberger F, Smieskova R, Vogler C, et al. Impact of polygenic schizophrenia-related risk and hippocampal volumes on the onset of psychosis. *Translational Psychiatry*. 2016;6(8):e868-e868. doi:10.1038/tp.2016.143

530. Harrisberger F, Smieskova R, Egli T, et al. Impact on the Onset of Psychosis of a Polygenic Schizophrenia-Related Risk Score and Changes in White Matter Volume. *Cellular Physiology and Biochemistry*. 2018;48(3):1201-1214. doi:10.1159/000491986

531. Hartmann JA, McGorry PD, Schmidt SJ, et al. Opening the Black Box of Cognitive-Behavioural Case Management in Clients with Ultra-High Risk for Psychosis. *Psychotherapy and Psychosomatics*. 2017;86(5):292-299. doi:10.1159/000477551

532. Hartmann JA, Nelson B, Spooner R, et al. Broad clinical high‐risk mental state (CHARMS): Methodology of a cohort study validating criteria for pluripotent risk. *Early Intervention in Psychiatry*. 2019;13(3):379-386. doi:10.1111/eip.12483

533. Hartmann JA, Schmidt SJ, McGorry PD, et al. Trajectories of symptom severity and functioning over a three-year period in a psychosis high-risk sample: A secondary analysis of the Neurapro trial. *Behaviour Research and Therapy*. 2020;124:103527. doi:10.1016/j.brat.2019.103527

534. Hasan A, Wobrock T, Grefkes C, et al. Deficient Inhibitory Cortical Networks in Antipsychotic-Naive Subjects at Risk of Developing First-Episode Psychosis and First-Episode Schizophrenia Patients: A Cross-Sectional Study. *Biological Psychiatry*. 2012;72(9):744-751. doi:10.1016/j.biopsych.2012.03.005

535. Hauser M, Lautenschlager M, Gudlowski Y, et al. Psychoeducation with patients at-risk for schizophrenia—An exploratory pilot study. *Patient Education and Counseling*. 2009;76(1):138-142. doi:10.1016/j.pec.2008.11.003

536. Hauser M, Knoblich G, Repp BH, et al. Altered sense of agency in schizophrenia and the putative psychotic prodrome. *Psychiatry Research*. 2011;186(2-3):170-176. doi:10.1016/j.psychres.2010.08.003

537. Haut KM, van Erp TGM, Knowlton B, et al. Contributions of Feature Binding During Encoding and Functional Connectivity of the Medial Temporal Lobe Structures to Episodic Memory Deficits Across the Prodromal and First-Episode Phases of Schizophrenia. *Clinical Psychological Science*. 2015;3(2):159-174. doi:10.1177/2167702614533949

538. Hawkins KA, Addington J, Keefe RSE, et al. Neuropsychological status of subjects at high risk for a first episode of psychosis. *Schizophrenia Research*. 2004;67(2-3):115-122. doi:10.1016/j.schres.2003.08.007

539. Hawkins KA, Keefe RSE, Christensen BK, et al. Neuropsychological course in the prodrome and first episode of psychosis: Findings from the PRIME North America Double Blind Treatment Study. *Schizophrenia Research*. 2008;105(1-3):1-9. doi:10.1016/j.schres.2008.07.008

540. He Y, Kosciolek T, Tang J, et al. Gut microbiome and magnetic resonance spectroscopy study of subjects at ultra-high risk for psychosis may support the membrane hypothesis. *European Psychiatry*. 2018;53:37-45. doi:10.1016/j.eurpsy.2018.05.011

541. He Y, Li Z, Ma X, et al. Olfactory and cognitive functions in Chinese individuals at clinical high risk for psychosis. *Psychiatry Research*. 2019;272:51-53. doi:10.1016/j.psychres.2018.12.074

542. He Y, Yuan L, Li Z, et al. Plasma protein levels of brain-derived neurotrophic factor pathways and their association with cognitive performance in patients with clinical high risk for psychosis and first episode psychosis. *Schizophrenia Research*. 2019;206:460-461. doi:10.1016/j.schres.2018.11.016

543. Healey KM, Penn DL, Perkins D, Woods SW, Addington J. Theory of mind and social judgments in people at clinical high risk of psychosis. *Schizophrenia Research*. 2013;150(2-3):498-504. doi:10.1016/j.schres.2013.08.038

544. Healey KM, Penn DL, Perkins D, Woods SW, Keefe RSE, Addington J. Latent Profile Analysis and Conversion to Psychosis: Characterizing Subgroups to Enhance Risk Prediction. *Schizophrenia Bulletin*. 2018;44(2):286-296. doi:10.1093/schbul/sbx080

545. Hechtman L. ADHD medication treatment and risk of psychosis. *The Lancet Psychiatry*. 2019;6(8):632-633. doi:10.1016/S2215-0366(19)30248-2

546. Heinimaa M, Salokangas RKR, Ristkari T, et al. PROD-screen – a screen for prodromal symptoms of psychosis. *International Journal of Methods in Psychiatric Research*. 2003;12(2):92-104. doi:10.1002/mpr.146

547. Heinze K, Reniers RLEP, Nelson B, et al. Discrete Alterations of Brain Network Structural Covariance in Individuals at Ultra-High Risk for Psychosis. *Biological Psychiatry*. 2015;77(11):989-996. doi:10.1016/j.biopsych.2014.10.023

548. Heinze K, Lin A, Nelson B, et al. The impact of psychotic experiences in the early stages of mental health problems in young people. *BMC Psychiatry*. 2018;18(1):214. doi:10.1186/s12888-018-1767-y

549. Heitz U, Papmeyer M, Studerus E, et al. Plasma and serum brain-derived neurotrophic factor (BDNF) levels and their association with neurocognition in at-risk mental state, first episode psychosis and chronic schizophrenia patients. *The World Journal of Biological Psychiatry*. 2019;20(7):545-554. doi:10.1080/15622975.2018.1462532

550. Heitz U, Studerus E, Menghini‐Müller S, et al. Gender differences in first self‐perceived signs and symptoms in patients with an at‐risk mental state and first‐episode psychosis. *Early Intervention in Psychiatry*. 2019;13(3):582-588. doi:10.1111/eip.12528

551. Hengartner MP, Heekeren K, Dvorsky D, Walitza S, Rössler W, Theodoridou A. Course of psychotic symptoms, depression and global functioning in persons at clinical high risk of psychosis: Results of a longitudinal observation study over three years focusing on both converters and non-converters. *Schizophrenia Research*. 2017;189:19-26. doi:10.1016/j.schres.2017.01.040

552. Hermans K, van der Steen Y, Kasanova Z, et al. Temporal dynamics of suspiciousness and hallucinations in clinical high risk and first episode psychosis. *Psychiatry Research*. 2020;290:113039. doi:10.1016/j.psychres.2020.113039

553. Hickey T, Nelson B, Enticott J, Meadows G. The MAC-P program: A pilot study of a mindfulness and compassion program for youth with psychotic experiences. *Early Intervention in Psychiatry*. 2021;15(5):1326-1334. doi:10.1111/eip.13085

554. Hickie IB, Hermens DF, Naismith SL, et al. Evaluating differential developmental trajectories to adolescent-onset mood and psychotic disorders. *BMC Psychiatry*. 2013;13(1):303. doi:10.1186/1471-244X-13-303

555. Higuchi Y, Sumiyoshi T, Seo T, Miyanishi T, Kawasaki Y, Suzuki M. Mismatch Negativity and Cognitive Performance for the Prediction of Psychosis in Subjects with At-Risk Mental State. *PLoS ONE*. 2013;8(1):e54080. doi:10.1371/journal.pone.0054080

556. Higuchi Y, Seo T, Miyanishi T, Kawasaki Y, Suzuki M, Sumiyoshi T. Mismatch Negativity and P3a/Reorienting Complex in Subjects with Schizophrenia or At-Risk Mental State. *Frontiers in Behavioral Neuroscience*. 2014;8. doi:10.3389/fnbeh.2014.00172

557. Hlastala SA, McClellan J. Phenomenology and Diagnostic Stability of Youths with Atypical Psychotic Symptoms. *Journal of Child and Adolescent Psychopharmacology*. 2005;15(3):497-509. doi:10.1089/cap.2005.15.497

558. Ho BC. *MRI Brain Volume Abnormalities in Young, Nonpsychotic Relatives of Schizophrenia Probands Are Associated with Subsequent Prodromal Symptoms*.

559. Hodgekins J, French P, Birchwood M, et al. Comparing time use in individuals at different stages of psychosis and a non-clinical comparison group. *Schizophrenia Research*. 2015;161(2-3):188-193. doi:10.1016/j.schres.2014.12.011

560. Hoffman RE, Woods SW, Hawkins KA, et al. Extracting spurious messages from noise and risk of schizophrenia-spectrum disorders in a prodromal population. *British Journal of Psychiatry*. 2007;191(4):355-356. doi:10.1192/bjp.bp.106.031195

561. Hollis C, Groom MJ, Das D, et al. Different psychological effects of cannabis use in adolescents at genetic high risk for schizophrenia and with attention deficit/hyperactivity disorder (ADHD). *Schizophrenia Research*. 2008;105(1-3):216-223. doi:10.1016/j.schres.2008.07.010

562. Holtzman CW, Walker EF, Brennan P, et al. *Sex Differences in Stress Exposure and Reactivity in Individuals at Clinical High Risk for Psychosis*.; 2016.

563. Holzer L, Halfon O, Laget J. INTERVENTION IN ADOLESCENT PSYCHOSIS RISK. *Journal of the American Academy of Child & Adolescent Psychiatry*. 2005;44(6). doi:10.1097/01.chi.0000159167.74096.16

564. Holzer L, Urben S, Passini CM, et al. A Randomized Controlled Trial of the Effectiveness of Computer-Assisted Cognitive Remediation (CACR) in Adolescents with Psychosis or at High Risk of Psychosis. *Behavioural and Cognitive Psychotherapy*. 2014;42(4). doi:10.1017/S1352465813000313

565. Hong S bin, Lee TY, Kwak Y bin, Kim SN, Kwon JS. Baseline putamen volume as a predictor of positive symptom reduction in patients at clinical high risk for psychosis: A preliminary study. *Schizophrenia Research*. 2015;169(1-3):178-185. doi:10.1016/j.schres.2015.10.029

566. Hooker CI, Carol EE, Eisenstein TJ, et al. A pilot study of cognitive training in clinical high risk for psychosis: Initial evidence of cognitive benefit. *Schizophrenia Research*. 2014;157(1-3):314-316. doi:10.1016/j.schres.2014.05.034

567. Hopkinson G. The Prodromal Phase of the Depressive Psychosis. *European Neurology*. 1965;149(1). doi:10.1159/000128798

568. Hoptman M, Nierenberg J, Bertisch H, et al. A DTI study of white matter microstructure in individuals at high genetic risk for schizophrenia. *Schizophrenia Research*. 2008;106(2-3):115-124. doi:10.1016/j.schres.2008.07.023

569. Hou CL, Xiang YT, Wang ZL, et al. Cognitive functioning in individuals at ultra-high risk for psychosis, first-degree relatives of patients with psychosis and patients with first-episode schizophrenia. *Schizophrenia Research*. 2016;174(1-3):71-76. doi:10.1016/j.schres.2016.04.034

570. Howes OD, Montgomery AJ, Asselin MC, et al. *Elevated Striatal Dopamine Function Linked to Prodromal Signs of Schizophrenia*. Vol 66.; 2009.

571. Howes OD, Bose SK, Turkheimer F, et al. Dopamine Synthesis Capacity Before Onset of Psychosis: A Prospective [ ^18^ F]-DOPA PET Imaging Study. *American Journal of Psychiatry*. 2011;168(12):1311-1317. doi:10.1176/appi.ajp.2011.11010160

572. Howes OD, Bonoldi I, McCutcheon RA, et al. Glutamatergic and dopaminergic function and the relationship to outcome in people at clinical high risk of psychosis: a multi-modal PET-magnetic resonance brain imaging study. *Neuropsychopharmacology*. 2020;45(4):641-648. doi:10.1038/s41386-019-0541-2

573. Hsieh MH, Shan JC, Huang WL, et al. Auditory event-related potential of subjects with suspected pre-psychotic state and first‐episode psychosis. *Schizophrenia Research*. 2012;140(1-3):243-249. doi:10.1016/j.schres.2012.06.021

574. Hua JPY, Karcher NR, Merrill AM, et al. Psychosis risk is associated with decreased resting-state functional connectivity between the striatum and the default mode network. *Cognitive, Affective, & Behavioral Neuroscience*. 2019;19(4):998-1011. doi:10.3758/s13415-019-00698-z

575. Huang JTJ, Leweke FM, Tsang TM, et al. CSF Metabolic and Proteomic Profiles in Patients Prodromal for Psychosis. *PLoS ONE*. 2007;2(8). doi:10.1371/journal.pone.0000756

576. Huang ZH, Hou CL, Huang YH, et al. Individuals at high risk for psychosis experience more childhood trauma, life events and social support deficit in comparison to healthy controls. *Psychiatry Research*. 2019;273:296-302. doi:10.1016/j.psychres.2019.01.060

577. Huber CG, Smieskova R, Schroeder K, et al. Evidence for an agitated–aggressive syndrome predating the onset of psychosis. *Schizophrenia Research*. 2014;157(1-3):26-32. doi:10.1016/j.schres.2014.06.014

578. Huber CG, Widmayer S, Smieskova R, et al. Voxel-Based Morphometry Correlates of an Agitated-Aggressive Syndrome in the At-Risk Mental State for Psychosis and First Episode Psychosis. *Scientific Reports*. 2018;8(1):16516. doi:10.1038/s41598-018-33770-8

579. Hur JW, Shin NY, Jang JH, et al. Clinical and neurocognitive profiles of subjects at high risk for psychosis with and without obsessive–compulsive symptoms. *Australian & New Zealand Journal of Psychiatry*. 2012;46(2):161-169. doi:10.1177/0004867411432851

580. Hur JW, Byun MS, Shin NY, et al. General intellectual functioning as a buffer against theory-of-mind deficits in individuals at ultra-high risk for psychosis. *Schizophrenia Research*. 2013;149(1-3):83-87. doi:10.1016/j.schres.2013.06.019

581. Hur JW, Choi SH, Yun JY, Chon MW, Kwon JS. Parental socioeconomic status and prognosis in individuals with ultra-high risk for psychosis: A 2-year follow-up study. *Schizophrenia Research*. 2015;168(1-2):56-61. doi:10.1016/j.schres.2015.07.020

582. Hurlemann R, Boy C, Meyer PT, et al. Decreased prefrontal 5-HT2A receptor binding in subjects at enhanced risk for schizophrenia. *Anatomy and Embryology*. 2005;210(5-6). doi:10.1007/s00429-005-0036-2

583. Hurlemann R, Matusch A, Kuhn KU, et al. 5-HT2A receptor density is decreased in the at-risk mental state. *Psychopharmacology (Berl)*. 2008;195(4):579-590. doi:10.1007/s00213-007-0921-x

584. Hurlemann R, Jessen F, Wagner M, et al. Interrelated neuropsychological and anatomical evidence of hippocampal pathology in the at-risk mental state. *Psychological Medicine*. 2008;38(6):843-851. doi:10.1017/S0033291708003279

585. Hurtig TM, Taanila A, Veijola J, et al. Associations between psychotic-like symptoms and inattention/hyperactivity symptoms. *Social Psychiatry and Psychiatric Epidemiology*. 2011;46(1):17-27. doi:10.1007/s00127-009-0165-7

586. Hutton P, Bowe S, Parker S, Ford S. Prevalence of suicide risk factors in people at ultra-high risk of developing psychosis: a service audit. *Early Intervention in Psychiatry*. 2011;5(4):375-380. doi:10.1111/j.1751-7893.2011.00302.x

587. Hutton P, Parker S, Bowe S, Ford S. Prevalence of violence risk factors in people at ultra-high risk of developing psychosis: a service audit. *Early Intervention in Psychiatry*. 2012;6(1):91-96. doi:10.1111/j.1751-7893.2011.00307.x

588. Hafner H, Maurer K, Ruhrmann S, et al. Early detection and secondary prevention of psychosis: facts and visions*. *European Archives of Psychiatry and Clinical Neuroscience*. 2004;254(2):117-128. doi:10.1007/s00406-004-0508-z

589. Iorfino F, Scott EM, Carpenter JS, et al. Clinical Stage Transitions in Persons Aged 12 to 25 Years Presenting to Early Intervention Mental Health Services With Anxiety, Mood, and Psychotic Disorders. *JAMA Psychiatry*. 2019;76(11):1167. doi:10.1001/jamapsychiatry.2019.2360

590. Ising HK, Veling W, Loewy RL, et al. The Validity of the 16-Item Version of the Prodromal Questionnaire (PQ-16) to Screen for Ultra High Risk of Developing Psychosis in the General Help-Seeking Population. *Schizophrenia Bulletin*. 2012;38(6):1288-1296. doi:10.1093/schbul/sbs068

591. Ising HK, Smit F, Veling W, et al. Cost-effectiveness of preventing first-episode psychosis in ultra-high-risk subjects: multi-centre randomized controlled trial. *Psychological Medicine*. 2015;45(7):1435-1446. doi:10.1017/S0033291714002530

592. Ising HK, Kraan TC, Rietdijk J, et al. Four-Year Follow-up of Cognitive Behavioral Therapy in Persons at Ultra-High Risk for Developing Psychosis: The Dutch Early Detection Intervention Evaluation (EDIE-NL) Trial. *Schizophrenia Bulletin*. 2016;42(5):1243-1252. doi:10.1093/schbul/sbw018

593. Ising HK, Lokkerbol J, Rietdijk J, et al. Four-Year Cost-effectiveness of Cognitive Behavior Therapy for Preventing First-episode Psychosis: The Dutch Early Detection Intervention Evaluation (EDIE-NL) Trial. *Schizophrenia Bulletin*. Published online June 15, 2016:sbw084. doi:10.1093/schbul/sbw084

594. Ittig S, Studerus E, Papmeyer M, et al. Sex Differences in Cognitive Functioning in At-Risk Mental State for Psychosis, First Episode Psychosis and Healthy Control Subjects. *European Psychiatry*. 2015;30(2):242-250. doi:10.1016/j.eurpsy.2014.11.006

595. Ittig S, Studerus E, Heitz U, et al. Sex differences in prolactin levels in emerging psychosis: Indication for enhanced stress reactivity in women. *Schizophrenia Research*. 2017;189:111-116. doi:10.1016/j.schres.2017.02.010

596. Iwashiro N, Suga M, Takano Y, et al. Localized gray matter volume reductions in the pars triangularis of the inferior frontal gyrus in individuals at clinical high-risk for psychosis and first episode for schizophrenia. *Schizophrenia Research*. 2012;137(1-3):124-131. doi:10.1016/j.schres.2012.02.024

597. Iwashiro N, Koike S, Satomura Y, et al. Association between impaired brain activity and volume at the sub-region of Broca’s area in ultra-high risk and first-episode schizophrenia: A multi-modal neuroimaging study. *Schizophrenia Research*. 2016;172(1-3):9-15. doi:10.1016/j.schres.2016.02.005

598. Iyer S, Mustafa S, Gariépy G, et al. A NEET distinction: youths not in employment, education or training follow different pathways to illness and care in psychosis. *Social Psychiatry and Psychiatric Epidemiology*. 2018;53(12):1401-1411. doi:10.1007/s00127-018-1565-3

599. Izon E, Berry K, Wearden A, Carter LA, Law H, French P. Investigating expressed emotion in individuals at-risk of developing psychosis and their families over 12 months. *Clinical Psychology and Psychotherapy*. Published online 2021. doi:10.1002/cpp.2576

600. Jabben N, van Os J, Janssen I, Versmissen D, Krabbendam L. Cognitive alterations in groups at risk for psychosis: Neutral markers of genetic risk or indicators of social disability? *Acta Psychiatrica Scandinavica*. 2007;116(4):253-262. doi:10.1111/j.1600-0447.2006.00990.x

601. Jablensky A, Cole SW. Is the earlier age at onset of schizophrenia in males a confounded finding? *British Journal of Psychiatry*. 1997;170(3):234-240. doi:10.1192/bjp.170.3.234

602. Jacobson S, Kelleher I, Harley M, et al. Structural and functional brain correlates of subclinical psychotic symptoms in 11-13 year old schoolchildren. *Neuroimage*. 2010;49(2):1875-1885. doi:10.1016/j.neuroimage.2009.09.015

603. Jagannath V, Theodoridou A, Gerstenberg M, et al. Prediction Analysis for Transition to Schizophrenia in Individuals at Clinical High Risk for Psychosis: The Relationship of DAO, DAOA, and NRG1 Variants with Negative Symptoms and Cognitive Deficits. *Frontiers in Psychiatry*. 2017;8. doi:10.3389/fpsyt.2017.00292

604. Jagannath V, Gerstenberg M, Walitza S, et al. Neuregulin 1 (NRG1) gene expression predicts functional outcomes in individuals at clinical high-risk for psychosis. *Psychiatry Research*. 2018;266:143-146. doi:10.1016/j.psychres.2018.05.025

605. Jagannath V, Grünblatt E, Theodoridou A, et al. Rare copy number variants in individuals at clinical high risk for psychosis: Enrichment of synaptic/brain‐related functional pathways. *American Journal of Medical Genetics Part B: Neuropsychiatric Genetics*. 2020;183(2):140-151. doi:10.1002/ajmg.b.32770

606. Jahchan C. *Automatic Sensory Discrimination Impairment in Prodromal and Recent-Onset Schizophrenia*. 2010.

607. Jahshan C, Heaton RK, Golshan S, Cadenhead KS. Course of neurocognitive deficits in the prodrome and first episode of schizophrenia. *Neuropsychology*. 2010;24(1):109-120. doi:10.1037/a0016791

608. Jahshan C, Cadenhead KS, Rissling AJ, Kirihara K, Braff DL, Light GA. Automatic sensory information processing abnormalities across the illness course of schizophrenia. *Psychological Medicine*. 2012;42(1):85-97. doi:10.1017/S0033291711001061

609. Jalbrzikowski M, Carter C, Senturk D, et al. Social cognition in 22q11.2 microdeletion syndrome: Relevance to psychosis? *Schizophrenia Research*. 2012;142(1-3):99-107. doi:10.1016/j.schres.2012.10.007

610. Jalbrzikowski M, Krasileva KE, Marvin S, et al. Reciprocal social behavior in youths with psychotic illness and those at clinical high risk. *Development and Psychopathology*. 2013;25(4pt1):1187-1197. doi:10.1017/S095457941300045X

611. Jalbrzikowski M, Sugar CA, Zinberg J, Bachman P, Cannon TD, Bearden CE. Coping styles of individuals at clinical high risk for developing psychosis. *Early Intervention in Psychiatry*. 2014;8(1). doi:10.1111/eip.12005

612. Jalbrzikowski M, Villalon-Reina JE, Karlsgodt KH, et al. Altered white matter microstructure is associated with social cognition and psychotic symptoms in 22q11.2 microdeletion syndrome. *Frontiers in Behavioral Neuroscience*. 2014;8. doi:10.3389/fnbeh.2014.00393

613. Jang JH, Shin NY, Shim G, et al. Longitudinal Patterns of Social Functioning and Conversion to Psychosis in Subjects at Ultra-High Risk. *Australian & New Zealand Journal of Psychiatry*. 2011;45(9):763-770. doi:10.3109/00048674.2011.595684

614. Jarrett M, Craig T, Parrott J, et al. Identifying men at ultra high risk of psychosis in a prison population. *Schizophrenia Research*. 2012;136(1-3):1-6. doi:10.1016/j.schres.2012.01.025

615. Jarrett M, Jamieson-Craig TK, Forrester A, et al. Prison and Community Populations at Ultra-High Risk of Psychosis: Differences and Challenges for Service Provision. *Psychiatric Services*. 2016;67(9):990-995. doi:10.1176/appi.ps.201500355

616. Jarrett M, Valmaggia L, Parrott J, et al. Prisoners at ultra-high-risk for psychosis: a cross-sectional study. *Epidemiology and Psychiatric Sciences*. 2016;25(2):150-159. doi:10.1017/S2045796015000062

617. Jeffries CD, Perkins DO, Chandler SD, et al. Insights into psychosis risk from leukocyte microRNA expression. *Translational Psychiatry*. 2016;6(12):e981-e981. doi:10.1038/tp.2016.148

618. Jessen F, Scherk H, Träber F, et al. Proton magnetic resonance spectroscopy in subjects at risk for schizophrenia. *Schizophrenia Research*. 2006;87(1-3):81-88. doi:10.1016/j.schres.2006.06.011

619. Jhung K, Cho SH, Jang JH, et al. Small-world networks in individuals at ultra-high risk for psychosis and first-episode schizophrenia during a working memory task. *Neuroscience Letters*. 2013;535(1):35-39. doi:10.1016/j.neulet.2012.11.051

620. Jia H, Yang J, Zhu H, Liu J, Barnaby N. Self-face recognition in the ultra-high risk for psychosis population. *Early Intervention in Psychiatry*. 2015;9(2):126-132. doi:10.1111/eip.12097

621. Jimeno N, Gomez-Pilar J, Poza J, et al. Main symptomatic treatment targets in suspected and early psychosis: New insights from network analysis. *Schizophrenia Bulletin*. 2020;46(4):884-895. doi:10.1093/schbul/sbz140

622. Jin H, Tappenden P, MacCabe JH, Robinson S, Byford S. Evaluation of the Cost-effectiveness of Services for Schizophrenia in the UK Across the Entire Care Pathway in a Single Whole-Disease Model. *JAMA Network Open*. 2020;3(5):e205888. doi:10.1001/jamanetworkopen.2020.5888

623. Johns LC, Allen P, Valli I, et al. Impaired verbal self-monitoring in individuals at high risk of psychosis. *Psychological Medicine*. 2010;40(9):1433-1442. doi:10.1017/S0033291709991991

624. Jongeneel A, Pot-Kolder R, Counotte J, van der Gaag M, Veling W. Self-esteem moderates affective and psychotic responses to social stress in psychosis: A virtual reality study. *Schizophrenia Research*. 2018;202:80-85. doi:10.1016/j.schres.2018.06.042

625. Juckel G, Friedel E, Koslowski M, et al. Ventral Striatal Activation during Reward Processing in Subjects with Ultra-High Risk for Schizophrenia. *Neuropsychobiology*. 2012;66(1):50-56. doi:10.1159/000337130

626. Jukuri T, Kiviniemi V, Nikkinen J, et al. Default mode network in young people with familial risk for psychosis — The Oulu Brain and Mind Study. *Schizophrenia Research*. 2013;143(2-3):239-245. doi:10.1016/j.schres.2012.11.020

627. Yeon Jung H, Seung Chang J, Seo Yi J, et al. Measuring psychosis proneness in a nonclinical Korean population: is the Peters et al Delusions Inventory useful for assessing high-risk individuals? *Comprehensive Psychiatry*. 2008;49(2):202-210. doi:10.1016/j.comppsych.2007.08.011

628. Jung WH, Jang JH, Shin NY, et al. Regional Brain Atrophy and Functional Disconnection in Broca’s Area in Individuals at Ultra-High Risk for Psychosis and Schizophrenia. *PLoS ONE*. 2012;7(12). doi:10.1371/journal.pone.0051975

629. Jutla A, Califano A, Dishy G, et al. Neurodevelopmental predictors of conversion to schizophrenia and other psychotic disorders in adolescence and young adulthood in clinical high risk individuals. *Schizophrenia Research*. 2020;224:170-172. doi:10.1016/j.schres.2020.10.008

630. Kafadar E, Mittal VA, Strauss GP, et al. Modeling perception and behavior in individuals at clinical high risk for psychosis: Support for the predictive processing framework. *Schizophrenia Research*. 2020;226:167-175. doi:10.1016/j.schres.2020.04.017

631. Kafali HY, Bildik T, Bora E, Yuncu Z, Erermis HS. Distinguishing prodromal stage of bipolar disorder and early onset schizophrenia spectrum disorders during adolescence. *Psychiatry Research*. 2019;275:315-325. doi:10.1016/j.psychres.2019.03.051

632. Kalin NH. Psychotic Experiences, Cognitive Decline, and Genetic Vulnerabilities in Relation to Developing Psychotic Disorders. *American Journal of Psychiatry*. 2020;177(4):279-281. doi:10.1176/appi.ajp.2020.20020162

633. Kalin NH. Treating Substance Use Disorders, Binge Eating, and Depression, and Identifying Factors Underlying Psychosis Risk. *American Journal of Psychiatry*. 2020;177(2):101-103. doi:10.1176/appi.ajp.2019.19121275

634. Kamath V, Moberg PJ, Calkins ME, et al. An odor-specific threshold deficit implicates abnormal cAMP signaling in youths at clinical risk for psychosis. *Schizophrenia Research*. 2012;138(2-3):280-284. doi:10.1016/j.schres.2012.03.029

635. Kamath V, Turetsky BI, Calkins ME, et al. Olfactory processing in schizophrenia, non-ill first-degree family members, and young people at-risk for psychosis. *The World Journal of Biological Psychiatry*. 2014;15(3). doi:10.3109/15622975.2011.615862

636. Kang NI, Park TW, Yang JC, Oh KY, Shim SH, Chung YC. Prevalence and clinical features of Thought–Perception–Sensitivity Symptoms: Results from a community survey of Korean high school students. *Psychiatry Research*. 2012;198(3):501-508. doi:10.1016/j.psychres.2012.03.005

637. Kang JI, Park HJ, Kim SJ, et al. Reduced Binding Potential of GABA-A/Benzodiazepine Receptors in Individuals at Ultra-high Risk for Psychosis: An [18F]-Fluoroflumazenil Positron Emission Tomography Study. *Schizophrenia Bulletin*. 2014;40(3):548-557. doi:10.1093/schbul/sbt052

638. Karanikas E, Griveas I, Ntouros E, Floros G, Garyfallos G. Evidence for increased immune mobilization in First Episode Psychosis compared with the prodromal stage in males. *Psychiatry Research*. 2016;244:333-338. doi:10.1016/j.psychres.2016.07.059

639. Karanikas E, Manganaris S, Ntouros E, Floros G, Antoniadis D, Garyfallos G. Cytokines, cortisol and IGF-1 in first episode psychosis and ultra high risk males. Evidence for TNF-α, IFN-γ, ΤNF-β, IL-4 deviation. *Asian Journal of Psychiatry*. 2017;26:99-103. doi:10.1016/j.ajp.2017.01.026

640. Karcher NR, Hua JPY, Kerns JG. Striatum-related functional activation during reward- versus punishment-based learning in psychosis risk. *Neuropsychopharmacology*. 2019;44(11):1967-1974. doi:10.1038/s41386-019-0455-z

641. Karcher NR, Hua JPY, Kerns JG. Probabilistic Category Learning and Striatal Functional Activation in Psychosis Risk. *Schizophrenia Bulletin*. 2019;45(2):396-404. doi:10.1093/schbul/sby033

642. Karlsgodt KH. Using Advanced Diffusion Metrics to Probe White Matter Microstructure in Individuals at Clinical High Risk for Psychosis. *American Journal of Psychiatry*. 2019;176(10):777-779. doi:10.1176/appi.ajp.2019.19080808

643. Katagiri N, Pantelis C, Nemoto T, et al. A longitudinal study investigating sub-threshold symptoms and white matter changes in individuals with an ‘at risk mental state’ (ARMS). *Schizophrenia Research*. 2015;162(1-3):7-13. doi:10.1016/j.schres.2015.01.002

644. Katagiri N, Pantelis C, Nemoto T, et al. Symptom recovery and relationship to structure of corpus callosum in individuals with an ‘at risk mental state.’ *Psychiatry Research: Neuroimaging*. 2018;272:1-6. doi:10.1016/j.pscychresns.2017.11.016

645. Katagiri N, Pantelis C, Nemoto T, et al. Longitudinal changes in striatum and sub-threshold positive symptoms in individuals with an ‘at risk mental state’ (ARMS). *Psychiatry Research: Neuroimaging*. 2019;285:25-30. doi:10.1016/j.pscychresns.2019.01.008

646. Kates WR, Antshel KM, Faraone S v., et al. Neuroanatomic Predictors to Prodromal Psychosis in Velocardiofacial Syndrome (22q11.2 Deletion Syndrome): A Longitudinal Study. *Biological Psychiatry*. 2011;69(10):945-952. doi:10.1016/j.biopsych.2010.10.027

647. Katsura M, Ohmuro N, Obara C, et al. A naturalistic longitudinal study of at-risk mental state with a 2.4year follow-up at a specialized clinic setting in Japan. *Schizophrenia Research*. 2014;158(1-3):32-38. doi:10.1016/j.schres.2014.06.013

648. Kayser J, Tenke CE, Kroppmann CJ, et al. Olfaction in the psychosis prodrome: Electrophysiological and behavioral measures of odor detection. *International Journal of Psychophysiology*. 2013;90(2):190-206. doi:10.1016/j.ijpsycho.2013.07.003

649. Kayser J, Tenke CE, Kroppmann CJ, et al. Auditory event-related potentials and alpha oscillations in the psychosis prodrome: Neuronal generator patterns during a novelty oddball task. *International Journal of Psychophysiology*. 2014;91(2):104-120. doi:10.1016/j.ijpsycho.2013.12.003

650. Kebir O, Chaumette B, Krebs MO. Epigenetic variability in conversion to psychosis: novel findings from an innovative longitudinal methylomic analysis. *Translational Psychiatry*. 2018;8(1):93. doi:10.1038/s41398-018-0138-2

651. Keefe RSE, Woods SW, Cannon TD, et al. A randomized Phase II trial evaluating efficacy, safety, and tolerability of oral BI 409306 in attenuated psychosis syndrome: Design and rationale. *Early Intervention in Psychiatry*. 2021;15(5):1315-1325. doi:10.1111/eip.13083

652. Keefe RSE, Perkins DO, Gu H, Zipursky RB, Christensen BK, Lieberman JA. A longitudinal study of neurocognitive function in individuals at-risk for psychosis. *Schizophrenia Research*. 2006;88(1-3):26-35. doi:10.1016/j.schres.2006.06.041

653. Kegeles LS, Ciarleglio A, León-Ortiz P, et al. An imaging-based risk calculator for prediction of conversion to psychosis in clinical high-risk individuals using glutamate 1H MRS. *Schizophrenia Research*. 2020;226:70-73. doi:10.1016/j.schres.2019.09.004

654. Kelleher I, Murtagh A, Molloy C, et al. Identification and Characterization of Prodromal Risk Syndromes in Young Adolescents in the Community: A Population-Based Clinical Interview Study. *Schizophrenia Bulletin*. 2012;38(2):239-246. doi:10.1093/schbul/sbr164

655. Kelleher I, Murtagh A, Clarke MC, Murphy J, Rawdon C, Cannon M. Neurocognitive performance of a community-based sample of young people at putative ultra high risk for psychosis: Support for the processing speed hypothesis. *Cognitive Neuropsychiatry*. 2013;18(1-2):9-25. doi:10.1080/13546805.2012.682363

656. Kelleher I, Devlin N, Wigman JTW, et al. Psychotic experiences in a mental health clinic sample: implications for suicidality, multimorbidity and functioning. *Psychological Medicine*. 2014;44(8). doi:10.1017/S0033291713002122

657. Kéri S, Benedek G. *Visual Contrast Sensitivity Alterations in Inferred Magnocellular Pathways and Anomalous Perceptual Experiences in People at High-Risk for Psychosis*.; 2007.

658. Kéri S. Psychosis prevention and early intervention in Hungary. *Clinical Neuropsychiatry: Journal of Treatment Evaluation*. 2008;5(6):295-302.

659. Kéri S, Kiss I, Kelemen O. Effects of a neuregulin 1 variant on conversion to schizophrenia and schizophreniform disorder in people at high risk for psychosis. *Molecular Psychiatry*. 2009;14(2):118-119. doi:10.1038/mp.2008.1

660. Kerns JG, Berenbaum H. Aberrant semantic and affective processing in people at risk for psychosis. *Journal of Abnormal Psychology*. 2000;109(4):728-732. doi:10.1037/0021-843X.109.4.728

661. Keshavan MS, Vora A, Montrose D, Diwadkar VA, Sweeney J. Olfactory identification in young relatives at risk for schizophrenia. *Acta Neuropsychiatrica*. 2009;21(3):121-124. doi:10.1111/j.1601-5215.2009.00390.x

662. Keshavan MS, Eack SM, Montrose DM, et al. Do premorbid impairments predict emergent “prodromal” symptoms in young relatives at risk for schizophrenia? *Early Intervention in Psychiatry*. 2009;3(3):213-220. doi:10.1111/j.1751-7893.2009.00135.x

663. Kim SW, Schäfer MR, Klier CM, et al. Relationship between membrane fatty acids and cognitive symptoms and information processing in individuals at ultra-high risk for psychosis. *Schizophrenia Research*. 2014;158(1-3):39-44. doi:10.1016/j.schres.2014.06.032

664. Kim HS, Shin NY, Choi JS, et al. Processing of facial configuration in individuals at ultra-high risk for schizophrenia. *Schizophrenia Research*. 2010;118(1-3):81-87. doi:10.1016/j.schres.2010.01.003

665. Kim HS, Shin NY, Jang JH, et al. Social cognition and neurocognition as predictors of conversion to psychosis in individuals at ultra-high risk. *Schizophrenia Research*. 2011;130(1-3):170-175. doi:10.1016/j.schres.2011.04.023

666. Kim KR, Lee SY, Kang JI, et al. Clinical efficacy of individual cognitive therapy in reducing psychiatric symptoms in people at ultra-high risk for psychosis. *Early Intervention in Psychiatry*. 2011;5(2):174-178. doi:10.1111/j.1751-7893.2011.00267.x

667. Kim SN, Park JS, Jang JH, et al. Increased white matter integrity in the corpus callosum in subjects with high genetic loading for schizophrenia. *Progress in Neuro-Psychopharmacology and Biological Psychiatry*. 2012;37(1):50-55. doi:10.1016/j.pnpbp.2011.11.015

668. Kim M, Lee TY, Lee S, Kim SN, Kwon JS. Auditory P300 as a predictor of short-term prognosis in subjects at clinical high risk for psychosis. *Schizophrenia Research*. 2015;165(2-3):138-144. doi:10.1016/j.schres.2015.04.033

669. Kim SW, Jhon M, Kim JM, et al. Relationship between Erythrocyte Fatty Acid Composition and Psychopathology in the Vienna Omega-3 Study. *PLOS ONE*. 2016;11(3):e0151417. doi:10.1371/journal.pone.0151417

670. Kim M, Cho KIK, Yoon YB, Lee TY, Kwon JS. Aberrant temporal behavior of mismatch negativity generators in schizophrenia patients and subjects at clinical high risk for psychosis. *Clinical Neurophysiology*. 2017;128(2):331-339. doi:10.1016/j.clinph.2016.11.027

671. Kim M, Lee TH, Kim JH, et al. Decomposing P300 into correlates of genetic risk and current symptoms in schizophrenia: An inter-trial variability analysis. *Schizophrenia Research*. 2018;192:232-239. doi:10.1016/j.schres.2017.04.001

672. Kim M, Lee TH, Yoon YB, Lee TY, Kwon JS. Predicting Remission in Subjects at Clinical High Risk for Psychosis Using Mismatch Negativity. *Schizophrenia Bulletin*. 2018;44(3):575-583. doi:10.1093/schbul/sbx102

673. Kimhy D, Jobson-Ahmed L, Ben-David S, Ramadhar L, Malaspina D, Corcoran CM. Cognitive insight in individuals at clinical high risk for psychosis. *Early Intervention in Psychiatry*. 2014;8(2). doi:10.1111/eip.12023

674. Kimhy D, Corcoran C, Harkavy-Friedman JM, Ritzler B, Javitt DC, Malaspina D. Visual form perception: A comparison of individuals at high risk for psychosis, recent onset schizophrenia and chronic schizophrenia. *Schizophrenia Research*. 2007;97(1-3):25-34. doi:10.1016/j.schres.2007.08.022

675. Kimhy D, Gill KE, Brucato G, et al. The impact of emotion awareness and regulation on social functioning in individuals at clinical high risk for psychosis. *Psychological Medicine*. 2016;46(14):2907-2918. doi:10.1017/S0033291716000490

676. Kindler J, Michel C, Schultze-Lutter F, et al. Functional and structural correlates of abnormal involuntary movements in psychosis risk and first episode psychosis. *Schizophrenia Research*. 2019;212:196-203. doi:10.1016/j.schres.2019.07.032

677. Kirkbride JB, Stochl J, Zimbrón J, et al. Social and spatial heterogeneity in psychosis proneness in a multilevel case-prodrome-control study. *Acta Psychiatrica Scandinavica*. 2015;132(4):283-292. doi:10.1111/acps.12384

678. Kiss I, Kelemen O, Kéri S. Decreased peripheral expression of neuregulin 1 in high-risk individuals who later converted to psychosis. *Schizophrenia Research*. 2012;135(1-3):198-199. doi:10.1016/j.schres.2011.12.012

679. Klaassen RMC, Velthorst E, Nieman DH, et al. Factor Analysis of the Scale of Prodromal Symptoms: Differentiating between Negative and Depression Symptoms. *Psychopathology*. 2011;44(6):379-385. doi:10.1159/000325169

680. Kleineidam L, Frommann I, Ruhrmann S, et al. Antisaccade and prosaccade eye movements in individuals clinically at risk for psychosis: comparison with first-episode schizophrenia and prediction of conversion. *European Archives of Psychiatry and Clinical Neuroscience*. 2019;269(8):921-930. doi:10.1007/s00406-018-0973-4

681. Kline E, Wilson C, Ereshefsky S, et al. Psychosis risk screening in youth: A validation study of three self-report measures of attenuated psychosis symptoms. *Schizophrenia Research*. 2012;141(1):72-77. doi:10.1016/j.schres.2012.07.022

682. Kline E, Thompson E, Schimunek C, et al. Parent–adolescent agreement on psychosis risk symptoms. *Schizophrenia Research*. 2013;147(1):147-152. doi:10.1016/j.schres.2013.03.007

683. Kline E, Thompson E, Bussell K, Pitts SC, Reeves G, Schiffman J. Psychosis-like experiences and distress among adolescents using mental health services. *Schizophrenia Research*. 2014;152(2-3):498-502. doi:10.1016/j.schres.2013.12.012

684. Kline E, Thompson E, Demro C, Bussell K, Reeves G, Schiffman J. Longitudinal validation of psychosis risk screening tools. *Schizophrenia Research*. 2015;165(2-3):116-122. doi:10.1016/j.schres.2015.04.026

685. Kline E, Millman ZB, Denenny D, et al. Trauma and psychosis symptoms in a sample of help-seeking youth. *Schizophrenia Research*. 2016;175(1-3):174-179. doi:10.1016/j.schres.2016.04.006

686. Klippel A, Myin-Germeys I, Chavez-Baldini U, et al. Modeling the Interplay Between Psychological Processes and Adverse, Stressful Contexts and Experiences in Pathways to Psychosis: An Experience Sampling Study. *Schizophrenia Bulletin*. 2017;43(2):302-315. doi:10.1093/schbul/sbw185

687. Klosterkötter J, Ebel H, Schultze-Lutter F, Steinmeyer EM. Diagnostic validity of basic symptoms. *European Archives of Psychiatry and Clinical Neuroscience*. 1996;246(3):147-154. doi:10.1007/BF02189116

688. Klosterkötter J, Hellmich M, Schultze-Lutter F. [Is the diagnosis of schizophrenic illness possible in the initial prodromal phase to the first psychotic manifestation?]. *Fortschr Neurol Psychiatr*. 2000;68 Suppl 1:S13-21.

689. Kobayashi H, Nemoto T, Koshikawa H, et al. A self-reported instrument for prodromal symptoms of psychosis: Testing the clinical validity of the PRIME Screen-Revised (PS-R) in a Japanese population. *Schizophrenia Research*. 2008;106(2-3):356-362. doi:10.1016/j.schres.2008.08.018

690. Kobayashi H, Morita K, Takeshi K, et al. Effects of aripiprazole on insight and subjective experience in individuals with an at-risk mental state. *Journal of Clinical Psychopharmacology*. 2009;29(5):421-425. doi:10.1097/JCP.0b013e3181b2fe22

691. Koethe D, Gerth CW, Neatby MA, et al. Disturbances of visual information processing in early states of psychosis and experimental delta-9-tetrahydrocannabinol altered states of consciousness. *Schizophrenia Research*. 2006;88(1-3):142-150. doi:10.1016/j.schres.2006.07.023

692. Koethe D, Giuffrida A, Schreiber D, et al. Anandamide elevation in cerebrospinal fluid in initial prodromal states of psychosis. *British Journal of Psychiatry*. 2009;194(4):371-372. doi:10.1192/bjp.bp.108.053843

693. Koethe D, Kranaster L, Hoyer C, et al. Binocular depth inversion as a paradigm of reduced visual information processing in prodromal state, antipsychotic-naïve and treated schizophrenia. *European Archives of Psychiatry and Clinical Neuroscience*. 2009;259(4):195-202. doi:10.1007/s00406-008-0851-6

694. Koh Y, Shin KS, Kim JS, et al. An MEG study of alpha modulation in patients with schizophrenia and in subjects at high risk of developing psychosis. *Schizophrenia Research*. 2011;126(1-3):36-42. doi:10.1016/j.schres.2010.10.001

695. Köhler S, van Os J, Graaf R, Vollebergh W, Verhey F, Krabbendam L. Psychosis risk as a function of age at onset. *Social Psychiatry and Psychiatric Epidemiology*. 2007;42(4):288-294. doi:10.1007/s00127-007-0171-6

696. Kohler CG, Richard JA, Brensinger CM, et al. Facial emotion perception differs in young persons at genetic and clinical high-risk for psychosis. *Psychiatry Research*. 2014;216(2):206-212. doi:10.1016/j.psychres.2014.01.023

697. Koike S, Takizawa R, Nishimura Y, et al. Different hemodynamic response patterns in the prefrontal cortical sub-regions according to the clinical stages of psychosis. *Schizophrenia Research*. 2011;132(1):54-61. doi:10.1016/j.schres.2011.07.014

698. Koike S, Takano Y, Iwashiro N, et al. A multimodal approach to investigate biomarkers for psychosis in a clinical setting: The integrative neuroimaging studies in schizophrenia targeting for early intervention and prevention (IN-STEP) project. *Schizophrenia Research*. 2013;143(1):116-124. doi:10.1016/j.schres.2012.11.012

699. Koike S, Satomura Y, Kawasaki S, et al. Application of functional near infrared spectroscopy as supplementary examination for diagnosis of clinical stages of psychosis spectrum. *Psychiatry and Clinical Neurosciences*. 2017;71(12):794-806. doi:10.1111/pcn.12551

700. Koivukangas J, Tammelin T, Kaakinen M, et al. Physical activity and fitness in adolescents at risk for psychosis within the Northern Finland 1986 Birth Cohort. *Schizophrenia Research*. 2010;116(2-3):152-158. doi:10.1016/j.schres.2009.10.022

701. Kollias K, Xenaki LA, Vlachos I, et al. The development of the Early Intervention in Psychosis (EIP) outpatient unit of Eginition University Hospital into an EIP Network. *Psychiatriki*. 2020;31(2):177-182. doi:10.22365/jpsych.2020.312.177

702. Kommescher M, Wagner M, Pützfeld V, et al. Coping as a predictor of treatment outcome in people at clinical high risk of psychosis. *Early Intervention in Psychiatry*. 2016;10(1):17-27. doi:10.1111/eip.12130

703. Kommescher M, Gross S, Pützfeld V, Klosterkötter J, Bechdolf A. Coping and the stages of psychosis: an investigation into the coping styles in people at risk of psychosis, in people with first-episode and multiple-episode psychoses. *Early Intervention in Psychiatry*. 2017;11(2):147-155. doi:10.1111/eip.12223

704. Kong L, Cui H, Zhang T, et al. Neurological soft signs and grey matter abnormalities in individuals with ultra‐high risk for psychosis. *PsyCh Journal*. 2019;8(2):252-260. doi:10.1002/pchj.258

705. Konings M, Stefanis N, Kuepper R, et al. Replication in two independent population-based samples that childhood maltreatment and cannabis use synergistically impact on psychosis risk. *Psychological Medicine*. 2012;42(1):149-159. doi:10.1017/S0033291711000973

706. Konishi J, del Re EC, Bouix S, et al. Abnormal relationships between local and global brain measures in subjects at clinical high risk for psychosis: a pilot study. *Brain Imaging and Behavior*. 2018;12(4):974-988. doi:10.1007/s11682-017-9758-z

707. Koponen H, Vuononvirta J, Mäki P, et al. No difference in insulin resistance and lipid levels between controls and adolescent subjects who later develop psychosis. *Schizophrenia Research*. 2008;104(1-3):31-35. doi:10.1016/j.schres.2008.05.021

708. Koponen H, Mäki P, Halonen H, et al. Insulin resistance and lipid levels in adolescents with familial risk for psychosis. *Acta Psychiatrica Scandinavica*. 2008;117(5):337-341. doi:10.1111/j.1600-0447.2008.01154.x

709. Korkeila JA, Svirskis T, Heinimaa M, et al. Substance abuse and related diagnoses in early psychosis. *Comprehensive Psychiatry*. 2005;46(6):447-452. doi:10.1016/j.comppsych.2005.03.008

710. Korkeila JA, Svirkis T, Heinimaa M, et al. Physical ill health and risk of psychosis. *Psychiatry Research*. 2007;150(3):255-263. doi:10.1016/j.psychres.2006.03.016

711. Korver N, Nieman DH, Becker HE, et al. Symptomatology and Neuropsychological Functioning in Cannabis Using Subjects at Ultra-High Risk for Developing Psychosis and Healthy Controls. *Australian & New Zealand Journal of Psychiatry*. 2010;44(3):230-236. doi:10.3109/00048670903487118

712. Koshiyama D, Kirihara K, Tada M, et al. Duration and frequency mismatch negativity shows no progressive reduction in early stages of psychosis. *Schizophrenia Research*. 2017;190:32-38. doi:10.1016/j.schres.2017.03.015

713. Koshiyama D, Kirihara K, Tada M, et al. Electrophysiological evidence for abnormal glutamate-GABA association following psychosis onset. *Translational Psychiatry*. 2018;8(1):211. doi:10.1038/s41398-018-0261-0

714. Koshiyama D, Kirihara K, Tada M, et al. Association between mismatch negativity and global functioning is specific to duration deviance in early stages of psychosis. *Schizophrenia Research*. 2018;195:378-384. doi:10.1016/j.schres.2017.09.045

715. Koshiyama D, Kirihara K, Tada M, et al. Auditory gamma oscillations predict global symptomatic outcome in the early stages of psychosis: A longitudinal investigation. *Clinical Neurophysiology*. 2018;129(11):2268-2275. doi:10.1016/j.clinph.2018.08.007

716. Köther U, Lincoln TM, Moritz S. Emotion perception and overconfidence in errors under stress in psychosis. *Psychiatry Research*. 2018;270:981-991. doi:10.1016/j.psychres.2018.03.044

717. Kotlicka-Antczak M, Pawełczyk T, Rabe-Jabłońska J, Pawełczyk A. PORT (Programme of Recognition and Therapy): the first Polish recognition and treatment programme for patients with an at-risk mental state. *Early Intervention in Psychiatry*. 2015;9(4):339-342. doi:10.1111/eip.12146

718. Kotlicka-Antczak M, Pawełczyk A, Pawełczyk T, Strzelecki D, Żurner N, Karbownik MS. A history of obstetric complications is associated with the risk of progression from an at risk mental state to psychosis. *Schizophrenia Research*. 2018;197:498-503. doi:10.1016/j.schres.2017.10.039

719. Kotlicka-Antczak M, Karbownik MS, Pawełczyk A, et al. A developmentally-stable pattern of premorbid schizoid-schizotypal features predicts psychotic transition from the clinical high-risk for psychosis state. *Comprehensive Psychiatry*. 2019;90:95-101. doi:10.1016/j.comppsych.2019.02.003

720. Koutsouleris N, Schmitt GJE, Gaser C, et al. Neuroanatomical correlates of different vulnerability states for psychosis and their clinical outcomes. *British Journal of Psychiatry*. 2009;195(3):218-226. doi:10.1192/bjp.bp.108.052068

721. Koutsouleris N, Meisenzahl EM, Davatzikos C, et al. *Use of Neuroanatomical Pattern Classification to Identify Subjects in At-Risk Mental States of Psychosis and Predict Disease Transition*. Vol 66.; 2009.

722. Koutsouleris N, Patschurek-Kliche K, Scheuerecker J, et al. Neuroanatomical correlates of executive dysfunction in the at-risk mental state for psychosis. *Schizophrenia Research*. 2010;123(2-3):160-174. doi:10.1016/j.schres.2010.08.026

723. Koutsouleris N, Borgwardt S, Meisenzahl EM, Bottlender R, Möller HJ, Riecher-Rössler A. Disease Prediction in the At-Risk Mental State for Psychosis Using Neuroanatomical Biomarkers: Results From the FePsy Study. *Schizophrenia Bulletin*. 2012;38(6):1234-1246. doi:10.1093/schbul/sbr145

724. Koutsouleris N, Riecher-Rössler A, Meisenzahl EM, et al. Detecting the Psychosis Prodrome Across High-Risk Populations Using Neuroanatomical Biomarkers. *Schizophrenia Bulletin*. 2015;41(2):471-482. doi:10.1093/schbul/sbu078

725. Koutsouleris N, Upthegrove R, Wood SJ. Importance of Variable Selection in Multimodal Prediction Models in Patients at Clinical High Risk for Psychosis and Recent Onset Depression—Reply. *JAMA Psychiatry*. 2019;76(3):339. doi:10.1001/jamapsychiatry.2018.4237

726. Kraan T, van Dam DS, Velthorst E, et al. Childhood trauma and clinical outcome in patients at ultra-high risk of transition to psychosis. *Schizophrenia Research*. 2015;169(1-3):193-198. doi:10.1016/j.schres.2015.10.030

727. Kraan TC, Ising HK, Fokkema M, et al. The effect of childhood adversity on 4-year outcome in individuals at ultra high risk for psychosis in the Dutch Early Detection Intervention Evaluation (EDIE-NL) Trial. *Psychiatry Research*. 2017;247:55-62. doi:10.1016/j.psychres.2016.11.014

728. Krakauer K, Ebdrup BH, Glenthøj BY, et al. Patterns of white matter microstructure in individuals at ultra-high-risk for psychosis: associations to level of functioning and clinical symptoms. *Psychological Medicine*. 2017;47(15):2689-2707. doi:10.1017/S0033291717001210

729. Krakauer K, Nordentoft M, Glenthøj BY, et al. White matter maturation during 12 months in individuals at ultra-high-risk for psychosis. *Acta Psychiatrica Scandinavica*. 2018;137(1):65-78. doi:10.1111/acps.12835

730. Kristensen K, Cadenhead KS. Cannabis abuse and risk for psychosis in a prodromal sample. *Psychiatry Research*. 2007;151(1-2):151-154.

731. Krkovic K, Moritz S, Lincoln TM. Neurocognitive deficits or stress overload: Why do individuals with schizophrenia show poor performance in neurocognitive tests? *Schizophrenia Research*. 2017;183:151-156. doi:10.1016/j.schres.2016.11.002

732. Kuharic DB, Kekin I, Hew J, Kuzman MR, Puljak L. Preventive treatments in patients at high risk of psychosis. *The Lancet Psychiatry*. 2020;7(5):384-385. doi:10.1016/S2215-0366(20)30100-0

733. Kunwar A, Ramanathan S, Nelson J, et al. Cortical gyrification in velo-cardio-facial (22q11.2 deletion) syndrome: A longitudinal study. *Schizophrenia Research*. 2012;137(1-3):20-25. doi:10.1016/j.schres.2012.01.032

734. Kwapil TR, Chapman JP, Chapman LJ, Miller MB. Deviant Olfactory Experiences as Indicators of Risk for Psychosis. *Schizophrenia Bulletin*. 1996;22(2):371-380. doi:10.1093/schbul/22.2.371

735. Kwapil TR, Raulin ML, Midthun JC. A Ten-Year Longitudinal Study of Intense Ambivalence as a Predictor of Risk for Psychopathology. *The Journal of Nervous and Mental Disease*. 2000;188(7):402-408. doi:10.1097/00005053-200007000-00002

736. Labad J, Armario A, Nadal R, et al. Clinical correlates of hypothalamic-pituitary-adrenal axis measures in individuals at risk for psychosis and with first-episode psychosis. *Psychiatry Research*. 2018;265:284-291. doi:10.1016/j.psychres.2018.05.018

737. Lagopoulos J, Hermens DF, Hatton SN, et al. Microstructural white matter changes are correlated with the stage of psychiatric illness. *Translational Psychiatry*. 2013;3. doi:10.1038/tp.2013.25

738. Laloyaux J, Dessart G, van der Linden M, Lemaire M, Larøi F. Maladaptive emotion regulation strategies and stress sensitivity mediate the relation between adverse life events and attenuated positive psychotic symptoms. *Cognitive Neuropsychiatry*. 2016;21(2):116-129. doi:10.1080/13546805.2015.1137213

739. Langbein K, Schmidt U, Schack S, et al. State marker properties of niacin skin sensitivity in ultra-high risk groups for psychosis - An optical reflection spectroscopy study. *Schizophrenia Research*. 2018;192:377-384. doi:10.1016/j.schres.2017.06.007

740. Lappin JM, Morgan KD, Valmaggia LR, et al. Insight in individuals with an At Risk Mental State. *Schizophrenia Research*. 2007;90(1-3):238-244. doi:10.1016/j.schres.2006.11.018

741. Larsen EM, Herrera S, Bilgrami ZR, et al. Self-stigma related feelings of shame and facial fear recognition in individuals at clinical high risk for psychosis: A brief report. *Schizophrenia Research*. 2019;208:483-485. doi:10.1016/j.schres.2019.01.027

742. Larson MK. *The Relationship between Alcohol/Cannabis Use and Symptom Profile and Progression in Individuals at Risk for Psychosis*. 2011.

743. Laskaris L, Zalesky A, Weickert CS, et al. Investigation of peripheral complement factors across stages of psychosis. *Schizophrenia Research*. 2019;204:30-37. doi:10.1016/j.schres.2018.11.035

744. Lataster T, Myin-Germeys I, Derom C, Thiery E, van Os J. Evidence that self-reported psychotic experiences represent the transitory developmental expression of genetic liability to psychosis in the general population. *American Journal of Medical Genetics, Part B: Neuropsychiatric Genetics*. 2009;150(8):1078-1084. doi:10.1002/ajmg.b.30933

745. Lavoie S, Schäfer MR, Whitford TJ, et al. Frontal delta power associated with negative symptoms in ultra-high risk individuals who transitioned to psychosis. *Schizophrenia Research*. 2012;138(2-3):206-211. doi:10.1016/j.schres.2012.03.033

746. Lavoie S, Bartholomeuz CF, Nelson B, et al. Sulcogyral pattern and sulcal count of the orbitofrontal cortex in individuals at ultra high risk for psychosis. *Schizophrenia Research*. 2014;154(1-3). doi:10.1016/j.schres.2014.02.008

747. Lavoie S, Whitford TJ, Benninger F, et al. Correlates of electroencephalographic resting states and erythrocyte membrane docosahexaenoic and eicosapentaenoic acid levels in individuals at ultra-high risk of psychosis. *Australian & New Zealand Journal of Psychiatry*. 2016;50(1):56-63. doi:10.1177/0004867415571168

748. Lavoie S, Berger M, Schlögelhofer M, et al. Erythrocyte glutathione levels as long-term predictor of transition to psychosis. *Translational Psychiatry*. 2017;7(3):e1064-e1064. doi:10.1038/tp.2017.30

749. Lavoie S, Jack BN, Griffiths O, et al. Impaired mismatch negativity to frequency deviants in individuals at ultra-high risk for psychosis, and preliminary evidence for further impairment with transition to psychosis. *Schizophrenia Research*. 2018;191:95-100. doi:10.1016/j.schres.2017.11.005

750. Leanza L, Egloff L, Studerus E, et al. The relationship between negative symptoms and cognitive functioning in patients at clinical high risk for psychosis. *Psychiatry Research*. 2018;268:21-27. doi:10.1016/j.psychres.2018.06.047

751. Lederman O, Rosenbaum S, Maloney C, Curtis J, Ward PB. Modifiable cardiometabolic risk factors in youth with at-risk mental states: A cross-sectional pilot study. *Psychiatry Research*. 2017;257:424-430. doi:10.1016/j.psychres.2017.08.034

752. Lee SJ, Yoo SY, Kang DH, et al. Potential vulnerability markers within the affective domain in subjects at genetic and clinical high risk for schizophrenia. *Psychopathology*. 2008;41(4):236-244. doi:10.1159/000125557

753. Lee SY, Namkoong K, Cho HH, Song DH, An SK. Reduced visual P300 amplitudes in individuals at ultra-high risk for psychosis and first-episode schizophrenia. *Neuroscience Letters*. 2010;486(3):156-160. doi:10.1016/j.neulet.2010.09.035

754. Lee SY, Kim KR, Park JY, et al. Coping Strategies and Their Relationship to Psychopathologies in People at Ultra High-Risk for Psychosis and With Schizophrenia. *Journal of Nervous & Mental Disease*. 2011;199(2):106-110. doi:10.1097/NMD.0b013e3182083b96

755. Lee YJ, Cho SJ, Cho IH, Jang JH, Kim SJ. The relationship between psychotic-like experiences and sleep disturbances in adolescents. *Sleep Medicine*. 2012;13(8):1021-1027. doi:10.1016/j.sleep.2012.06.002

756. Lee TY, Kim SN, Jang JH, et al. Neural correlate of impulsivity in subjects at ultra-high risk for psychosis. *Progress in Neuro-Psychopharmacology and Biological Psychiatry*. 2013;45:165-169. doi:10.1016/j.pnpbp.2013.04.008

757. Lee TY, Shin YS, Shin NY, et al. Neurocognitive function as a possible marker for remission from clinical high risk for psychosis. *Schizophrenia Research*. 2014;153(1-3):48-53. doi:10.1016/j.schres.2014.01.018

758. Lee TY, Kim SN, Correll CU, et al. Symptomatic and functional remission of subjects at clinical high risk for psychosis: A 2-year naturalistic observational study. *Schizophrenia Research*. 2014;156(2-3):266-271. doi:10.1016/j.schres.2014.04.002

759. Lee SY, Bang M, Kim KR, et al. Impaired facial emotion recognition in individuals at ultra-high risk for psychosis and with first-episode schizophrenia, and their associations with neurocognitive deficits and self-reported schizotypy. *Schizophrenia Research*. 2015;165(1):60-65. doi:10.1016/j.schres.2015.03.026

760. Lee J, Nuechterlein KH, Knowlton BJ, et al. Episodic Memory for Dynamic Social Interaction Across Phase of Illness in Schizophrenia. *Schizophrenia Bulletin*. 2018;44(3):620-630. doi:10.1093/schbul/sbx081

761. Lee TY, Hwang WJ, Kim NS, et al. Prediction of psychosis: Model development and internal validation of a personalized risk calculator. *Psychological Medicine*. Published online 2020. doi:10.1017/S0033291720004675

762. Lehembre-Shiah E, Leong W, Brucato G, et al. Distinct Relationships Between Visual and Auditory Perceptual Abnormalities and Conversion to Psychosis in a Clinical High-Risk Population. *JAMA Psychiatry*. 2017;74(1):104. doi:10.1001/jamapsychiatry.2016.3055

763. Lemmers-Jansen ILJ, Fett AKJ, Hanssen E, Veltman DJ, Krabbendam L. Learning to trust: social feedback normalizes trust behavior in first-episode psychosis and clinical high risk. *Psychological Medicine*. 2019;49(5):780-790. doi:10.1017/S003329171800140X

764. Lemmers-Jansen ILJ, Fett AKJ, van Doesum NJ, van Lange PAM, Veltman DJ, Krabbendam L. Social Mindfulness and Psychosis: Neural Response to Socially Mindful Behavior in First-Episode Psychosis and Patients at Clinical High-Risk. *Frontiers in Human Neuroscience*. 2019;13. doi:10.3389/fnhum.2019.00047

765. Lemmers-Jansen IL, Fett AKJ, van Os J, Veltman DJ, Krabbendam L. Trust and the city: Linking urban upbringing to neural mechanisms of trust in psychosis. *Australian & New Zealand Journal of Psychiatry*. 2020;54(2):138-149. doi:10.1177/0004867419865939

766. Lemos S, Vallina O, Fernández P, et al. Predictive validity of the Scale of Prodromal Symptoms (SOPS). *Actas espanolas de psiquiatria*. 2006;34(4):216-223.

767. Lemos-Giráldez S, Vallina-Fernández O, Fernández-Iglesias P, et al. Symptomatic and functional outcome in youth at ultra-high risk for psychosis: A longitudinal study. *Schizophrenia Research*. 2009;115(2-3):121-129. doi:10.1016/j.schres.2009.09.011

768. Lencz T, Smith CW, Auther AM, Correll CU, Cornblatt BA. The Assessment of “Prodromal Schizophrenia”: Unresolved Issues and Future Directions. In: *Schizophrenia Bulletin*. Vol 29. DHHS Public Health Service; 2003:717-728. doi:10.1093/oxfordjournals.schbul.a007041

769. Lencz T, Smith CW, McLaughlin D, et al. Generalized and Specific Neurocognitive Deficits in Prodromal Schizophrenia. *Biological Psychiatry*. 2006;59(9):863-871. doi:10.1016/j.biopsych.2005.09.005

770. Lennertz L, Wagner M, Wölwer W, et al. A promoter variant of SHANK1 affects auditory working memory in schizophrenia patients and in subjects clinically at risk for psychosis. *European Archives of Psychiatry and Clinical Neuroscience*. 2012;262(2):117-124. doi:10.1007/s00406-011-0233-3

771. Lepock JR, Mizrahi R, Korostil M, et al. N400 event-related brain potential evidence for semantic priming deficits in persons at clinical high risk for psychosis. *Schizophrenia Research*. 2019;204:434-436. doi:10.1016/j.schres.2018.08.033

772. Lewis G, Dykxhoorn J, Karlsson H, et al. Assessment of the Role of IQ in Associations Between Population Density and Deprivation and Nonaffective Psychosis. *JAMA Psychiatry*. 2020;77(7):729. doi:10.1001/jamapsychiatry.2020.0103

773. Lho SK, Oh S, Moon SY, et al. Reliability and validity of the Korean version of the comprehensive assessment of at-risk mental states. *Early Intervention in Psychiatry*. Published online 2021. doi:10.1111/eip.13123

774. Li H, Yang S, Chi H, et al. Enhancing attention and memory of individuals at clinical high risk for psychosis with mHealth technology. *Asian Journal of Psychiatry*. 2021;58. doi:10.1016/j.ajp.2021.102587

775. Li R, Lyu H, Liu F, et al. Altered functional connectivity strength and its correlations with cognitive function in subjects with ultra‐high risk for psychosis at rest. *CNS Neuroscience & Therapeutics*. 2018;24(12):1140-1148. doi:10.1111/cns.12865

776. Li XB, Wang LB, Xiong YB, et al. Altered resting-state functional connectivity of the insula in individuals with clinical high-risk and patients with first-episode schizophrenia. *Psychiatry Research*. 2019;282:112608. doi:10.1016/j.psychres.2019.112608

777. Lian N, Lv H, Guo W, et al. A comparative study of magnetic resonance imaging on the gray matter and resting-state function in prodromal and first-episode schizophrenia. *American Journal of Medical Genetics Part B: Neuropsychiatric Genetics*. 2018;177(6):537-545. doi:10.1002/ajmg.b.32644

778. Liemburg E, Sibeijn-Kuiper A, Bais L, et al. Prefrontal NAA and Glx Levels in Different Stages of Psychotic Disorders: a 3T 1H-MRS Study. *Scientific Reports*. 2016;6(1):21873. doi:10.1038/srep21873

779. Lin A, Wood SJ, Nelson B, et al. Neurocognitive predictors of functional outcome two to 13years after identification as ultra-high risk for psychosis. *Schizophrenia Research*. 2011;132(1):1-7. doi:10.1016/j.schres.2011.06.014

780. Lin A, Wigman JTW, Nelson B, et al. Follow-up factor structure of schizotypy and its clinical associations in a help-seeking sample meeting ultra-high risk for psychosis criteria at baseline. *Comprehensive Psychiatry*. 2013;54(2):173-180. doi:10.1016/j.comppsych.2012.06.011

781. Lin A, Yung AR, Nelson B, et al. Neurocognitive predictors of transition to psychosis: medium- to long-term findings from a sample at ultra-high risk for psychosis. *Psychological Medicine*. 2013;43(11):2349-2360. doi:10.1017/S0033291713000123

782. Lin A, Brewer WJ, Yung AR, Nelson B, Pantelis C, Wood SJ. Olfactory identification deficits at identification as ultra-high risk for psychosis are associated with poor functional outcome. *Schizophrenia Research*. 2015;161(2-3):156-162. doi:10.1016/j.schres.2014.10.051

783. Lin K, Shao R, Lu R, et al. Resting-state fMRI signals in offspring of parents with bipolar disorder at the high-risk and ultra-high-risk stages and their relations with cognitive function. *Journal of Psychiatric Research*. 2018;98:99-106. doi:10.1016/j.jpsychires.2018.01.001

784. Lindgren M, Manninen M, Laajasalo T, et al. The relationship between psychotic-like symptoms and neurocognitive performance in a general adolescent psychiatric sample. *Schizophrenia Research*. 2010;123(1):77-85. doi:10.1016/j.schres.2010.07.025

785. Lindgren M, Manninen M, Kalska H, et al. Evaluation of verbal list learning as a predictor of psychosis. *Early Intervention in Psychiatry*. 2017;11(2):171-176. doi:10.1111/eip.12287

786. Lindgren M, Jonninen M, Jokela M, Therman S. Adolescent psychosis risk symptoms predicting persistent psychiatric service use: A 7-year follow-up study. *European Psychiatry*. 2019;55:102-108. doi:10.1016/j.eurpsy.2018.10.004

787. Lindgren M, Kuvaja H, Jokela M, Therman S. Predictive validity of psychosis risk models when applied to adolescent psychiatric patients. *Psychological Medicine*. Published online 2021. doi:10.1017/S0033291721001938

788. Liu CC, Hwu HG, Chiu YN, Lai MC, Tseng HH. Creating a platform to bridge service and research for early psychosis. *Journal of the Formosan Medical Association*. 2010;109(7):543-549. doi:10.1016/S0929-6646(10)60089-7

789. Liu CC, Lai MC, Liu CM, et al. Follow-up of subjects with suspected pre-psychotic state in Taiwan. *Schizophrenia Research*. 2011;126(1-3):65-70. doi:10.1016/j.schres.2010.10.028

790. Liu CC, Tien YJ, Chen CH, et al. Development of a brief self-report questionnaire for screening putative pre-psychotic states. *Schizophrenia Research*. 2013;143(1):32-37. doi:10.1016/j.schres.2012.10.042

791. Liu CC, Chien YL, Hsieh MH, Hwang TJ, Hwu HG, Liu CM. Aripiprazole for Drug-Naive or Antipsychotic-Short-Exposure Subjects With Ultra-High Risk State and First-Episode Psychosis. *Journal of Clinical Psychopharmacology*. 2013;33(1):18-23. doi:10.1097/JCP.0b013e31827cb017

792. Liu CC, Hua MS, Hwang TJ, et al. Neurocognitive functioning of subjects with putative pre-psychotic states and early psychosis. *Schizophrenia Research*. 2015;164(1-3):40-46. doi:10.1016/j.schres.2015.03.006

793. Liu Y, Wang G, Jin H, et al. Cognitive deficits in subjects at risk for psychosis, first-episode and chronic schizophrenia patients. *Psychiatry Research*. 2019;274:235-242. doi:10.1016/j.psychres.2019.01.089

794. Loch AA, Chianca C, Alves TM, et al. Poverty, low education, and the expression of psychotic-like experiences in the general population of São Paulo, Brazil. *Psychiatry Research*. 2017;253:182-188. doi:10.1016/j.psychres.2017.03.052

795. Loch AA, Freitas EL, Hortêncio L, et al. Hearing spirits? Religiosity in individuals at risk for psychosis—Results from the Brazilian SSAPP cohort. *Schizophrenia Research*. 2019;204:353-359. doi:10.1016/j.schres.2018.09.020

796. Loewy RL, Bearden CE, Johnson JK, Raine A, Cannon TD. The prodromal questionnaire (PQ): preliminary validation of a self-report screening measure for prodromal and psychotic syndromes. *Schizophr Res*. 2005;79(1):117-125.

797. Loewy RL, Johnson JK, Cannon TD. Self-report of attenuated psychotic experiences in a college population. *Schizophrenia Research*. 2007;93(1-3):144-151. doi:10.1016/j.schres.2007.02.010

798. Loewy RL, Pearson R, Vinogradov S, Bearden CE, Cannon TD. Psychosis risk screening with the Prodromal Questionnaire — Brief Version (PQ-B). *Schizophrenia Research*. 2011;129(1):42-46. doi:10.1016/j.schres.2011.03.029

799. Loewy RL, Therman S, Manninen M, Huttunen MO, Cannon TD. Prodromal psychosis screening in adolescent psychiatry clinics. *Early Intervention in Psychiatry*. 2012;6(1):69-75. doi:10.1111/j.1751-7893.2011.00286.x

800. Loewy R, Fisher M, Schlosser DA, et al. Intensive Auditory Cognitive Training Improves Verbal Memory in Adolescents and Young Adults at Clinical High Risk for Psychosis. *Schizophrenia Bulletin*. 2016;42(suppl 1):S118-S126. doi:10.1093/schbul/sbw009

801. Long X, Liu F, Huang N, et al. Brain regional homogeneity and function connectivity in attenuated psychosis syndrome —based on a resting state fMRI study. *BMC Psychiatry*. 2018;18(1):383. doi:10.1186/s12888-018-1954-x

802. LoPilato AM, Goines K, Addington J, et al. Impact of childhood adversity on corticolimbic volumes in youth at clinical high-risk for psychosis. *Schizophrenia Research*. 2019;213:48-55. doi:10.1016/j.schres.2019.01.048

803. LoPilato AM, Zhang Y, Pike M, et al. Associations between childhood adversity, cognitive schemas and attenuated psychotic symptoms. *Early Intervention in Psychiatry*. 2021;15(4):818-827. doi:10.1111/eip.13017

804. Lord LD, Allen P, Expert P, et al. Characterization of the anterior cingulate’s role in the at-risk mental state using graph theory. *Neuroimage*. 2011;56(3):1531-1539. doi:10.1016/j.neuroimage.2011.02.012

805. Lorenzo P, Silvia A, Federica P, et al. The Italian version of the 16-item prodromal questionnaire (iPQ-16): Field-test and psychometric features. *Schizophrenia Research*. 2018;199:353-360. doi:10.1016/j.schres.2018.03.023

806. Loughland C. Visual scanpath dysfunction in first-degree relatives of schizophrenia probands: evidence for a vulnerability marker? *Schizophrenia Research*. 2004;67(1):11-21. doi:10.1016/S0920-9964(03)00094-X

807. Louza MR, Azevedo Y, Macedo G, Gattaz W. An early psychosis research program in Sao Paulo, Brazil. Organization and implementation. *Clinical Neuropsychiatry*. 2008;5(6):273-278.

808. Lu Y, Marshall C, Cadenhead KS, et al. Perceptual abnormalities in clinical high risk youth and the role of trauma, cannabis use and anxiety. *Psychiatry Research*. 2017;258:462-468. doi:10.1016/j.psychres.2017.08.045

809. Lucas-Molina B, Pérez-Albéniz A, Satorres E, Ortuño-Sierra J, Domínguez Garrido E, Fonseca-Pedrero E. Identifying extended psychosis phenotypes at school: Associations with socio-emotional adjustment, academic, and neurocognitive outcomes. *PLOS ONE*. 2020;15(8):e0237968. doi:10.1371/journal.pone.0237968

810. Lucy Poe S, Gill KE, Brucato G, Corcoran CM, Girgis RR. Family history of psychosis as a predictor or protective factor of social maladjustment in a population at clinical high risk for psychosis. *Psychiatry Research*. 2014;219(3):696-699. doi:10.1016/j.psychres.2014.07.007

811. Lunsford-Avery JR, Orr JM, Gupta T, et al. Sleep dysfunction and thalamic abnormalities in adolescents at ultra high-risk for psychosis. *Schizophrenia Research*. 2013;151(1-3):148-153. doi:10.1016/j.schres.2013.09.015

812. Lunsford-Avery JR, LeBourgeois MK, Gupta T, Mittal VA. Actigraphic-measured sleep disturbance predicts increased positive symptoms in adolescents at ultra high-risk for psychosis: A longitudinal study. *Schizophrenia Research*. 2015;164(1-3):15-20. doi:10.1016/j.schres.2015.03.013

813. Lunsford-Avery JR, Gonçalves B da SB, Brietzke E, et al. Adolescents at clinical-high risk for psychosis: Circadian rhythm disturbances predict worsened prognosis at 1-year follow-up. *Schizophrenia Research*. 2017;189:37-42. doi:10.1016/j.schres.2017.01.051

814. Lunsford-Avery JR, Dean DJ, Mittal VA. Self-reported sleep disturbances associated with procedural learning impairment in adolescents at ultra-high risk for psychosis. *Schizophrenia Research*. 2017;190:160-163. doi:10.1016/j.schres.2017.03.025

815. Luo Y, Zhang J, Wang C, et al. Discriminating schizophrenia disease progression using a P50 sensory gating task with dense-array EEG, clinical assessments, and cognitive tests. *Expert Review of Neurotherapeutics*. 2019;19(5):459-470. doi:10.1080/14737175.2019.1601558

816. Lyngberg K, Buchy L, Liu L, Perkins D, Woods S, Addington J. Patterns of premorbid functioning in individuals at clinical high risk of psychosis. *Schizophrenia Research*. 2015;169(1-3):209-213. doi:10.1016/j.schres.2015.11.004
