# Supplementary material for: Meta-analytic prevalence of comorbid mental disorders in individuals at clinical high risk of psychosis: the case for transdiagnostic assessment
Source: Mol Psychiatry. 2023 Jun 9;28(6):2291–300. doi: 10.1038/s41380-023-02029-8 (PMC10611568; doi:10.1038/s41380-023-02029-8)
Supplement: Supplementary file 3 — Supplementary material part 3 [file 41380_2023_2029_MOESM3_ESM.docx]

**APPENDIX 3 – Studies excluded after full-text assessment, part 2**

**Meta-analytic prevalence of comorbid mental disorders in individuals at clinical high risk of psychosis: the case for transdiagnostic assessment**

Marco Solmi1-6, Livia Soardo7, Simi Kaur5, Matilza Azis5, Anna Cabras8, Marco Censori9-10, Luigi Fausti7, Filippo Besana7, Gonzalo Salazar de Pablo5-11-13, Paolo Fusar-Poli5,7

1 Department of Psychiatry, University of Ottawa, Ontario, Canada.

2 On Track, First Episode Psychosis Program, Department of Mental Health, The Ottawa Hospital, Ontario, Canada.

3 Ottawa Hospital Research Institute (OHRI) Clinical Epidemiology Program University of Ottawa Ottawa Ontario

4 School of Epidemiology and Public Health, Faculty of Medicine, University of Ottawa, Ottawa, Canada

5 Early Psychosis: Interventions and Clinical-detection (EPIC) Lab, Institute of Psychiatry, Psychology & Neuroscience, Department of Psychosis Studies, King's College London, London, United Kingdom.

6 Department of Child and Adolescent Psychiatry, Charité Universitätsmedizin, Berlin, Germany

7 Department of Brain and Behavioral Sciences, University of Pavia, Pavia, Italy

8 Sapienza University of Rome, Department of Neurology and Psychiatry

9 Department of Neuroscience (DNS), University of Padova, Padua, Italy

10 Dipartimento di Salute Mentale, Azienda ULSS 3 Serenissima, Venezia, Italy

11 Department of Child and Adolescent Psychiatry, Institute of Psychiatry, Psychology & Neuroscience, King’s College London UK

12 Child and Adolescent Mental Health Services, South London and Maudsley NHS Foundation Trust, London, UK

13 Institute of Psychiatry and Mental Health. Department of Child and Adolescent Psychiatry, Hospital General Universitario Gregorio Marañón School of Medicine, Universidad Complutense, Instituto de Investigación Sanitaria Gregorio Marañón (IiSGM), CIBERSAM, Madrid, Spain

Corresponding author

Paolo Fusar-Poli

paolo.fusar-poli@kcl.ac.uk

| **Author, year** | **Reason for exclusion after full-text assessment** |
| --- | --- |
| 1. MacCabe 2013 ^1^ | No CHR-P population |
| 1. Madeira 2016 ^2^ | No relevant outcome |
| 1. Madeira 2016 ^3^ | No relevant outcome |
| 1. Magaud 2010 ^4^ | No dsm/icd/cut off scale criteria |
| 1. Magaud 2013 ^5^ | No relevant outcome |
| 1. Magaud 2014 ^6^ | No dsm/icd/cut off scale criteria |
| 1. Maggini 2004 ^7^ | No CHR-P population |
| 1. Malla 2018 ^8^ | No dsm/icd/cut off scale criteria |
| 1. Malm 1995 ^9^ | No CHR-P population |
| 1. Mamah 2012 ^10^ | No CHR-P population |
| 1. Mamah 2013 ^11^ | No relevant outcome |
| 1. Mamah 2014 ^12^ | No CHR-P population |
| 1. Mamah 2016 ^13^ | No dsm/icd/cut off scale criteria |
| 1. Mantere 2018 ^14^ | No relevant outcome |
| 1. Manzanares 2014 ^15^ | No relevant outcome |
| 1. Mariné 2015 ^16^ | No relevant outcome |
| 1. Markulev 2017 ^17^ | No relevant outcome |
| 1. Marques 2012 ^18^ | No dsm/icd/cut off scale criteria |
| 1. Marshall 2016 ^19^ | No relevant outcome |
| 1. Marshall 2019 ^20^ | No relevant outcome |
| 1. Martinez 2018 ^21^ | No relevant outcome |
| 1. Martorell 2019^22^ | No dsm/icd/cut off scale criteria |
| 1. Marulanda 2016 ^23^ | No dsm/icd/cut off scale criteria |
| 1. Marvin 2016 ^24^ | No relevant outcome |
| 1. Masaoka 2020 ^25^ | No relevant outcome |
| 1. Masillo 2012 ^26^ | No dsm/icd/cut off scale criteria |
| 1. Masillo 2016 ^27^ | No relevant outcome |
| 1. Masillo 2018 ^28^ | No relevant outcome |
| 1. Masillo 2018 ^29^ | No relevant outcome |
| 1. Masillo 2019 ^30^ | No relevant outcome |
| 1. Mason 2004 ^31^ | No CHR-P population |
| 1. Masucci 2018 ^32^ | No relevant outcome |
| 1. Mathalon 2019 ^33^ | No relevant outcome |
| 1. Matsumoto 2019 ^34^ | No dsm/icd/cut off scale criteria |
| 1. Maurya 2017 ^35^ | No relevant outcome |
| 1. Mayeli 2021 ^36^ | No relevant outcome |
| 1. McDonnell 2018 ^37^ | No relevant outcome |
| 1. McFarlane 2010 ^38^ | No relevant outcome |
| 1. McFarlane 2014 ^39^ | No relevant outcome |
| 1. McGlashan 2003 ^40^ | Study Design |
| 1. McGlashan 2006 ^41^ | No relevant outcome |
| 1. McGorry 1995 ^42^ | No CHR-P population |
| 1. McGorry 2002 ^43^ | No dsm/icd/cut off scale criteria |
| 1. McGorry 2002^44^ | No CHR-P population |
| 1. McGorry 2013^45^ | No relevant outcome |
| 1. McGorry 2017 ^46^ | No relevant outcome |
| 1. McGuire 2009 ^47^ | Study Design |
| 1. McGuire 2011 ^48^ | Study Design |
| 1. McHugh 2017 ^49^ | No dsm/icd/cut off scale criteria |
| 1. McHugh 2018 ^50^ | No dsm/icd/cut off scale criteria |
| 1. McIntosh 2007 ^51^ | No relevant outcome |
| 1. McKechanie 2016 ^52^ | No dsm/icd/cut off scale criteria |
| 1. McLaughlin 2016 ^53^ | No dsm/icd/cut off scale criteria |
| 1. McNeil 2009 ^54^ | No CHR-P population |
| 1. Mechelli 2011 ^55^ | No relevant outcome |
| 1. Mechelli 2017 ^56^ | No relevant outcome |
| 1. Meijer 2011 ^57^ | No relevant outcome |
| 1. Meisenzahl 2008 ^58^ | No relevant outcome |
| 1. Melton 2013 ^59^ | No relevant outcome |
| 1. Meneghelli 2003 ^60^ | Article not in English |
| 1. Meneghelli 2011 ^61^ | No relevant outcome |
| 1. Meneghelli 2014 ^62^ | Article not in English |
| 1. Meng 2009 ^63^ | No CHR-P population |
| 1. Mennigen 2018 ^64^ | No relevant outcome |
| 1. Mennigen 2019 ^65^ | No relevant outcome |
| 1. Menschikov 2016 ^66^ | No relevant outcome |
| 1. Metzler 2016 ^67^ | No dsm/icd/cut off scale criteria |
| 1. Meyer 2014 ^68^ | No relevant outcome |
| 1. Michel 2014 ^69^ | Study Design |
| 1. Michel 2017 ^70^ | No relevant outcome |
| 1. Michel 2019 ^71^ | No relevant outcome |
| 1. Michels 2014 ^72^ | No relevant outcome |
| 1. Miettunen 2008 ^73^ | No dsm/icd/cut off scale criteria |
| 1. Miklowitz 2014 ^74^ | No relevant outcome |
| 1. Miller 1999 ^75^ | Study Design |
| 1. Miller 2002 ^76^ | No relevant outcome |
| 1. Miller 2003 ^77^ | No dsm/icd/cut off scale criteria |
| 1. Millman 2014 ^78^ | No relevant outcome |
| 1. Millman 2020^79^ | No relevant outcome |
| 1. Mills 2017 ^80^ | No relevant outcome |
| 1. Minichino 2017 ^81^ | No relevant outcome |
| 1. Minichino 2019 ^82^ | No CHR-P population |
| 1. Miron 2017 ^83^ | Study Design |
| 1. Mirzakhanian 2013 ^84^ | No relevant outcome |
| 1. Mittal 2007 ^85^ | No CHR-P population |
| 1. Mittal 2008 ^86^ | Study Design |
| 1. Mittal 2008 ^87^ | No CHR-P population |
| 1. Mittal 2009 ^88^ | No relevant outcome |
| Mittal 2009^89^. | No CHR-P population |
| 1. Mittal 2010 ^90^ | No relevant outcome |
| 1. Mittal 2011 ^91^ | No relevant outcome |
| 1. Mittal 2011 ^92^ | No CHR-P population |
| 1. Mittal 2012 ^93^ | No CHR-P population |
| 1. Mittal 2013 ^94^ | No relevant outcome |
| 1. Mittal 2013 ^95^ | No CHR-P population |
| 1. Mittal 2014 ^96^ | No relevant outcome |
| 1. Mittal 2015 ^97^ | No relevant outcome |
| 1. Mitter 2014 ^98^ | No relevant outcome |
| 1. Miyakoshi 2009 ^99^ | No relevant outcome |
| 1. Mizrahi 2012 ^100^ | No dsm/icd/cut off scale criteria |
| 1. Mizrahi 2014 ^101^ | No dsm/icd/cut off scale criteria |
| 1. Modinos 2009 ^102^ | Study Design |
| 1. Modinos 2010 ^103^ | No relevant outcome |
| 1. Modinos 2012 ^104^ | No CHR-P population |
| 1. Modinos 2015 ^105^ | No relevant outcome |
| 1. Modinos 2018 ^106^ | No relevant outcome |
| 1. Modinos 2020 ^107^ | No dsm/icd/cut off scale criteria |
| 1. Mondragòn-Maya 2013 ^108^ | No relevant outcome |
| 1. Mondrup 2010 ^109^ | No dsm/icd/cut off scale criteria |
| 1. Montagnese 2020 ^110^ | No relevant outcome |
| 1. Montalvo 2014 ^111^ | No dsm/icd/cut off scale criteria |
| 1. Montojo 2014 ^112^ | No CHR-P population |
| 1. Montreuil 2016 ^113^ | Study Design |
| 1. Moorhead 2009 ^114^ | No CHR-P population |
| 1. Morelli 2019 ^115^ | No dsm/icd/cut off scale criteria |
| 1. Morey 2005 ^116^ | No relevant outcome |
| 1. Morita 2014 ^117^ | No dsm/icd/cut off scale criteria |
| 1. Morley 2008 ^118^ | No CHR-P population |
| 1. Morrison 2002 ^119^ | No dsm/icd/cut off scale criteria |
| 1. Morrison 2004 ^120^ | No dsm/icd/cut off scale criteria |
| 1. Morrison 2006 ^121^ | No relevant outcome |
| 1. Morrison 2007 ^122^ | No relevant outcome |
| 1. Morrison 2007 ^123^ | No CHR-P population |
| 1. Morrison 2011 ^124^ | No dsm/icd/cut off scale criteria |
| 1. Morrison 2013 ^125^ | No relevant outcome |
| 1. Moskow 2016 ^126^ | No relevant outcome |
| 1. Mossaheb 2012 ^127^ | No relevant outcome |
| 1. Mossaheb 2013 ^128^ | No relevant outcome |
| 1. Mossaheb 2018 ^129^ | No dsm/icd/cut off scale criteria |
| 1. Mossaheb 2018 ^130^ | No relevant outcome |
| 1. Mössner 2010 ^131^ | No relevant outcome |
| 1. Moukas 2010 ^132^ | No dsm/icd/cut off scale criteria |
| 1. Mourik 2017 ^133^ | No relevant outcome |
| 1. Mukkala 2011 ^134^ | No relevant outcome |
| 1. Müller 2018 ^135^ | No dsm/icd/cut off scale criteria |
| 1. Murphy 2012 ^136^ | No relevant outcome |
| 1. Murphy 2013 ^137^ | No relevant outcome |
| 1. Myin-Germeys 2005 ^138^ | No CHR-P population |
| 1. Myin-Germeys 2005 ^139^ | No CHR-P population |
| 1. Myles-Worsley 2004 ^140^ | No dsm/icd/cut off scale criteria |
| 1. Myles-Worsley 2007 ^141^ | No relevant outcome |
| 1. Mӓki 2004 ^142^ | Study Design |
| 1. Mӓki 2014 ^143^ | No CHR-P population |
| 1. Nagai 2013 ^144^ | No relevant outcome |
| 1. Nagai 2017 ^145^ | No relevant outcome |
| 1. Nakamura 2013 ^146^ | No dsm/icd/cut off scale criteria |
| 1. Nakamura 2019 ^147^ | No relevant outcome |
| 1. Naqvi 2014 ^148^ | No dsm/icd/cut off scale criteria |
| 1. Natsubori 2014 ^149^ | No relevant outcome |
| 1. Negueruela-Lopez 2012 ^150^ | Study Design |
| 1. Nelson 2007 ^151^ | No dsm/icd/cut off scale criteria |
| 1. Nelson 2010 ^152^ | No relevant outcome |
| 1. Nelson 2011 ^153^ | No relevant outcome |
| 1. Nelson 2012^154^ | Study Design |
| 1. Nelson 2013 ^155^ | No relevant outcome |
| 1. Nelson 2013 ^156^ | No relevant outcome |
| 1. Nelson 2016 ^157^ | No relevant outcome |
| 1. Nelson 2018 ^158^ | No dsm/icd/cut off scale criteria |
| 1. Nelson 2019 ^159^ | Study Design |
| 1. Nelson 2019 ^160^ | No relevant outcome |
| 1. Nelson 2020 ^161^ | Study Design |
| 1. Nelson 2020 ^162^ | Study Design |
| 1. Nenadic 2015 ^163^ | No relevant outcome |
| 1. Nenadic 2015 ^164^ | No relevant outcome |
| 1. Newberry 2018 ^165^ | No relevant outcome |
| 1. Ngiralmau 2005 ^166^ | No CHR-P population |
| 1. Nieman 2007 ^167^ | No relevant outcome |
| 1. Nieman 2009 ^168^ | No relevant outcome |
| 1. Nieman 2013 ^169^ | No relevant outcome |
| 1. Nieman 2014 ^170^ | No relevant outcome |
| 1. Nieman 2016 ^171^ | No relevant outcome |
| 1. Niemi 2005 ^172^ | No CHR-P population |
| 1. Niendam 2007 ^173^ | No relevant outcome |
| 1. Niendam 2007 ^174^ | No relevant outcome |
| 1. Niendam 2007 ^175^ | No relevant outcome |
| 1. Niendam 2014 ^176^ | No relevant outcome |
| 1. Niles 2019 ^177^ | No relevant outcome |
| 1. Nitka 2016 ^178^ | No relevant outcome |
| 1. Nogovitsyn 2020 ^179^ | No dsm/icd/cut off scale criteria |
| 1. Nogueira 2021 ^180^ | No dsm/icd/cut off scale criteria |
| 1. Nordholm 2016 ^181^ | No relevant outcome |
| 1. Nordholm 2018 ^182^ | No relevant outcome |
| 1. Novak Sarotar 2008 ^183^ | No CHR-P population |
| 1. Nowak 2018 ^184^ | No dsm/icd/cut off scale criteria |
| 1. Ntouros 2018 ^185^ | No relevant outcome |
| 1. Nuchpongsai 1999 ^186^ | No CHR-P population |
| 1. Nussbaum 2016 ^187^ | No relevant outcome |
| 1. Nussbaum 2017 ^188^ | No relevant outcome |
| 1. O’Banion 2010^189^ | No relevant outcome |
| 1. O’Brien 2006 ^190^ | No relevant outcome |
| 1. O’Brien 2008 ^191^ | No relevant outcome |
| 1. O’Brien 2009 ^192^ | No relevant outcome |
| 1. O’Brien 2014 ^193^ | No relevant outcome |
| 1. O’Brien 2015 ^194^ | No relevant outcome |
| 1. O’Connor 2007 ^195^ | Study Design |
| 1. O’Connor 2016 ^196^ | No relevant outcome |
| 1. O’Connor 2019 ^197^ | No CHR-P population |
| 1. Obyedkov 2019 ^198^ | No relevant outcome |
| 1. O'Donoghue 2012 ^199^ | No CHR-P population |
| 1. O'Donoghue 2015 ^200^ | No relevant outcome |
| 1. O'Donoghue 2015 ^201^ | No relevant outcome |
| 1. O'Donoghue 2018 ^202^ | No relevant outcome |
| 1. Ohmuro 2016 ^203^ | No relevant outcome |
| 1. Ohmuro 2018 ^204^ | No dsm/icd/cut off scale criteria |
| 1. Oliver 2020 ^205^ | Study Design |
| 1. Olvet 2010 ^206^ | No relevant outcome |
| 1. Olvet 2015 ^207^ | No relevant outcome |
| 1. Omer 2014 ^208^ | No CHR-P population |
| 1. Onozato 2020 ^209^ | No relevant outcome |
| 1. Onozato 2020 ^210^ | No dsm/icd/cut off scale criteria |
| 1. Ord 2008^211^ | Article not found |
| 1. Oribe 2013 ^212^ | No relevant outcome |
| 1. Oribe 2019 ^213^ | No relevant outcome |
| 1. Oribe 2020 ^214^ | No relevant outcome |
| 1. Orosz 2011 ^215^ | No relevant outcome |
| 1. Ortega 2019 ^216^ | No dsm/icd/cut off scale criteria |
| 1. Osborne 2017 ^217^ | No relevant outcome |
| 1. Osborne 2019 ^218^ | No relevant outcome |
| 1. Osborne 2021 ^219^ | No relevant outcome |
| 1. Ota 2011 ^220^ | No relevant outcome |
| 1. Owens 2005 ^221^ | No dsm/icd/cut off scale criteria |
| 1. Owoso 2014 ^222^ | No CHR-P population |
| 1. Özgürdal 2008 ^223^ | No relevant outcome |
| 1. Özgürdal 2009 ^224^ | No relevant outcome |
| 1. Padmanabhan 2017 ^225^ | No CHR-P population |
| 1. Padula 2018 ^226^ | No relevant outcome |
| 1. Paetzold 2021 ^227^ | No dsm/icd/cut off scale criteria |
| 1. Palmier-Claus 2012 ^228^ | No relevant outcome |
| 1. Palmier-Claus 2012 ^229^ | No relevant outcome |
| 1. Palmier-Claus 2013 ^230^ | No relevant outcome |
| 1. Palmier-Claus 2014 ^231^ | No dsm/icd/cut off scale criteria |
| 1. Palmier-Claus 2016 ^232^ | No relevant outcome |
| 1. Papmeyer 2016 ^233^ | No relevant outcome |
| 1. Papmeyer 2018 ^234^ | No relevant outcome |
| 1. Parham 2019 ^235^ | No relevant outcome |
| 1. Park 2013 ^236^ | No relevant outcome |
| 1. Park 2021 ^237^ | No relevant outcome |
| 1. Parker 2020 ^238^ | No dsm/icd/cut off scale criteria |
| 1. Parrish 2019 ^239^ | No relevant outcome |
| 1. Paterlini 2019 ^240^ | No relevant outcome |
| 1. Patton 2021 ^241^ | No relevant outcome |
| 1. Pauly 2010 ^242^ | No dsm/icd/cut off scale criteria |
| 1. Pawelczyk 2015 ^243^ | No relevant outcome |
| 1. Pawelczyk 2016 ^244^ | No relevant outcome |
| 1. Pawelczyk 2017 ^245^ | No relevant outcome |
| 1. Pawelczyk 2019 ^246^ | No relevant outcome |
| 1. Peedicayil 2008 ^247^ | Study Design |
| 1. Pelayo-Teran 2008 ^248^ | No CHR-P population |
| 1. Pelizza 2016 ^249^ | No dsm/icd/cut off scale criteria |
| 1. Pelizza 2018 ^250^ | No relevant outcome |
| 1. Pelizza 2019 ^251^ | No dsm/icd/cut off scale criteria |
| 1. Pelizza 2019 ^252^ | No dsm/icd/cut off scale criteria |
| 1. Pelizza 2019 ^253^ | No relevant outcome |
| 1. Pelizza 2019 ^254^ | No dsm/icd/cut off scale criteria |
| 1. Pelizza 2019 ^255^ | No relevant outcome |
| 1. Pelizza 2020 ^256^ | No relevant outcome |
| 1. Pelizza 2020 ^257^ | No dsm/icd/cut off scale criteria |
| 1. Pelizza 2021^258^ | No relevant outcome |
| 1. Pelletier-Baldelli 2017 ^259^ | No dsm/icd/cut off scale criteria |
| 1. Pelletier-Baldelli 2018 ^260^ | No dsm/icd/cut off scale criteria |
| 1. Peralta 2019 ^261^ | No relevant outcome |
| 1. Perez 2012 ^262^ | No relevant outcome |
| 1. Perez 2012 ^263^ | No relevant outcome |
| 1. Perez 2012 ^264^ | No relevant outcome |
| 1. Perez 2013 ^265^ | No relevant outcome |
| 1. Perez 2014 ^266^ | No relevant outcome |
| 1. Perivoliotis 2009 ^267^ | No dsm/icd/cut off scale criteria |
| 1. Perkins 2020 ^268^ | No relevant outcome |
| 1. Peters 2009 ^269^ | No relevant outcome |
| 1. Peters 2009 ^270^ | No CHR-P population |
| 1. Peters 2010 ^271^ | No relevant outcome |
| 1. Petruzzelli 2019 ^272^ | No relevant outcome |
| 1. Pettersson-Yeo 2013 ^273^ | No relevant outcome |
| 1. Pettersson-Yeo 2015 ^274^ | No relevant outcome |
| 1. Pflueger 2007 ^275^ | No relevant outcome |
| 1. Pflueger 2018 ^276^ | No relevant outcome |
| 1. Phillips 1999 ^277^ | No relevant outcome |
| 1. Phillips 2000 ^278^ | No dsm/icd/cut off scale criteria |
| 1. Phillips 2007 ^279^ | No relevant outcome |
| 1. Phillips 2012 ^280^ | No relevant outcome |
| 1. Pineiro 2003 ^281^ | Article not in English |
| 1. Pinkham 2007 ^282^ | No relevant outcome |
| 1. Piotrowski 2019 ^283^ | No CHR-P population |
| 1. Piskulic 2015 ^284^ | No relevant outcome |
| 1. Piskulic 2016 ^285^ | No relevant outcome |
| 1. Pitzianti 2019 ^286^ | No relevant outcome |
| 1. Pitzianti 2019 ^287^ | No relevant outcome |
| 1. Platz 2006 ^288^ | No relevant outcome |
| 1. Poe 2017 ^289^ | No relevant outcome |
| 1. Polari 2018 ^290^ | No dsm/icd/cut off scale criteria |
| 1. Polari 2020 ^291^ | No relevant outcome |
| 1. Pollice 2007 ^292^ | No dsm/icd/cut off scale criteria |
| 1. Pontillo 2020 ^293^ | Study Design |
| 1. Popovic 2020 ^294^ | No relevant outcome |
| 1. Pos 2017 ^295^ | No relevant outcome |
| 1. Pot-Kolder 2018 ^296^ | No relevant outcome |
| 1. Poustka 2007 ^297^ | No relevant outcome |
| 1. Power 2016 ^298^ | No relevant outcome |
| 1. Preti 2012 ^299^ | No relevant outcome |
| 1. Preti 2012 ^300^ | No dsm/icd/cut off scale criteria |
| 1. Preti 2021 ^301^ | No relevant outcome |
| 1. Provenzano 2020 ^302^ | No relevant outcome |
| 1. Pruessner 2013 ^303^ | No dsm/icd/cut off scale criteria |
| 1. Pruessner 2017 ^304^ | No dsm/icd/cut off scale criteria |
| 1. Pruessner 2017 ^305^ | No relevant outcome |
| 1. Pu 2020 ^306^ | No relevant outcome |
| 1. Pukrop 2006 ^307^ | No relevant outcome |
| 1. Pukrop 2007 ^308^ | No relevant outcome |
| 1. Pulkkinen 2015 ^309^ | No CHR-P population |
| 1. Purcell 2015 ^310^ | No relevant outcome |
| 1. Pyle 2015 ^311^ | No relevant outcome |
| 1. Pyle 2017 ^312^ | No dsm/icd/cut off scale criteria |
| 1. Quarmley 2019 ^313^ | No dsm/icd/cut off scale criteria |
| 1. Quednow 2008 ^314^ | No relevant outcome |
| 1. Quijada 2010 ^315^ | No dsm/icd/cut off scale criteria |
| Quijada 2010^316^ | No dsm/icd/cut off scale criteria |
| 1. Quijada 2012 ^317^ | No relevant outcome |
| 1. Quijada 2015 ^318^ | No CHR-P population |
| 1. Quinones 2021 ^319^ | No relevant outcome |
| 1. Raballo 2010 ^320^ | Study Design |
| 1. Raballo 2011 ^321^ | No dsm/icd/cut off scale criteria |
| 1. Raballo 2018 ^322^ | Study Design |
| 1. Raballo 2018 ^323^ | Study Design |
| 1. Raballo 2018 ^324^ | No relevant outcome |
| 1. Rahayu 2019 ^325^ | No CHR-P population |
| 1. Raij 2018 ^326^ | No relevant outcome |
| 1. Rajarethinam 2011 ^327^ | No CHR-P population |
| 1. Raju 2019 ^328^ | No CHR-P population |
| 1. Ramyead 2015 ^329^ | No relevant outcome |
| 1. Ramyead 2017 ^330^ | No relevant outcome |
| 1. Randers 2020 ^331^ | No dsm/icd/cut off scale criteria |
| 1. Rapado-Castro 2015 ^332^ | No dsm/icd/cut off scale criteria |
| 1. Rapado-Castro 2020 ^333^ | No relevant outcome |
| 1. Rapp 2013 ^334^ | No relevant outcome |
| 1. Rapp 2013 ^335^ | No relevant outcome |
| 1. Rapp 2017 ^336^ | No relevant outcome |
| 1. Ratheesh 2015 ^337^ | No relevant outcome |
| 1. Ratheesh 2018 ^338^ | No dsm/icd/cut off scale criteria |
| 1. Rauchensteiner 2011 ^339^ | No relevant outcome |
| 1. Rausch 2015 ^340^ | No relevant outcome |
| 1. Rausch 2016 ^341^ | No relevant outcome |
| 1. Razali 2011 ^342^ | No CHR-P population |
| Razali 2013 ^343^ | No CHR-P population |
| 1. Razali 2015 ^344^ | No relevant outcome |
| 1. Raznahan 2011 ^345^ | No CHR-P population |
| 1. Raznahan 2011 ^346^ | No CHR-P population |
| 1. Reeves 2014 ^347^ | No dsm/icd/cut off scale criteria |
| 1. Reichenberg 2016 ^348^ | Study Design |
| 1. Reininghaus 2016 ^349^ | No relevant outcome |
| 1. Reininghaus 2019 ^350^ | No dsm/icd/cut off scale criteria |
| 1. Reniers 2017 ^351^ | No relevant outcome |
| 1. Resch 2002 ^352^ | No relevant outcome |
| 1. Revheim 2014 ^353^ | No relevant outcome |
| 1. Rice 2015 ^354^ | No relevant outcome |
| 1. Rice 2019 ^355^ | No dsm/icd/cut off scale criteria |
| 1. Rice 2019 ^356^ | No dsm/icd/cut off scale criteria |
| 1. Rietdijk 2010 ^357^ | Study Design |
| 1. Rietdijk 2011 ^358^ | No CHR-P population |
| 1. Rietdijk 2011^359^ | No dsm/icd/cut off scale criteria |
| 1. Rietdijk 2012 ^360^ | No dsm/icd/cut off scale criteria |
| 1. Rietdijk 2014 ^361^ | No CHR-P population |
| 1. Rigucci 2016 ^362^ | No relevant outcome |
| 1. Ristanovic 2021 ^363^ | No relevant outcome |
| 1. Roalf 2019 ^364^ | No relevant outcome |
| 1. Robustelli 2017 ^365^ | No relevant outcome |
| 1. Roddy 2012 ^366^ | No CHR-P population |
| 1. Roiser 2013 ^367^ | No relevant outcome |
| 1. Roman-Urrestarazu 2014 ^368^ | No dsm/icd/cut off scale criteria |
| Rosen 2020^369^ | No relevant outcome |
| 1. Rosenberg 2019 ^370^ | Study Design |
| 1. Rothlisberger 2012 ^371^ | No dsm/icd/cut off scale criteria |
| 1. Rouhakhtar 2019 ^372^ | Study Design |
| 1. Ruff 2012 ^373^ | No relevant outcome |
| 1. Ruhrmann 2005 ^374^ | Study Design |
| 1. Ruhrmann 2007 ^375^ | No dsm/icd/cut off scale criteria |
| 1. Ruhrmann 2008 ^376^ | No relevant outcome |
| 1. Ruiz 2021 ^377^ | No relevant outcome |
| 1. Rüsch 2014 ^378^ | No relevant outcome |
| 1. Rüsch 2014 ^379^ | No relevant outcome |
| 1. Rüsch 2015 ^380^ | No relevant outcome |
| 1. Russo 2012 ^381^ | No relevant outcome |
| 1. Rutigliano 2018 ^382^ | No CHR-P population |
| 1. Ryan 1997 ^383^ | Study Design |
| 1. Ryan 2018 ^384^ | No relevant outcome |
| 1. Saarinen 2020 ^385^ | No CHR-P population |
| 1. Sahin 2013 ^386^ | No dsm/icd/cut off scale criteria |
| 1. Saito 2017 ^387^ | No relevant outcome |
| 1. Saito 2020 ^388^ | No relevant outcome |
| 1. Sakuma 2018 ^389^ | No relevant outcome |
| 1. Saleem 2014 ^390^ | No relevant outcome |
| 1. Salokangas 2009 ^391^ | No relevant outcome |
| 1. Salokangas 2012 ^392^ | No relevant outcome |
| 1. Salokangas 2013 ^393^ | No CHR-P population |
| 1. Salokangas 2014 ^394^ | No relevant outcome |
| 1. Salokangas 2021 ^395^ | No relevant outcome |
| 1. Sanada 2018 ^396^ | No relevant outcome |
| 1. Santoro 2015 ^397^ | No relevant outcome |
| 1. Sarac 2021 ^398^ | No relevant outcome |
| 1. Sasabayashi 2017 ^399^ | No relevant outcome |
| 1. Sasabayashi 2020 ^400^ | No relevant outcome |
| 1. Sawada 2017 ^401^ | No relevant outcome |
| 1. Scala 2013 ^402^ | No CHR-P population |
| 1. Scazza 2018 ^403^ | No relevant outcome |
| 1. Scheyer 2014 ^404^ | No dsm/icd/cut off scale criteria |
| 1. Schifani 2018 ^405^ | No dsm/icd/cut off scale criteria |
| 1. Schifani 2019 ^406^ | No relevant outcome |
| 1. Schifani 2020 ^407^ | No relevant outcome |
| 1. Schiffman 2017 ^408^ | Study Design |
| 1. Schimmelmann 2011 ^409^ | No relevant outcome |
| 1. Schlosser 2009 ^410^ | No relevant outcome |
| 1. Schlosser 2010 ^411^ | No relevant outcome |
| 1. Schlosser 2012 ^412^ | No relevant outcome |
| 1. Schlosser 2014 ^413^ | No dsm/icd/cut off scale criteria |
| 1. Schlosser 2015 ^414^ | No relevant outcome |
| 1. Schmidt 2013 ^415^ | No relevant outcome |
| 1. Schmidt 2014 ^416^ | No relevant outcome |
| 1. Schmidt 2015 ^417^ | No relevant outcome |
| 1. Schmidt 2017 ^418^ | No relevant outcome |
| 1. Schmidt 2017 ^419^ | No relevant outcome |
| 1. Schmidt 2019 ^420^ | No dsm/icd/cut off scale criteria |
| 1. Schneider 2012 ^421^ | No CHR-P population |
| 1. Schneider 2014 ^422^ | No relevant outcome |
| 1. Schneider 2019 ^423^ | No relevant outcome |
| 1. Schofield 2011 ^424^ | No relevant outcome |
| 1. Schubert 2004 ^425^ | No CHR-P population |
| 1. Schubert 2005 ^426^ | No CHR-P population |
| 1. Schubert 2007 ^427^ | No CHR-P population |
| 1. Schultze-Lutter 2007 ^428^ | No relevant outcome |
| Schultze-Lutter 2007^429^ | No relevant outcome |
| 1. Schultze-Lutter 2008 ^430^ | No relevant outcome |
| 1. Schultze-Lutter 2009 ^431^ | No relevant outcome |
| 1. Schultze-Lutter 2010 ^432^ | No CHR-P population |
| 1. Schultze-Lutter 2012^433^ | No relevant outcome |
| 1. Schultze-Lutter 2014 ^434^ | No CHR-P population |
| 1. Schultze-Lutter 2014 ^435^ | No CHR-P population |
| 1. Schultze-Lutter 2014^436^ | No relevant outcome |
| 1. Schultze-Lutter 2015 ^437^ | No CHR-P population |
| 1. Schultze-Lutter 2018 ^438^ | No relevant outcome |
| 1. Schultze-Lutter 2020 ^439^ | No relevant outcome |
| 1. Seidman 2010 ^440^ | No relevant outcome |
| 1. Seidman 2016 ^441^ | No relevant outcome |
| 1. Seiferth 2008 ^442^ | No dsm/icd/cut off scale criteria |
| 1. Seiler 2020 ^443^ | No relevant outcome |
| 1. Selten 2017 ^444^ | No relevant outcome |
| 1. Selvaraj 2018 ^445^ | No relevant outcome |
| 1. Severaid 2019 ^446^ | No dsm/icd/cut off scale criteria |
| 1. Shah 2019 ^447^ | No CHR-P population |
| 1. Shaikh 2012 ^448^ | No relevant outcome |
| 1. Shaikh 2015 ^449^ | No CHR-P population |
| 1. Shaikh 2016 ^450^ | No relevant outcome |
| 1. Shakeel 2019 ^451^ | No relevant outcome |
| 1. Shakeel 2020 ^452^ | No dsm/icd/cut off scale criteria |
| 1. Shakeel 2020 ^453^ | No relevant outcome |
| 1. Shakory 2018 ^454^ | No relevant outcome |
| 1. Shan 2019 ^455^ | No relevant outcome |
| Shapiro 2011^456^ | No relevant outcome |
| 1. Shapiro 2015 ^457^ | Article not found |
| 1. Sharma 2017 ^458^ | No CHR-P population |
| 1. Sheinbaum 2015 ^459^ | No relevant outcome |
| 1. Shetty 2021 ^460^ | No relevant outcome |
| 1. Shevlin 2009 ^461^ | No CHR-P population |
| 1. Shi 2016 ^462^ | No relevant outcome |
| 1. Shim 2008 ^463^ | No dsm/icd/cut off scale criteria |
| 1. Shim 2008 ^464^ | No dsm/icd/cut off scale criteria |
| 1. Shim 2010 ^465^ | No relevant outcome |
| 1. Shin 2009 ^466^ | No dsm/icd/cut off scale criteria |
| 1. Shin 2010 ^467^ | No relevant outcome |
| 1. Shin 2012 ^468^ | No relevant outcome |
| 1. Shin 2012 ^469^ | No dsm/icd/cut off scale criteria |
| 1. Shin 2013 ^470^ | No dsm/icd/cut off scale criteria |
| 1. Shin 2016 ^471^ | No relevant outcome |
| 1. Sichlinger 2019 ^472^ | No dsm/icd/cut off scale criteria |
| 1. Siira 2004 ^473^ | No CHR-P population |
| 1. Silverstein 2006 ^474^ | No relevant outcome |
| Simeonova 2009^475^ | No relevant outcome |
| 1. Simeonova 2011 ^476^ | No dsm/icd/cut off scale criteria |
| 1. Simeonova 2014 ^477^ | No relevant outcome |
| 1. Simon 2009 ^478^ | No CHR-P population |
| 1. Simon 2009 ^479^ | No relevant outcome |
| 1. Simon 2014 ^480^ | No relevant outcome |
| 1. Sinclair-McBride 2018 ^481^ | No dsm/icd/cut off scale criteria |
| 1. Singh 2014 ^482^ | No relevant outcome |
| 1. Slavutskaya 2018 ^483^ | No CHR-P population |
| 1. Smesny 2014 ^484^ | No relevant outcome |
| 1. Smesny 2015 ^485^ | No relevant outcome |
| 1. Smesny 2017 ^486^ | No relevant outcome |
| 1. Smieskova 2012 ^487^ | No relevant outcome |
| 1. Smieskova 2012 ^488^ | No relevant outcome |
| 1. Smieskova 2015 ^489^ | No relevant outcome |
| 1. Söder 2019 ^490^ | No CHR-P population |
| 1. Söder 2020 ^491^ | No CHR-P population |
| 1. Solé-Padullés 2017 ^492^ | No relevant outcome |
| 1. Solis-Vivanco 2014 ^493^ | No relevant outcome |
| 1. Sollychin 2019 ^494^ | No dsm/icd/cut off scale criteria |
| 1. Solmi 2020 ^495^ | No CHR-P population |
| 1. Solomon 2011 ^496^ | No relevant outcome |
| 1. Souaiby 2019 ^497^ | No relevant outcome |
| 1. Soyata 2018 ^498^ | No dsm/icd/cut off scale criteria |
| 1. Spauwen 2004 ^499^ | No CHR-P population |
| 1. Spauwen 2004 ^500^ | No CHR-P population |
| 1. Spauwen 2006 ^501^ | No CHR-P population |
| 1. Spencer 2007 ^502^ | No CHR-P population |
| 1. Spencer 2021 ^503^ | No relevant outcome |
| 1. Spitz 2017 ^504^ | No relevant outcome |
| Sprong 2008^505^ | No relevant outcome |
| 1. Srihari 2014 ^506^ | No CHR-P population |
| 1. Stanford 2011 ^507^ | No relevant outcome |
| 1. Stoddard 2010 ^508^ | No CHR-P population |
| 1. Stojanovic 2014 ^509^ | No relevant outcome |
| 1. Stone 2009 ^510^ | No dsm/icd/cut off scale criteria |
| 1. Stone 2010 ^511^ | No relevant outcome |
| 1. Stone 2012 ^512^ | No dsm/icd/cut off scale criteria |
| 1. Stone 2014 ^513^ | No relevant outcome |
| 1. Stowkowy 2012 ^514^ | No dsm/icd/cut off scale criteria |
| 1. Stowkowy 2013 ^515^ | No dsm/icd/cut off scale criteria |
| 1. Stowkowy 2013 ^516^ | No dsm/icd/cut off scale criteria |
| 1. Stowkowy 2015 ^517^ | No relevant outcome |
| 1. Stowkowy 2016 ^518^ | No relevant outcome |
| 1. Stowkowy 2016 ^519^ | No relevant outcome |
| 1. Stowkowy 2020 ^520^ | No CHR-P population |
| 1. Straub 2020 ^521^ | No CHR-P population |
| 1. Strauss 2018 ^522^ | Study Design |
| 1. Strauss 2021 ^523^ | No relevant outcome |
| 1. Strauss 2021 ^524^ | No relevant outcome |
| 1. Strelchuk 2020 ^525^ | Study Design |
| 1. Studerus 2018 ^526^ | No relevant outcome |
| 1. Studerus 2020 ^527^ | No relevant outcome |
| 1. Sugranyes 2012 ^528^ | No dsm/icd/cut off scale criteria |
| 1. Sullivan 2020 ^529^ | No CHR-P population |
| 1. Sumiyoshi 2000 ^530^ | No dsm/icd/cut off scale criteria |
| 1. Sumiyoshi 2013 ^531^ | No relevant outcome |
| 1. Sun 2009 ^532^ | No relevant outcome |
| 1. Suridjan 2013 ^533^ | No relevant outcome |
| 1. Svirskis 2005 ^534^ | No relevant outcome |
| 1. Svirskis 2007 ^535^ | No relevant outcome |
| 1. Szily 2009 ^536^ | No relevant outcome |
| 1. Tada 2016 ^537^ | No relevant outcome |
| 1. Tagore 2019 ^538^ | No relevant outcome |
| 1. Takahashi 2008 ^539^ | No relevant outcome |
| 1. Takahashi 2008 ^540^ | No relevant outcome |
| 1. Takahashi 2009 ^541^ | No relevant outcome |
| 1. Takahashi 2010 ^542^ | No relevant outcome |
| Takahashi 2013^543^ | No relevant outcome |
| 1. Takahashi 2014 ^544^ | No relevant outcome |
| 1. Takahashi 2018 ^545^ | No relevant outcome |
| 1. Takahashi 2019 ^546^ | No dsm/icd/cut off scale criteria |
| 1. Takano 2017 ^547^ | No relevant outcome |
| 1. Takayanagi 2017 ^548^ | No relevant outcome |
| 1. Tandon 2012 ^549^ | No CHR-P population |
| 1. Tang 2014 ^550^ | No relevant outcome |
| 1. Tang 2014 ^551^ | No relevant outcome |
| 1. Tang 2014 ^552^ | No relevant outcome |
| 1. Tang 2017 ^553^ | No CHR-P population |
| 1. Tang 2019 ^554^ | No relevant outcome |
| 1. Tapley Gasper 2001 ^555^ | No CHR-P population |
| 1. Tarbox 2013 ^556^ | No relevant outcome |
| 1. Tarbox 2014 ^557^ | No relevant outcome |
| 1. Tarbox-Berry 2018 ^558^ | No relevant outcome |
| 1. Tay 2015^559^ | Article not found |
| 1. Taylor 2013 ^560^ | No relevant outcome |
| 1. Taylor 2014 ^561^ | No relevant outcome |
| 1. Taylor 2014 ^562^ | No dsm/icd/cut off scale criteria |
| 1. Tessner 2011 ^563^ | No CHR-P population |
| 1. Theodoridou 2019 ^564^ | No dsm/icd/cut off scale criteria |
| 1. Therman 2009 ^565^ | No CHR-P population |
| 1. Therman 2011 ^566^ | No CHR-P population |
| 1. Therman 2014 ^567^ | No CHR-P population |
| 1. Thermenos 2016 ^568^ | No relevant outcome |
| 1. Thompson 2007 ^569^ | No relevant outcome |
| 1. Thompson 2009 ^570^ | No dsm/icd/cut off scale criteria |
| 1. Thompson 2012 ^571^ | No relevant outcome |
| 1. Thompson 2013 ^572^ | No dsm/icd/cut off scale criteria |
| 1. Thompson 2013 ^573^ | No relevant outcome |
| 1. Thompson 2013 ^574^ | No relevant outcome |
| 1. Thompson 2014 ^575^ | No dsm/icd/cut off scale criteria |
| 1. Thompson 2014 ^576^ | No dsm/icd/cut off scale criteria |
| 1. Thompson 2019 ^577^ | No relevant outcome |
| 1. Tijms 2015 ^578^ | No relevant outcome |
| 1. Tikka 2013 ^579^ | No relevant outcome |
| 1. Tikka 2020 ^580^ | Study Design |
| 1. Tognin 2014 ^581^ | No relevant outcome |
| 1. Tognin 2014 ^582^ | No relevant outcome |
| 1. Tognin 2020 ^583^ | No relevant outcome |
| 1. Tomyshev 2019 ^584^ | No dsm/icd/cut off scale criteria |
| 1. Torjesen 2015 ^585^ | Study Design |
| 1. Trotman 2014 ^586^ | No relevant outcome |
| 1. Tseng 2016 ^587^ | No relevant outcome |
| 1. Tso 2017 ^588^ | No relevant outcome |
| 1. Tsuji 2019 ^589^ | No relevant outcome |
| 1. Tsujino 2013 ^590^ | No relevant outcome |
| 1. Turetsky 2018 ^591^ | No relevant outcome |
| 1. Uchida 2014 ^592^ | No relevant outcome |
| 1. [Üçok](https://pubmed.ncbi.nlm.nih.gov/?term=%C3%9C%C3%A7ok+A&cauthor_id=24262680) 2013 ^593^ | No relevant outcome |
| 1. Üçok 2015 ^594^ | No relevant outcome |
| 1. Uhl 2011 ^595^ | No relevant outcome |
| 1. Uhlhaas 2017 ^596^ | No relevant outcome |
| 1. Urben 2012 ^597^ | No relevant outcome |
| 1. Uttinger 2018 ^598^ | No relevant outcome |
| 1. Uttinger 2018 ^599^ | No relevant outcome |
| 1. Uzdawinis 2010 ^600^ | Article not in English |
| 1. Vadhan 2017 ^601^ | No relevant outcome |
| 1. Vaever 2005 ^602^ | No CHR-P population |
| 1. Valdes-Florido 2021 ^603^ | No relevant outcome |
| 1. Valli 2011 ^604^ | No relevant outcome |
| 1. Valli 2016 ^605^ | No dsm/icd/cut off scale criteria |
| 1. Valli 2016 ^606^ | No relevant outcome |
| 1. Valmaggia 2007 ^607^ | No dsm/icd/cut off scale criteria |
| 1. Valmaggia 2013 ^608^ | No dsm/icd/cut off scale criteria |
| 1. Valmaggia 2014 ^609^ | No dsm/icd/cut off scale criteria |
| 1. Valmaggia 2015 ^610^ | No relevant outcome |
| 1. Valmaggia 2015 ^611^ | No relevant outcome |
| 1. van der Stelt 2005 ^612^ | No relevant outcome |
| 1. van der Velde 2015 ^613^ | No relevant outcome |
| 1. van Kampen 2006 ^614^ | No CHR-P population |
| 1. van Kampen 2009 ^615^ | No CHR-P population |
| 1. Van Os 1997 ^616^ | No CHR-P population |
| 1. van Rijn 2011 ^617^ | No relevant outcome |
| 1. van Rijn 2011 ^618^ | No relevant outcome |
| 1. van Tricht 2010 ^619^ | No relevant outcome |
| 1. van Tricht 2010 ^620^ | No relevant outcome |
| 1. van Tricht 2011 ^621^ | No CHR-P population |
| 1. van Tricht 2013 ^622^ | No relevant outcome |
| 1. van Tricht 2014 ^623^ | No relevant outcome |
| 1. van Tricht 2015 ^624^ | No relevant outcome |
| 1. Van Tricht 2015 ^625^ | No relevant outcome |
| 1. Vargas 2018 ^626^ | No dsm/icd/cut off scale criteria |
| 1. Vargas 2019 ^627^ | No relevant outcome |
| 1. Vargas 2019 ^628^ | No dsm/icd/cut off scale criteria |
| 1. Vasilyeva 2016 ^629^ | No relevant outcome |
| 1. Veijola 2013 ^630^ | No relevant outcome |
| 1. Veling 2016 ^631^ | No relevant outcome |
| 1. Veling 2016 ^632^ | No dsm/icd/cut off scale criteria |
| 1. Velthorst 2010 ^633^ | No relevant outcome |
| 1. Velthorst 2011 ^634^ | No relevant outcome |
| 1. Velthorst 2013 ^635^ | No relevant outcome |
| 1. Velthorst 2013 ^636^ | No dsm/icd/cut off scale criteria |
| 1. Velthorst 2018 ^637^ | No relevant outcome |
| 1. Velthorst 2019 ^638^ | No relevant outcome |
| 1. Ventura 2021 ^639^ | No relevant outcome |
| 1. Verma 2012 ^640^ | No relevant outcome |
| 1. Versmissen 2007 ^641^ | No CHR-P population |
| 1. Vingerhoets 2018 ^642^ | No relevant outcome |
| 1. Visser 2021 ^643^ | Study Design |
| 1. Vollmer-Larsen 2007 ^644^ | No dsm/icd/cut off scale criteria |
| 1. Waford 2015 ^645^ | No relevant outcome |
| 1. Waite 2018 ^646^ | No relevant outcome |
| 1. Waite 2020 ^647^ | No relevant outcome |
| 1. Wake 2021 ^648^ | No relevant outcome |
| 1. Walder 2008 ^649^ | No CHR-P population |
| 1. Walder 2013 ^650^ | No relevant outcome |
| 1. Walker 2009 ^651^ | No relevant outcome |
| 1. Walker 2013 ^652^ | No relevant outcome |
| 1. Walter 2012 ^653^ | No relevant outcome |
| 1. Walter 2015 ^654^ | No relevant outcome |
| 1. Walterfang 2008 ^655^ | No relevant outcome |
| 1. Wang 2015 ^656^ | No relevant outcome |
| 1. Wang 2016 ^657^ | No relevant outcome |
| 1. Wang 2016 ^658^ | No dsm/icd/cut off scale criteria |
| 1. Wang 2016 ^659^ | No relevant outcome |
| 1. Wang 2016 ^660^ | No relevant outcome |
| 1. Wang 2020 ^661^ | No CHR-P population |
| 1. Wang 2020 ^662^ | No relevant outcome |
| 1. Ward 2019 ^663^ | No dsm/icd/cut off scale criteria |
| 1. Warner 2002 ^664^ | Study Design |
| 1. Warnick 2005 ^665^ | No CHR-P population |
| 1. Washida 2013 ^666^ | No relevant outcome |
| 1. Watters 2018 ^667^ | No relevant outcome |
| 1. Weinberger 2018 ^668^ | No relevant outcome |
| 1. Weintraub 2019 ^669^ | No CHR-P population |
| 1. Welch 2011 ^670^ | No relevant outcome |
| 1. Welsh 2011 ^671^ | No CHR-P population |
| 1. Welsh 2012 ^672^ | Study Design |
| 1. Welsh 2014 ^673^ | No relevant outcome |
| 1. Welsh 2014 ^674^ | No relevant outcome |
| 1. Welsh 2015 ^675^ | No relevant outcome |
| 1. Wenneberg 2020 ^676^ | No relevant outcome |
| 1. Wenneberg 2020 ^677^ | No relevant outcome |
| 1. Werbeloff 2012 ^678^ | No CHR-P population |
| 1. Wessels 2019 ^679^ | No dsm/icd/cut off scale criteria |
| 1. Whalley 2016 ^680^ | No CHR-P population |
| 1. Whitford 2012 ^681^ | No relevant outcome |
| 1. Whitford 2018 ^682^ | No relevant outcome |
| 1. Whiting 2015 ^683^ | Study Design |
| 1. Wigman 2012 ^684^ | No CHR-P population |
| 1. Wijnen 2020 ^685^ | No relevant outcome |
| 1. Wilcox 2014 ^686^ | No dsm/icd/cut off scale criteria |
| 1. Willhite 2008 ^687^ | No relevant outcome |
| 1. Wilquin 2012 ^688^ | No relevant outcome |
| 1. Wilson 2019 ^689^ | No relevant outcome |
| 1. Wiltink 2015 ^690^ | No relevant outcome |
| 1. Winton Brown 2017 ^691^ | No relevant outcome |
| 1. Winton-Brown 2015 ^692^ | No relevant outcome |
| 1. Winton-Brown 2015 ^693^ | No relevant outcome |
| 1. Witthaus 2008 ^694^ | No relevant outcome |
| 1. Witthaus 2009 ^695^ | No relevant outcome |
| 1. Witthaus 2010 ^696^ | Study Design |
| 1. Witthaus 2010 ^697^ | No relevant outcome |
| 1. Wittman 1948 ^698^ | No CHR-P population |
| 1. Wolwer 2012 ^699^ | No relevant outcome |
| 1. Wong 2008 ^700^ | No dsm/icd/cut off scale criteria |
| 1. Wong 2009 ^701^ | No relevant outcome |
| 1. Wood 2003 ^702^ | No relevant outcome |
| 1. Wood 2003 ^703^ | No relevant outcome |
| 1. Wood 2005 ^704^ | No relevant outcome |
| 1. Wood 2010 ^705^ | No relevant outcome |
| 1. Woodberry 2009^706^ | Study Design |
| 1. Woodberry 2010 ^707^ | No relevant outcome |
| 1. Woodberry 2013 ^708^ | No relevant outcome |
| 1. Woodberry 2014 ^709^ | No relevant outcome |
| 1. Woods 2003^710^ | No dsm/icd/cut off scale criteria |
| 1. Woods 2007 ^711^ | No relevant outcome |
| 1. Woods 2013 ^712^ | No relevant outcome |
| 1. Woods 2018 ^713^ | No relevant outcome |
| 1. Worland 1979 ^714^ | Study Design |
| 1. Worthington 2021 ^715^ | No relevant outcome |
| 1. Wotruba 2014 ^716^ | No dsm/icd/cut off scale criteria |
| 1. Wotruba 2014 ^717^ | No relevant outcome |
| 1. Wu 2014 ^718^ | Study Design |
| 1. Wu 2021 ^719^ | No relevant outcome |
| 1. Xu 2016 ^720^ | No relevant outcome |
| 1. Xu 2016 ^721^ | No relevant outcome |
| 1. Xu 2018 ^722^ | No relevant outcome |
| 1. Yaakub 2013 ^723^ | No relevant outcome |
| 1. Yang 2015 ^724^ | No dsm/icd/cut off scale criteria |
| 1. Yang 2018 ^725^ | No dsm/icd/cut off scale criteria |
| 1. Yassin 2020 ^726^ | No relevant outcome |
| 1. Yee 2010 ^727^ | No relevant outcome |
| 1. Yee 2020 ^728^ | No relevant outcome |
| 1. Yiend 2019 ^729^ | No dsm/icd/cut off scale criteria |
| 1. Yong 2014 ^730^ | No dsm/icd/cut off scale criteria |
| 1. Yoon 2015 ^731^ | No relevant outcome |
| 1. Yuen 2018 ^732^ | No dsm/icd/cut off scale criteria |
| 1. Yun 2005 ^733^ | No relevant outcome |
| 1. Yung 1998 ^734^ | No dsm/icd/cut off scale criteria |
| 1. Yung 1998 ^735^ | No relevant outcome |
| 1. Yung 2004 ^736^ | No dsm/icd/cut off scale criteria |
| 1. Yung 2005 ^737^ | No relevant outcome |
| 1. Yung 2007 ^738^ | Study Design |
| 1. Yung 2008 ^739^ | No relevant outcome |
| 1. Yung 2011 ^740^ | No dsm/icd/cut off scale criteria |
| 1. Yung 2012 ^741^ | Study Design |
| 1. Yung 2015 ^742^ | No relevant outcome |
| 1. Yung 2019 ^743^ | No relevant outcome |
| 1. Zammit 2002 ^744^ | No CHR-P population |
| 1. Zammit 2011 ^745^ | No CHR-P population |
| 1. Zanini 2015 ^746^ | No relevant outcome |
| 1. Zeni-Graiff 2016 ^747^ | No relevant outcome |
| 1. Zeni-Graiff 2019 ^748^ | No dsm/icd/cut off scale criteria |
| 1. Zhan 2017 ^749^ | No dsm/icd/cut off scale criteria |
| 1. Zhang 2014 ^750^ | No dsm/icd/cut off scale criteria |
| 1. Zhang 2015 ^751^ | No relevant outcome |
| 1. Zhang 2016 ^752^ | No relevant outcome |
| 1. Zhang 2016 ^753^ | No relevant outcome |
| 1. Zhang 2016 ^754^ | No relevant outcome |
| 1. Zhang 2017 ^755^ | No relevant outcome |
| 1. Zhang 2017^756^ | Article not in English |
| 1. Zhang 2018 ^757^ | No relevant outcome |
| 1. Zhang 2018 ^758^ | No relevant outcome |
| 1. Zhang 2018 ^759^ | No relevant outcome |
| 1. Zhang 2018 ^760^ | No relevant outcome |
| 1. Zhang 2018 ^761^ | No dsm/icd/cut off scale criteria |
| 1. Zhang 2018 ^762^ | No CHR-P population |
| 1. Zhang 2018 ^763^ | No relevant outcome |
| 1. Zhang 2019 ^764^ | No relevant outcome |
| 1. Zhang 2019 ^765^ | No relevant outcome |
| 1. Zhang 2020 ^766^ | No relevant outcome |
| 1. Zhang 2020 ^767^ | No relevant outcome |
| 1. Zhang 2021 ^768^ | No relevant outcome |
| 1. Zhang 2021 ^769^ | No relevant outcome |
| 1. Zhang 2021 ^770^ | No relevant outcome |
| 1. Zhao 2018 ^771^ | No relevant outcome |
| 1. Zhu 2019 ^772^ | No relevant outcome |
| 1. Ziermans 2011 ^773^ | No relevant outcome |
| 1. Ziermans 2011 ^774^ | No relevant outcome |
| 1. Ziermans 2012 ^775^ | No relevant outcome |
| 1. Ziermans 2012 ^776^ | No relevant outcome |
| 1. Ziermans 2014 ^777^ | No relevant outcome |
| 1. Zimbron 2013 ^778^ | No relevant outcome |
| 1. Zimmermann 2010 ^779^ | No relevant outcome |
| 1. Zipursky 2002 ^780^ | No CHR-P population |
| 1. Zoghbi 2019 ^781^ | No CHR-P population |
| 1. Zolkowska 2001 ^782^ | No CHR-P population |
| 1. Zugman 2013 ^783^ | Study Design |
| 1. Zuschlag 2018 ^784^ | No CHR-P population |

1. MacCabe JH, Wicks S, Löfving S, et al. Decline in Cognitive Performance Between Ages 13 and 18 Years and the Risk for Psychosis in Adulthood. *JAMA Psychiatry*. 2013;70(3):261. doi:10.1001/2013.jamapsychiatry.43

2. Madeira L, Bonoldi I, Rocchetti M, et al. Prevalence and implications of Truman symptoms in subjects at ultra high risk for psychosis. *Psychiatry Research*. 2016;238:270-276. doi:10.1016/j.psychres.2016.02.001

3. Madeira L, Bonoldi I, Rocchetti M, et al. An initial investigation of abnormal bodily phenomena in subjects at ultra high risk for psychosis: Their prevalence and clinical implications. *Comprehensive Psychiatry*. 2016;66:39-45. doi:10.1016/j.comppsych.2015.12.005

4. Magaud E, Kebir O, Gut A, et al. Altered semantic but not phonological verbal fluency in young help-seeking individuals with ultra high risk of psychosis. *Schizophrenia Research*. 2010;123(1):53-58. doi:10.1016/j.schres.2010.05.005

5. Magaud E, Nyman K, Addington J. Cyberbullying in those at clinical high risk for psychosis. *Early Intervention in Psychiatry*. 2013;7(4):427-430. doi:10.1111/eip.12013

6. Magaud E, Morvan Y, Rampazzo A, et al. Subjects at Ultra High Risk for psychosis have ‘heterogeneous’ intellectual functioning profile: A multiple-case study. *Schizophrenia Research*. 2014;152(2-3):415-420. doi:10.1016/j.schres.2013.11.002

7. Maggini C, Raballo A. Subjective Experience of Schizotropic Vulnerability in Siblings of Schizophrenics. *Psychopathology*. 2004;37(1). doi:10.1159/000077016

8. Malla A, de Bonneville M, Shah J, et al. Outcome in patients converting to psychosis following a treated clinical high risk state. *Early Intervention in Psychiatry*. 2018;12(4):715-719. doi:10.1111/eip.12431

9. Malm U. Depot combined with oral antipsychotic medication in schizophrenia: A parachute for the patient in prodromal phase. *Nordic Journal of Psychiatry*. 1995;49(sup35):29-30. doi:10.3109/08039489509099717

10. Mamah D, Mbwayo A, Mutiso V, et al. A survey of psychosis risk symptoms in Kenya. *Comprehensive Psychiatry*. 2012;53(5):516-524. doi:10.1016/j.comppsych.2011.08.003

11. Mamah D, Striley CW, Ndetei DM, et al. Knowledge of psychiatric terms and concepts among Kenyan youth: Analysis of focus group discussions. *Transcultural Psychiatry*. 2013;50(4):515-531. doi:10.1177/1363461513499809

12. Mamah D, Owoso A, Sheffield JM, Bayer C. The WERCAP Screen and the WERC Stress Screen: Psychometrics of self-rated instruments for assessing bipolar and psychotic disorder risk and perceived stress burden. *Comprehensive Psychiatry*. 2014;55(7):1757-1771. doi:10.1016/j.comppsych.2014.07.004

13. Mamah D, Musau A, Mutiso VN, et al. Characterizing psychosis risk traits in Africa: A longitudinal study of Kenyan adolescents. *Schizophrenia Research*. 2016;176(2-3):340-348. doi:10.1016/j.schres.2016.08.004

14. Mantere O, Saarela M, Kieseppä T, et al. Anti-neuronal anti-bodies in patients with early psychosis. *Schizophrenia Research*. 2018;192:404-407. doi:10.1016/j.schres.2017.04.027

15. Manzanares N, Monseny R, Ortega L, et al. Unhealthy lifestyle in early psychoses: The role of life stress and the hypothalamic–pituitary–adrenal axis. *Psychoneuroendocrinology*. 2014;39:1-10. doi:10.1016/j.psyneuen.2013.09.023

16. Mariné R, Creus M, Solé M, et al. Clinical correlates of obsessive-compulsive symptom dimensions in at-risk mental states and psychotic disorders at early stages. *Psychiatry Research*. 2015;228(3):363-367. doi:10.1016/j.psychres.2015.05.083

17. Markulev C, McGorry PD, Nelson B, et al. NEURAPRO-E study protocol: a multicentre randomized controlled trial of omega-3 fatty acids and cognitive-behavioural case management for patients at ultra high risk of schizophrenia and other psychotic disorders. *Early Intervention in Psychiatry*. 2017;11(5):418-428. doi:10.1111/eip.12260

18. Marques TR, Smith S, Bonaccorso S, et al. Sexual dysfunction in people with prodromal or first-episode psychosis. *British Journal of Psychiatry*. 2012;201(2):131-136. doi:10.1192/bjp.bp.111.101220

19. Marshall C, Deighton S, Cadenhead KS, et al. The Violent Content in Attenuated Psychotic Symptoms. *Psychiatry Research*. 2016;242:61-66. doi:10.1016/j.psychres.2016.05.005

20. Marshall C, Lu Y, Lyngberg K, et al. Changes in symptom content from a clinical high‐risk state to conversion to psychosis. *Early Intervention in Psychiatry*. 2019;13(2):257-263. doi:10.1111/eip.12473

21. Martínez A, Gaspar PA, Hillyard SA, et al. Impaired Motion Processing in Schizophrenia and the Attenuated Psychosis Syndrome: Etiological and Clinical Implications. *American Journal of Psychiatry*. 2018;175(12):1243-1254. doi:10.1176/appi.ajp.2018.18010072

22. Martorell L, Muntané G, Porta-López S, et al. Increased levels of serum leptin in the early stages of psychosis. *Journal of Psychiatric Research*. 2019;111:24-29. doi:10.1016/j.jpsychires.2019.01.006

23. Marulanda S, Addington J. Resilience in individuals at clinical high risk for psychosis. *Early Intervention in Psychiatry*. 2016;10(3):212-219. doi:10.1111/eip.12174

24. Marvin SE, Miklowitz DJ, O’Brien MP, Cannon TD. Family-focused therapy for individuals at clinical high risk for psychosis: treatment fidelity within a multisite randomized trial. *Early Intervention in Psychiatry*. 2016;10(2):137-143. doi:10.1111/eip.12144

25. Masaoka Y, Velakoulis D, Brewer WJ, et al. Impaired olfactory ability associated with larger left hippocampus and rectus volumes at earliest stages of schizophrenia: A sign of neuroinflammation? *Psychiatry Research*. 2020;289. doi:10.1016/j.psychres.2020.112909

26. Masillo A, Day F, Laing J, et al. Interpersonal sensitivity in the at-risk mental state for psychosis. *Psychological Medicine*. 2012;42(9):1835-1845. doi:10.1017/S0033291711002996

27. Masillo A, Valmaggia LR, Saba R, et al. Interpersonal sensitivity and functioning impairment in youth at ultra-high risk for psychosis. *European Child & Adolescent Psychiatry*. 2016;25(1):7-16. doi:10.1007/s00787-015-0692-6

28. Masillo A, Brandizzi M, Nelson B, et al. Youth mental health services in Italy: An achievable dream? *Early Intervention in Psychiatry*. 2018;12(3):433-443. doi:10.1111/eip.12328

29. Masillo A, Brandizzi M, Valmaggia LR, et al. Interpersonal sensitivity and persistent attenuated psychotic symptoms in adolescence. *European Child & Adolescent Psychiatry*. 2018;27(3):309-318. doi:10.1007/s00787-017-1047-2

30. Masillo A, Valmaggia LR, Saba R, et al. Interpersonal sensitivity, bullying victimization and paranoid ideation among help-seeking adolescents and young adults. *Early Intervention in Psychiatry*. 2019;13(1):57-63. doi:10.1111/eip.12447

31. Mason OJ, Booth H, Olivers C. Proneness to psychosis and selection of objects of visual attention: Individual differences in visual marking. *Personality and Individual Differences*. 2004;36(8):1771-1779. doi:10.1016/j.paid.2003.07.015

32. Masucci MD, Lister A, Corcoran CM, Brucato G, Girgis RR. Motor Dysfunction as a Risk Factor for Conversion to Psychosis Independent of Medication Use in a Psychosis-Risk Cohort. *Journal of Nervous & Mental Disease*. 2018;206(5):356-361. doi:10.1097/NMD.0000000000000806

33. Mathalon DH, Roach BJ, Ferri JM, et al. Deficient auditory predictive coding during vocalization in the psychosis risk syndrome and in early illness schizophrenia: the final expanded sample. *Psychological Medicine*. 2019;49(11):1897-1904. doi:10.1017/S0033291718002659

34. Matsumoto K, Ohmuro N, Tsujino N, et al. Open-label study of cognitive behavioural therapy for individuals with at-risk mental state: Feasibility in the Japanese clinical setting. *Early Intervention in Psychiatry*. 2019;13(1):137-141. doi:10.1111/eip.12541

35. Maurya PK, Rizzo LB, Xavier G, et al. Shorter leukocyte telomere length in patients at ultra high risk for psychosis. *European Neuropsychopharmacology*. 2017;27(5):538-542. doi:10.1016/j.euroneuro.2017.02.008

36. Mayeli A, LaGoy A, Donati FL, Kaskie RE, Najibi SM, Ferrarelli F. Sleep abnormalities in individuals at clinical high risk for psychosis. *Journal of Psychiatric Research*. 2021;137. doi:10.1016/j.jpsychires.2021.03.006

37. McDonnell J, Stahl D, Day F, McGuire P, Valmaggia LR. Interpersonal sensitivity in those at clinical high risk for psychosis mediates the association between childhood bullying victimisation and paranoid ideation: A virtual reality study. *Schizophrenia Research*. 2018;192:89-95. doi:10.1016/j.schres.2017.04.029

38. McFarlane W, Cook W, Downing D, Verdi M, Woodberry K, Ruff A. Portland Identification and Early Referral: A Community-Based System for Identifying and Treating Youths at High Risk of Psychosis. *Psychiatric Services*. 2010;61(5). doi:10.1176/appi.ps.61.5.512

39. McFarlane WR, Susser E, McCleary R, et al. Reduction in Incidence of Hospitalizations for Psychotic Episodes Through Early Identification and Intervention. *Psychiatric Services*. 2014;65(10):1194-1200. doi:10.1176/appi.ps.201300336

40. McGlashan TH, Zipursky RB, Perkins D, et al. The PRIME North America randomized double-blind clinical trial of olanzapine versus placebo in patients at risk of being prodromally symptomatic for psychosis. *Schizophrenia Research*. 2003;61(1):7-18. doi:10.1016/S0920-9964(02)00439-5

41. McGlashan TH, Zipursky RB, Perkins D, et al. *Randomized, Double-Blind Trial of Olanzapine Versus Placebo in Patients Prodromally Symptomatic for Psychosis*. Vol 163.; 2006.

42. McGorry PD, McFarlane C, Patton GC, et al. The prevalence of prodromal features of schizophrenia in adolescence: a preliminary survey. *Acta Psychiatrica Scandinavica*. 1995;92(4):241-249. doi:10.1111/j.1600-0447.1995.tb09577.x

43. McGorry PD, Yung AR, Phillips LJ, et al. Randomized Controlled Trial of Interventions Designed to Reduce the Risk of Progression to First-Episode Psychosis in a Clinical Sample With Subthreshold Symptoms. *Archives of General Psychiatry*. 2002;59(10):921. doi:10.1001/archpsyc.59.10.921

44. McGorry PD, Yung AR, Phillips LJ. “Closing in”: What features predict the onset of first-episode psychosis within an ultra-high-risk group? In: Zipursky RB, Schulz SC, eds. *The Early Stages of Schizophrenia.* American Psychiatric Publishing, Inc.; 2002:3-31.

45. McGorry PD, Nelson B, Phillips LJ, et al. Randomized Controlled Trial of Interventions for Young People at Ultra-High Risk of Psychosis. *The Journal of Clinical Psychiatry*. 2013;74(04):349-356. doi:10.4088/JCP.12m07785

46. McGorry PD, Nelson B, Markulev C, et al. Effect of ω-3 Polyunsaturated Fatty Acids in Young People at Ultrahigh Risk for Psychotic Disorders. *JAMA Psychiatry*. 2017;74(1):19. doi:10.1001/jamapsychiatry.2016.2902

47. McGuire P. Clinical and research work in people with prodromal signs of psychosis. *Eur Psychiatry*. 2009;24(4)(I-II).

48. McGuire P, Selvaraj S, Howes O. Is clinical intervention in the ultra high risk phase effective? *Revista Brasileira de Psiquiatria*. 2011;33(suppl 2):s161-s174. doi:10.1590/S1516-44462011000600004

49. McHugh MJ, McGorry PD, Yung AR, et al. Cannabis-induced attenuated psychotic symptoms: implications for prognosis in young people at ultra-high risk for psychosis. *Psychological Medicine*. 2017;47(4):616-626. doi:10.1017/S0033291716002671

50. McHugh MJ, McGorry PD, Yuen HP, et al. The Ultra-High-Risk for psychosis groups: Evidence to maintain the status quo. *Schizophrenia Research*. 2018;195:543-548. doi:10.1016/j.schres.2017.09.003

51. McIntosh AM, Baig BJ, Hall J, et al. Relationship of Catechol-O-Methyltransferase Variants to Brain Structure and Function in a Population at High Risk of Psychosis. *Biological Psychiatry*. 2007;61(10):1127-1134. doi:10.1016/j.biopsych.2006.05.020

52. McKechanie AG, Moorhead TWJ, Stanfield AC, et al. Negative symptoms and longitudinal grey matter tissue loss in adolescents at risk of psychosis: Preliminary findings from a 6-year follow-up study. *British Journal of Psychiatry*. 2016;208(6):565-570. doi:10.1192/bjp.bp.114.154526

53. McLaughlin D, Carrión RE, Auther AM, et al. Functional Capacity Assessed by the Map Task in Individuals at Clinical High-Risk for Psychosis. *Schizophrenia Bulletin*. 2016;42(5):1234-1242. doi:10.1093/schbul/sbw039

54. McNeil TF, Schubert EW, Cantor-Graae E, Brossner M, Schubert P, Henriksson KM. Unwanted pregnancy as a risk factor for offspring schizophrenia-spectrum and affective disorders in adulthood: a prospective high-risk study. *Psychological Medicine*. 2009;39(6):957-965. doi:10.1017/S0033291708004479

55. Mechelli A, Riecher-Rössler A, Meisenzahl EM, et al. Neuroanatomical Abnormalities That Predate the Onset of Psychosis. *Archives of General Psychiatry*. 2011;68(5):489. doi:10.1001/archgenpsychiatry.2011.42

56. Mechelli A, Lin A, Wood S, et al. Using clinical information to make individualized prognostic predictions in people at ultra high risk for psychosis. *Schizophrenia Research*. 2017;184:32-38. doi:10.1016/j.schres.2016.11.047

57. Meijer JH, Schmitz N, Nieman DH, et al. Semantic fluency deficits and reduced grey matter before transition to psychosis: A voxelwise correlational analysis. *Psychiatry Research: Neuroimaging*. 2011;194(1):1-6. doi:10.1016/j.pscychresns.2011.01.004

58. Meisenzahl EM, Koutsouleris N, Gaser C, et al. Structural brain alterations in subjects at high-risk of psychosis: A voxel-based morphometric study. *Schizophrenia Research*. 2008;102(1-3):150-162. doi:10.1016/j.schres.2008.02.023

59. Melton RP, Dykeman C. Family Aided Community Treatment for the Treatment of Early Psychosis: A Proof of Concept Study. *Community Mental Health Journal*. 2016;52(6):623-630. doi:10.1007/s10597-015-9984-z

60. Meneghelli A, Breda A, Caprin C, Patelli G, Cocchi A. L’intervento precoce nelle psicosi: strategie di valutazione. Psicoterapia cognitiva e comportamentale. 2003;9(1):17-30.

61. Meneghelli A, Alpi A, Pafumi N, Patelli G, Preti A, Cocchi A. Expressed emotion in first-episode schizophrenia and in ultra high-risk patients: Results from the Programma2000 (Milan, Italy). *Psychiatry Research*. 2011;189(3):331-338. doi:10.1016/j.psychres.2011.03.021

62. Meneghelli A, Alpi A, Cascio M, et al. Italian validation of the “Early Recognition Inventory for the retrospective assessment of the Onset of Schizophrenia Checklist”: Reliability, validity and instructions for use. *Journal of psychopathology*. 2014;20:186-198.

63. Meng H, Graf Schimmelmann B, Koch E, et al. Basic symptoms in the general population and in psychotic and non-psychotic psychiatric adolescents. *Schizophrenia Research*. 2009;111(1-3):32-38. doi:10.1016/j.schres.2009.03.001

64. Mennigen E, Miller RL, Rashid B, et al. Reduced higher-dimensional resting state fMRI dynamism in clinical high-risk individuals for schizophrenia identified by meta-state analysis. *Schizophrenia Research*. 2018;201:217-223. doi:10.1016/j.schres.2018.06.007

65. Mennigen E, Fryer SL, Rashid B, et al. Transient Patterns of Functional Dysconnectivity in Clinical High Risk and Early Illness Schizophrenia Individuals Compared with Healthy Controls. *Brain Connectivity*. 2019;9(1):60-76. doi:10.1089/brain.2018.0579

66. Menschikov PE, Semenova NA, Ublinskiy M v., et al. 1H-MRS and MEGA-PRESS pulse sequence in the study of balance of inhibitory and excitatory neurotransmitters in the human brain of ultra-high risk of schizophrenia patients. *Doklady Biochemistry and Biophysics*. 2016;468(1):168-172. doi:10.1134/S1607672916030029

67. Metzler S, Dvorsky D, Wyss C, et al. Neurocognition in help-seeking individuals at risk for psychosis: Prediction of outcome after 24 months. *Psychiatry Research*. 2016;246:188-194. doi:10.1016/j.psychres.2016.08.065

68. Meyer EC, Carrion RE, Cornblatt BA, et al. The Relationship of Neurocognition and Negative Symptoms to Social and Role Functioning Over Time in Individuals at Clinical High Risk in the First Phase of the North American Prodrome Longitudinal Study. *Schizophrenia Bulletin*. 2014;40(6):1452-1461. doi:10.1093/schbul/sbt235

69. Michel C, Schimmelmann BG, Kupferschmid S, Siegwart M, Schultze-Lutter F. Reliability of telephone assessments of at-risk criteria of psychosis: A comparison to face-to-face interviews. *Schizophrenia Research*. 2014;153(1-3):251-253. doi:10.1016/j.schres.2014.01.025

70. Michel C, Kutschal C, Schimmelmann BG, Schultze-Lutter F. Convergent and concurrent validity of the Frankfurt Complaint Questionnaire as a screener for psychosis risk. *Journal of Risk Research*. 2017;20(11):1480-1496. doi:10.1080/13669877.2016.1179209

71. Michel C, Schmidt SJ, Schnyder N, et al. Associations of psychosis-risk symptoms with quality of life and self-rated health in the Community. *European Psychiatry*. 2019;62:116-123. doi:10.1016/j.eurpsy.2019.08.008

72. Michels Franziska, Schilling Claudia, Rausch Franziska, et al. Nightmare frequency in schizophrenic patients, healthy relatives of schizophrenic patients, patients at high risk states for psychosis, and healthy controls. *International Journal of Dream Research*. 2014;7.

73. Miettunen J, Törmänen S, Murray GK, et al. Association of cannabis use with prodromal symptoms of psychosis in adolescence. *British Journal of Psychiatry*. 2008;192(6):470-471. doi:10.1192/bjp.bp.107.045740

74. Miklowitz DJ, O’Brien MP, Schlosser DA, et al. Family-Focused Treatment for Adolescents and Young Adults at High Risk for Psychosis: Results of a Randomized Trial. *Journal of the American Academy of Child & Adolescent Psychiatry*. 2014;53(8):848-858. doi:10.1016/j.jaac.2014.04.020

75. Miller TJ, McGlashan TH, Woods SW, et al. Symptom assessment in schizophrenic prodromal states. *Psychiatric Quarterly*. 1999;70(4):273-287. doi:10.1023/A:1022034115078

76. Miller TJ, McGlashan TH, Rosen JL, et al. Prospective Diagnosis of the Initial Prodrome for Schizophrenia Based on the Structured Interview for Prodromal Syndromes: Preliminary Evidence of Interrater Reliability and Predictive Validity. *American Journal of Psychiatry*. 2002;159(5):863-865. doi:10.1176/appi.ajp.159.5.863

77. Miller TJ, Zipursky RB, Perkins D, et al. The PRIME North America randomized double-blind clinical trial of olanzapine versus placebo in patients at risk of being prodromally symptomatic for psychosis. *Schizophrenia Research*. 2003;61(1):19-30. doi:10.1016/S0920-9964(02)00440-1

78. Millman ZB, Goss J, Schiffman J, Mejias J, Gupta T, Mittal VA. Mismatch and lexical retrieval gestures are associated with visual information processing, verbal production, and symptomatology in youth at high risk for psychosis. *Schizophrenia Research*. 2014;158(1-3):64-68. doi:10.1016/j.schres.2014.06.007

79. Millman ZB. *Facial Emotion Recognition in Attenuated Psychosis, Depressive, and Anxiety Syndromes: Are Impairments Common or Specific?* Diss. University of Maryland; 2020.

80. Mills JG, Fusar-Poli P, Morgan C, Azis M, McGuire P. People meeting ultra high risk for psychosis criteria in the community. *World Psychiatry*. 2017;16(3):322-323. doi:10.1002/wps.20463

81. Minichino A, Francesconi M, Carrión RE, et al. From neurological soft signs to functional outcome in young individuals in treatment with secondary services for non-psychotic disorders: a path analysis. *Psychological Medicine*. 2017;47(7):1192-1203. doi:10.1017/S0033291716003056

82. Minichino A, Rutigliano G, Merlino S, et al. Unmet needs in patients with brief psychotic disorders: Too ill for clinical high risk services and not ill enough for first episode services. *European Psychiatry*. 2019;57:26-32. doi:10.1016/j.eurpsy.2018.12.006

83. Miron JP, Abdel-Baki A. Cognitive–behavioural therapy can prevent transition to psychosis in ultra-high-risk participants in the long term. *Evidence Based Mental Health*. 2017;20(2):e10-e10. doi:10.1136/eb-2016-102569

84. Mirzakhanian H, Singh F, Seeber K, Shafer KM, Cadenhead KS. A developmental look at the attentional system in the at risk and first episode of psychosis: Age related changes in attention along the psychosis spectrum. *Cognitive Neuropsychiatry*. 2013;18(1-2):26-43. doi:10.1080/13546805.2012.713770

85. Mittal VA, Walker EF. Movement abnormalities predict conversion to Axis I psychosis among prodromal adolescents. *Journal of Abnormal Psychology*. 2007;116(4):796-803. doi:10.1037/0021-843X.116.4.796

86. Mittal VA, Saczawa ME, Walder D, Willhite R, Walker EF. *Prenatal Exposure to Viral Infection and Conversion among Adolescents at High-Risk for Psychotic Disorders*.

87. Mittal VA, Neumann C, Saczawa M, Walker EF. Longitudinal Progression of Movement Abnormalities in Relation to Psychotic Symptoms in Adolescents at High Risk of Schizophrenia. *Archives of General Psychiatry*. 2008;65(2):165. doi:10.1001/archgenpsychiatry.2007.23

88. Mittal VA, Willhite R, Daley M, et al. Obstetric complications and risk for conversion to psychosis among individuals at high clinical risk. *Early Intervention in Psychiatry*. 2009;3(3):226-230. doi:10.1111/j.1751-7893.2009.00137.x

89. Mittal VA. Movement abnormalities in adolescents at-risk for psychosis. *Dissertation Abstracts International: Section B: The Sciences and Engineering*. 2009;69(10-B):6425.

90. Mittal VA, Daley M, Shiode MF, Bearden CE, O’Neill J, Cannon TD. Striatal volumes and dyskinetic movements in youth at high-risk for psychosis. *Schizophrenia Research*. 2010;123(1):68-70. doi:10.1016/j.schres.2010.08.002

91. Mittal VA, Jalbrzikowski M, Daley M, Roman C, Bearden CE, Cannon TD. Abnormal movements are associated with poor psychosocial functioning in adolescents at high risk for psychosis. *Schizophrenia Research*. 2011;130(1-3):164-169. doi:10.1016/j.schres.2011.05.007

92. Mittal VA, Walker EF. Minor physical anomalies and vulnerability in prodromal youth. *Schizophrenia Research*. 2011;129(2-3):116-121. doi:10.1016/j.schres.2011.02.022

93. Mittal VA, Dean DJ, Pelletier A. Dermatoglyphic asymmetries and fronto-striatal dysfunction in young adults reporting non-clinical psychosis. *Acta Psychiatrica Scandinavica*. 2012;126(4):290-297. doi:10.1111/j.1600-0447.2012.01869.x

94. Mittal VA, Gupta T, Orr JM, et al. Physical activity level and medial temporal health in youth at ultra high-risk for psychosis. *Journal of Abnormal Psychology*. 2013;122(4):1101-1110. doi:10.1037/a0034085

95. Mittal VA, Dean DJ, Pelletier A. Internet addiction, reality substitution and longitudinal changes in psychotic-like experiences in young adults. *Early Intervention in Psychiatry*. 2013;7(3):261-269. doi:10.1111/j.1751-7893.2012.00390.x

96. Mittal VA, Dean DJ, Bernard JA, et al. Neurological Soft Signs Predict Abnormal Cerebellar-Thalamic Tract Development and Negative Symptoms in Adolescents at High Risk for Psychosis: A Longitudinal Perspective. *Schizophrenia Bulletin*. 2014;40(6):1204-1215. doi:10.1093/schbul/sbt199

97. Mittal VA, Gupta T, Keane BP, Silverstein SM. Visual context processing dysfunctions in youth at high risk for psychosis: Resistance to the Ebbinghaus illusion and its symptom and social and role functioning correlates. *Journal of Abnormal Psychology*. 2015;124(4):953-960. doi:10.1037/abn0000082

98. Mitter N, Nah GQR, Bong YL, Lee J, Chong SA. Longitudinal Youth-At-Risk Study (LYRIKS): outreach strategies based on a community-engaged framework. *Early Intervention in Psychiatry*. 2014;8(3):298-303. doi:10.1111/eip.12049

99. Miyakoshi T, Matsumoto K, Ito F, Ohmuro N, Matsuoka H. Application of the Comprehensive Assessment of At-Risk Mental States (CAARMS) to the Japanese population: reliability and validity of the Japanese version of the CAARMS. *Early Intervention in Psychiatry*. 2009;3(2):123-130. doi:10.1111/j.1751-7893.2009.00118.x

100. Mizrahi R, Addington J, Rusjan PM, et al. Increased Stress-Induced Dopamine Release in Psychosis. *Biological Psychiatry*. 2012;71(6):561-567. doi:10.1016/j.biopsych.2011.10.009

101. Mizrahi R, Kenk M, Suridjan I, et al. Stress-Induced Dopamine Response in Subjects at Clinical High Risk for Schizophrenia with and without Concurrent Cannabis Use. *Neuropsychopharmacology*. 2014;39(6):1479-1489. doi:10.1038/npp.2013.347

102. Modinos G, Aleman A, Ormel J. Cortisol Levels in Childhood and Psychosis Risk in late Adolescence. *Journal of the American Academy of Child & Adolescent Psychiatry*. 2009;48(7). doi:10.1097/CHI.0b013e3181a5e3ff

103. Modinos G, Mechelli A, Ormel J, Groenewold NA, Aleman A, McGuire PK. Schizotypy and brain structure: A voxel-based morphometry study. *Psychological Medicine*. 2010;40(9):1423-1431. doi:10.1017/S0033291709991875

104. Modinos G, Pettersson-Yeo W, Allen P, McGuire PK, Aleman A, Mechelli A. Multivariate pattern classification reveals differential brain activation during emotional processing in individuals with psychosis proneness. *Neuroimage*. 2012;59(3):3033-3041. doi:10.1016/j.neuroimage.2011.10.048

105. Modinos G, Tseng HH, Falkenberg I, Samson C, McGuire P, Allen P. Neural correlates of aberrant emotional salience predict psychotic symptoms and global functioning in high-risk and first-episode psychosis. *Social Cognitive and Affective Neuroscience*. 2015;10(10):1429-1436. doi:10.1093/scan/nsv035

106. Modinos G, Şimşek F, Horder J, et al. Cortical GABA in Subjects at Ultra-High Risk of Psychosis: Relationship to Negative Prodromal Symptoms. *International Journal of Neuropsychopharmacology*. 2018;21(2):114-119. doi:10.1093/ijnp/pyx076

107. Modinos G, Kempton MJ, Tognin S, et al. Association of Adverse Outcomes With Emotion Processing and Its Neural Substrate in Individuals at Clinical High Risk for Psychosis. *JAMA Psychiatry*. 2020;77(2):190. doi:10.1001/jamapsychiatry.2019.3501

108. Mondragón-Maya A, Solís-Vivanco R, León-Ortiz P, et al. Reduced P3a amplitudes in antipsychotic naïve first-episode psychosis patients and individuals at clinical high-risk for psychosis. *Journal of Psychiatric Research*. 2013;47(6):755-761. doi:10.1016/j.jpsychires.2012.12.017

109. Mondrup L, Rosenbaum B. Interpersonal problems in the prodromal state of schizophrenia: An exploratory study. *Psychosis*. 2010;2(3):238-247. doi:10.1080/17522430903288340

110. Montagnese M, Knolle F, Haarsma J, et al. Reinforcement learning as an intermediate phenotype in psychosis? Deficits sensitive to illness stage but not associated with polygenic risk of schizophrenia in the general population. *Schizophrenia Research*. 2020;222:389-396. doi:10.1016/j.schres.2020.04.022

111. Montalvo I, Gutiérrez-Zotes A, Creus M, et al. Increased Prolactin Levels Are Associated with Impaired Processing Speed in Subjects with Early Psychosis. *PLoS ONE*. 2014;9(2):e89428. doi:10.1371/journal.pone.0089428

112. Montojo CA, Ibrahim A, Karlsgodt KH, et al. Disrupted working memory circuitry and psychotic symptoms in 22q11.2 deletion syndrome. *NeuroImage: Clinical*. 2014;4:392-402. doi:10.1016/j.nicl.2014.01.010

113. Montreuil TC, Malla AK, Joober R, Bélanger C, Myhr G, Lepage M. Manualized Group Cognitive-Behavioral Therapy for Social Anxiety in At-Risk Mental State and First Episode Psychosis: A Pilot Study of Feasibility and Outcomes. *International Journal of Group Psychotherapy*. 2016;66(2):225-245. doi:10.1080/00207284.2015.1106190

114. Moorhead TWJ, Stanfield A, Spencer M, et al. Progressive temporal lobe grey matter loss in adolescents with schizotypal traits and mild intellectual impairment. *Psychiatry Research - Neuroimaging*. 2009;174(2):105-109. doi:10.1016/j.pscychresns.2009.04.003

115. Morelli N, Fogler J, Tembulkar S, et al. Potentially traumatic events in youth with and at clinical high risk for psychosis. *Early Intervention in Psychiatry*. 2019;13(4):805-809. doi:10.1111/eip.12565

116. Morey RA, Inan S, Mitchell T v., Perkins DO, Lieberman JA, Belger A. Imaging Frontostriatal Function in Ultra-High-Risk, Early, and Chronic Schizophrenia During Executive Processing. *Archives of General Psychiatry*. 2005;62(3):254. doi:10.1001/archpsyc.62.3.254

117. Morita K, Kobayashi H, Takeshi K, Tsujino N, Nemoto T, Mizuno M. Poor outcome associated with symptomatic deterioration among help-seeking individuals at risk for psychosis: a naturalistic follow-up study. *Early Intervention in Psychiatry*. 2014;8(1):24-31. doi:10.1111/eip.12032

118. Morley KI, Cotton SM, Conus P, et al. *Familial Psychopathology in the First Episode Psychosis Outcome Study*.

119. Morrison AP, Bentall RP, French P, et al. Randomised controlled trial of early detection and cognitive therapy for preventing transition to psychosis in high-risk individuals. *British Journal of Psychiatry*. 2002;181(S43):s78-s84. doi:10.1192/bjp.181.43.s78

120. Morrison AP, French P, Walford L, et al. Cognitive therapy for the prevention of psychosis in people at ultra-high risk. *British Journal of Psychiatry*. 2004;185(4):291-297. doi:10.1192/bjp.185.4.291

121. Morrison AP, French P, Lewis SW, et al. Psychological factors in people at ultra-high risk of psychosis: Comparisons with non-patients and associations with symptoms. *Psychological Medicine*. 2006;36(10):1395-1404. doi:10.1017/S0033291706007768

122. Morrison AP, French P, Wells A. Metacognitive beliefs across the continuum of psychosis: Comparisons between patients with psychotic disorders, patients at ultra-high risk and non-patients. *Behaviour Research and Therapy*. 2007;45(9):2241-2246. doi:10.1016/j.brat.2007.01.002

123. Morrison AP, French P, Parker S, et al. Three-year follow-up of a randomized controlled trial of cognitive therapy for the prevention of psychosis in people at ultrahigh risk. *Schizophrenia Bulletin*. 2007;33(3):682-687. doi:10.1093/schbul/sbl042

124. Morrison AP, Stewart SLK, French P, et al. Early detection and intervention evaluation for people at high-risk of psychosis-2 (EDIE-2): trial rationale, design and baseline characteristics. *Early Intervention in Psychiatry*. 2011;5(1):24-32. doi:10.1111/j.1751-7893.2010.00254.x

125. Morrison AP, Birchwood M, Pyle M, et al. Impact of cognitive therapy on internalised stigma in people with at-risk mental states. *British Journal of Psychiatry*. 2013;203(2):140-145. doi:10.1192/bjp.bp.112.123703

126. Moskow DM, Addington J, Bearden CE, et al. The relations of age and pubertal development with cortisol and daily stress in youth at clinical risk for psychosis. *Schizophrenia Research*. 2016;172(1-3):29-34. doi:10.1016/j.schres.2016.02.002

127. Mossaheb N, Becker J, Schaefer MR, et al. The Community Assessment of Psychic Experience (CAPE) questionnaire as a screening-instrument in the detection of individuals at ultra-high risk for psychosis. *Schizophrenia Research*. 2012;141(2-3):210-214. doi:10.1016/j.schres.2012.08.008

128. Mossaheb N, Schäfer MR, Schlögelhofer M, et al. Effect of omega-3 fatty acids for indicated prevention of young patients at risk for psychosis: When do they begin to be effective? *Schizophrenia Research*. 2013;148(1-3):163-167. doi:10.1016/j.schres.2013.05.027

129. Mossaheb N, Schäfer MR, Schlögelhofer M, et al. Predictors of longer-term outcome in the Vienna omega-3 high-risk study. *Schizophrenia Research*. 2018;193:168-172. doi:10.1016/j.schres.2017.08.010

130. Mossaheb N, Papageorgiou K, Schäfer MR, Becker J, Schloegelhofer M, Amminger GP. Changes in triglyceride levels in ultra-high risk for psychosis individuals treated with omega-3 fatty acids. *Early Intervention in Psychiatry*. 2018;12(1):30-36. doi:10.1111/eip.12275

131. Mössner R, Schuhmacher A, Wagner M, et al. DAOA/G72 predicts the progression of prodromal syndromes to first episode psychosis. *European Archives of Psychiatry and Clinical Neuroscience*. 2010;260(3):209-215. doi:10.1007/s00406-009-0044-y

132. Moukas G, Gourzis P, Beratis IN, Beratis S. Sex differences in prepsychotic “prodromal” symptomatology and its association with Positive and Negative Syndrome Scale active phase psychopathology in male and female patients. *Comprehensive Psychiatry*. 2010;51(5):546-551. doi:10.1016/j.comppsych.2009.11.003

133. Mourik K, Decrescenzo P, Brucato G, et al. Various neurocognitive deficits and conversion risk in individuals at clinical high risk for psychosis. *Early Intervention in Psychiatry*. 2017;11(3):250-254. doi:10.1111/eip.12296

134. Mukkala S, Ilonen T, Nordström T, et al. Different vulnerability indicators for psychosis and their neuropsychological characteristics in the Northern Finland 1986 Birth Cohort. *Journal of Clinical and Experimental Neuropsychology*. 2011;33(4):385-394. doi:10.1080/13803395.2010.524148

135. Müller H, de Millas W, Gaebel W, et al. Negative schemata about the self and others and paranoid ideation in at-risk states and those with persisting positive symptoms. *Early Intervention in Psychiatry*. 2018;12(6):1157-1165. doi:10.1111/eip.12433

136. Murphy J, Blanchard MM, Rawdon C, et al. Language processing abnormalities in adolescents with psychotic-like experiences: An event related potential study. *Schizophrenia Research*. 2012;137(1-3):91-96. doi:10.1016/j.schres.2012.01.017

137. Murphy JR, Rawdon C, Kelleher I, et al. Reduced duration mismatch negativity in adolescents with psychotic symptoms: further evidence for mismatch negativity as a possible biomarker for vulnerability to psychosis. *BMC Psychiatry*. 2013;13(1):45. doi:10.1186/1471-244X-13-45

138. Myin-Germeys I, Marcelis M, Krabbendam L, Delespaul P, van Os J. Subtle Fluctuations in Psychotic Phenomena as Functional States of Abnormal Dopamine Reactivity in Individuals at Risk. *Biological Psychiatry*. 2005;58(2):105-110. doi:10.1016/j.biopsych.2005.02.012

139. MYIN-GERMEYS I, DELESPAUL PH, van OS J. Behavioural sensitization to daily life stress in psychosis. *Psychological Medicine*. 2005;35(5):733-741. doi:10.1017/S0033291704004179

140. Myles-Worsley M, Ord L, Blailes F, Ngiralmau H, Freedman R. P50 sensory gating in adolescents from a pacific island isolate with elevated risk for schizophrenia. *Biological Psychiatry*. 2004;55(7):663-667. doi:10.1016/j.biopsych.2003.12.006

141. Myles-Worsley M, Blailes F, Ord LM, Weaver S, Dever G, Faraone S v. The Palau Early Psychosis Study: Distribution of cases by level of genetic risk. *American Journal of Medical Genetics, Part B: Neuropsychiatric Genetics*. 2007;144(1):5-9. doi:10.1002/ajmg.b.30362

142. Mäki P, Veijola J, Rantakallio P, Jokelainen J, Jones PB, Isohanni M. Schizophrenia in the offspring of antenatally depressed mothers: a 31-year follow-up of the Northern Finland 1966 Birth Cohort. *Schizophrenia Research*. 2004;66(1):79-81. doi:10.1016/S0920-9964(02)00437-1

143. Mäki P, Koskela S, Murray GK, et al. Difficulty in making contact with others and social withdrawal as early signs of psychosis in adolescents – the Northern Finland Birth Cohort 1986. *European Psychiatry*. 2014;29(6). doi:10.1016/j.eurpsy.2013.11.003

144. Nagai T, Tada M, Kirihara K, et al. Auditory mismatch negativity and P3a in response to duration and frequency changes in the early stages of psychosis. *Schizophrenia Research*. 2013;150(2-3):547-554. doi:10.1016/j.schres.2013.08.005

145. Nagai T, Kirihara K, Tada M, et al. Reduced Mismatch Negativity is Associated with Increased Plasma Level of Glutamate in First-episode Psychosis. *Scientific Reports*. 2017;7(1):2258. doi:10.1038/s41598-017-02267-1

146. Nakamura K, Takahashi T, Nemoto K, et al. Gray Matter Changes in Subjects at High Risk for Developing Psychosis and First-Episode Schizophrenia: A Voxel-Based Structural MRI Study. *Frontiers in Psychiatry*. 2013;4. doi:10.3389/fpsyt.2013.00016

147. Nakamura M, Takahashi T, Takayanagi Y, et al. Surface morphology of the orbitofrontal cortex in individuals at risk of psychosis: a multicenter study. *European Archives of Psychiatry and Clinical Neuroscience*. 2019;269(4):397-406. doi:10.1007/s00406-018-0890-6

148. Naqvi HA, Hussain S, Islam M, Huma S. Early psychosis symptoms. *J Coll Physicians Surg Pak*. 2014;24(3). doi:03.2014/JCPSP.198202

149. Natsubori T, Hashimoto R ichiro, Yahata N, et al. An fMRI study of visual lexical decision in patients with schizophrenia and clinical high-risk individuals. *Schizophrenia Research*. 2014;157(1-3):218-224. doi:10.1016/j.schres.2014.05.027

150. Negueruela-López M, Jesús Mardomingo M, Sánchez P, Jiménez Díaz F. *Letter to the Editor PRODROMAL OBSESSIVE-COMPULSIVE SYMPTOMS IN A CASE OF SCHIZOPHRENIA*. Vol 40.; 2012.

151. Nelson B, Yung AR. When things are not as they seem: Detecting first-episode psychosis upon referral to ultra high risk ('prodromal’) clinics. *Early Intervention in Psychiatry*. 2007;1(2):208-211. doi:10.1111/j.1751-7893.2007.00028.x

152. Nelson B, Yung AR. Can Clinicians Predict Psychosis in an Ultra High Risk Group? *Australian & New Zealand Journal of Psychiatry*. 2010;44(7):625-630. doi:10.3109/00048671003620210

153. Nelson B, Yuen K, Yung AR. Ultra high risk (UHR) for psychosis criteria: Are there different levels of risk for transition to psychosis? *Schizophrenia Research*. 2011;125(1):62-68. doi:10.1016/j.schres.2010.10.017

154. Nelson B, Killackey E, Yung AR, Alvarez-Jimenez M, McGorry PD. Prodromal phase and first-episode schizophrenia. In: Lauriello J, Pallanti S, eds. *Clinical Manual for Treatment of Schizophrenia.* XXV. American Psychiatric Publishing, Inc.; 2012:71-109.

155. Nelson B, Yuen HP, Wood SJ, et al. Long-term Follow-up of a Group at Ultra High Risk (“Prodromal”) for Psychosis. *JAMA Psychiatry*. 2013;70(8):793. doi:10.1001/jamapsychiatry.2013.1270

156. Nelson B, Thompson A, Chanen AM, Amminger GP, Yung AR. Is basic self-disturbance in ultra-high risk for psychosis (‘prodromal’) patients associated with borderline personality pathology? *Early Intervention in Psychiatry*. 2013;7(3):306-310. doi:10.1111/eip.12011

157. Nelson B, Yuen HP, Lin A, et al. Further examination of the reducing transition rate in ultra high risk for psychosis samples: The possible role of earlier intervention. *Schizophrenia Research*. 2016;174(1-3):43-49. doi:10.1016/j.schres.2016.04.040

158. Nelson B, Amminger GP, Yuen HP, et al. Staged Treatment in Early Psychosis: A sequential multiple assignment randomised trial of interventions for ultra high risk of psychosis patients. *Early Intervention in Psychiatry*. 2018;12(3):292-306. doi:10.1111/eip.12459

159. Nelson B, Yung AR, McGorry PD. Importance of Variable Selection in Multimodal Prediction Models in Patients at Clinical High Risk for Psychosis and Recent-Onset Depression. *JAMA Psychiatry*. 2019;76(3):339. doi:10.1001/jamapsychiatry.2018.4234

160. Nelson B, Li E, Cicero DC, et al. The construct validity of the Inventory of Psychotic‐Like Anomalous Self‐Experiences (IPASE) as a measure of minimal self‐disturbance: Preliminary data. *Early Intervention in Psychiatry*. 2019;13(3):686-691. doi:10.1111/eip.12711

161. Nelson B. The challenges of neuroimaging the self, perspective-taking and psychosis risk. *Australian & New Zealand Journal of Psychiatry*. 2020;54(10):1037-1038. doi:10.1177/0004867420937802

162. Nelson B, Amminger GP, Bechdolf A, et al. Evidence for preventive treatments in young patients at clinical high risk of psychosis: the need for context. *The Lancet Psychiatry*. 2020;7(5):378-380. doi:10.1016/S2215-0366(19)30513-9

163. Nenadic I, Dietzek M, Schönfeld N, et al. Brain structure in people at ultra-high risk of psychosis, patients with first-episode schizophrenia, and healthy controls: a VBM study. *Schizophrenia Research*. 2015;161(2-3):169-176. doi:10.1016/j.schres.2014.10.041

164. Nenadic I, Maitra R, Basu S, et al. Associations of hippocampal metabolism and regional brain grey matter in neuroleptic-naïve ultra-high-risk subjects and first-episode schizophrenia. *European Neuropsychopharmacology*. 2015;25(10):1661-1668. doi:10.1016/j.euroneuro.2015.05.005

165. Newberry RE, Dean DJ, Sayyah MD, Mittal VA. What prevents youth at clinical high risk for psychosis from engaging in physical activity? An examination of the barriers to physical activity. *Schizophrenia Research*. 2018;201:400-405. doi:10.1016/j.schres.2018.06.011

166. Ngiralmau H, Blailes F, Myles-Worsley M, Ord LM. Preventive intervention for early psychosis in adolescents--the Palau Youth at Risk Project. *Pac Health Dialog*. 2005;12(1).

167. Nieman D, Becker H, van de Fliert R, et al. Antisaccade task performance in patients at ultra high risk for developing psychosis. *Schizophrenia Research*. 2007;95(1-3):54-60. doi:10.1016/j.schres.2007.06.022

168. Nieman DH, Rike WH, Becker HE, et al. Prescription of antipsychotic medication to patients at ultra high risk of developing psychosis. *International Clinical Psychopharmacology*. 2009;24(4):223-228. doi:10.1097/YIC.0b013e32832e0a76

169. Nieman DH, Velthorst E, Becker HE, et al. The Strauss and Carpenter Prognostic Scale in subjects clinically at high risk of psychosis. *Acta Psychiatrica Scandinavica*. 2013;127(1):53-61. doi:10.1111/j.1600-0447.2012.01899.x

170. Nieman DH, Ruhrmann S, Dragt S, et al. Psychosis Prediction: Stratification of Risk Estimation With Information-Processing and Premorbid Functioning Variables. *Schizophrenia Bulletin*. 2014;40(6):1482-1490. doi:10.1093/schbul/sbt145

171. Nieman DH, Dragt S, van Duin EDA, et al. COMT Val 158 Met genotype and cannabis use in people with an At Risk Mental State for psychosis: Exploring Gene x Environment interactions. *Schizophrenia Research*. 2016;174(1-3):24-28. doi:10.1016/j.schres.2016.03.015

172. Niemi LT, Suvisaari JM, Haukka JK, Lönnqvist JK. Childhood predictors of future psychiatric morbidity in offspring of mothers with psychotic disorder. *British Journal of Psychiatry*. 2005;186(2):108-114. doi:10.1192/bjp.186.2.108

173. Niendam TA, Bearden CE, Zinberg J, Johnson JK, O’Brien M, Cannon TD. The course of neurocognition and social functioning in individuals at ultra high risk for psychosis. *Schizophrenia Bulletin*. 2007;33(3):772-781. doi:10.1093/schbul/sbm020

174. Niendam TA, Bearden CE, Johnson JK, et al. Neurocognitive performance and functional disability in the psychosis prodrome. *Schizophrenia Research*. 2006;84(1):100-111. doi:10.1016/j.schres.2006.02.005

175. Niendam TA, Horwitz J, Bearden CEB, Cannon TD. *Ecological Assessment of Executive Dysfunction in the Psychosis Prodrome: A Pilot Study*.

176. Niendam TA, Lesh TA, Yoon J, et al. Impaired context processing as a potential marker of psychosis risk state. *Psychiatry Research: Neuroimaging*. 2014;221(1). doi:10.1016/j.pscychresns.2013.09.001

177. Niles HF, Walsh BC, Woods SW, Powers AR. Does hallucination perceptual modality impact psychosis risk? *Acta Psychiatrica Scandinavica*. 2019;140(4):360-370. doi:10.1111/acps.13078

178. Nitka F, Richter J, Parzer P, Resch F, Henze R. Health-related quality of life among adolescents: A comparison between subjects at ultra-high risk for psychosis and healthy controls. *Psychiatry Research*. 2016;235:110-115. doi:10.1016/j.psychres.2015.11.040

179. Nogovitsyn N, Souza R, Muller M, et al. Aberrant limbic brain structures in young individuals at risk for mental illness. *Psychiatry and Clinical Neurosciences*. 2020;74(5):294-302. doi:10.1111/pcn.12985

180. Nogueira AS, Serpa MH, Alves TM, et al. Influence of migration on the thought process of individuals at ultra-high risk for psychosis. doi:10.1590/1516-4446

181. Nordholm D, Poulsen HE, Hjorthøj C, et al. Systemic oxidative DNA and RNA damage are not increased during early phases of psychosis: A case control study. *Psychiatry Research*. 2016;241:201-206. doi:10.1016/j.psychres.2016.04.062

182. Nordholm D, Rostrup E, Mondelli V, et al. Multiple measures of HPA axis function in ultra high risk and first-episode schizophrenia patients. *Psychoneuroendocrinology*. 2018;92:72-80. doi:10.1016/j.psyneuen.2018.03.015

183. Novak Sarotar B, Pesek MB, Agius M, Kocmur M. Duration of untreated psychosis and it’s effect on the symptomatic recovery in schizophrenia - preliminary results. *Neuro Endocrinol Lett*. 2008;29(6).

184. Nowak U, Eisenacher S, Braun H, et al. Monocausal Attributions Along Cross-Sections of Psychosis Development and Links with Psychopathology and Data Gathering Style. *Cognitive Therapy and Research*. 2018;42(5):699-710. doi:10.1007/s10608-018-9907-8

185. Ntouros E, Karanikas E, Floros G, et al. Social cognition in the course of psychosis and its correlation with biomarkers in a male cohort. *Cognitive Neuropsychiatry*. 2018;23(2):103-115. doi:10.1080/13546805.2018.1440201

186. Nuchpongsai P, Arakaki H, Langman P, Ogura C. N2 and P3b components of the event-related potential in students at risk for psychosis. *Psychiatry Research*. 1999;88(2):131-141. doi:10.1016/S0165-1781(99)00037-2

187. Nussbaum LA, Hogea LM, Andreescu NI, Grădinaru RC, Puiu M, Todica A. The prognostic and clinical significance of neuroimagistic and neurobiological vulnerability markers in correlation with the molecular pharmacogenetic testing in psychoses and ultra high-risk categories. *Rom J Morphol Embryol*. 2016;57(3):959-967.

188. Nussbaum LA, Hogea LM, Chiriac DV, et al. The effect of neurobiological changes in the brain of children with schizophrenia, ultra high-risk for psychosis and epilepsy: clinical correlations with EEG and neuroimagistic abnormalities. *Rom J Morphol Embryol*. 2017;58(4):1435-1446.

189. O’Banion MKG. Memory functions in youth with prodromal schizophrenia. *Dissertation Abstracts International: Section B: The Sciences and Engineering*. 2010;70(9-B):5837.

190. O’Brien MP, Gordon JL, Bearden CE, Lopez SR, Kopelowicz A, Cannon TD. Positive family environment predicts improvement in symptoms and social functioning among adolescents at imminent risk for onset of psychosis. *Schizophrenia Research*. 2006;81(2-3):269-275. doi:10.1016/j.schres.2005.10.005

191. O’Brien MP, Zinberg JL, Bearden CE, et al. Parent attitudes and parent adolescent interaction in families of youth at risk for psychosis and with recent-onset psychotic symptoms. *Early Intervention in Psychiatry*. 2008;2(4). doi:10.1111/j.1751-7893.2008.00088.x

192. O’Brien MP, Zinberg JL, Ho L, et al. Family problem solving interactions and 6-month symptomatic and functional outcomes in youth at ultra-high risk for psychosis and with recent onset psychotic symptoms: A longitudinal study. *Schizophrenia Research*. 2009;107(2-3):198-205. doi:10.1016/j.schres.2008.10.008

193. O’Brien MP, Miklowitz DJ, Candan KA, et al. A randomized trial of family focused therapy with populations at clinical high risk for psychosis: Effects on interactional behavior. *Journal of Consulting and Clinical Psychology*. 2014;82(1):90-101. doi:10.1037/a0034667

194. O’Brien MP, Miklowitz DJ, Cannon TD. Decreases in perceived maternal criticism predict improvement in subthreshold psychotic symptoms in a randomized trial of family-focused therapy for individuals at clinical high risk for psychosis. *Journal of Family Psychology*. 2015;29(6):945-951. doi:10.1037/fam0000123

195. O’Connor R, Sota M, Cortesi M, Fusar-Poli P. Quetiapine as a first-choice agent in subjects at high-risk to psychosis? *Medical Hypotheses*. 2007;69(1). doi:10.1016/j.mehy.2006.11.037

196. O’Connor K, Nelson B, Lin A, Wood SJ, Yung A, Thompson A. Are UHR patients who present with hallucinations alone at lower risk of transition to psychosis? *Psychiatry Research*. 2016;235:177-196. doi:10.1016/j.psychres.2015.05.085

197. O’ Connor K, Nelson B, Cannon M, Yung A, Thompson A. Perceptual abnormalities in an ultra‐high risk for psychosis population relationship to trauma and co‐morbid disorder. *Early Intervention in Psychiatry*. 2019;13(2):231-240. doi:10.1111/eip.12469

198. Obyedkov I, Skuhareuskaya M, Skugarevsky O, et al. Saccadic eye movements in different dimensions of schizophrenia and in clinical high-risk state for psychosis. *BMC Psychiatry*. 2019;19(1):110. doi:10.1186/s12888-019-2093-8

199. O’Donoghue B, Lyne J, Renwick L, et al. A descriptive study of ‘non-cases’ and referral rates to an early intervention for psychosis service. *Early Intervention in Psychiatry*. 2012;6(3):276-282. doi:10.1111/j.1751-7893.2011.00328.x

200. O’Donoghue B, Nelson B, Yuen HP, et al. Social environmental risk factors for transition to psychosis in an Ultra-High Risk population. *Schizophrenia Research*. 2015;161(2-3):150-155. doi:10.1016/j.schres.2014.10.050

201. O’Donoghue B, Yung AR, Wood S, et al. Neighbourhood characteristics and the rate of identification of young people at ultra-high risk for psychosis. *Schizophrenia Research*. 2015;169(1-3):214-216. doi:10.1016/j.schres.2015.09.002

202. O’Donoghue B, Rudhran V, Kumar S, et al. Screening for the ultra-high risk state in a youth mental health service. *Schizophrenia Research*. 2018;202:401-403. doi:10.1016/j.schres.2018.06.045

203. Ohmuro N, Katsura M, Obara C, et al. Deficits of cognitive theory of mind and its relationship with functioning in individuals with an at-risk mental state and first-episode psychosis. *Psychiatry Research*. 2016;243:318-325. doi:10.1016/j.psychres.2016.06.051

204. Ohmuro N, Katsura M, Obara C, et al. The relationship between cognitive insight and cognitive performance among individuals with at-risk mental state for developing psychosis. *Schizophrenia Research*. 2018;192:281-286. doi:10.1016/j.schres.2017.04.031

205. Oliver D, Spada G, Englund A, et al. Real-world digital implementation of the Psychosis Polyrisk Score (PPS): A pilot feasibility study. *Schizophrenia Research*. 2020;226:176-183. doi:10.1016/j.schres.2020.04.015

206. Olvet DM, Stearns WH, McLaughlin D, Auther AM, Correll CU, Cornblatt BA. Comparing clinical and neurocognitive features of the schizophrenia prodrome to the bipolar prodrome. *Schizophrenia Research*. 2010;123(1):59-63. doi:10.1016/j.schres.2010.07.005

207. Olvet DM, Carrión RE, Auther AM, Cornblatt BA. Self-awareness of functional impairment in individuals at clinical high-risk for psychosis. *Early Intervention in Psychiatry*. 2015;9(2):100-107. doi:10.1111/eip.12086

208. Omer S, Kirkbride JB, Pringle DG, Russell V, O’Callaghan E, Waddington JL. Neighbourhood-level socio-environmental factors and incidence of first episode psychosis by place at onset in rural Ireland: The Cavan–Monaghan First Episode Psychosis Study [CAMFEPS]. *Schizophrenia Research*. 2014;152(1):152-157. doi:10.1016/j.schres.2013.11.019

209. Onozato M, Umino M, Shoji A, et al. Serum d- and l-lactate, pyruvate and glucose levels in individuals with at-risk mental state and correlations with clinical symptoms. *Early Intervention in Psychiatry*. 2020;14(4):410-417. doi:10.1111/eip.12866

210. Onozato M, Uta A, Magarida A, et al. Alterations in methionine to homocysteine ratio in individuals with first-episode psychosis and those with at-risk mental state. *Clinical Biochemistry*. 2020;77:48-53. doi:10.1016/j.clinbiochem.2019.12.003

211. Ord LM. Stress as predictor of worsening psychopathology: Palauan adolescents with psychosis prodrome. *Dissertation Abstracts International Section A: Humanities and Social Sciences*. 2008;69 (2-A):756.

212. Oribe N, Hirano Y, Kanba S, et al. Early and late stages of visual processing in individuals in prodromal state and first episode schizophrenia: An ERP study. *Schizophrenia Research*. 2013;146(1-3):95-102. doi:10.1016/j.schres.2013.01.015

213. Oribe N, Hirano Y, del Re E, et al. Progressive reduction of auditory evoked gamma in first episode schizophrenia but not clinical high risk individuals. *Schizophrenia Research*. 2019;208:145-152. doi:10.1016/j.schres.2019.03.025

214. Oribe N, Hirano Y, Re E, et al. Longitudinal evaluation of visual P300 amplitude in clinical high‐risk subjects: An event‐related potential study. *Psychiatry and Clinical Neurosciences*. 2020;74(10):527-534. doi:10.1111/pcn.13083

215. Orosz AT, Feldon J, Simon AE, et al. Learned Irrelevance and Associative Learning Is Attenuated in Individuals at Risk for Psychosis but not in Asymptomatic First-Degree Relatives of Schizophrenia Patients: Translational State Markers of Psychosis? *Schizophrenia Bulletin*. 2011;37(5):973-981. doi:10.1093/schbul/sbp165

216. Ortega L, Montalvo I, Monseny R, Vilella E, Labad J. Perceived stress mediates the relationship between social adaptation and quality of life in individuals at ultra high risk of psychosis. *Early Intervention in Psychiatry*. 2019;13(6):1447-1454. doi:10.1111/eip.12791

217. Osborne KJ, Bernard JA, Gupta T, et al. Beat gestures and postural control in youth at ultrahigh risk for psychosis. *Schizophrenia Research*. 2017;185:197-199. doi:10.1016/j.schres.2016.11.028

218. Osborne KJ, Mittal VA. External validation and extension of the NAPLS-2 and SIPS-RC personalized risk calculators in an independent clinical high-risk sample. *Psychiatry Research*. 2019;279:9-14. doi:10.1016/j.psychres.2019.06.034

219. Osborne KJ, Mittal VA. Postural sway and neurocognition in individuals meeting criteria for a clinical high-risk syndrome. *European Archives of Psychiatry and Clinical Neuroscience*. 2022;272(1):155-160. doi:10.1007/s00406-021-01234-2

220. Ota M, Obu S, Sato N, Asada T. Neuroimaging study in subjects at high risk of psychosis revealed by the Rorschach test and first-episode schizophrenia. *Acta Neuropsychiatrica*. 2011;23(3):125-131. doi:10.1111/j.1601-5215.2011.00547.x

221. Owens DGC, Miller P, Lawrie SM, Johnstone EC. Pathogenesis of schizophrenia: a psychopathological perspective. *British Journal of Psychiatry*. 2005;186(5):386-393. doi:10.1192/bjp.186.5.386

222. Owoso A, Ndetei DM, Mbwayo AW, Mutiso VN, Khasakhala LI, Mamah D. Validation of a modified version of the PRIME screen for psychosis-risk symptoms in a non-clinical Kenyan youth sample. *Comprehensive Psychiatry*. 2014;55(2):380-387. doi:10.1016/j.comppsych.2013.10.004

223. Özgürdal S, Gudlowski Y, Witthaus H, et al. Reduction of auditory event-related P300 amplitude in subjects with at-risk mental state for schizophrenia. *Schizophrenia Research*. 2008;105(1-3):272-278. doi:10.1016/j.schres.2008.05.017

224. Özgurdal S, Littmann E, Hauser M, et al. Neurocognitive performances in participants of at-risk mental state for schizophrenia and in first-episode patients. *Journal of Clinical and Experimental Neuropsychology*. 2009;31(4):392-401. doi:10.1080/13803390802206406

225. Padmanabhan JL, Shah JL, Tandon N, Keshavan MS. The “polyenviromic risk score”: Aggregating environmental risk factors predicts conversion to psychosis in familial high-risk subjects. *Schizophrenia Research*. 2017;181:17-22. doi:10.1016/j.schres.2016.10.014

226. Padula MC, Schaer M, Armando M, et al. Cortical morphology development in patients with 22q11.2 deletion syndrome at ultra-high risk of psychosis. *Psychological Medicine*. 2018;48(14):2375-2383. doi:10.1017/S0033291717003920

227. Paetzold I, Myin-Germeys I, Schick A, et al. Stress reactivity as a putative mechanism linking childhood trauma with clinical outcomes in individuals at ultra-high-risk for psychosis: Findings from the EU-GEI High Risk Study. *Epidemiology and Psychiatric Sciences*. 2021;30:e40. doi:10.1017/S2045796021000251

228. Palmier-Claus JE, Taylor PJ, Gooding P, Dunn G, Lewis SW. Affective variability predicts suicidal ideation in individuals at ultra-high risk of developing psychosis: An experience sampling study. *British Journal of Clinical Psychology*. 2012;51(1):72-83. doi:10.1111/j.2044-8260.2011.02013.x

229. Palmier-Claus JE, Dunn G, Lewis SW. Emotional and symptomatic reactivity to stress in individuals at ultra-high risk of developing psychosis. *Psychological Medicine*. 2012;42(5):1003-1012. doi:10.1017/S0033291711001929

230. Palmier-Claus JE, Dunn G, Taylor H, Morrison AP, Lewis SW. Cognitive-self consciousness and metacognitive beliefs: Stress sensitization in individuals at ultra-high risk of developing psychosis. *British Journal of Clinical Psychology*. 2013;52(1):26-41. doi:10.1111/j.2044-8260.2012.02043.x

231. Palmier-Claus JE, Taylor PJ, Ainsworth J, Machin M, Dunn G, Lewis SW. The temporal association between self-injurious thoughts and psychotic symptoms: A mobile phone assessment study. *Suicide and Life-Threatening Behavior*. 2014;44(1):101-110. doi:10.1111/sltb.12064

232. Palmier-Claus J, Berry K, Darrell-Berry H, et al. Childhood adversity and social functioning in psychosis: Exploring clinical and cognitive mediators. *Psychiatry Research*. 2016;238:25-32. doi:10.1016/j.psychres.2016.02.004

233. Papmeyer M, Würsch I, Studerus E, Stieglitz RD, Riecher-Rössler A. The role of vulnerability factors in individuals with an at-risk mental state of psychosis. *neuropsychiatrie*. 2016;30(1):18-26. doi:10.1007/s40211-016-0179-9

234. Papmeyer M, Aston J, Everts-Graber J, et al. Outcome of individuals “not at risk of psychosis” and prognostic accuracy of the Basel Screening Instrument for Psychosis (BSIP). *Early Intervention in Psychiatry*. 2018;12(5):907-914. doi:10.1111/eip.12401

235. Parham LD, Roush S, Downing DT, Michael PG, McFarlane WR. Sensory characteristics of youth at clinical high risk for psychosis. *Early Intervention in Psychiatry*. 2019;13(2):264-271. doi:10.1111/eip.12475

236. Park HY, Hwang JY, Jung WH, et al. Altered asymmetry of the anterior cingulate cortex in subjects at genetic high risk for psychosis. *Schizophrenia Research*. 2013;150(2-3):512-518. doi:10.1016/j.schres.2013.08.027

237. Park J, Lho SK, Hwang WJ, et al. Impaired error-related processing in patients with first-episode psychosis and subjects at clinical high risk for psychosis: An event-related potential study. *Psychiatry and Clinical Neurosciences*. Published online 2021. doi:10.1111/pcn.13219

238. Parker SK, Mulligan LD, Milner P, Bowe S, Palmier-Claus JE. Metacognitive Therapy for Individuals at High Risk of Developing Psychosis: A Pilot Study. *Frontiers in Psychology*. 2020;10. doi:10.3389/fpsyg.2019.02741

239. Parrish EM, Kim NS, Woodberry KA, Friedman-Yakoobian M. Clinical high risk for psychosis: The effects of labelling on public stigma in a undergraduate population. *Early Intervention in Psychiatry*. 2019;13(4):874-881. doi:10.1111/eip.12691

240. Paterlini F, Pelizza L, Galli G, et al. Interrater reliability of the authorized Italian version of the Comprehensive Assessment of At-Risk Mental States (CAARMS-ITA). *Journal of Psychopathology*. 2019;25(1):24-28.

241. Patton HN, Cowan HR, Mittal VA. Changes in core beliefs over time predict symptoms and functioning in clinical high risk for psychosis. *Early Intervention in Psychiatry*. Published online 2021. doi:10.1111/eip.13156

242. Pauly K, Seiferth NY, Kellermann T, et al. The interaction of working memory and emotion in persons clinically at risk for psychosis: An fMRI pilot study. *Schizophrenia Research*. 2010;120(1-3):167-176. doi:10.1016/j.schres.2009.12.008

243. Pawełczyk A, Kotlicka-Antczak M, Rabe-Jabłońska J, Pawełczyk T, Ruszpel A, Łojek E. Figural fluency and immediate visual memory in patients with at-risk mental state for psychosis: empirical study. *Early Intervention in Psychiatry*. 2015;9(4):324-330. doi:10.1111/eip.12116

244. Pawełczyk T, Trafalska E, Kotlicka-Antczak M, Pawełczyk A. The association between polyunsaturated fatty acid consumption and the transition to psychosis in ultra-high risk individuals. *Prostaglandins, Leukotrienes and Essential Fatty Acids*. 2016;108:30-37. doi:10.1016/j.plefa.2016.03.010

245. Pawełczyk T, Trafalska E, Pawełczyk A, Kotlicka-Antczak M. Differences in omega-3 and omega-6 polyunsaturated fatty acid consumption in people at ultra-high risk of psychosis, first-episode schizophrenia, and in healthy controls. *Early Intervention in Psychiatry*. 2017;11(6):498-508. doi:10.1111/eip.12267

246. Pawełczyk A, Kotlicka‐Antczak M, Łojek E, Pawełczyk T. Preliminary study of higher‐order language and extralinguistic impairments in individuals with high clinical risk of psychosis and first episode of schizophrenia. *Early Intervention in Psychiatry*. 2019;13(3):369-378. doi:10.1111/eip.12482

247. Peedicayil J. Epigenetic mechanisms may underlie the increased risk for psychosis among immigrants. *Medical Hypotheses*. 2008;70(1). doi:10.1016/j.mehy.2007.04.002

248. Pelayo-Terán JM, Pérez-Iglesias R, Ramírez-Bonilla ML, et al. Epidemiological factors associated with treated incidence of first-episode non-affective psychosis in Cantabria: Insights from the Clinical Programme on Early Phases of Psychosis. *Early Intervention in Psychiatry*. 2008;2(3):178-187. doi:10.1111/j.1751-7893.2008.00074.x

249. Pelizza L, Raballo A, Semrov E, et al. Identification of young people at “Ultra-High Risk” (UHR) of developing psychosis: validation of the “Checklist per la valutazione dell’esordio psicotico” for use in primary care setting. *Journal of psychopathology*. 2016;22:172-179.

250. Pelizza L, Azzali S, Paterlini F, et al. The Italian Version of the Brief 21-Item Prodromal Questionnaire: Field Test, Psychometric Properties and Age-Sensitive Cut-Offs. *Psychopathology*. 2018;51(4):234-244. doi:10.1159/000490708

251. Pelizza L, Poletti M, Azzali S, et al. Suicidal Thinking and Behavior in Adolescents at Ultra‐High Risk of Psychosis: A Two‐year Longitudinal Study. *Suicide and Life-Threatening Behavior*. 2019;49(6):1637-1652. doi:10.1111/sltb.12549

252. Pelizza L, Raballo A, Semrov E, et al. Validation of the “early detection Primary Care Checklist” in an Italian community help-seeking sample: The “checklist per la Valutazione dell’Esordio Psicotico.” *Early Intervention in Psychiatry*. 2019;13(1):86-94. doi:10.1111/eip.12455

253. Pelizza L, Azzali S, Garlassi S, et al. Examining subjective experience of social cognition in early psychosis: Validation of the Italian version of the GEOPTE scale in an adolescent and young adult clinical sample. *Journal of Psychopathology*. 2019;25(4):220-230.

254. Pelizza L, Azzali S, Paterlini F, et al. Screening for psychosis risk among help-seeking adolescents: Application of the Italian version of the 16-item prodromal questionnaire (iPQ-16) in child and adolescent neuropsychiatry services. *Early Intervention in Psychiatry*. 2019;13(4):752-760. doi:10.1111/eip.12554

255. Pelizza L, Paterlini F, Azzali S, et al. The approved Italian version of the comprehensive assessment of at-risk mental states (CAARMS-ITA): Field test and psychometric features. *Early Intervention in Psychiatry*. 2019;13(4):810-817. doi:10.1111/eip.12669

256. Pelizza L, Poletti M, Azzali S, et al. Subjective experience of social cognition in adolescents at ultra-high risk of psychosis: findings from a 24-month follow-up study. *European Child & Adolescent Psychiatry*. 2020;29(12):1645-1657. doi:10.1007/s00787-020-01482-y

257. Pelizza L, Poletti M, Azzali S, et al. Anhedonia in adolescents at ultra-high risk (UHR) of psychosis: findings from a 1-year longitudinal study. *European Archives of Psychiatry and Clinical Neuroscience*. 2020;270(3):337-350. doi:10.1007/s00406-019-01018-9

258. Pelizza L, Poletti M, Azzali S, et al. Subjective experience of social cognition in young people at Ultra-High Risk of psychosis: a 2-year longitudinal study. *Nordic Journal of Psychiatry*. 2021;75(2):97-108. doi:10.1080/08039488.2020.1799430

259. Pelletier-Baldelli A, Strauss GP, Visser KH, Mittal VA. Initial development and preliminary psychometric properties of the Prodromal Inventory of Negative Symptoms (PINS). *Schizophrenia Research*. 2017;189:43-49. doi:10.1016/j.schres.2017.01.055

260. Pelletier-Baldelli A, Andrews-Hanna JR, Mittal VA. Resting state connectivity dynamics in individuals at risk for psychosis. *Journal of Abnormal Psychology*. 2018;127(3):314-325. doi:10.1037/abn0000330

261. Peralta D, Studerus E, Andreou C, et al. Exploring the predictive power of the unspecific risk category of the Basel Screening Instrument for Psychosis. *Early Intervention in Psychiatry*. 2019;13(4):969-976. doi:10.1111/eip.12719

262. Perez VB, Ford JM, Roach BJ, et al. Error monitoring dysfunction across the illness course of schizophrenia. *Journal of Abnormal Psychology*. 2012;121(2):372-387. doi:10.1037/a0025487

263. Perez VB, Shafer KM, Cadenhead KS. Visual information processing dysfunction across the developmental course of early psychosis. *Psychological Medicine*. 2012;42(10):2167-2179. doi:10.1017/S0033291712000426

264. Perez VB, Ford JM, Roach BJ, et al. Auditory Cortex Responsiveness During Talking and Listening: Early Illness Schizophrenia and Patients at Clinical High-Risk for Psychosis. *Schizophrenia Bulletin*. 2012;38(6):1216-1224. doi:10.1093/schbul/sbr124

265. Perez VB, Roach BJ, Woods SW, et al. Early auditory gamma-band responses in patients at clinical high risk for schizophrenia. In: *Supplements to Clinical Neurophysiology*. Vol 62. Elsevier B.V.; 2013:147-162. doi:10.1016/B978-0-7020-5307-8.00010-7

266. Perez VB, Woods SW, Roach BJ, et al. Automatic Auditory Processing Deficits in Schizophrenia and Clinical High-Risk Patients: Forecasting Psychosis Risk with Mismatch Negativity. *Biological Psychiatry*. 2014;75(6). doi:10.1016/j.biopsych.2013.07.038

267. Perivoliotis D, Morrison AP, Grant PM, French P, Beck AT. Negative performance beliefs and negative symptoms in individuals at ultra-high risk of psychosis: A preliminary study. *Psychopathology*. 2009;42(6):375-379. doi:10.1159/000236909

268. Perkins DO, Olde Loohuis L, Barbee J, et al. Polygenic Risk Score Contribution to Psychosis Prediction in a Target Population of Persons at Clinical High Risk. *American Journal of Psychiatry*. 2020;177(2):155-163. doi:10.1176/appi.ajp.2019.18060721

269. Peters BD, Schmitz N, Dingemans PM, et al. Preliminary evidence for reduced frontal white matter integrity in subjects at ultra-high-risk for psychosis. *Schizophrenia Research*. 2009;111(1-3):192-193. doi:10.1016/j.schres.2009.03.018

270. Peters BD, de Koning P, Dingemans P, Becker H, Linszen DH, de Haan L. Subjective Effects of Cannabis Before the First Psychotic Episode. *Australian & New Zealand Journal of Psychiatry*. 2009;43(12):1155-1162. doi:10.3109/00048670903179095

271. Peters BD, Dingemans PM, Dekker N, et al. White matter connectivity and psychosis in ultra-high-risk subjects: A diffusion tensor fiber tracking study. *Psychiatry Research: Neuroimaging*. 2010;181(1):44-50. doi:10.1016/j.pscychresns.2009.10.008

272. Petruzzelli MG, Margari M, Peschechera A, et al. Hyperprolactinemia and insulin resistance in drug naive patients with early onset first episode psychosis. *BMC Psychiatry*. 2018;18(1):246. doi:10.1186/s12888-018-1827-3

273. Pettersson-Yeo W, Benetti S, Marquand AF, et al. Using genetic, cognitive and multi-modal neuroimaging data to identify ultra-high-risk and first-episode psychosis at the individual level. *Psychological Medicine*. 2013;43(12):2547-2562. doi:10.1017/S003329171300024X

274. Pettersson-Yeo W, Benetti S, Frisciata S, et al. Does neuroanatomy account for superior temporal dysfunction in early psychosis? A multimodal MRI investigation. *Journal of Psychiatry and Neuroscience*. 2015;40(2):100-107. doi:10.1503/jpn.140082

275. Pflueger MO, Gschwandtner U, Stieglitz RD, Riecher-Rössler A. Neuropsychological deficits in individuals with an at risk mental state for psychosis - Working memory as a potential trait marker. *Schizophrenia Research*. 2007;97(1-3):14-24. doi:10.1016/j.schres.2007.09.003

276. Pflueger MO, Calabrese P, Studerus E, et al. The neuropsychology of emerging psychosis and the role of working memory in episodic memory encoding. *Psychology Research and Behavior Management*. 2018;Volume 11:157-168. doi:10.2147/PRBM.S149425

277. Phillips L, Yung AR, Hearn N, McFarlane C, Hallgren M, McGorry PD. Preventative Mental Health Care: Accessing the Target Population. *Australian & New Zealand Journal of Psychiatry*. 1999;33(6):912-917. doi:10.1046/j.1440-1614.1999.00613.x

278. Phillips LJ, Yung AR, McGorry PD. Identification of Young People at Risk of Psychosis: Validation of Personal Assessment and Crisis Evaluation Clinic Intake Criteria. *Australian & New Zealand Journal of Psychiatry*. 2000;34(2_suppl):S164-S169. doi:10.1080/000486700239

279. Phillips LJ, McGorry PD, Yuen HP, et al. Medium term follow-up of a randomized controlled trial of interventions for young people at ultra high risk of psychosis. *Schizophrenia Research*. 2007;96(1-3):25-33. doi:10.1016/j.schres.2007.05.018

280. Phillips LJ, Edwards J, McMurray N, Francey S. Comparison of Experiences of Stress and Coping Between Young People at Risk of Psychosis and a Non-Clinical Cohort. *Behavioural and Cognitive Psychotherapy*. 2012;40(1):69-88. doi:10.1017/S1352465811000397

281. Paino Piñeros MM, Lemos Giráldez S. Developing a combined predictor measure for early detection of psychosis proneness. *Actas espanolas de psiquiatria*. 31(5):244-251.

282. Pinkham AE, Penn DL, Perkins DO, Graham KA, Siegel M. Emotion perception and social skill over the course of psychosis: A comparison of individuals “at-risk” for psychosis and individuals with early and chronic schizophrenia spectrum illness. *Cognitive Neuropsychiatry*. 2007;12(3):198-212. doi:10.1080/13546800600985557

283. Piotrowski P, Kotowicz K, Rymaszewska J, et al. Allostatic load index and its clinical correlates at various stages of psychosis. *Schizophrenia Research*. 2019;210:73-80. doi:10.1016/j.schres.2019.06.009

284. Piskulic D, Barbato M, Liu L, Addington J. Pilot study of cognitive remediation therapy on cognition in young people at clinical high risk of psychosis. *Psychiatry Research*. 2015;225(1-2):93-98. doi:10.1016/j.psychres.2014.10.021

285. Piskulic D, Liu L, Cadenhead KS, et al. Social cognition over time in individuals at clinical high risk for psychosis: Findings from the NAPLS-2 cohort. *Schizophrenia Research*. 2016;171(1-3):176-181. doi:10.1016/j.schres.2016.01.017

286. Pitzianti M, Pontillo M, Vicari S, Armando M, Pasini A. 22q11 microdeletion syndrome and ultra‐high risk for psychosis: The role of neurological soft signs as an independent marker of vulnerability for psychosis. *Early Intervention in Psychiatry*. 2019;13(5):1191-1198. doi:10.1111/eip.12754

287. Pitizianti M, Casarelli L, Pontillo M, Vicari S, Armando M, Pasini A. Antipsychotics Do Not Influence Neurological Soft Signs in Children and Adolescents at Ultra-High Risk for Psychosis: A Pilot Study. *Journal of Psychiatric Practice*. 2019;25(3):186-191. doi:10.1097/PRA.0000000000000387

288. Platz C, Umbricht DS, Cattapan-Ludewig K, et al. Help-seeking pathways in early psychosis. *Social Psychiatry and Psychiatric Epidemiology*. 2006;41(12):967-974. doi:10.1007/s00127-006-0117-4

289. Poe SL, Brucato G, Bruno N, et al. Sleep disturbances in individuals at clinical high risk for psychosis. *Psychiatry Research*. 2017;249:240-243. doi:10.1016/j.psychres.2016.12.029

290. Polari A, Lavoie S, Yuen HP, et al. Clinical trajectories in the ultra-high risk for psychosis population. *Schizophrenia Research*. 2018;197:550-556. doi:10.1016/j.schres.2018.01.022

291. Polari A, Yuen HP, Amminger P, et al. Prediction of clinical outcomes beyond psychosis in the <scp>ultra‐high</scp> risk for psychosis population. *Early Intervention in Psychiatry*. 2021;15(3):642-651. doi:10.1111/eip.13002

292. Pollice R, di Giovambattista E, Ussorio D, et al. The service for Monitoring and Early Intervention against psychoLogical and mEntal suffering in young people (SMILE) at the University of L’Aquila: first year experience. *Early Intervention in Psychiatry*. 2007;1(3):271-275. doi:10.1111/j.1751-7893.2007.00037.x

293. Pontillo M, Tata MC, Averna R, Gargiullo P, Guerrera S, Vicari S. Clinical profile, conversion rate, and suicidal thinking and behaviour in children and adolescents at ultra-high risk for psychosis: a theoretical perspective. *Research in Psychotherapy: Psychopathology, Process and Outcome*. 2020;23(1). doi:10.4081/ripppo.2020.455

294. Popovic D, Ruef A, Dwyer DB, et al. Traces of Trauma: A Multivariate Pattern Analysis of Childhood Trauma, Brain Structure, and Clinical Phenotypes. *Biological Psychiatry*. 2020;88(11):829-842. doi:10.1016/j.biopsych.2020.05.020

295. Pos K, de Wit IE, van Dijk FA, et al. An experience sampling study on the ecological validity of the SWN-20: Indication that subjective well-being is associated with momentary affective states above and beyond psychosis susceptibility. *Psychiatry Research*. 2017;258:234-238. doi:10.1016/j.psychres.2017.08.017

296. Pot-Kolder R, Veling W, Counotte J, van der Gaag M. Self-reported Cognitive Biases Moderate the Associations Between Social Stress and Paranoid Ideation in a Virtual Reality Experimental Study. *Schizophrenia Bulletin*. Published online October 10, 2017. doi:10.1093/schbul/sbx119

297. Poustka L, Parzer P, Brunner R, Resch F. Basic symptoms, temperament and character in adolescent psychiatric disorders. *Psychopathology*. 2007;40(5):321-328. doi:10.1159/000105530

298. Power L, Polari AR, Yung AR, McGorry PD, Nelson B. Distress in relation to attenuated psychotic symptoms in the ultra-high-risk population is not associated with increased risk of psychotic disorder. *Early Intervention in Psychiatry*. 2016;10(3):258-262. doi:10.1111/eip.12233

299. Preti A, Pisano A, Cascio MT, Monzani E, Meneghelli A, Cocchi A. Obstetric complications in early psychosis: Relation with family history of psychosis. *Psychiatry Research*. 2012;200(2-3):708-714. doi:10.1016/j.psychres.2012.07.013

300. Preti A, Pisano A, Cascio MT, et al. Validation of the Health of the Nation Outcome Scales as a routine measure of outcome in early intervention programmes. *Early Intervention in Psychiatry*. 2012;6(4):423-431. doi:10.1111/j.1751-7893.2011.00329.x

301. Preti A, Raballo A, Meneghelli A, et al. Antipsychotics are related to psychometric conversion to psychosis in ultra-high-risk youth. *Early Intervention in Psychiatry*. Published online 2021. doi:10.1111/eip.13158

302. Provenzano FA, Guo J, Wall MM, et al. Hippocampal Pathology in Clinical High-Risk Patients and the Onset of Schizophrenia. *Biological Psychiatry*. 2020;87(3):234-242. doi:10.1016/j.biopsych.2019.09.022

303. Pruessner M, Béchard-Evans L, Boekestyn L, Iyer SN, Pruessner JC, Malla AK. Attenuated cortisol response to acute psychosocial stress in individuals at ultra-high risk for psychosis. *Schizophrenia Research*. 2013;146(1-3):79-86. doi:10.1016/j.schres.2013.02.019

304. Pruessner M, Faridi K, Shah J, et al. The Clinic for Assessment of Youth at Risk (CAYR): 10 years of service delivery and research targeting the prevention of psychosis in Montreal, Canada. *Early Intervention in Psychiatry*. 2017;11(2):177-184. doi:10.1111/eip.12300

305. Pruessner M, Bechard-Evans L, Pira S, et al. Interplay of hippocampal volume and hypothalamus-pituitary-adrenal axis function as markers of stress vulnerability in men at ultra-high risk for psychosis. *Psychological Medicine*. 2017;47(3):471-483. doi:10.1017/S0033291716002658

306. Pu C, Wang Y, Zheng H, et al. Altered cerebellocerebral structural covariance in individuals with attenuated psychosis syndrome. *Asian Journal of Psychiatry*. 2020;53. doi:10.1016/j.ajp.2020.102238

307. Pukrop R, Schultze-Lutter F, Ruhrmann S, et al. Neurocognitive functioning in subjects at risk for a first episode of psychosis compared with first- and multiple-episode schizophrenia. *Journal of Clinical and Experimental Neuropsychology*. 2006;28(8):1388-1407. doi:10.1080/13803390500434425

308. Pukrop R, Ruhrmann S, Schultze-Lutter F, Bechdolf A, Brockhaus-Dumke A, Klosterkötter J. Neurocognitive indicators for a conversion to psychosis: Comparison of patients in a potentially initial prodromal state who did or did not convert to a psychosis. *Schizophrenia Research*. 2007;92(1-3):116-125. doi:10.1016/j.schres.2007.01.020

309. Pulkkinen J, Nikkinen J, Kiviniemi V, et al. Functional mapping of dynamic happy and fearful facial expressions in young adults with familial risk for psychosis — Oulu Brain and Mind Study. *Schizophrenia Research*. 2015;164(1-3):242-249. doi:10.1016/j.schres.2015.01.039

310. Purcell R, Harrigan S, Glozier N, Amminger GP, Yung AR. Self reported rates of criminal offending and victimization in young people at-risk for psychosis. *Schizophrenia Research*. 2015;166(1-3):55-59. doi:10.1016/j.schres.2015.05.024

311. Pyle M, Stewart SLK, French P, et al. Internalized stigma, emotional dysfunction and unusual experiences in young people at risk of psychosis. *Early Intervention in Psychiatry*. 2015;9(2):133-140. doi:10.1111/eip.12098

312. Pyle M, Morrison AP. Internalised stereotypes across ultra-high risk of psychosis and psychosis populations. *Psychosis*. 2017;9(2):110-118. doi:10.1080/17522439.2017.1295097

313. Quarmley M, Gur RC, Turetsky BI, et al. Reduced safety processing during aversive social conditioning in psychosis and clinical risk. *Neuropsychopharmacology*. 2019;44(13):2247-2253. doi:10.1038/s41386-019-0421-9

314. Quednow BB, Frommann I, Berning J, Kühn KU, Maier W, Wagner M. Impaired Sensorimotor Gating of the Acoustic Startle Response in the Prodrome of Schizophrenia. *Biological Psychiatry*. 2008;64(9):766-773. doi:10.1016/j.biopsych.2008.04.019

315. Quijada Y, Tizón JL, Artigue J, Parra B. At-risk mental state (ARMS) detection in a community service center for early attention to psychosis in Barcelona. *Early Intervention in Psychiatry*. 2010;4(3). doi:10.1111/j.1751-7893.2010.00192.x

316. Quijada Y, Tizón JL, Artigue J, Parra B. At-risk mental state (ARMS) detection in a community service center for early attention to psychosis in Barcelona. *Early Intervention in Psychiatry*. 2010;4(3):257-262. doi:10.1111/j.1751-7893.2010.00192.x

317. Quijada Y, Tizón JL, Artigue J, Kwapil TR, Barrantes-Vidal N. Attachment style predicts 6-month improvement in psychoticism in persons with at-risk mental states for psychosis. *Early Intervention in Psychiatry*. 2012;6(4):442-449. doi:10.1111/j.1751-7893.2012.00342.x

318. Quijada Y, Kwapil TR, Tizón J, Sheinbaum T, Barrantes-Vidal N. Impact of attachment style on the 1-year outcome of persons with an at-risk mental state for psychosis. *Psychiatry Research*. 2015;228(3):849-856. doi:10.1016/j.psychres.2015.05.013

319. Quiñones GM, Mayeli A, Yushmanov VE, Hetherington HP, Ferrarelli F. Reduced GABA/glutamate in the thalamus of individuals at clinical high risk for psychosis. *Neuropsychopharmacology*. 2021;46(6):1133-1139. doi:10.1038/s41386-020-00920-4

320. Raballo A. Dimensional psychopathology and vulnerability to psychosis: Envisaging the third generation of prodromal/ultra high-risk models. *CNS Spectrums*. 2010;15(7):350-351. doi:10.1017/s109285290000033x

321. Raballo A, Nelson B, Thompson A, Yung A. The Comprehensive Assessment of At-Risk Mental States: From mapping the onset to mapping the structure. *Schizophrenia Research*. 2011;127(1-3):107-114. doi:10.1016/j.schres.2010.12.021

322. Raballo A. Further angles to this story: Time consumption in mind-reading, psychosis risk and phenomenology of social cognition. *Schizophrenia Research*. 2018;197:566-567. doi:10.1016/j.schres.2017.10.038

323. Raballo A, Poletti M, Carpenter WT. Rethinking the Psychosis Threshold in Clinical High Risk. *Schizophrenia Bulletin*. 2019;45(1):1-2. doi:10.1093/schbul/sby149

324. Raballo A, Monducci E, Ferrara M, Fiori Nastro P, Dario C. Developmental vulnerability to psychosis: Selective aggregation of basic self-disturbance in early onset schizophrenia. *Schizophrenia Research*. 2018;201:367-372. doi:10.1016/j.schres.2018.05.012

325. Rahayu A, Keliat BA, Susanti H. The effectiveness of cognitive therapy and family psycho-education on prodromal psychosis and self-esteem of adolescents in orphanages. *Enfermería Clínica*. 2019;29:10-15. doi:10.1016/j.enfcli.2019.04.003

326. Raij TT, Riekki TJJ, Rikandi E, Mäntylä T, Kieseppä T, Suvisaari J. Activation of the motivation-related ventral striatum during delusional experience. *Translational Psychiatry*. 2018;8(1):283. doi:10.1038/s41398-018-0347-8

327. Rajarethinam R, Venkatesh BK, Peethala R, Phan KL, Keshavan M. Reduced activation of superior temporal gyrus during auditory comprehension in young offspring of patients with schizophrenia. *Schizophrenia Research*. 2011;130(1-3):101-105. doi:10.1016/j.schres.2011.05.025

328. Raju V V, Grover S, Nehra R. Social cognitions in siblings of patients with schizophrenia: a comparison with patients with schizophrenia and healthy controls - a cross-sectional study. *Asian Journal of Psychiatry*. 2019;43:24-33. doi:10.1016/j.ajp.2019.04.005

329. Ramyead A, Kometer M, Studerus E, et al. Aberrant Current Source-Density and Lagged Phase Synchronization of Neural Oscillations as Markers for Emerging Psychosis. *Schizophrenia Bulletin*. 2015;41(4):919-929. doi:10.1093/schbul/sbu134

330. Ramyead A, Kometer M, Studerus E, Baumeler D, von Rotz R, Riecher-Rössler A. Alpha oscillations underlie working memory abnormalities in the psychosis high-risk state. *Biological Psychology*. 2017;126:12-18. doi:10.1016/j.biopsycho.2017.04.002

331. Randers L, Jepsen JRM, Fagerlund B, et al. Associations between facial affect recognition and neurocognition in subjects at ultra-high risk for psychosis: A case-control study. *Psychiatry Research*. 2020;290:112969. doi:10.1016/j.psychres.2020.112969

332. Rapado-Castro M, McGorry PD, Yung A, Calvo A, Nelson B. Sources of clinical distress in young people at ultra high risk of psychosis. *Schizophrenia Research*. 2015;165(1):15-21. doi:10.1016/j.schres.2015.03.022

333. Rapado-Castro M, Whittle S, Pantelis C, et al. Does cortical brain morphology act as a mediator between childhood trauma and transition to psychosis in young individuals at ultra-high risk? *Schizophrenia Research*. 2020;224:116-125. doi:10.1016/j.schres.2020.09.017

334. Rapp C, Walter A, Studerus E, et al. Cannabis use and brain structural alterations of the cingulate cortex in early psychosis. *Psychiatry Research: Neuroimaging*. 2013;214(2):102-108. doi:10.1016/j.pscychresns.2013.06.006

335. Rapp C, Studerus E, Bugra H, et al. Duration of untreated psychosis and cognitive functioning. *Schizophrenia Research*. 2013;145(1-3):43-49. doi:10.1016/j.schres.2012.12.016

336. Rapp C, Canela C, Studerus E, et al. Duration of untreated psychosis/illness and brain volume changes in early psychosis. *Psychiatry Research*. 2017;255:332-337. doi:10.1016/j.psychres.2017.06.004

337. Ratheesh A, Cotton SM, Betts JK, et al. Prospective progression from high-prevalence disorders to bipolar disorder: Exploring characteristics of pre-illness stages. *Journal of Affective Disorders*. 2015;183:45-48. doi:10.1016/j.jad.2015.04.025

338. Ratheesh A, Cotton SM, Davey CG, et al. Pre-onset risk characteristics for mania among young people at clinical high risk for psychosis. *Schizophrenia Research*. 2018;192:345-350. doi:10.1016/j.schres.2017.04.036

339. Rauchensteiner S, Kawohl W, Ozgurdal S, et al. Test-performance after cognitive training in persons at risk mental state of schizophrenia and patients with schizophrenia. *Psychiatry Research*. 2011;185(3):334-339. doi:10.1016/j.psychres.2009.09.003

340. Rausch F, Mier D, Eifler S, et al. Reduced activation in the ventral striatum during probabilistic decision-making in patients in an at-risk mental state. *Journal of Psychiatry and Neuroscience*. 2015;40(3):163-173. doi:10.1503/jpn.140191

341. Rausch F, Eisenacher S, Elkin H, et al. Evaluation of the ‘Jumping to conclusions’ bias in different subgroups of the at-risk mental state: from cognitive basic symptoms to UHR criteria. *Psychological Medicine*. 2016;46(10):2071-2081. doi:10.1017/S0033291716000465

342. Razali SM, Othman Z, Abidin ZZ, Mohd Azhar Mohd Yassin. Early Intervention in Psychosis: Efficacy of the Screening Program. *International Medical Journal June* . 2011;18(2):117-120.

343. Razali MS, Abidin Z, Othman Z, mohd yasin mohd azhar. Screening of Genetic Risk among Relatives and the General Public: Exploring the Spectrum of the Psychosis Prodrome. *International Medical Journal (1994)*. 2013;20:747-751. doi:10.5281/zenodo.2596350

344. Razali SM, Abidin ZZ, Othman Z, Yassin MAM. Screening for schizophrenia in initial prodromal phase: Detecting the sub-threshold psychosis. *Asian Journal of Psychiatry*. 2015;16:26-31. doi:10.1016/j.ajp.2015.06.011

345. Raznahan A, Lee Y, Long R, et al. Common functional polymorphisms of DISC1 and cortical maturation in typically developing children and adolescents. *Molecular Psychiatry*. 2011;16(9):917-926. doi:10.1038/mp.2010.72

346. Raznahan A, Greenstein D, Lee Y, et al. Catechol-o-methyl transferase (COMT) val158met polymorphism and adolescent cortical development in patients with childhood-onset schizophrenia, their non-psychotic siblings, and healthy controls. *Neuroimage*. 2011;57(4):1517-1523. doi:10.1016/j.neuroimage.2011.05.032

347. Reeves LE, Anglin DM, Heimberg RG, et al. Anxiety mediates the association between cannabis use and attenuated positive psychotic symptoms. *Psychiatry Research*. 2014;218(1-2):180-186. doi:10.1016/j.psychres.2014.03.040

348. Reichenberg A, Mollon J. Challenges and Opportunities in Studies of Cognition in the Prodrome to Psychosis. *JAMA Psychiatry*. 2016;73(12):1249. doi:10.1001/jamapsychiatry.2016.2655

349. Reininghaus U, Gayer-Anderson C, Valmaggia L, et al. Psychological processes underlying the association between childhood trauma and psychosis in daily life: an experience sampling study. *Psychological Medicine*. 2016;46(13):2799-2813. doi:10.1017/S003329171600146X

350. Reininghaus U, Klippel A, Steinhart H, et al. Efficacy of Acceptance and Commitment Therapy in Daily Life (ACT-DL) in early psychosis: study protocol for a multi-centre randomized controlled trial. *Trials*. 2019;20(1):769. doi:10.1186/s13063-019-3912-4

351. Reniers RLEP, Lin A, Yung AR, et al. Neuroanatomical Predictors of Functional Outcome in Individuals at Ultra-High Risk for Psychosis. *Schizophrenia Bulletin*. Published online July 1, 2016:sbw086. doi:10.1093/schbul/sbw086

352. Resch F, Koch E, Möhler E, Parzer P, Brunner R. Early Detection of Psychotic Disorders in Adolescents: Specificity of Basic Symptoms in Psychiatric Patient Samples. *Psychopathology*. 2002;35(5):259-266. doi:10.1159/000067069

353. Revheim N, Corcoran CM, Dias E, et al. Reading Deficits in Schizophrenia and Individuals at High Clinical Risk: Relationship to Sensory Function, Course of Illness, and Psychosocial Outcome. *American Journal of Psychiatry*. 2014;171(9):949-959. doi:10.1176/appi.ajp.2014.13091196

354. Rice SM, Schäfer MR, Klier C, Mossaheb N, Vijayakumar N, Amminger GP. Erythrocyte polyunsaturated fatty acid levels in young people at ultra-high risk for psychotic disorder and healthy adolescent controls. *Psychiatry Research*. 2015;228(1):174-176. doi:10.1016/j.psychres.2015.04.036

355. Rice SM, McGorry PD, Amminger GP, Nelson B. Current versus recently resolved attenuated psychotic symptoms: Same level of risk for transition to psychosis? *Schizophrenia Research*. 2019;204:450-451. doi:10.1016/j.schres.2018.09.023

356. Rice S, Polari A, Thompson A, Hartmann J, McGorry P, Nelson B. Does reason for referral to an ultra‐high risk clinic predict transition to psychosis? *Early Intervention in Psychiatry*. 2019;13(2):318-321. doi:10.1111/eip.12679

357. Rietdijk J, Dragt S, Klaassen R, et al. A single blind randomized controlled trial of cognitive behavioural therapy in a help-seeking population with an At Risk Mental State for psychosis: The Dutch Early Detection and Intervention Evaluation (EDIE-NL) trial. *Trials*. 2010;11. doi:10.1186/1745-6215-11-30

358. Rietdijk J, Hogerzeil SJ, van Hemert AM, Cuijpers P, Linszen DH, van der Gaag M. Pathways to psychosis: Help-seeking behavior in the prodromal phase. *Schizophrenia Research*. 2011;132(2-3):213-219. doi:10.1016/j.schres.2011.08.009

359. Rietdijk J, Linszen D, van der Gaag M. Field Testing Attenuated Psychosis Syndrome Criteria. *American Journal of Psychiatry*. 2011;168(11):1221-1221. doi:10.1176/appi.ajp.2011.11060859

360. Rietdijk J, Klaassen R, Ising H, et al. Detection of people at risk of developing a first psychosis: comparison of two recruitment strategies. *Acta Psychiatrica Scandinavica*. 2012;126(1):21-30. doi:10.1111/j.1600-0447.2012.01839.x

361. Rietdijk J, Fokkema M, Stahl D, et al. The distribution of self-reported psychotic-like experiences in non-psychotic help-seeking mental health patients in the general population; a factor mixture analysis. *Social Psychiatry and Psychiatric Epidemiology*. 2014;49(3):349-358. doi:10.1007/s00127-013-0772-1

362. Rigucci S, Santi G, Corigliano V, et al. White matter microstructure in ultra-high risk and first episode schizophrenia: A prospective study. *Psychiatry Research: Neuroimaging*. 2016;247:42-48. doi:10.1016/j.pscychresns.2015.11.003

363. Ristanovic I, Damme KSF, DeVylder JE, Schiffman J, Mittal VA. Cannabis use, self‐perceived risk, perceived peer approval and parental attitudes among youth at clinical high‐risk for psychosis. *Early Intervention in Psychiatry*. Published online May 3, 2021. doi:10.1111/eip.13153

364. Roalf DR, de la Garza AG, Rosen A, et al. Alterations in white matter microstructure in individuals at persistent risk for psychosis. *Molecular Psychiatry*. 2020;25(10):2441-2454. doi:10.1038/s41380-019-0360-1

365. Robustelli BL, Newberry RE, Whisman MA, Mittal VA. Social relationships in young adults at ultra high risk for psychosis. *Psychiatry Research*. 2017;247:345-351. doi:10.1016/j.psychres.2016.12.008

366. Roddy S, Tiedt L, Kelleher I, et al. Facial emotion recognition in adolescents with psychotic-like experiences: a school-based sample from the general population. *Psychological Medicine*. 2012;42(10):2157-2166. doi:10.1017/S0033291712000311

367. Roiser JP, Howes OD, Chaddock CA, Joyce EM, McGuire P. Neural and Behavioral Correlates of Aberrant Salience in Individuals at Risk for Psychosis. *Schizophrenia Bulletin*. 2013;39(6):1328-1336. doi:10.1093/schbul/sbs147

368. Roman-Urrestarazu A, Murray GK, Barnes A, et al. Brain structure in different psychosis risk groups in the Northern Finland 1986 Birth Cohort. *Schizophrenia Research*. 2014;153(1-3):143-149. doi:10.1016/j.schres.2013.12.019

369. Rosen M, Haidl TK, Ruhrmann S, Vogeley K, Schultze-Lutter F. Sex differences in symptomatology of psychosis-risk patients and in prediction of psychosis. *Archives of Women’s Mental Health*. 2020;23(3):339-349. doi:10.1007/s00737-019-01000-3

370. Rosenberg K. Early Symptoms may Help Identify Patients at High Risk for Psychosis. *AJN, American Journal of Nursing*. 2019;119(3):50. doi:10.1097/01.NAJ.0000554039.60873.dd

371. Rothlisberger M, Riecher-Rossler A, Aston J, Fusar-Poli P, Radu EW, Borgwardt S. Cingulate Volume Abnormalities in Emerging Psychosis. *Current Pharmaceutical Design*. 2012;18(4). doi:10.2174/138161212799316316

372. Rakhshan Rouhakhtar PJ, Pitts SC, Millman ZB, et al. The impact of age on the validity of psychosis-risk screening in a sample of help-seeking youth. *Psychiatry Research*. 2019;274:30-35. doi:10.1016/j.psychres.2019.02.020

373. Ruff A, McFarlane WR, Downing D, Cook W, Woodberry K. A Community Outreach and Education Model for Early Identification of Mental Illness in Young People. *Adolescent Psychiatrye*. 2012;2(2):140-145. doi:10.2174/2210676611202020140

374. Ruhrmann S, Schultze-Lutter F, Maier W, Klosterkötter J. Pharmacological intervention in the initial prodromal phase of psychosis. *European Psychiatry*. 2005;20(1):1-6. doi:10.1016/j.eurpsy.2004.11.001

375. Ruhrmann S, Bechdolf A, Kühn KU, et al. Acute effects of treatment for prodromal symptoms for people putatively in a late initial prodromal state of psychosis. *British Journal of Psychiatry*. 2007;191(S51). doi:10.1192/bjp.191.51.s88

376. Ruhrmann S, Paruch J, Bechdolf A, et al. Reduced subjective quality of life in persons at risk for psychosis. *Acta Psychiatrica Scandinavica*. 2008;117(5):357-368. doi:10.1111/j.1600-0447.2008.01152.x

377. Ruiz B, Ceccolini CJ, Shah BB, et al. Stigma and coping experiences in Latinx individuals at clinical high-risk for psychosis. *Early Intervention in Psychiatry*. Published online 2021. doi:10.1111/eip.13126

378. Rüsch N, Corrigan PW, Heekeren K, et al. Well-Being Among Persons at Risk of Psychosis: The Role of Self-Labeling, Shame, and Stigma Stress. *Psychiatric Services*. 2014;65(4):483-489. doi:10.1176/appi.ps.201300169

379. Rüsch N, Müller M, Heekeren K, et al. Longitudinal course of self-labeling, stigma stress and well-being among young people at risk of psychosis. *Schizophrenia Research*. 2014;158(1-3):82-84. doi:10.1016/j.schres.2014.07.016

380. Rüsch N, Heekeren K, Theodoridou A, et al. Stigma as a stressor and transition to schizophrenia after one year among young people at risk of psychosis. *Schizophrenia Research*. 2015;166(1-3):43-48. doi:10.1016/j.schres.2015.05.027

381. Russo DA, Stochl J, Croudace TJ, et al. Use of the theory of planned behaviour to assess factors influencing the identification of individuals at ultra‐high risk for psychosis in primary care. *Early Intervention in Psychiatry*. 2012;6(3):265-275. doi:10.1111/j.1751-7893.2011.00296.x

382. Rutigliano G, Merlino S, Minichino A, et al. Long term outcomes of acute and transient psychotic disorders: The missed opportunity of preventive interventions. *European Psychiatry*. 2018;52:126-133. doi:10.1016/j.eurpsy.2018.05.004

383. Ryan A. Psychopathological syndromes and familial morbid risk of psychosis. *British Journal of Psychiatry*. 1997;171(3). doi:10.1192/bjp.171.3.289a

384. Ryan AT, Addington J, Bearden CE, et al. Latent class cluster analysis of symptom ratings identifies distinct subgroups within the clinical high risk for psychosis syndrome. *Schizophrenia Research*. 2018;197:522-530. doi:10.1016/j.schres.2017.12.001

385. Saarinen A, Lieslehto J, Kiviniemi V, Tuovinen T, Veijola J, Hintsanen M. The relationship of genetic susceptibilities for psychosis with physiological fluctuation in functional MRI data. *Psychiatry Research: Neuroimaging*. 2020;297:111031. doi:10.1016/j.pscychresns.2020.111031

386. Şahin S, Yüksel Ç, Güler J, et al. The history of childhood trauma among individuals with ultra high risk for psychosis is as common as among patients with first-episode schizophrenia. *Early Intervention in Psychiatry*. 2013;7(4):414-420. doi:10.1111/eip.12022

387. Saito J, Hori M, Nemoto T, et al. Longitudinal study examining abnormal white matter integrity using a tract-specific analysis in individuals with a high risk for psychosis. *Psychiatry and Clinical Neurosciences*. 2017;71(8):530-541. doi:10.1111/pcn.12515

388. Saito J, Nemoto T, Katagiri N, et al. Can reduced leftward asymmetry of white matter integrity be a marker of transition to psychosis in at-risk mental state? *Asian Journal of Psychiatry*. 2020;54. doi:10.1016/j.ajp.2020.102450

389. Sakuma A, Obara C, Katsura M, et al. No regional gray matter volume reduction observed in young Japanese people at ultra-high risk for psychosis: A voxel-based morphometry study. *Asian Journal of Psychiatry*. 2018;37:167-171. doi:10.1016/j.ajp.2018.09.009

390. Saleem MM, Stowkowy J, Cadenhead KS, et al. Perceived discrimination in those at clinical high risk for psychosis. *Early Intervention Psychiatry*. 2014;Feb;8(1):77-81.

391. Salokangas RKR, Heinimaa M, Svirskis T, et al. Perceived negative attitude of others as an early sign of psychosis. *European Psychiatry*. 2009;24(4):233-238. doi:10.1016/j.eurpsy.2008.12.011

392. Salokangas RKR, Patterson P, Heinimaa M, et al. Perceived negative attitude of others predicts transition to psychosis in patients at risk of psychosis. *European Psychiatry*. 2012;27(4):264-266. doi:10.1016/j.eurpsy.2010.11.004

393. Salokangas RKR, Patterson P, Heinimaa M, et al. Perceived negative attitude of others predicts transition to psychosis in patients at risk of psychosis. *European Psychiatry*. 2012;27(4):264-266. doi:10.1016/j.eurpsy.2010.11.004

394. Salokangas RKR, Heinimaa M, From T, et al. Short-term functional outcome and premorbid adjustment in clinical high-risk patients. Results of the EPOS project. *European Psychiatry*. 2014;29(6). doi:10.1016/j.eurpsy.2013.10.003

395. Salokangas RKR, Hietala J, Armio RL, et al. Effect of childhood physical abuse on social anxiety is mediated via reduced frontal lobe and amygdala-hippocampus complex volume in adult clinical high-risk subjects. *Schizophrenia Research*. 2021;227:101-109. doi:10.1016/j.schres.2020.05.041

396. Sanada K, de Azúa SR, Nakajima S, et al. Correlates of neurocognitive functions in individuals at ultra-high risk for psychosis - A 6-month follow-up study. *Psychiatry Research*. 2018;268:1-7. doi:10.1016/j.psychres.2018.06.053

397. Santoro ML, Gadelha A, Ota VK, et al. Gene expression analysis in blood of ultra-high risk subjects compared to first-episode of psychosis patients and controls. *The World Journal of Biological Psychiatry*. 2015;16(6):441-446. doi:10.3109/15622975.2015.1048724

398. Sarac C, Bilgrami ZR, Haas SS, et al. Processing speed and brain volume in individuals at clinical high-risk for psychosis with comorbid eating disorders: A brief report. *Schizophrenia Research*. 2021;227:92-94. doi:10.1016/j.schres.2020.08.025

399. Sasabayashi D, Takayanagi Y, Takahashi T, et al. Increased Occipital Gyrification and Development of Psychotic Disorders in Individuals With an At-Risk Mental State: A Multicenter Study. *Biological Psychiatry*. 2017;82(10):737-745. doi:10.1016/j.biopsych.2017.05.018

400. Sasabayashi D, Takayanagi Y, Takahashi T, et al. Subcortical brain volume abnormalities in individuals with an at-risk mental state. *Schizophrenia Bulletin*. 2020;46(4):834-845. doi:10.1093/schbul/sbaa011

401. Sawada K, Kanehara A, Sakakibara E, et al. Identifying neurocognitive markers for outcome prediction of global functioning in individuals with first-episode and ultra-high-risk for psychosis. *Psychiatry and Clinical Neurosciences*. 2017;71(5):318-327. doi:10.1111/pcn.12522

402. Scala S, Pousada A, Stone WS, et al. Verbal and visual–spatial memory impairment in youth at familial risk for schizophrenia or affective psychosis: A pilot study. *Schizophrenia Research*. 2013;144(1-3):122-128. doi:10.1016/j.schres.2012.11.027

403. Scazza I, Pelizza L, Azzali S, et al. Reliability of the Italian version of the Brief (21-item) Prodromal Questionnaire (IPQ-B) for psychosis risk screening in a young help-seeking population. *Journal of psychopathology*. 2018;24:204-214.

404. Scheyer R, Reznik N, Apter A, Seidman LJ, Koren D. Metacognition in non-psychotic help-seeking adolescents: associations with prodromal symptoms, distress and psychosocial deterioration. *Isr J Psychiatry Relat Sci*. 2014;51(1).

405. Schifani C, Tseng HH, Kenk M, et al. Cortical stress regulation is disrupted in schizophrenia but not in clinical high risk for psychosis. *Brain*. 2018;141(7):2213-2224. doi:10.1093/brain/awy133

406. Schifani C, Hafizi S, Tseng HH, et al. Preliminary data indicating a connection between stress-induced prefrontal dopamine release and hippocampal TSPO expression in the psychosis spectrum. *Schizophrenia Research*. 2019;213:80-86. doi:10.1016/j.schres.2018.10.008

407. Schifani C, Pruessner J, Tseng HH, et al. Stress-induced cortical dopamine response is altered in subjects at clinical high risk for psychosis using cannabis. *Addiction Biology*. 2020;25(4). doi:10.1111/adb.12812

408. Schiffman J. Motor Issues in the Clinical High Risk Phase of Psychosis. *Schizophrenia Bulletin*. 2017;43(5):937-938. doi:10.1093/schbul/sbx086

409. Schimmelmann BG, Michel C, Schaffner N, Schultze-Lutter F. What percentage of people in the general population satisfies the current clinical at-risk criteria of psychosis? *Schizophrenia Research*. 2011;125(1):99-100. doi:10.1016/j.schres.2010.09.018

410. Schlosser DA, Zinberg JL, Loewy RL, et al. Predicting the longitudinal effects of the family environment on prodromal symptoms and functioning in patients at-risk for psychosis. *Schizophrenia Research*. 2010;118(1-3):69-75. doi:10.1016/j.schres.2010.01.017

411. Schlosser DA, Zinberg JL, Loewy RL, et al. Predicting the longitudinal effects of the family environment on prodromal symptoms and functioning in patients at-risk for psychosis. *Schizophrenia Research*. 2010;118(1-3):69-75. doi:10.1016/j.schres.2010.01.017

412. Schlosser DA, Miklowitz DJ, O’Brien MP, de Silva SD, Zinberg JL, Cannon TD. A randomized trial of family focused treatment for adolescents and young adults at risk for psychosis: study rationale, design and methods. *Early Intervention in Psychiatry*. 2012;6(3):283-291. doi:10.1111/j.1751-7893.2011.00317.x

413. Schlosser DA, Fisher M, Gard D, Fulford D, Loewy RL, Vinogradov S. Motivational deficits in individuals at-risk for psychosis and across the course of schizophrenia. *Schizophrenia Research*. 2014;158(1-3):52-57. doi:10.1016/j.schres.2014.06.024

414. Schlosser DA, Campellone TR, Biagianti B, et al. Modeling the role of negative symptoms in determining social functioning in individuals at clinical high risk of psychosis. *Schizophrenia Research*. 2015;169(1-3):204-208. doi:10.1016/j.schres.2015.10.036

415. Schmidt A, Smieskova R, Aston J, et al. Brain Connectivity Abnormalities Predating the Onset of Psychosis. *JAMA Psychiatry*. 2013;70(9):903. doi:10.1001/jamapsychiatry.2013.117

416. Schmidt A, Smieskova R, Simon A, et al. Abnormal effective connectivity and psychopathological symptoms in the psychosis high-risk state. *Journal of Psychiatry and Neuroscience*. 2014;39(4):239-248. doi:10.1503/jpn.130102

417. Schmidt A, Lenz C, Smieskova R, et al. Brain Diffusion Changes in Emerging Psychosis and the Impact of State-Dependent Psychopathology. *Neurosignals*. 2015;23(1):71-83. doi:10.1159/000442605

418. Schmidt A, Crossley NA, Harrisberger F, et al. Structural Network Disorganization in Subjects at Clinical High Risk for Psychosis. *Schizophrenia Bulletin*. Published online August 1, 2016:sbw110. doi:10.1093/schbul/sbw110

419. Schmidt A, Antoniades M, Allen P, et al. Longitudinal alterations in motivational salience processing in ultra-high-risk subjects for psychosis. *Psychological Medicine*. 2017;47(2):243-254. doi:10.1017/S0033291716002439

420. Schmidt SJ, Hurlemann R, Schultz J, et al. Multimodal prevention of first psychotic episode through N‐acetyl‐ <scp>l</scp> ‐cysteine and integrated preventive psychological intervention in individuals clinically at high risk for psychosis: Protocol of a randomized, placebo‐controlled, parallel‐group trial. *Early Intervention in Psychiatry*. 2019;13(6):1404-1415. doi:10.1111/eip.12781

421. Schneider M, van der Linden M, Glaser B, et al. Preliminary structure and predictive value of attenuated negative symptoms in 22q11.2 deletion syndrome. *Psychiatry Research*. 2012;196(2-3):277-284. doi:10.1016/j.psychres.2011.08.017

422. Schneider M, Schaer M, Mutlu AK, et al. Clinical and cognitive risk factors for psychotic symptoms in 22q11.2 deletion syndrome: A transversal and longitudinal approach. *European Child and Adolescent Psychiatry*. 2014;23(6):425-436. doi:10.1007/s00787-013-0469-8

423. Schneider M, Armando M, Schultze-Lutter F, et al. Prevalence, course and psychosis-predictive value of negative symptoms in 22q11.2 deletion syndrome. *Schizophrenia Research*. 2019;206:386-393. doi:10.1016/j.schres.2018.10.014

424. Schofield P, Ashworth M, Jones R. Ethnic isolation and psychosis: re-examining the ethnic density effect. *Psychological Medicine*. 2011;41(6):1263-1269. doi:10.1017/S0033291710001649

425. Schubert EW, McNeil TF. Prospective Study of Neurological Abnormalities in Offspring of Women With Psychosis: Birth to Adulthood. *American Journal of Psychiatry*. 2004;161(6):1030-1037. doi:10.1176/appi.ajp.161.6.1030

426. Schubert EW, McNeil TF. Neuropsychological Impairment and Its Neurological Correlates in Adult Offspring With Heightened Risk for Schizophrenia and Affective Psychosis. *American Journal of Psychiatry*. 2005;162(4):758-766. doi:10.1176/appi.ajp.162.4.758

427. Schubert EW, McNeil TF. Neurobehavioral deficits in young adult offspring with heightened risk for psychosis who developed schizophrenia-spectrum disorder. *Schizophrenia Research*. 2007;94(1-3):107-113. doi:10.1016/j.schres.2007.05.015

428. Schultze-Lutter F, Klosterkötter J, Ruhrmann S. *Predicting First-Episode Psychosis by Basic Symptom Criteria*. https://www.researchgate.net/publication/228483398

429. Schultze-Lutter F, Ruhrmann S, Hoyer C, Klosterkotter J, Leweke FM. The initial syndrome of schizophrenia: Different duration, different underlying deficits? *Comprehensive Psychiatry*. 2007;48(5):479-488.

430. Schultze-Lutter F, Klosterkötter J. *The Dimensional Structure of Self-Reported ’prodromal’disturbances in Schizophrenia Psychotic Risk Detection in Children and Adolescents View Project Schizotypy Research View Project*.; 2008. https://www.researchgate.net/publication/228483396

431. Schultze-Lutter F, Ruhrmann S, Klosterkötter J. Early detection of psychosis – Establishing a service for persons at risk. *European Psychiatry*. 2009;24(1):1-10. doi:10.1016/j.eurpsy.2008.08.004

432. Schultze-Lutter F, Ruhrmann S, Berning J, Maier W, Klosterkotter J. Basic Symptoms and Ultrahigh Risk Criteria: Symptom Development in the Initial Prodromal State. *Schizophrenia Bulletin*. 2010;36(1):182-191. doi:10.1093/schbul/sbn072

433. Schultze-Lutter F, Schimmelmann BG, Klosterkötter J, Ruhrmann S. Comparing the prodrome of schizophrenia-spectrum psychoses and affective disorders with and without psychotic features. *Schizophrenia Research*. 2012;138(2-3):218-222. doi:10.1016/j.schres.2012.04.001

434. Schultze-Lutter F, Michel C, Ruhrmann S, Schimmelmann BG. Prevalence and Clinical Significance of DSM-5–Attenuated Psychosis Syndrome in Adolescents and Young Adults in the General Population: The Bern Epidemiological At-Risk (BEAR) Study. *Schizophrenia Bulletin*. 2014;40(6). doi:10.1093/schbul/sbt171

435. Schultze-Lutter F, Klosterkötter J, Ruhrmann S. Improving the clinical prediction of psychosis by combining ultra-high risk criteria and cognitive basic symptoms. *Schizophrenia Research*. 2014;154(1-3):100-106. doi:10.1016/j.schres.2014.02.010

436. Schultze-Lutter F, Renner F, Paruch J, Julkowski D, Klosterkötter J, Ruhrmann S. Self-Reported Psychotic-Like Experiences Are a Poor Estimate of Clinician-Rated Attenuated and Frank Delusions and Hallucinations. *Psychopathology*. 2014;47(3). doi:10.1159/000355554

437. Schultze-Lutter F, Klosterkötter J, Nikolaides A, Ruhrmann S. Personality dimensions in persons symptomatically at risk of psychosis: pronounced but lacking a characteristic profile. *Early Intervention in Psychiatry*. 2015;9(3):242-247. doi:10.1111/eip.12147

438. Schultze-Lutter F, Michel C, Ruhrmann S, Schimmelmann BG. Prevalence and clinical relevance of interview-assessed psychosis-risk symptoms in the young adult community. *Psychological Medicine*. 2018;48(7):1167-1178. doi:10.1017/S0033291717002586

439. Schultze-Lutter F, Ruhrmann S, Michel C, Kindler J, Schimmelmann BG, Schmidt SJ. Age effects on basic symptoms in the community: A route to gain new insight into the neurodevelopment of psychosis? *European Archives of Psychiatry and Clinical Neuroscience*. 2020;270(3):311-324. doi:10.1007/s00406-018-0949-4

440. Seidman LJ. Neuropsychology of the Prodrome to Psychosis in the NAPLS Consortium&lt;subtitle&gt;Relationship to Family History and Conversion to Psychosis&lt;/subtitle&gt;&lt;alt-title&gt;Neuropsychology of Prodrome to Psychosis&lt;/alt-title&gt; *Archives of General Psychiatry*. 2010;67(6):578. doi:10.1001/archgenpsychiatry.2010.66

441. Seidman LJ, Shapiro DI, Stone WS, et al. Association of Neurocognition With Transition to Psychosis. *JAMA Psychiatry*. 2016;73(12):1239. doi:10.1001/jamapsychiatry.2016.2479

442. Seiferth NY, Pauly K, Habel U, et al. Increased neural response related to neutral faces in individuals at risk for psychosis. *Neuroimage*. 2008;40(1). doi:10.1016/j.neuroimage.2007.11.020

443. Seiler N, Maguire J, Nguyen T, et al. Prevalence of subthreshold positive symptoms in young people without psychotic disorders presenting to a youth mental health service. *Schizophrenia Research*. 2020;215:446-448. doi:10.1016/j.schres.2019.10.041

444. Selten JP, Termorshuizen F. “Ethnic density of neighbourhood at age 15 modifies the risk for psychosis”. So what? *Schizophrenia Research*. 2017;190:88-89. doi:10.1016/j.schres.2017.11.002

445. Selvaraj S, Bloomfield PS, Cao B, Veronese M, Turkheimer F, Howes OD. Brain TSPO imaging and gray matter volume in schizophrenia patients and in people at ultra high risk of psychosis: An [11C]PBR28 study. *Schizophrenia Research*. 2018;195:206-214. doi:10.1016/j.schres.2017.08.063

446. Severaid KB, Osborne KJ, Mittal VA. Implications of religious and spiritual practices for youth at clinical high risk for psychosis. *Schizophrenia Research*. 2019;208:481-482. doi:10.1016/j.schres.2019.01.033

447. Shah JL, Tandon N, Montrose DM, et al. Clinical psychopathology in youth at familial high risk for psychosis. *Early Intervention in Psychiatry*. 2019;13(2):297-303. doi:10.1111/eip.12480

448. Shaikh M, Valmaggia L, Broome MR, et al. Reduced mismatch negativity predates the onset of psychosis. *Schizophrenia Research*. 2012;134(1):42-48. doi:10.1016/j.schres.2011.09.022

449. Shaikh M, Dutt A, Broome MR, et al. Sensory gating deficits in the attenuated psychosis syndrome. *Schizophrenia Research*. 2015;161(2-3):277-282. doi:10.1016/j.schres.2014.12.021

450. Shaikh M, Ellett L, Dutt A, et al. Perceived ethnic discrimination and persecutory paranoia in individuals at ultra-high risk for psychosis. *Psychiatry Research*. 2016;241:309-314. doi:10.1016/j.psychres.2016.05.006

451. Shakeel MK, Lu L, Cannon TD, et al. Longitudinal changes in social cognition in individuals at clinical high risk for psychosis: An outcome based analysis. *Schizophrenia Research*. 2019;204:334-336. doi:10.1016/j.schres.2018.08.032

452. Shakeel MK, MacQueen G, Addington J, et al. White Matter Connectivity in Youth at Risk for Serious Mental Illness: A Longitudinal Analysis. *Psychiatry Research: Neuroimaging*. 2020;302:111106. doi:10.1016/j.pscychresns.2020.111106

453. Shakeel MK, Lu L, Woods SW, Perkins DO, Addington J. Neurocognitive predictors of metacognition in individuals at clinical high risk for psychosis. *Behavioural and Cognitive Psychotherapy*. 2020;48(2):248-252. doi:10.1017/S1352465819000328

454. Shakory S, Watts JJ, Hafizi S, et al. Hippocampal glutamate metabolites and glial activation in clinical high risk and first episode psychosis. *Neuropsychopharmacology*. 2018;43(11):2249-2255. doi:10.1038/s41386-018-0163-0

455. Shan X, Ou Y, Pan P, et al. Increased frontal gray matter volume in individuals with prodromal psychosis. *CNS Neuroscience & Therapeutics*. 2019;25(9):987-994. doi:10.1111/cns.13143

456. Shapiro DI, Cubells JF, Ousley OY, Rockers K, Walker EF. Prodromal symptoms in adolescents with 22q11.2 deletion syndrome and schizotypal personality disorder. *Schizophrenia Research*. 2011;129(1):20-28. doi:10.1016/j.schres.2011.03.030

457. Shapiro DI. *The Relationship between Cortisol and Cognitive Functions in Individuals at Clunical High-Risk of Developing Psychosis*. 2015.

458. Sharma A, Wolf DH, Ciric R, et al. Common Dimensional Reward Deficits Across Mood and Psychotic Disorders: A Connectome-Wide Association Study. *American Journal of Psychiatry*. 2017;174(7):657-666. doi:10.1176/appi.ajp.2016.16070774

459. Sheinbaum T, Bifulco A, Ballespí S, Mitjavila M, Kwapil TR, Barrantes-Vidal N. Interview Investigation of Insecure Attachment Styles as Mediators between Poor Childhood Care and Schizophrenia-Spectrum Phenomenology. *PLOS ONE*. 2015;10(8):e0135150. doi:10.1371/journal.pone.0135150

460. Shetty JJ, Nicholas C, Nelson B, et al. Greater preference for eveningness is associated with negative symptoms in an ultra-high risk for psychosis sample. *Early Intervention in Psychiatry*. Published online 2021. doi:10.1111/eip.13112

461. Houston JE, Murphy J, Adamson G, Stringer M, Shevlin M. Childhood sexual abuse, early cannabis use, and psychosis: Testing an interaction model based on the national comorbidity survey. *Schizophrenia Bulletin*. 2008;34(3):580-585. doi:10.1093/schbul/sbm127

462. Shi J, Wang L, Yao Y, et al. Protective factors in Chinese university students at clinical high risk for psychosis. *Psychiatry Research*. 2016;239:239-244. doi:10.1016/j.psychres.2016.03.036

463. Shim GS, Kang DH, Chung YS, Yoo SY, Shin NY, Kwon JS. Social functioning deficits in young people at risk for schizophrenia. *Australian and New Zealand Journal of Psychiatry*. 2008;42(8):678-685. doi:10.1080/00048670802203459

464. Shim GS, Kang DH, Choi JS, et al. Prospective outcome of early intervention for individuals at ultra-high-risk for psychosis. *Early Intervention in Psychiatry*. 2008;2(4):277-284. doi:10.1111/j.1751-7893.2008.00089.x

465. Shim G, Oh JS, Jung WH, et al. Altered resting-state connectivity in subjects at ultra-high risk for psychosis: an fMRI study. *Behavioral and Brain Functions*. 2010;6(1):58. doi:10.1186/1744-9081-6-58

466. Shin KS, Kim JS, Kang DH, et al. Pre-Attentive Auditory Processing in Ultra-High-Risk for Schizophrenia with Magnetoencephalography. *Biological Psychiatry*. 2009;65(12):1071-1078. doi:10.1016/j.biopsych.2008.12.024

467. Shin YM, Jung HY, Kim SW, et al. A descriptive study of pathways to care of high risk for psychosis adolescents in Korea. *Early Intervention in Psychiatry*. 2010;4(2):119-123. doi:10.1111/j.1751-7893.2010.00180.x

468. Shin KS, Jung WH, Kim JS, et al. Neuromagnetic auditory response and its relation to cortical thickness in ultra-high-risk for psychosis. *Schizophrenia Research*. 2012;140(1-3):93-98. doi:10.1016/j.schres.2012.06.014

469. Shin KS, Kim JS, Kim SN, et al. Aberrant Auditory Processing in Schizophrenia and in Subjects at Ultra-High-Risk for Psychosis. *Schizophrenia Bulletin*. 2012;38(6):1258-1267. doi:10.1093/schbul/sbr138

470. Shin YS, Kim SN, Shin NY, et al. Correction: Increased intra-individual variability of cognitive processing in subjects at risk mental state and schizophrenia patients. *PLoS ONE*. 2016;11(5). doi:10.1371/journal.pone.0155573

471. Shin YS, Kim SY, Lee TY, et al. Longitudinal change in neurocognition and its relation to symptomatic and functional changes over 2 years in individuals at clinical high-risk for psychosis. *Schizophrenia Research*. 2016;174(1-3):50-57. doi:10.1016/j.schres.2016.03.024

472. Sichlinger L, Cibelli E, Goldrick M, Mittal VA. Clinical correlates of aberrant conversational turn-taking in youth at clinical high-risk for psychosis. *Schizophrenia Research*. 2019;204:419-420. doi:10.1016/j.schres.2018.08.009

473. Siira V, Wahlberg KE, Miettunen J, Lasky K, Pekka Tienari PT. Psychometric Deviance Measured by MMPI in Adoptees at High Risk for Schizophrenia and Their Adoptive Controls. *Journal of Personality Assessment*. 2004;83(1):14-21. doi:10.1207/s15327752jpa8301_02

474. Silverstein S, Uhlhaas PJ, Essex B, Halpin S, Schall U, Carr V. Perceptual organization in first episode schizophrenia and ultra-high-risk states. *Schizophrenia Research*. 2006;83(1):41-52. doi:10.1016/j.schres.2006.01.003

475. Simeonova DI. *Social and Behavioral Precursors of Conversion to Psychosis: An Investigation of Youth at Risk for Psychosis.* PhD Dissertation Thesis. Laney Graduate School; 2009.

476. Simeonova DI, Attalla A, Trotman H, Esterberg M, Walker EF. Does a parent-report measure of behavioral problems enhance prediction of conversion to psychosis in clinical high-risk adolescents? *Schizophrenia Research*. 2011;130(1-3):157-163. doi:10.1016/j.schres.2011.03.034

477. Simeonova DI, Nguyen T, Walker EF. Psychosis risk screening in clinical high-risk adolescents: A longitudinal investigation using the Child Behavior Checklist. *Schizophrenia Research*. 2014;159(1):7-13. doi:10.1016/j.schres.2014.07.046

478. Simon AE, Cattapan-Ludewig K, Gruber K, et al. Subclinical hallucinations in adolescent outpatients: An outcome study. *Schizophrenia Research*. 2009;108(1-3):265-271. doi:10.1016/j.schres.2008.12.018

479. Simon AE, Umbricht D. Is Elevated Striatal Dopamine Function a Prodromal Sign of Schizophrenia? *Archives of General Psychiatry*. 2009;66(8):915. doi:10.1001/archgenpsychiatry.2009.100

480. Simon AE, Umbricht D, Lang UE, Borgwardt S. Declining transition rates to psychosis: The role of diagnostic spectra and symptom overlaps in individuals with attenuated psychosis syndrome. *Schizophrenia Research*. 2014;159(2-3). doi:10.1016/j.schres.2014.09.016

481. Sinclair-McBride K, Morelli N, Tembulkar S, Graber K, Gonzalez-Heydrich J, D’Angelo EJ. Young children with psychotic symptoms and risk for suicidal thoughts and behaviors: a research note. *BMC Research Notes*. 2018;11(1):568. doi:10.1186/s13104-018-3680-3

482. Singh SP, Winsper C, Wolke D, Bryson A. School Mobility and Prospective Pathways to Psychotic-like Symptoms in Early Adolescence: A Prospective Birth Cohort Study. *Journal of the American Academy of Child & Adolescent Psychiatry*. 2014;53(5). doi:10.1016/j.jaac.2014.01.016

483. Slavutskaya M v., Lebedeva IS, Karelin SA, et al. Characteristics of Saccadic Responses in an Experimental “Go/No Go” Scheme in Healthy Subjects and Those at Ultra High Risk of Developing Schizophrenia. *Neuroscience and Behavioral Physiology*. 2018;48(3):261-266. doi:10.1007/s11055-018-0559-7

484. Smesny S, Milleit B, Hipler UC, et al. Omega-3 fatty acid supplementation changes intracellular phospholipase A2 activity and membrane fatty acid profiles in individuals at ultra-high risk for psychosis. *Molecular Psychiatry*. 2014;19(3):317-324. doi:10.1038/mp.2013.7

485. Smesny S, Milleit B, Schaefer MR, et al. Effects of omega-3 PUFA on the vitamin E and glutathione antioxidant defense system in individuals at ultra-high risk of psychosis. *Prostaglandins, Leukotrienes and Essential Fatty Acids*. 2015;101:15-21. doi:10.1016/j.plefa.2015.07.001

486. Smesny S, Milleit B, Schaefer MR, et al. Effects of omega-3 PUFA on immune markers in adolescent individuals at ultra-high risk for psychosis – Results of the randomized controlled Vienna omega-3 study. *Schizophrenia Research*. 2017;188:110-117. doi:10.1016/j.schres.2017.01.026

487. Smieskova R, Allen P, Simon A, et al. Different duration of at-risk mental state associated with neurofunctional abnormalities. A multimodal imaging study. *Human Brain Mapping*. 2012;33(10):2281-2294. doi:10.1002/hbm.21360

488. Smieskova R, Fusar-Poli P, Aston J, et al. Insular volume abnormalities associated with different transition probabilities to psychosis. *Psychological Medicine*. 2012;42(8):1613-1625. doi:10.1017/S0033291711002716

489. Smieskova R, Roiser JP, Chaddock CA, et al. Modulation of motivational salience processing during the early stages of psychosis. *Schizophrenia Research*. 2015;166(1-3):17-23. doi:10.1016/j.schres.2015.04.036

490. Söder E, Clamor A, Lincoln TM. Hair cortisol concentrations as an indicator of potential HPA axis hyperactivation in risk for psychosis. *Schizophrenia Research*. 2019;212:54-61. doi:10.1016/j.schres.2019.08.012

491. Söder E, Krkovic K, Lincoln TM. The relevance of chronic stress for the acute stress reaction in people at elevated risk for psychosis. *Psychoneuroendocrinology*. 2020;119:104684. doi:10.1016/j.psyneuen.2020.104684

492. Solé-Padullés C, Castro-Fornieles J, de la Serna E, et al. Intrinsic functional connectivity of fronto-temporal networks in adolescents with early psychosis. *European Child & Adolescent Psychiatry*. 2017;26(6):669-679. doi:10.1007/s00787-016-0931-5

493. Solís-Vivanco R, Mondragón-Maya A, León-Ortiz P, Rodríguez-Agudelo Y, Cadenhead KS, de la Fuente-Sandoval C. Mismatch Negativity reduction in the left cortical regions in first-episode psychosis and in individuals at ultra high-risk for psychosis. *Schizophrenia Research*. 2014;158(1-3):58-63. doi:10.1016/j.schres.2014.07.009

494. Sollychin M, Jack BN, Polari A, et al. Frontal slow wave resting EEG power is higher in individuals at Ultra High Risk for psychosis than in healthy controls but is not associated with negative symptoms or functioning. *Schizophrenia Research*. 2019;208:293-299. doi:10.1016/j.schres.2019.01.039

495. Solmi M, Campeol M, Gentili F, Favaro A, Cremonese C. Clinical presentation and need for treatment of a cohort of subjects accessing to a mental illness prevention service. *Research in Psychotherapy: Psychopathology, Process and Outcome*. 2020;23(1). doi:10.4081/ripppo.2020.434

496. Solomon M, Olsen E, Niendam T, et al. From lumping to splitting and back again: Atypical social and language development in individuals with clinical-high-risk for psychosis, first episode schizophrenia, and autism spectrum disorders. *Schizophrenia Research*. 2011;131(1-3):146-151. doi:10.1016/j.schres.2011.03.005

497. Souaiby L, Gauthier C, Kazes M, et al. Individual factors influencing the duration of untreated psychosis. *Early Intervention in Psychiatry*. 2019;13(4):798-804. doi:10.1111/eip.12562

498. Soyata AZ, Akışık S, İnhanlı D, Noyan H, Üçok A. Relationship of obsessive-compulsive symptoms to clinical variables and cognitive functions in individuals at ultra high risk for psychosis. *Psychiatry Research*. 2018;261:332-337. doi:10.1016/j.psychres.2018.01.004

499. Spauwen J, Krabbendam L, Lieb R, Wittchen HU, van Os J. Does urbanicity shift the population expression of psychosis? *Journal of Psychiatric Research*. 2004;38(6):613-618. doi:10.1016/j.jpsychires.2004.04.003

500. Spauwen J, Krabbendam L, Lieb R, Wittchen HU, Os J. Early maternal stress and health behaviours and offspring expression of psychosis in adolescence. *Acta Psychiatrica Scandinavica*. 2004;110(5):356-364. doi:10.1111/j.1600-0447.2004.00429.x

501. Spauwen J, Krabbendam L, Lieb R, Wittchen HU, van Os J. Evidence that the outcome of developmental expression of psychosis is worse for adolescents growing up in an urban environment. *Psychological Medicine*. 2006;36(3):407-415. doi:10.1017/S0033291705006902

502. Spencer MD, Moorhead TWJ, McIntosh AM, et al. Grey matter correlates of early psychotic symptoms in adolescents at enhanced risk of psychosis: A voxel-based study. *Neuroimage*. 2007;35(3). doi:10.1016/j.neuroimage.2007.01.008

503. Spencer TJ, Thompson B, Oliver D, et al. Lower speech connectedness linked to incidence of psychosis in people at clinical high risk. *Schizophrenia Research*. 2021;228:493-501. doi:10.1016/j.schres.2020.09.002

504. Spitz A, Studerus E, Koranyi S, et al. Correlations between self-rating and observer-rating of psychopathology in at-risk mental state and first-episode psychosis patients: influence of disease stage and gender. *Early Intervention in Psychiatry*. 2017;11(6):461-470. doi:10.1111/eip.12270

505. Sprong M, Becker HE, Schothorst PF, et al. Pathways to psychosis: A comparison of the pervasive developmental disorder subtype Multiple Complex Developmental Disorder and the “At Risk Mental State.” *Schizophrenia Research*. 2008;99(1-3):38-47. doi:10.1016/j.schres.2007.10.031

506. Srihari Vinod H, Tek Cenk, Pollard Jessica, et al. Reducing the duration of untreated psychosis and its impact in the U.S.: the STEP-ED study. *BMC Psychiatry*. Published online 2014:14-335.

507. Stanford AD, Messinger J, Malaspina D, Corcoran CM. Theory of Mind in patients at clinical high risk for psychosis. *Schizophrenia Research*. 2011;131(1-3):11-17. doi:10.1016/j.schres.2011.06.005

508. Stoddard J, Niendam T, Hendren R, Carter C, Simon TJ. Attenuated positive symptoms of psychosis in adolescents with chromosome 22q11.2 deletion syndrome. *Schizophrenia Research*. 2010;118(1-3):118-121. doi:10.1016/j.schres.2009.12.011

509. Stojanovic A, Martorell L, Montalvo I, et al. Increased serum interleukin-6 levels in early stages of psychosis: Associations with at-risk mental states and the severity of psychotic symptoms. *Psychoneuroendocrinology*. 2014;41:23-32. doi:10.1016/j.psyneuen.2013.12.005

510. Stone JM, Day F, Tsagaraki H, et al. Glutamate Dysfunction in People with Prodromal Symptoms of Psychosis: Relationship to Gray Matter Volume. *Biological Psychiatry*. 2009;66(6):533-539. doi:10.1016/j.biopsych.2009.05.006

511. Stone JM, Howes OD, Egerton A, et al. Altered Relationship Between Hippocampal Glutamate Levels and Striatal Dopamine Function in Subjects at Ultra High Risk of Psychosis. *Biological Psychiatry*. 2010;68(7):599-602. doi:10.1016/j.biopsych.2010.05.034

512. Stone JM, Bhattacharyya S, Barker GJ, McGuire PK. Substance use and regional gray matter volume in individuals at high risk of psychosis. *European Neuropsychopharmacology*. 2012;22(2):114-122. doi:10.1016/j.euroneuro.2011.06.004

513. Stone JM, Pepper F, Fam J, et al. Glutamate, N-acetyl aspartate and psychotic symptoms in chronic ketamine users. *Psychopharmacology (Berl)*. 2014;231(10):2107-2116. doi:10.1007/s00213-013-3354-8

514. Stowkowy J, Addington J. Maladaptive schemas as a mediator between social defeat and positive symptoms in young people at clinical high risk for psychosis. *Early Intervention in Psychiatry*. 2012;6(1):87-90. doi:10.1111/j.1751-7893.2011.00297.x

515. Stowkowy J, Colijn MA, Addington J. Pathways to care for those at clinical high risk of developing psychosis. *Early Intervention in Psychiatry*. 2013;7(1):80-83. doi:10.1111/j.1751-7893.2012.00368.x

516. Stowkowy J, Addington J. Predictors of a clinical high risk status among individuals with a family history of psychosis. *Schizophrenia Research*. 2013;147(2-3):281-286. doi:10.1016/j.schres.2013.03.030

517. Stowkowy J, Perkins DO, Woods SW, Nyman K, Addington J. Personal Beliefs about Experiences in those at Clinical High Risk for Psychosis. *Behavioural and Cognitive Psychotherapy*. 2015;43(6):669-675. doi:10.1017/S1352465814000307

518. Stowkowy J, Liu L, Cadenhead KS, et al. Early traumatic experiences, perceived discrimination and conversion to psychosis in those at clinical high risk for psychosis. *Social Psychiatry and Psychiatric Epidemiology*. 2016;51(4):497-503. doi:10.1007/s00127-016-1182-y

519. Stowkowy J, Liu L, Cadenhead KS, et al. Core Schemas in Youth at Clinical High Risk for Psychosis. *Behavioural and Cognitive Psychotherapy*. 2016;44(2):203-213. doi:10.1017/S1352465815000144

520. Stowkowy J, Goldstein BI, MacQueen G, et al. Trauma in Youth At-Risk for Serious Mental Illness. *Journal of Nervous & Mental Disease*. 2020;208(1):70-76. doi:10.1097/NMD.0000000000001069

521. Straub KT, Hua JPY, Karcher NR, Kerns JG. Psychosis risk is associated with decreased white matter integrity in limbic network corticostriatal tracts. *Psychiatry Research: Neuroimaging*. 2020;301:111089. doi:10.1016/j.pscychresns.2020.111089

522. Strauss GP, Chapman HC. Preliminary psychometric properties of the brief Negative Symptom Scale in youth at Clinical High-Risk for psychosis. *Schizophrenia Research*. 2018;193:435-437. doi:10.1016/j.schres.2017.07.051

523. Strauss GP, Bartolomeo LA, Luther L. Reduced willingness to expend effort for rewards is associated with risk for conversion and negative symptom severity in youth at clinical high-risk for psychosis. *Psychological Medicine*. Published online June 14, 2021. doi:10.1017/S003329172100204X

524. Strauss GP, Macdonald KI, Ruiz I, Raugh IM, Bartolomeo LA, James SH. The impact of the COVID-19 pandemic on negative symptoms in individuals at clinical high-risk for psychosis and outpatients with chronic schizophrenia. *European Archives of Psychiatry and Clinical Neuroscience*. Published online 2021. doi:10.1007/s00406-021-01260-0

525. Strelchuk D, Wiles N, Turner KM, Derrick C, Zammit S. Feasibility study of eye movement desensitisation and reprocessing (EMDR) in people with an at-risk mental state (ARMS) for psychosis: study protocol. *BMJ Open*. 2020;10(10):e038620. doi:10.1136/bmjopen-2020-038620

526. Studerus E, Corbisiero S, Mazzariello N, et al. Can neuropsychological testing facilitate differential diagnosis between at-risk mental state (ARMS) for psychosis and adult attention-deficit/hyperactivity disorder (ADHD)? *European Psychiatry*. 2018;52:38-44. doi:10.1016/j.eurpsy.2018.02.006

527. Studerus E, Ittig S, Beck K, et al. Relation between self-perceived stress, psychopathological symptoms and the stress hormone prolactin in emerging psychosis. *Journal of Psychiatric Research*. 2021;136:428-434. doi:10.1016/j.jpsychires.2020.06.014

528. Sugranyes G, Thompson JL, Corcoran CM. HPA-axis function, symptoms, and medication exposure in youths at clinical high risk for psychosis. *Journal of Psychiatric Research*. 2012;46(11):1389-1393. doi:10.1016/j.jpsychires.2012.07.011

529. Sullivan SA, Kounali D, Cannon M, et al. A Population-Based Cohort Study Examining the Incidence and Impact of Psychotic Experiences From Childhood to Adulthood, and Prediction of Psychotic Disorder. *American Journal of Psychiatry*. 2020;177(4):308-317. doi:10.1176/appi.ajp.2019.19060654

530. Sumiyoshi T, Kurachi M, Kurokawa K, et al. Plasma homovanillic acid in the prodromal phase of schizophrenia. *Biological Psychiatry*. 2000;47(5):428-433. doi:10.1016/S0006-3223(99)00186-9

531. Sumiyoshi T, Miyanishi T, Seo T, Higuchi Y. Electrophysiological and Neuropsychological Predictors of Conversion to Schizophrenia in At-Risk Subjects. *Frontiers in Behavioral Neuroscience*. 2013;7. doi:10.3389/fnbeh.2013.00148

532. Sun D, Phillips L, Velakoulis D, et al. Progressive brain structural changes mapped as psychosis develops in “at risk” individuals. *Schizophrenia Research*. 2009;108(1-3):85-92. doi:10.1016/j.schres.2008.11.026

533. Suridjan I, Rusjan P, Addington J, Wilson A, Houle S, Mizrahi R. Dopamine D2 and D3 binding in people at clinical high risk for schizophrenia, antipsychotic-naive patients and healthy controls while performing a cognitive task. *Journal of Psychiatry & Neuroscience*. 2013;38(2):98-106. doi:10.1503/jpn.110181

534. Svirskis T, Korkeila J, Heinimaa M, et al. Axis-I disorders and vulnerability to psychosis. *Schizophrenia Research*. 2005;75(2-3):439-446. doi:10.1016/j.schres.2004.11.002

535. Svirskis T, Korkeila J, Heinimaa M, et al. Quality of life and functioning ability in subjects vulnerable to psychosis. *Comprehensive Psychiatry*. 2007;48(2):155-160. doi:10.1016/j.comppsych.2006.10.008

536. Szily E, K&eacute;ri S. Anomalous Subjective Experience and Psychosis Risk in Young Depressed Patients. *Psychopathology*. 2009;42(4):229-235. doi:10.1159/000218520

537. Tada M, Nagai T, Kirihara K, et al. Differential Alterations of Auditory Gamma Oscillatory Responses Between Pre-Onset High-Risk Individuals and First-Episode Schizophrenia. *Cerebral Cortex*. 2016;26(3):1027-1035. doi:10.1093/cercor/bhu278

538. Tagore A, Schifani C, Rao N, et al. Prefrontal cortical dopamine release in clinical high risk for psychosis during a cognitive task: a [11C]FLB457 positron emission tomography study. *European Neuropsychopharmacology*. 2019;29(9):1023-1032. doi:10.1016/j.euroneuro.2019.06.004

539. Takahashi T, Yücel M, Yung AR, et al. Adhesio interthalamica in individuals at high-risk for developing psychosis and patients with psychotic disorders. *Progress in Neuro-Psychopharmacology and Biological Psychiatry*. 2008;32(7):1708-1714. doi:10.1016/j.pnpbp.2008.07.007

540. Takahashi T, Yung AR, Yücel M, et al. Prevalence of large cavum septi pellucidi in ultra high-risk individuals and patients with psychotic disorders. *Schizophrenia Research*. 2008;105(1-3):236-244. doi:10.1016/j.schres.2008.06.021

541. Takahashi T, Wood SJ, Yung AR, et al. *Progressive Gray Matter Reduction of the Superior Temporal Gyrus During Transition to Psychosis*. Vol 66.; 2009.

542. Takahashi T, Wood SJ, Yung AR, et al. Superior temporal gyrus volume in antipsychotic-naive people at risk of psychosis. *British Journal of Psychiatry*. 2010;196(3):206-211. doi:10.1192/bjp.bp.109.069732

543. Takahashi T, Nakamura K, Nishiyama S, et al. Increased pituitary volume in subjects at risk for psychosis and patients with first-episode schizophrenia. *Psychiatry and Clinical Neurosciences*. 2013;67(7):540-548. doi:10.1111/pcn.12093

544. Takahashi T, Wood SJ, Yung AR, et al. Altered depth of the olfactory sulcus in ultra high-risk individuals and patients with psychotic disorders. *Schizophrenia Research*. 2014;153(1-3):18-24. doi:10.1016/j.schres.2014.01.041

545. Takahashi T, Nakamura M, Sasabayashi D, et al. Olfactory deficits in individuals at risk for psychosis and patients with schizophrenia: relationship with socio-cognitive functions and symptom severity. *European Archives of Psychiatry and Clinical Neuroscience*. 2018;268(7):689-698. doi:10.1007/s00406-017-0845-3

546. Takahashi T, Nakamura M, Sasabayashi D, et al. Reduced pineal gland volume across the stages of schizophrenia. *Schizophrenia Research*. 2019;206:163-170. doi:10.1016/j.schres.2018.11.032

547. Takano Y, Aoki Y, Yahata N, et al. Neural basis for inferring false beliefs and social emotions in others among individuals with schizophrenia and those at ultra-high risk for psychosis. *Psychiatry Research: Neuroimaging*. 2017;259:34-41. doi:10.1016/j.pscychresns.2016.11.003

548. Takayanagi Y, Kulason S, Sasabayashi D, et al. Reduced Thickness of the Anterior Cingulate Cortex in Individuals With an At-Risk Mental State Who Later Develop Psychosis. *Schizophrenia Bulletin*. 2017;43(4):907-913. doi:10.1093/schbul/sbw167

549. Tandon N, Montrose D, Shah J, Rajarethinam RP, Diwadkar VA, Keshavan MS. Early prodromal symptoms can predict future psychosis in familial high-risk youth. *Journal of Psychiatric Research*. 2012;46(1):105-110. doi:10.1016/j.jpsychires.2011.09.019

550. Tang Y, Zhang T, Edelman B, et al. Prolonged cortical silent period among drug-naive subjects at ultra-high risk of psychosis. *Schizophrenia Research*. 2014;160(1-3). doi:10.1016/j.schres.2014.10.004

551. Tang SX, Yi JJ, Moore TM, et al. Subthreshold Psychotic Symptoms in 22q11.2 Deletion Syndrome. *Journal of the American Academy of Child & Adolescent Psychiatry*. 2014;53(9):991-1000.e2. doi:10.1016/j.jaac.2014.05.009

552. Tang SX, Yi JJ, Calkins ME, et al. Psychiatric disorders in 22q11.2 deletion syndrome are prevalent but undertreated. *Psychological Medicine*. 2014;44(6):1267-1277. doi:10.1017/S0033291713001669

553. Tang SX, Moore TM, Calkins ME, et al. Emergent, remitted and persistent psychosis-spectrum symptoms in 22q11.2 deletion syndrome. *Translational Psychiatry*. 2017;7(7):e1180-e1180. doi:10.1038/tp.2017.157

554. Tang Y, Pasternak O, Kubicki M, et al. Altered Cellular White Matter But Not Extracellular Free Water on Diffusion MRI in Individuals at Clinical High Risk for Psychosis. *American Journal of Psychiatry*. 2019;176(10):820-828. doi:10.1176/appi.ajp.2019.18091044

555. Tapley Gasper AE. *The Effects of High -Intensity Rehabilitation and Cognitive Therapy on Insight in Schizophrenia.* . AAI9989185. ETD collection for University of Nebraska - Lincoln; 2000.

556. Tarbox SI, Addington J, Cadenhead KS, et al. Premorbid functional development and conversion to psychosis in clinical high-risk youths. *Development and Psychopathology*. 2013;25(4pt1):1171-1186. doi:10.1017/S0954579413000448

557. Tarbox SI, Addington J, Cadenhead KS, et al. Functional development in clinical high risk youth: Prediction of schizophrenia versus other psychotic disorders. *Psychiatry Research*. 2014;215(1):52-60. doi:10.1016/j.psychres.2013.10.006

558. Tarbox-Berry SI, Perkins DO, Woods SW, Addington J. Premorbid social adjustment and association with attenuated psychotic symptoms in clinical high-risk and help-seeking youth. *Psychological Medicine*. 2018;48(6):983-997. doi:10.1017/S0033291717002343

559. Tay SA, Yuen S, Lim LK, et al. Early intervention in the real world. . *Early Intervention in Psychiatry*. 2015;9(6):516-522.

560. Taylor HE, Parker S, Mansell W, Morrison AP. Effects of Appraisals of Anomalous Experience on Distress in People at Risk of Psychosis. *Behavioural and Cognitive Psychotherapy*. 2013;41(1):24-33. doi:10.1017/S1352465812000227

561. Taylor HE, Stewart SLK, Dunn G, Parker S, Fowler D, Morrison AP. Core Schemas across the Continuum of Psychosis: A Comparison of Clinical and Non-Clinical Groups. *Behavioural and Cognitive Psychotherapy*. 2014;42(6):718-730. doi:10.1017/S1352465813000593

562. Taylor HE, Stewart SLK, Dunn G, et al. Psychopathology and affect dysregulation across the continuum of psychosis: a multiple comparison group study. *Early Intervention in Psychiatry*. 2014;8(3):221-228. doi:10.1111/eip.12064

563. Tessner KD, Mittal V, Walker EF. Longitudinal Study of Stressful Life Events and Daily Stressors Among Adolescents at High Risk for Psychotic Disorders. *Schizophrenia Bulletin*. 2011;37(2):432-441. doi:10.1093/schbul/sbp087

564. Theodoridou A, Hengartner MP, Heekeren K, et al. Influence of demographic characteristics on attenuated positive psychotic symptoms in a young, help-seeking, at-risk population. *Early Intervention in Psychiatry*. 2019;13(1):53-56. doi:10.1111/eip.12444

565. Therman S, Suvisaari JM, Kalska H, Huttunen MO, Manninen M, Cannon TD. Lack of Association Between Neuropsychological Performance and Level of Psychosis-Proneness in an Adolescent Psychiatric Sample. *Journal of Nervous & Mental Disease*. 2009;197(9):669-674. doi:10.1097/NMD.0b013e3181b3b152

566. Therman S, Heinimaa M, Miettunen J, et al. Symptoms associated with psychosis risk in an adolescent birth cohort: improving questionnaire utility with a multidimensional approach. *Early Intervention in Psychiatry*. 2011;5(4):343-348. doi:10.1111/j.1751-7893.2011.00290.x

567. Therman S, Lindgren M, Manninen M, et al. Predicting psychosis and psychiatric hospital care among adolescent psychiatric patients with the Prodromal Questionnaire. *Schizophrenia Research*. 2014;158(1-3):7-10. doi:10.1016/j.schres.2014.06.031

568. Thermenos HW, Juelich RJ, DiChiara SR, et al. Hyperactivity of caudate, parahippocampal, and prefrontal regions during working memory in never-medicated persons at clinical high-risk for psychosis. *Schizophrenia Research*. 2016;173(1-2):1-12. doi:10.1016/j.schres.2016.02.023

569. Thompson KN, Berger G, Phillips LJ, Komesaroff P, Purcell R, McGorry PD. HPA axis functioning associated with transition to psychosis: Combined DEX/CRH test. *Journal of Psychiatric Research*. 2007;41(5):446-450. doi:10.1016/j.jpsychires.2005.11.010

570. Thompson JL, Kelly M, Kimhy D, et al. Childhood trauma and prodromal symptoms among individuals at clinical high risk for psychosis. *Schizophrenia Research*. 2009;108(1-3):176-181. doi:10.1016/j.schres.2008.12.005

571. Thompson A, Papas A, Bartholomeusz C, et al. Social cognition in clinical “at risk” for psychosis and first episode psychosis populations. *Schizophrenia Research*. 2012;141(2-3):204-209. doi:10.1016/j.schres.2012.08.007

572. Thompson A, Papas A, Bartholomeusz C, Nelson B, Yung A. Externalized attributional bias in the Ultra High Risk (UHR) for psychosis population. *Psychiatry Research*. 2013;206(2-3):200-205. doi:10.1016/j.psychres.2012.10.017

573. Thompson E, Kline E, Reeves G, Pitts SC, Schiffman J. Identifying youth at risk for psychosis using the Behavior Assessment System for Children, Second Edition. *Schizophrenia Research*. 2013;151(1-3):238-244. doi:10.1016/j.schres.2013.09.022

574. Thompson A, Nelson B, Bruxner A, et al. Does specific psychopathology predict development of psychosis in ultra high-risk (UHR) patients? *Australian & New Zealand Journal of Psychiatry*. 2013;47(4):380-390. doi:10.1177/0004867413476753

575. Thompson E, Kline E, Reeves G, Pitts SC, Bussell K, Schiffman J. Using parent and youth reports from the Behavior Assessment System for Children, Second Edition to identify individuals at clinical high-risk for psychosis. *Schizophrenia Research*. 2014;154(1-3):107-112. doi:10.1016/j.schres.2014.02.009

576. Thompson AD, Nelson B, Yuen HP, et al. Sexual Trauma Increases the Risk of Developing Psychosis in an Ultra High-Risk “Prodromal” Population. *Schizophrenia Bulletin*. 2014;40(3):697-706. doi:10.1093/schbul/sbt032

577. Thompson E, Rakhshan P, Pitts SC, et al. Family functioning moderates the impact of psychosis-risk symptoms on social and role functioning. *Schizophrenia Research*. 2019;204:337-342. doi:10.1016/j.schres.2018.08.035

578. Tijms BM, Sprooten E, Job D, et al. Grey matter networks in people at increased familial risk for schizophrenia. *Schizophrenia Research*. 2015;168(1-2):1-8. doi:10.1016/j.schres.2015.08.025

579. Tikka M, Luutonen S, Ilonen T, et al. Childhood trauma and premorbid adjustment among individuals at clinical high risk for psychosis and normal control subjects. *Early Intervention in Psychiatry*. 2013;7(1):51-57. doi:10.1111/j.1751-7893.2012.00391.x

580. Tikka DL, Singh AR, Tikka SK. Higher number of minor physical anomalies correlates with frequency of prodromal symptoms in youth at elevated clinical risk for psychosis. *Asian Journal of Psychiatry*. 2020;47:101869. doi:10.1016/j.ajp.2019.101869

581. Tognin S, Riecher-Rössler A, Meisenzahl EM, et al. Reduced parahippocampal cortical thickness in subjects at ultra-high risk for psychosis. *Psychological Medicine*. 2014;44(3):489-498. doi:10.1017/S0033291713000998

582. Tognin S, Pettersson-Yeo W, Valli I, et al. Using Structural Neuroimaging to Make Quantitative Predictions of Symptom Progression in Individuals at Ultra-High Risk for Psychosis. *Frontiers in Psychiatry*. 2014;4. doi:10.3389/fpsyt.2013.00187

583. Tognin S, Catalan A, Modinos G, et al. Emotion recognition and adverse childhood experiences in individuals at clinical high risk of psychosis. *Schizophrenia Bulletin*. 2020;46(4):823-833. doi:10.1093/schbul/sbz128

584. Tomyshev AS, Lebedeva IS, Akhadov TA, Omelchenko MA, Rumyantsev AO, Kaleda VG. Alterations in white matter microstructure and cortical thickness in individuals at ultra-high risk of psychosis: A multimodal tractography and surface-based morphometry study. *Psychiatry Research: Neuroimaging*. 2019;289:26-36. doi:10.1016/j.pscychresns.2019.05.002

585. Torjesen I. High potency cannabis is associated with tripled risk of psychosis, study indicates. *BMJ*. 2015;350(feb18 12):h939-h939. doi:10.1136/bmj.h939

586. Trotman HD, Holtzman CW, Walker EF, et al. Stress exposure and sensitivity in the clinical high-risk syndrome: Initial findings from the North American Prodrome Longitudinal Study (NAPLS). *Schizophrenia Research*. 2014;160(1-3). doi:10.1016/j.schres.2014.09.017

587. Tseng HH, Roiser JP, Modinos G, et al. Corticolimbic dysfunction during facial and prosodic emotional recognition in first-episode psychosis patients and individuals at ultra-high risk. *NeuroImage: Clinical*. 2016;12:645-654. doi:10.1016/j.nicl.2016.09.006

588. Tso IF, Taylor SF, Grove TB, et al. Factor analysis of the Scale of Prodromal Symptoms: data from the Early Detection and Intervention for the Prevention of Psychosis Program. *Early Intervention in Psychiatry*. 2017;11(1):14-22. doi:10.1111/eip.12209

589. Tsuji T, Phalen P, Rakhshan Rouhakhtar P, et al. Using the K-SADS psychosis screen to identify people with early psychosis or psychosis risk syndromes. *Clinical Child Psychology and Psychiatry*. 2019;24(4):809-820. doi:10.1177/1359104519846582

590. Tsujino N, Nemoto T, Morita K, Katagiri N, Ito S, Mizuno M. Long-term Efficacy and Tolerability of Perospirone for Young Help-seeking People at Clinical High Risk: a Preliminary Open Trial. *Clinical Psychopharmacology and Neuroscience*. 2013;11(3):132-136. doi:10.9758/cpn.2013.11.3.132

591. Turetsky BI, Moberg PJ, Quarmley M, et al. Structural anomalies of the peripheral olfactory system in psychosis high-risk subjects. *Schizophrenia Research*. 2018;195:197-205. doi:10.1016/j.schres.2017.09.015

592. Uchida T, Matsumoto K, Ito F, et al. Relationship between cognitive insight and attenuated delusional symptoms in individuals with at-risk mental state. *Psychiatry Research*. 2014;217(1-2):20-24. doi:10.1016/j.psychres.2014.01.003

593. Üçok A, Direk N, Koyuncu A, et al. Cognitive deficits in clinical and familial high risk groups for psychosis are common as in first episode schizophrenia. *Schizophrenia Research*. 2013;151(1-3):265-269. doi:10.1016/j.schres.2013.10.030

594. Üçok A, Kaya H, Uğurpala C, et al. History of childhood physical trauma is related to cognitive decline in individuals with ultra-high risk for psychosis. *Schizophrenia Research*. 2015;169(1-3):199-203. doi:10.1016/j.schres.2015.08.038

595. Uhl I, Mavrogiorgou P, Norra C, et al. 1H-MR spectroscopy in ultra-high risk and first episode stages of schizophrenia. *Journal of Psychiatric Research*. 2011;45(9):1135-1139. doi:10.1016/j.jpsychires.2011.02.004

596. Uhlhaas PJ, Gajwani R, Gross J, Gumley AI, Lawrie SM, Schwannauer M. The Youth Mental Health Risk and Resilience Study (YouR-Study). *BMC Psychiatry*. 2017;17(1):43. doi:10.1186/s12888-017-1206-5

597. Urben S, Pihet S, Jaugey L, Halfon O, Holzer L. Computer-assisted cognitive remediation in adolescents with psychosis or at risk for psychosis: a 6-month follow-up. *Acta Neuropsychiatrica*. 2012;24(6):328-335. doi:10.1111/j.1601-5215.2012.00651.x

598. Uttinger M, Studerus E, Ittig S, Heitz U, Schultze-Lutter F, Riecher-Rössler A. The Frankfurt Complaint Questionnaire for self-assessment of basic symptoms in the early detection of psychosis-Factor structure, reliability, and predictive validity. *International Journal of Methods in Psychiatric Research*. 2018;27(2):e1600. doi:10.1002/mpr.1600

599. Uttinger M, Koranyi S, Papmeyer M, et al. Early detection of psychosis: helpful or stigmatizing experience? A qualitative study. *Early Intervention in Psychiatry*. 2018;12(1):66-73. doi:10.1111/eip.12273

600. Uzdawinis D, Edel MA, Özgürdal S, et al. Operationalisierte Psychodynamische Diagnostik (OPD) bei Patienten im schizophrenen Prodromalstadium - Eine explorative Studie. *Zeitschrift für Psychosomatische Medizin und Psychotherapie*. 2010;56(2):150-162. doi:10.13109/zptm.2010.56.2.150

601. Vadhan NP, Corcoran CM, Bedi G, Keilp JG, Haney M. Acute effects of smoked marijuana in marijuana smokers at clinical high-risk for psychosis: A preliminary study. *Psychiatry Research*. 2017;257:372-374. doi:10.1016/j.psychres.2017.07.070

602. Væver MS, Licht DM, Møller L, et al. Thinking within the spectrum: schizophrenic thought disorder in six Danish pedigrees. *Schizophrenia Research*. 2005;72(2-3):137-149. doi:10.1016/j.schres.2004.04.001

603. Valdés-Florido MJ, López-Díaz Á, Palermo-Zeballos FJ, et al. Clinical characterization of brief psychotic disorders triggered by the COVID-19 pandemic: a multicenter observational study. *European Archives of Psychiatry and Clinical Neuroscience*. 2022;272(1):5-15. doi:10.1007/s00406-021-01256-w

604. Valli I, Stone J, Mechelli A, et al. Altered Medial Temporal Activation Related to Local Glutamate Levels in Subjects with Prodromal Signs of Psychosis. *Biological Psychiatry*. 2011;69(1):97-99. doi:10.1016/j.biopsych.2010.08.033

605. Valli I, Crossley NA, Day F, et al. HPA-axis function and grey matter volume reductions: imaging the diathesis-stress model in individuals at ultra-high risk of psychosis. *Translational Psychiatry*. 2016;6(5):e797-e797. doi:10.1038/tp.2016.68

606. Valli I, Marquand AF, Mechelli A, et al. Identifying Individuals at High Risk of Psychosis: Predictive Utility of Support Vector Machine using Structural and Functional MRI Data. *Frontiers in Psychiatry*. 2016;7. doi:10.3389/fpsyt.2016.00052

607. Valmaggia Lucia R., Freeman Daniel, Green Catherine, et al. Virtual reality and paranoid ideations in peoplewith an ‘at-riskmental state’ for psychosis. *British Journal of Psychiatry*. 2007;191(suppl. 51):s63-s68.

608. Valmaggia LR, Stahl D, Yung AR, et al. Negative psychotic symptoms and impaired role functioning predict transition outcomes in the at-risk mental state: a latent class cluster analysis study. *Psychological Medicine*. 2013;43(11):2311-2325. doi:10.1017/S0033291713000251

609. Valmaggia LR, Day FL, Jones C, et al. Cannabis use and transition to psychosis in people at ultra-high risk. *Psychological Medicine*. 2014;44(12):2503-2512. doi:10.1017/S0033291714000117

610. Valmaggia LR, Day F, Garety P, et al. Social defeat predicts paranoid appraisals in people at high risk for psychosis. *Schizophrenia Research*. 2015;168(1-2):16-22. doi:10.1016/j.schres.2015.07.050

611. Valmaggia LR, Day FL, Kroll J, et al. Bullying victimisation and paranoid ideation in people at ultra high risk for psychosis. *Schizophrenia Research*. 2015;168(1-2):68-73. doi:10.1016/j.schres.2015.08.029

612. van der Stelt O, Lieberman JA, Belger A. Auditory P300 in high-risk, recent-onset and chronic schizophrenia. *Schizophrenia Research*. 2005;77(2-3):309-320. doi:10.1016/j.schres.2005.04.024

613. van der Velde J, Swart M, van Rijn S, et al. Cognitive Alexithymia Is Associated with the Degree of Risk for Psychosis. *PLOS ONE*. 2015;10(6):e0124803. doi:10.1371/journal.pone.0124803

614. van Kampen D. The Schizotypic Syndrome Questionnaire (SSQ): Psychometrics, validation and norms. *Schizophrenia Research*. 2006;84(2-3):305-322. doi:10.1016/j.schres.2006.02.011

615. van Kampen D, Maurer K, an der Heiden W, Häfner H. Prodromal unfolding: the validation of the Schizotypic Syndrome Questionnaire model in a sample of first-episode schizophrenic patients. *Early Intervention in Psychiatry*. 2009;3(2):137-150. doi:10.1111/j.1751-7893.2009.00120.x

616. van Os J, Marcelis M, Sham P, Jones P, Gilvarry K, Murray R. Psychopathological syndromes and familial morbid risk of psychosis. *British Journal of Psychiatry*. 1997;170(3):241-246. doi:10.1192/bjp.170.3.241

617. van Rijn S, Schothorst P, Wout M van ’t, et al. Affective dysfunctions in adolescents at risk for psychosis: Emotion awareness and social functioning. *Psychiatry Research*. 2011;187(1-2):100-105. doi:10.1016/j.psychres.2010.10.007

618. van Rijn S, Schothorst P, Wout M van ’t, et al. Affective dysfunctions in adolescents at risk for psychosis: Emotion awareness and social functioning. *Psychiatry Research*. 2011;187(1-2):100-105. doi:10.1016/j.psychres.2010.10.007

619. van Tricht MJ, Nieman DH, Bour LJ, et al. Increased saccadic rate during smooth pursuit eye movements in patients at Ultra High Risk for developing a psychosis. *Brain and Cognition*. 2010;73(3):215-221. doi:10.1016/j.bandc.2010.05.005

620. van Tricht MJ, Nieman DH, Koelman JHTM, et al. Reduced Parietal P300 Amplitude is Associated with an Increased Risk for a First Psychotic Episode. *Biological Psychiatry*. 2010;68(7):642-648. doi:10.1016/j.biopsych.2010.04.022

621. van Tricht MJ, Nieman DH, Koelman JHTM, et al. Auditory ERP components before and after transition to a first psychotic episode. *Biological Psychology*. 2011;87(3):350-357. doi:10.1016/j.biopsycho.2011.04.005

622. van Tricht MJ, Harmsen EC, Koelman JHTM, et al. Effects of cannabis use on event related potentials in subjects at ultra high risk for psychosis and healthy controls. *International Journal of Psychophysiology*. 2013;88(2):149-156. doi:10.1016/j.ijpsycho.2013.03.012

623. van Tricht MJ, Ruhrmann S, Arns M, et al. Can quantitative EEG measures predict clinical outcome in subjects at Clinical High Risk for psychosis? A prospective multicenter study. *Schizophrenia Research*. 2014;153(1-3):42-47. doi:10.1016/j.schres.2014.01.019

624. van Tricht MJ, Bour LJ, Koelman JHTM, et al. Qualitative and quantitative aspects of information processing in first psychosis: Latent class analyses in patients, at-risk subjects, and controls. *Psychophysiology*. 2015;52(4):585-593. doi:10.1111/psyp.12379

625. van Tricht MJ, Nieman DH, Koelman JTM, et al. Sensory gating in subjects at ultra high risk for developing a psychosis before and after a first psychotic episode. *The World Journal of Biological Psychiatry*. 2015;16(1):12-21. doi:10.3109/15622975.2012.680911

626. Vargas T, Snyder H, Banich M, et al. Altered selection during language processing in individuals at high risk for psychosis. *Schizophrenia Research*. 2018;202:303-309. doi:10.1016/j.schres.2018.06.036

627. Vargas T, Damme KSF, Hooker CI, Gupta T, Cowan HR, Mittal VA. Differentiating implicit and explicit theory of mind and associated neural networks in youth at Clinical High Risk (CHR) for psychosis. *Schizophrenia Research*. 2019;208:173-181. doi:10.1016/j.schres.2019.03.013

628. Vargas T, Ahmed AO, Strauss GP, et al. The latent structure of depressive symptoms across clinical high risk and chronic phases of psychotic illness. *Translational Psychiatry*. 2019;9(1):229. doi:10.1038/s41398-019-0563-x

629. Vasilyeva EF, Kushner SG, Factor MI, et al. The cellular factors of innate immunity in nonpsychotic patients at high risk for schizophrenia. *Zhurnal nevrologii i psikhiatrii im SS Korsakova*. 2016;116(10):60. doi:10.17116/jnevro201611610160-65

630. Veijola J, Mäki P, Jääskeläinen E, et al. Young people at risk for psychosis: case finding and sample characteristics of the Oulu Brain and Mind Study. *Early Intervention in Psychiatry*. 2013;7(2):146-154. doi:10.1111/j.1751-7893.2012.00360.x

631. Veling W, Pot-Kolder R, Counotte J, van Os J, van der Gaag M. Environmental Social Stress, Paranoia and Psychosis Liability: A Virtual Reality Study. *Schizophrenia Bulletin*. 2016;42(6):1363-1371. doi:10.1093/schbul/sbw031

632. Veling W, Counotte J, Pot-Kolder R, van Os J, van der Gaag M. Childhood trauma, psychosis liability and social stress reactivity: a virtual reality study. *Psychological Medicine*. 2016;46(16):3339-3348. doi:10.1017/S0033291716002208

633. Velthorst E, Nieman DH, Linszen D, et al. Disability in people clinically at high risk of psychosis. *British Journal of Psychiatry*. 2010;197(4):278-284. doi:10.1192/bjp.bp.109.075036

634. Velthorst E, Nieman DH, Klaassen RMC, et al. Three-year course of clinical symptomatology in young people at ultra high risk for transition to psychosis. *Acta Psychiatrica Scandinavica*. 2011;123(1):36-42. doi:10.1111/j.1600-0447.2010.01593.x

635. Velthorst E, Derks EM, Schothorst P, et al. Quantitative and qualitative symptomatic differences in individuals at Ultra-High Risk for psychosis and healthy controls. *Psychiatry Research*. 2013;210(2):432-437. doi:10.1016/j.psychres.2013.07.018

636. Velthorst E, Nelson B, Wiltink S, et al. Transition to first episode psychosis in ultra high risk populations: Does baseline functioning hold the key? *Schizophrenia Research*. 2013;143(1):132-137. doi:10.1016/j.schres.2012.10.025

637. Velthorst E, Zinberg J, Addington J, et al. Potentially important periods of change in the development of social and role functioning in youth at clinical high risk for psychosis. *Development and Psychopathology*. 2018;30(1):39-47. doi:10.1017/S0954579417000451

638. Velthorst E, Meyer EC, Giuliano AJ, et al. Neurocognitive profiles in the prodrome to psychosis in NAPLS-1. *Schizophrenia Research*. 2019;204:311-319. doi:10.1016/j.schres.2018.07.038

639. Ventura J, Jouini L, Aissa A, et al. Establishing a clinical high-risk program in Tunisia, North Africa: A pilot study in early detection and identification. *Early Intervention in Psychiatry*. Published online 2021. doi:10.1111/eip.13119

640. Verma S, Poon LY, Lee H, Rao S, Chong SA. Evolution of early psychosis intervention services in Singapore. *East Asian Arch Psychiatry*. 2012;22(3):114-117.

641. Versmissen D, Myin-Germeys I, Janssen I, et al. Impairment of self-monitoring: part of the endophenotypic risk for psychosis. *British Journal of Psychiatry*. 2007;191(S51). doi:10.1192/bjp.191.51.s58

642. Vingerhoets C, Bloemen OJN, Boot E, et al. Dopamine in high-risk populations: A comparison of subjects with 22q11.2 deletion syndrome and subjects at ultra high-risk for psychosis. *Psychiatry Research: Neuroimaging*. 2018;272:65-70. doi:10.1016/j.pscychresns.2017.11.014

643. Visser KF. *Emotion Regulation in Individuals at Risk for Psychosis: An Ecological Momentary Assessment and Ambulatory Psychophysiological Study*. 2020.

644. Vollmer-Larsen A, Handest P, Parnas J. Reliability of measuring anomalous experience: The Bonn Scale for the Assessment of Basic Symptoms. *Psychopathology*. 2007;40(5):345-348. doi:10.1159/000106311

645. Waford RN, MacDonald A, Goines K, et al. Demographic correlates of attenuated positive psychotic symptoms. *Schizophrenia Research*. 2015;166(1-3):31-36. doi:10.1016/j.schres.2015.04.035

646. Waite F, Bradley J, Chadwick E, Reeve S, Bird JC, Freeman D. The Experience of Sleep Problems and Their Treatment in Young People at Ultra-High Risk of Psychosis: A Thematic Analysis. *Frontiers in Psychiatry*. 2018;9. doi:10.3389/fpsyt.2018.00375

647. Waite F, Kabir T, Johns L, et al. Treating sleep problems in young people at ultra-high-risk of psychosis: Study protocol for a single-blind parallel group randomised controlled feasibility trial (SleepWell). *BMJ Open*. 2020;10(11). doi:10.1136/bmjopen-2020-045235

648. Wake R, Araki T, Fukushima M, et al. Urinary biopyrrins and free immunoglobin light chains are biomarker candidates for screening at-risk mental state in adolescents. *Early Intervention in Psychiatry*. Published online 2021. doi:10.1111/eip.13154

649. Walder DJ, Mittal V, Trotman HD, McMillan AL, Walker EF. Neurocognition and conversion to psychosis in adolescents at high-risk. *Schizophrenia Research*. 2008;101(1-3):161-168. doi:10.1016/j.schres.2007.12.477

650. Walder DJ, Holtzman CW, Addington J, et al. Sexual dimorphisms and prediction of conversion in the NAPLS psychosis prodrome. *Schizophrenia Research*. 2013;144(1-3):43-50. doi:10.1016/j.schres.2012.11.039

651. Walker EF, Cornblatt BA, Addington J, et al. The relation of antipsychotic and antidepressant medication with baseline symptoms and symptom progression: A naturalistic study of the North American Prodrome Longitudinal Sample. *Schizophrenia Research*. 2009;115(1):50-57. doi:10.1016/j.schres.2009.07.023

652. Walker EF, Trotman HD, Pearce BD, et al. Cortisol Levels and Risk for Psychosis: Initial Findings from the North American Prodrome Longitudinal Study. *Biological Psychiatry*. 2013;74(6):410-417. doi:10.1016/j.biopsych.2013.02.016

653. Walter A, Studerus E, Smieskova R, et al. Hippocampal volume in subjects at high risk of psychosis: A longitudinal MRI study. *Schizophrenia Research*. 2012;142(1-3):217-222. doi:10.1016/j.schres.2012.10.013

654. Walter A, Studerus E, Smieskova R, et al. Pituitary gland volume in at-risk mental state for psychosis: a longitudinal MRI analysis. *CNS Spectrums*. 2015;20(2):122-129. doi:10.1017/S109285291400011X

655. Walterfang M, Yung A, Wood AG, et al. Corpus callosum shape alterations in individuals prior to the onset of psychosis. *Schizophrenia Research*. 2008;103(1-3):1-10. doi:10.1016/j.schres.2008.04.042

656. Wang L, Shi J, Chen F, et al. Family Perception and 6-Month Symptomatic and Functioning Outcomes in Young Adolescents at Clinical High Risk for Psychosis in a General Population in China. *PLOS ONE*. 2015;10(9):e0138361. doi:10.1371/journal.pone.0138361

657. Wang J, Tang Y, Zhang T, et al. Reduced *γ* -Aminobutyric Acid and Glutamate+Glutamine Levels in Drug-Naïve Patients with First-Episode Schizophrenia but Not in Those at Ultrahigh Risk. *Neural Plasticity*. 2016;2016:1-9. doi:10.1155/2016/3915703

658. Wang S, Wang G, Lv H, Wu R, Zhao J, Guo W. Abnormal regional homogeneity as potential imaging biomarker for psychosis risk syndrome: a resting-state fMRI study and support vector machine analysis. *Scientific Reports*. 2016;6(1):27619. doi:10.1038/srep27619

659. Wang H, Guo W, Liu F, et al. Patients with first-episode, drug-naive schizophrenia and subjects at ultra-high risk of psychosis shared increased cerebellar-default mode network connectivity at rest. *Scientific Reports*. 2016;6(1):26124. doi:10.1038/srep26124

660. Wang C, Ji F, Hong Z, et al. Disrupted salience network functional connectivity and white-matter microstructure in persons at risk for psychosis: findings from the LYRIKS study. *Psychological Medicine*. 2016;46(13):2771-2783. doi:10.1017/S0033291716001410

661. Wang Y, Lin Y, Liu C, et al. Stigmas toward psychosis‐related clinical features among the general public in Taiwan. *Asia-Pacific Psychiatry*. 2020;12(1). doi:10.1111/appy.12370

662. Wang G, Lyu H, Wu R, et al. Resting-state functional hypoconnectivity of amygdala in clinical high risk state and first-episode schizophrenia. *Brain Imaging and Behavior*. 2020;14(5):1840-1849. doi:10.1007/s11682-019-00124-5

663. Ward HB, Lawson MT, Addington J, et al. Tobacco use and psychosis risk in persons at clinical high risk. *Early Intervention in Psychiatry*. 2019;13(5):1173-1181. doi:10.1111/eip.12751

664. Warner R. Limitations of the Bonn Scale for the Assessment of Basic Symptoms as a Screening Measure. *Archives of General Psychiatry*. 2002;59(5). doi:10.1001/archpsyc.59.5.470

665. Warnick EL, Allen DN. Verbal and spatial working memory differences among individuals with familial and non-familial risk for schizophrenia. *Schizophrenia Research*. 2005;77(2-3):361-362. doi:10.1016/j.schres.2005.03.010

666. Washida K, Aoki, Habara, et al. Efficacy of second-generation antipsychotics in patients at ultra-high risk and those with first- episode or multi-episode schizophrenia. *Neuropsychiatric Disease and Treatment*. Published online June 2013:861. doi:10.2147/NDT.S45697

667. Watters AJ, Rupert PE, Wolf DH, et al. Social aversive conditioning in youth at clinical high risk for psychosis and with psychosis: An ERP study. *Schizophrenia Research*. 2018;202:291-296. doi:10.1016/j.schres.2018.06.027

668. Weinberger R, Weisman O, Guri Y, Harel T, Weizman A, Gothelf D. The interaction between neurocognitive functioning, subthreshold psychotic symptoms and pharmacotherapy in 22q11.2 deletion syndrome: A longitudinal comparative study. *European Psychiatry*. 2018;48(1):20-26. doi:10.1016/j.eurpsy.2017.10.010

669. Weintraub MJ, Weisman de Mamani A, Villano WJ, et al. Affective and physiological reactivity to emotional comments in individuals at elevated risk for psychosis. *Schizophrenia Research*. 2019;206:428-435. doi:10.1016/j.schres.2018.10.006

670. Welch KA, McIntosh AM, Job DE, et al. The Impact of Substance Use on Brain Structure in People at High Risk of Developing Schizophrenia. *Schizophrenia Bulletin*. 2011;37(5):1066-1076. doi:10.1093/schbul/sbq013

671. Welsh P, Mediavilla JL, Tiffin PA. Attitudes and knowledge of child and adolescent mental health clinicians in relation to the psychosis risk syndrome. *Early Intervention in Psychiatry*. 2011;5(4):355-359. doi:10.1111/j.1751-7893.2011.00285.x

672. Welsh P, Tiffin PA. Observations of a Small Sample of Adolescents Experiencing an At-Risk Mental State (ARMS) for Psychosis. *Schizophrenia Bulletin*. 2012;38(2):215-218. doi:10.1093/schbul/sbr139

673. Welsh P, Cartwright-Hatton S, Wells A, Snow L, Tiffin PA. Metacognitive beliefs in adolescents with an at-risk mental state for psychosis. *Early Intervention in Psychiatry*. 2014;8(1):82-86. doi:10.1111/eip.12052

674. Welsh P, Tiffin PA. Assessing adolescent preference in the treatment of first-episode psychosis and psychosis risk. *Early Intervention in Psychiatry*. 2014;8(3):281-285. doi:10.1111/eip.12077

675. Welsh P, Tiffin PA. Adolescent family perceptions in the At-Risk Mental State for psychosis. *Early Intervention in Psychiatry*. 2015;9(4):316-323. doi:10.1111/eip.12115

676. Wenneberg C, Glenthøj BY, Glenthøj LB, et al. Baseline measures of cerebral glutamate and GABA levels in individuals at ultrahigh risk for psychosis: Implications for clinical outcome after 12 months. *European Psychiatry*. 2020;63(1). doi:10.1192/j.eurpsy.2020.77

677. Wenneberg C, Nordentoft M, Rostrup E, et al. Cerebral Glutamate and Gamma-Aminobutyric Acid Levels in Individuals at Ultra-high Risk for Psychosis and the Association With Clinical Symptoms and Cognition. *Biological Psychiatry: Cognitive Neuroscience and Neuroimaging*. 2020;5(6):569-579. doi:10.1016/j.bpsc.2019.12.005

678. Werbeloff N. Self-reported Attenuated Psychotic Symptoms as Forerunners of Severe Mental Disorders Later in Life. *Archives of General Psychiatry*. 2012;69(5):467. doi:10.1001/archgenpsychiatry.2011.1580

679. Wessels H, Wagner M, Kuhr K, et al. Predictors of treatment response to psychological interventions in people at clinical high risk of first-episode psychosis. *Early Intervention in Psychiatry*. 2019;13(1):120-127. doi:10.1111/eip.12460

680. Whalley HC, Adams MJ, Hall LS, et al. Dissection of major depressive disorder using polygenic risk scores for schizophrenia in two independent cohorts. *Translational Psychiatry*. 2016;6(11):e938-e938. doi:10.1038/tp.2016.207

681. Whitford TJ, Wood SJ, Yung A, et al. Structural abnormalities in the cuneus associated with Herpes Simplex Virus (type 1) infection in people at ultra high risk of developing psychosis. *Schizophrenia Research*. 2012;135(1-3):175-180. doi:10.1016/j.schres.2011.11.003

682. Whitford TJ, Oestreich LKL, Ford JM, et al. Deficits in Cortical Suppression During Vocalization are Associated With Structural Abnormalities in the Arcuate Fasciculus in Early Illness Schizophrenia and Clinical High Risk for Psychosis. *Schizophrenia Bulletin*. 2018;44(6):1312-1322. doi:10.1093/schbul/sbx144

683. Whiting D. Synthetic Cannabinoid Receptor Agonists: a heterogeneous class of novel psychoactive substance with emerging risk of psychosis. *Evidence Based Mental Health*. 2015;18(4):110-110. doi:10.1136/eb-2015-102188

684. Wigman JTW, van Nierop M, Vollebergh WAM, et al. Evidence That Psychotic Symptoms Are Prevalent in Disorders of Anxiety and Depression, Impacting on Illness Onset, Risk, and Severity--Implications for Diagnosis and Ultra-High Risk Research. *Schizophrenia Bulletin*. 2012;38(2):247-257. doi:10.1093/schbul/sbr196

685. Wijnen BFM, Thielen FW, Konings S, et al. Designing and Testing of a Health-Economic Markov Model for Prevention and Treatment of Early Psychosis. *Expert Review of Pharmacoeconomics and Outcomes Research*. 2020;20(3):269-279. doi:10.1080/14737167.2019.1632194

686. Wilcox J, Briones D, Quadri S, Tsuang M. Prognostic implications of paranoia and thought disorder in new onset psychosis. *Comprehensive Psychiatry*. 2014;55(4):813-817. doi:10.1016/j.comppsych.2013.12.010

687. Willhite RK, Niendam TA, Bearden CE, Zinberg J, O’Brien MP, Cannon TD. Gender differences in symptoms, functioning and social support in patients at ultra-high risk for developing a psychotic disorder. *Schizophrenia Research*. 2008;104(1-3):237-245. doi:10.1016/j.schres.2008.05.019

688. Wilquin H, Delevoye-Turrell Y. Motor Agency: A New and Highly Sensitive Measure to Reveal Agency Disturbances in Early Psychosis. *PLoS ONE*. 2012;7(2):e30449. doi:10.1371/journal.pone.0030449

689. Wilson R, Bossong MG, Appiah-Kusi E, et al. Cannabidiol attenuates insular dysfunction during motivational salience processing in subjects at clinical high risk for psychosis. *Translational Psychiatry*. 2019;9(1):203. doi:10.1038/s41398-019-0534-2

690. Wiltink S, Velthorst E, Nelson B, McGorry PM, Yung AR. Declining transition rates to psychosis: the contribution of potential changes in referral pathways to an ultra-high-risk service. *Early Intervention in Psychiatry*. 2015;9(3):200-206. doi:10.1111/eip.12105

691. Winton-Brown T, Schmidt A, Roiser JP, et al. Correction: Altered activation and connectivity in a hippocampal–basal ganglia–midbrain circuit during salience processing in subjects at ultra high risk for psychosis. *Translational Psychiatry*. 2018;8(1):170. doi:10.1038/s41398-018-0189-4

692. Winton-Brown TT, Broome MR, Allen P, et al. Misattributing speech and jumping to conclusions: A longitudinal study in people at high risk of psychosis. *European Psychiatry*. 2015;30(1):32-37. doi:10.1016/j.eurpsy.2014.09.416

693. Winton-Brown T, Kumari V, Windler F, et al. Sensorimotor gating, cannabis use and the risk of psychosis. *Schizophrenia Research*. 2015;164(1-3):21-27. doi:10.1016/j.schres.2015.02.017

694. Witthaus H, Brüne M, Kaufmann C, et al. White matter abnormalities in subjects at ultra high-risk for schizophrenia and first-episode schizophrenic patients. *Schizophrenia Research*. 2008;102(1-3):141-149. doi:10.1016/j.schres.2008.03.022

695. Witthaus H, Kaufmann C, Bohner G, et al. Gray matter abnormalities in subjects at ultra-high risk for schizophrenia and first-episode schizophrenic patients compared to healthy controls. *Psychiatry Research - Neuroimaging*. 2009;173(3):163-169. doi:10.1016/j.pscychresns.2008.08.002

696. Witthaus H, Brune M, Juckel G. Hippocampal alterations in ultra-high risk patients are independent from medication and cannabis use. *Journal of Psychiatry & Neuroscience*. 2010;35(3):215.

697. Witthaus H, Mendes U, Brüne M, et al. Hippocampal subdivision and amygdalar volumes in patients in an at-risk mental state for schizophrenia. *Journal of Psychiatry and Neuroscience*. 2010;35(1):33-40. doi:10.1503/jpn.090013

698. Wittman P. Diagnostic and prognostic significance of the shut-in personality type as a prodromal factor in schizophrenia. *J Clin Psychol  4(2):*. 1948;4(2):211-214.

699. Wolwer W, Brinkmeyer J, Stroth S, et al. Neurophysiological Correlates of Impaired Facial Affect Recognition in Individuals at Risk for Schizophrenia. *Schizophrenia Bulletin*. 2012;38(5):1021-1029. doi:10.1093/schbul/sbr013

700. Wong C, Davidson L, McGlashan T, Gerson R, Malaspina D, Corcoran C. Comparable family burden in families of clinical high-risk and recent-onset psychosis patients. *Early Intervention in Psychiatry*. 2008;2(4). doi:10.1111/j.1751-7893.2008.00086.x

701. Wong C, Davidson L, Anglin D, et al. Stigma in families of individuals in early stages of psychotic illness: Family stigma and early psychosis. *Early Intervention in Psychiatry*. 2009;3(2):108-115. doi:10.1111/j.1751-7893.2009.00116.x

702. Wood SJ, Berger G, Velakoulis D, et al. Proton Magnetic Resonance Spectroscopy in First Episode Psychosis and Ultra High-Risk Individuals. In: *Schizophrenia Bulletin*. Vol 29. DHHS Public Health Service; 2003:831-843. doi:10.1093/oxfordjournals.schbul.a007049

703. WOOD SJ, PANTELIS C, PROFFITT T, et al. Spatial working memory ability is a marker of risk-for-psychosis. *Psychological Medicine*. 2003;33(7):1239-1247. doi:10.1017/S0033291703008067

704. Wood SJ, Yücel M, Velakoulis D, et al. Hippocampal and anterior cingulate morphology in subjects at ultra-high-risk for psychosis: the role of family history of psychotic illness. *Schizophrenia Research*. 2005;75(2-3):295-301. doi:10.1016/j.schres.2004.10.008

705. Wood SJ, Kennedy D, Phillips LJ, et al. Hippocampal pathology in individuals at ultra-high risk for psychosis: A multi-modal magnetic resonance study. *Neuroimage*. 2010;52(1):62-68. doi:10.1016/j.neuroimage.2010.04.012

706. Woodberry KA. *Global and Specific Neuropsychological Impairments over Time during the Putative Prodrome to Psychosis.* Dissertation Thesis. Harvard University; 2009.

707. Woodberry KA, Seidman LJ, Giuliano AJ, Verdi MB, Cook WL, McFarlane WR. Neuropsychological profiles in individuals at clinical high risk for psychosis: Relationship to psychosis and intelligence. *Schizophrenia Research*. 2010;123(2-3):188-198. doi:10.1016/j.schres.2010.06.021

708. Woodberry KA, McFarlane WR, Giuliano AJ, et al. Change in neuropsychological functioning over one year in youth at clinical high risk for psychosis. *Schizophrenia Research*. 2013;146(1-3):87-94. doi:10.1016/j.schres.2013.01.017

709. Woodberry KA, Serur RA, Hallinan SB, et al. Frequency and pattern of childhood symptom onset reported by first episode schizophrenia and clinical high risk youth. *Schizophrenia Research*. 2014;158(1-3):45-51. doi:10.1016/j.schres.2014.05.017

710. Woods SW, Breier A, Zipursky RB, et al. Randomized trial of olanzapine versus placebo in the symptomatic acute treatment of the schizophrenic prodrome. *Biological Psychiatry*. 2003;54(4):453-464. doi:10.1016/S0006-3223(03)00321-4

711. Woods SW, Tully EM, Walsh BC, et al. Aripiprazole in the treatment of the psychosis prodrome. *British Journal of Psychiatry*. 2007;191(S51):s96-s101. doi:10.1192/bjp.191.51.s96

712. Woods SW, Walsh BC, Hawkins KA, et al. Glycine treatment of the risk syndrome for psychosis: Report of two pilot studies. *European Neuropsychopharmacology*. 2013;23(8):931-940. doi:10.1016/j.euroneuro.2012.09.008

713. Woods SW, Powers AR, Taylor JH, et al. Lack of Diagnostic Pluripotentiality in Patients at Clinical High Risk for Psychosis: Specificity of Comorbidity Persistence and Search for Pluripotential Subgroups. *Schizophrenia Bulletin*. 2018;44(2):254-263. doi:10.1093/schbul/sbx138

714. Worland J. Adolescents and adults at genetic risk for psychosis. *International Journal of Rehabilitation Research*. 1979;2(4). doi:10.1097/00004356-197912000-00019

715. Worthington MA, Walker EF, Addington J, et al. Incorporating cortisol into the NAPLS2 individualized risk calculator for prediction of psychosis. *Schizophrenia Research*. 2021;227:95-100. doi:10.1016/j.schres.2020.09.022

716. Wotruba D, Heekeren K, Michels L, et al. Symptom dimensions are associated with reward processing in unmedicated persons at risk for psychosis. *Frontiers in Behavioral Neuroscience*. 2014;8. doi:10.3389/fnbeh.2014.00382

717. Wotruba D, Michels L, Buechler R, et al. Aberrant Coupling Within and Across the Default Mode, Task-Positive, and Salience Network in Subjects at Risk for Psychosis. *Schizophrenia Bulletin*. 2014;40(5):1095-1104. doi:10.1093/schbul/sbt161

718. Wu SS, Mittal V, Pennington B, Willcutt EG. Mathematics achievement scores and early psychosis in school-aged children. *Schizophrenia Research*. 2014;156(1):133-134. doi:10.1016/j.schres.2014.03.027

719. Wu J, Long X, Liu F, et al. Screening of the college students at clinical high risk for psychosis in China: a multicenter epidemiological study. *BMC Psychiatry*. 2021;21(1). doi:10.1186/s12888-021-03229-8

720. Xu Z, Mayer B, Müller M, et al. Stigma and suicidal ideation among young people at risk of psychosis after one year. *Psychiatry Research*. 2016;243:219-224. doi:10.1016/j.psychres.2016.06.041

721. Xu Z, Müller M, Heekeren K, et al. Self-labelling and stigma as predictors of attitudes towards help-seeking among people at risk of psychosis: 1-year follow-up. *European Archives of Psychiatry and Clinical Neuroscience*. 2016;266(1):79-82. doi:10.1007/s00406-015-0576-2

722. Xu L, Wang Y, Cui H, et al. Identification and prediction of clinical high risk of psychosis in Chinese outpatients using two-stage screening. *Schizophrenia Research*. 2018;202:284-290. doi:10.1016/j.schres.2018.06.026

723. Yaakub SN, Dorairaj K, Poh JS, et al. Preserved Working Memory and Altered Brain Activation in Persons at Risk for Psychosis. *American Journal of Psychiatry*. 2013;170(11):1297-1307. doi:10.1176/appi.ajp.2013.12081135

724. Yang LH, Link BG, Ben-David S, et al. Stigma related to labels and symptoms in individuals at clinical high-risk for psychosis. *Schizophrenia Research*. 2015;168(1-2):9-15. doi:10.1016/j.schres.2015.08.004

725. Yang Z, Lim K, Lam M, Keefe R, Lee J. Factor structure of the positive and negative syndrome scale (PANSS) in people at ultra high risk (UHR) for psychosis. *Schizophrenia Research*. 2018;201:85-90. doi:10.1016/j.schres.2018.05.024

726. Yassin W, Nakatani H, Zhu Y, et al. Machine-learning classification using neuroimaging data in schizophrenia, autism, ultra-high risk and first-episode psychosis. *Translational Psychiatry*. 2020;10(1). doi:10.1038/s41398-020-00965-5

727. Yee CM, Mathis KI, Sun JC, et al. Integrity of emotional and motivational states during the prodromal, first-episode, and chronic phases of schizophrenia. *Journal of Abnormal Psychology*. 2010;119(1):71-82. doi:10.1037/a0018475

728. Yee CI, Vargas T, Mittal VA, Haase CM. Adaptability and cohesion in youth at clinical high-risk for psychosis: A multi-informant approach. *Schizophrenia Research*. 2021;228:604-610. doi:10.1016/j.schres.2020.11.039

729. Yiend J, Allen P, Lopez ND, Falkenberg I, Tseng HH, McGuire P. Negative Interpretation Biases Precede the Onset of Psychosis. *Behavior Therapy*. 2019;50(4):718-731. doi:10.1016/j.beth.2018.11.002

730. Yong E, Barbato M, Penn DL, et al. Exploratory analysis of social cognition and neurocognition in individuals at clinical high risk for psychosis. *Psychiatry Research*. 2014;218(1-2):39-43. doi:10.1016/j.psychres.2014.04.003

731. Yoon YB, Yun JY, Jung WH, et al. Altered Fronto-Temporal Functional Connectivity in Individuals at Ultra-High-Risk of Developing Psychosis. *PLOS ONE*. 2015;10(8):e0135347. doi:10.1371/journal.pone.0135347

732. Yuen HP, Mackinnon A, Hartmann J, et al. Dynamic prediction of transition to psychosis using joint modelling. *Schizophrenia Research*. 2018;202:333-340. doi:10.1016/j.schres.2018.07.002

733. Yun Y, Phillips LJ, Cotton S, et al. Obstetric Complications and Transition to Psychosis in an ‘Ultra’ High Risk Sample. *Australian & New Zealand Journal of Psychiatry*. 2005;39(6):460-466. doi:10.1080/j.1440-1614.2005.01604.x

734. Yung AR, Phillips LJ, McGorry PD, et al. Prediction of psychosis. A step towards indicated prevention of schizophrenia. *Br J Psychiatry Suppl*. 1998;172(33):14-20.

735. Yung AR, Phillips LJ, McGorry PD, et al. Can we predict the onset of first-episode psychosis in a high-risk group? *International Clinical Psychopharmacology*. 1998;13:S23-S30. doi:10.1097/00004850-199801001-00005

736. Yung AR, Phillips LJ, Yuen HP, McGorry PD. Risk factors for psychosis in an ultra high-risk group: psychopathology and clinical features. *Schizophrenia Research*. 2004;67(2-3):131-142. doi:10.1016/S0920-9964(03)00192-0

737. Yung AR, Yung AR, Pan Yuen H, et al. Mapping the Onset of Psychosis: The Comprehensive Assessment of At-Risk Mental States. *Australian & New Zealand Journal of Psychiatry*. 2005;39(11-12):964-971. doi:10.1080/j.1440-1614.2005.01714.x

738. Yung AR, McGorry PD, Francey SM, et al. PACE: a specialised service for young people at risk of psychotic disorders. *Medical Journal of Australia*. 2007;187(S7). doi:10.5694/j.1326-5377.2007.tb01336.x

739. Yung AR, Nelson B, Stanford C, et al. Validation of “prodromal” criteria to detect individuals at ultra high risk of psychosis: 2 year follow-up. *Schizophrenia Research*. 2008;105(1-3):10-17. doi:10.1016/j.schres.2008.07.012

740. Yung AR, Phillips LJ, Nelson B, et al. Randomized Controlled Trial of Interventions for Young People at Ultra High Risk for Psychosis. *The Journal of Clinical Psychiatry*. 2011;72(04):430-440. doi:10.4088/JCP.08m04979ora

741. Yung AR. Is it appropriate to treat people at high‐risk of psychosis before first onset ‐ Yes. *Medical Journal of Australia*. 2012;196(9):556-556. doi:10.5694/mja12.10668

742. Yung AR, Cotter J, Wood SJ, et al. Childhood maltreatment and transition to psychotic disorder independently predict long-term functioning in young people at ultra-high risk for psychosis. *Psychological Medicine*. 2015;45(16):3453-3465. doi:10.1017/S003329171500135X

743. Yung AR, Nelson B, McGorry PD, Wood SJ, Lin A. Persistent negative symptoms in individuals at Ultra High Risk for psychosis. *Schizophrenia Research*. 2019;206:355-361. doi:10.1016/j.schres.2018.10.019

744. Zammit S. Self reported cannabis use as a risk factor for schizophrenia in Swedish conscripts of 1969: historical cohort study. *BMJ*. 2002;325(7374):1199-1199. doi:10.1136/bmj.325.7374.1199

745. Zammit S, Owen MJ, Evans J, Heron J, Lewis G. Cannabis, COMT and psychotic experiences. *British Journal of Psychiatry*. 2011;199(5):380-385. doi:10.1192/bjp.bp.111.091421

746. Zanini MA, Castro J, Cunha GR, et al. Abnormalities in sleep patterns in individuals at risk for psychosis and bipolar disorder. *Schizophrenia Research*. 2015;169(1-3):262-267. doi:10.1016/j.schres.2015.08.023

747. Zeni-Graiff M, Rizzo LB, Mansur RB, et al. Peripheral immuno-inflammatory abnormalities in ultra-high risk of developing psychosis. *Schizophrenia Research*. 2016;176(2-3):191-195. doi:10.1016/j.schres.2016.06.031

748. Zeni-Graiff M, Rios AC, Maurya PK, et al. Peripheral levels of superoxide dismutase and glutathione peroxidase in youths in ultra-high risk for psychosis: a pilot study. *CNS Spectrums*. 2019;24(03):333-337. doi:10.1017/S1092852917000803

749. Zhan C, Shi J, Mao Z, Zhao X. Perceived stress and coping style in college students with psychosis-risk syndrome. *Chinese Mental Health Journal*. 2017;12:614-618.

750. Zhang T, Li H, Woodberry KA, et al. Prodromal psychosis detection in a counseling center population in China: An epidemiological and clinical study. *Schizophrenia Research*. 2014;152(2-3):391-399. doi:10.1016/j.schres.2013.11.039

751. Zhang T, Li H, Stone WS, et al. Neuropsychological Impairment in Prodromal, First-Episode, and Chronic Psychosis: Assessing RBANS Performance. *PLOS ONE*. 2015;10(5):e0125784. doi:10.1371/journal.pone.0125784

752. Zhang T, Cui H, Tang Y, et al. Correlation of social cognition and neurocognition on psychotic outcome: a naturalistic follow-up study of subjects with attenuated psychosis syndrome. *Scientific Reports*. 2016;6(1):35017. doi:10.1038/srep35017

753. Zhang T, Tang Y, Cui H, et al. Theory of Mind Impairments in Youth at Clinical High Risk of Psychosis. *Psychiatry*. 2016;79(1):40-55. doi:10.1080/00332747.2015.1123592

754. Zhang T, Yi Z, Li H, et al. Faux pas recognition performance in a help-seeking population at clinical high risk of psychosis. *European Archives of Psychiatry and Clinical Neuroscience*. 2016;266(1):71-78. doi:10.1007/s00406-015-0615-z

755. Zhang TH, Li HJ, Woodberry KA, et al. Two-year follow-up of a Chinese sample at clinical high risk for psychosis: timeline of symptoms, help-seeking and conversion. *Epidemiology and Psychiatric Sciences*. 2017;26(3):287-298. doi:10.1017/S2045796016000184

756. Zhang Z, Zhou FC, He F, Yang NB, Zhang L, Wang CY. Cognitive function in patients with first-episode schizophrenia and individuals at high-risk for psychosis. *J Chinese Mental Health Journal* . 2017;5:345-349.

757. Zhang T, Xu L, Tang Y, et al. Isolated hallucination is less predictive than thought disorder in psychosis: Insight from a longitudinal study in a clinical population at high risk for psychosis. *Scientific Reports*. 2018;8(1):13962. doi:10.1038/s41598-018-32215-6

758. Zhang T, Xu L, Cui H, et al. Changes in correlation characteristics of time consumption and mind-reading performance in pre-onset and post-onset psychosis. *Psychiatry Research*. 2018;262:168-174. doi:10.1016/j.psychres.2018.02.008

759. Zhang T, Guo X, Tang Y, et al. Time consumption in mind-reading: A potentially important factor for social cognition assessment in early psychosis. *Schizophrenia Research*. 2018;192:491-492. doi:10.1016/j.schres.2017.06.014

760. Zhang T, Cui H, Wei Y, et al. Progressive decline of cognition during the conversion from prodrome to psychosis with a characteristic pattern of the theory of mind compensated by neurocognition. *Schizophrenia Research*. 2018;195:554-559. doi:10.1016/j.schres.2017.08.020

761. Zhang T, Xu L, Tang Y, et al. Duration of untreated prodromal symptoms in a Chinese sample at a high risk for psychosis: demographic, clinical, and outcome. *Psychological Medicine*. 2018;48(8):1274-1281. doi:10.1017/S0033291717002707

762. Zhang T, Xu L, Tang Y, et al. Using ‘WeChat’ online social networking in a real-world needs analysis of family members of youths at clinical high risk of psychosis. *Australian & New Zealand Journal of Psychiatry*. 2018;52(4):375-382. doi:10.1177/0004867417712460

763. Zhang T, Li H, Tang Y, et al. Validating the Predictive Accuracy of the NAPLS-2 Psychosis Risk Calculator in a Clinical High-Risk Sample From the SHARP (Shanghai At Risk for Psychosis) Program. *American Journal of Psychiatry*. 2018;175(9):906-908. doi:10.1176/appi.ajp.2018.18010036

764. Zhang T, Xu L, Tang Y, et al. Prediction of psychosis in prodrome: development and validation of a simple, personalized risk calculator. *Psychological Medicine*. 2019;49(12):1990-1998. doi:10.1017/S0033291718002738

765. Zhang T, Xu L, Tang Y, et al. Relationship between duration of untreated prodromal symptoms and symptomatic and functional recovery. *European Archives of Psychiatry and Clinical Neuroscience*. 2019;269(8):871-877. doi:10.1007/s00406-018-0917-z

766. Zhang T, Yang S, Xu L, et al. Poor functional recovery is better predicted than conversion in studies of outcomes of clinical high risk of psychosis: insight from SHARP. *Psychological Medicine*. 2020;50(9):1578-1584. doi:10.1017/S0033291719002174

767. Zhang T, Xu L, Tang X, et al. Real-world effectiveness of antipsychotic treatment in psychosis prevention in a 3-year cohort of 517 individuals at clinical high risk from the SHARP (ShangHai At Risk for Psychosis). *Australian & New Zealand Journal of Psychiatry*. 2020;54(7):696-706. doi:10.1177/0004867420917449

768. Zhang F, Cho KIK, Tang Y, et al. MK-Curve improves sensitivity to identify white matter alterations in clinical high risk for psychosis. *Neuroimage*. 2021;226. doi:10.1016/j.neuroimage.2020.117564

769. Zhang TH, Xu LH, Li HJ, et al. Calculating individualized risk components using a mobile app-based risk calculator for clinical high risk of psychosis: Findings from ShangHai at Risk for Psychosis (SHARP) program. *Psychological Medicine*. 2021;51(4):653-660. doi:10.1017/S003329171900360X

770. Zhang T, Wang J, Xu L, et al. Subtypes of Clinical High Risk for Psychosis that Predict Antipsychotic Effectiveness in Long-Term Remission. *Pharmacopsychiatry*. 2021;54(1):23-30. doi:10.1055/a-1252-2942

771. Zhao C, Zhu J, Liu X, et al. Structural and functional brain abnormalities in schizophrenia: A cross-sectional study at different stages of the disease. *Progress in Neuro-Psychopharmacology and Biological Psychiatry*. 2018;83:27-32. doi:10.1016/j.pnpbp.2017.12.017

772. Zhu F, Liu Y, Liu F, et al. Functional asymmetry of thalamocortical networks in subjects at ultra-high risk for psychosis and first-episode schizophrenia. *European Neuropsychopharmacology*. 2019;29(4):519-528. doi:10.1016/j.euroneuro.2019.02.006

773. Ziermans TB, Schothorst PF, Sprong M, van Engeland H. Transition and remission in adolescents at ultra-high risk for psychosis. *Schizophrenia Research*. 2011;126(1-3):58-64. doi:10.1016/j.schres.2010.10.022

774. Ziermans T, Schothorst P, Magnée M, van Engeland H, Kemner C. Reduced prepulse inhibition in adolescents at risk for psychosis: a 2-year follow-up study. *Journal of Psychiatry & Neuroscience*. 2011;36(2):127-134. doi:10.1503/jpn.100063

775. Ziermans TB, Schothorst PF, Sprong M, Magnée MJCM, van Engeland H, Kemner C. Reduced prepulse inhibition as an early vulnerability marker of the psychosis prodrome in adolescence. *Schizophrenia Research*. 2012;134(1):10-15. doi:10.1016/j.schres.2011.10.009

776. Ziermans TB, Schothorst PF, Schnack HG, et al. Progressive Structural Brain Changes During Development of Psychosis. *Schizophrenia Bulletin*. 2012;38(3):519-530. doi:10.1093/schbul/sbq113

777. Ziermans T, Wit S de, Schothorst P, et al. Neurocognitive and Clinical Predictors of Long-Term Outcome in Adolescents at Ultra-High Risk for Psychosis: A 6-Year Follow-Up. *PLoS ONE*. 2014;9(4):e93994. doi:10.1371/journal.pone.0093994

778. Zimbrón J, Ruiz de Azúa S, Khandaker GM, et al. Clinical and sociodemographic comparison of people at high-risk for psychosis and with first-episode psychosis. *Acta Psychiatrica Scandinavica*. 2013;127(3):210-216. doi:10.1111/acps.12000

779. Zimmermann R, Gschwandtner U, Wilhelm FH, Pflueger MO, Riecher-Rössler A, Fuhr P. EEG spectral power and negative symptoms in at-risk individuals predict transition to psychosis. *Schizophrenia Research*. 2010;123(2-3):208-216. doi:10.1016/j.schres.2010.08.031

780. Zipursky RB, Schulz SC. *The Early Stages of Schizophrenia* . American Psychiatric Pub; 2002.

781. Zoghbi AW, Bernanke JA, Gleichman J, et al. Schizotypal personality disorder in individuals with the Attenuated Psychosis Syndrome: Frequent co-occurrence without an increased risk for conversion to threshold psychosis. *Journal of Psychiatric Research*. 2019;114:88-92. doi:10.1016/j.jpsychires.2019.04.018

782. Zolkowska K, Cantor-Graae E, McNeil TF. Increased rates of psychosis among immigrants to Sweden: is migration a risk factor for psychosis ? *Psychological Medicine*. 2001;31(4):669-678. doi:10.1017/S0033291701003786

783. Zugman A, Pan PM, Gadelha A, et al. Brain tumor in a patient with attenuated psychosis syndrome. *Schizophrenia Research*. 2013;144(1-3):151-152. doi:10.1016/j.schres.2012.11.036

784. Zuschlag ZD, Korte JE, Hamner M. Predictors of Lifetime Suicide Attempts in Individuals With Attenuated Psychosis Syndrome. *Journal of Psychiatric Practice*. 2018;24(3):169-178. doi:10.1097/PRA.0000000000000303
